# Genetic inactivation of RIP1 kinase activity in rats protects against ischemic brain injury

**DOI:** 10.1038/s41419-021-03651-6

**Published:** 2021-04-07

**Authors:** Kimberly Stark, Tatiana Goncharov, Eugene Varfolomeev, Luke Xie, Hai Ngu, Ivan Peng, Keith R. Anderson, Erik Verschueren, Meena Choi, Donald S. Kirkpatrick, Amy Easton, Joshua D. Webster, Brent S. McKenzie, Domagoj Vucic, Baris Bingol

**Affiliations:** 1 Department of Neuroscience, Genentech, South San Francisco, 94080 CA USA; 2 Department of Early Discovery Biochemistry, Genentech, South San Francisco, 94080 CA USA; 3 Department of Biomedical Imaging, Genentech, South San Francisco, 94080 CA USA; 4 Department of Pathology, Genentech, South San Francisco, 94080 CA USA; 5 Department of Translational Immunology, Genentech, South San Francisco, 94080 CA USA; 6 Department of Molecular Biology, Genentech, South San Francisco, 94080 CA USA; 7 Department of Microchemistry, Proteomics and Lipidomics, Genentech, South San Francisco, 94080 CA USA

**Keywords:** Cell death in the nervous system, Diseases of the nervous system

## Abstract

RIP1 kinase-mediated inflammatory and cell death pathways have been implicated in the pathology of acute and chronic disorders of the nervous system. Here, we describe a novel animal model of RIP1 kinase deficiency, generated by knock-in of the kinase-inactivating RIP1(D138N) mutation in rats. Homozygous RIP1 kinase-dead (KD) rats had normal development, reproduction and did not show any gross phenotypes at baseline. However, cells derived from RIP1 KD rats displayed resistance to necroptotic cell death. In addition, RIP1 KD rats were resistant to TNF-induced systemic shock. We studied the utility of RIP1 KD rats for neurological disorders by testing the efficacy of the genetic inactivation in the transient middle cerebral artery occlusion/reperfusion model of brain injury. RIP1 KD rats were protected in this model in a battery of behavioral, imaging, and histopathological endpoints. In addition, RIP1 KD rats had reduced inflammation and accumulation of neuronal injury biomarkers. Unbiased proteomics in the plasma identified additional changes that were ameliorated by RIP1 genetic inactivation. Together these data highlight the utility of the RIP1 KD rats for target validation and biomarker studies for neurological disorders.

## Introduction

Proper regulation of cell death is critical for the development and homeostasis in metazoans. Dysregulation of cell death pathways is associated with a number of human diseases, including cancer, tissue damage/inflammation, and neurodegeneration^1,2^. The best-known form of regulated cell death is apoptosis, which relies on the activation of cysteine proteases called caspases^3^. Alternatively, necroptosis is activated when caspases are inhibited or insufficiently stimulated^4–6^. Necroptosis signaling involves activation of receptor-interacting proteins kinases 1 and 3 (RIP1 and RIP3), and pseudokinase MLKL (mixed lineage kinase-like)^7,8^. In addition to prototypical activation by TNF (tumor necrosis factor), necroptosis can be induced by several members of TNF ligand family, TLR3/4 (toll-like receptors 3/4), viral infection, and tissue damage^9,10^.

Binding of TNF to TNFR1 (TNF receptor 1) triggers the assembly of a signaling complex where ubiquitin ligases c-IAP1/2 (cellular inhibitors of apoptosis 1/2) promote ubiquitination of RIP1, themselves, and other proteins within this complex to stimulate recruitment of downstream signaling mediators and activate NF-B and MAPK (mitogen-activated protein kinases) signaling^11^. TNF can also induce cell death by stimulating the formation of cytoplasmic apoptotic and necroptotic complexes. Induction of apoptosis requires caspase-8 activation and leads to processing and activation of apoptosis effectors caspases 3 and 7^3^. However, if caspase-8 is not sufficiently activated or is inhibited, RIP1 can autophosphorylate and recruit RIP3 to form a necrosome, where RIP3 gets activated via autophosphorylation^12^. RIP3 phosphorylates MLKL, which triggers MLKL oligomerization, membrane translocation, and cellular rupture^13,14^.

RIP1 kinase activity has been shown to be instrumental in many inflammatory and neurodegenerative diseases, and in tissue damage^8,15^. For example, in animal models where NF-κB signaling is disrupted, RIP1 kinase inactivation and/or inhibition is effective in blocking inflammation and ameliorating disease severity^16–21^. Similarly, the kinase activity of RIP1 has been implicated in ischemia-reperfusion injury (IRI) and genetic inactivation or chemical inhibition of RIP1 provides great protection from IRI-associated kidney injury^22–24^. Here, we describe a genetic rat model of RIP1 kinase inactivation and show that RIP1 kinase activity drives tissue damage and inflammation in ischemic brain injury. We furthermore examine the correlates of ischemic injury in plasma proteome in the same rat model and identify changes in clotting and complement components. Overall, our results validate the value of RIP1 KD rat model and suggest that targeting RIP kinase activity in neuronal injury could be an effective strategy for the treatment of ischemic stroke.

## Materials and methods

### Animals

RIP1 D138N knock-in rats (Sprague Dawley) were generated at Genentech by CRISPR approach^25^ using the following single-guide RNA (sgRNA): 5′- TGACGAAGGTGTAATACACA-3′ (located in the RIP1 exon 4, rRIP1 ENSRNOG00000017787) and 100 ng/μl donor oligo (sequence provided in Supplementary Fig. 1A). The donor template contained the intended amino acid change D138N and one silent mutation (wt: 5′-CACAAGGAC-3′ changed to ki: 5′-CAtAAGaAC-3′, translates to HKD-HKN) that disrupted the sgRNA recognition and excision. RIP1 D138N rats were genotyped with PCR primers (1-5′-TACACAAGGACCTGAAGCC; 2-5′-AATCCAGTCAAGCCCACA and 3-5′-AGGTGTAATACATAAGAACCTGAA) yielding a 223-bp WT DNA fragment and a 230-bp mutant DNA fragment.

RIP1 D138N and WT littermate rats were housed and maintained at Genentech in accordance with American Association of Laboratory Animal Care guidelines. All experimental animal studies were conducted under the approval of the Institutional Animal Care and Use Committees of Genentech Lab Animal Research.

### Cells and reagents

Primary BMDMs were obtained from rats’ rear leg’s bone marrow and differentiated to macrophages for 6 days in DMEM medium supplemented with 10% heat-inactivated FBS, 1% non-essential amino acids, penicillin, streptomycin, 2 mM glutamine, and 25 ng/ml M-CSF at 37 °C with 5% CO_2_.

Human recombinant TNF, Nec-1, zVAD, and BV6 were all synthesized at Genentech. LPS was purchased from InvivoGen, San Diego, CA, USA and TAK1 inhibitor 5z-7-oxozeanol from Bio-Techne Corporation, Minneapolis, MN, USA.

### Lymphocyte and hematology profiling

Single cell suspensions were prepared from organs (spleen or mesenteric, axillary, brachial, and inguinal lymph nodes) of unchallenged WT or RIPK1 D138N knock-in rat littermates of 6–8 weeks old and used for analysis. Non-specific antibody binding was minimized by anti-CD16/CD32 monoclonal antibody (#14-0161-82, AB_467133, Thermo Scientific, Waltham, MA, USA), and cells were stained with fluorophore-conjugated antibodies: anti-CD45R (B220) Monoclonal Antibody (HIS24), FITC, (#11-0460-820); anti-CD4 monoclonal antibody (OX35), PE, (#12-0040-82, AB_2572548); anti-CD8a monoclonal antibody (OX8), Super Bright 600 (#63-0084-82, AB_2784849); anti-CD3 monoclonal antibody (G4.18), APC, (#17-0030-82, AB_11220081), and the following isotype controls: mouse IgG2b kappa isotype control, FITC (#11-4732-81, AB_763658); mouse IgG2a kappa isotype control, PE (#12-4724-82, AB_470064); mouse IgG1 kappa isotype control, Super Bright 600 (#63-4714-82, AB_2637448); mouse IgM isotype control, APC (#17-4752-80, AB_10670203) (Thermo Scientific). Dead cells stained by 7-AAD (7-amino-actinomycin D) (#00-6993-50, Thermo Scientific). Flow cytometry was conducted on an LSR II (BD Biosciences, San Jose, CA, USA) and analyzed with FACSDiva software. Clinical Hematology assays were run on a Sysmex XT-2000iV automated hematology analyzer.

### Viability assays

BMDMs derived from WT and RIP1 KD rats were treated with TNF (10 ng/ml), BV6 (1 μM) and zVAD (20 μM) or LPS (1 μg/ml) and zVAD (20 µM) or TNF (20 ng/ml) and TAK1 inhibitor 5z-7-oxozeanol (2.5 μM) with or without Nec1 (20 μM) overnight. Cell viability was assessed using Cell TiterGlo (Promega, Madison, WI, USA) following the manufacturer’s specifications.

### SIRS model

For TNF driven SIRS, 7–11 weeks old female rats of the indicated genotype were treated with PBS, 300 μg/kg mTNF, or 300 μg/kg mTNF + 10 mg/kg zVAD-FMK. Body temperatures were measured using Braun Thermoscan Pro 4000 thermometer.

### Western blot analysis

Cellular lysates were prepared in the following buffer: 1% Triton X-100, 25 mM Tris-HCl buffer (pH 7.5), 150 mM NaCl, 1 mM EDTA, Halt Protease, and Phosphatase Inhibitor Cocktail (Thermo Scientific). Cells were lysed on ice for 30 min and centrifuged at 14,000 rpm for 10 min at 4 °C. Proteins were resolved on SDS-PAGE and immunoblotted with the primary antibodies as described before^26^. The primary antibodies used in immunoblotting experiments were directed against: RIPK1 (#610459, AB_397832, BD Biosciences); actin (#3700, AB_2242334), pIkBɑ (#5209, AB_10829358), IkBɑ (#9242, AB_331623), HSP90 (#4874, AB_2121214) and mouse pRIP1 S166 (#31122, AB_2799000) (Cell Signaling Technology, Danvers, MA, USA).

### tMCAO model

All animal experiments were performed as specified in the license authorized by the national Animal Experiment Board of Finland (Eläinkoelautakunta, ELLA) and according to the National Institutes of Health (Bethesda, MD, USA) guidelines for the care and use of laboratory animals. In total, 50 adult male rats (25 WT and 25 RIP1 KD) were generated through intercrossing and assigned to the study at the age of ~9–10 weeks old. Animals were housed at a standard temperature (21 ± 1 °C) and in a light-controlled environment (lights on from 7 am to 8 pm) with ad libitum access to food and water. Transient focal cerebral ischemia was produced by middle cerebral artery (MCA) occlusion in right hemisphere of brain in male rats. The rats were anesthetized with 5% isoflurane (Piramal Healthcare Lot# B51D16B, in 70% N_2_O and 30% O_2_; flow 300 mL/min, Eagle Eye Anesthesia Inc. Isoflurane Tec 5 Model). During the operation, the concentration of anesthetic was reduced to 1.0–1.5%. The rectal temperature was maintained at 37.0 ± 1.5 °C with a homeothermic blanket system (Harvard Apparatus, Cambridge, MA, USA) during the surgery until the introduction of monofilament to block MCA.

After midline skin incision, the right common carotid artery (CCA) was exposed, and the external carotid artery (ECA) was ligated distal from the carotid bifurcation. Filament with silicon-covered tip (Doccol filament 4–0, dia 0,185 mm, silicon 5–6 mm/dia 0.35 mm) was inserted 22–23 mm into the internal carotid artery (ICA) up to the origin of MCA. Directly after occlusion, animals were assessed with DWI as described below.

After 90 min of ischemia, the MCA blood flow was restored by removal of the thread. The wound was closed and the animals were allowed to recover from anesthesia. The rats were carefully monitored for possible post-surgical complications after the tMCAO. Both surgical procedures (filament insertion & reperfusion) were performed altogether within 40–50 min or less. Rectal temperature was continued to be measured after occlusion, until the surgical procedure was finished. Sham animals underwent identical procedures, including anesthesia regime, but without the filament insertion and actual tMCAO.

The rats were fed with standard laboratory diet suspended in tap water on days 0–7 after the tMCAO (or as long as needed to maintain the rehydration status). To prevent dehydration, all rats were given an i.p. injection of saline (4 mL per rat) twice-a-day for 7 days if needed (or as long as needed to maintain the rehydration status). No analgesia or pain medication was used in the study in pre-or post-operative care.

### Micro-CT imaging

Animals were anesthetized with isoflurane, a midline abdominal incision was made, and a catheter was inserted into the heart. Transcardial perfusion was performed with inflow to the left ventricle and outflow from the right atrium and inferior vena cava (superior to the renal veins). Animals were perfused with PBS for 5 min at 4 ml/min and with the CT contrast agent for 5 min at 4 ml/min using a perfusion pump. A barium sulfate medium was used as the CT contrast agent (BriteVu, Scarlett Imaging, Murray, UT, USA). BriteVu is selected for its ease of use and minimal environmental toxicity. Once the contrast agent solidified, the brain was removed from the skull and immersed in 10% formalin for at least 24 h before imaging.

Imaging was performed on a Micro-CT 50 system (SCANCO Medical AG, Switzerland). Brain specimens were placed in individual holders and queued up for sequential imaging using the multi-sample carousel in the micro-CT system. The scanning parameters were: 500 projections per full rotation, 4.5 s integration time, 90 kVp photon energy, 155 μA tube current, 14 W power, 0.5 mm aluminum filter, and three averages. Images were reconstructed at 1024 × 1024 × 1200 voxels and 20 × 20 × 20 μm resolution. The number of voxels in the third dimension spanned from 1158 to 1284 depending on the length of the brain and thus affected the acquisition time (7.1 or 8.8 h). The neurovasculature was segmented using semi-automated approach in Amira (Thermo Scientific, Hillsboro, OR, USA) and then post processed using thresholding and morphological operations in MATLAB (MathWorks, Natick, MA, USA).

### Magnetic resonance imaging (MRI)

MRI acquisition for DWI and T2-weighted were performed using a horizontal 7T magnet with bore size 160 mm equipped with a gradient set capable of max. gradient strength 750 mT/m and interfaced to a Bruker Avance III console (Bruker Biospin GmbH, Ettlingen, Germany). A volume coil (Bruker Biospin GmbH, Ettlingen, Germany) was used for transmission and a two-element surface array coil for receiving (Rapid Biomedical GmbH, Rimpar, Germany). Isoflurane anesthetized rats (70% N_2_O and 30% O_2_; flow 300 ml/min, induction with 5%, maintenance 1.5%) were fixed to a head holder and positioned in the magnet bore in a standard orientation relative to gradient coils. An MR-compatible small animal monitoring system, including rectal temperature probe and pneumatic respiration sensor was used to monitor the physiology (MP150, Biopac Systems Corp., Goweta, USA). The rats were maintained at a stable body temperature of ~37 °C in the rat holder with warm water circulation system (Thermo Fischer Scientific, Loughborough, England).

### Diffusion-weighted imaging (DWI)

After placement of the filament for tMCAO, rats were recovered from the anesthesia for ~20 min, re-anesthetized and imaged by DWI at ~30 min post occlusion. Spin-echo based diffusion weighted MRI sequence with in-plane resolution of 118 m and 0.7 mm slice thickness was used to obtain whole brain apparent diffusion coefficient map in 12 min. Full imaging session was completed in under 20 min.

Diffusion change (drop in ADC of 20% or more compared to contralateral side) was used to confirm the lesion in the ipsilateral hemisphere and used as exclusion criteria to remove rats with unsuccessful MCAO from the study. Rats showing no change in DWI-MRI ipsilaterally were excluded from the study and replaced.

### T2-MRI

Lesion size, tissue viability (T2 in milliseconds) and brain edema were determined using absolute T2-MRI on days 2, 15, and 30. Multi-slice multi-echo sequence was used with the following parameters; TR = 2.5 s, 12 different echo times (10–120 ms in 10 ms steps) and 4 averages. Eighteen (18) coronal slices of thickness 1 mm were acquired using field-of-view 30 × 30 mm^2^ and 256 × 128 imaging matrix. Blinded volumetric analysis was performed manually, with lesions delineated based on the contrast differences between lesioned and healthy tissues in the ipsilateral side taking into account reference values and contralateral hemisphere as internal control.

### Behavioral testing

#### Twenty-point neuroscore

A 20-point neuroscore test was used to assess post-tMCAO motor and behavioral deficits at baseline, and on days 1, 3, 7, 14, 21, and 28 post-tMCAO. The neurological test was conducted and analyzed in a blinded manner. The following parameters were analyzed: Spontaneous circling (max. score 4), motility (max. score 3), general condition (max. score 3), paw placement on table top (max. score 4, 1 point per paw), righting reflex when placed on back (max. score 1), grip strength (max. score 2), contralateral reflex (max. score 1), visual forepaw reaching (max. score 2, 1 for each paw).

#### Limb placing test

The limb placing test was used to assess the sensorimotor integration of fore- and hind limb responses to tactile and proprioceptive stimulation at pre, and on days 1, 3, 7, 14, 21, and 28 post-tMCAO. The test had seven limb placing tasks. Each task was scored from 0 to 2 points: 2 points, the rat performs normally; 1 point, the rat performs with a delay (>2 s) and/or incompletely and 0 points, the rat does not perform normally. Both sides of the body were tested. Test was carried out and analyzed by an observer blind to the experimental groups.

#### Beam walking test

Sensorimotor functions of forelimbs and hindlimbs were tested using tapered/ledged beam by an experimenter blind to the experimental groups. The rats were pre-trained for 3 days to traverse the beam before tMCAO. The animals were tested before surgery, and on days 7 and 21. The beam-walking apparatus consists of a tapered beam with underhanging ledges on each side to permit foot faults without falling. The end of the beam was connected to a black box (20.5 cm × 25 cm × 25 cm) with a platform at the starting point. A bright light was placed above the start point to motivate the rats to traverse the beam. The rats’ performance was videotaped and was analyzed by calculating the slip ratio of the impaired (contralateral to lesion) forelimb and hindlimb (number of slips/number of total steps). Steps onto the ledge were scored as a full slip and a half slip was given if the limb touched the side of the beam. The mean of three trials was used for statistical analysis. Analysis was carried out by an observer blind to the experimental groups.

### Plasma sampling for NfL, p-NfH, and mass spectrometry analysis

At baseline and on study days 2, 15 and 30 (terminal) the rats were subjected to blood sampling via tail vein under isoflurane anesthesia. 100 µl of blood was collected into pre-chilled EDTA microtubes, immediately centrifuged at 2000 × *g* at 4 °C for 10 min, and plasma was separated and stored at −80 °C until further analysis.

### Plasma NfL and p-NfH measurements

NfL and p-NfH were measured by Simoa platform at Quanterix, Inc, Billerica, MA, using NF-Light Advantage Kit (#103186) and pNF-heavy Discovery Kit (#102669).

### Immunohistochemistry

At the end-point (D30), after plasma sampling, the rats were transcardially perfused with heparinized (2.5 IU/ml) saline in order to remove blood from the brains and tissues. After perfusion with heparinized (2.5 IU/ml) saline, mice were perfused with 4% paraformaldehyde until fixed. Thereafter, whole brains were further immersion fixed in 4% paraformaldehyde in 0.1 M phosphate buffer (PB) for another 48 h. Brains were then transferred to 0.1 M phosphate buffer (PB) and stored at 2–4 °C until analysis.

Immunohistochemistry was performed at Neuroscience Associates, Knoxville, TN, USA as described before using the following antibodies: Rabbit anti-Iba1 (Abcam, Cambridge, UK, AB178846, AB_2636859, 1:100000), mouse anti-CD68 (BioRad, Oxford, UK, MCA341GA, AB_566872, 1:1500) and rabbit anti-GFAP (Dako, Glostrup, Denmark, z0334, AB_10013382, 1:14000). Biotinylated secondary antibodies (Vector, Burlingame, CA, USA, Goat anti-rabbit IgG, BA1000, AB_2313606 and Horse anti-mouse IgG BA-2001, AB_2336180) were used at 1:1000 dilution and signal was developed using NIDAB (for anti-Iba1) or DAB (for anti-CD68 and anti-GFAP) chromogens.

Ischemia Contrast staining was performed at Neuroscience Associates using a modification of the Weil method for myelin^27^. Sections were dehydrated through alcohols, then rehydrated and stained in Hematoxylin/Ferric Ammonium Sulfate staining solution. Sections were then differentiated first in 2% ferric ammonium sulfate, rinsed in deionized water rinses, and secondly in a potassium ferricyanide/sodium borate solution. Following deionized water rinses, the slides are dehydrated in a standard alcohol series, cleared in xylene and coverslipped.

The slides were digitally scanned using a Leica SCN whole slide scanner (Buffalo Grove, IL, USA). Quantitative image analysis was performed with the MatLab software package (MathWorks). Stained or immunolabeled areas were quantified in each hemisphere and normalized based on the total hemisphere tissue area. The contralateral brain was not available for analysis in a few mice. At least 26 serial sections were analyzed per stain/ marker per mouse and final scores were based on the average of those sections.

### Mass spectrometry

#### Sample preparation

5 μl of rat plasma samples were thawed, denatured, reduced, and alkylated for 30 min at 37 °C. Subsequently, 25 μl of reduced and alkylated sample (estimated 125 μg protein) was digested using 1.22 μg of trypsin (Promega) per sample overnight at 37 °C.

#### Clean-up for mass spectrometry

Peptides were desalted using C18 96-well MACROSpinplates (The Nest Group, Southborough, MA, USA) according to the manufacturer’s instructions and dried down using a SpeedVac system. Peptides were resuspended in 40 μl LC solvent A (1% acetonitrile, 0.1% formic acid (FA)) and spiked with iRTkit calibration peptides (Biognosys, Schlieren, Switzerland) prior to mass spectrometric analyses.

#### DIA mass spectrometry acquisition

For DIA LC-MS/MS measurements, 5 μg of peptides per sample were injected to a C18 column (CSH-C18 1,7 μm, 300 μm inner diameter, 150 mm length) on a Waters M-Class LC connected to a Thermo Fisher Scientific Fusion Lumos Tribrid mass spectrometer equipped with a next gen nanoFlex electrospray source. LC solvents were A: 1% acetonitrile in water with 0.1% formic acid; B: 15% water in acetonitrile with 0.1% formic acid. The nonlinear LC gradient was 1–49% solvent B in 40 min followed by steps of 90% B for 1 min and 1% B for 4 min. A DIA method with one full range survey scan and 29 DIA windows were used.

#### DIA data analysis

DIA mass spectrometric data were analyzed using Spectronaut Pulsar X software (Biognosys). The false discovery rate on protein and peptide level was set to 1%, data were filtered using row based extraction. The assay library (protein inventory) generated in the pilot phase of this project was used for the analysis. Batch correction was performed by determining the average value of each protein in each of the two batches for a subset of runs, and then scaling all individual values for each protein based on a correction factor calculated from the ratio of mean values. The DIA measurements analyzed with Spectronaut were normalized using local regression normalization^28^.

### Experimental blinding and statistical analysis

No statistical methods were used to pre-determine sample sizes. Sample sizes were similar to previously published reports^29,30^. Animals were randomly assigned to the experimental groups. All surgery procedures were performed blinded to the genotype. All behavioral tests and quantification of MRI and immunohistochemistry images were performed blinded to the genotype and surgery groups. ROIs were drawn using automated image analysis. The following animals were excluded from the study: 1 out of 15 RIP1 KD-tMCAO animals due to unsuccessful occlusion as judged by DWI, 5 out of 15 WT-tMCAO animals and 6 out of 14 RIP1 KD-tMCAO animals due to mortality. Survival curves in SIRS and tMCAO experiments were compared by Mantel–Cox test. Normality was tested using Shapiro-Wilk test. Homogeneity of variances was tested by Levene’s test within multiple comparisons between the genotypes. Comparisons between two groups were performed by Mann–Whitney test. Comparisons between multiple genotypes and treatments over time were performed by Three-Way ANOVA (Holm-Sidak). Comparisons between multiple genotypes and treatments or comparisons between genotypes over time were performed by Two-way ANOVA (Holm-Sidak). Correlative analysis between different measurements were performed by the Spearman test. *p* < 0.05 and *p* < 0.01 were represented by * and ** symbols, respectively. Protein abundance analysis for DIA data was performed by R package MSstats v3.22.0^31^. MSstats preprocessed the normalized peak intensities from Spectronaut, quantified protein abundance using top 200 features per protein, and performed differential abundance analysis. Log2 fold-change and the standard error were estimated by linear mixed effect model for each protein. To test two-sided null hypothesis of no changes in abundance, the model-based test statistics were compared to the Student *t*-test distribution with the degree of freedom appropriate for each protein. The resulting *P* values were adjusted to control the FDR with the method by Benjamini–Hochberg. All error bars represent standard error of the mean (S.E.M.) except Fig. 5D where error bars represent standard error of the log2 fold-change.

## Results

### RIP1 kinase-dead rats are resistant to necroptosis and TNF-driven SIRS

**Fig. 1:**
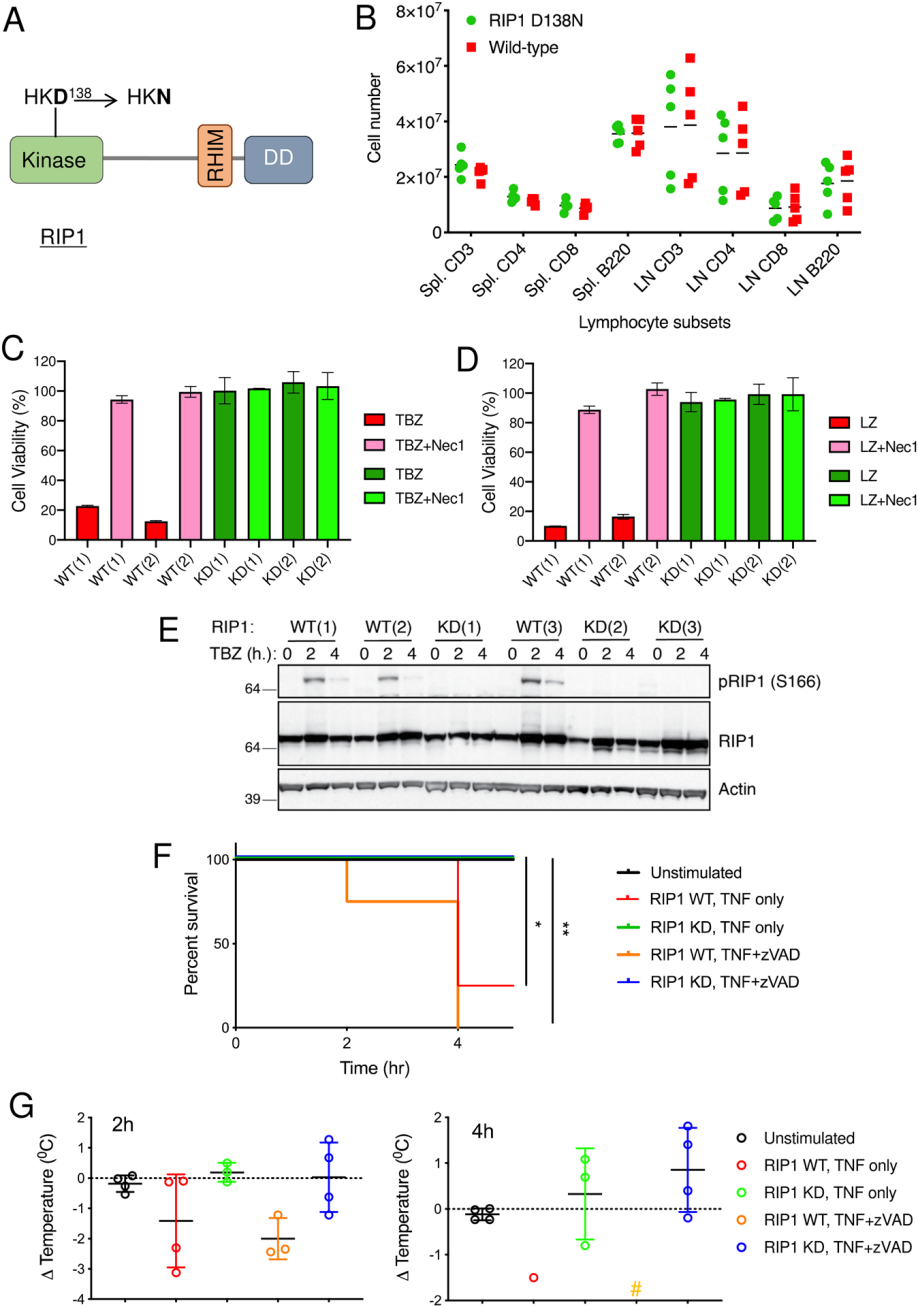
RIP1 KD rats and cells are resistant to necroptotic cell death and TNF driven hypothermia. **A** Schematic depiction of D138N kinase-inactivating mutation in rat RIP1. **B** Lymphocyte subsets from spleen (Spl) and lymph nodes (LN) of RIP1 KD and WT rats. Data from individual rats of each genotype (*n* = 5) are plotted, with the mean value for each group indicated by a horizontal line. There were no significant differences between two genotypes as evaluated by unpaired *t*-test (*p* > 0.1). **C**, **D** BMDMs derived from WT and RIP1 KD rats were treated with TNF, BV6, and zVAD (TBZ) (**C**) or LPS and zVAD (LZ) (**D**) with or without Nec1 overnight. Cell viability was assessed by Cell Titer-Glo assay. Numbers in parentheses indicate that cells were derived from different animals. **E** BMDMs derived from WT and RIP1 KD rats were treated with TBZ for indicated periods of time. Cellular lysates were immunoblotted with the indicated antibodies. Numbers in parentheses indicate that cells were derived from different animals. **F**, **G** WT and KD RIPK1 rats were treated with TNF (300 µg/kg) and zVAD 10 mg/kg or PBS (unstimulated) for 4 h. Survival (**F**) and body temperatures (**G**) were evaluated 2 and 4 h after treatment. *p < 0.05 and **p < 0.01 between WT and RIP1 KD by Mantel–Cox test. # in (**G**) indicates that no animals from this group survived to this time point. Data are represented as mean ± S.E.M.

Genetic inactivation of RIP1 in mice has proved extremely valuable for investigating the role of RIP1 kinase activity in disease models in vivo^17,22^. To examine the effect of RIP1 genetic inactivation in additional animal models, we generated Rip1 knock-in rats by mutating Asp 138 of the HKD RIP1 kinase motif to Asn (Fig. 1A and Supplementary Fig. S1A). Homozygous *Rip1 (D138N)* rats (referred to as “RIP1 KD” hereafter) were viable and reproduced normally. Heterozygous RIP1 WT/KD mice were bred to yield offspring at the Mendelian frequency (27 WT/WT, 52 WT/KD, 21 KD/KD). Histopathological characterization of RIP1 KD rats revealed no genotype-specific findings compared to WT rats at baseline in the pancreas, liver, small intestine, large intestine, thymus, lymph nodes, spleen, lung, heart, and kidney. Minimal histologic findings observed in both WT and RIP1 KD rats were consistent with expected age-related findings. Lymphocyte numbers in spleen and lymph nodes of RIP1 KD rats and numbers of various cell types, platelets, and other blood parameters were indistinguishable from WT rats as well (Fig. 1B, Supplementary Fig. S1B). In addition, bone marrow-derived macrophages (BMDMs) from RIP1 KD and WT rats activated NF-B signaling in response to TNF in a comparable manner (Supplementary Fig. S1C).

In agreement with a crucial role for the kinase activity of RIP1 in TNF-induced necroptosis, RIP1 KD BMDMs were completely resistant to killing by TNF/BV6/zVAD (TBZ; Fig. 1C). Similarly, BMDMs from RIP1 KD rats were also resistant to LPS/zVAD mediated necroptotic cell death (LZ; Fig. 1D) or apoptosis triggered by TNF and TAK1 inhibitor (T/5z-7; Supplementary Fig. S1D). RIP1 KD cells expressed comparable levels of RIP1 to WT cells, but showed no TBZ-induced RIP1 phosphorylation, consistent with their resistance to necroptotic cell death (Fig. 1E). RIP1 KD rats were also fully resistant to systemic shock caused by administration of TNF or TNF/zVAD as they all survived the treatment and exhibiting no hypothermia (Fig. 1F, G). Overall, these data suggest that inhibiting the kinase activity of RIP1 in rats has no deleterious effects and that RIP1 KD rats can be used to examine the contribution of RIP1 to animal models of human diseases.

### Genetic inactivation of RIP1 kinase activity provides protection against ischemic brain injury

**Fig. 2:**
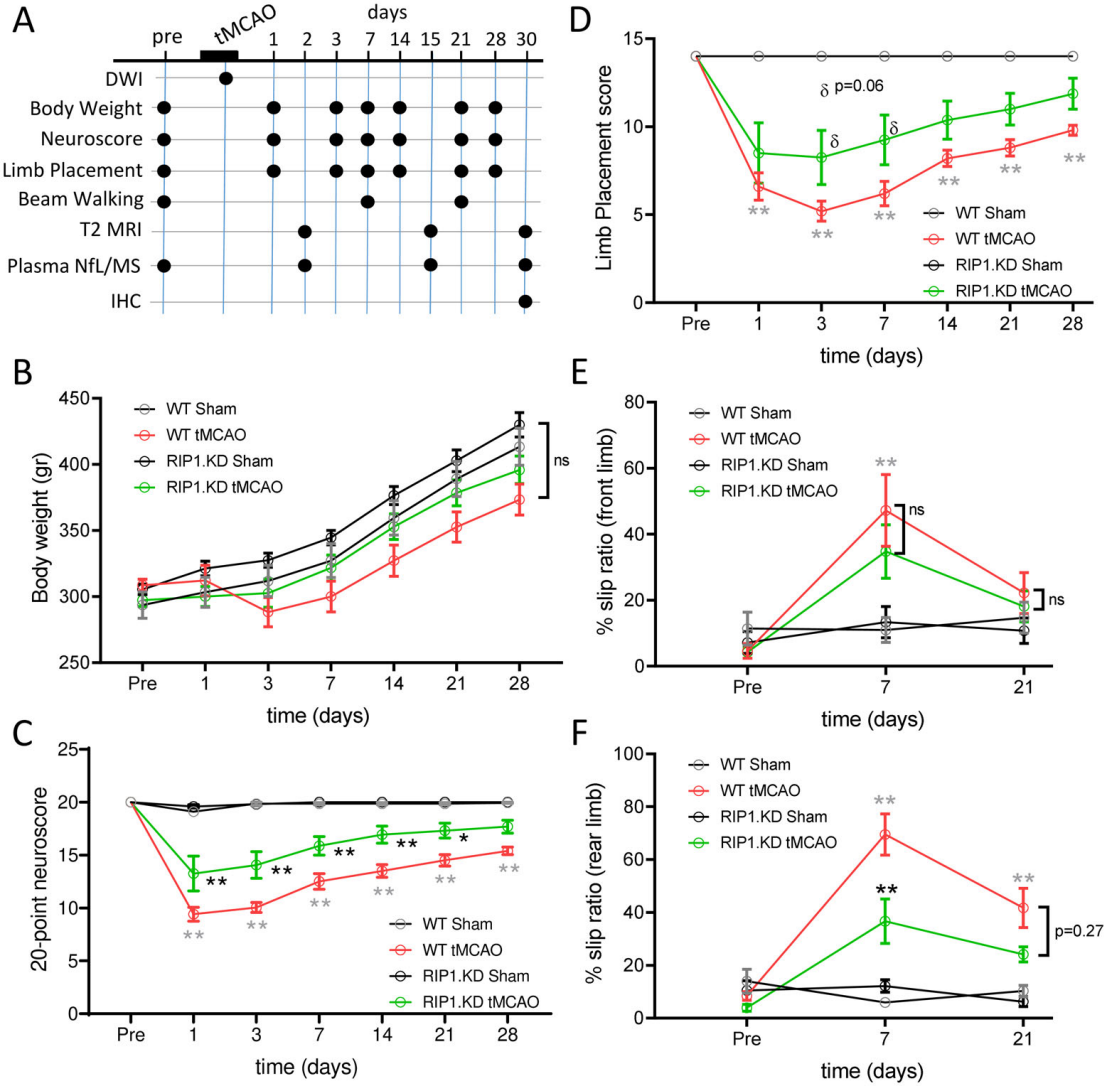
Genetic inactivation of RIP1 kinase activity ameliorates neurological function following ischemic brain injury. **A** Experimental timeline and endpoints in the tMCAO model. **B** Body weights of WT and RIP1 KD rats before and after sham or tMCAO surgery. **C**–**F** Behavioral scoring of WT and RIP1 KD rats before and after sham or tMCAO surgery. 20-point neuroscore (**C**), limb placing (**D**), and beam walking (forepaw (**E**) and hindpaw (**F**)) tests were performed. *n* = 8–10 rats/group. At the indicated time points, **p < 0.01 between WT-tMCAO and WT-Sham (in gray), and *p < 0.05 and **p < 0.01 between RIP1 KD-tMCAO and WT-tMCAO (in black) by Three-Way ANOVA (Holm-Sidak). Data are represented as mean ± S.E.M.

Previous studies have indicated that pharmacological inhibition of RIP1 kinase activity in mice^32–37^ or rats^33,38–43^, and genetic inhibition in mice^29,30^ ameliorate cell death and inflammation associated with ischemic brain injury. To test if RIP1 KD rats are similarly protected, we utilized the transient middle cerebral artery occlusion/reperfusion model (tMCAO)^44^. Since variations in the cerebrovascular anatomy^45^ and cerebral collateral circulation^46^ can contribute to variability in preclinical occlusion models, we compared the cerebrovascular anatomy between the WT and RIP1 KD rats using micro-CT imaging (Supplementary Fig. 2A). The analysis of vasculature morphology parameters did not reveal differences between the two genotypes (Supplementary Fig. 2B), indicating that RIP1 KD rats have similar vascular anatomy to WT rats and provide a suitable model for testing the efficacy of RIP1 kinase inactivation in the tMCAO model. We induced MCAO in the right hemisphere in male WT or RIP1 KD rats by inserting a filament into the internal carotid artery. Directly after occlusion, diffusion-weighted imaging (DWI) was used to confirm the lesion (rats displaying a drop in apparent diffusion coefficient of 20% or more compared to the contralateral side were included in the study). Post-hoc quantification of lesions in DWI images did not show a significant difference between WT and RIP1 KD rats (Supplementary Fig. S2C, D). After 90 min of occlusion, MCA blood flow was restored by removal of the filament and the animals were assessed by a battery of imaging and functional tests for up to 30 days, followed by histopathological analysis (Fig. 2A). Sham control animals received identical procedures as the tMCAO group except filament insertion and removal.

The mortality rates in tMCAO groups were ~33% and 40% in WT and RIP1 KD rats, respectively, and were not significantly different between the genotypes (*p* = 0.29). Throughout the study, no significant differences were observed between the experimental groups in their body weights, though tMCAO-operated WT rats (WT-tMCAO) had a transient reduction in their body weights at day 3 post-tMCAO (Fig. 2B). We evaluated behavioral deficits using a general neurological assessment score at baseline and on days 1, 3, 7, 14, 21, and 28 post-tMCAO. As expected, WT-tMCAO rats had a ~50% reduction in overall performance scores as compared to sham-operated rats (WT-Sham) (Fig. 2C). Importantly, RIP1 KD rats that had undergone tMCAO (RIP1 KD-tMCAO) were partially protected, and significantly performed better than WT-tMCAO rats. Similar functional benefits in RIP1 KD rats were also evident in additional tests measuring sensorimotor integration of limb responses to stimulation (limb placing test) and motor coordination (beam walking test) (Fig. 2D–F). These data indicate RIP1 kinase activity contributes to the functional deficits that follow ischemic brain injury.

**Fig. 3:**
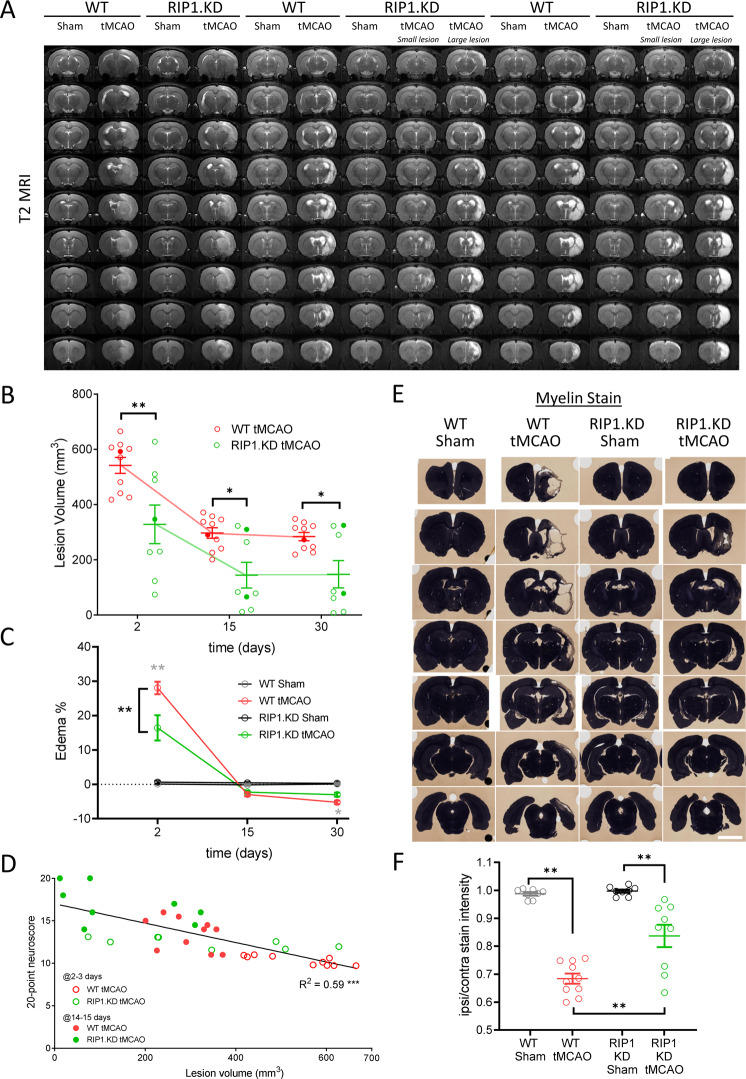
Genetic inactivation of RIP1 kinase activity preserves tissue integrity following ischemic brain injury. **A** T2-MRI imaging in WT and RIP1 KD rats following tMCAO surgery. Representative images at days 2-, 15-, and 30 post-tMCAO are shown. In groups where there are two clusters with respect to lesion sizes (as in RIP1 KD-tMCAO 15 and 30 days post-tMCAO, **B**), representative images from each cluster are separately shown. **B** Quantification of lesion size based on blinded volumetric analysis of T2-relaxation time, from (**A**) (longer time correlates with severity of the lesion). The closed symbols correspond to the animals with T2-MRI images shown in (**A**). *n* = 8–10 rats/group. *p < 0.05 and **p < 0.01 by Two-way ANOVA (Holm-Sidak). **C** Quantification of tissue percent area with edema, from (**A**). *n* = 8–10 rats/group. At the indicated time points, **p < 0.01 between WT-tMCAO and WT-Sham (in gray), and between RIP1 KD-tMCAO and WT-tMCAO (in black) by Three-way ANOVA (Holm-Sidak). **D** Correlation between 20-point neuroscore and lesion volume at 2–3 days (open circles) and 14–15 days (closed circles) post-tMCAO, from (**B**) and Fig. 2C. **p < 0.01 by Spearman test. **E** Immunohistochemistry by a myelin stain in WT and RIP1 KD rats at 30-days post-tMCAO. Representative images across the brain are shown. Darker color highlights the intact tissue. Scale bar, 5 mm. **F** Quantification of the ratio of ipsilateral to contralateral myelin stain intensity, from (**E**). *n* = 8–10 rats/group. **p < 0.01 by Two-way ANOVA (Holm-Sidak). Data are represented as mean ± S.E.M.

In addition to the behavioral endpoints, we measured the tissue viability and extent of edema using absolute T2-MRI on days 2, 15, and 30 post-tMCAO. Both WT and RIP1 KD rats displayed significant lesions at day 2 post-tMCAO that resolved further at 15- and 30-days post-tMCAO (Fig. 3A, B. Closed symbols in Fig. 3B indicate the animals shown in Fig. 3A). Importantly, the lesion size in RIP1 KD-tMCAO rats was significantly smaller than WT-tMCAO rats at all time points examined (Fig. 3A, B). At time points beyond 2-days post-tMCAO, lesion size in RIP1 KD-tMCAO rats separated into two groups with larger and smaller lesion sizes as highlighted in Fig. 3A, B. Edema was observed only at day 2 post-tMCAO and was similarly smaller in size in RIP1 KD animals (Fig. 3C). On a per animal basis, a strong correlation was observed between lesion size versus the neurological functional scores (Fig. 3D). At the end of the in-life portion of the study, the rats were sacrificed at 30 days post-tMCAO, and perfused and stained with a myelin-reactive dye as a surrogate for tissue viability^47^ (Fig. 3E). This histochemistry method further confirmed the protection of tissue integrity in RIP1 KD-tMCAO rats compared to the WT-tMCAO rats (Fig. 3E, F). Together these data show genetic inactivation of RIP1 kinase activity promotes tissue integrity following ischemic brain injury.

### Genetic inactivation of RIP1 kinase activity reduces inflammation and neuronal injury associated with brain ischemia

**Fig. 4:**
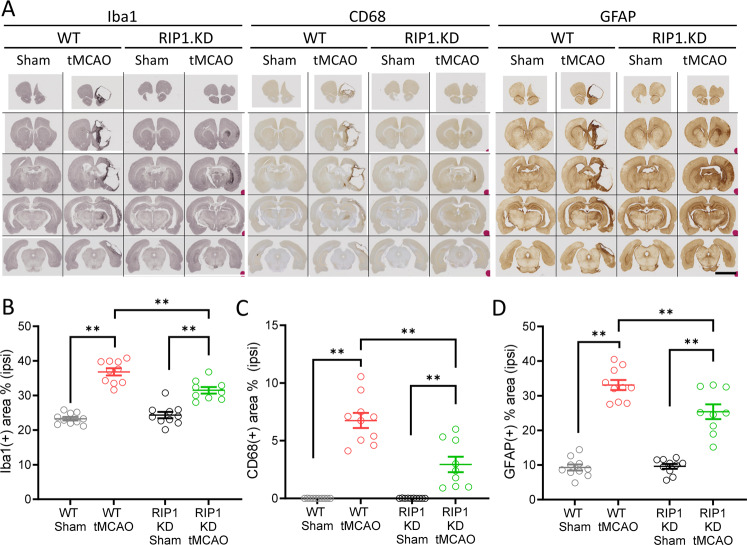
Genetic inactivation of RIP1 kinase activity reduces inflammation following ischemic brain injury. **A** Immunohistochemistry for Iba1, CD68, and GFAP in WT and RIP1 KD rats at 30-days post-tMCAO. Representative images across the brain are shown. Scale bar, 5 mm. **B**–**D** Quantification of percent tissue area positive for high-intensity Iba1 (**B**), CD68 (**C**) and GFAP (**D**) in the ipsilateral side, from (**A**). *n* = 8–10 rats/group. **p < 0.01 by Two-way ANOVA (Holm-Sidak). Data are represented as mean ± S.E.M.

RIP1 kinase activity has been proposed to drive neuroinflammation in acute and chronic neurological disorders^8^. To examine if RIP1 kinase inactivation altered neuroinflammation, we immunostained WT and RIP1 KD rat brains for Iba1 and CD68 to measure microgliosis and for GFAP to measure astrogliosis (Fig. 4A). As expected, WT-tMCAO rats displayed increased Iba1, CD68, and GFAP signal most prominently near the lesion at the ipsilateral hemisphere (Fig. 4A–D). Importantly, RIP1 KD-tMCAO rats had significantly less accumulation of these markers (Fig. 4A–D), consistent with the overall protection of tissue integrity in RIP1 KD rats.

**Fig. 5:**
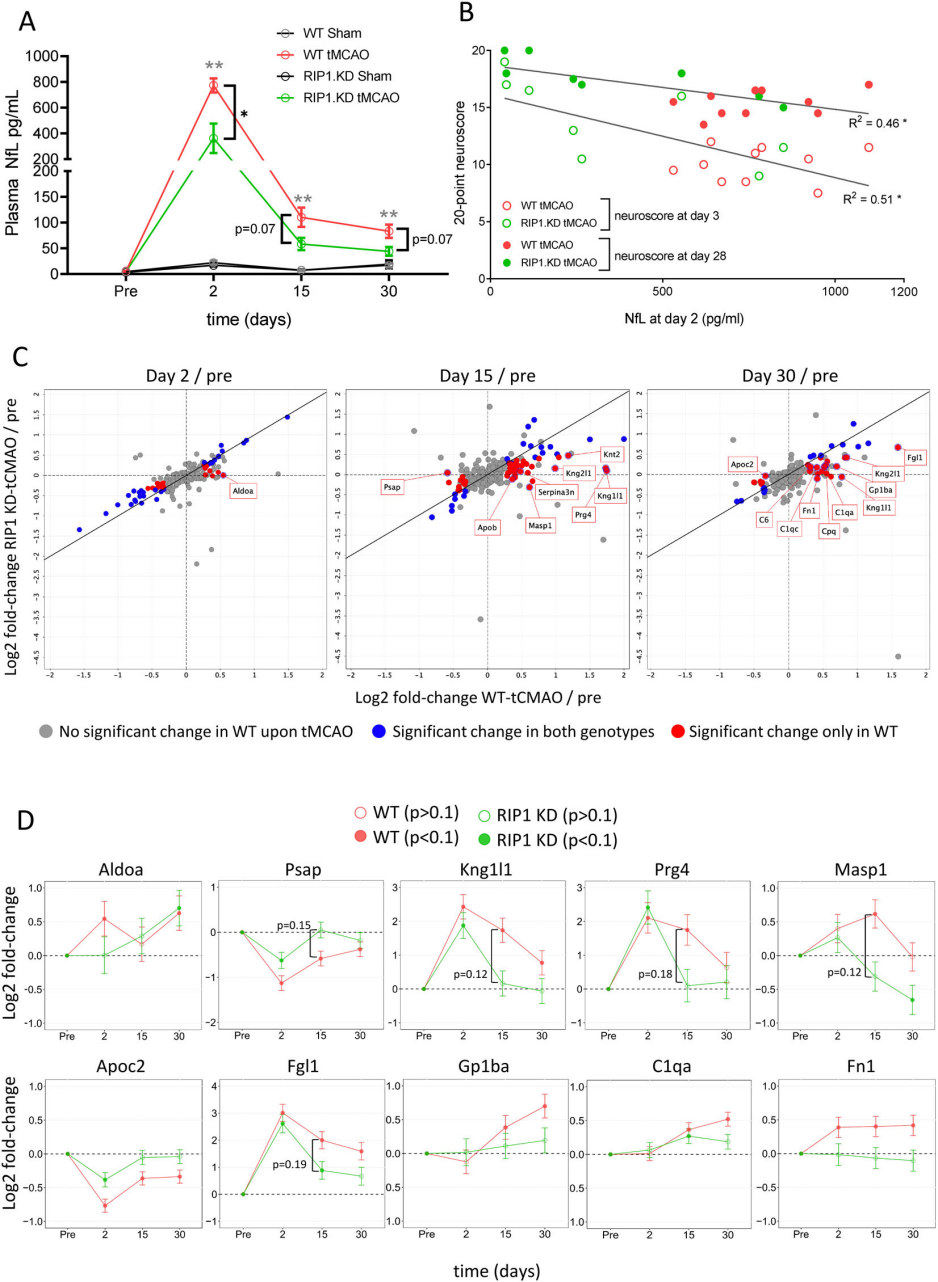
Genetic inactivation of RIP1 kinase activity reduces plasma NfL and additional markers of injury following ischemic brain injury. **A** Plasma NfL levels in WT and RIP1 KD rats before and 2-, 15-, and 30-days after sham or tMCAO surgery. *n* = 8–10 rats/group. At the indicated time points, **p < 0.01 between WT-tMCAO and WT-Sham (in gray), and *p < 0.05 and *p* = 0.07 between RIP1 KD-tMCAO and WT-tMCAO (in black) by Two-way ANOVA (Holm-Sidak). Data are represented as mean ± S.E.M. **B** Correlation between plasma NfL levels at 2 days versus 20-point neuroscore at 3 (open circles) and 28 days (closed circles) post-tMCAO, from (**A**) and Fig. 2C. *p < 0.05 by Spearman test. **C** Plasma protein profiling by mass spectrometry in WT and RIP1 KD rats before and 2-, 15-, and 30-days after sham or tMCAO surgery. *n* = 10 rats/group. Each graph represents log2 fold-change in plasma protein abundance at the indicated time points, post-surgery compared to pre-surgery, in WT (*x*-axes) and RIP1 KD (*y*-axes) rats. Proteins that show significant difference in abundance upon sham surgery in WT animals (>1.2 fold-change, adjusted *p*-value < 0.1, open symbols in Supplementary Fig. 3B) are excluded from the graphs. Symbol colors represent changes in plasma protein abundance according to the genotypes such that blue symbols represent significant change in both WT and RIP1 KD plasma, red symbols represent significant change only in WT plasma, and gray symbols represent no significant change in WT plasma. **D** Graphs representing the log2 fold-change in plasma protein abundance of the indicated proteins compared to baseline levels in WT and RIP1 KD rats. Symbol colors represent WT or RIP1 KD rats. Symbol types, closed or open, represent statistical significance as indicated. Error bars represent standard error of the log2 fold change.

We next examined the abundance of Neurofilament-L (NfL) in plasma of WT and RIP1 KD rats. NfL is an axonal protein released from injured neurons and serves as a marker of neuronal injury in neurological disorders^48^. Importantly, plasma NfL levels may reflect the ongoing neuronal injury and predict functional outcomes in ischemic stroke patients^49–57^. Plasma NfL levels were increased at day 2 post-tMCAO and returned towards baseline levels thereafter (but remained elevated even at day 30 post-tMCAO) (Fig. 5A). Importantly, RIP1 KD-tMCAO rats had ~2.5-fold reduction in plasma NfL levels compared to WT-tMCAO rats at day 2 post-tMCAO (Fig. 5A). At later time points, plasma NfL levels remained lower in the RIP1 KD rats compared to WT animals without reaching statistical significance (Fig. 5A). A related neuronal injury marker, phospho-Neurofilament-H (p-NfH), also displayed a similar trend (Supplementary Fig. 3A). We additionally examined if the plasma NfL level correlates with functional outcomes and found a significant correlation between the increased NfL levels measured at day 2 post-tMCAO and neurological functional scores measured at day 3 or day 28 (Fig. 5B).

### Unbiased measurements of plasma proteome in tMCAO rats

We next measured changes in the plasma proteome in an unbiased manner using data-independent acquisition (DIA) mass spectrometry based-proteomics in WT and RIP1 KD rats. Plasma samples collected before tMCAO and at days 2-, 15-, and 30 post-tMCAO were analyzed using DIA mass spectrometry, which led to the quantification of the relative abundance of 275 proteins represented by 14,604 peptides across the groups. To further analyze the identified proteins, we first filtered out proteins that showed a significant change in their abundance (fold-change >1.2, adjusted *p* < 0.1) upon sham surgery in WT animals compared to baseline levels (Supplementary Fig. 3B, open symbols. Supplementary Table 1; 85, 45, and 79 proteins at days 2, 15, and 30 post-sham surgery, respectively). Among the remaining proteins, 54, 67, and 38 proteins showed differential abundance in plasma in WT-tMCAO rats compared to their baseline levels at days 2, 15, and 30 post-tMCAO, respectively (Supplementary Fig. 3B, closed red symbols. Supplementary Table 1). Pathway analysis of plasma proteins specifically upregulated upon tMCAO in WT rats highlighted members of the complement pathway as well as the blood clotting cascades (Supplementary Fig. 3B, Supplementary Table 2). On the other hand, downregulated proteins belonged to lipoprotein assembly, remodeling, and clearance pathways (Supplementary Fig. 3B, Supplementary Table 2).

Further focusing on genotype-dependent effects, we compared the plasma proteome in tMCAO groups between the WT and RIP1 KD rats. Majority of the plasma proteins had similar changes in WT and RIP1 KD rats throughout the time course following tMCAO (Supplementary Tables 1 and 3), and none of the proteins examined showed significant differences between the genotypes after multiple comparison corrections (adjusted *p* < 0.1). However, out of the proteins that showed differential abundance in WT-tMCAO plasma (Supplementary Fig. 3B), around half of them were not significantly different in RIP1 KD-tMCAO plasma compared to their baseline levels (21/54, 46/67, and 24/38 proteins at days 2, 15, and 30 post-tMCAO, respectively. Figure 5C, red symbols: proteins with significant change in only WT-tMCAO rats. Blue symbols: proteins with significant change in both WT-tMCAO and RIP1 KD-tMCAO rats). Pathway analysis of proteins that did not change in RIP1 KD rats indicated that they belonged to platelet degranulation (e.g. Orm1, Psap, Kng1l1, Aldoa), complement (e.g., C1q, C5, Masp1) and other pathways (Fig. 5C, Supplementary Tables 1, 2). We highlight the most differentially-regulated proteins between the two genotypes in Fig. 5C, D. Together, these data indicate additional markers impacted by the RIP1 genetic inactivation that can be measured in the periphery.

## Discussion

Genetic inactivation of RIP1 kinase activity in mice has been instrumental in defining physiological conditions and diseases where RIP1 kinase function plays an important role^17,18,20,22,58^. These studies have also paved the way for therapeutic targeting of RIP1 in patients with various inflammatory and neurological diseases^59,60^. In this study, we have introduced a genetic inactivation of RIP1 kinase function in rats. Similar to RIP1 kinase-dead (KD) mice^18,58^, RIP1 KD rats showed no genotype-related abnormalities and their immune and hematology parameters, and NF-κB signaling profile mirrored wild-type rats. However, RIP1 KD rats were completely resistant to TNF-induced systemic shock and cells derived from RIP1 KD rats were resistant to RIP1 mediated necroptotic and apoptotic cell death. These data validate rats with genetically inactivated RIP1 kinase as a model for testing RIP1 function in animal disease models, especially those where the larger size of rats and their robust immune response is more advantageous for complex manipulations^61^. Given the broad usability of rats in preclinical efficacy and safety studies^61,62^, we believe that RIP1 KD rats will provide a valuable tool for studying and validating RIP1 as a target in numerous diseases.

RIP1 KD rats were protected in the tMCAO model highlighting the utility of RIP1 KD rats in target validation for neurological disorders. The beneficial effect of RIP1 kinase inactivation was evident even at the earliest time points post-injury. This early beneficial effect is most obvious in the longitudinal data measuring neurological function where a significant benefit in RIP1 KDs were observed even at 1-day post-tMCAO. Beyond the initial benefit in functional outcomes, both WT and RIP1 KD rats recover with similar rates over the time course of the experiment suggesting the effect of RIP1 kinase activation may be most related to the onset of ischemic injury. Consistent with this view, recent studies in mice indicate rapid activation of RIP1 (as judged by its autophosphorylation) as early as 1 h post-injury^30^. In rats, pharmacological inhibition of RIP1 similarly improves neurological scores measured at 12 h post-tMCAO^43^. Interestingly, in addition to neurons, endothelial cells upregulate markers of necroptosis within 2 h post-reperfusion^30^ via activation of TNFR1 signaling^63^. Early activation of RIP1 kinase in neurons and endothelial cells were also observed following intracerebral hemorrhage^64^. Future studies could focus on the conditional inactivation of RIP1 kinase activity to decipher the role of RIP1 in specific cell types during ischemic injury. In addition, given the early RIP1 pathway activation following ischemic/reperfusion insult, it would be critical to test the efficacy of RIP1 kinase inhibition in the therapeutic setting in animal models.

In addition to the reduced brain lesion, RIP1 KD animals had reduced accumulation of the plasma marker of neuronal injury, NfL. In the rat tMCAO model, NfL accumulation was transient with higher levels at day 2 post-injury compared to days 15 and 30 post-injury. This time course of plasma NfL concentration in the rat model is different than reported for ischemic stroke patients where plasma NfL concentration is highest between ~7 and 21 days and can remain elevated at least for ~6-months^50,52,57,65,66^. This difference is likely due to the large lesion size induced via ad hoc reperfusion in rodent tMCAO models compared to partial spontaneous reperfusion in humans with comparatively small infarcts^67,68^. Regardless, plasma NfL levels in rats remained elevated at least for 30 days post-tMCAO, potentially reflecting a combination of high stability of plasma NfL (~3 weeks in mouse^69^) as well as the ongoing neurodegeneration in the ischemic brain.

In addition to NfL, unbiased proteomics of the plasma samples identified proteins whose abundance were differentially regulated upon tMCAO and potentially between WT and RIP1 KD rats following ischemic brain surgery. Among the differentially-regulated proteins, RIP1 KD-tMCAO rats had a trend towards reduced activation of the blood clotting and complement pathways. Interestingly, the protection afforded by RIP1 kinase inhibition in the SIRS model also involves the rescue of the activation of the clotting cascade and the increased vascular permeability in the endothelial compartment^70^. Similarly, a recent plasma profiling study in ischemic stroke patients also highlighted the upregulation of blood clotting pathways as a diagnostic biomarker^71^. Related to the complement and similar to the ischemic injury, endothelial cell damage in autoimmune disorders can involve RIP1 kinase-dependent activation of the complement^72^. Interestingly, the complement may in turn activate RIP1-dependent necroptosis and mediate cellular toxicity via RIP1 kinase activity^73^. Since RIP1 kinase acts early in the pathological cascade following ischemic injury, it remains to be determined whether the RIP1-dependent changes in the plasma proteome reflect amelioration of the disease process rather than a specific molecular involvement of RIP1. Regardless, these data together highlight the utility of the RIP1 KD rat model for validation of targets and potential biomarkers for nervous system diseases.

## Supplementary information

Supplementary Materials Inventory

Supplementary Figures

Supplementary Figure and Table Legends

Supplementary Table 1

Supplementary Table 2

Supplementary Table 3

## Data Availability

Proteomics data, database search results, quantification data, R script, and result for statistical analysis have been deposited to the ProteomeXchange Consortium via the MassIVE partner repository with the dataset identifier MSV000086623 (or PXD023255).
