# Supplementary material for: Genetic inactivation of RIP1 kinase activity in rats protects against ischemic brain injury
Source: Cell Death Dis. 2021 Apr 7;12(4):379. doi: 10.1038/s41419-021-03651-6 (PMC8026634; doi:10.1038/s41419-021-03651-6)
Supplement: Supplementary file 1 — Supplementary Materials Inventory [file 41419_2021_3651_MOESM1_ESM.pdf]

Supplementary Materials Inventory

Supplementary Figure and Table Legends (.doc file)

Supplementary Figures S1-3 (.pdf file)

Supplementary Table 1 (.xls file)

Supplementary Table 2 (.xls file)

Supplementary Table 3 (.xls file)
