# Supplementary material for: Genetic inactivation of RIP1 kinase activity in rats protects against ischemic brain injury
Source: Cell Death Dis. 2021 Apr 7;12(4):379. doi: 10.1038/s41419-021-03651-6 (PMC8026634; doi:10.1038/s41419-021-03651-6)
Supplement: Supplementary file 2 — Supplementary Figures [file 41419_2021_3651_MOESM2_ESM.pdf]

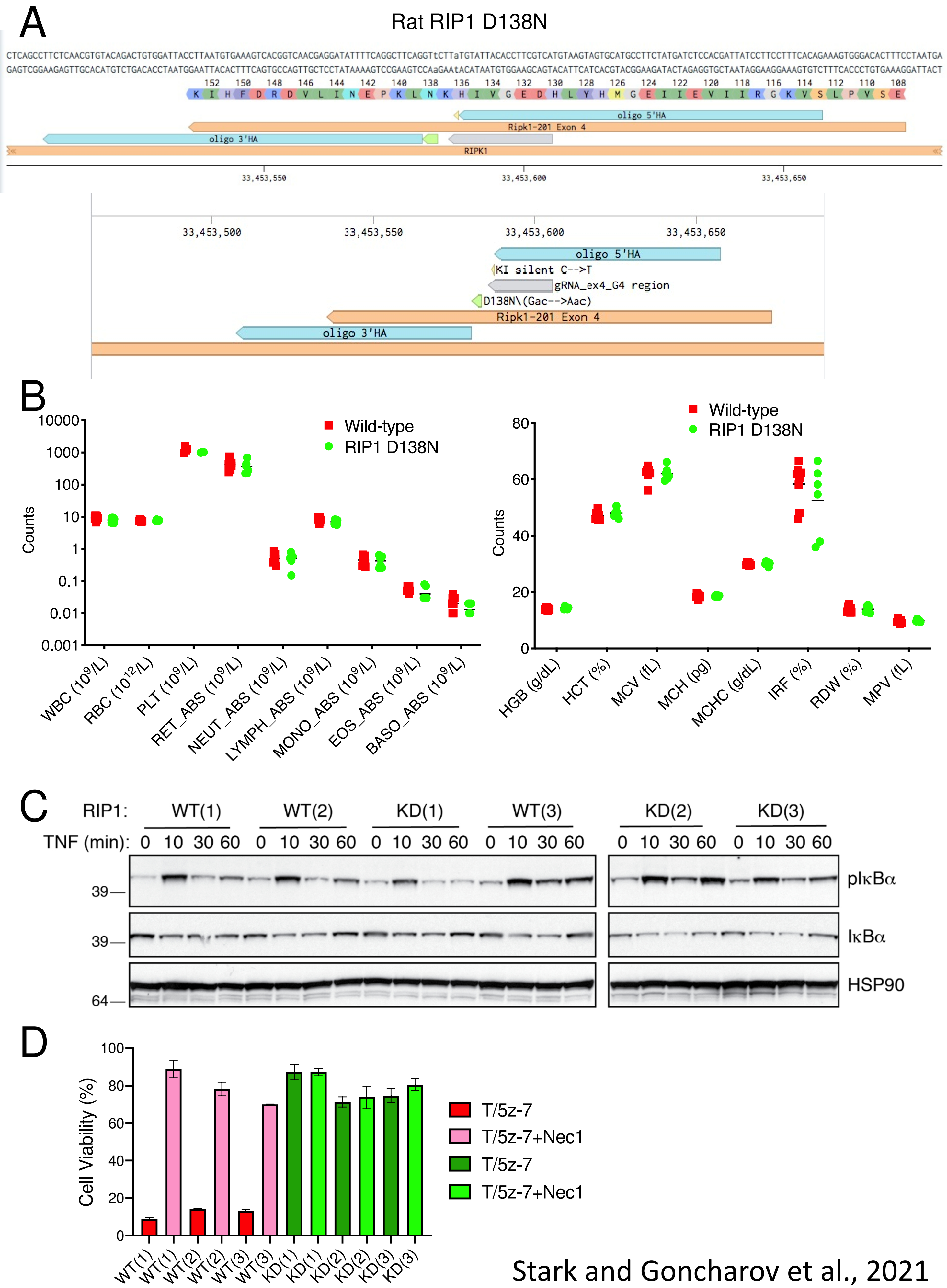

Stark and Goncharov et al., 2021
Supplementary Figure 1

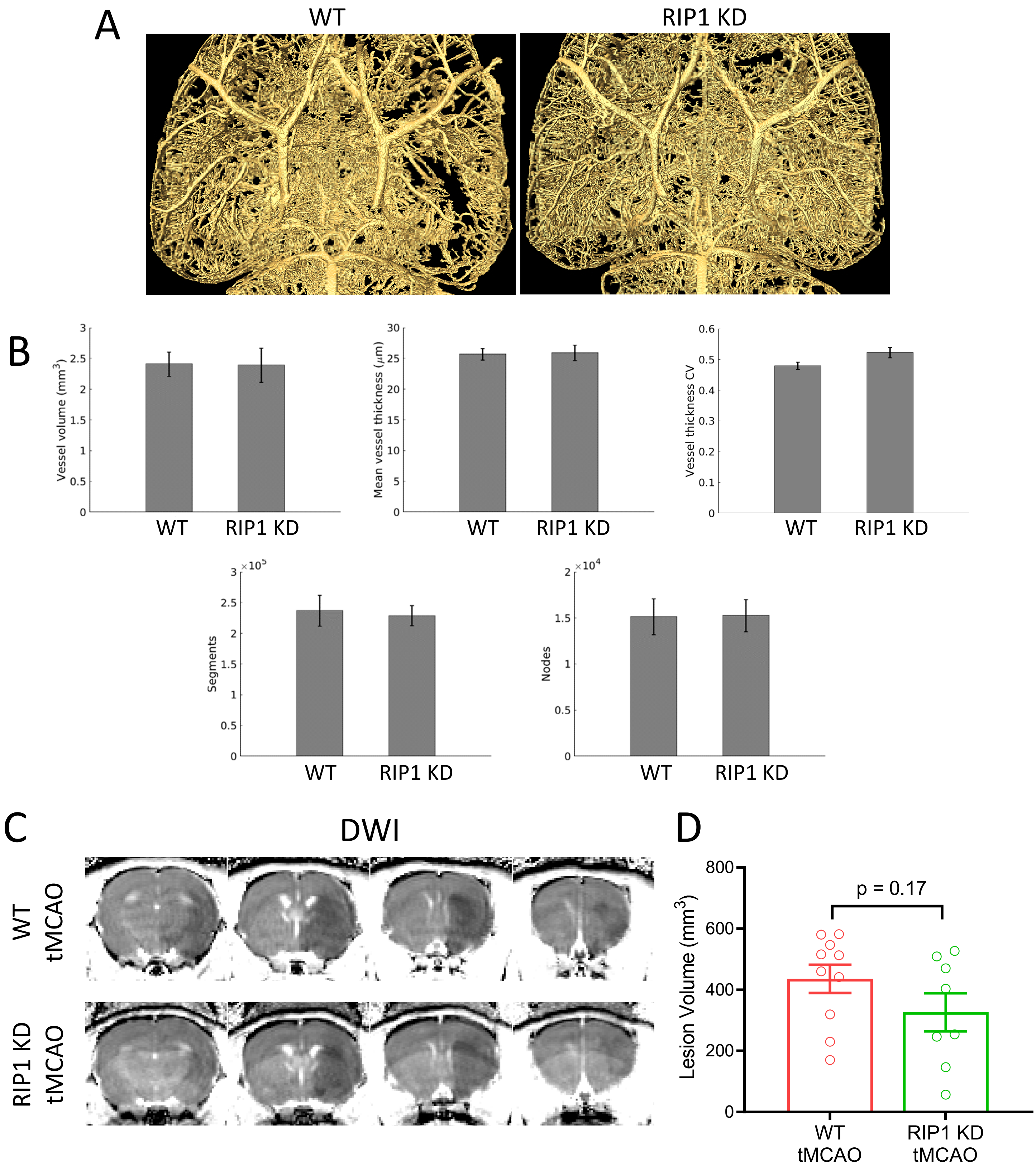

Stark and Goncharov et al., 2021
Supplementary Figure 2

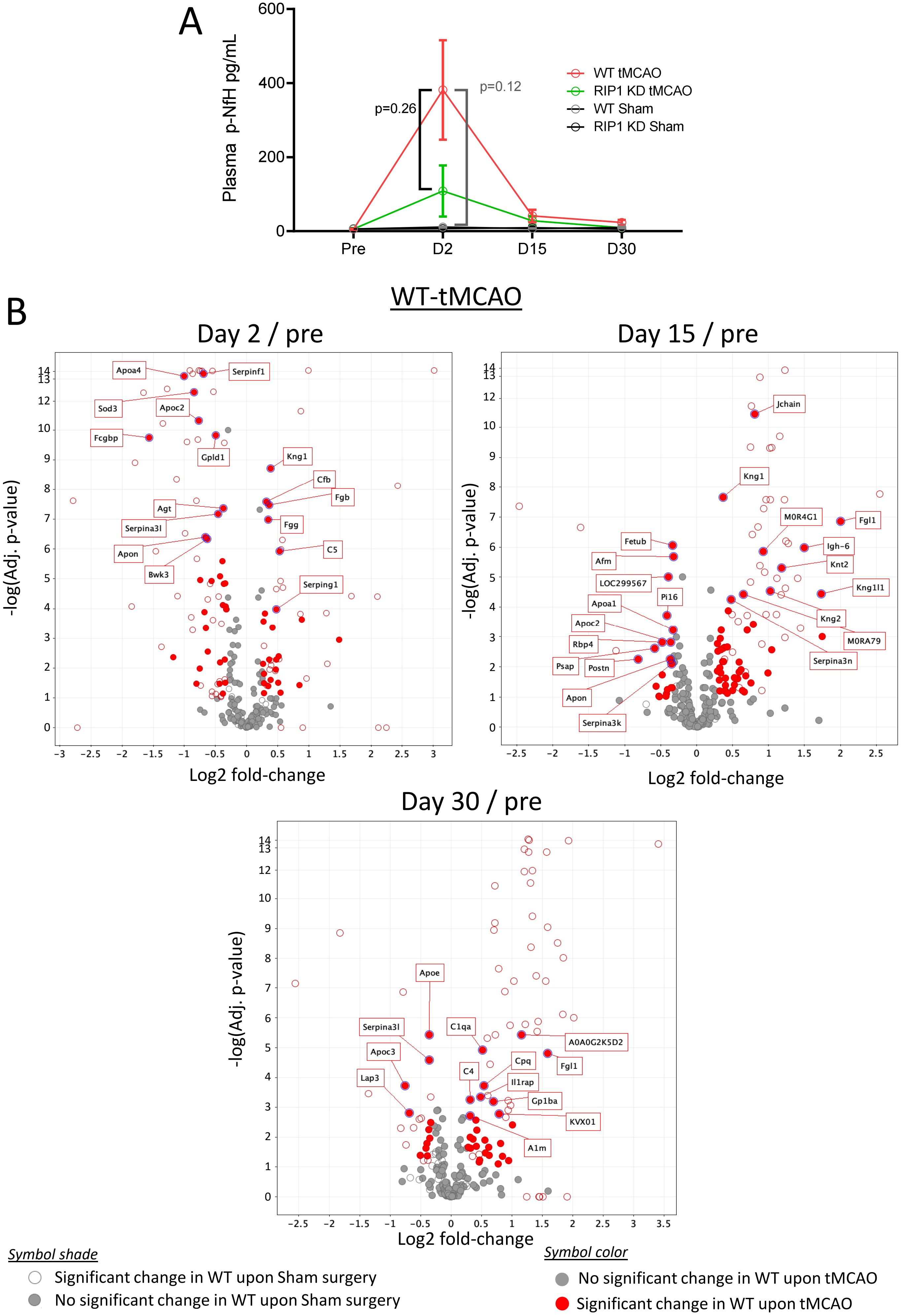

Stark and Goncharov et al., 2021 Supplementary Figure 3
