# Supplementary material for: Genetic inactivation of RIP1 kinase activity in rats protects against ischemic brain injury
Source: Cell Death Dis. 2021 Apr 7;12(4):379. doi: 10.1038/s41419-021-03651-6 (PMC8026634; doi:10.1038/s41419-021-03651-6)
Supplement: Supplementary file 3 — Supplementary Figure and Table Legends [file 41419_2021_3651_MOESM3_ESM.pdf]

Supplementary Figure Legends

Supplementary Figure 1. Generation of RIP1 kinase-dead rats and unaltered TNF signaling in RIP1 kinase-dead cells.

A) Map and design of RIP1 kinase-dead (KD) rats. Lower panel indicates the primers that were used for genotyping.

B) Hematology profiling of RIP1 KD and WT rats. Data from individual rats of each genotype (n = 3-8) are plotted, with the mean value for each group indicated by a horizontal line. There were no significant differences between two genotypes as evaluated by unpaired t-test ($p > 0.1$). Abbreviations designation: BASO ABS = Basophil Absolute Count, EOS ABS = Eosinophil Absolute Count, HCT = Hematocrit, HGB = Hemoglobin level, IRF = Immature Reticulocyte Fraction, LYMPH ABS = Lymphocyte Absolute Count, MCH = Mean Cell Hemoglobin, MCHC = Mean Cellular Hemoglobin per Cell, MCV = Mean Corpuscular Volume, MONO ABS = Monocyte Absolute Count, MPV = Mean Platelet Volume, NEUT ABS = Neutrophil Absolute Count, PCV = Packed Cell Volume, PDW = Platelet Distribution Width, PLT = Platelet Count, RBC = Erythrocyte Count, RDW = Red Cell Distribution Width, RET ABS = Reticulocytes Absolute Count, WBC = Leukocyte Count.

C) BMDMs derived from WT and RIP1 KD rats (numbers in parentheses indicate that cells were derived from different animals) treated with TNF (20 ng/ml) for indicated periods. Cellular lysates were examined by immunoblotting with the indicated antibodies.

D) BMDMs derived from WT and RIP1 KD rats (numbers in parentheses indicate that cells were derived from different animals) treated with TNF (20 ng/ml) and TAK1 inhibitor 5z-7-oxozeanol (2.5 μM) with or without Nec1 (20 μM) overnight. Cell viability was assessed by Cell Titer-Glo assay. Data are represented as mean +/- S.E.M.

Supplementary Figure 2. Quantification of cerebrovascular morphology parameters between WT and RIP1 KD rats.

A) Representative micro-CT images of cerebrovascular structure in WT and RIP1 KD rats.

B) Cerebrovascular morphology parameters measured by micro-CT imaging (~2-months old male rats. n = 5 and 3 rats for WT and RIP1 KD, respectively). No significant differences were found between WT and RIP1 KD rats. CV: Coefficient of variation.

C) Representative DWI images of WT RIP1 KD rat brains during MCAO occlusion.

D) Quantification of lesion size, from (C). Lesion size over four planes as shown were integrated for each animal. $p = 0.17$ by Mann-Whitney test.

Supplementary Figure 3. Plasma protein levels in WT and RIP1 KD rats following tMCAO

A) Plasma p-NfH levels in WT and RIP1 KD rats before and 2-, 15- and 30-days after sham or tMCAO surgery. n = 8-10 rats/group. p-values were calculated by Two-Way ANOVA (Holm-Sidak).

B) Plasma protein profiling by mass spectrometry in WT rats before and 2-, 15- and 30-days after sham or tMCAO surgery. Each graph represents log2 fold-change (x-axes) and adjusted p-value (y-axes) in plasma protein abundance at the indicated time points post-surgery compared to pre-surgery. Proteins that show significant difference in abundance upon sham surgery in WT animals (>1.2 fold-change, adjusted $p < 0.1$) are highlighted with open symbols. Symbol colors represent changes in WT animals post-tMCAO with gray and red symbols referring to "unchanged" and "significantly changed" proteins, respectively.

Supplementary Table Legends

Supplementary Table 1. Plasma protein profiling in WT and RIP1 KD rats

Protein name, Gene ID, protein description for the 275 plasma proteins quantified by mass spectrometry in WT and RIP1 KD rat plasma at day 2, 15 and 30-days post-sham or -

tMCAO surgery. log2 fold-change (log2FC), unadjusted and adjusted p-values are tabulated for each comparison between the genotypes and treatments.

Supplementary Table 2. Pathway analysis of differentially regulated proteins in WT and RIP1 KD rat plasma following tMCAO

Pathway analysis of upregulated and downregulated proteins in WT rat plasma following tMCAO (i.e. red closed symbols in Supplementary Fig. 3B), and proteins with significant change in WT, but not in RIP1 KD rat plasma, following tMCAO (i.e. red closed symbols in Fig. 5C). The Reactome Database was used to identify the enriched pathways and the associated statistics.

Supplementary Table 3. Quantification of 275 plasma proteins in WT and RIP1 KD rats following tMCAO

Log2 fold change in plasma proteins in WT (red) and RIP1 KD (green) rats over time. Open symbols represent proteins without significant change (adjusted $p > 0.1$) and closed symbols represent proteins with significant change (adjusted $p < 0.1$), as compared to their baseline within their respective genotypes. Each page highlights one of the 275 proteins quantified. The error bars show the standard error of the log2 fold change.
