# Supplementary material for: Genetic inactivation of RIP1 kinase activity in rats protects against ischemic brain injury
Source: Cell Death Dis. 2021 Apr 7;12(4):379. doi: 10.1038/s41419-021-03651-6 (PMC8026634; doi:10.1038/s41419-021-03651-6)
Supplement: Supplementary file 6 — Supplementary Table 3 [file 41419_2021_3651_MOESM6_ESM.pdf]

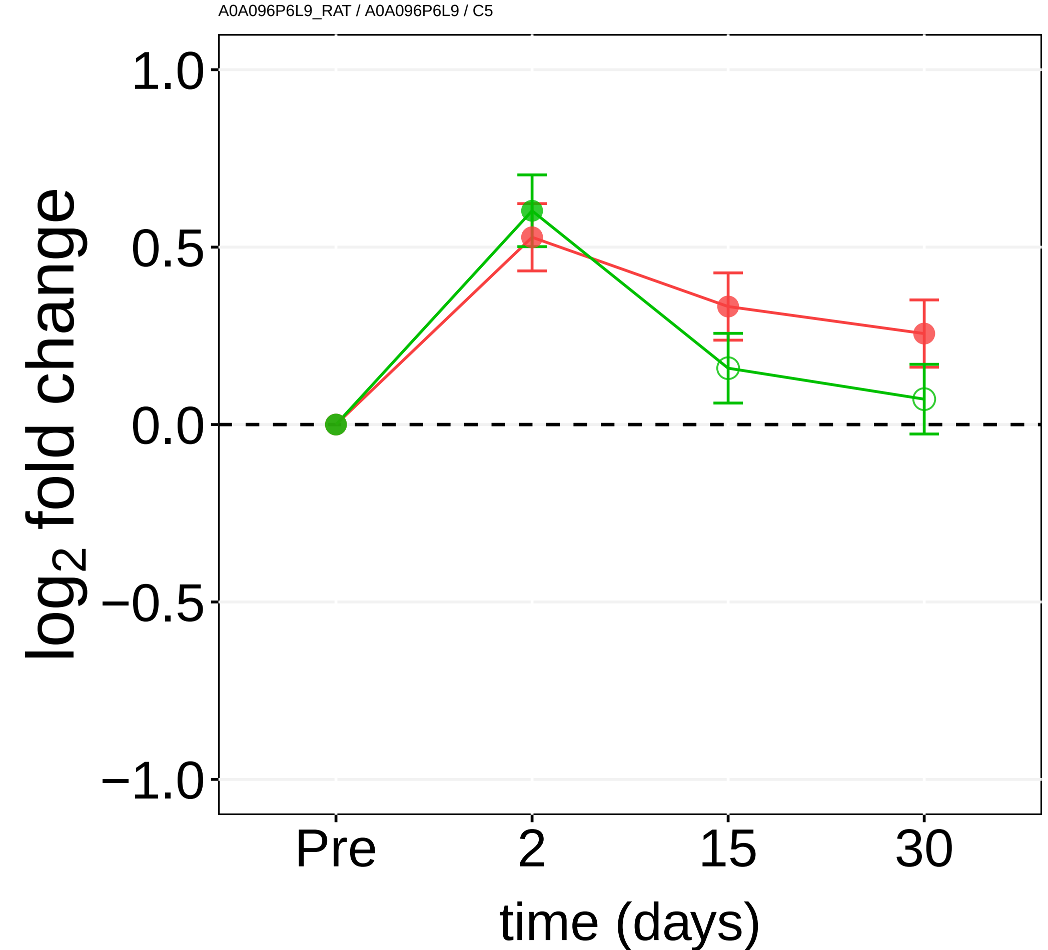
A0A096P6L9_RAT / A0A096P6L9 / C5
1.0
0.5
0.0
−0.5
−1.0
log2 fold change
Pre
2
15
30
time (days)

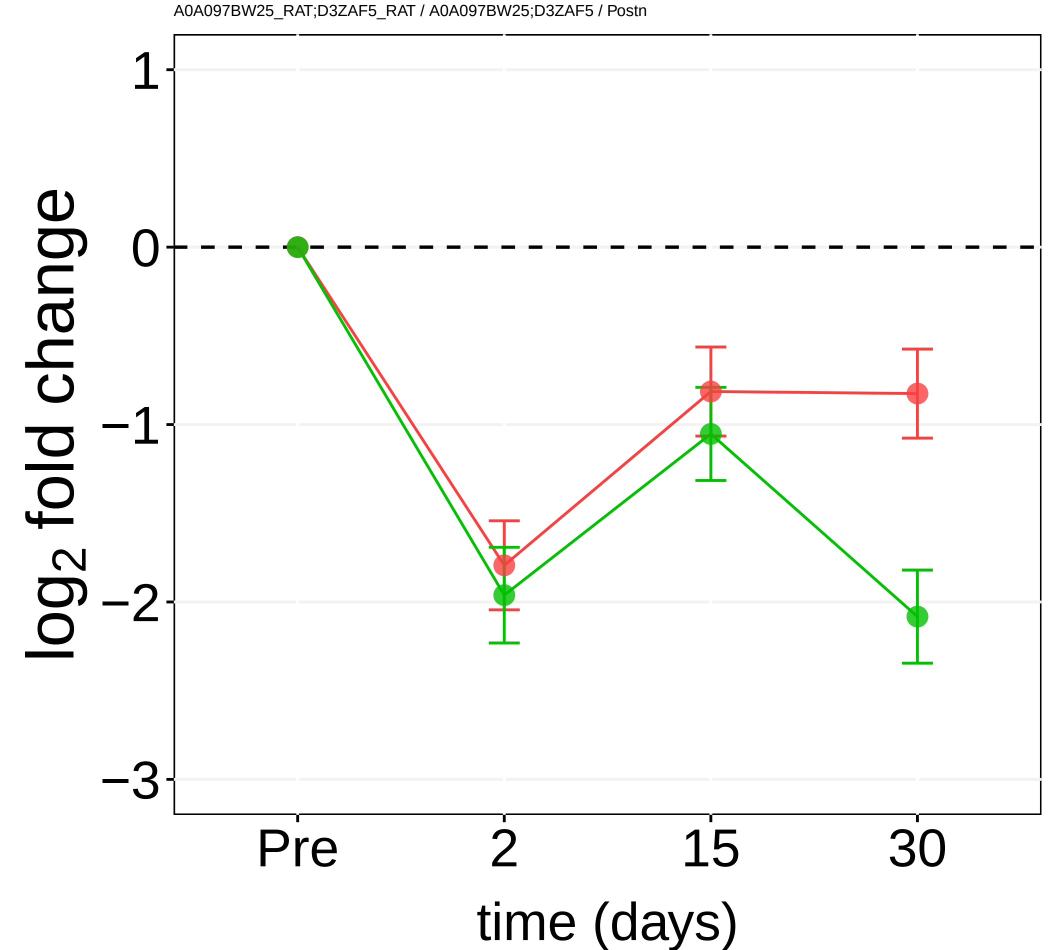
A0A097BW25_RAT;D3ZAF5_RAT / A0A097BW25;D3ZAF5 / Postn
1
0
−1
−2
−3
log2 fold change
Pre
2
15
30
time (days)

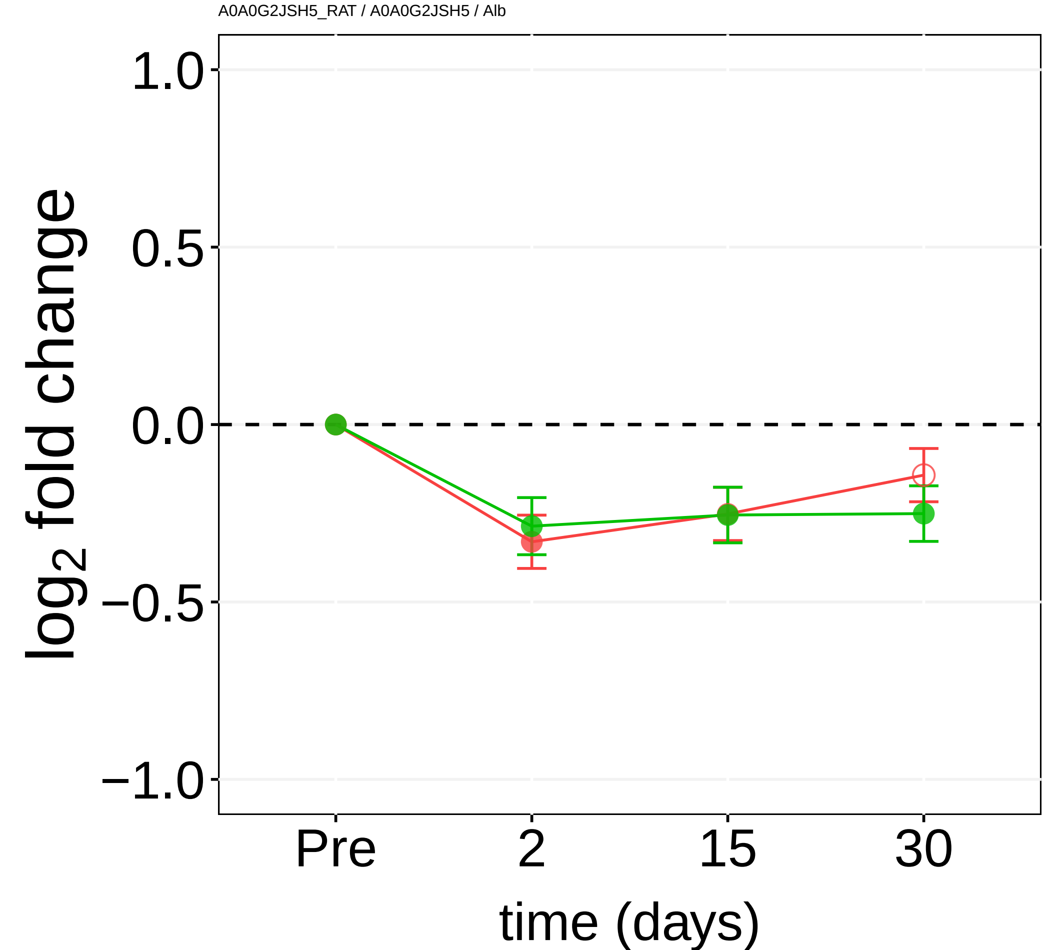
A0A0G2JSH5_RAT / A0A0G2JSH5 / Alb
1.0
0.5
0.0
−0.5
−1.0
log2 fold change
Pre
2
15
30
time (days)

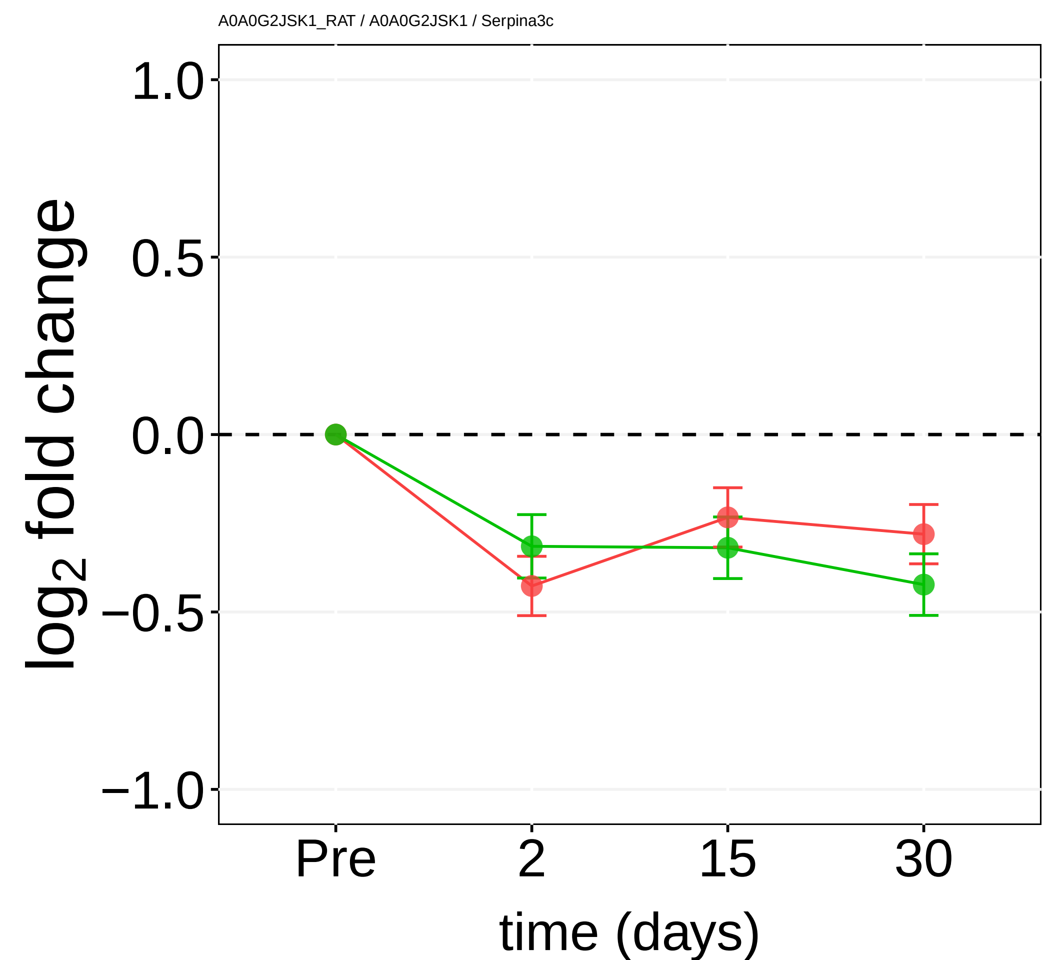

A0A0G2JSK1_RAT / A0A0G2JSK1 / Serpina3c
1.0
0.5
0.0
−0.5
−1.0
log2 fold change
Pre
2
15
30
time (days)

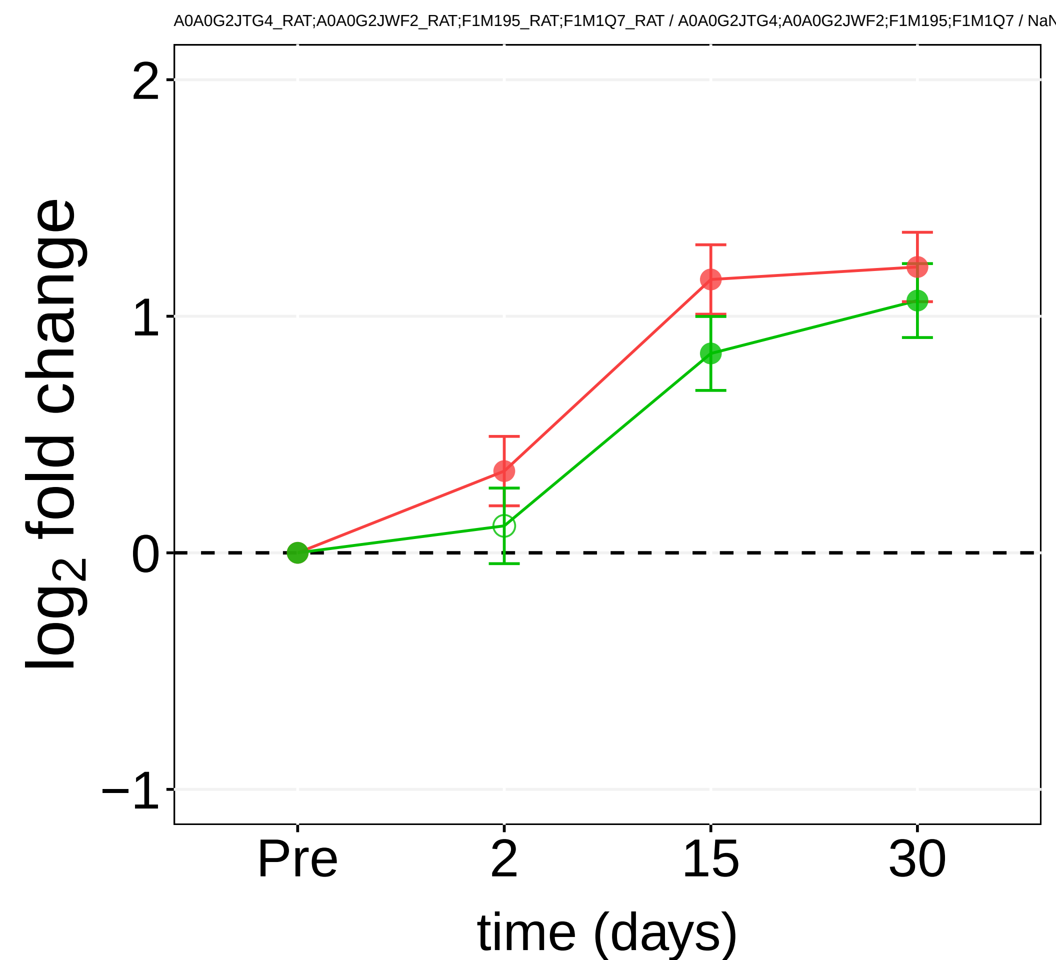
A0A0G2JTG4_RAT;A0A0G2JWF2_RAT;F1M195_RAT;F1M1Q7_RAT / A0A0G2JTG4;A0A0G2JWF2;F1M195;F1M1Q7 / NaN
log2 fold change
2
1
0
−1
Pre
2
15
30
time (days)

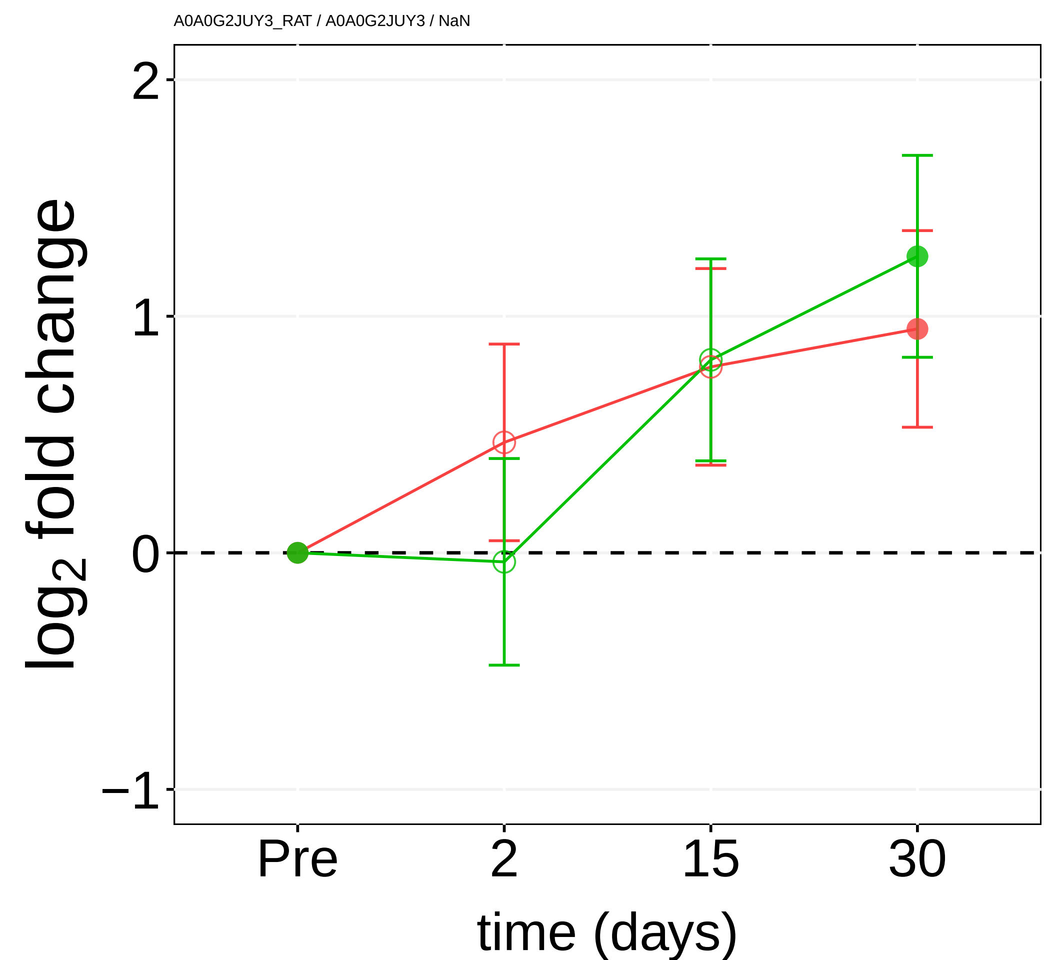

A0A0G2JUY3_RAT / A0A0G2JUY3 / NaN
2
1
0
−1
log2 fold change
Pre
2
15
30
time (days)

A0A0G2JUY4_RAT / A0A0G2JUY4 / NaN

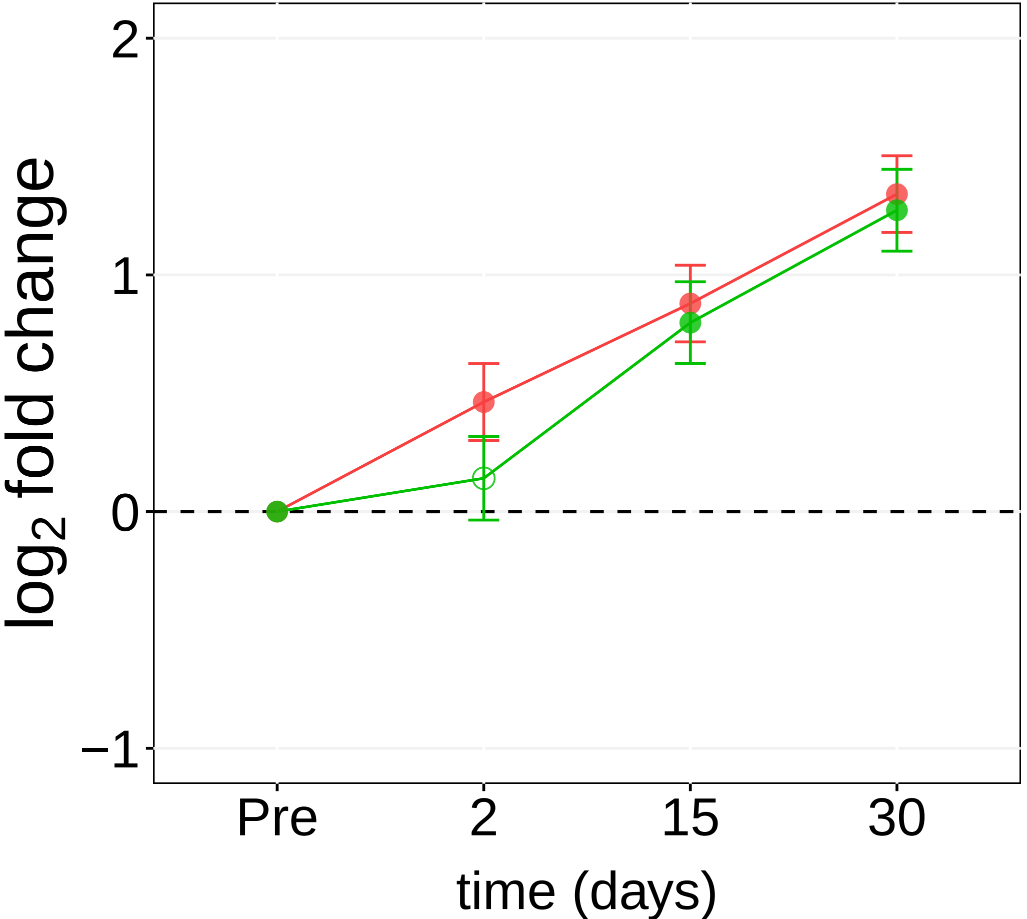

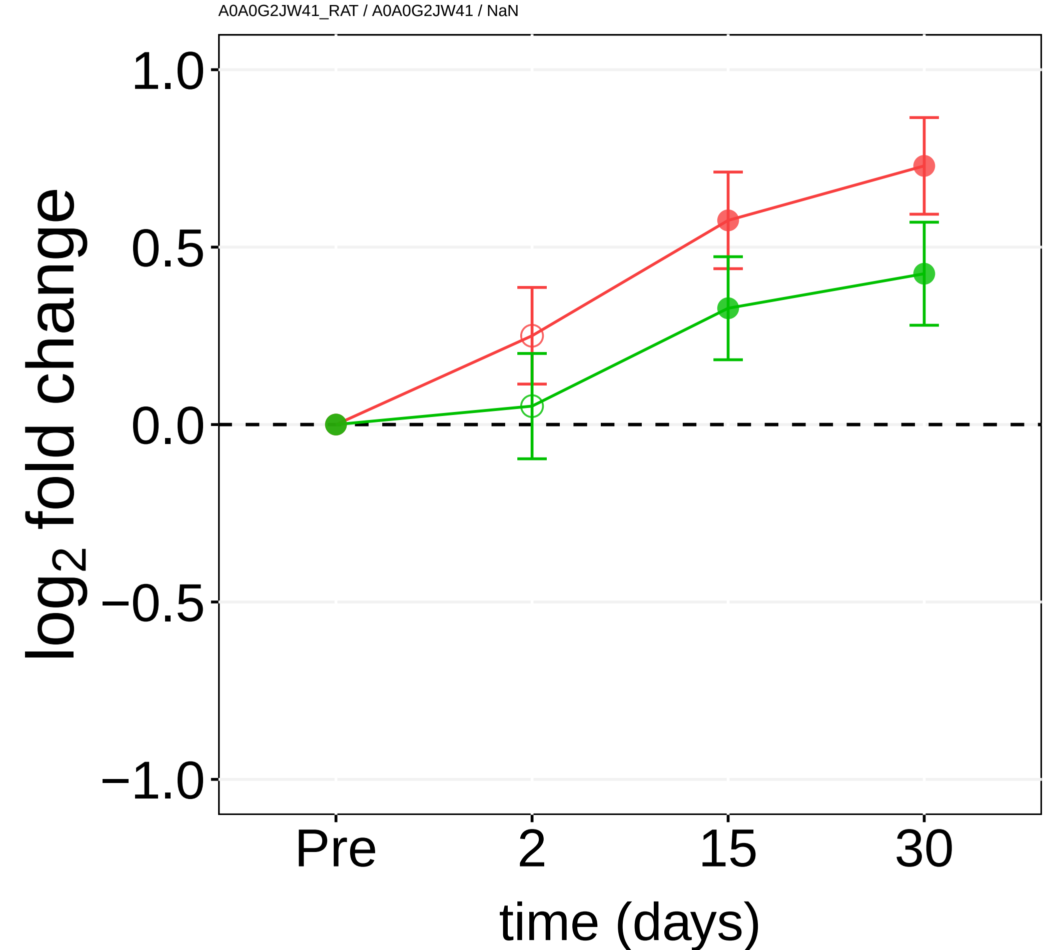

A0A0G2JW41_RAT / A0A0G2JW41 / NaN
1.0
0.5
0.0
−0.5
−1.0
log2 fold change
Pre
2
15
30
time (days)

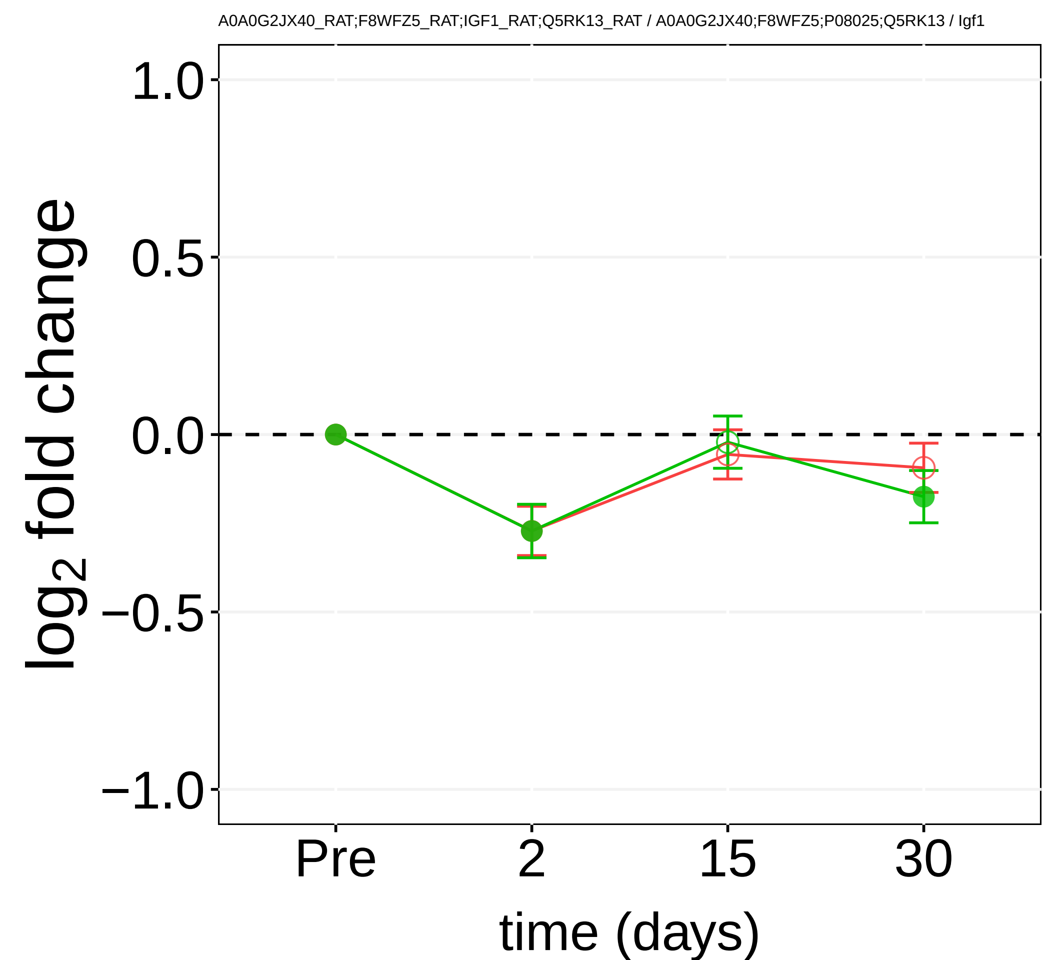

A0A0G2JX40_RAT;F8WFZ5_RAT;IGF1_RAT;Q5RK13_RAT / A0A0G2JX40;F8WFZ5;P08025;Q5RK13 / Igf1
log2 fold change
1.0
0.5
0.0
−0.5
−1.0
Pre
2
15
30
time (days)

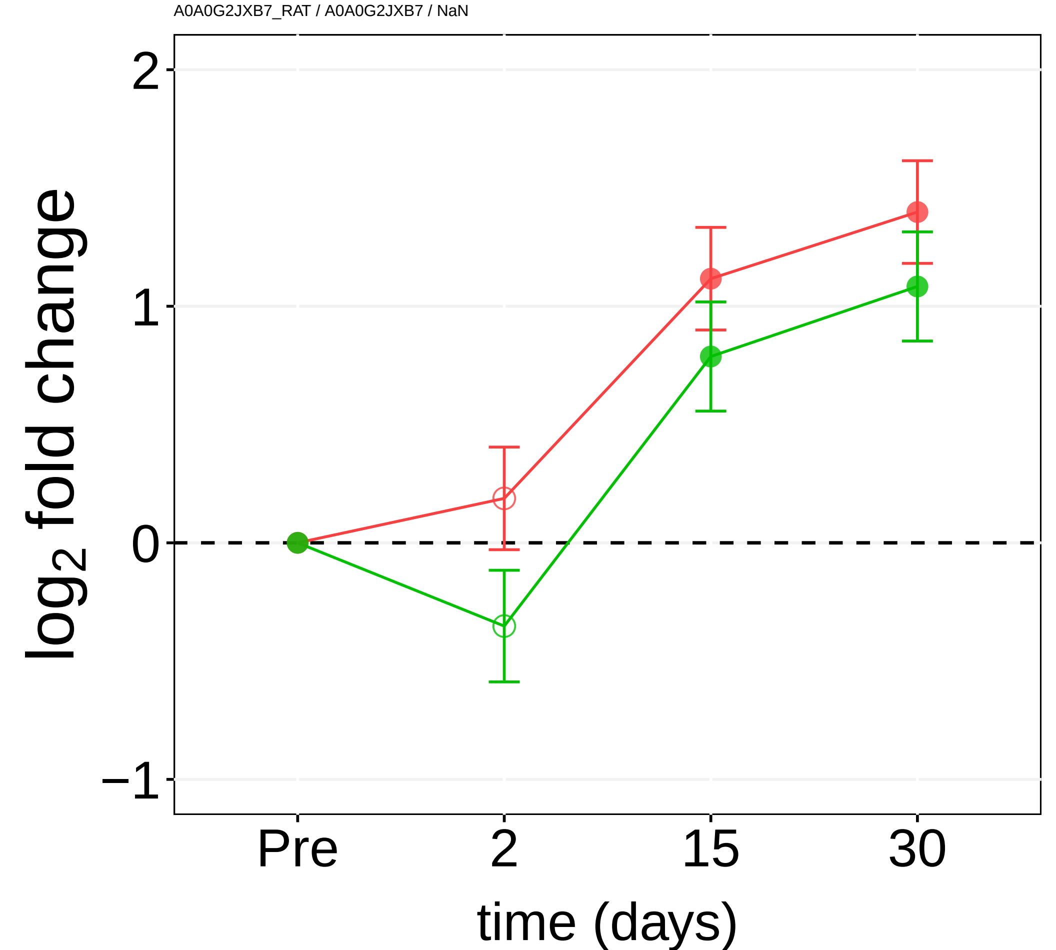
A0A0G2JXB7_RAT / A0A0G2JXB7 / NaN
2
1
0
−1
$\log_2$ fold change
Pre
2
15
30
time (days)

A0A0G2JXP0_RAT;M0RCK5_RAT;M0RDF0_RAT / A0A0G2JXP0;M0RCK5;M0RDF0 / NaN

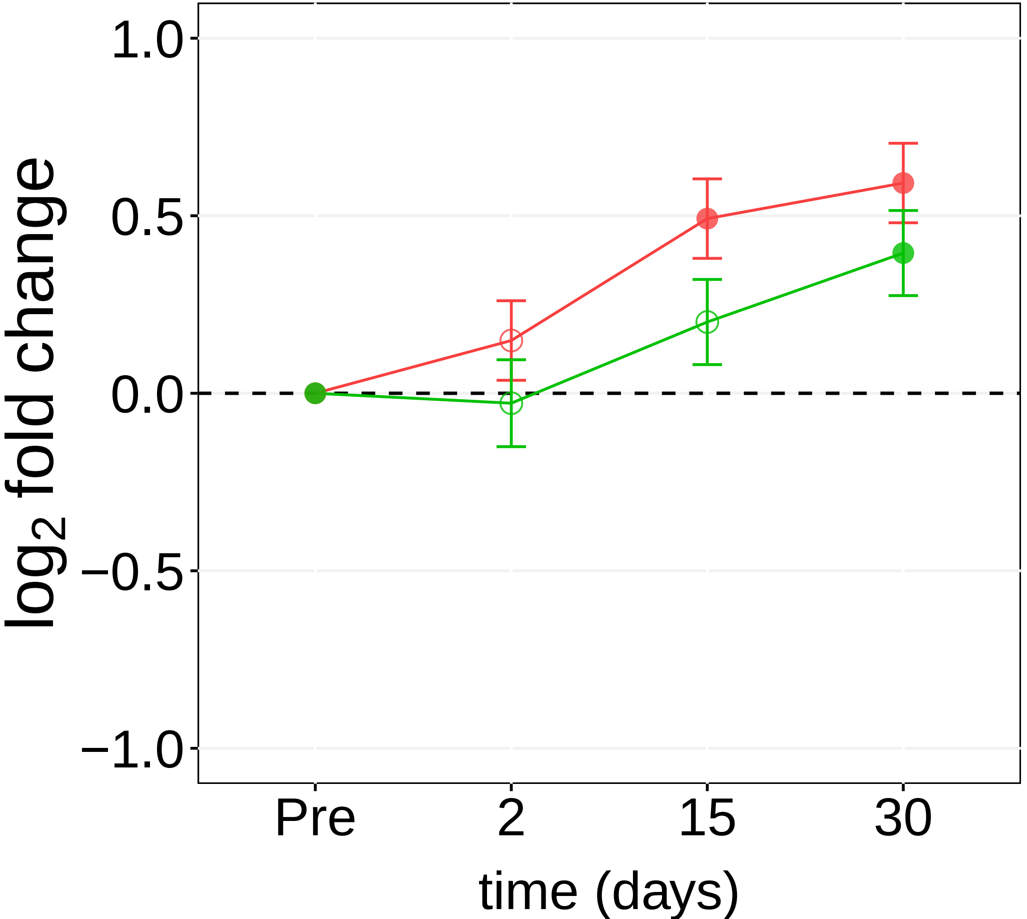

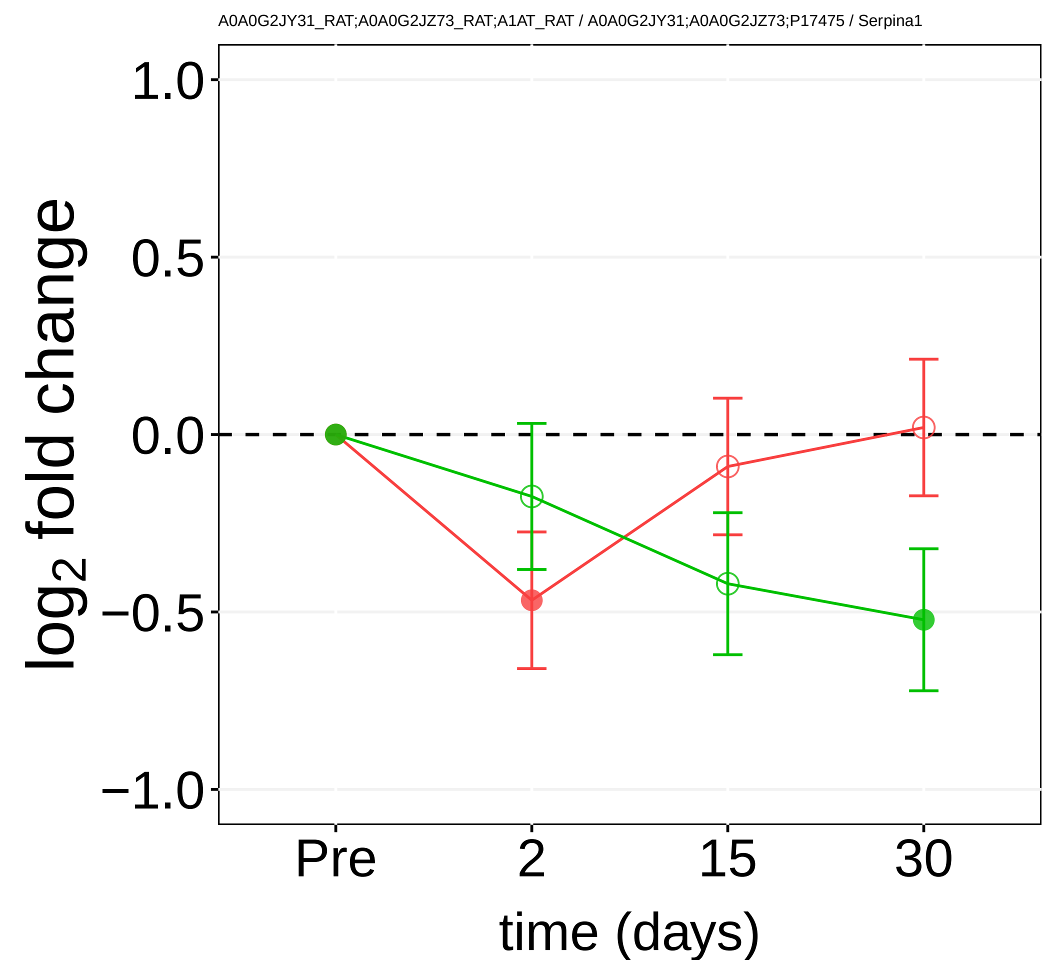
A0A0G2JY31_RAT;A0A0G2JZ73_RAT;A1AT_RAT / A0A0G2JY31;A0A0G2JZ73;P17475 / Serpina1
1.0
0.5
0.0
−0.5
−1.0
log2 fold change
Pre
2
15
30
time (days)

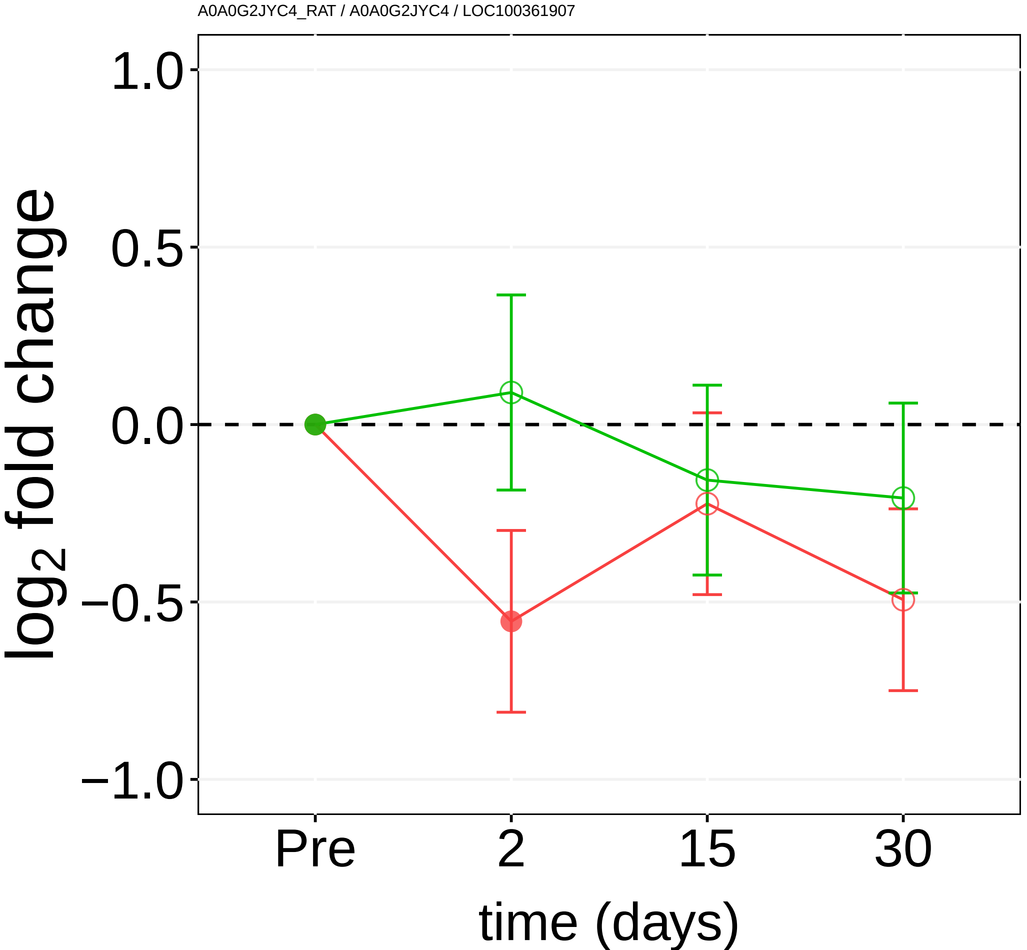
A0A0G2JYC4_RAT / A0A0G2JYC4 / LOC100361907
1.0
0.5
0.0
−0.5
−1.0
log2 fold change
Pre
2
15
30
time (days)

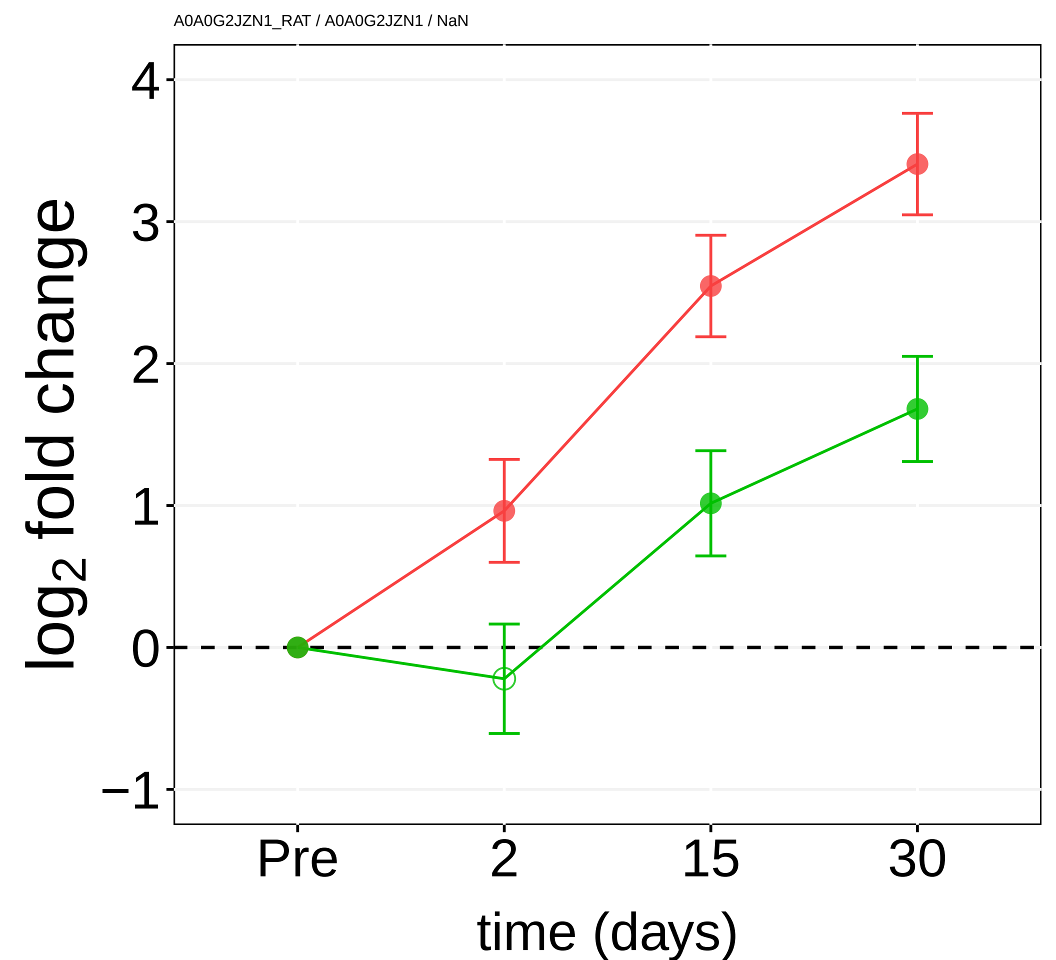
A0A0G2JZN1_RAT / A0A0G2JZN1 / NaN
log2 fold change
4
3
2
1
0
−1
Pre
2
15
30
time (days)

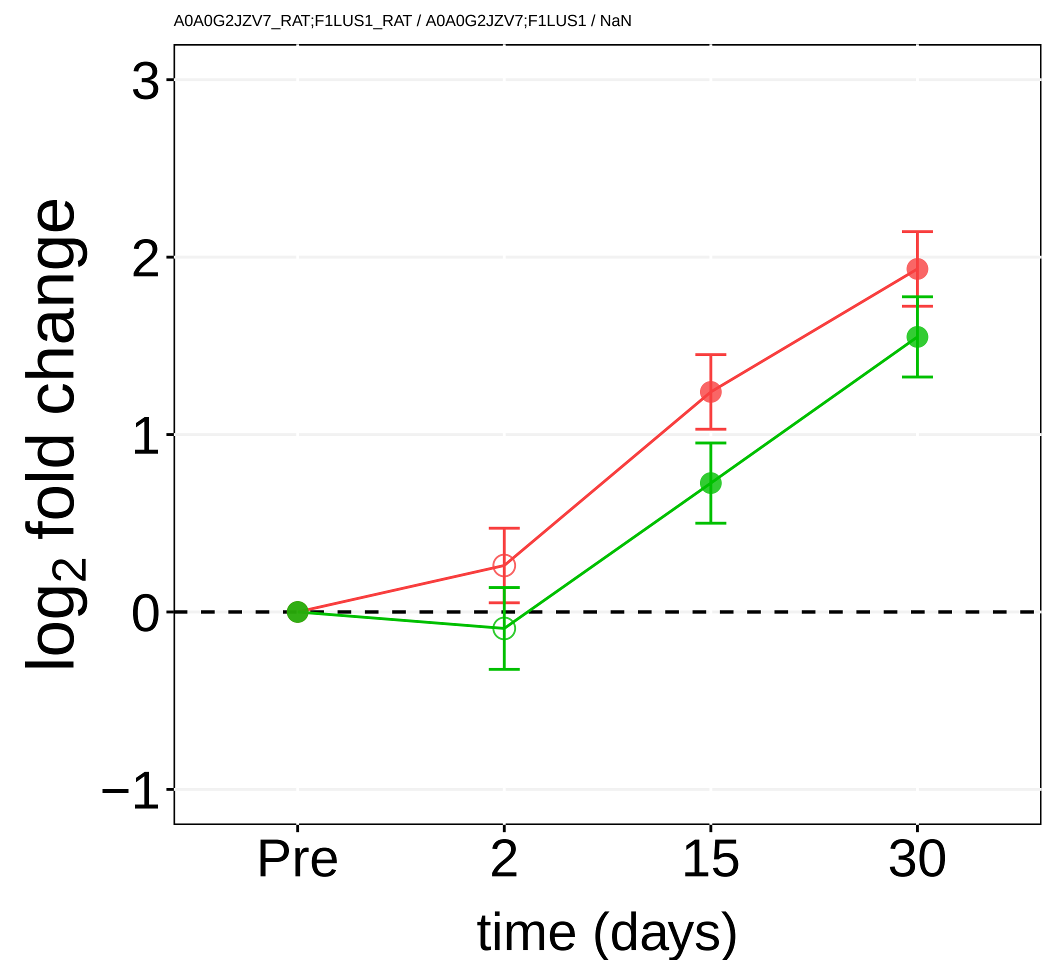

A0A0G2JZV7_RAT;F1LUS1_RAT / A0A0G2JZV7;F1LUS1 / NaN
log2 fold change
3
2
1
0
−1
Pre
2
15
30
time (days)

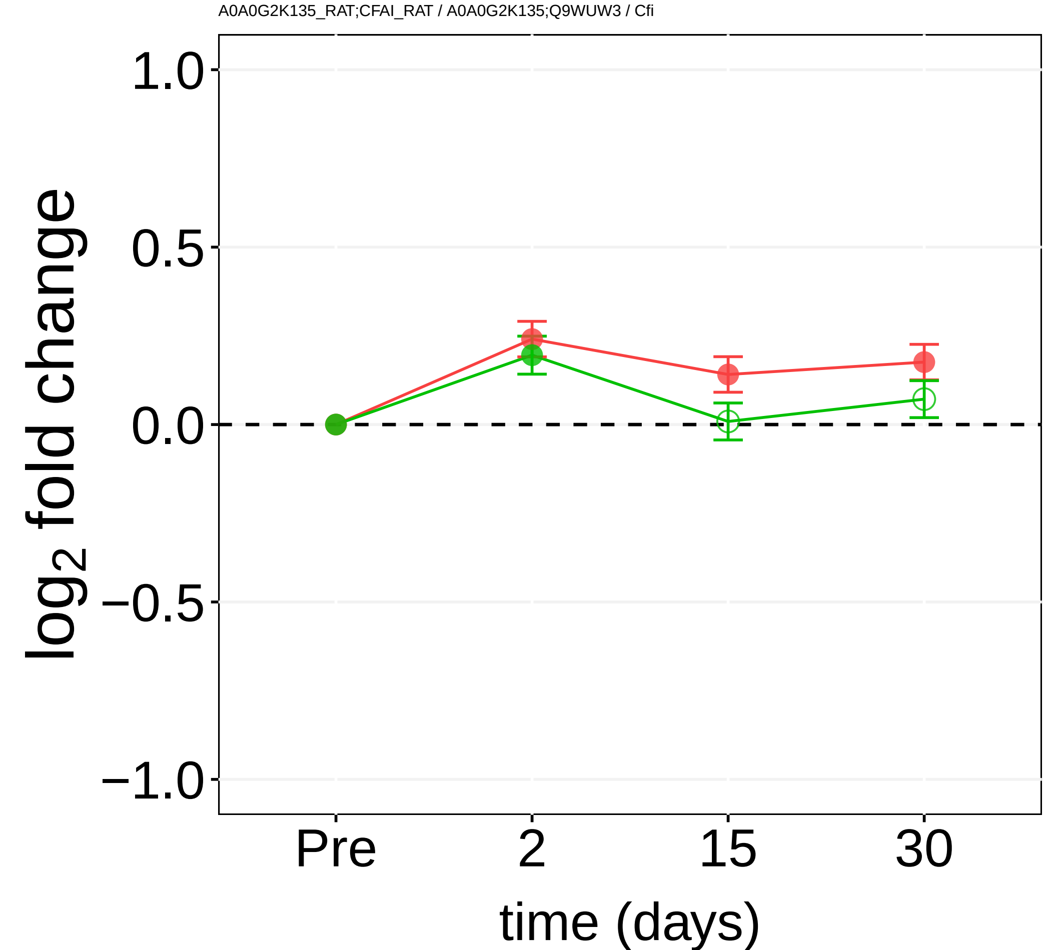
A0A0G2K135_RAT;CFAI_RAT / A0A0G2K135;Q9WUW3 / Cfi
log2 fold change
1.0
0.5
0.0
−0.5
−1.0
Pre
2
15
30
time (days)

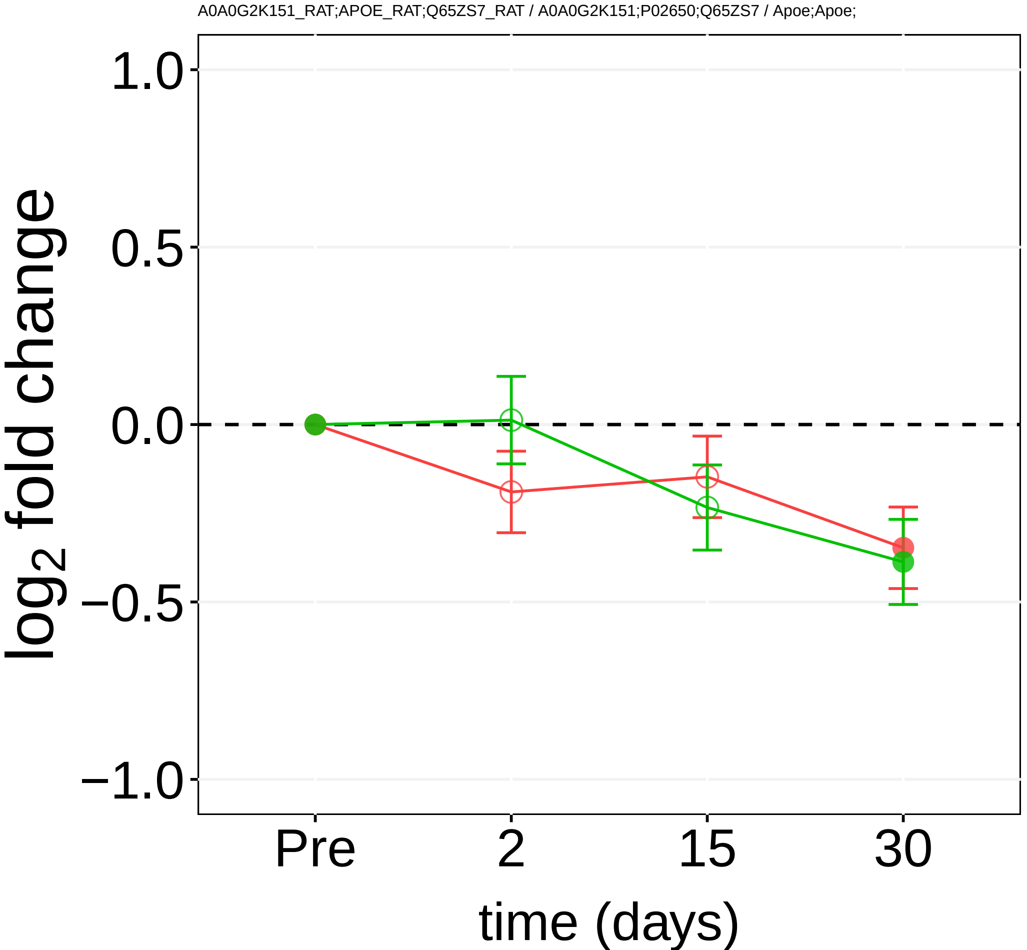

A0A0G2K151_RAT;APOE_RAT;Q65ZS7_RAT / A0A0G2K151;P02650;Q65ZS7 / Apoe;Apoe;
1.0
0.5
0.0
−0.5
−1.0
log2 fold change
Pre
2
15
30
time (days)

A0A0G2K245_RAT;A0A0G2KAL1_RAT / A0A0G2K245;A0A0G2KAL1 / NaN

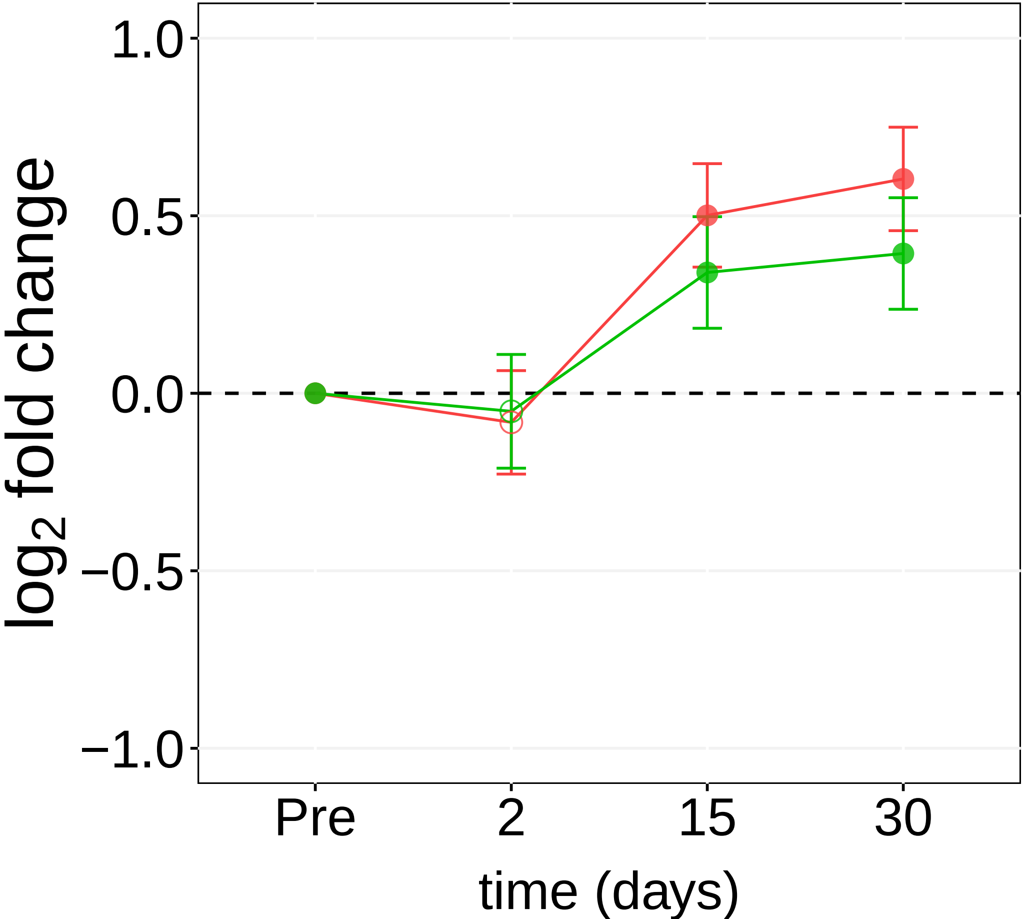

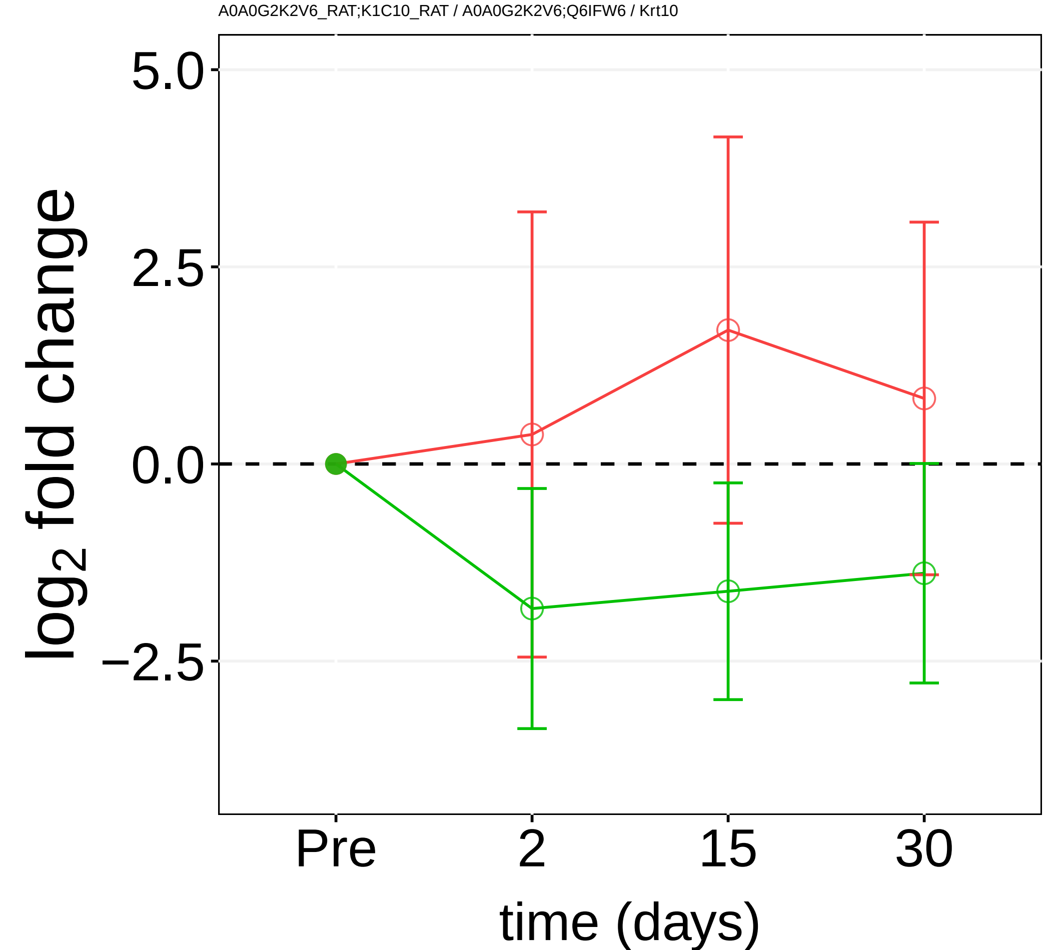
A0A0G2K2V6_RAT;K1C10_RAT / A0A0G2K2V6;Q6IFW6 / Krt10
5.0
2.5
0.0
−2.5
log2 fold change
Pre
2
15
30
time (days)

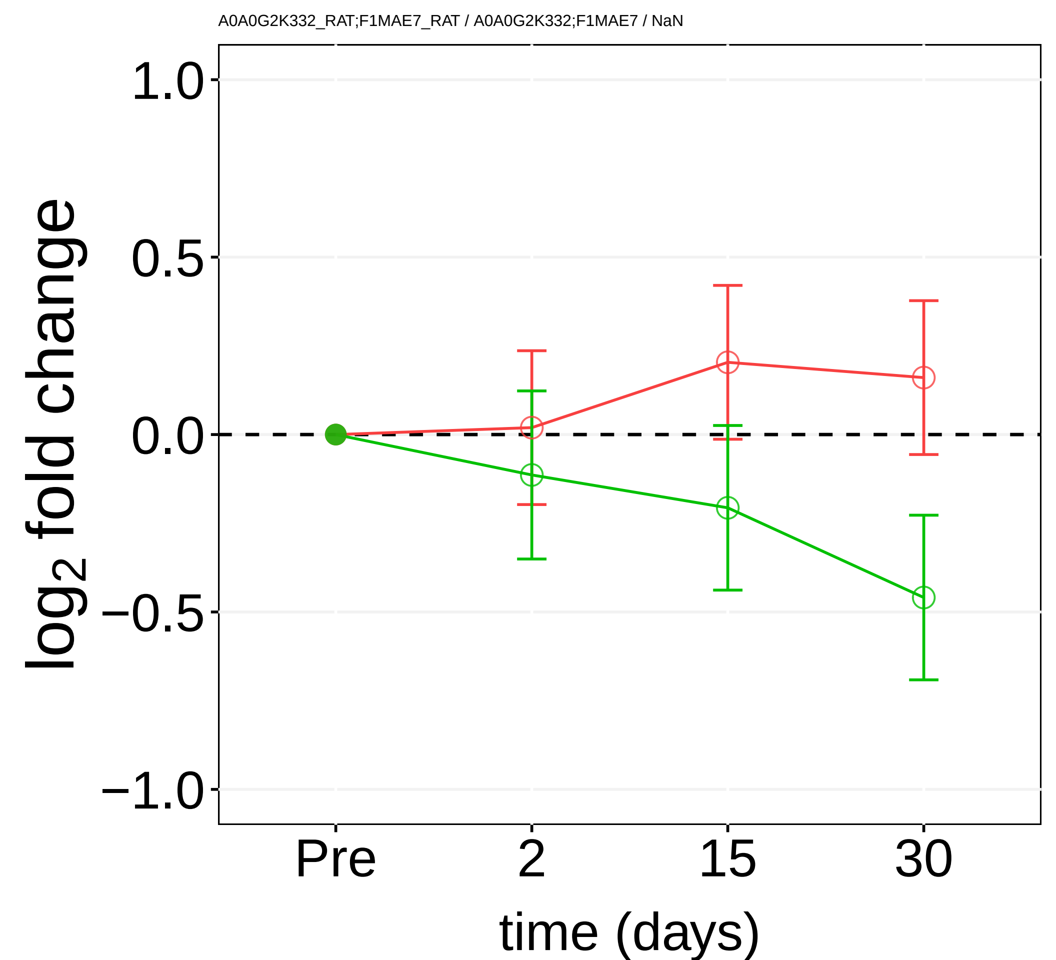
A0A0G2K332_RAT;F1MAE7_RAT / A0A0G2K332;F1MAE7 / NaN
1.0
0.5
0.0
−0.5
−1.0
log2 fold change
Pre
2
15
30
time (days)

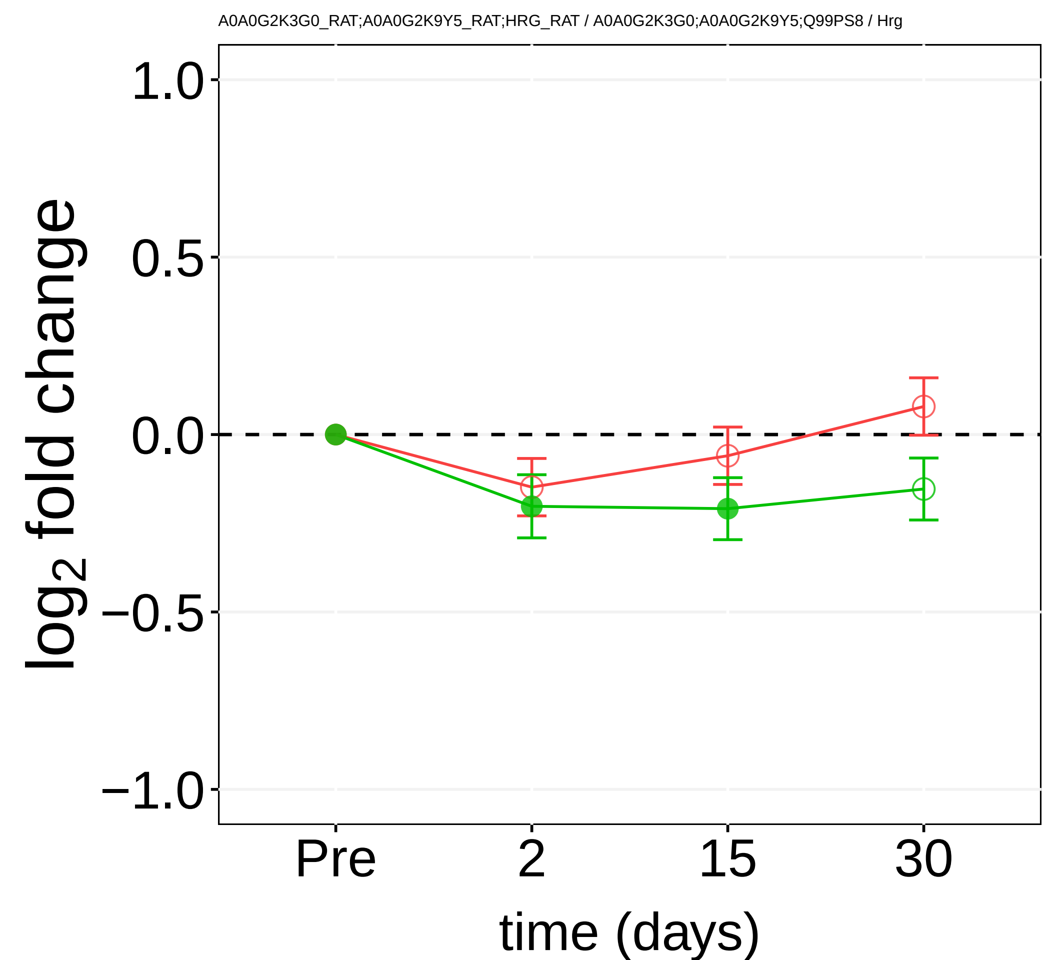
A0A0G2K3G0_RAT;A0A0G2K9Y5_RAT;HRG_RAT / A0A0G2K3G0;A0A0G2K9Y5;Q99PS8 / Hrg
log2 fold change
1.0
0.5
0.0
−0.5
−1.0
Pre
2
15
30
time (days)

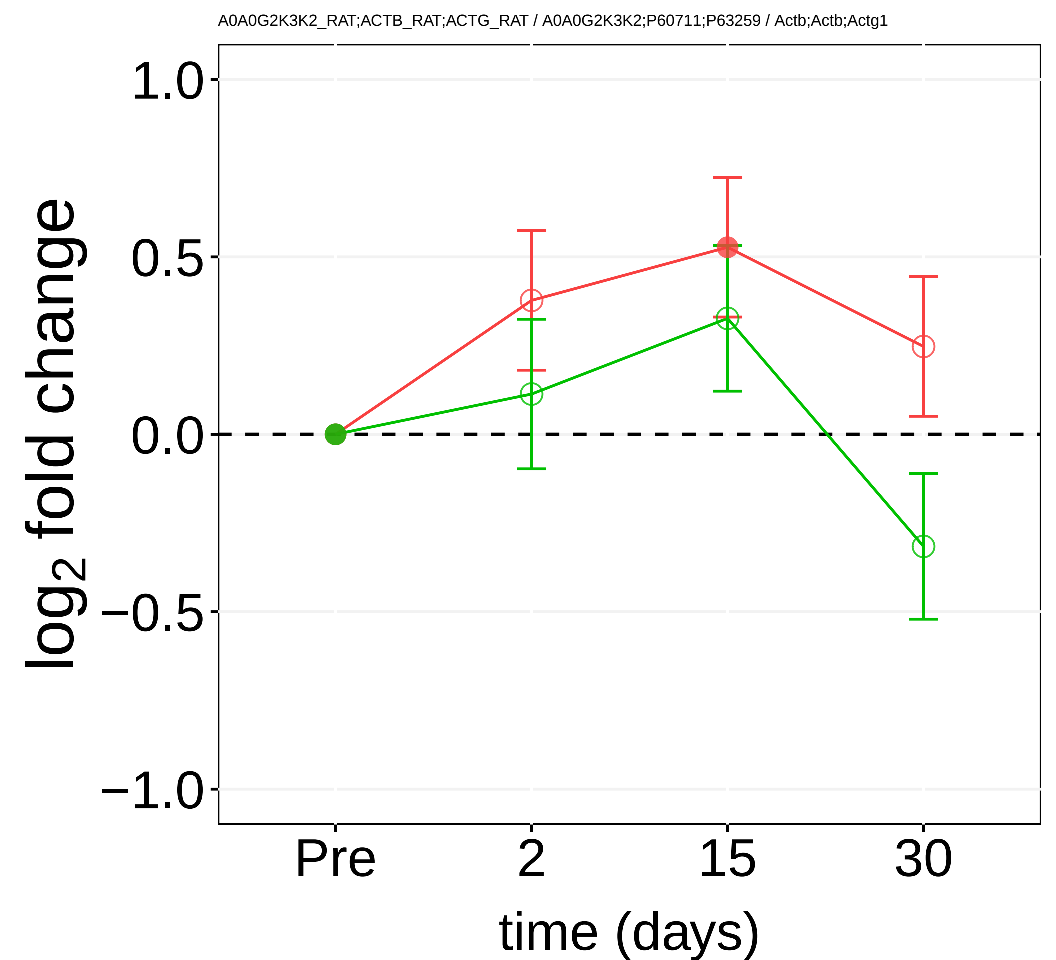

A0A0G2K3K2_RAT;ACTB_RAT;ACTG_RAT / A0A0G2K3K2;P60711;P63259 / Actb;Actb;Actg1
1.0
0.5
0.0
−0.5
−1.0
log2 fold change
Pre
2
15
30
time (days)

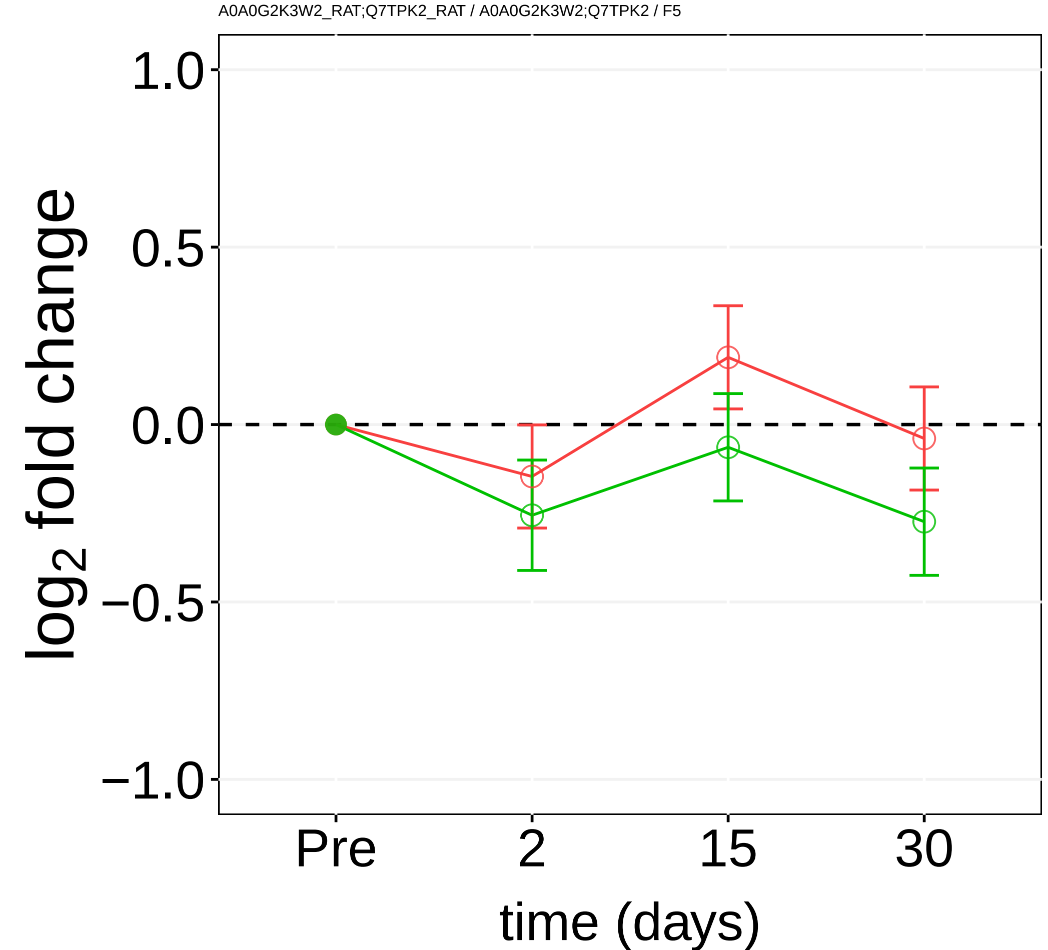
A0A0G2K3W2_RAT;Q7TPK2_RAT / A0A0G2K3W2;Q7TPK2 / F5
1.0
0.5
0.0
−0.5
−1.0
log2 fold change
Pre
2
15
30
time (days)

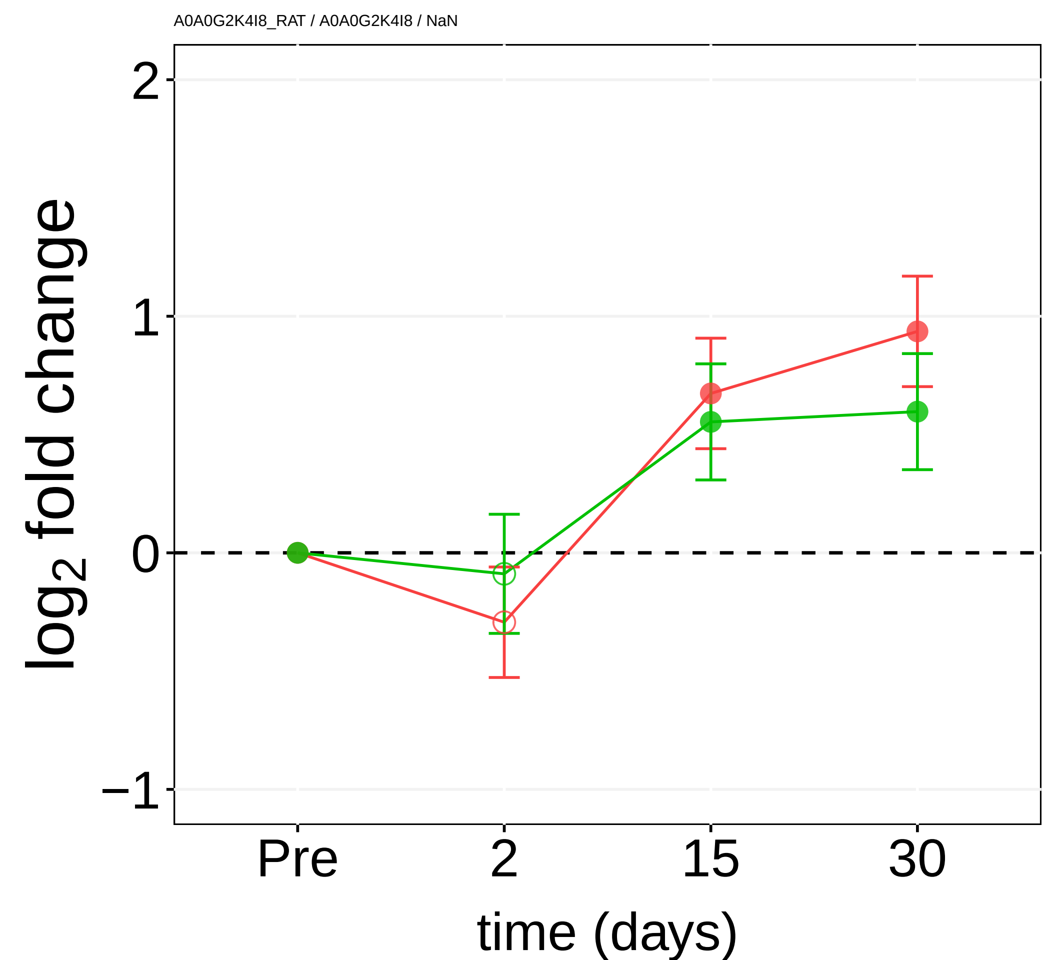
A0A0G2K4I8_RAT / A0A0G2K4I8 / NaN
log2 fold change
2
1
0
−1
Pre
2
15
30
time (days)

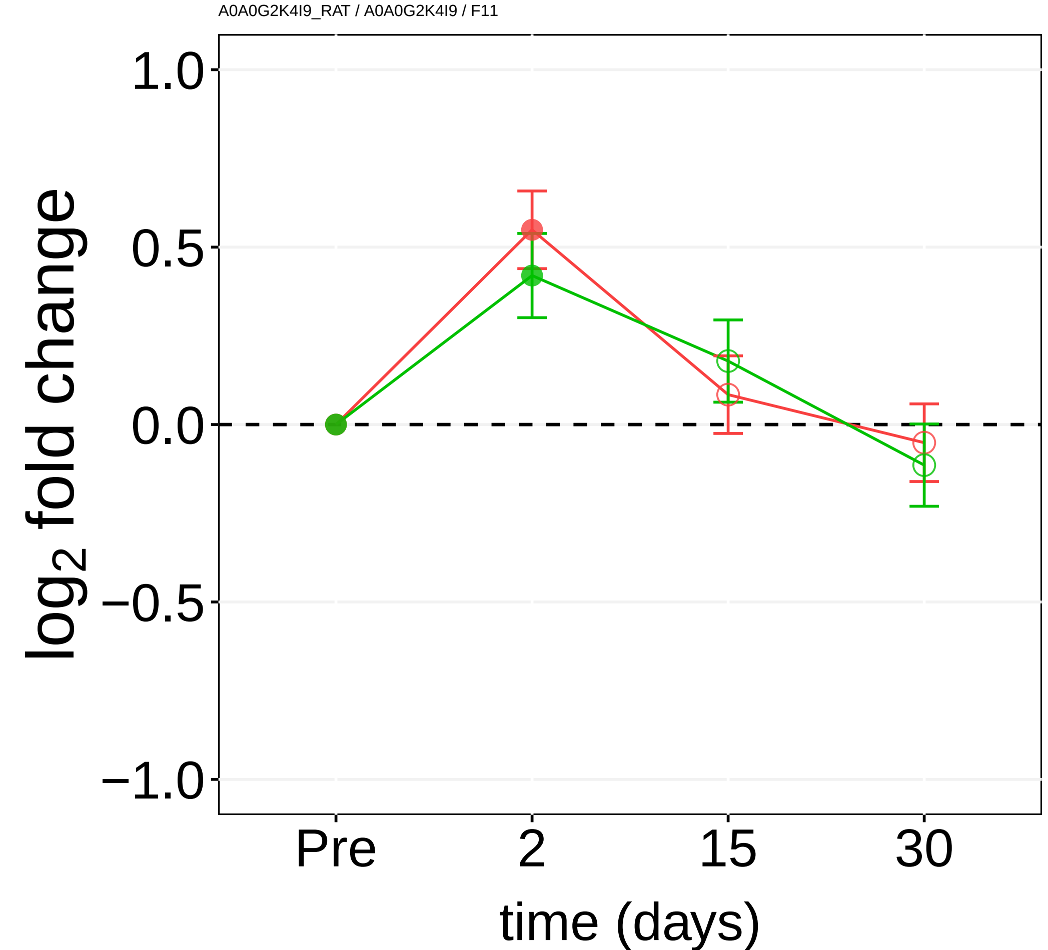
A0A0G2K4I9_RAT / A0A0G2K4I9 / F11
1.0
0.5
0.0
−0.5
−1.0
log2 fold change
Pre
2
15
30
time (days)

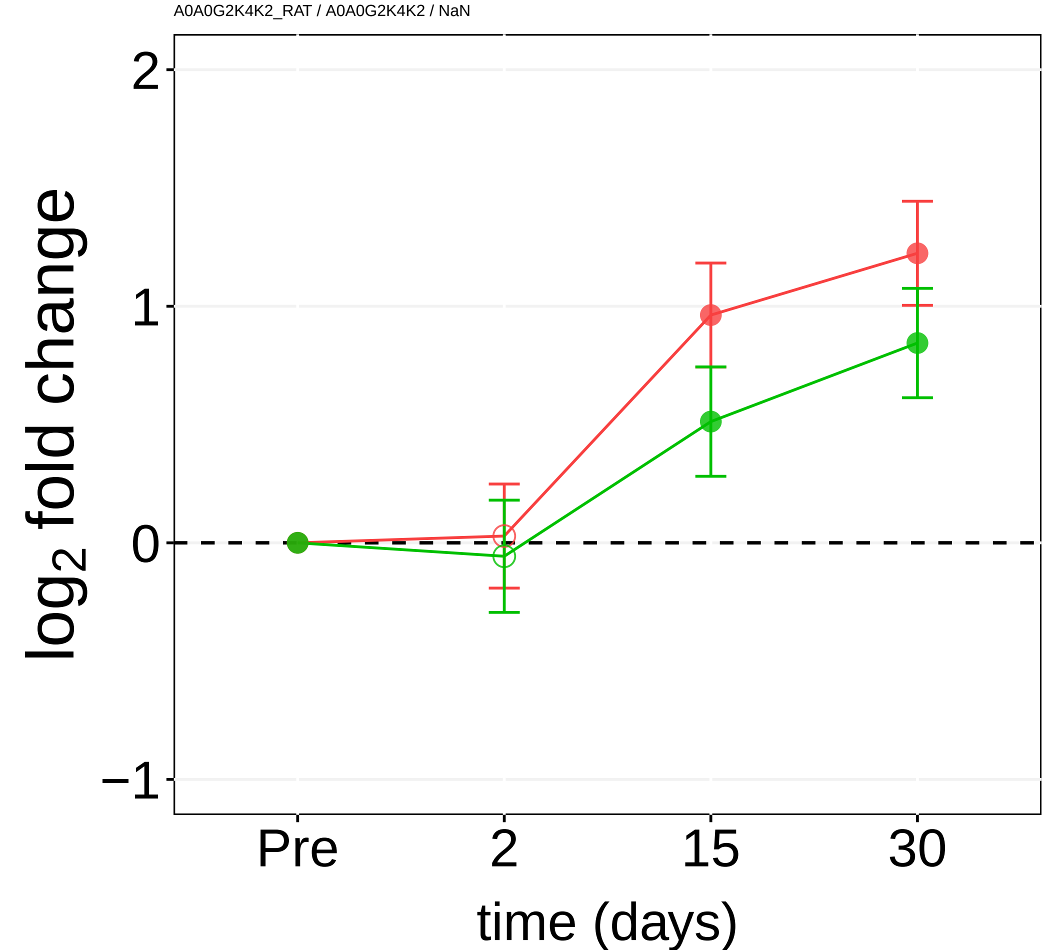

A0A0G2K4K2_RAT / A0A0G2K4K2 / NaN
log2 fold change
2
1
0
−1
Pre
2
15
30
time (days)

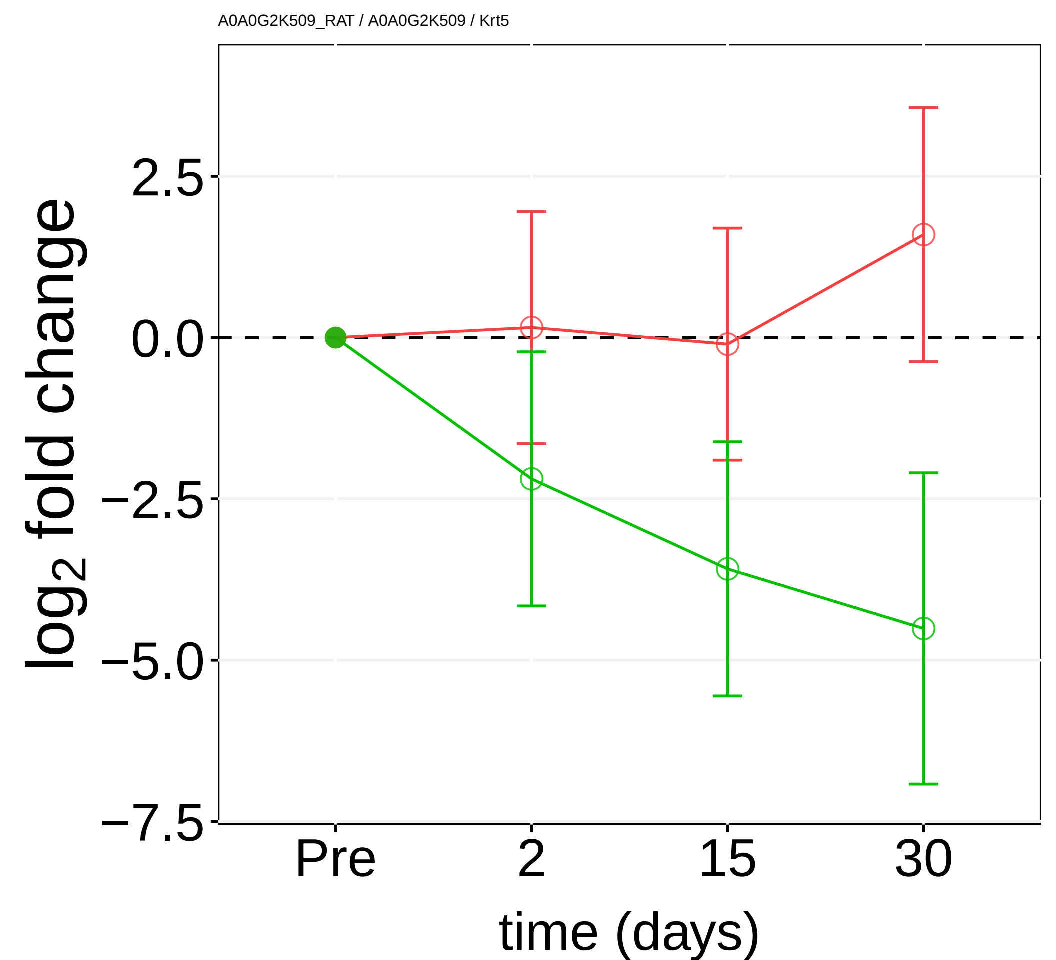
A0A0G2K509_RAT / A0A0G2K509 / Krt5
log2 fold change
2.5
0.0
−2.5
−5.0
−7.5
Pre
2
15
30
time (days)

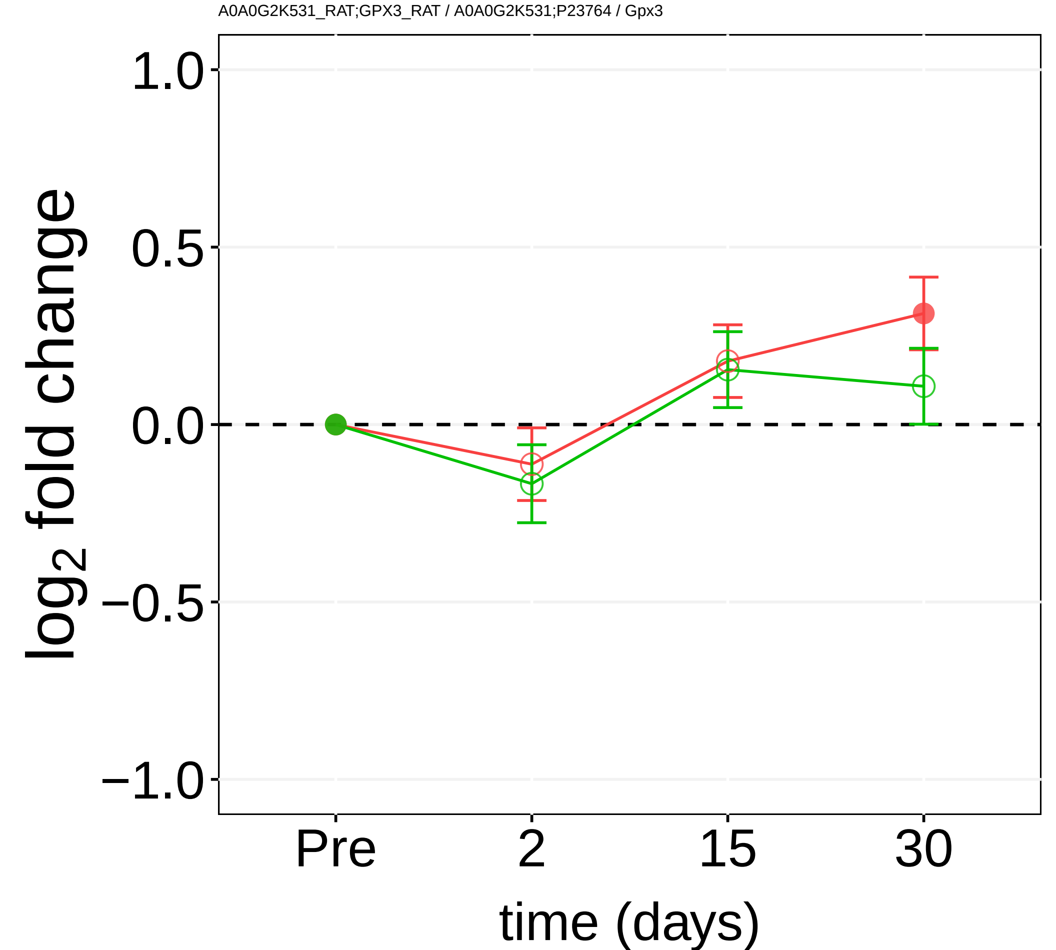

A0A0G2K531_RAT;GPX3_RAT / A0A0G2K531;P23764 / Gpx3
log2 fold change
1.0
0.5
0.0
−0.5
−1.0
Pre
2
15
30
time (days)

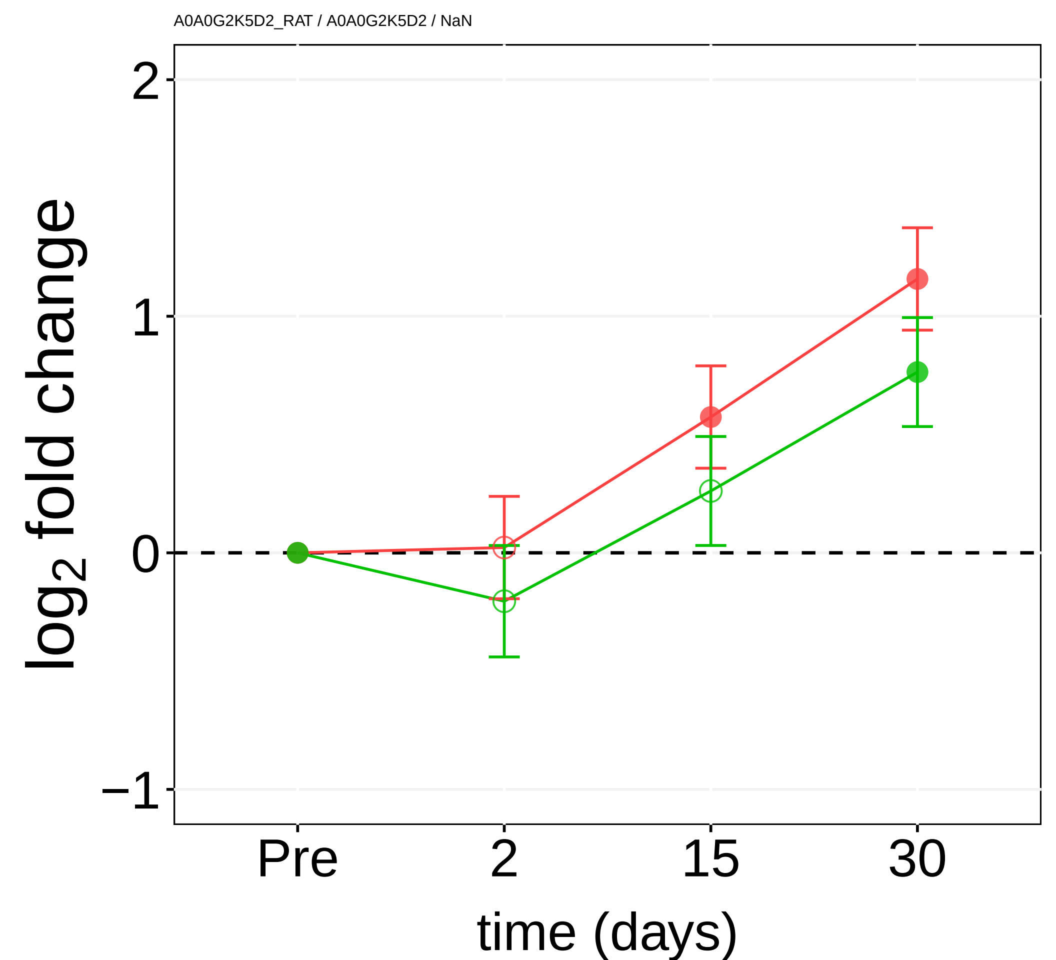

A0A0G2K5D2_RAT / A0A0G2K5D2 / NaN
log2 fold change
2
1
0
−1
Pre
2
15
30
time (days)

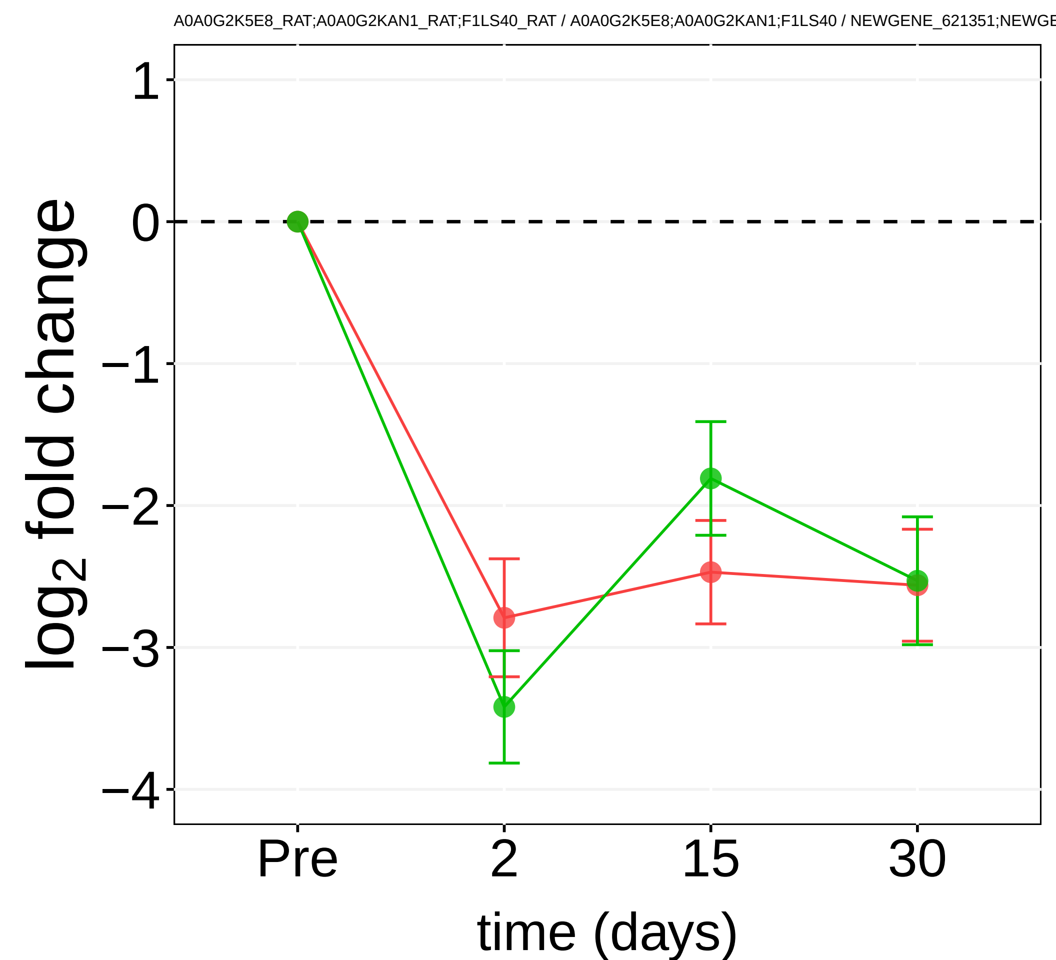
A0A0G2K5E8_RAT;A0A0G2KAN1_RAT;F1LS40_RAT / A0A0G2K5E8;A0A0G2KAN1;F1LS40 / NEWGENE_621351;NEWGE
log2 fold change
1
0
−1
−2
−3
−4
Pre
2
15
30
time (days)

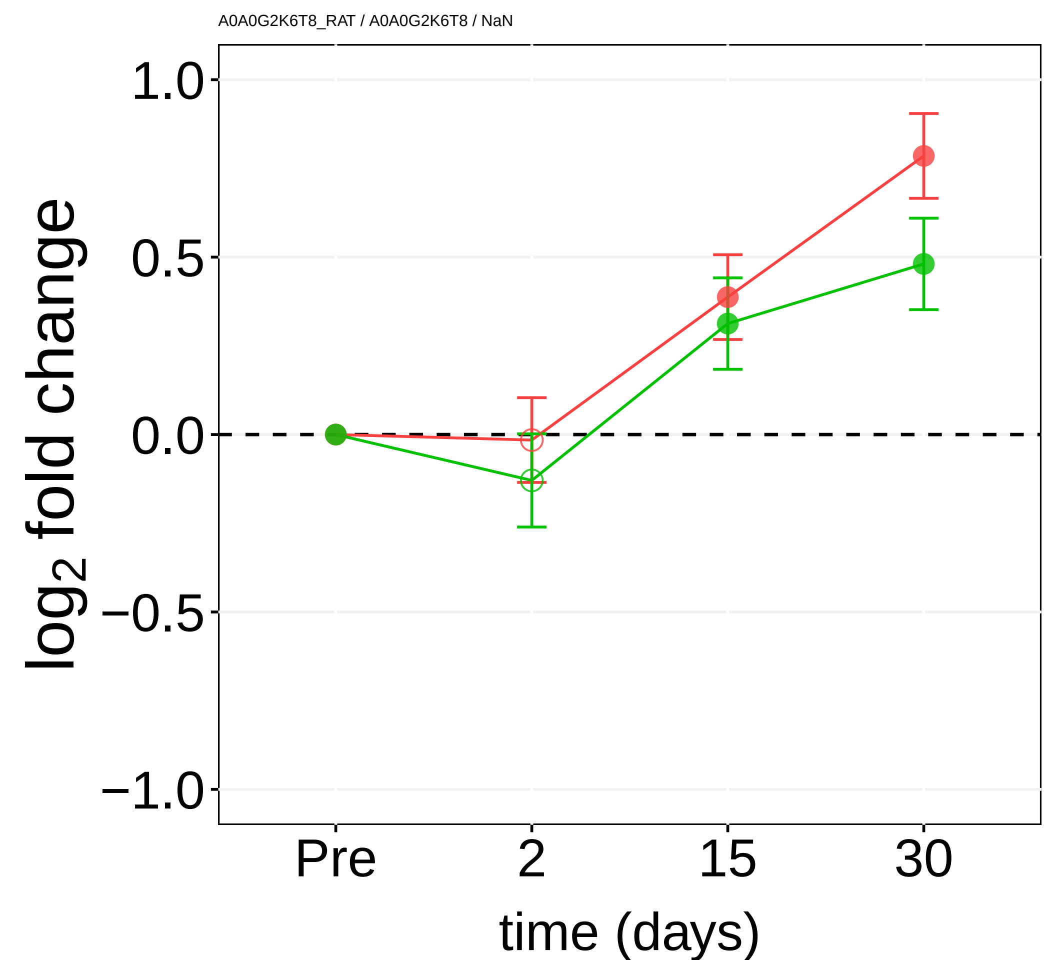

A0A0G2K6T8_RAT / A0A0G2K6T8 / NaN
1.0
0.5
0.0
−0.5
−1.0
log2 fold change
Pre
2
15
30
time (days)

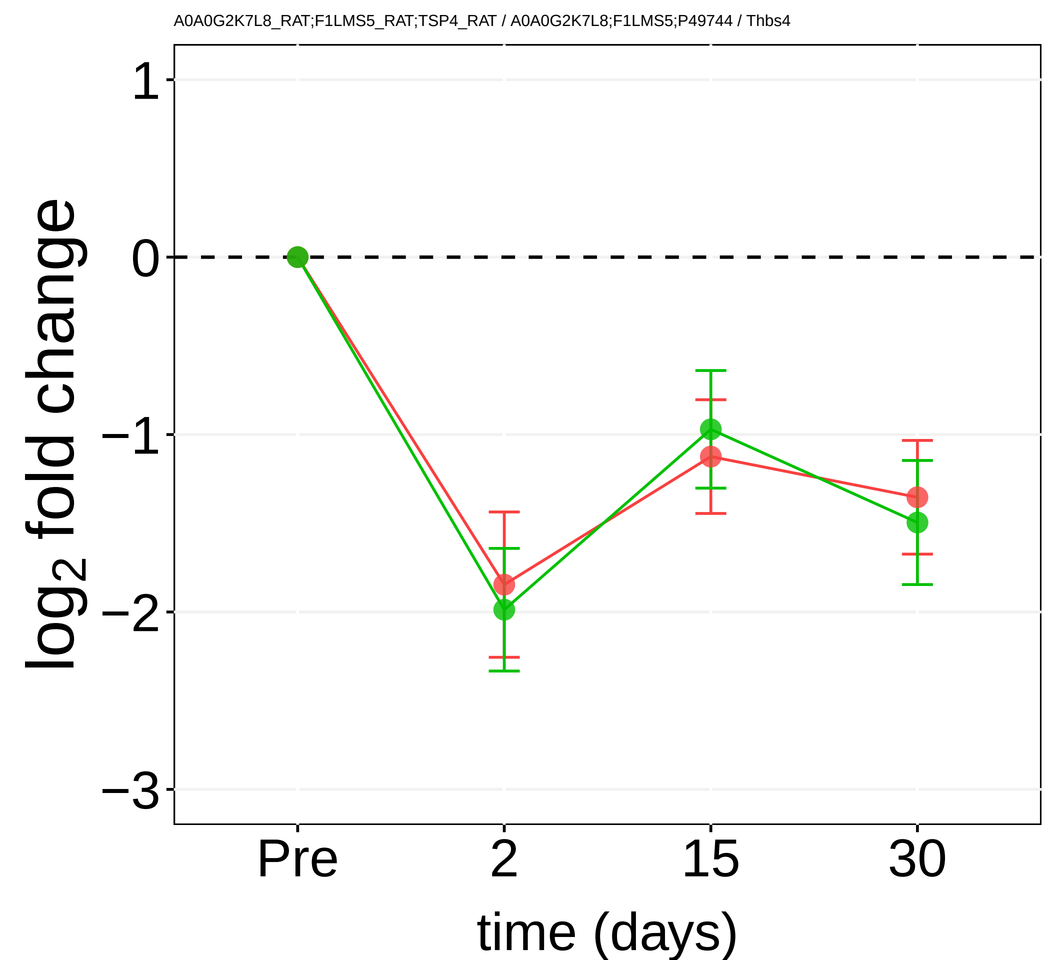

A0A0G2K7L8_RAT;F1LMS5_RAT;TSP4_RAT / A0A0G2K7L8;F1LMS5;P49744 / Thbs4
log2 fold change
1
0
−1
−2
−3
Pre
2
15
30
time (days)

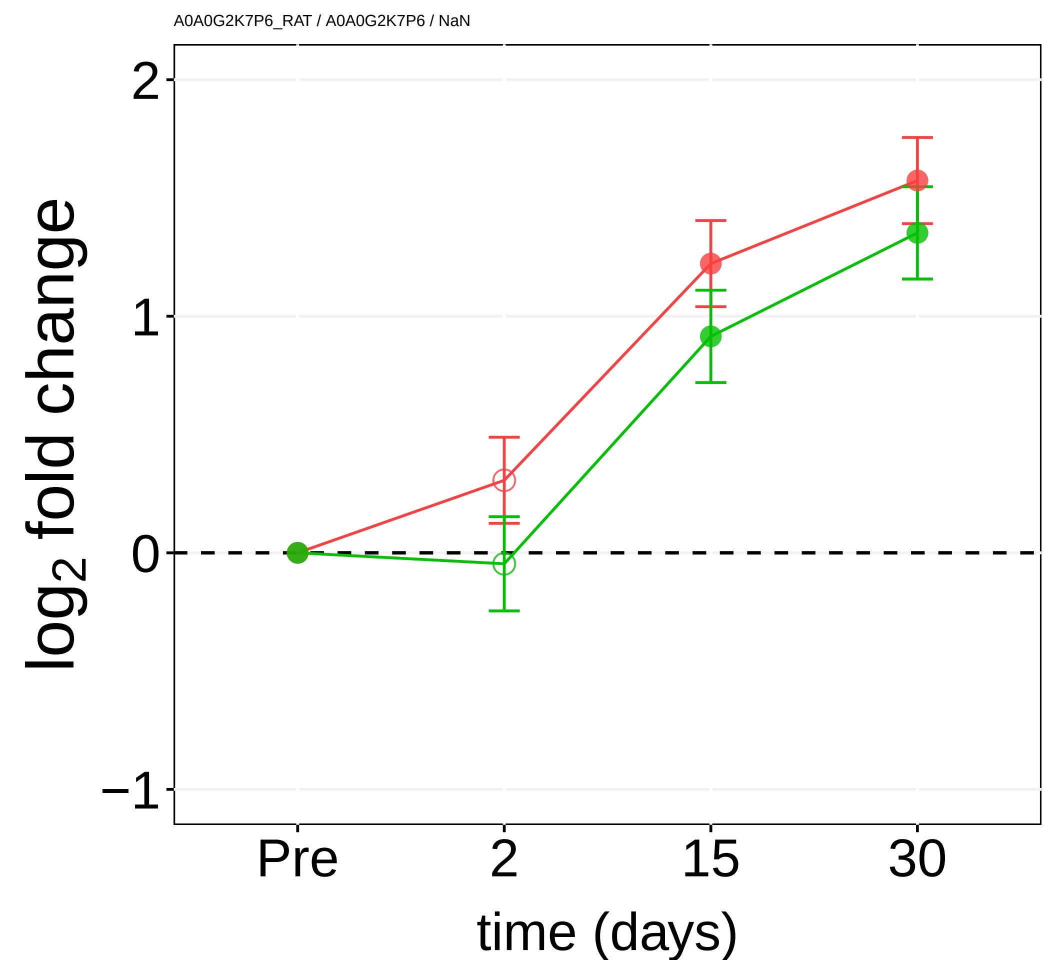

A0A0G2K7P6_RAT / A0A0G2K7P6 / NaN
2
1
0
−1
log2 fold change
Pre
2
15
30
time (days)

A0A0G2K7X7_RAT / A0A0G2K7X7 / C7

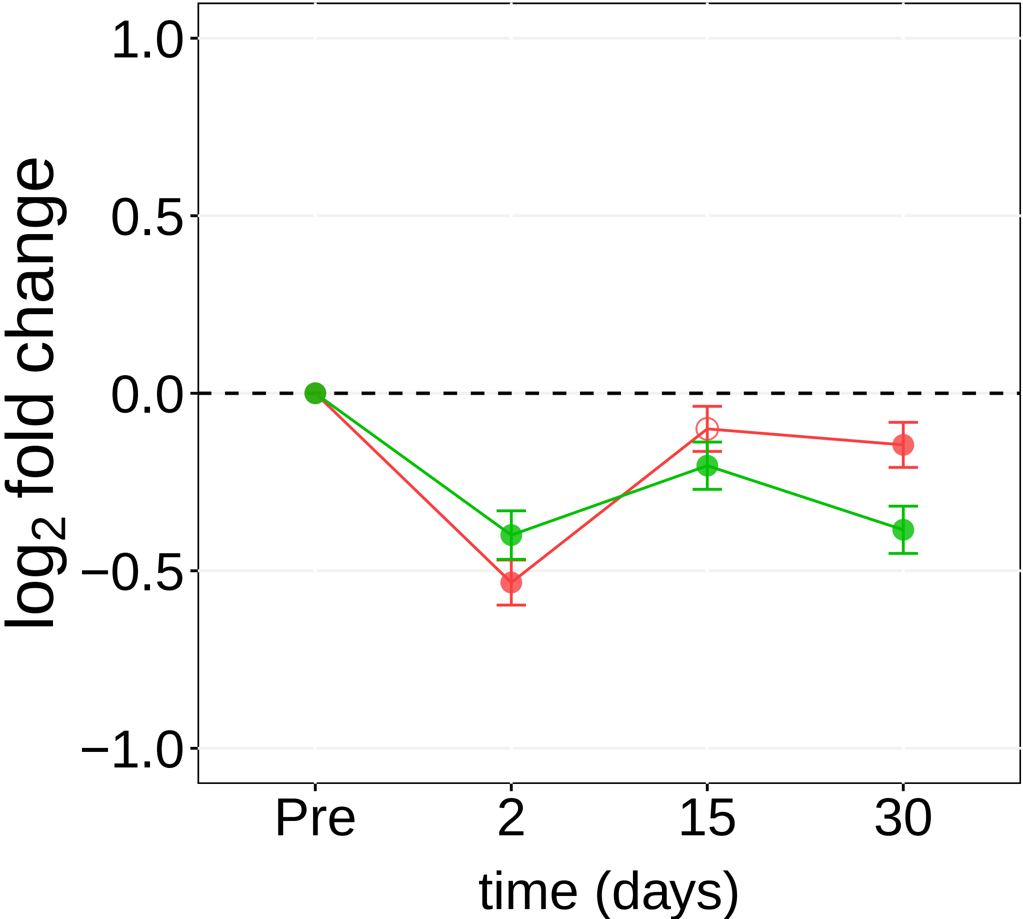

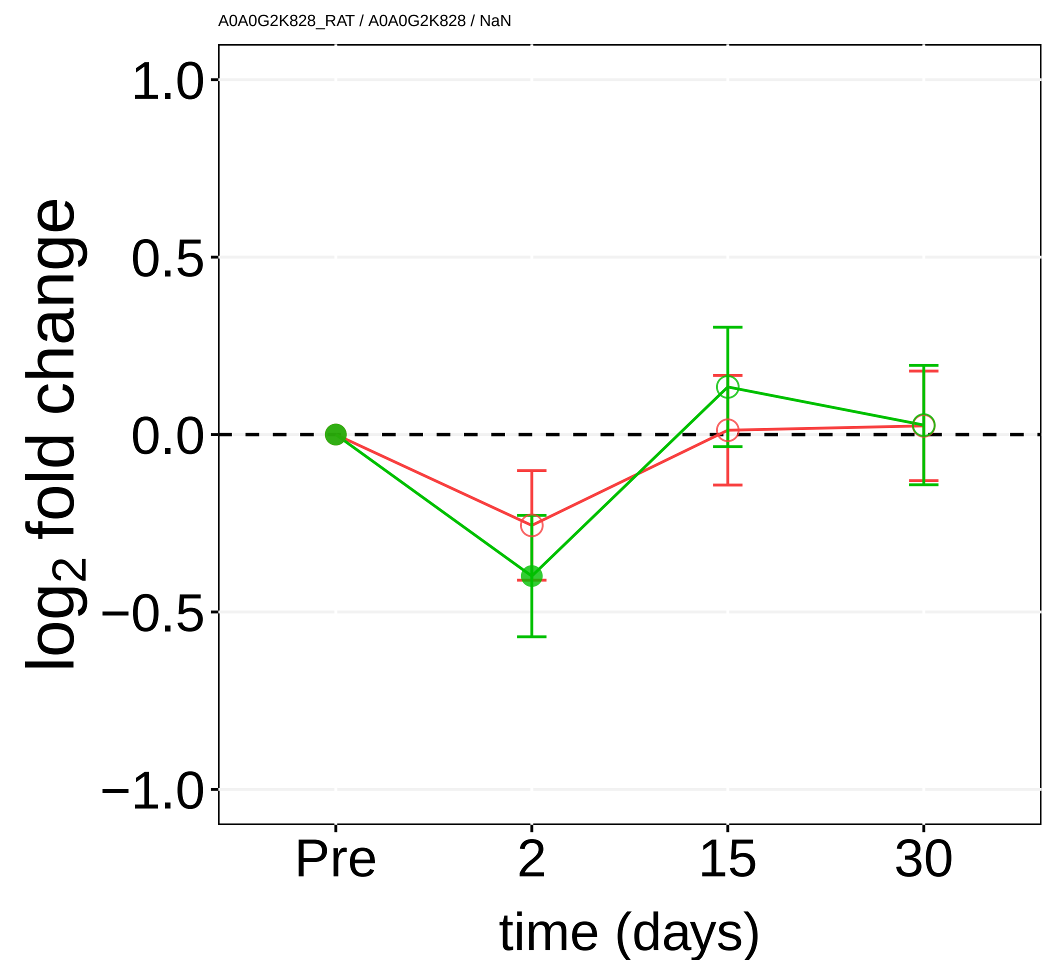

A0A0G2K828_RAT / A0A0G2K828 / NaN
log2 fold change
1.0
0.5
0.0
−0.5
−1.0
Pre
2
15
30
time (days)

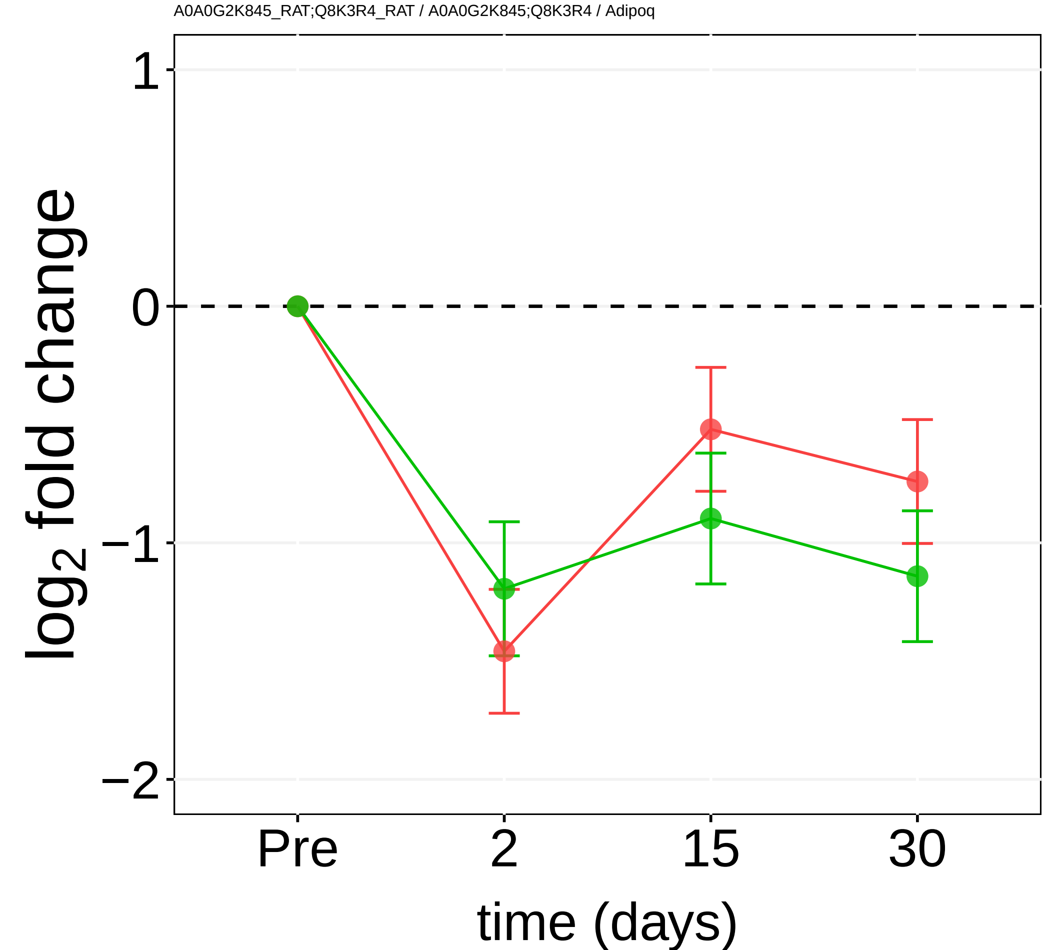

A0A0G2K845_RAT;Q8K3R4_RAT / A0A0G2K845;Q8K3R4 / Adipoq
log2 fold change
1
0
−1
−2
Pre
2
15
30
time (days)

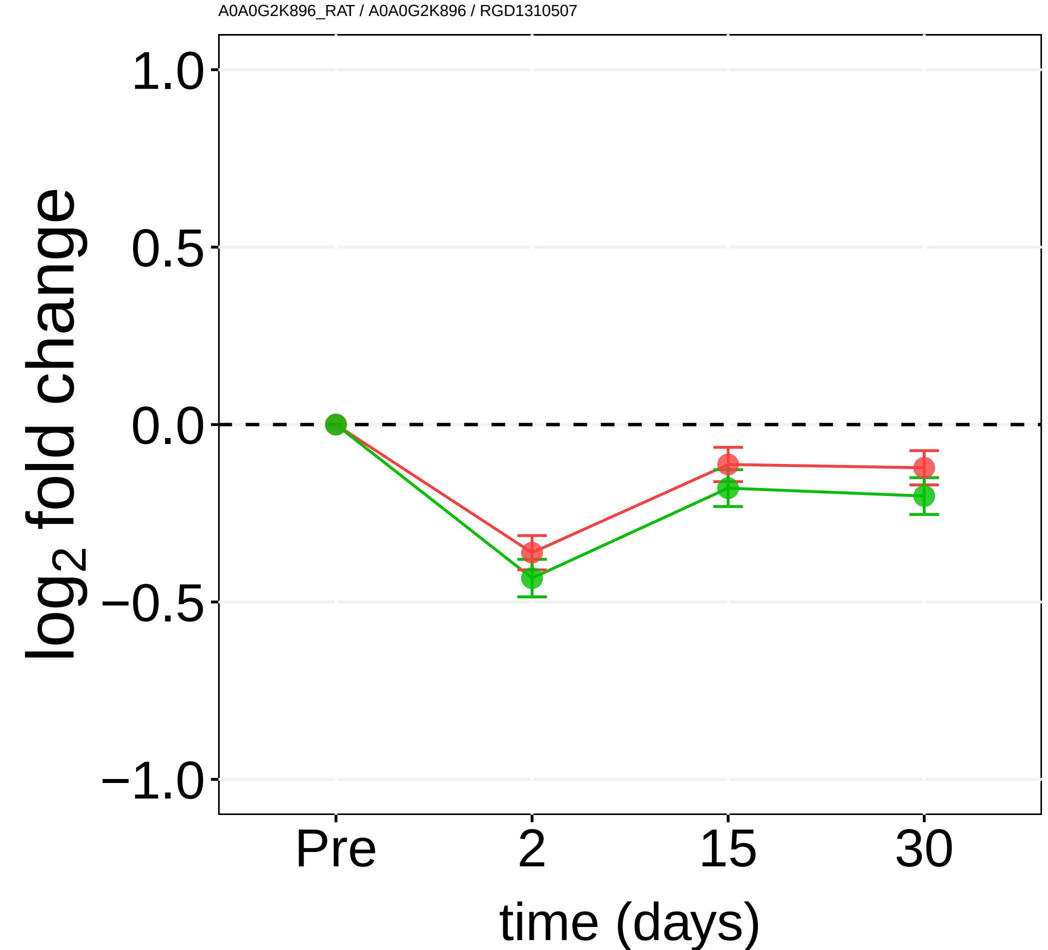
A0A0G2K896_RAT / A0A0G2K896 / RGD1310507
1.0
0.5
0.0
−0.5
−1.0
log2 fold change
Pre
2
15
30
time (days)

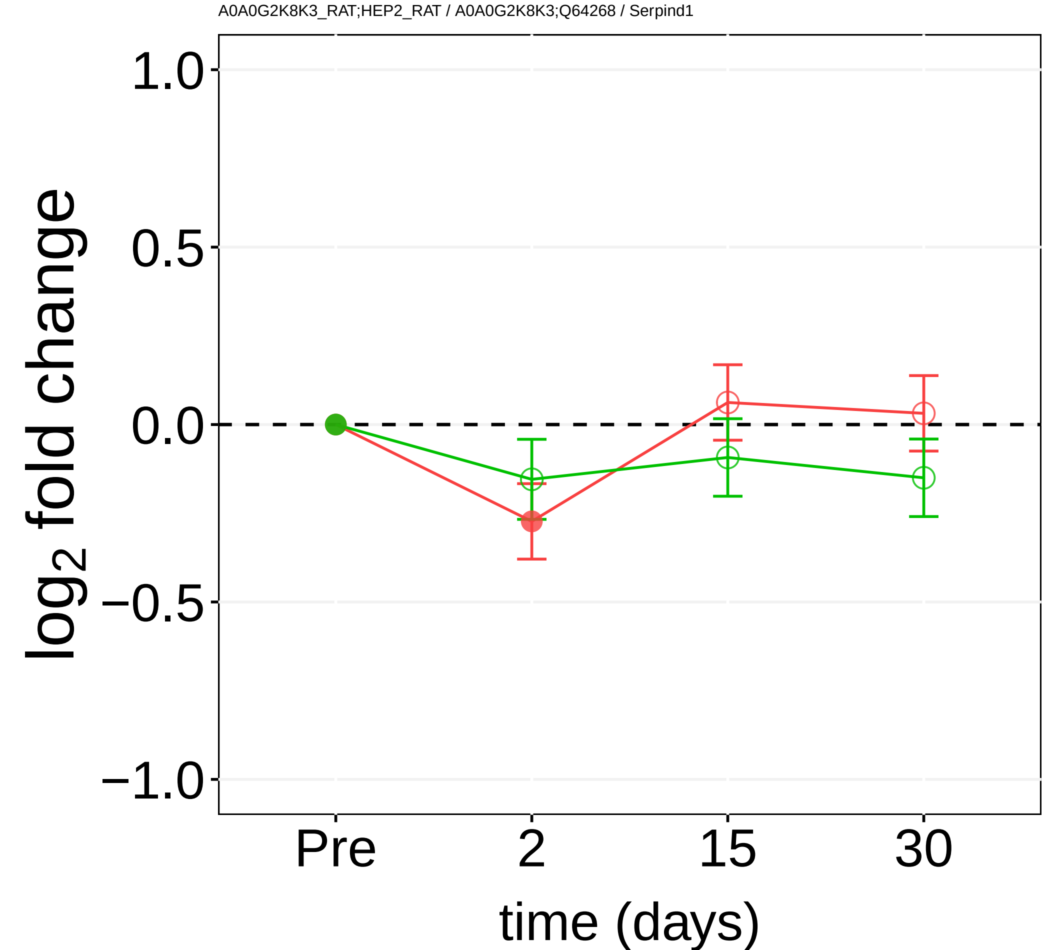
A0A0G2K8K3_RAT;HEP2_RAT / A0A0G2K8K3;Q64268 / Serpind1
1.0
0.5
0.0
−0.5
−1.0
log2 fold change
Pre
2
15
30
time (days)

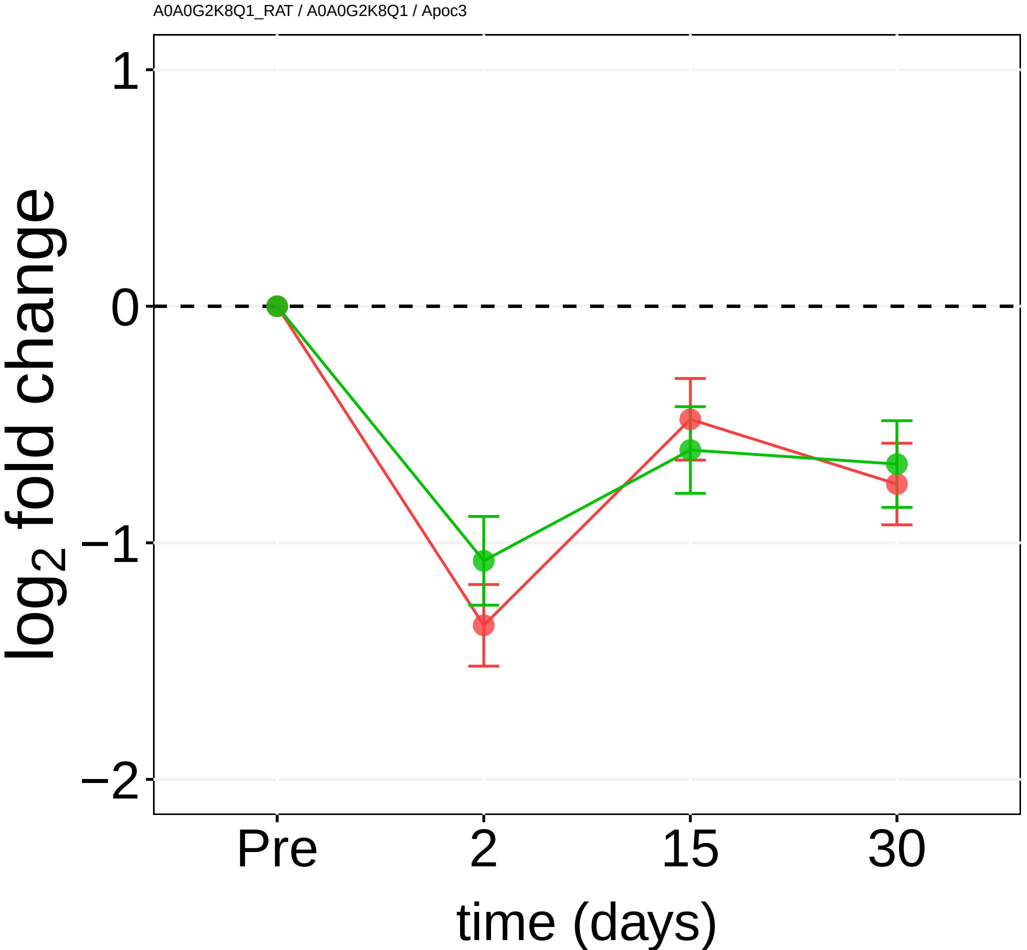

A0A0G2K8Q1_RAT / A0A0G2K8Q1 / Apoc3
1
0
−1
−2
log2 fold change
Pre
2
15
30
time (days)

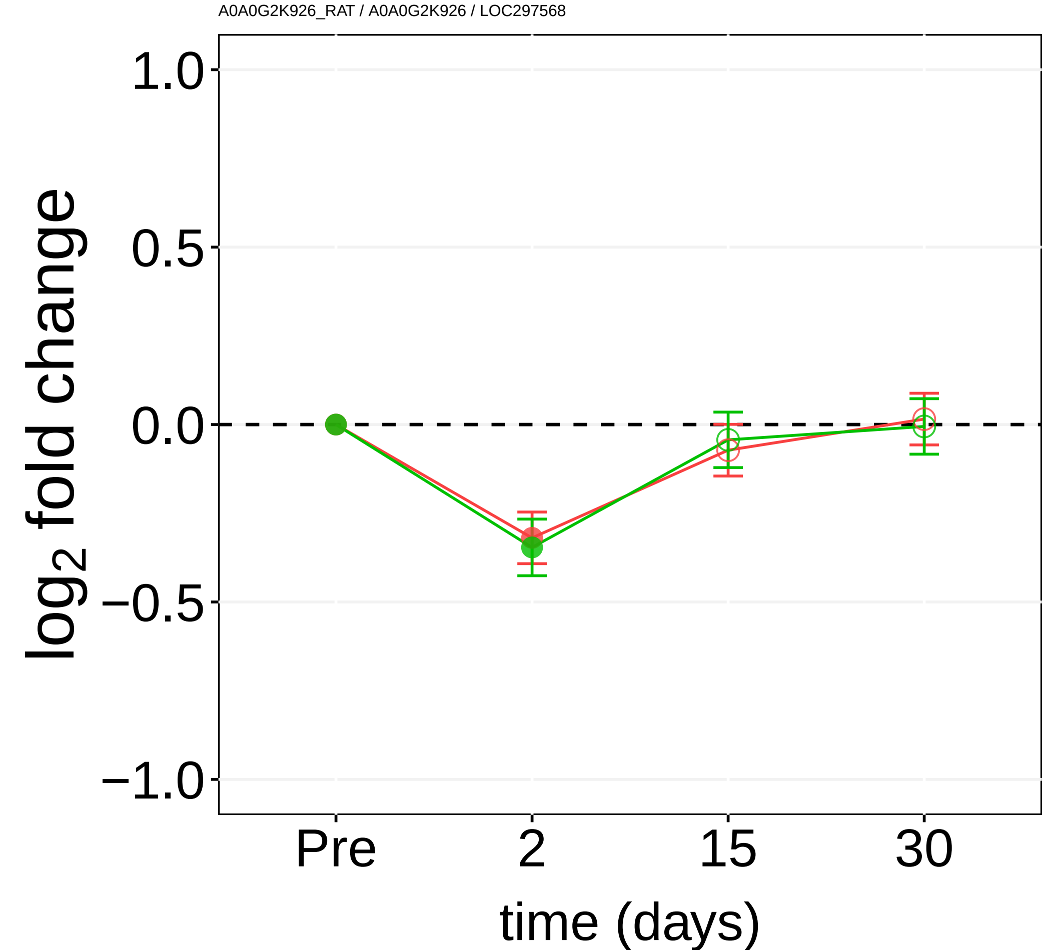

A0A0G2K926_RAT / A0A0G2K926 / LOC297568
1.0
0.5
0.0
−0.5
−1.0
log2 fold change
Pre
2
15
30
time (days)

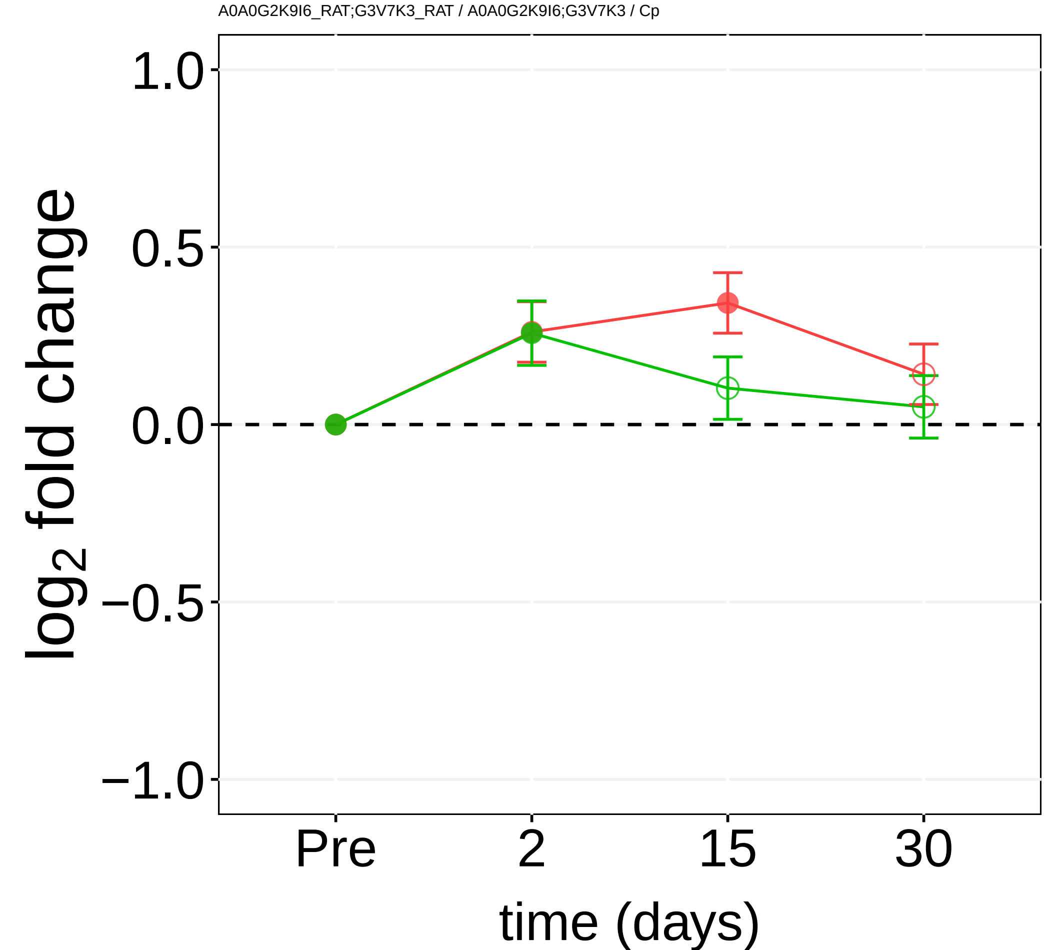
A0A0G2K9I6_RAT;G3V7K3_RAT / A0A0G2K9I6;G3V7K3 / Cp
log2 fold change
1.0
0.5
0.0
−0.5
−1.0
Pre
2
15
30
time (days)

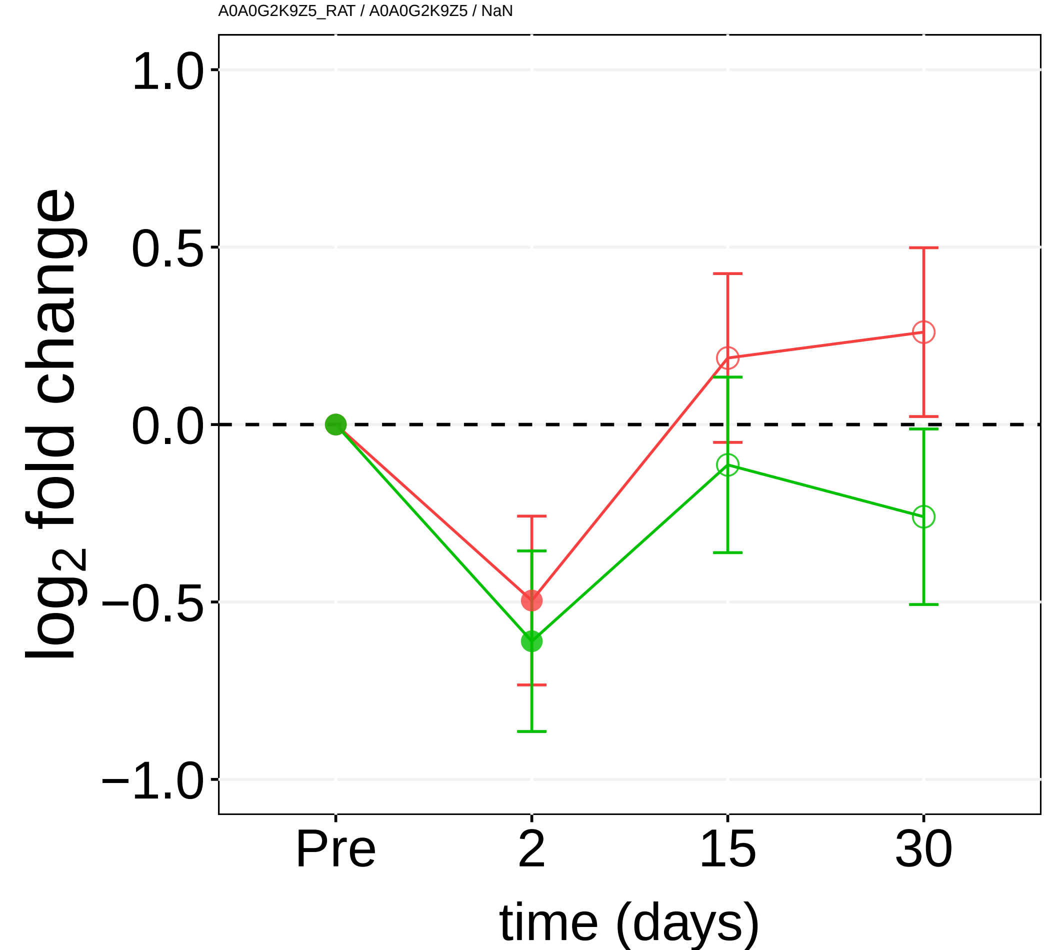
A0A0G2K9Z5_RAT / A0A0G2K9Z5 / NaN
1.0
0.5
0.0
−0.5
−1.0
log2 fold change
Pre
2
15
30
time (days)

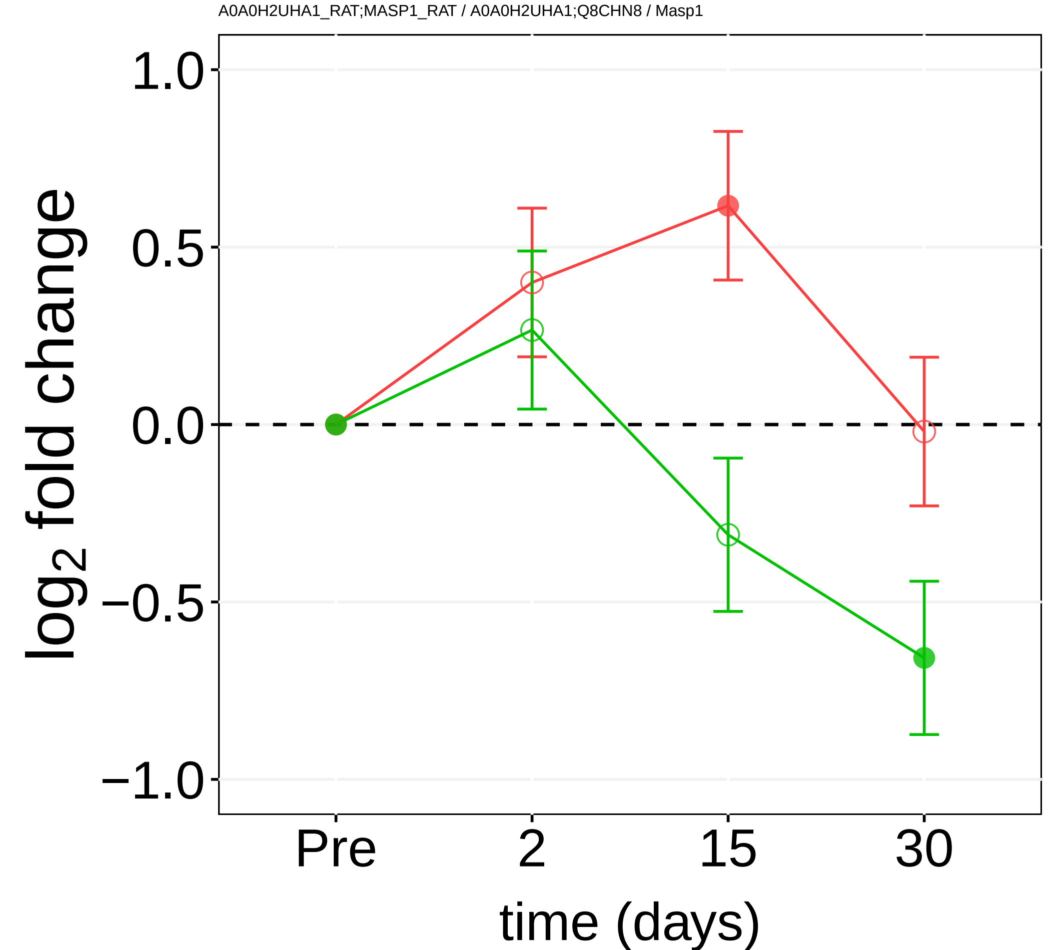
A0A0H2UHA1_RAT;MASP1_RAT / A0A0H2UHA1;Q8CHN8 / Masp1
1.0
0.5
0.0
−0.5
−1.0
log2 fold change
Pre
2
15
30
time (days)

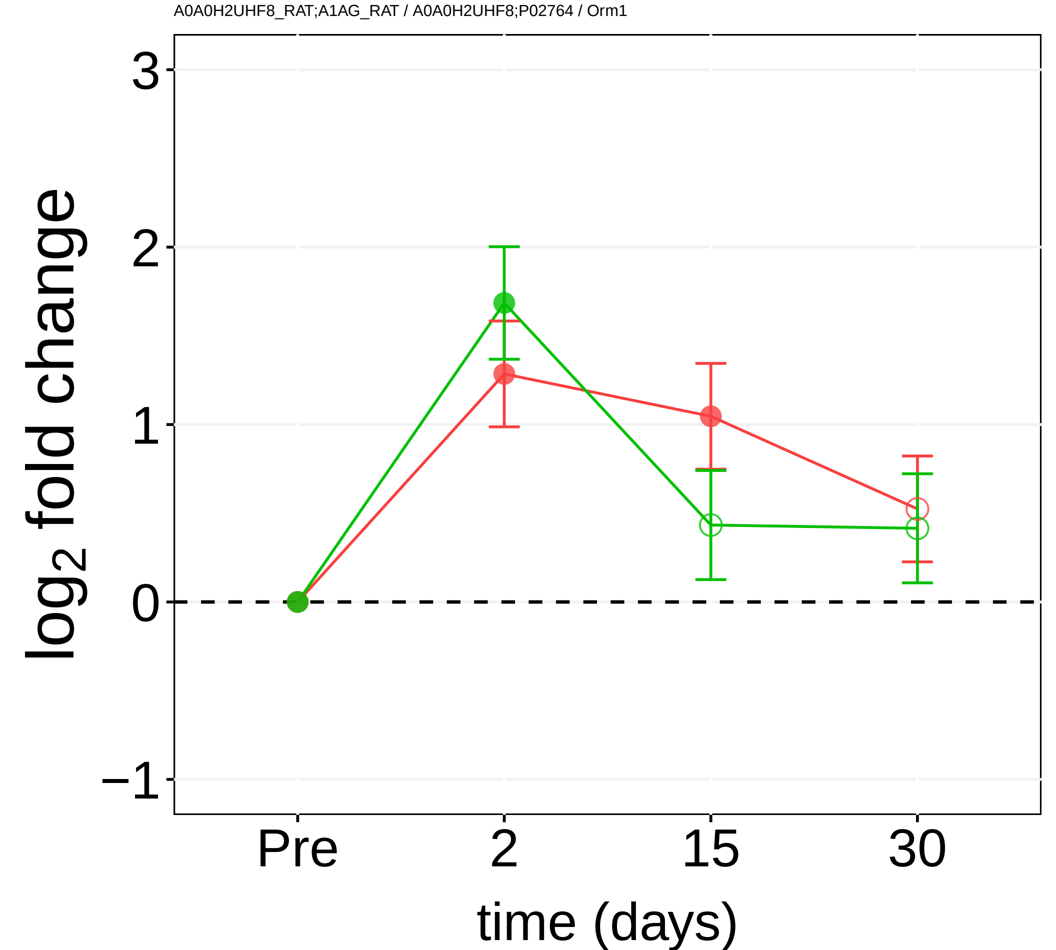
A0A0H2UHF8_RAT;A1AG_RAT / A0A0H2UHF8;P02764 / Orm1
3
2
1
0
−1
log2 fold change
Pre
2
15
30
time (days)

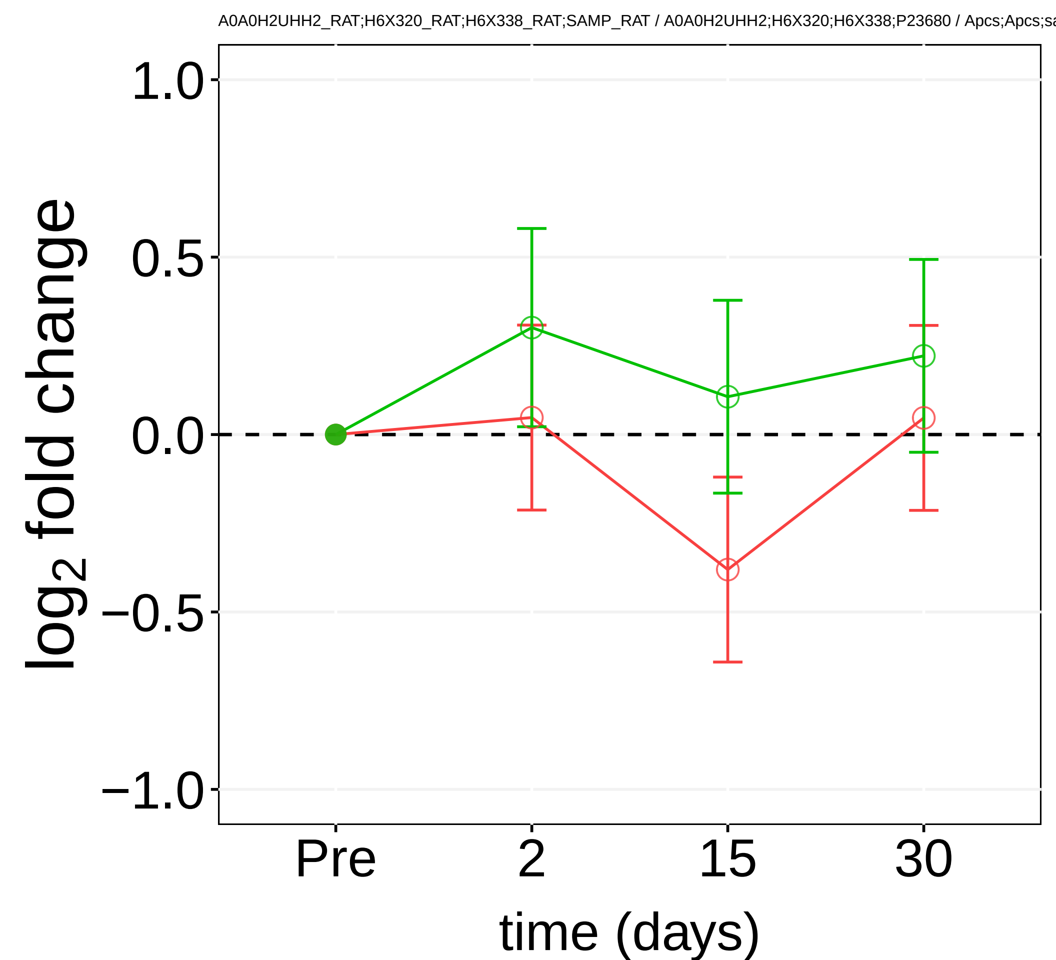
A0A0H2UHH2_RAT;H6X320_RAT;H6X338_RAT;SAMP_RAT / A0A0H2UHH2;H6X320;H6X338;P23680 / Apcs;Apcs;sa
1.0
0.5
0.0
−0.5
−1.0
log2 fold change
Pre
2
15
30
time (days)

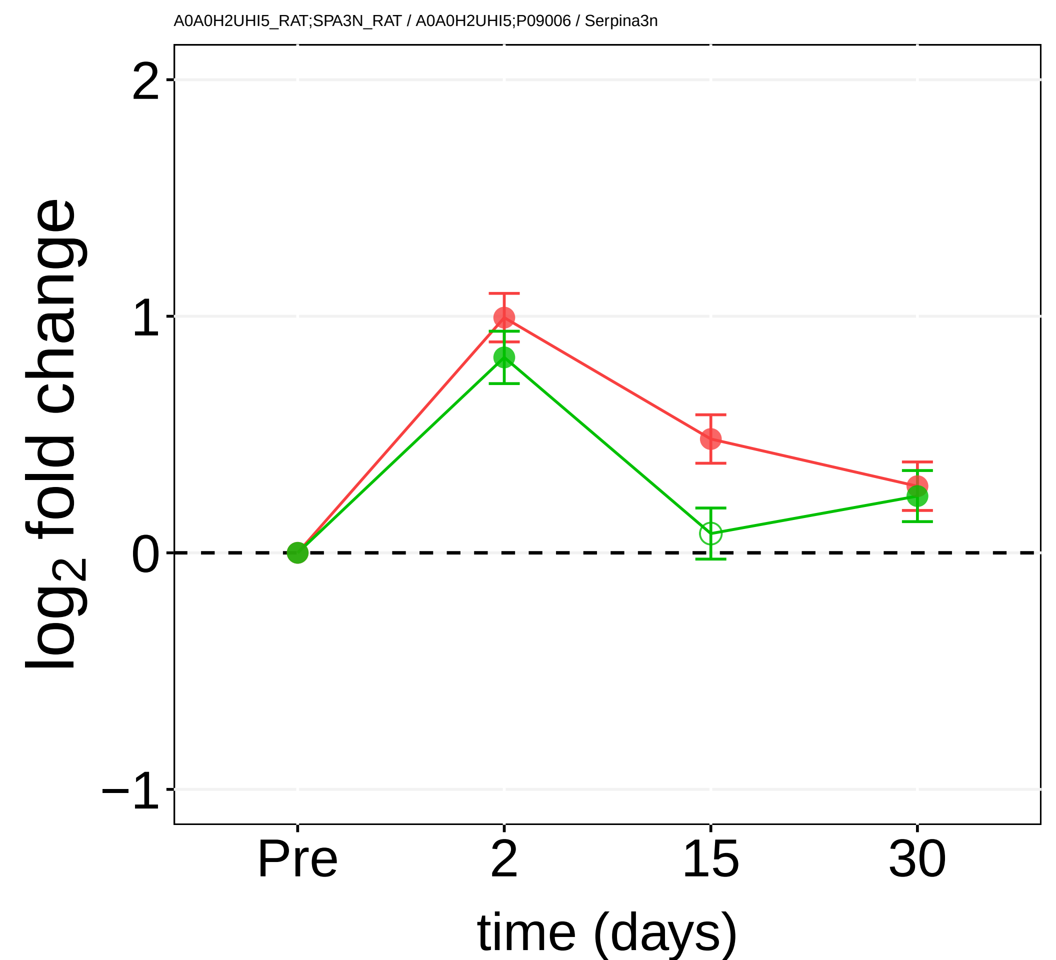

A0A0H2UHI5_RAT;SPA3N_RAT / A0A0H2UHI5;P09006 / Serpina3n
2
1
0
−1
log2 fold change
Pre
2
15
30
time (days)

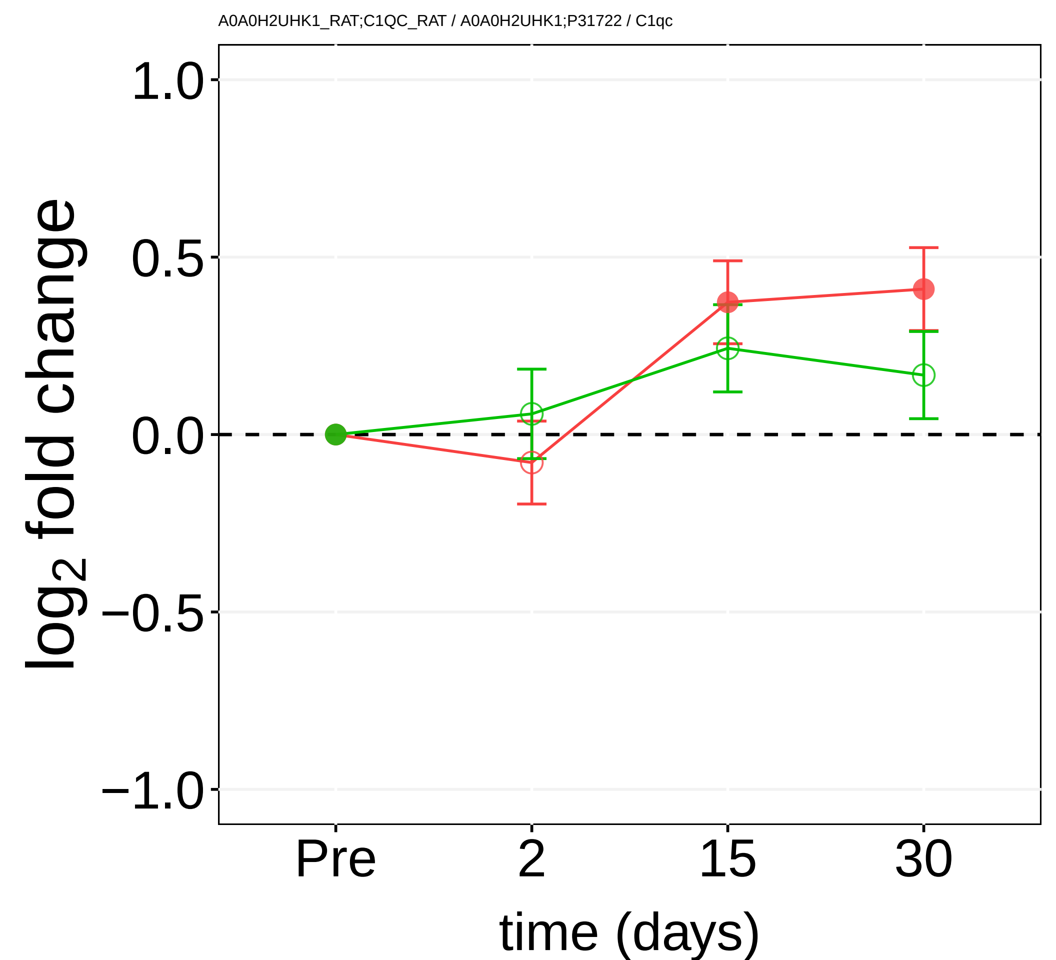

A0A0H2UHK1_RAT;C1QC_RAT / A0A0H2UHK1;P31722 / C1qc
1.0
0.5
0.0
−0.5
−1.0
log2 fold change
Pre
2
15
30
time (days)

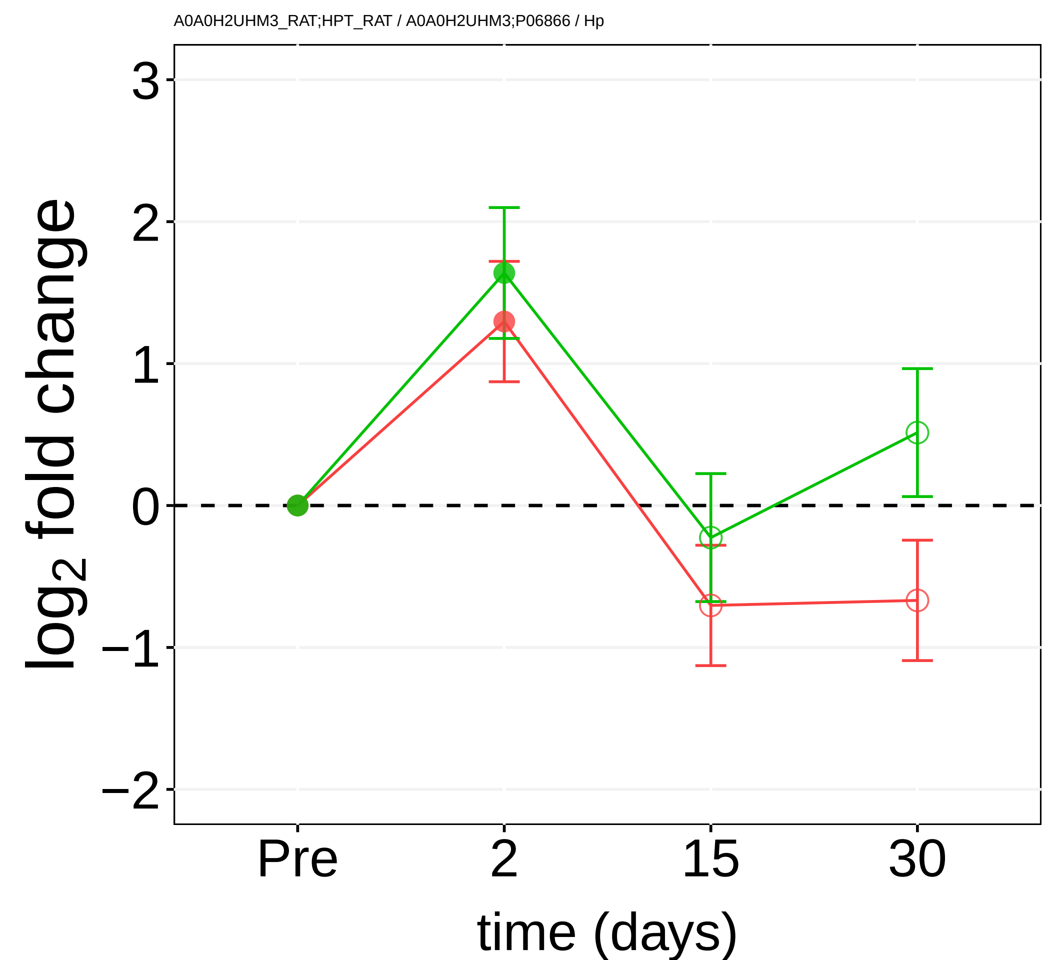
A0A0H2UHM3_RAT;HPT_RAT / A0A0H2UHM3;P06866 / Hp
log2 fold change
3
2
1
0
−1
−2
Pre
2
15
30
time (days)

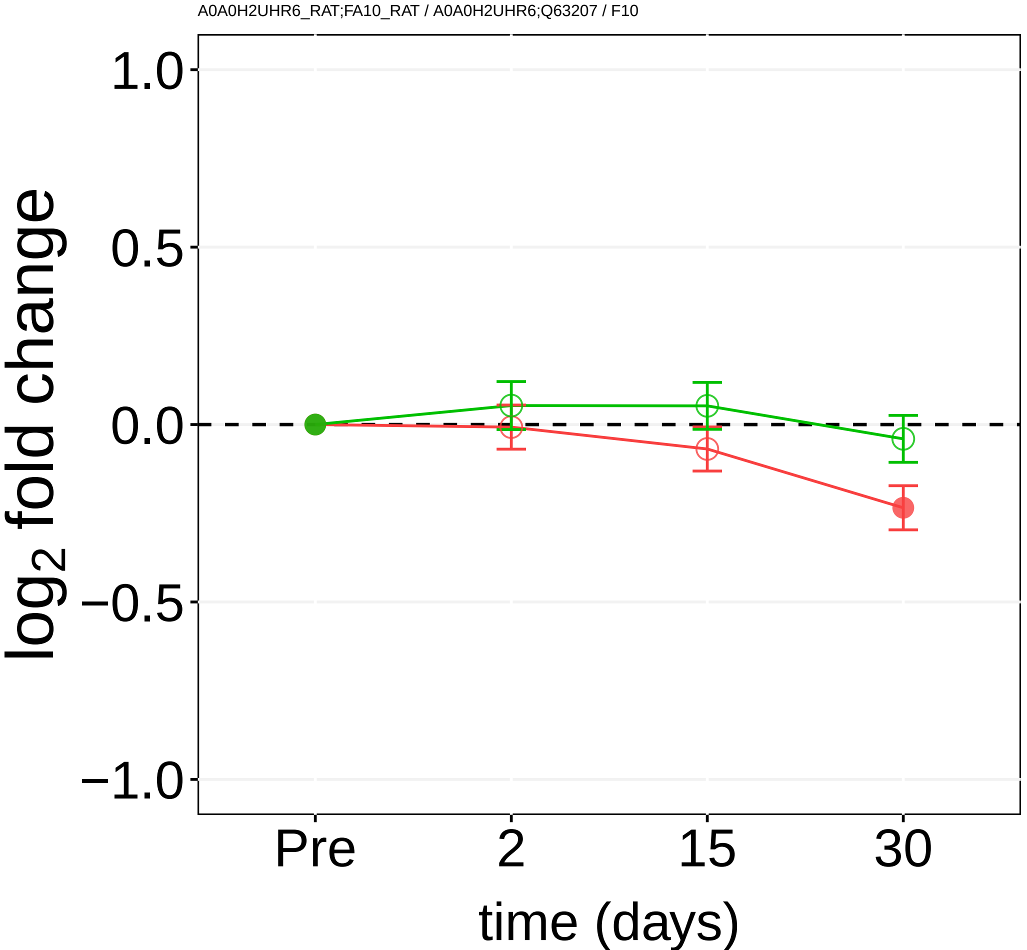
A0A0H2UHR6_RAT;FA10_RAT / A0A0H2UHR6;Q63207 / F10
1.0
0.5
0.0
−0.5
−1.0
log2 fold change
Pre
2
15
30
time (days)

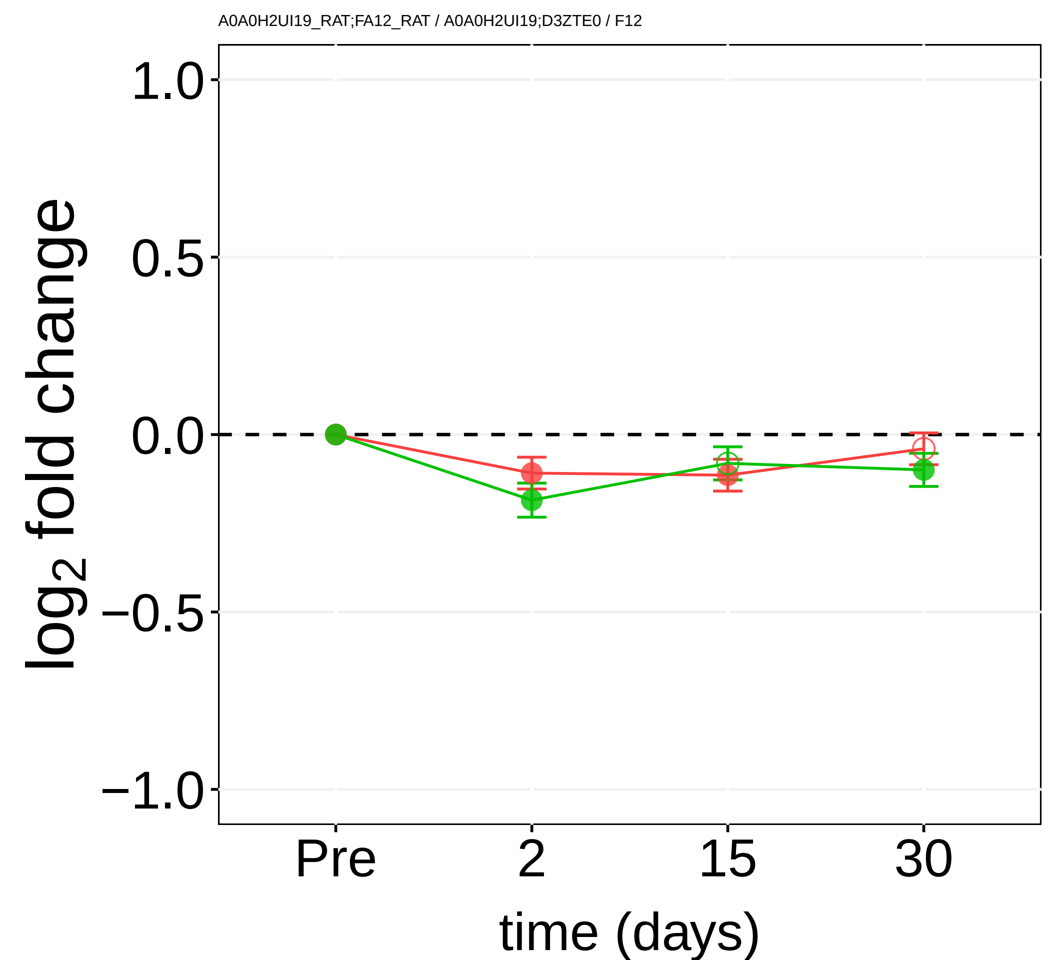

A0A0H2UI19_RAT;FA12_RAT / A0A0H2UI19;D3ZTE0 / F12
1.0
0.5
0.0
−0.5
−1.0
log2 fold change
Pre
2
15
30
time (days)

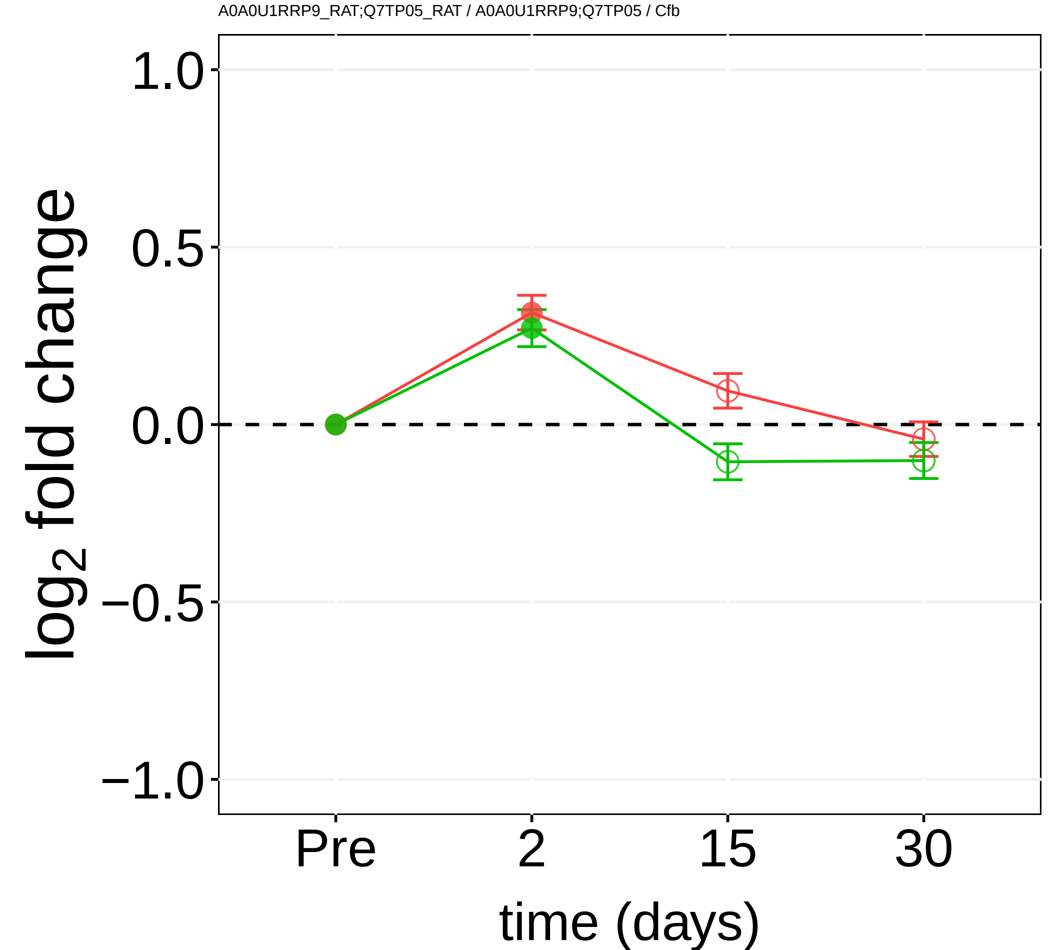
A0A0U1RRP9_RAT;Q7TP05_RAT / A0A0U1RRP9;Q7TP05 / Cfb
1.0
0.5
0.0
−0.5
−1.0
log2 fold change
Pre
2
15
30
time (days)

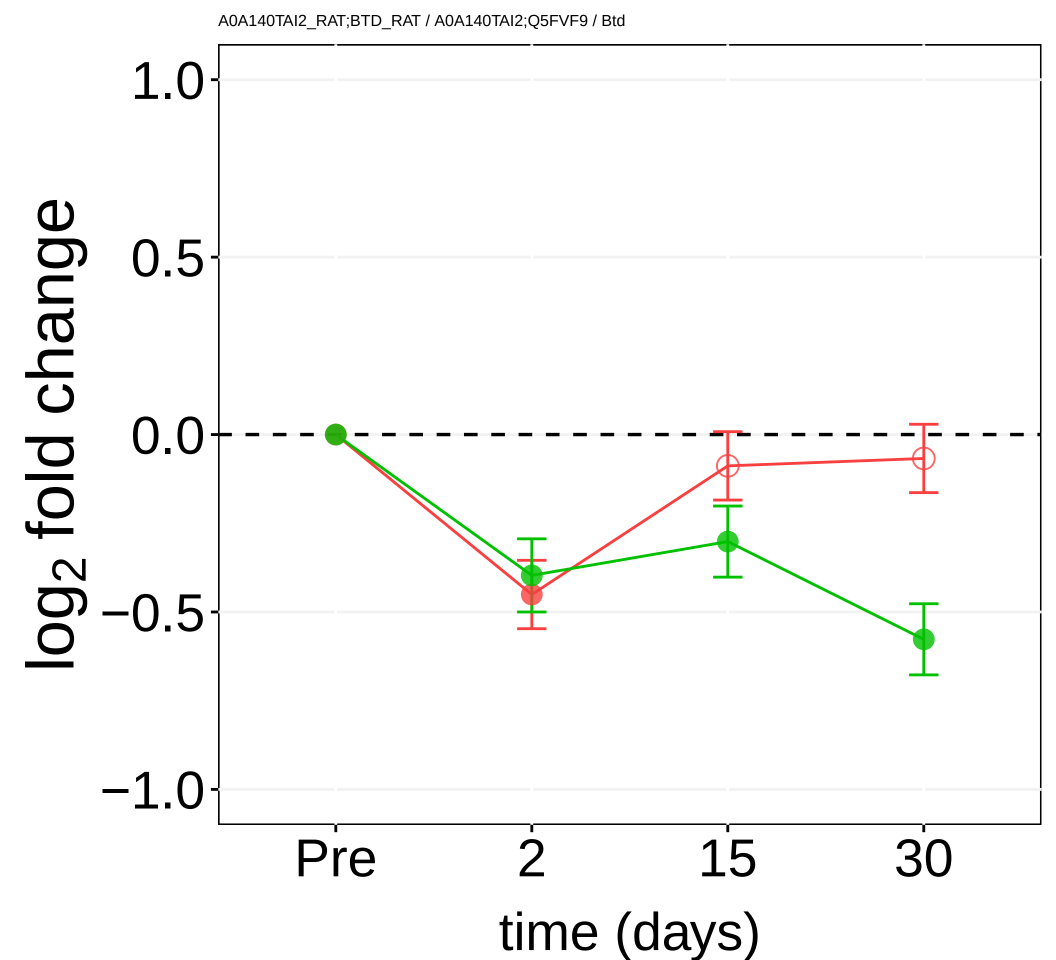
A0A140TAI2_RAT;BTD_RAT / A0A140TAI2;Q5FVF9 / Btd
1.0
0.5
0.0
−0.5
−1.0
log2 fold change
Pre
2
15
30
time (days)

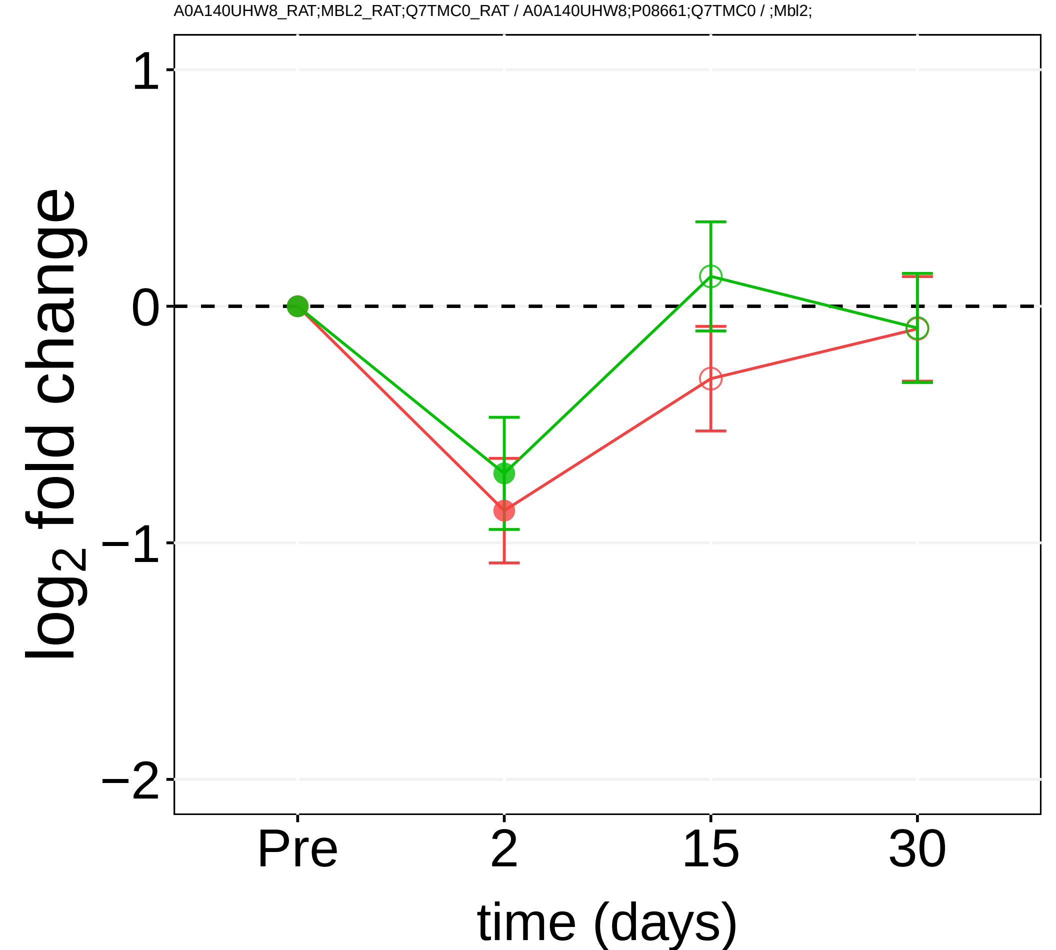
A0A140UHW8_RAT;MBL2_RAT;Q7TMC0_RAT / A0A140UHW8;P08661;Q7TMC0 / ;Mbl2;
1
0
−1
−2
log2 fold change
Pre
2
15
30
time (days)

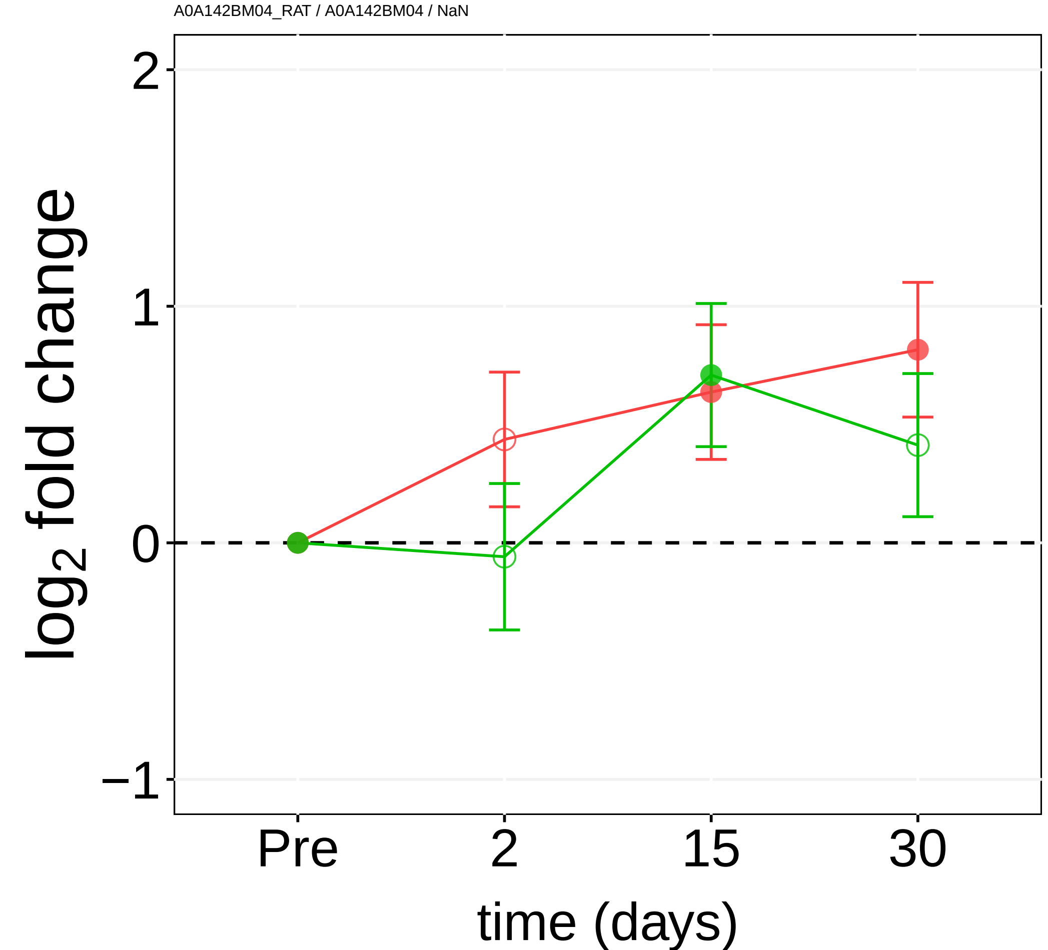

A0A142BM04_RAT / A0A142BM04 / NaN
2
1
0
−1
$\log_2$ fold change
Pre
2
15
30
time (days)

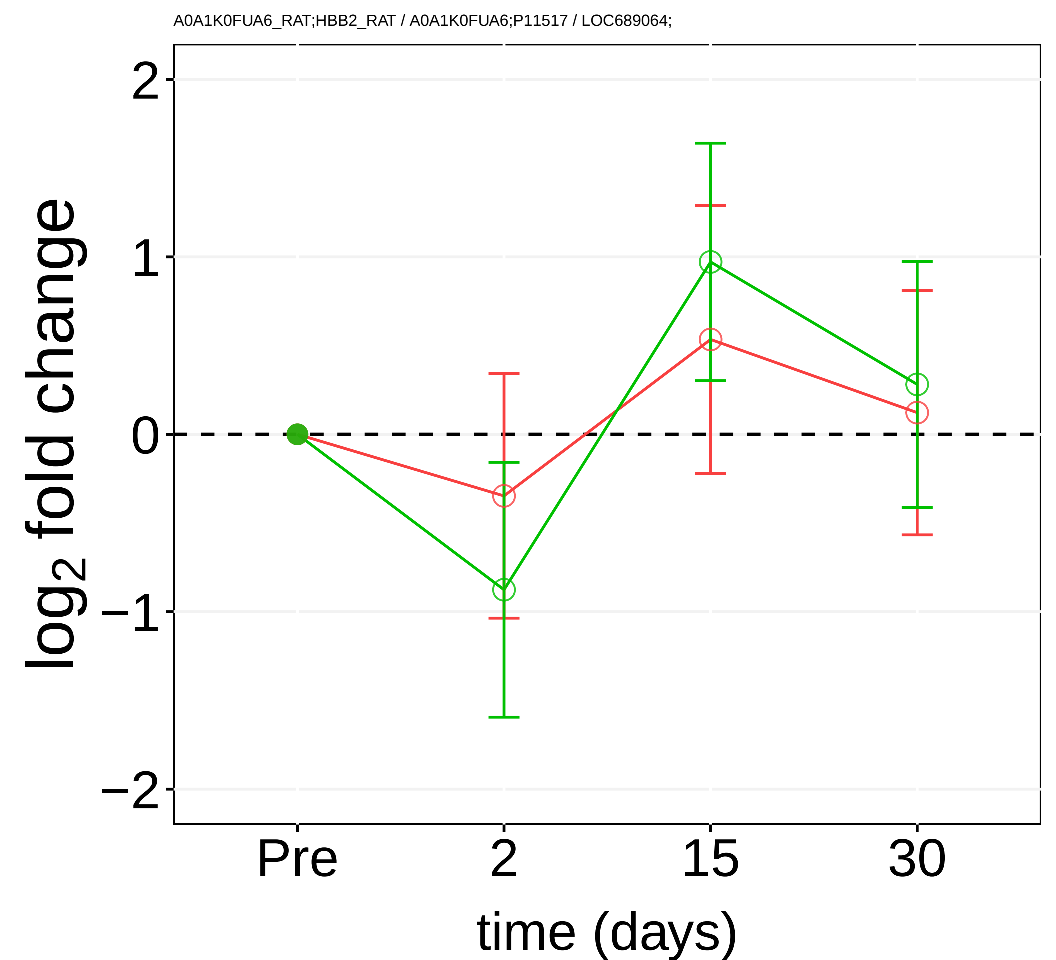

A0A1K0FUA6_RAT;HBB2_RAT / A0A1K0FUA6;P11517 / LOC689064;
2
1
0
−1
−2
log2 fold change
Pre
2
15
30
time (days)

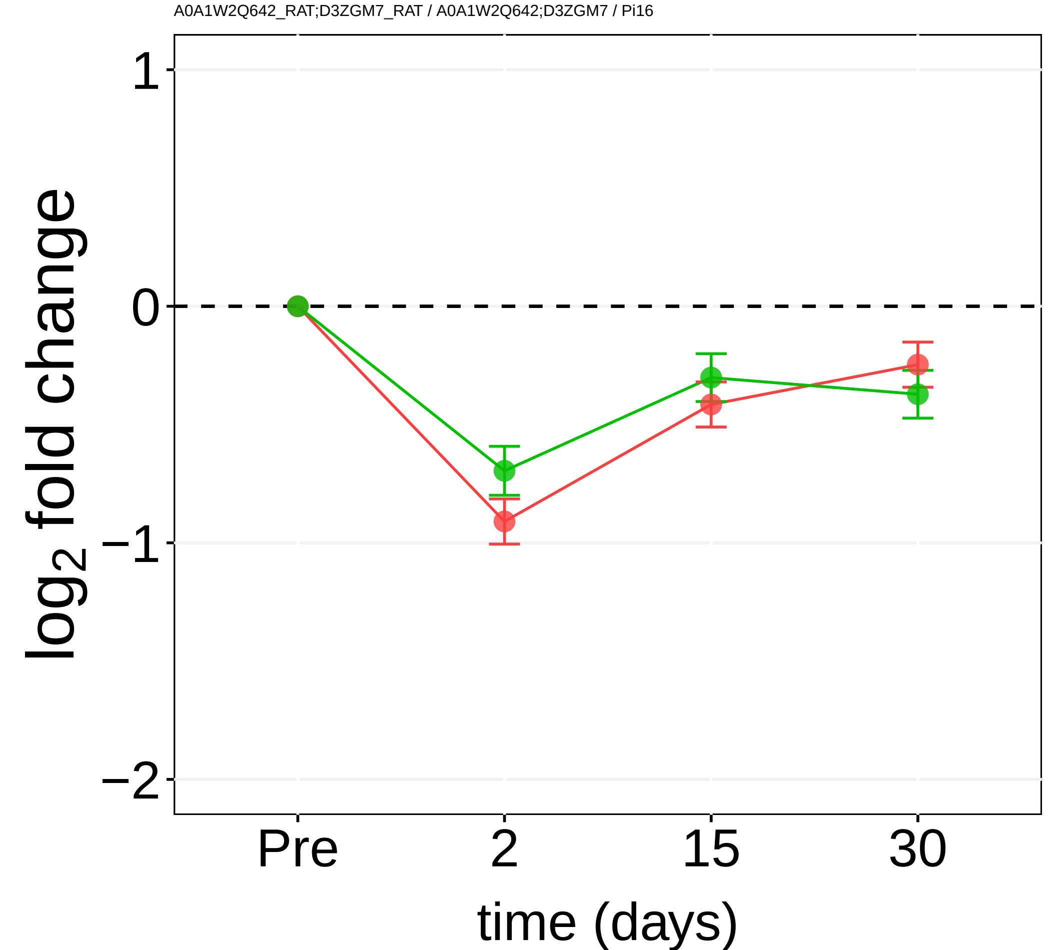
A0A1W2Q642_RAT;D3ZGM7_RAT / A0A1W2Q642;D3ZGM7 / Pi16
1
0
−1
−2
log2 fold change
Pre
2
15
30
time (days)

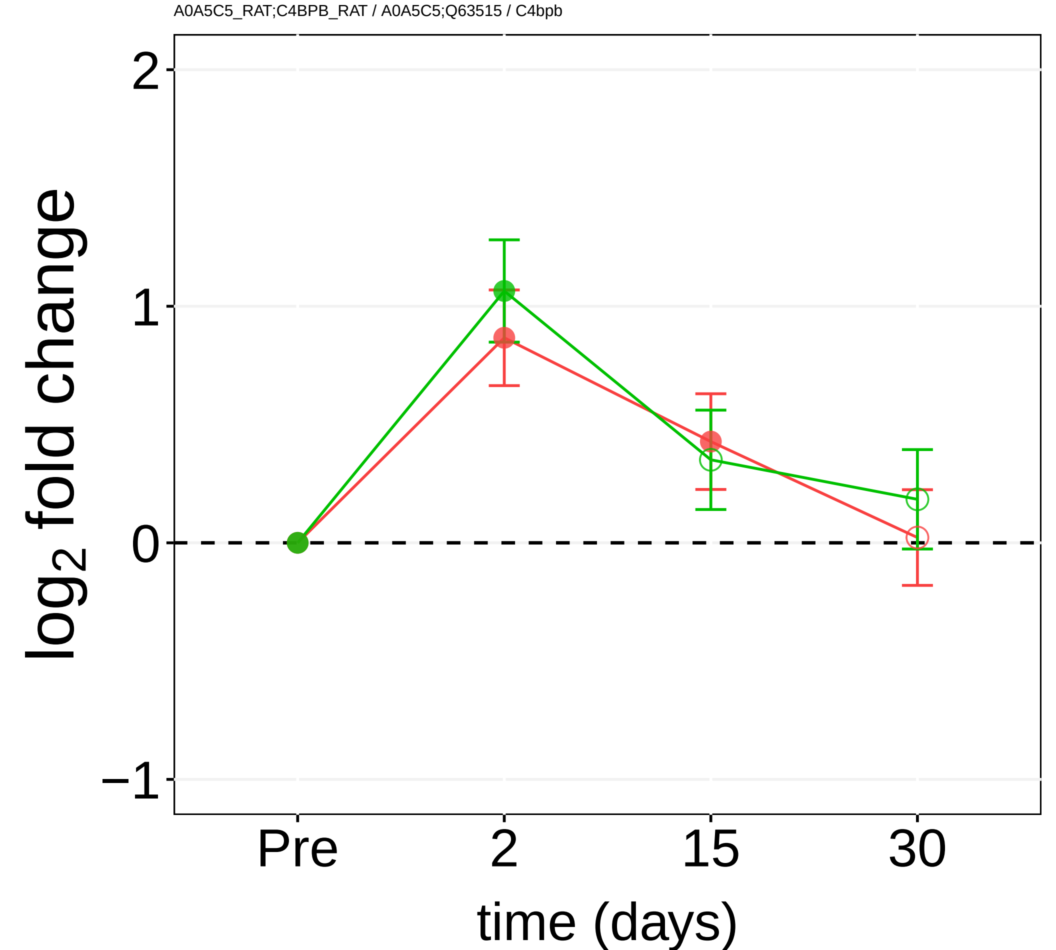

A0A5C5_RAT;C4BPB_RAT / A0A5C5;Q63515 / C4bpb
log2 fold change
2
1
0
−1
Pre
2
15
30
time (days)

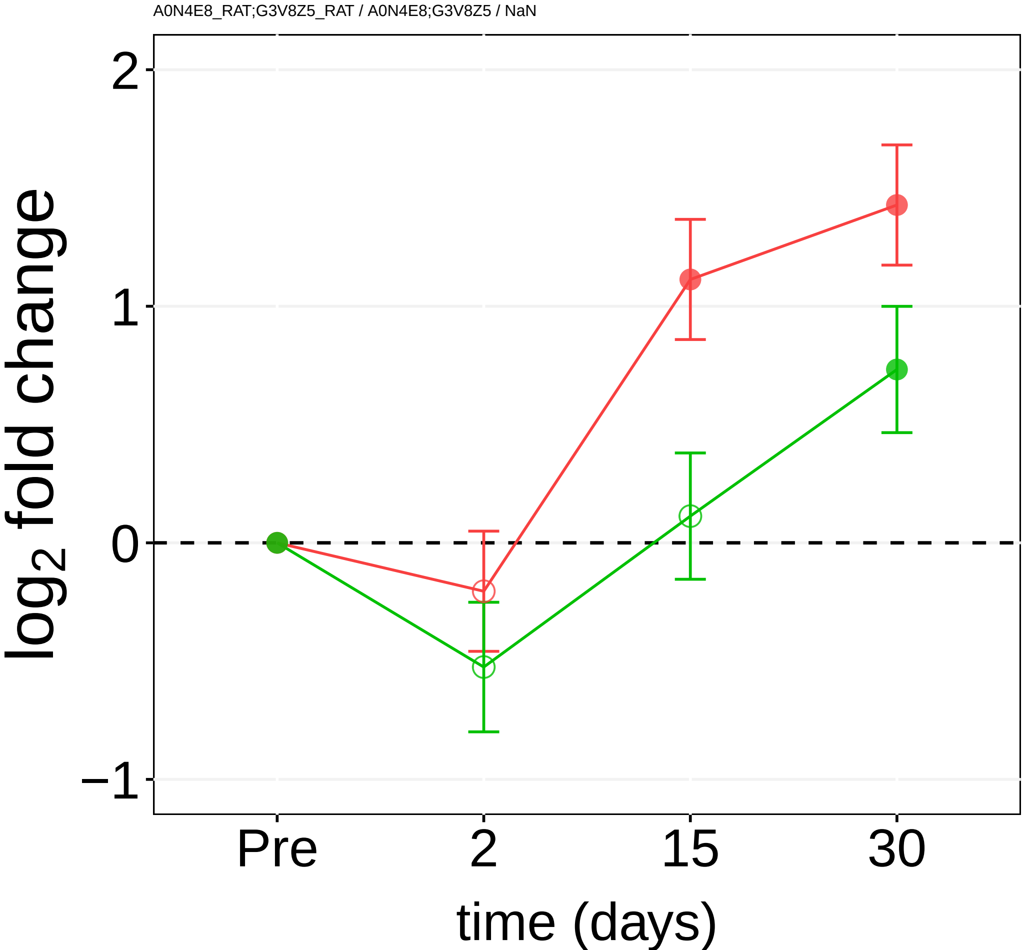

A0N4E8_RAT;G3V8Z5_RAT / A0N4E8;G3V8Z5 / NaN
2
1
0
−1
$\log_2$ fold change
Pre
2
15
30
time (days)

A2VCV7_RAT / A2VCV7 / Masp2

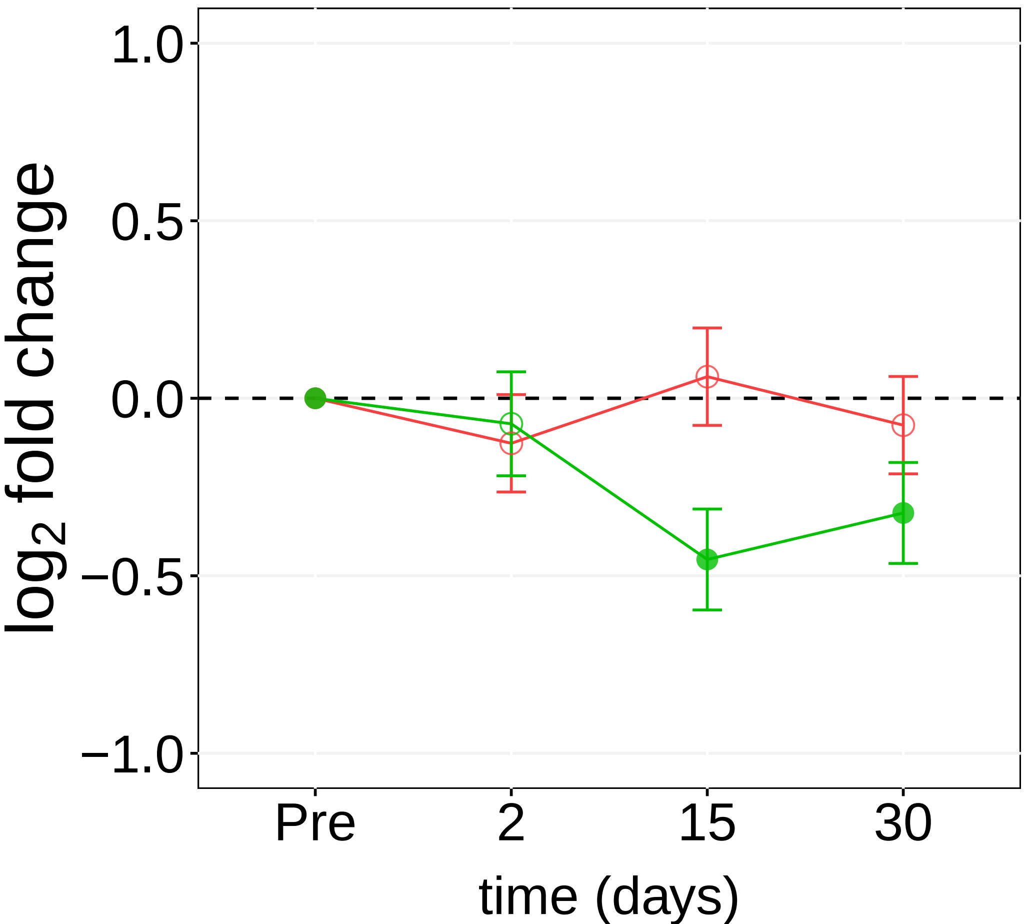

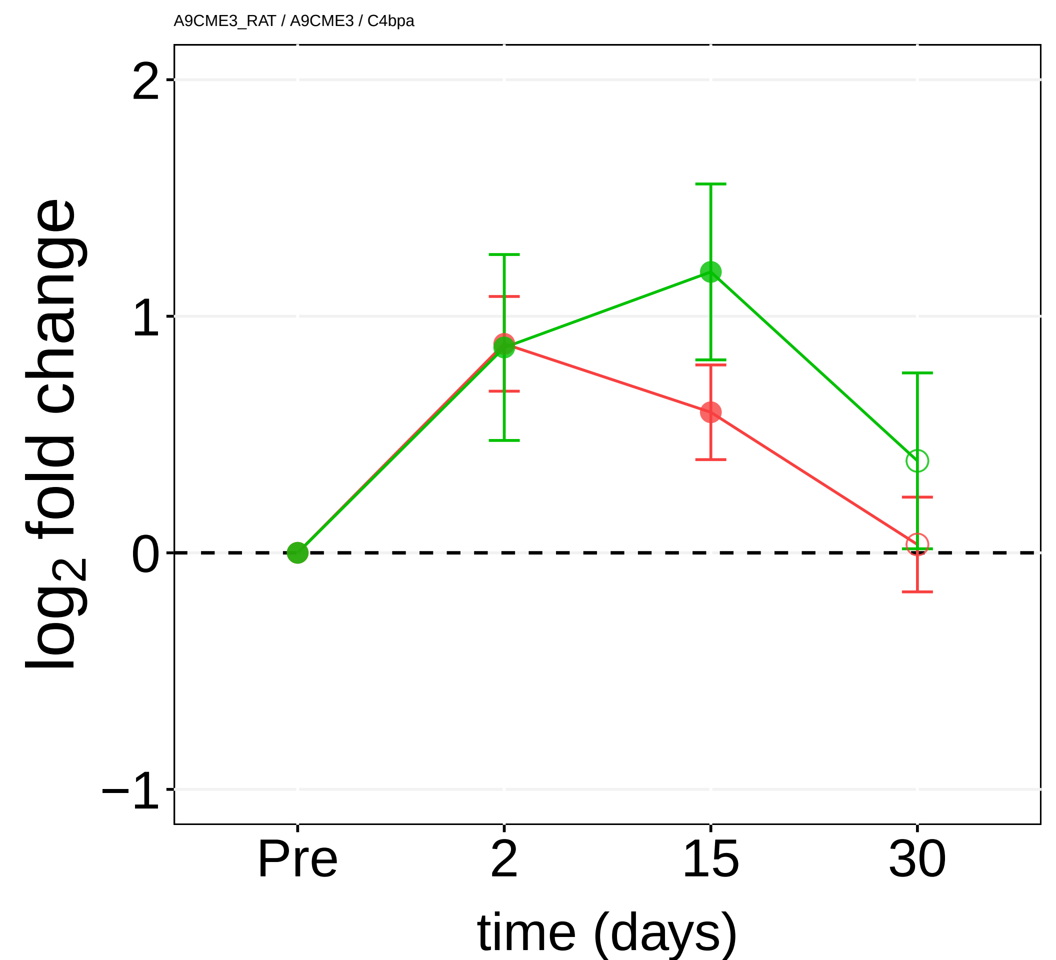

A9CME3_RAT / A9CME3 / C4bpa
2
1
0
−1
log2 fold change
Pre
2
15
30
time (days)

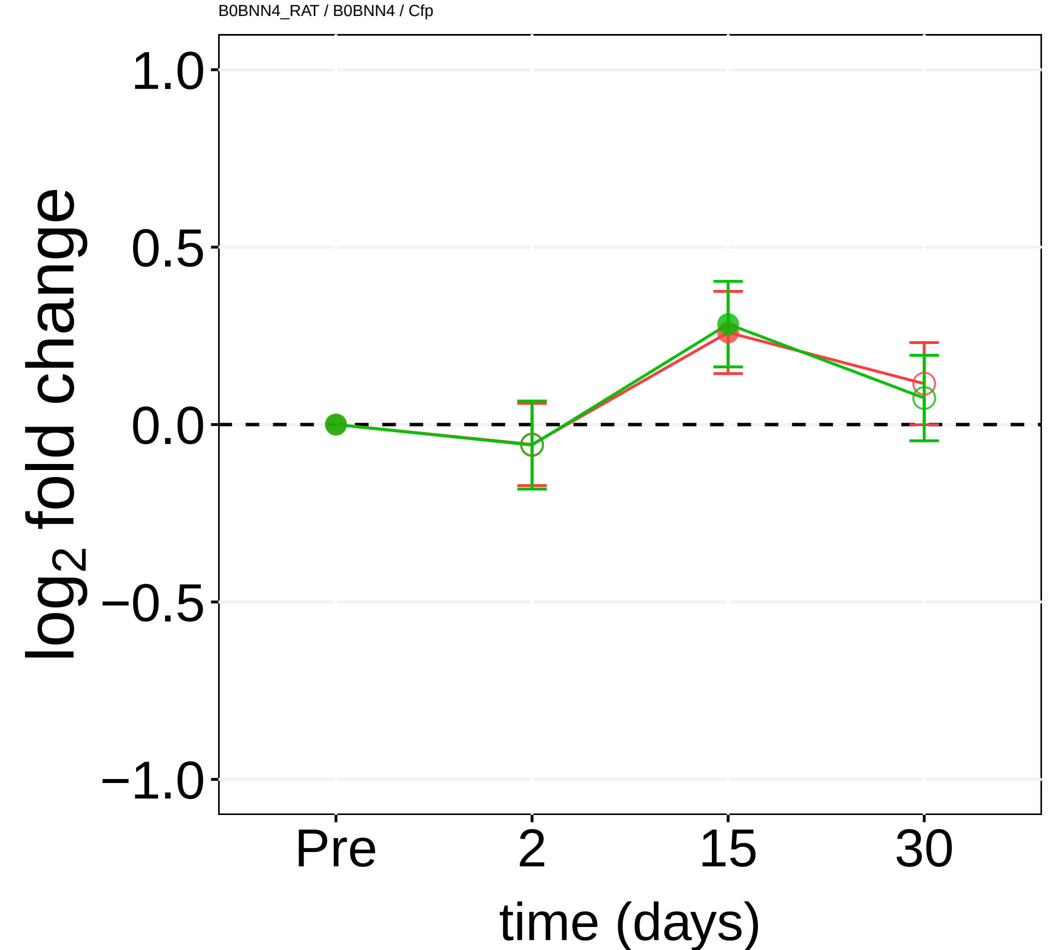
B0BNN4_RAT / B0BNN4 / Cfp
1.0
0.5
0.0
−0.5
−1.0
log2 fold change
Pre
2
15
30
time (days)

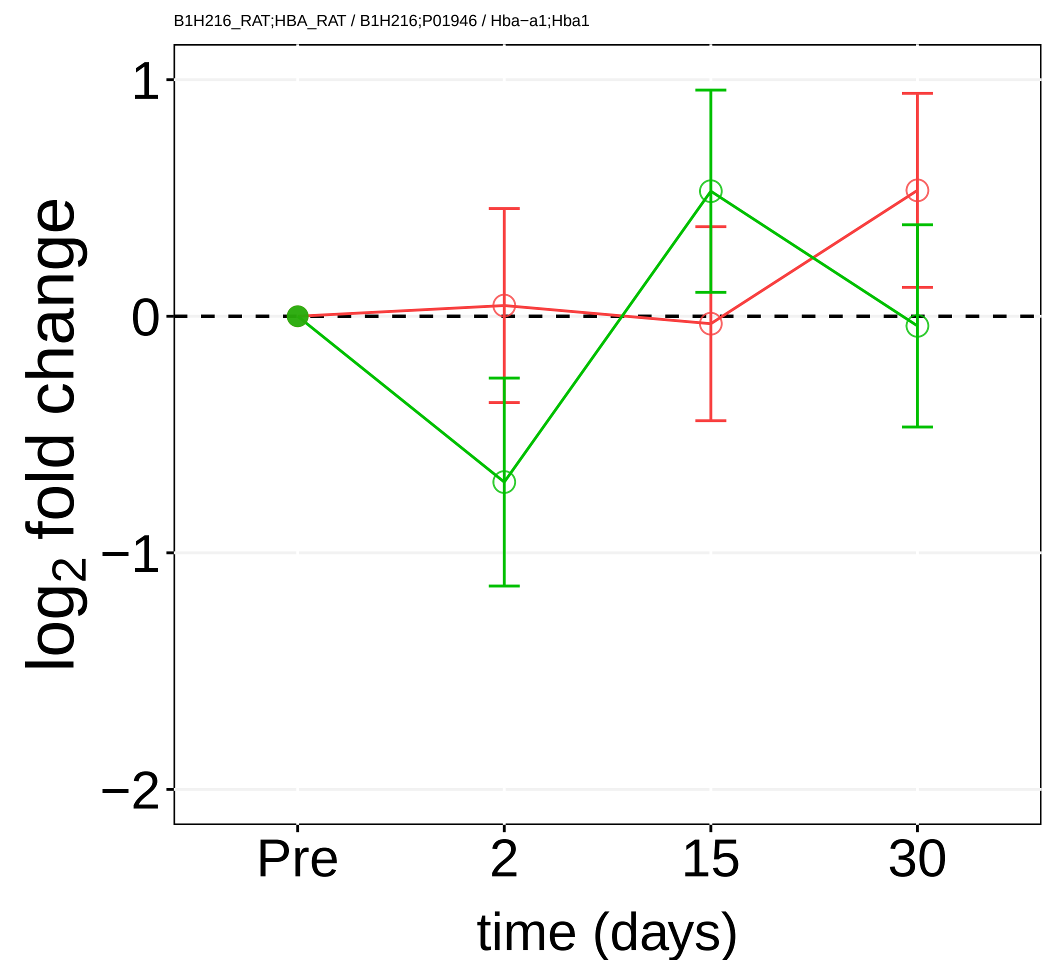

B1H216_RAT;HBA_RAT / B1H216;P01946 / Hba−a1;Hba1
1
0
−1
−2
log2 fold change
Pre
2
15
30
time (days)

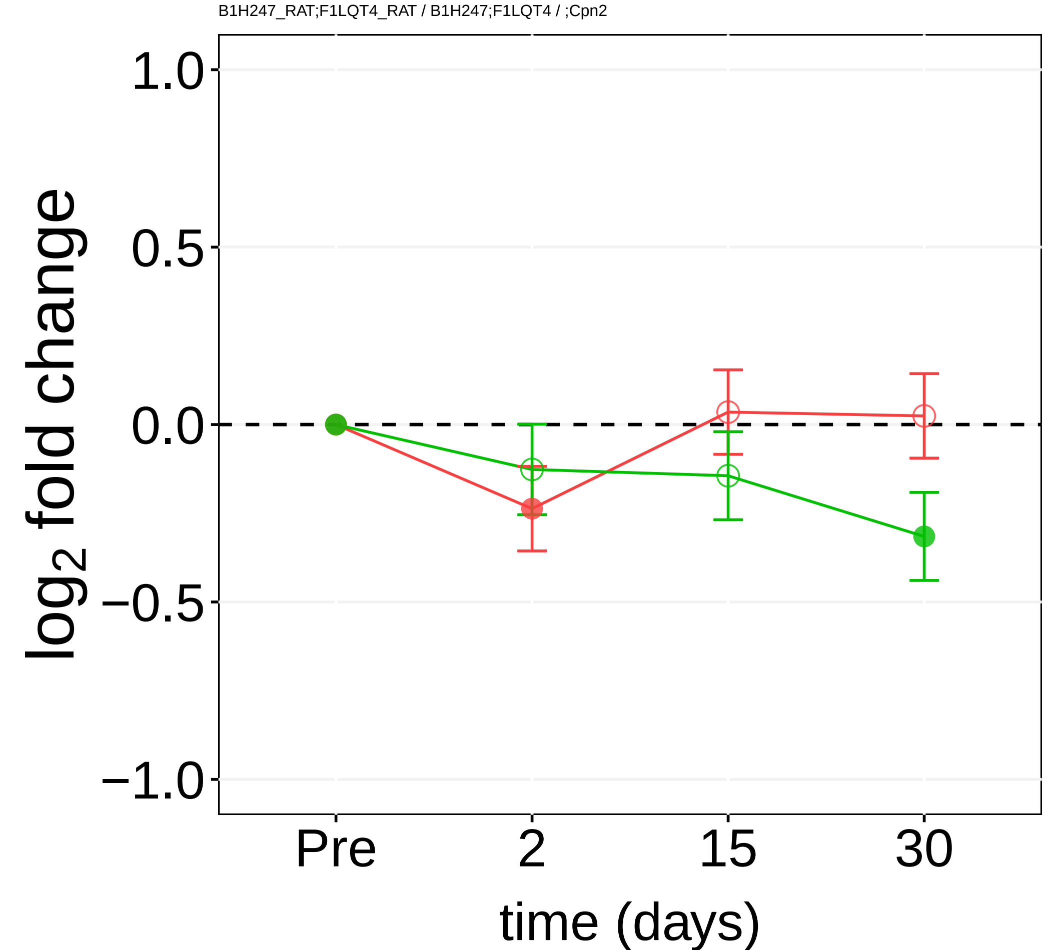

B1H247_RAT;F1LQT4_RAT / B1H247;F1LQT4 / ;Cpn2
1.0
0.5
0.0
−0.5
−1.0
log2 fold change
Pre
2
15
30
time (days)

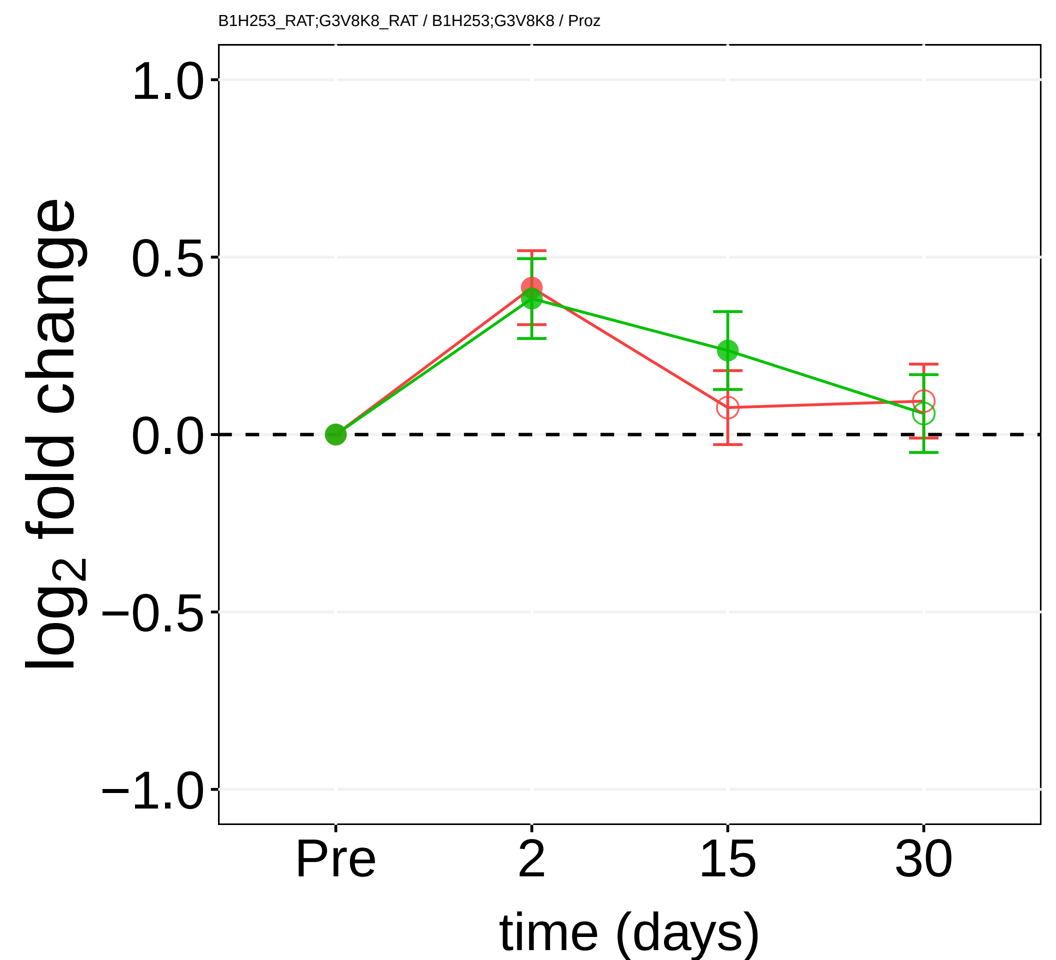
B1H253_RAT;G3V8K8_RAT / B1H253;G3V8K8 / Proz
1.0
0.5
0.0
−0.5
−1.0
log2 fold change
Pre
2
15
30
time (days)

B1H260_RAT / B1H260 / F13b

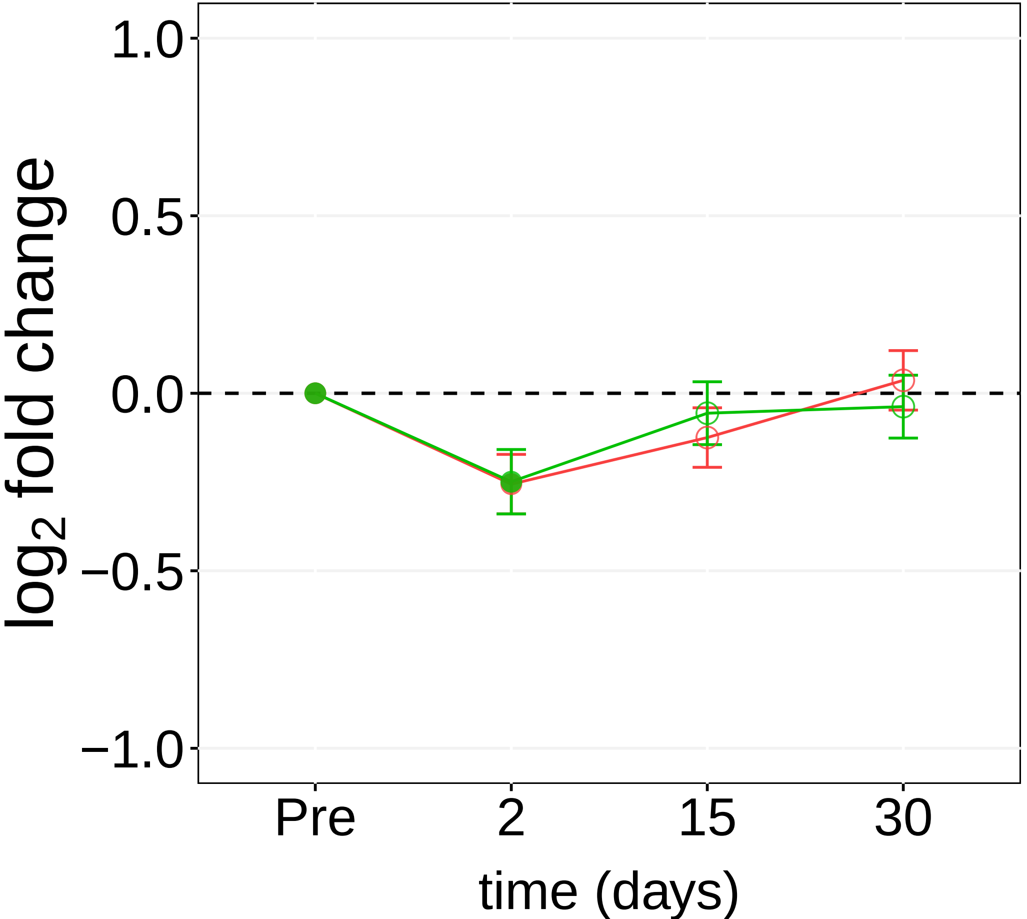

B1WC21_RAT / B1WC21 / Fbln1

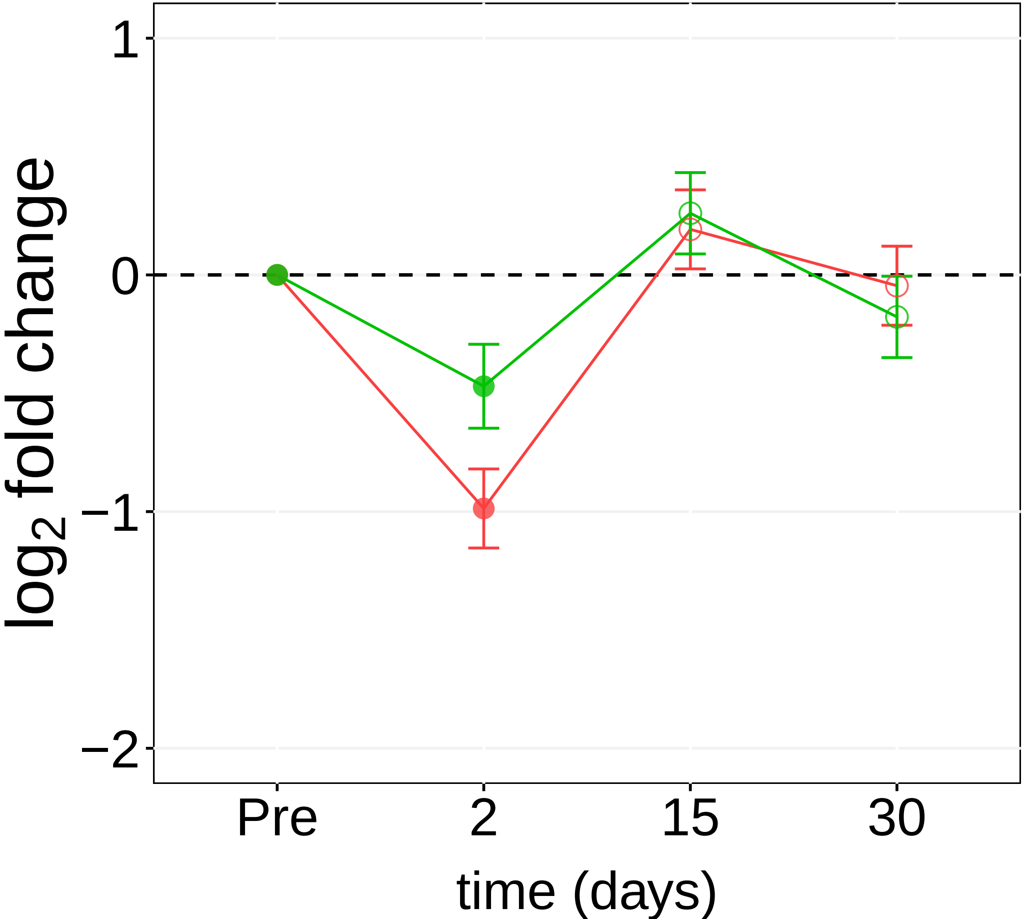

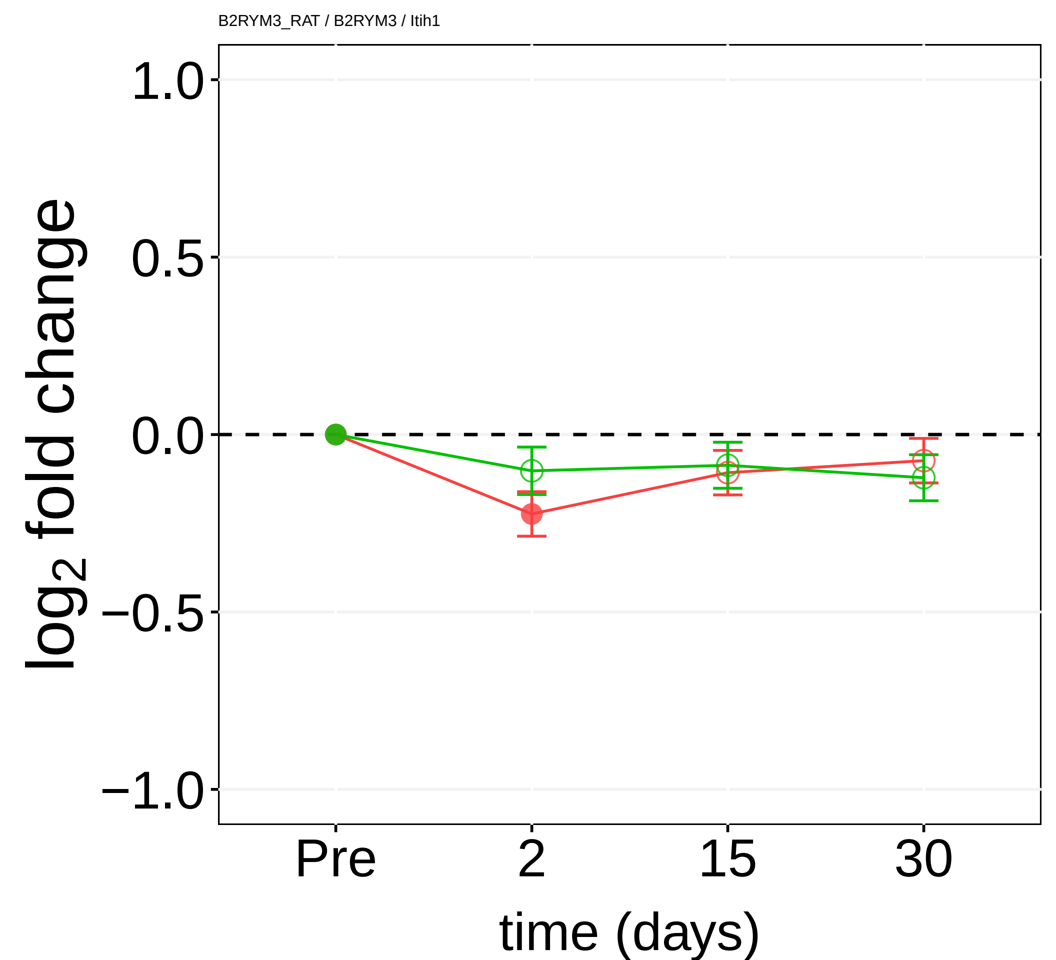

B2RYM3_RAT / B2RYM3 / Itih1
1.0
0.5
0.0
−0.5
−1.0
log2 fold change
Pre
2
15
30
time (days)

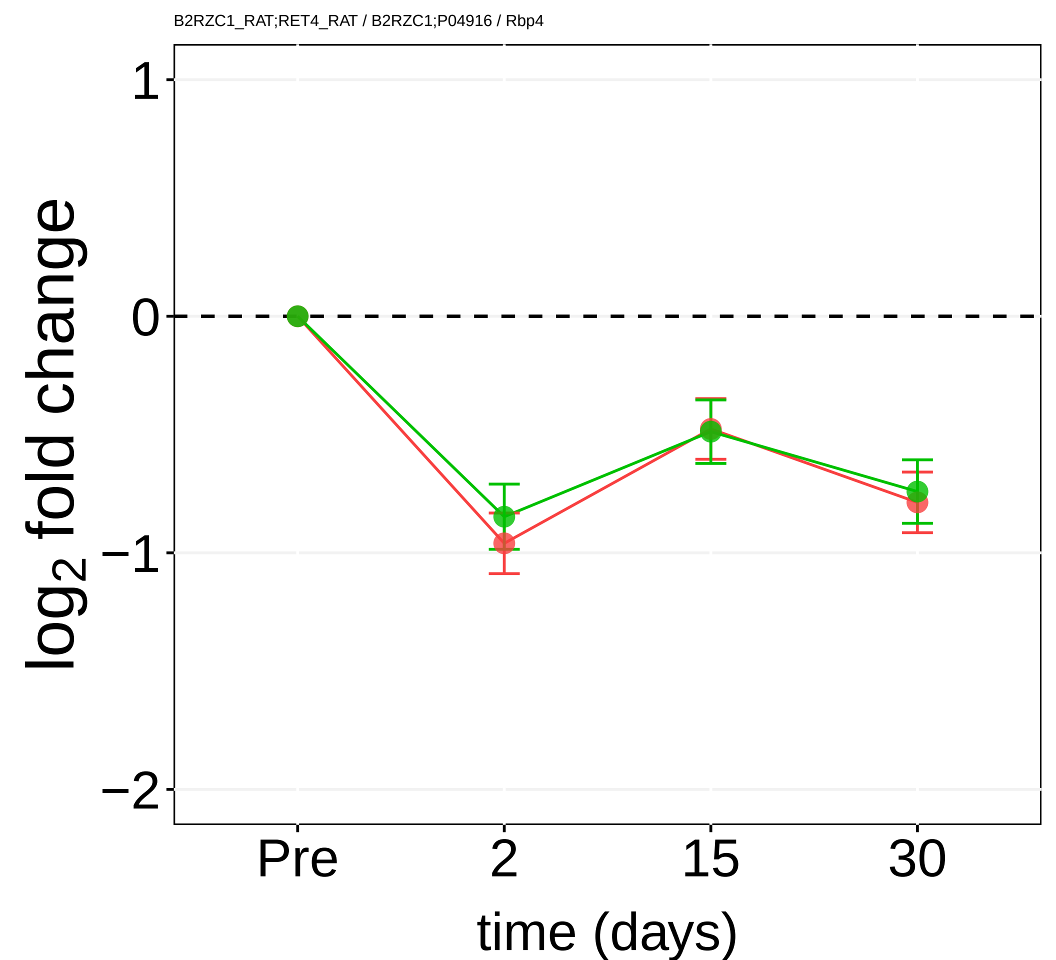
B2RZC1_RAT;RET4_RAT / B2RZC1;P04916 / Rbp4
log2 fold change
1
0
−1
−2
Pre
2
15
30
time (days)

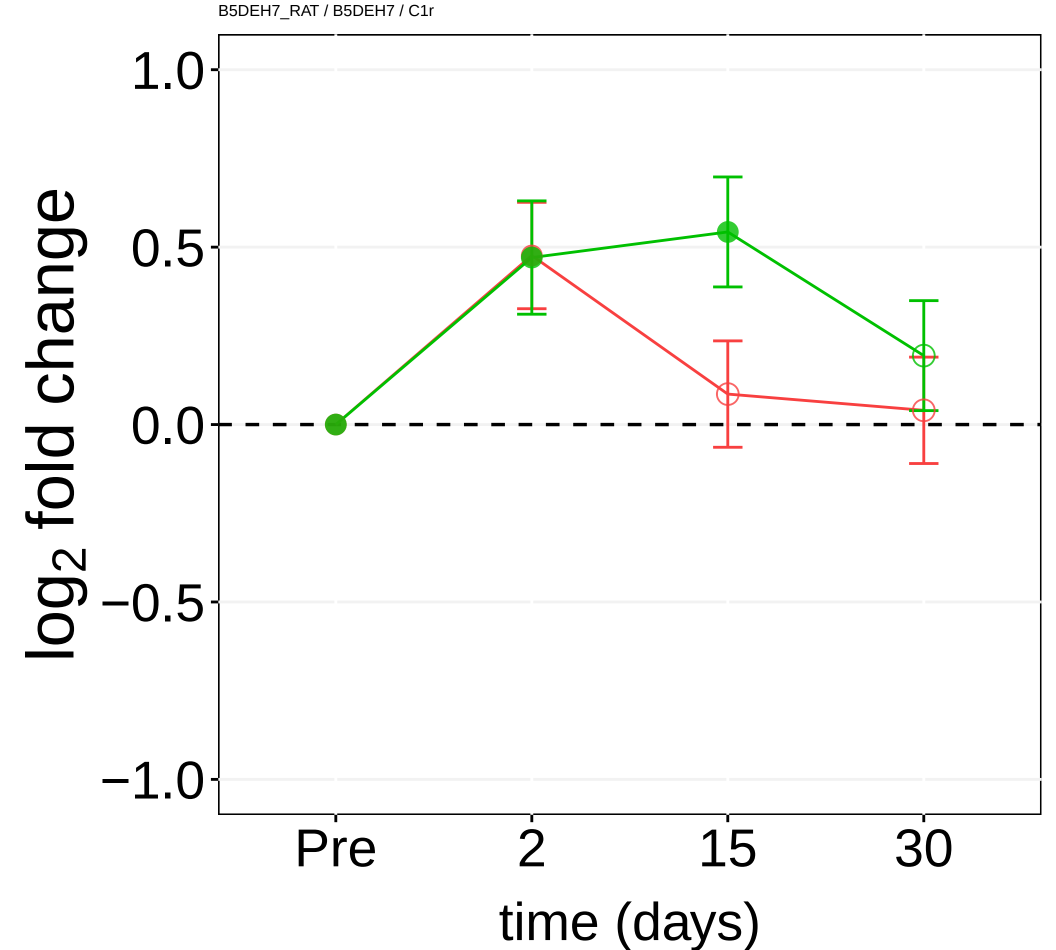

B5DEH7_RAT / B5DEH7 / C1r
1.0
0.5
0.0
−0.5
−1.0
log2 fold change
Pre
2
15
30
time (days)

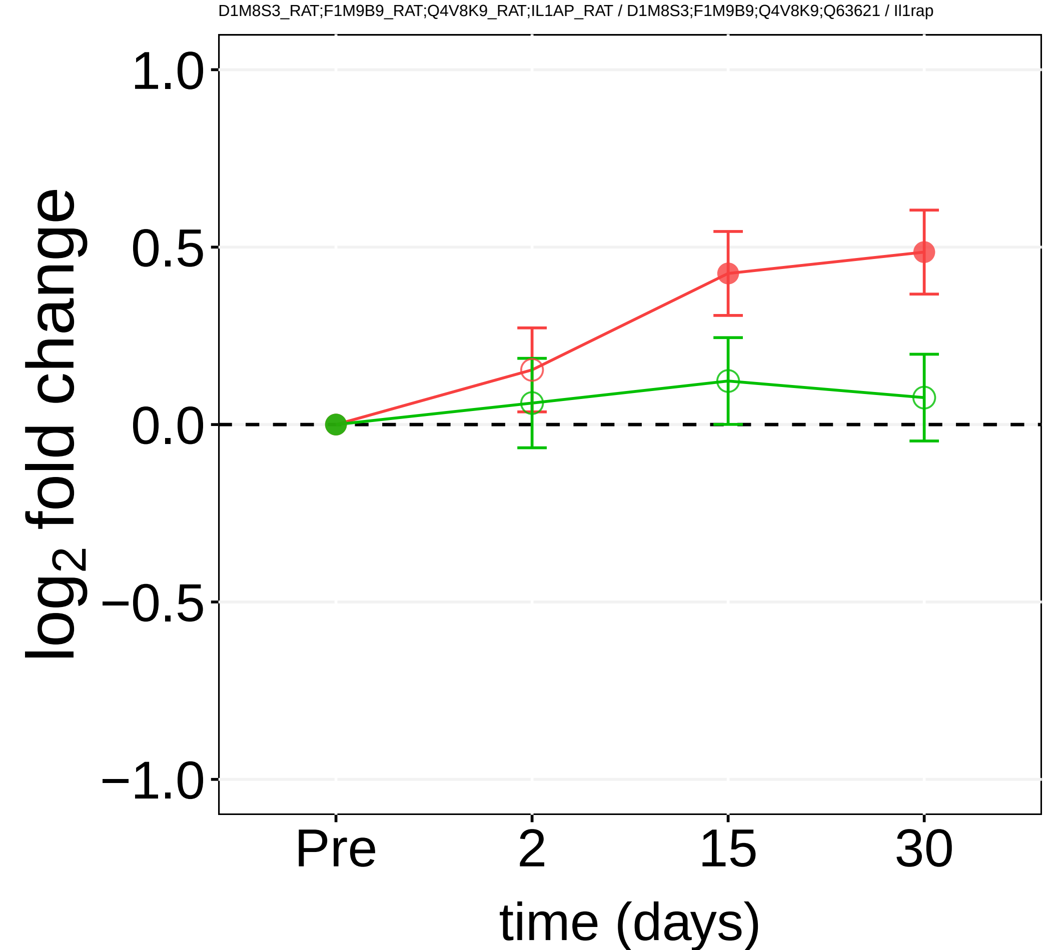
D1M8S3_RAT;F1M9B9_RAT;Q4V8K9_RAT;IL1AP_RAT / D1M8S3;F1M9B9;Q4V8K9;Q63621 / Il1rap
1.0
0.5
0.0
−0.5
−1.0
log2 fold change
Pre
2
15
30
time (days)

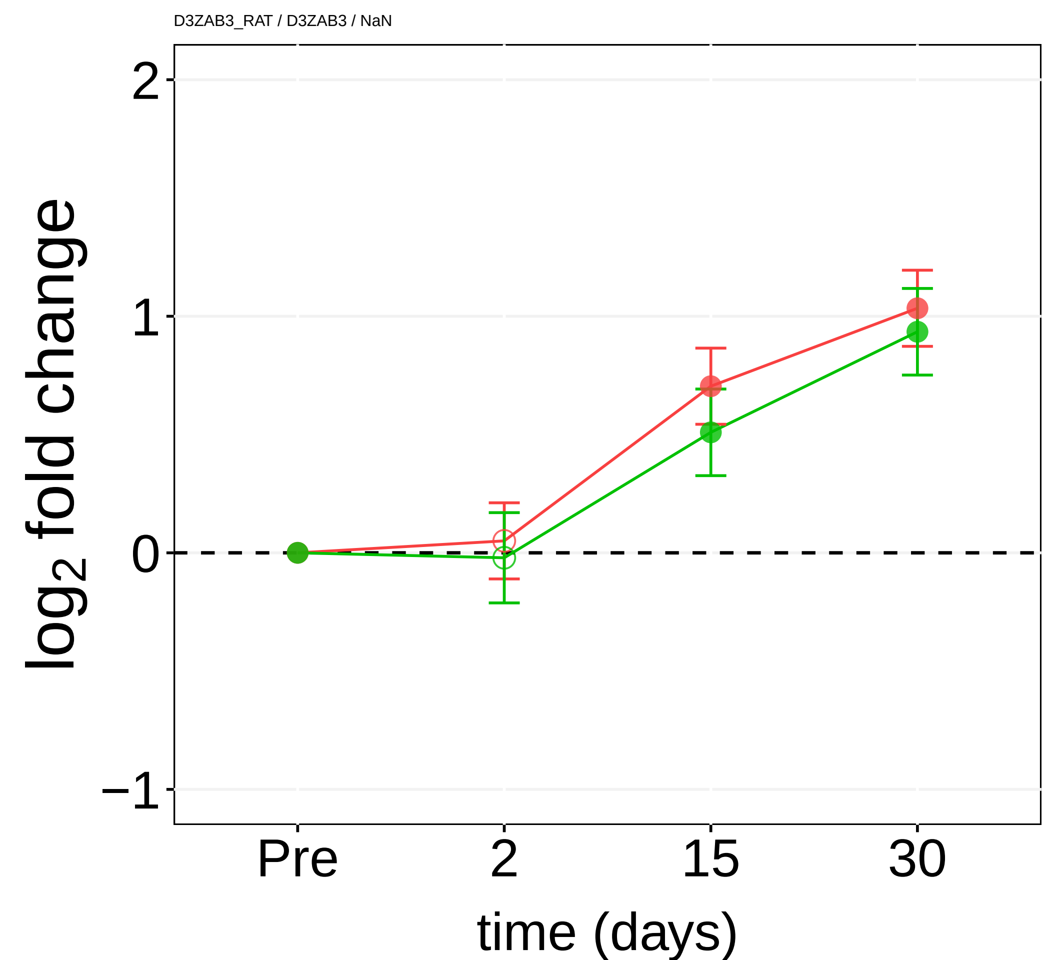

D3ZAB3_RAT / D3ZAB3 / NaN
log2 fold change
2
1
0
−1
Pre
2
15
30
time (days)

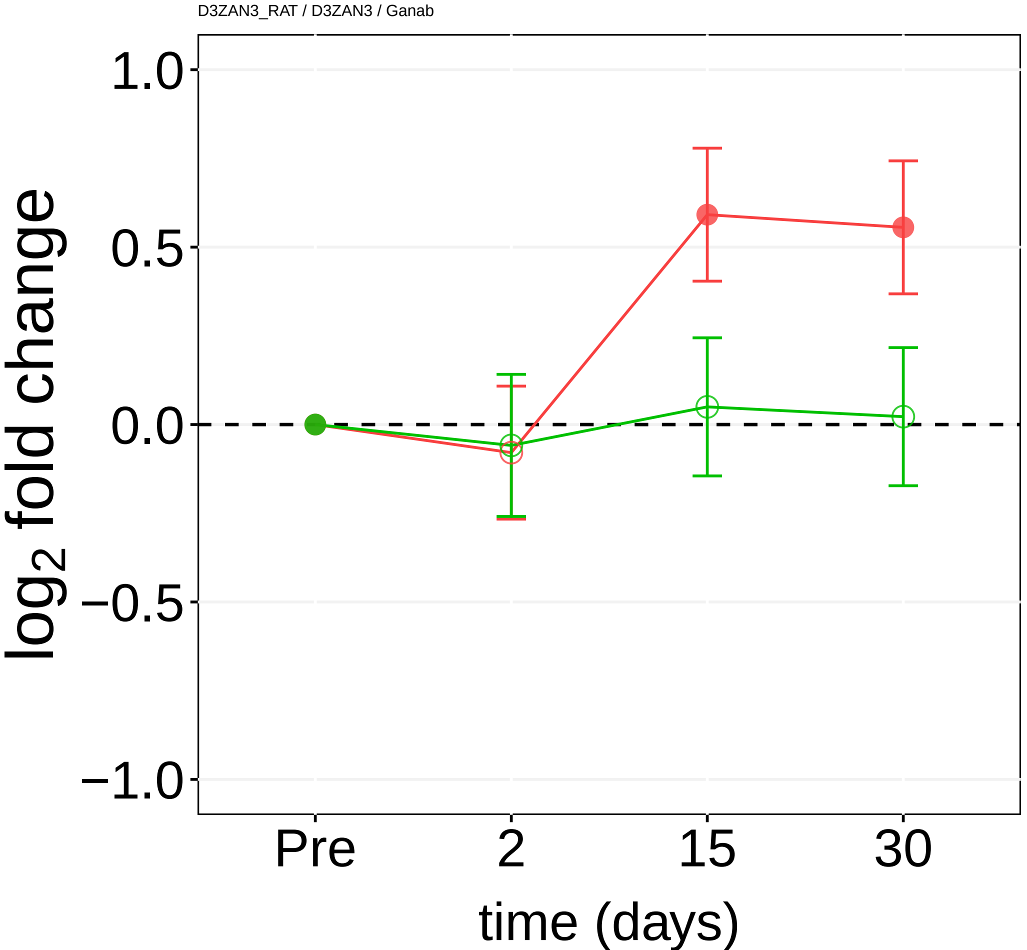

D3ZAN3_RAT / D3ZAN3 / Ganab
1.0
0.5
0.0
−0.5
−1.0
log2 fold change
Pre
2
15
30
time (days)

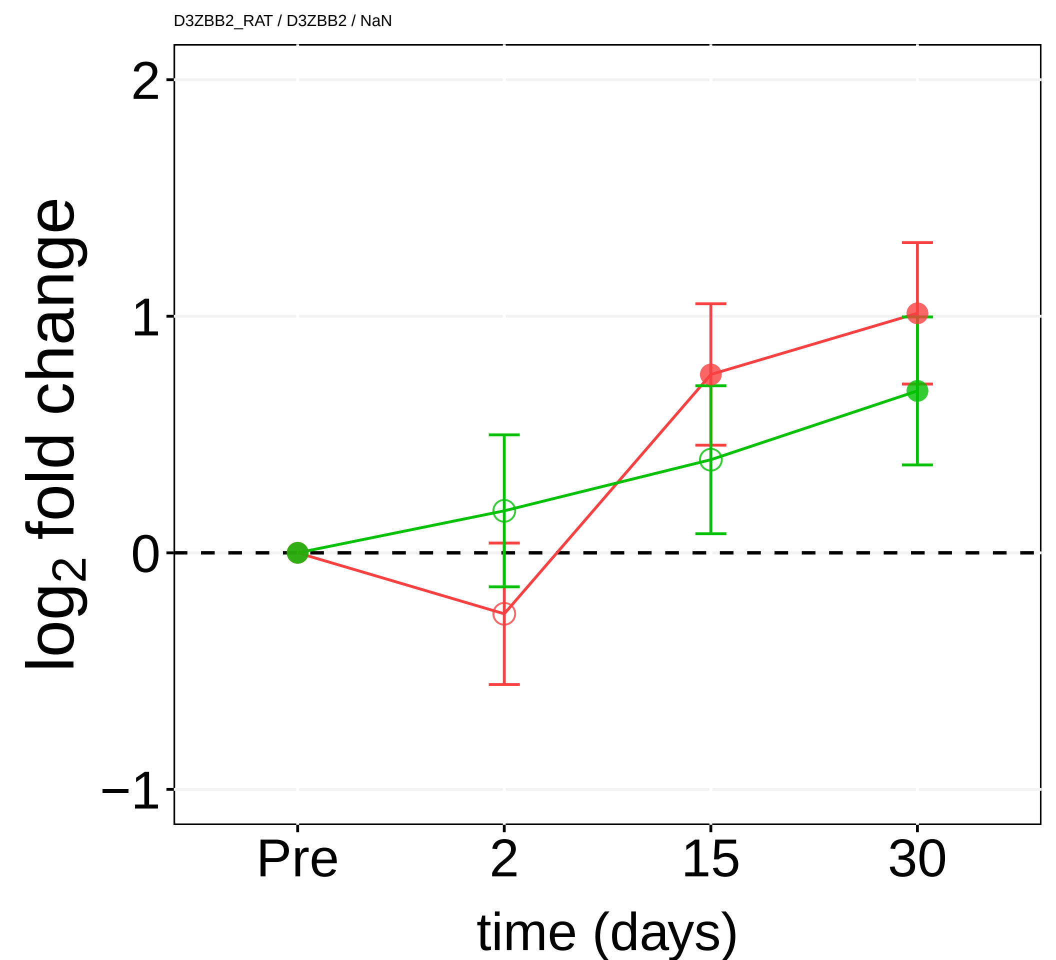
D3ZBB2_RAT / D3ZBB2 / NaN
log2 fold change
2
1
0
−1
Pre
2
15
30
time (days)

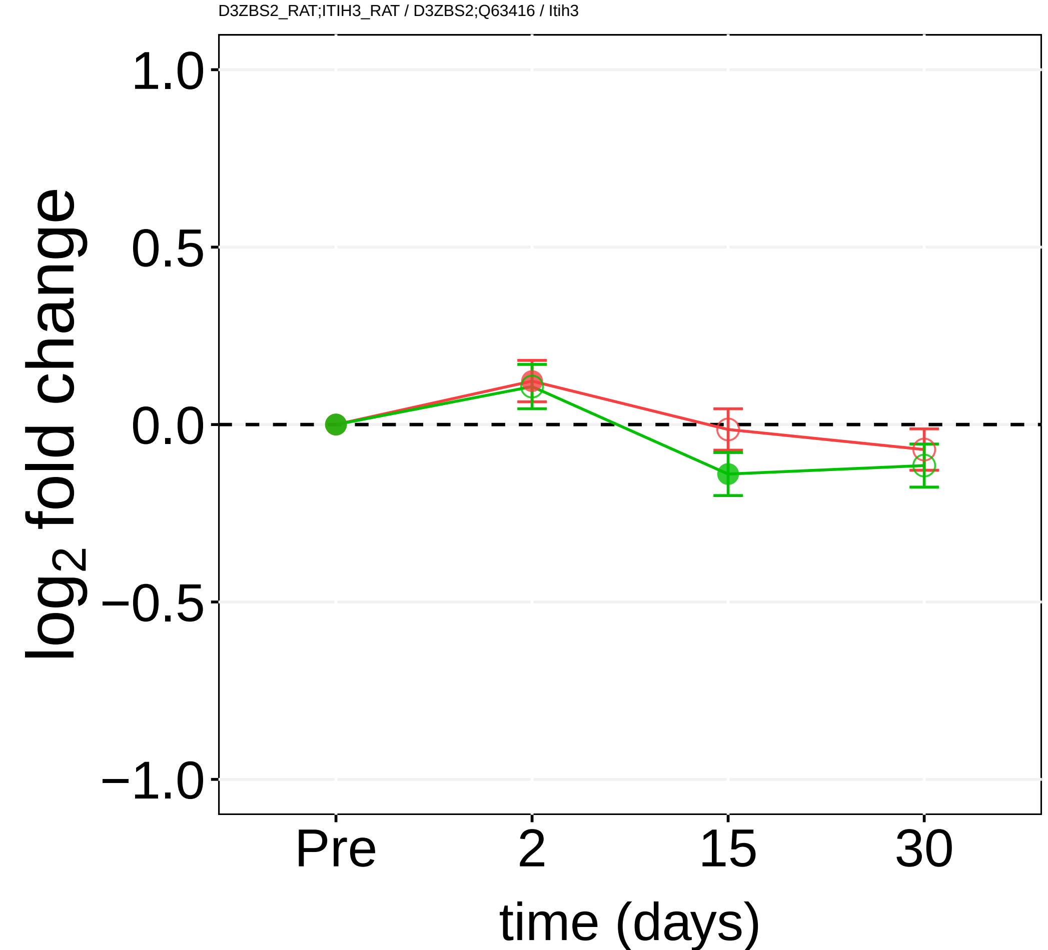

D3ZBS2_RAT;ITIH3_RAT / D3ZBS2;Q63416 / Itih3
1.0
0.5
0.0
−0.5
−1.0
$\log_2$ fold change
Pre
2
15
30
time (days)

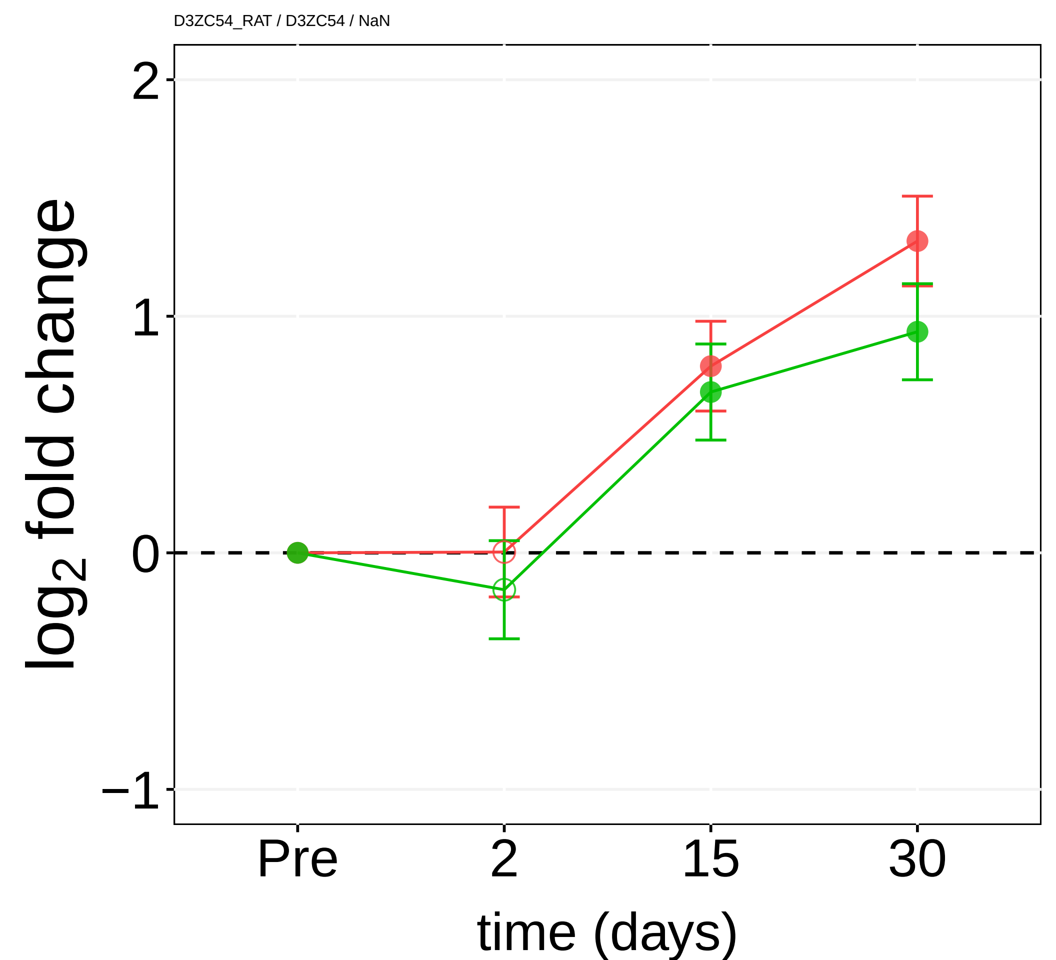

D3ZC54_RAT / D3ZC54 / NaN
2
1
0
−1
log2 fold change
Pre
2
15
30
time (days)

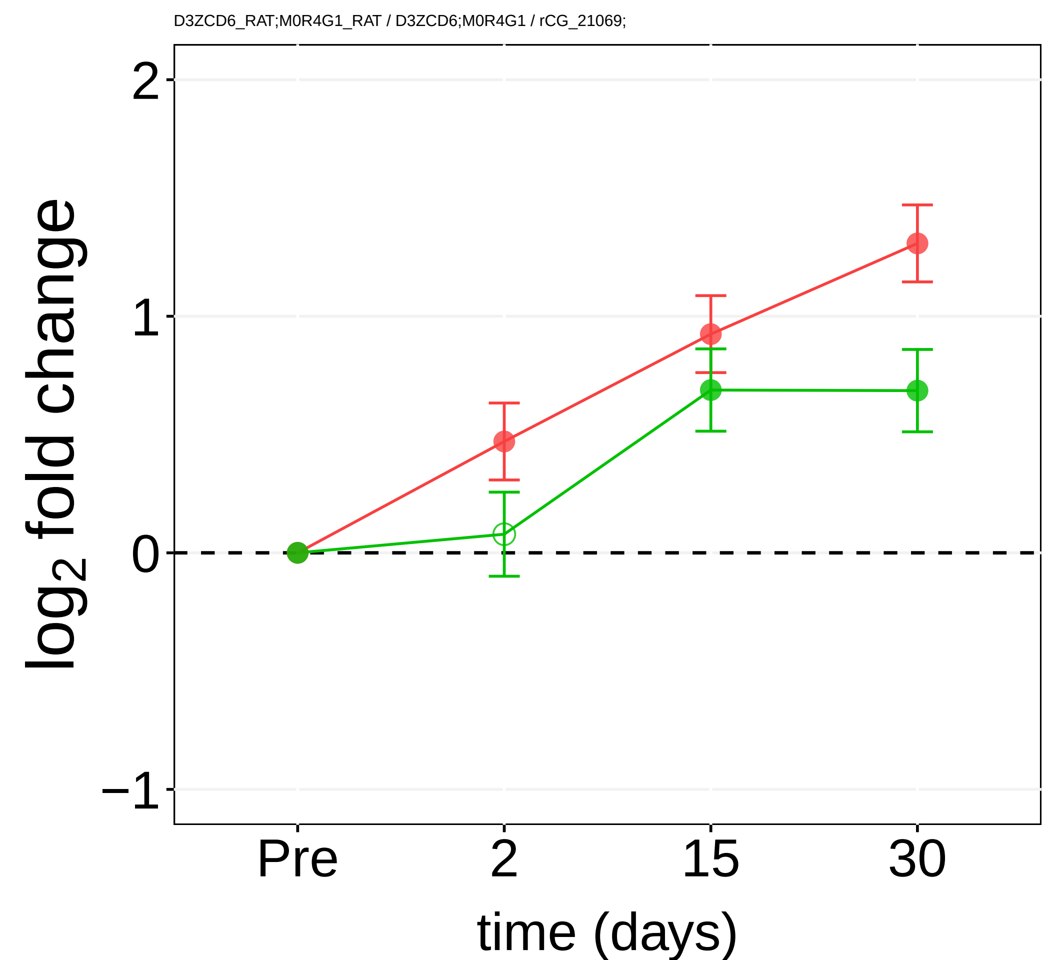
D3ZCD6_RAT;M0R4G1_RAT / D3ZCD6;M0R4G1 / rCG_21069;
log2 fold change
2
1
0
−1
Pre
2
15
30
time (days)

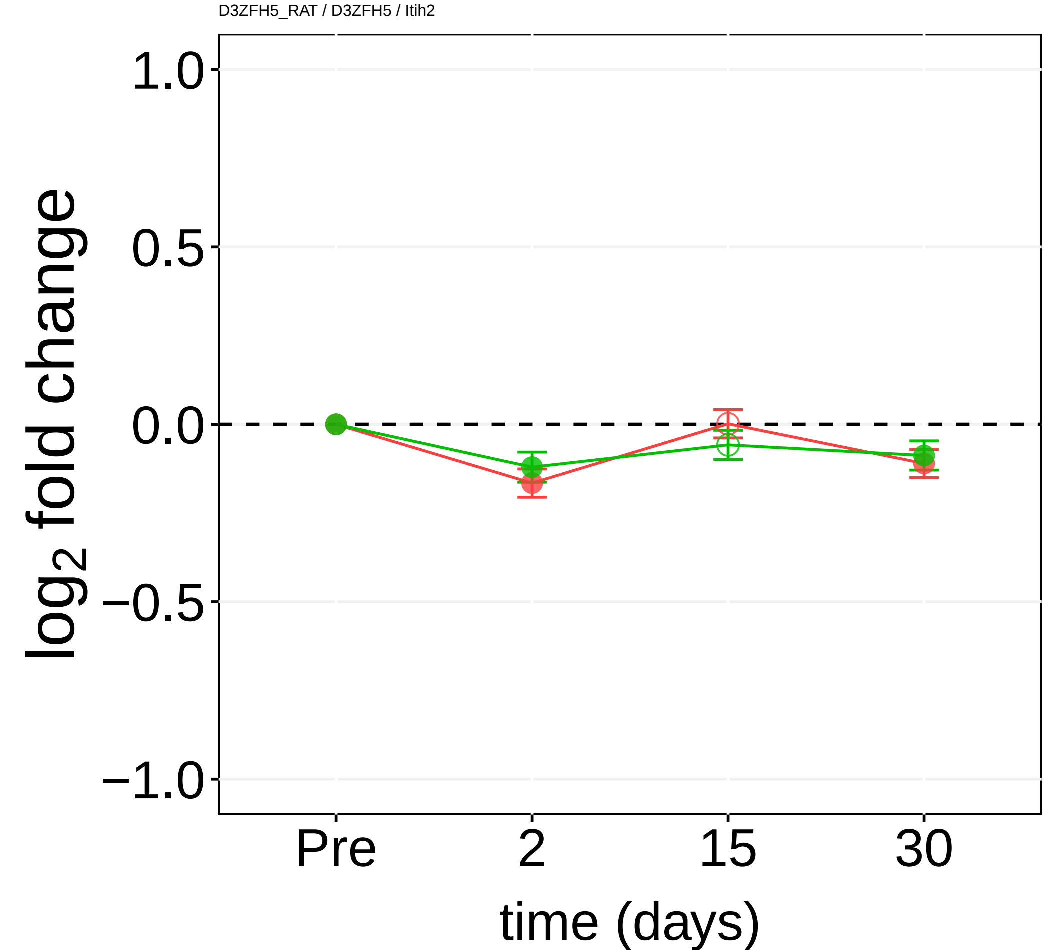
D3ZFH5_RAT / D3ZFH5 / Itih2
1.0
0.5
0.0
−0.5
−1.0
log2 fold change
Pre
2
15
30
time (days)

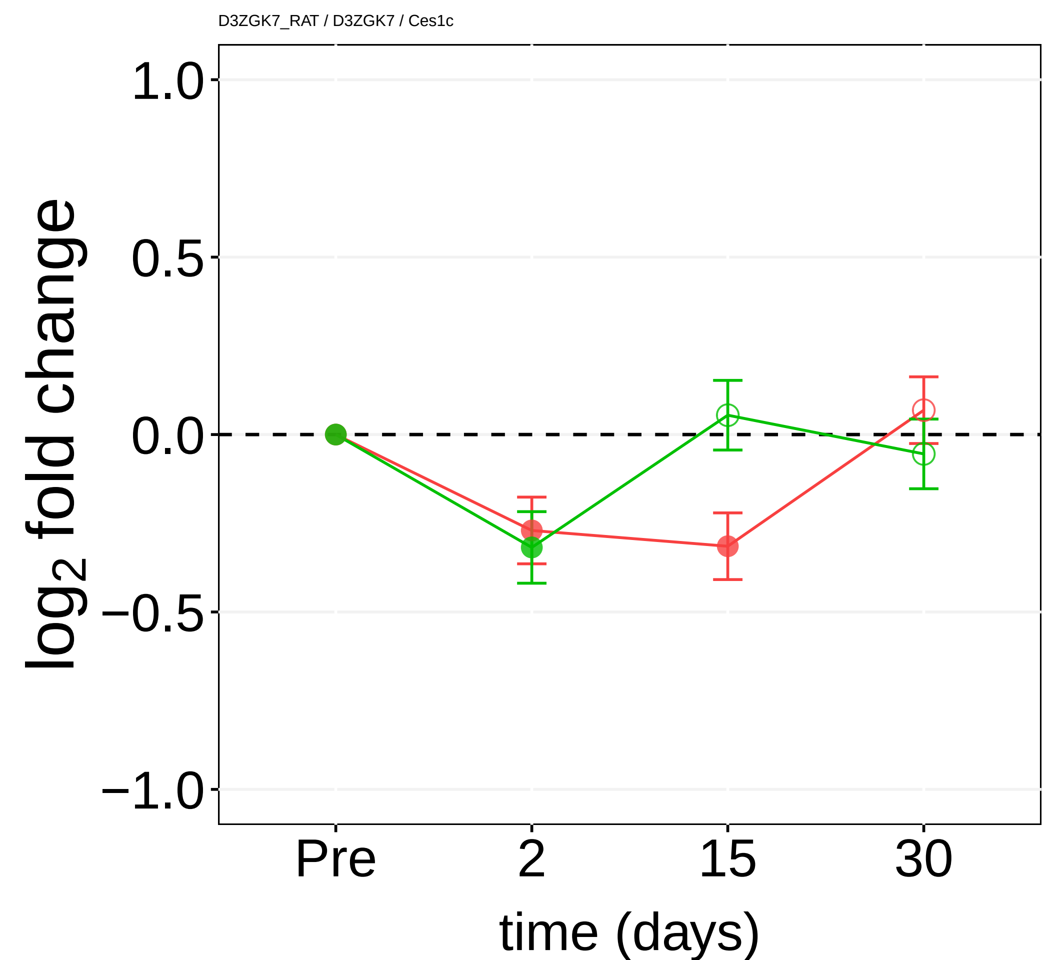
D3ZGK7_RAT / D3ZGK7 / Ces1c
log2 fold change
1.0
0.5
0.0
−0.5
−1.0
Pre
2
15
30
time (days)

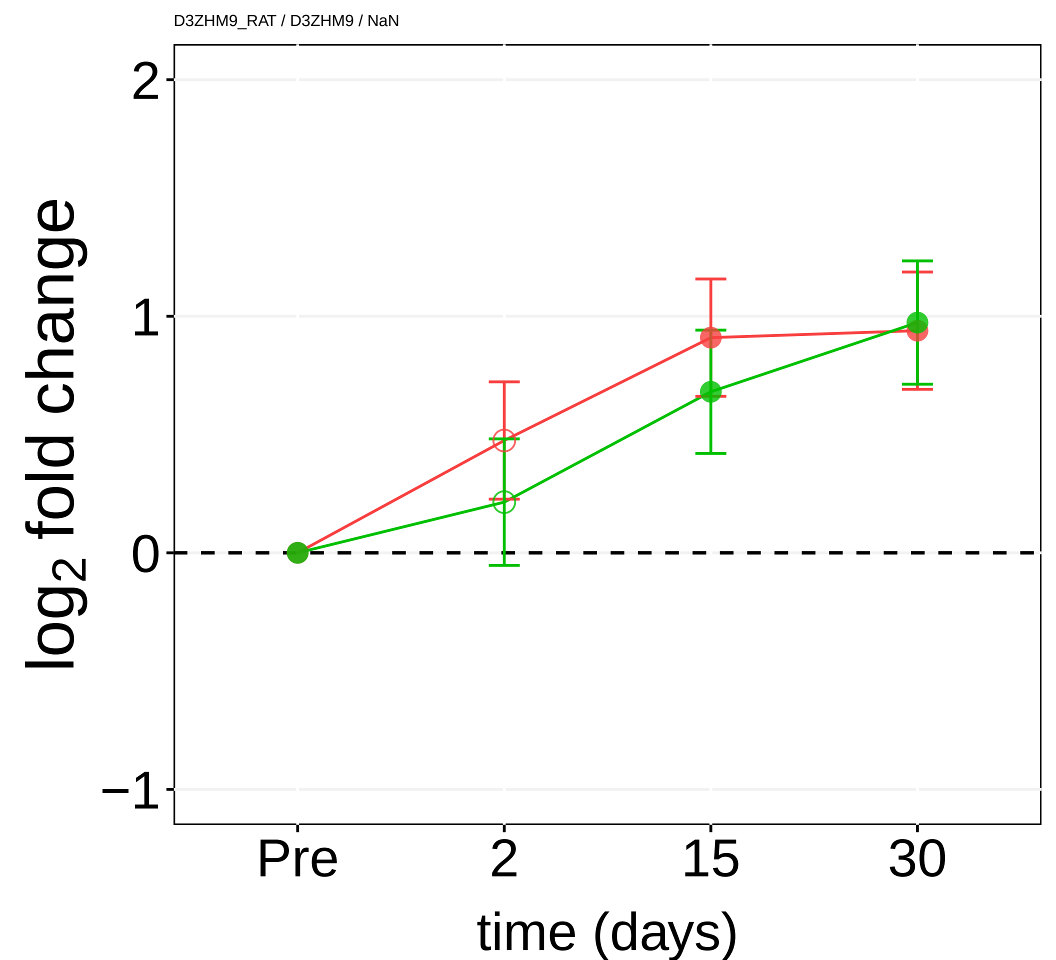

D3ZHM9_RAT / D3ZHM9 / NaN
2
1
0
−1
log2 fold change
Pre
2
15
30
time (days)

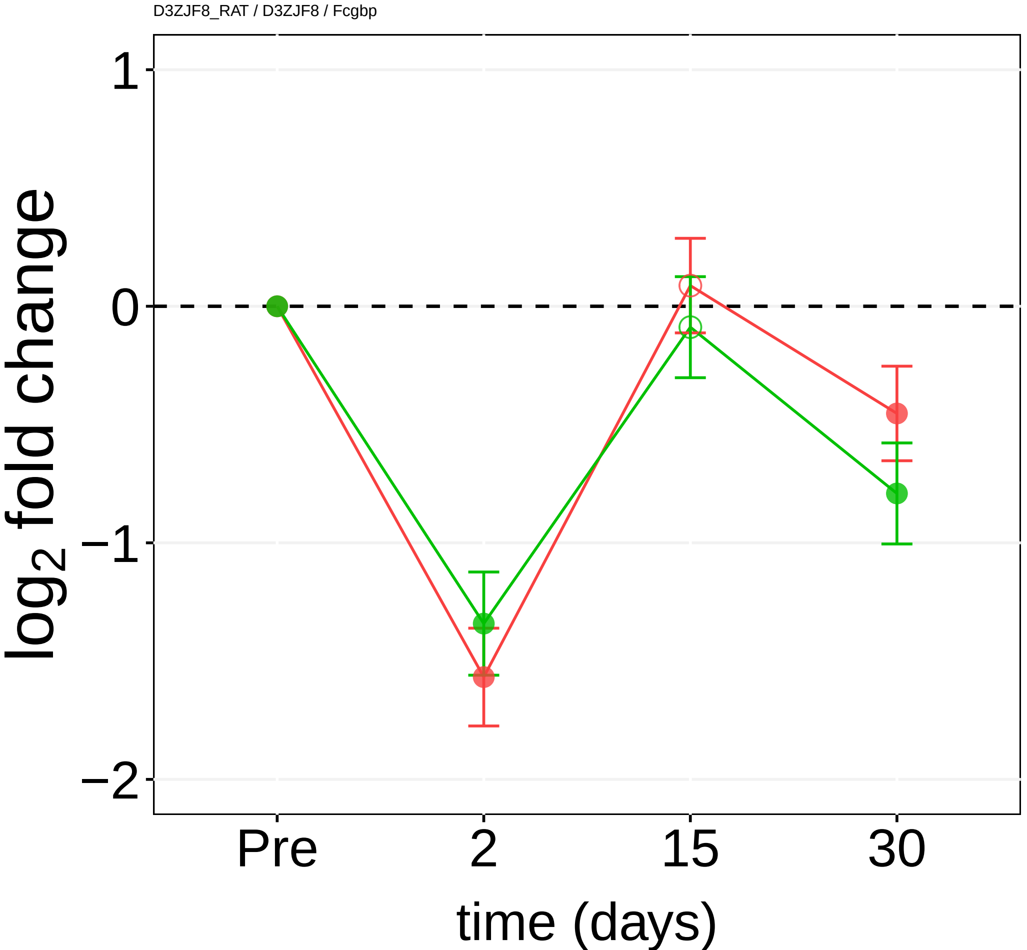

D3ZJF8_RAT / D3ZJF8 / Fcgbp
1
0
−1
−2
log2 fold change
Pre
2
15
30
time (days)

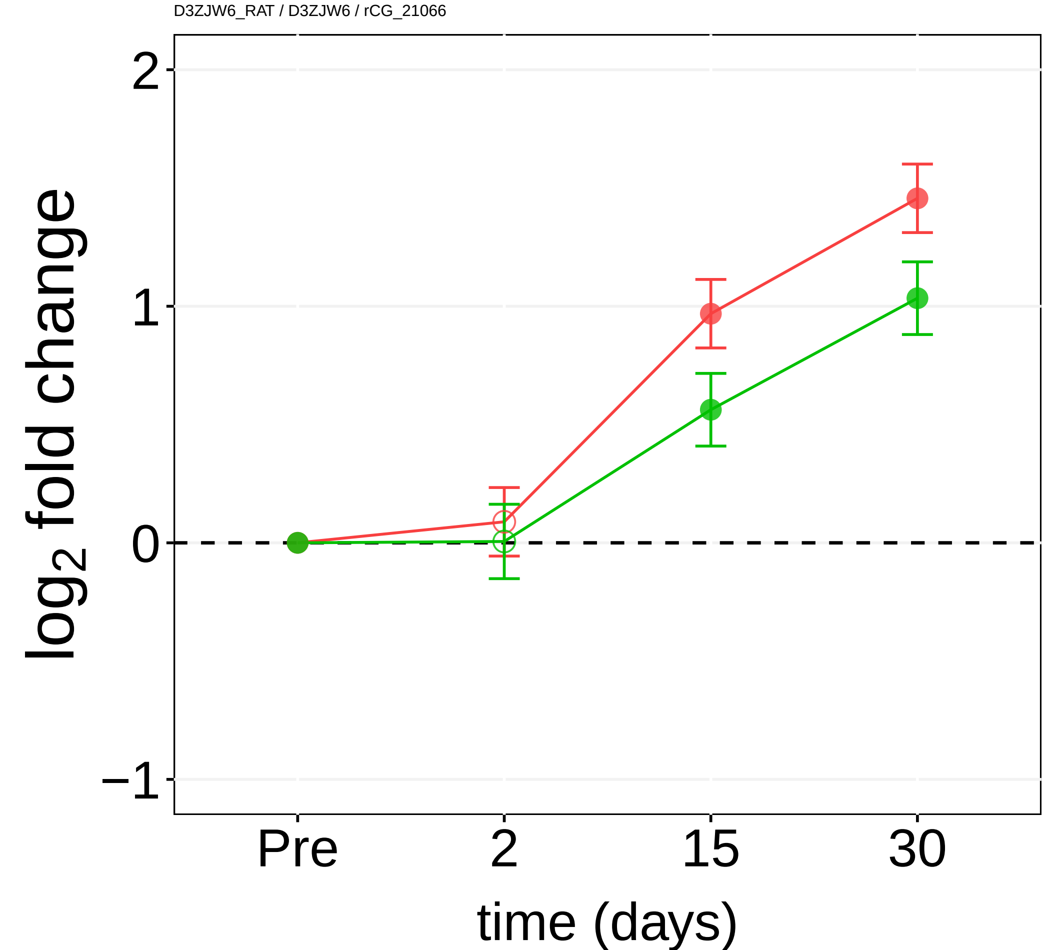

D3ZJW6_RAT / D3ZJW6 / rCG_21066
log2 fold change
2
1
0
−1
Pre
2
15
30
time (days)

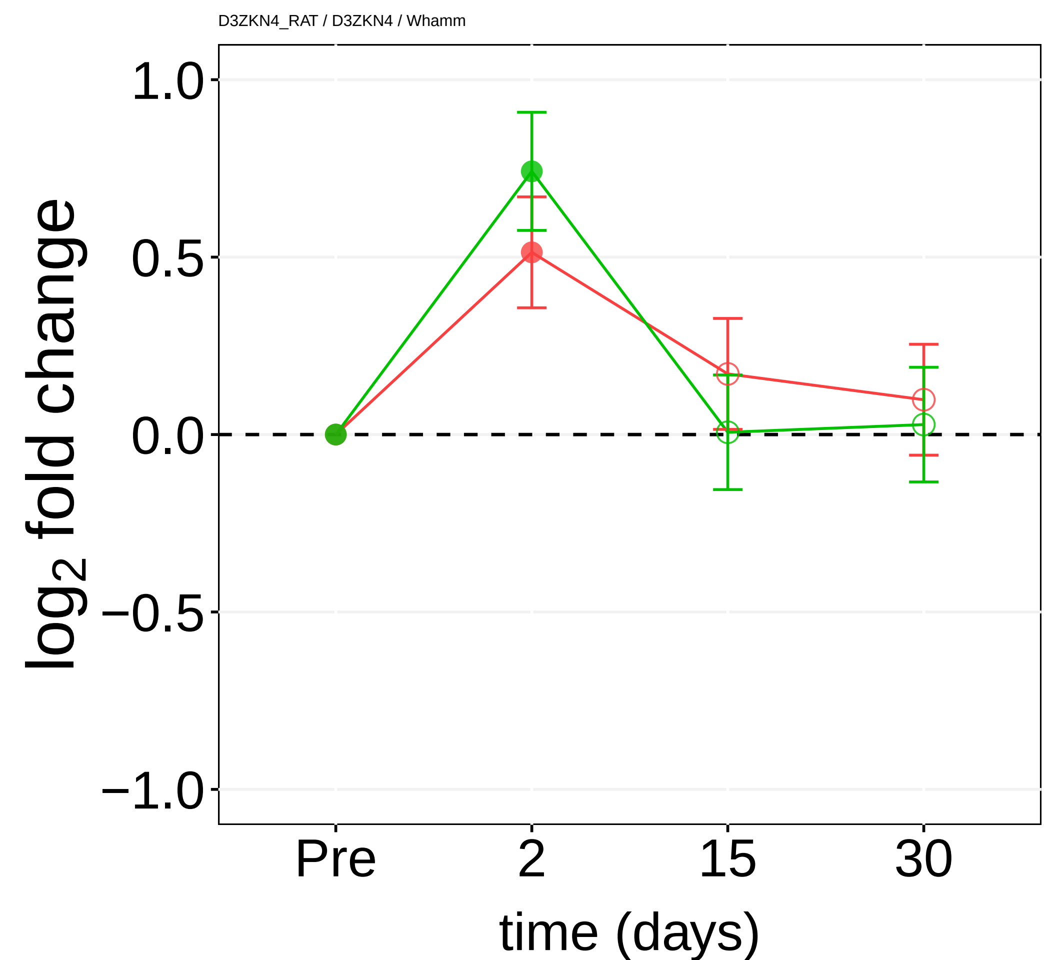
D3ZKN4_RAT / D3ZKN4 / Whamm
1.0
0.5
0.0
−0.5
−1.0
log2 fold change
Pre
2
15
30
time (days)

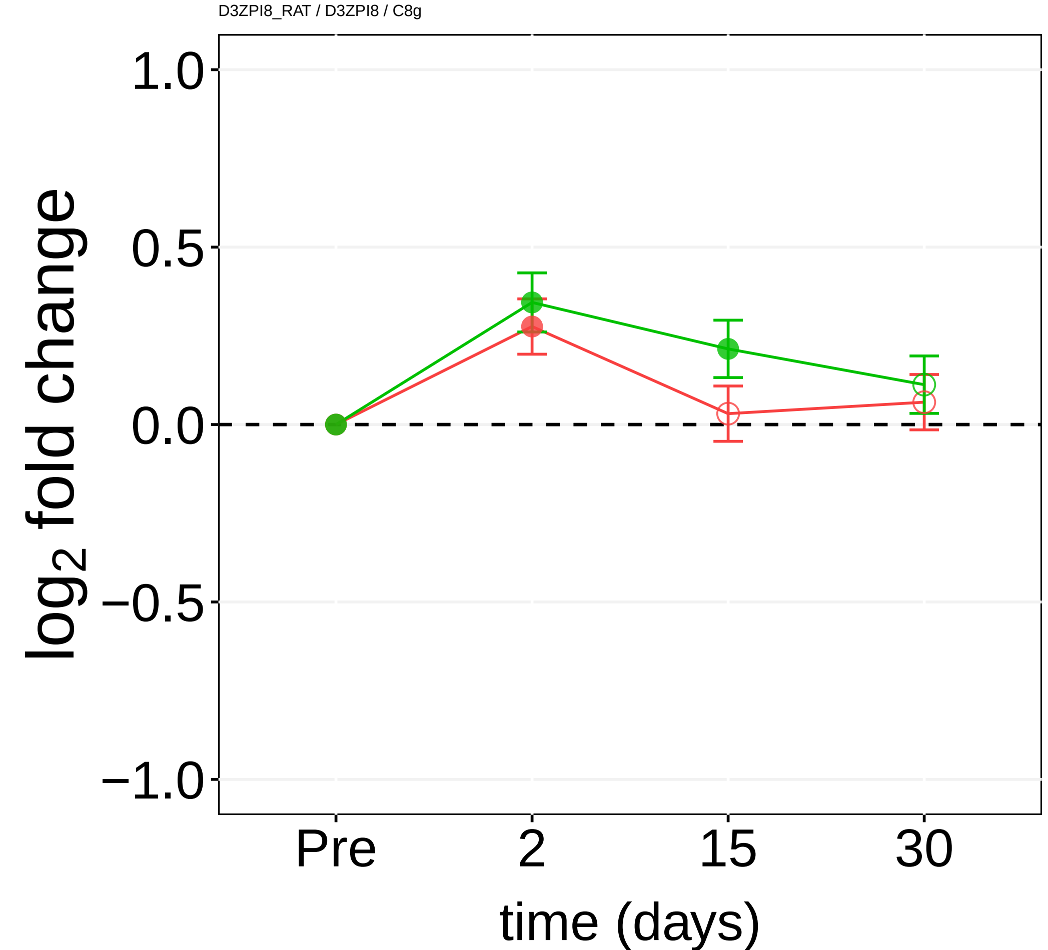

D3ZPI8_RAT / D3ZPI8 / C8g
1.0
0.5
0.0
−0.5
−1.0
log2 fold change
Pre
2
15
30
time (days)

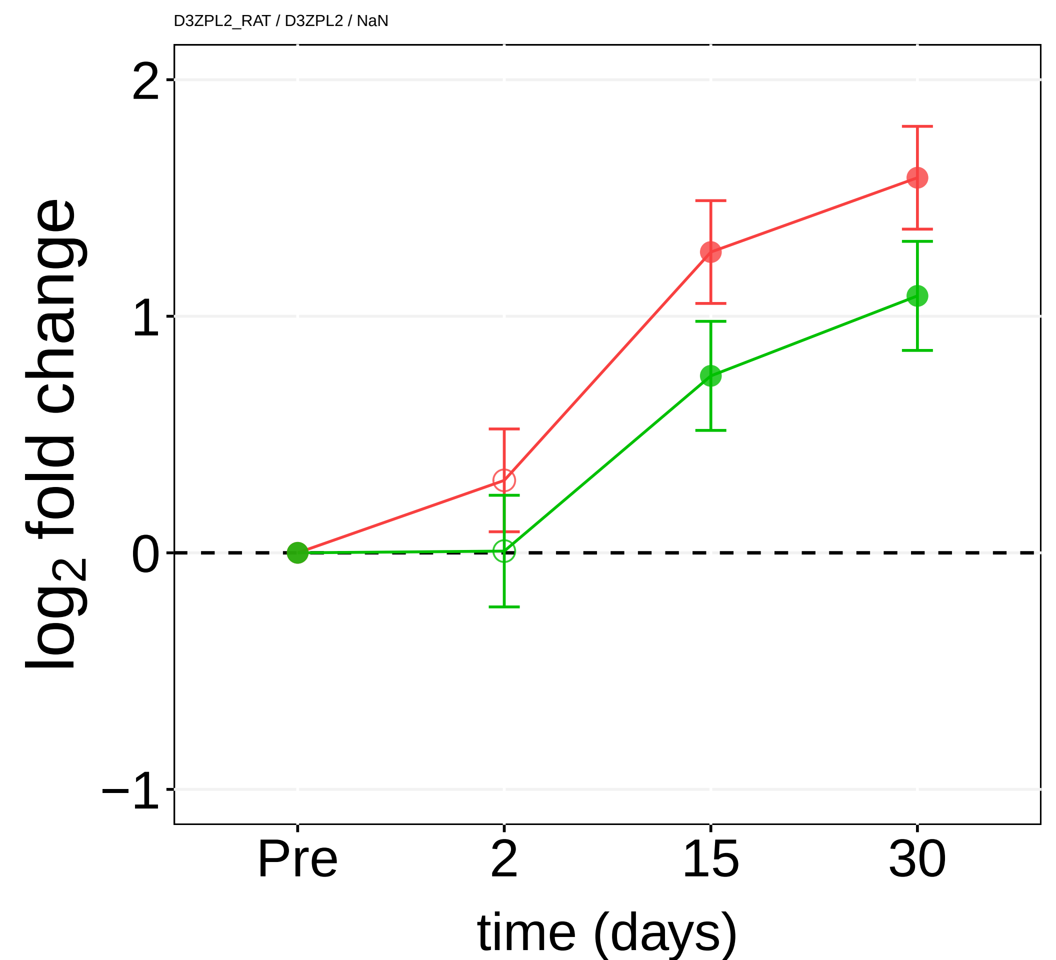
D3ZPL2_RAT / D3ZPL2 / NaN
log2 fold change
2
1
0
−1
Pre
2
15
30
time (days)

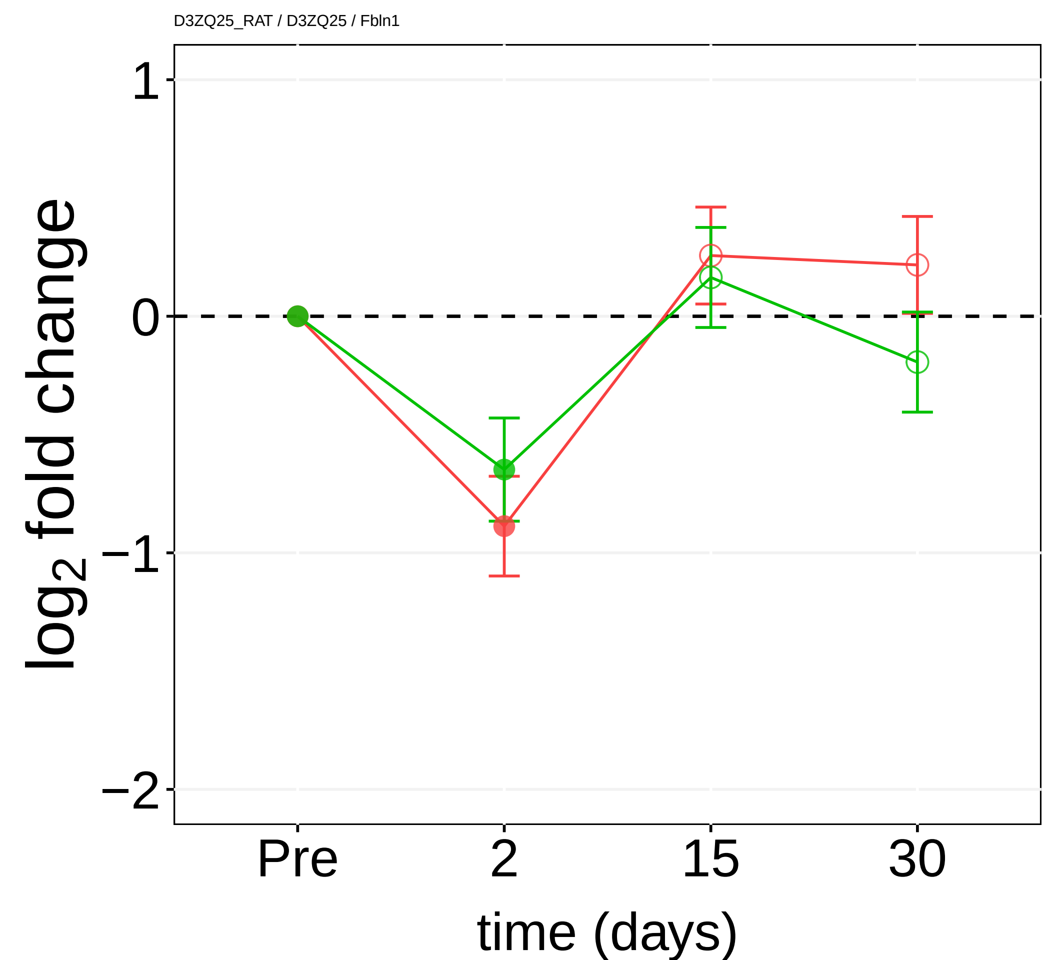
D3ZQ25_RAT / D3ZQ25 / Fbln1
1
0
−1
−2
log2 fold change
Pre
2
15
30
time (days)

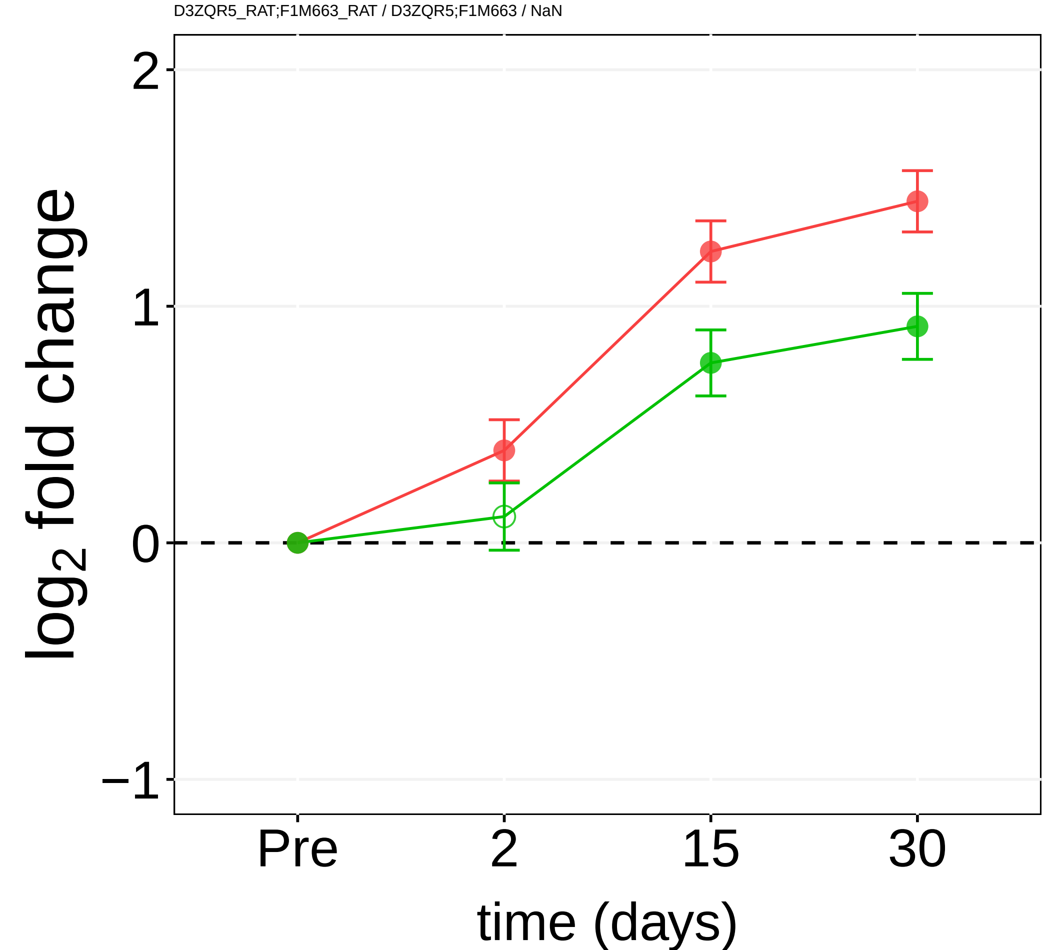
D3ZQR5_RAT;F1M663_RAT / D3ZQR5;F1M663 / NaN
2
1
0
−1
Pre
2
15
30
time (days)
log2 fold change

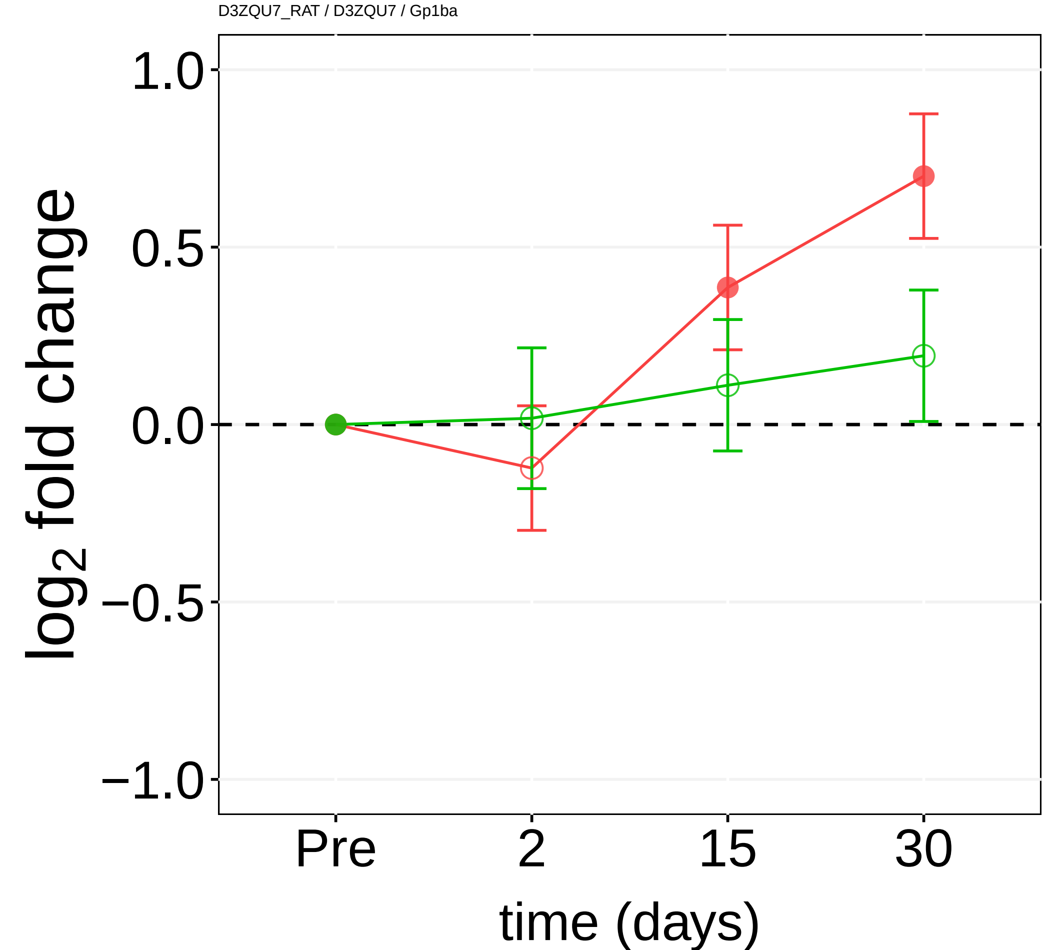
D3ZQU7_RAT / D3ZQU7 / Gp1ba
1.0
0.5
0.0
−0.5
−1.0
log2 fold change
Pre
2
15
30
time (days)

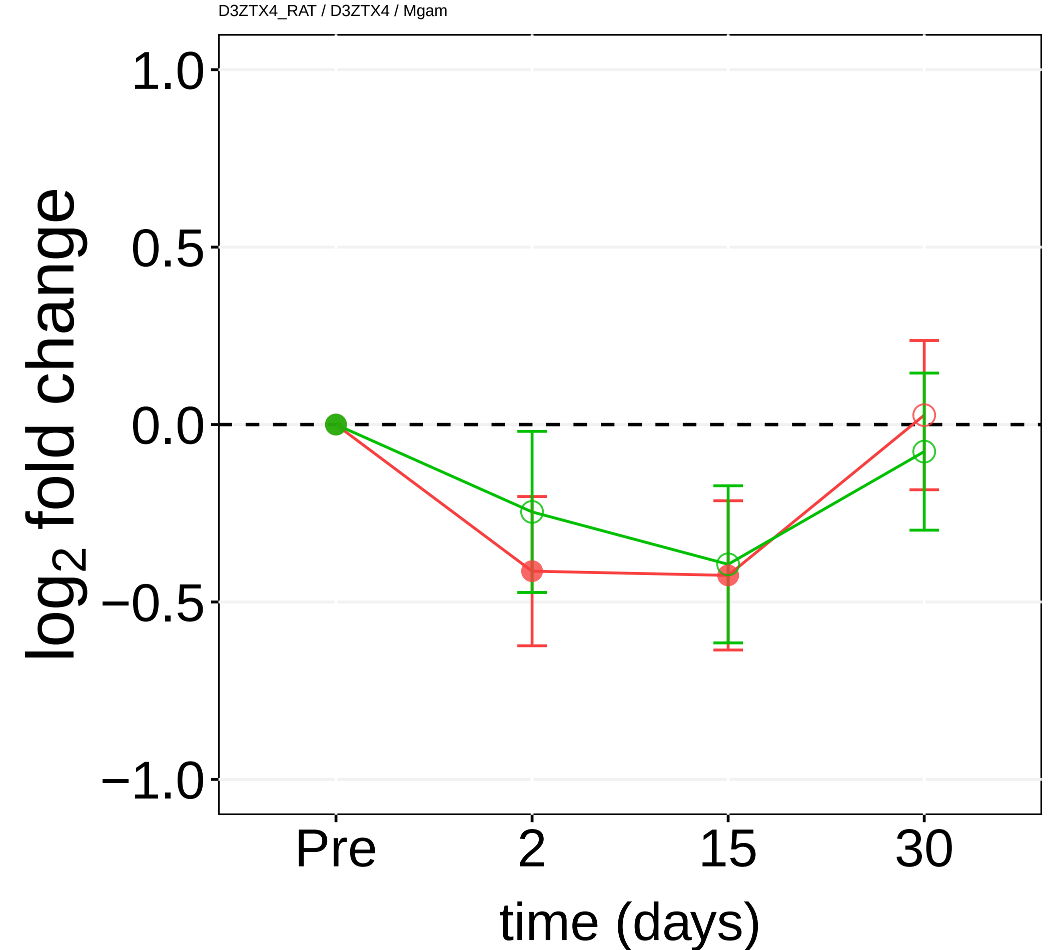
D3ZTX4_RAT / D3ZTX4 / Mgam
1.0
0.5
0.0
−0.5
−1.0
log2 fold change
Pre
2
15
30
time (days)

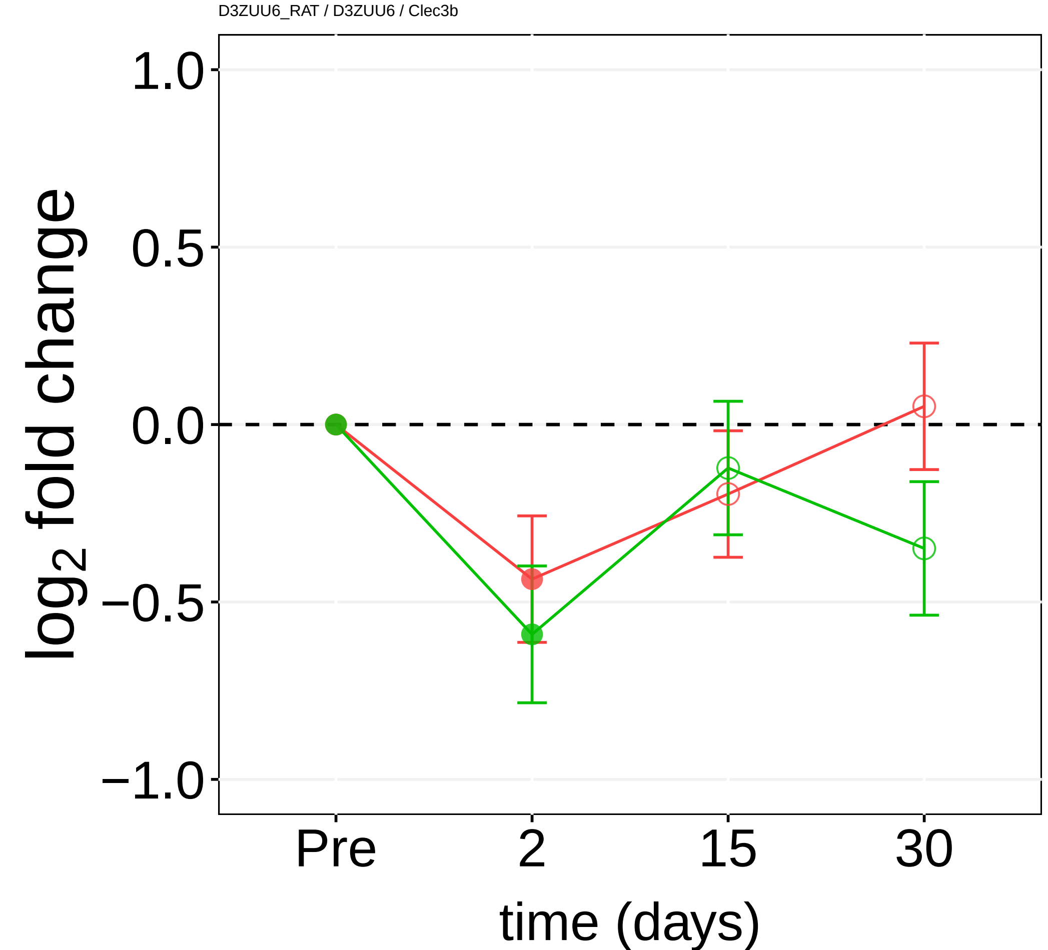

D3ZUU6_RAT / D3ZUU6 / Clec3b
1.0
0.5
0.0
−0.5
−1.0
log2 fold change
Pre
2
15
30
time (days)

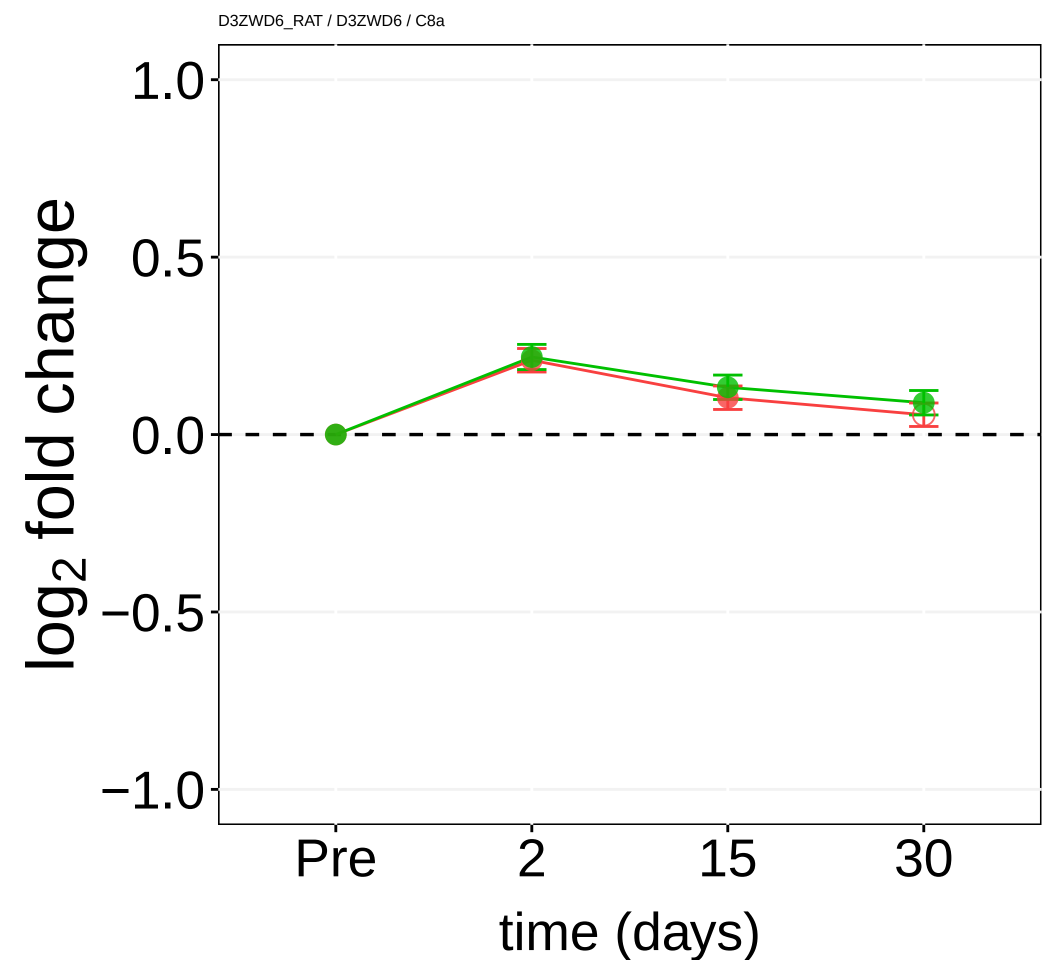
D3ZWD6_RAT / D3ZWD6 / C8a
1.0
0.5
0.0
−0.5
−1.0
log2 fold change
Pre
2
15
30
time (days)

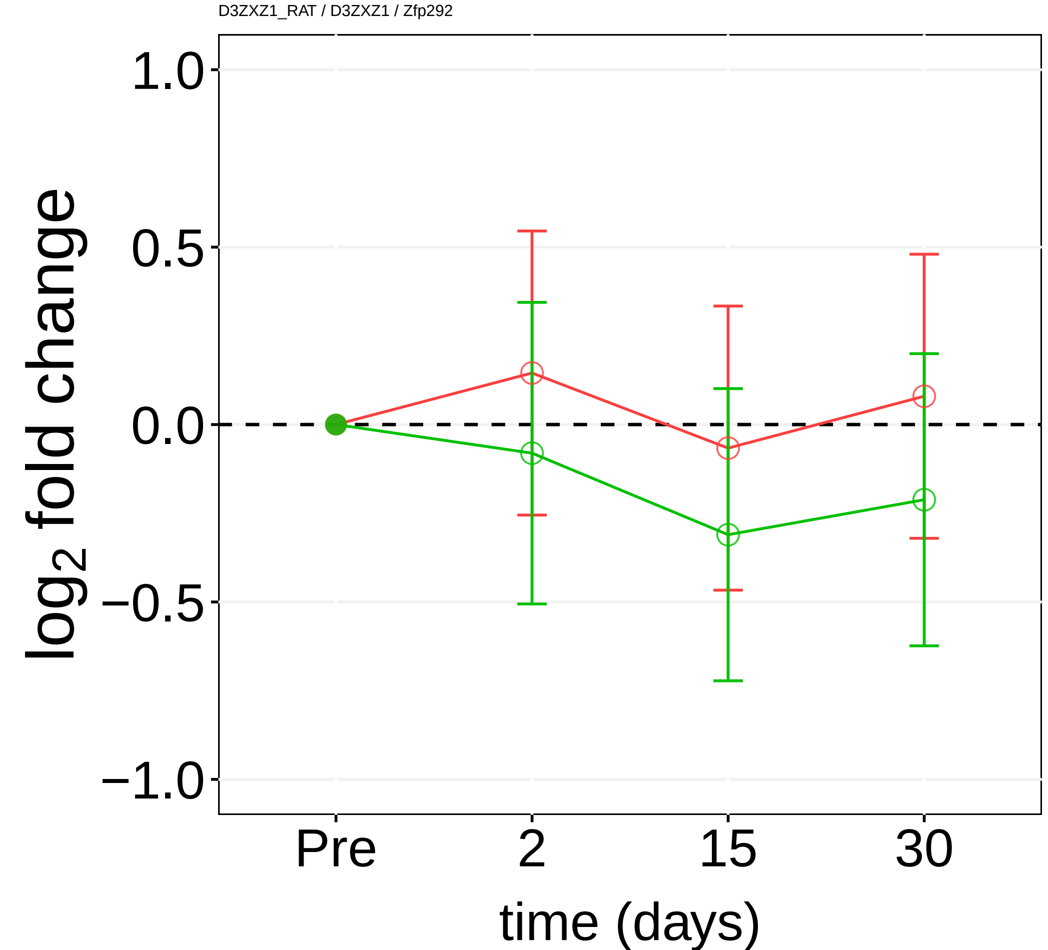
D3ZXZ1_RAT / D3ZXZ1 / Zfp292
1.0
0.5
0.0
−0.5
−1.0
log2 fold change
Pre
2
15
30
time (days)

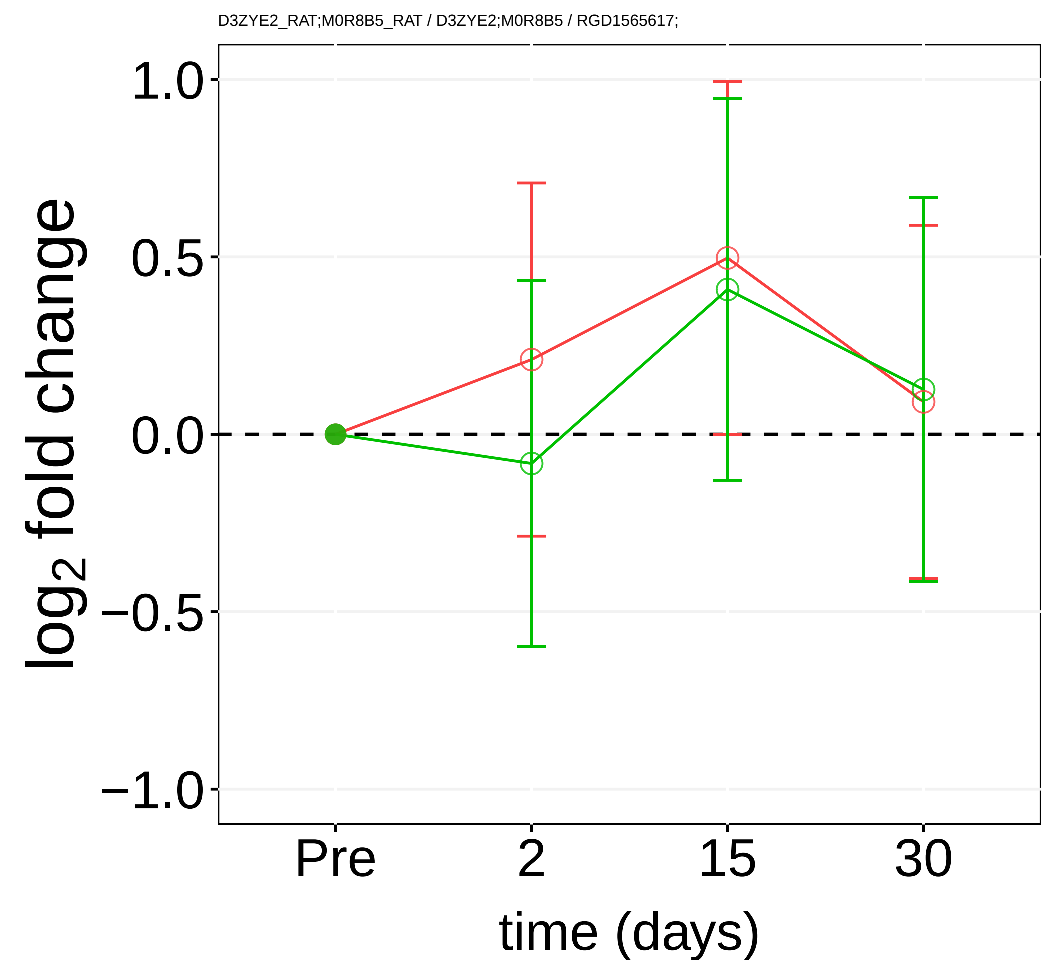
D3ZYE2_RAT;M0R8B5_RAT / D3ZYE2;M0R8B5 / RGD1565617;
1.0
0.5
0.0
−0.5
−1.0
log2 fold change
Pre
2
15
30
time (days)

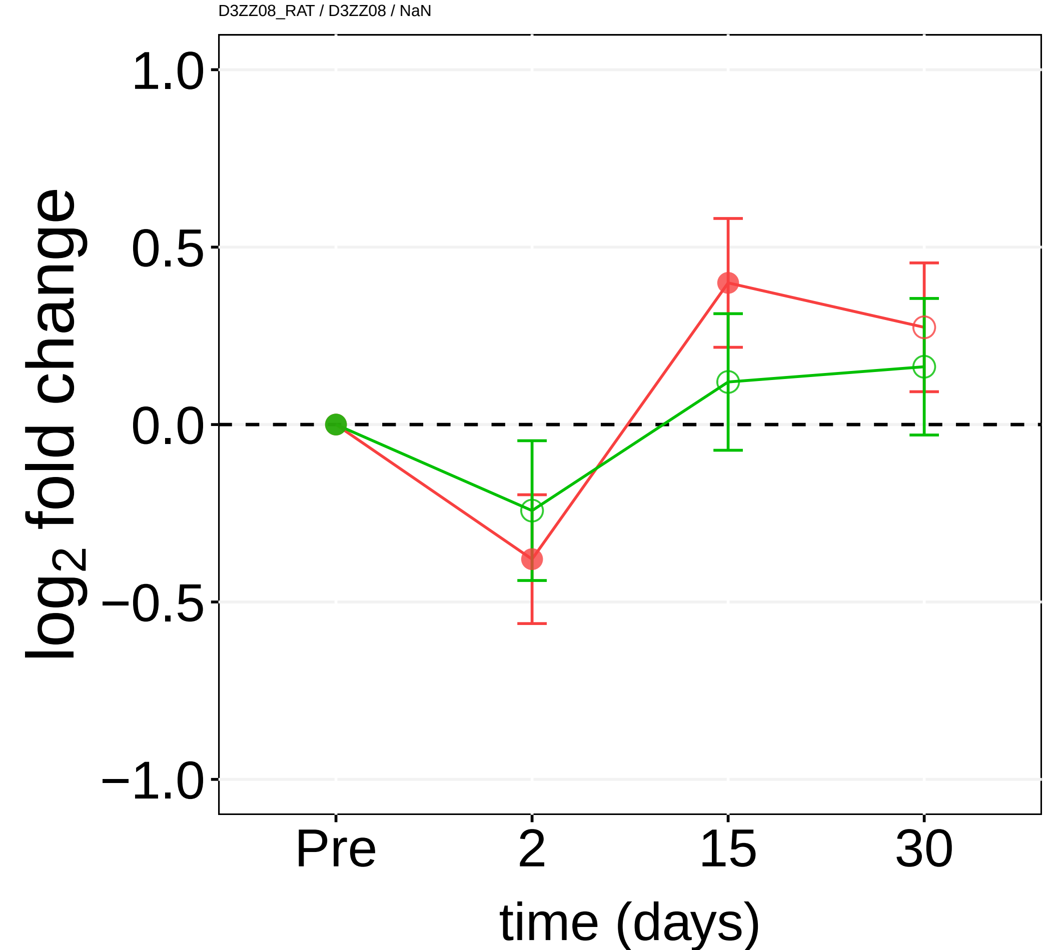
D3ZZ08_RAT / D3ZZ08 / NaN
1.0
0.5
0.0
−0.5
−1.0
log2 fold change
Pre
2
15
30
time (days)

D4A183_RAT / D4A183 / Vnn3

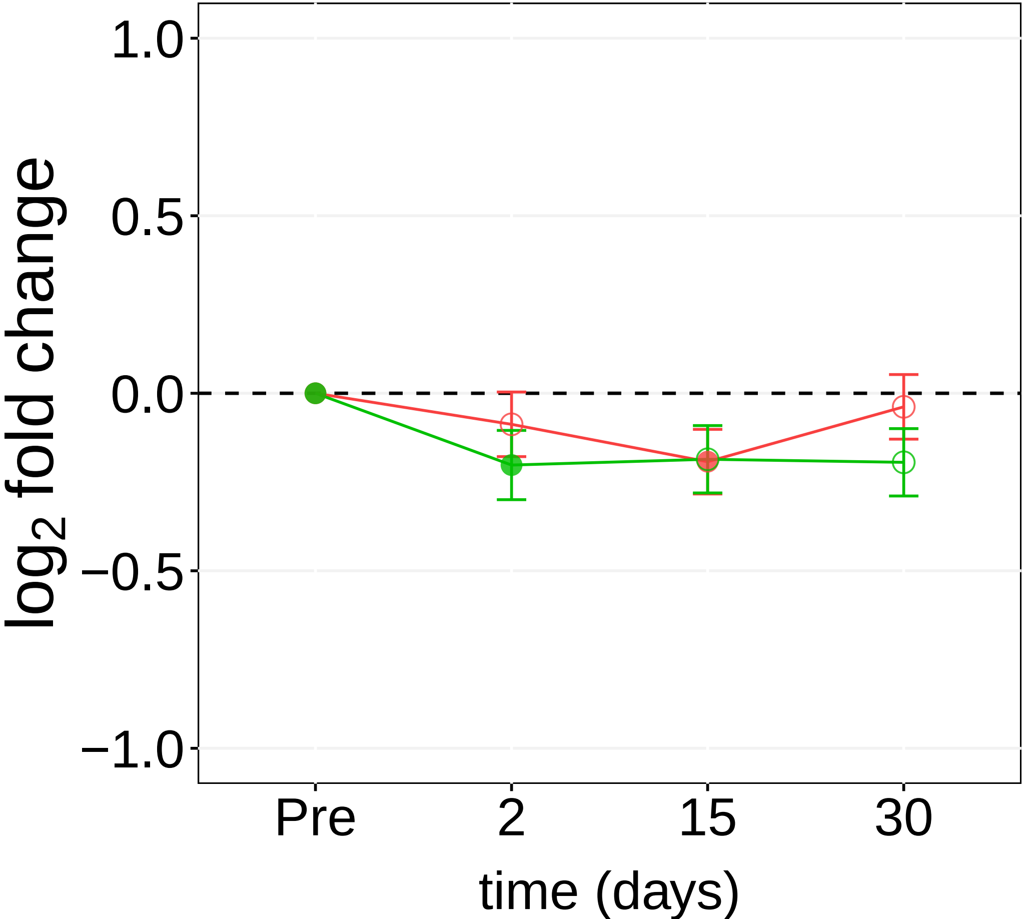

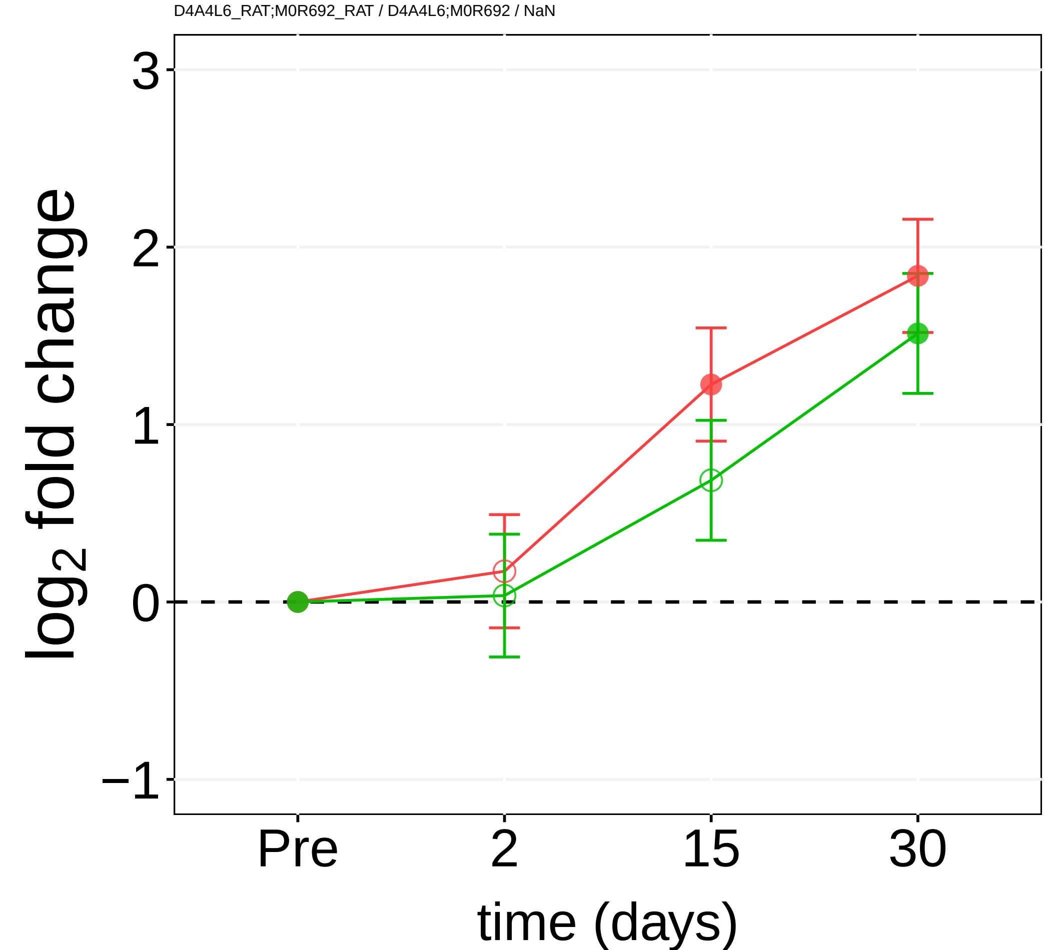

D4A4L6_RAT;M0R692_RAT / D4A4L6;M0R692 / NaN
3
2
1
0
−1
Pre
2
15
30
time (days)
$\log_2$ fold change

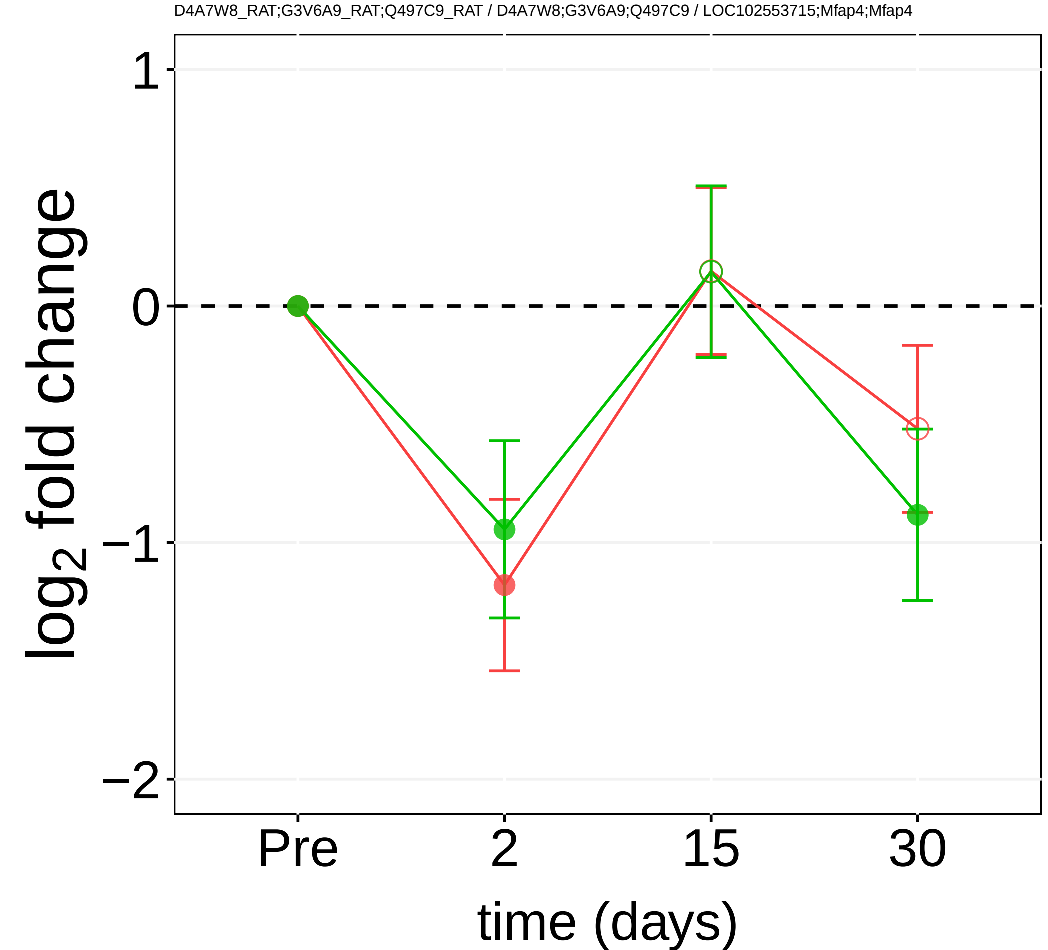
D4A7W8_RAT;G3V6A9_RAT;Q497C9_RAT / D4A7W8;G3V6A9;Q497C9 / LOC102553715;Mfap4;Mfap4
1
0
−1
−2
log2 fold change
Pre
2
15
30
time (days)

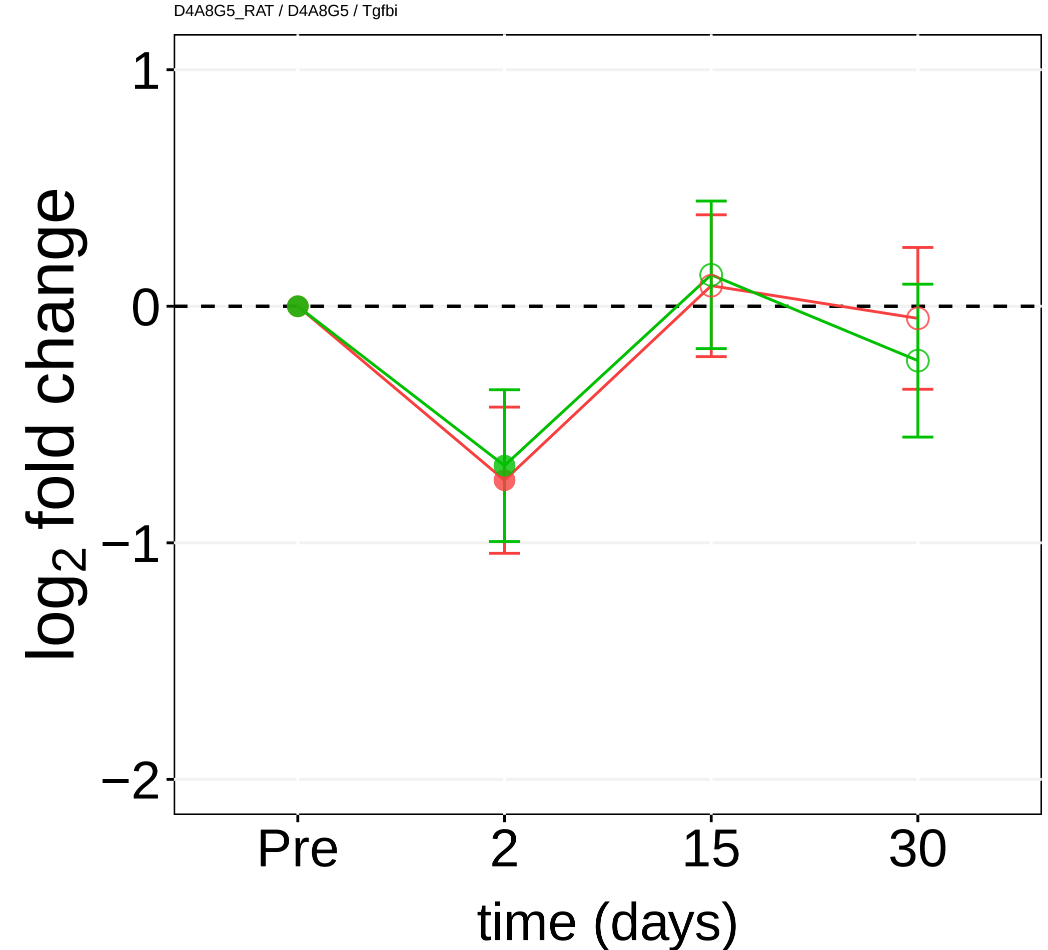
D4A8G5_RAT / D4A8G5 / Tgfbi
1
0
−1
−2
$\log_2$ fold change
Pre
2
15
30
time (days)

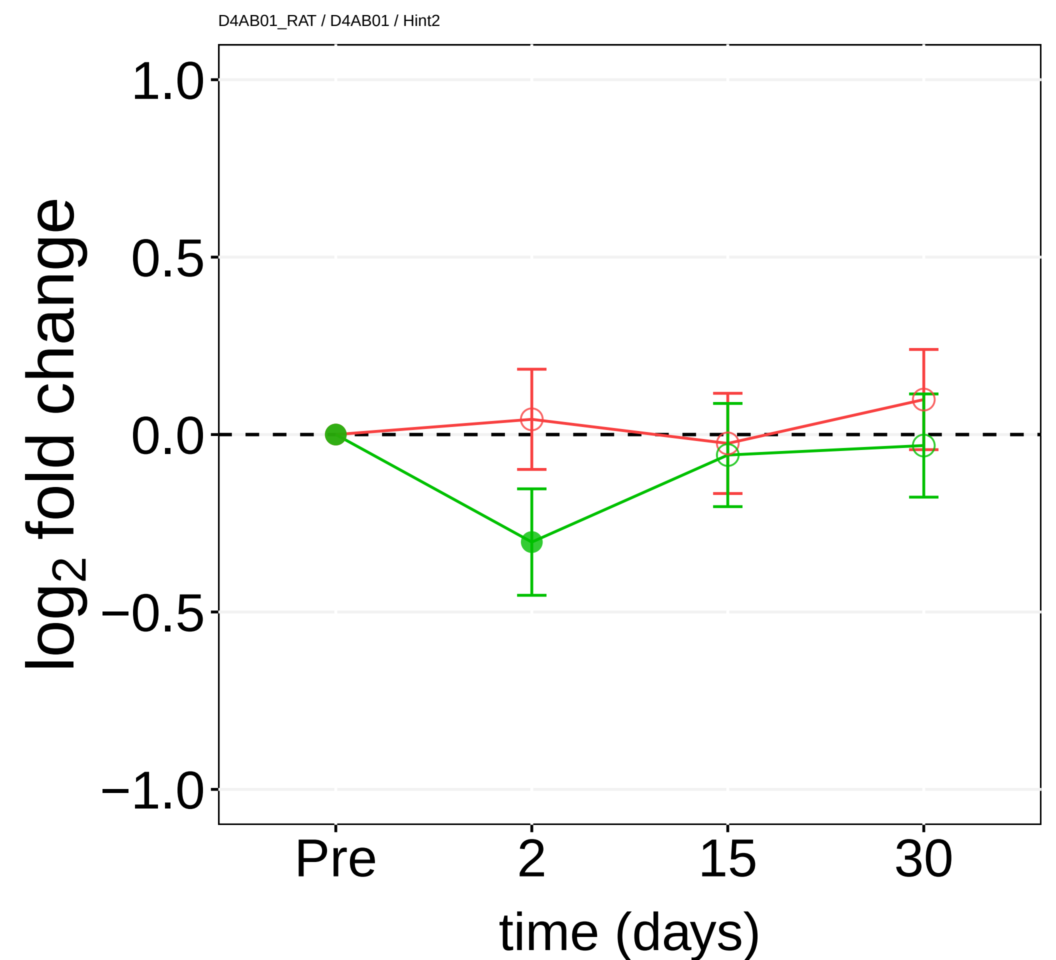
D4AB01_RAT / D4AB01 / Hint2
1.0
0.5
0.0
−0.5
−1.0
log2 fold change
Pre
2
15
30
time (days)

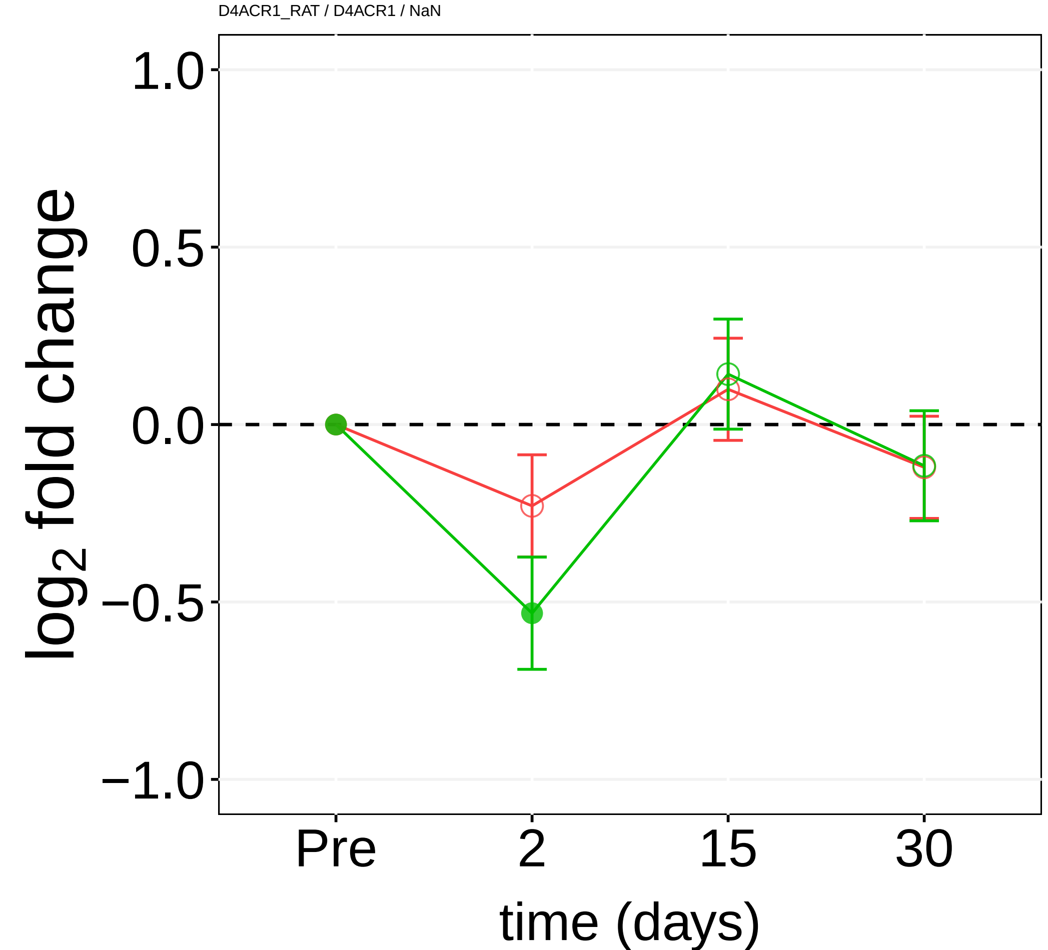

D4ACR1_RAT / D4ACR1 / NaN
1.0
0.5
0.0
−0.5
−1.0
log2 fold change
Pre
2
15
30
time (days)

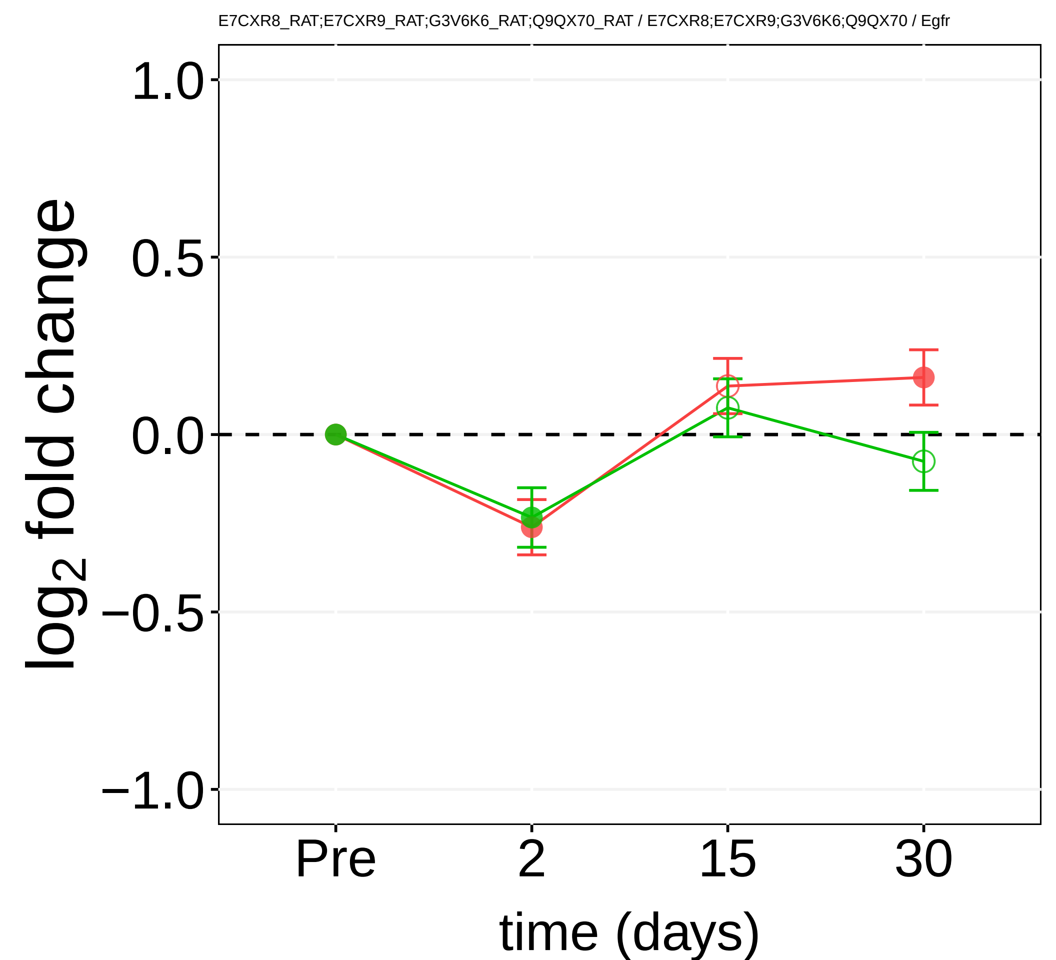
E7CXR8_RAT;E7CXR9_RAT;G3V6K6_RAT;Q9QX70_RAT / E7CXR8;E7CXR9;G3V6K6;Q9QX70 / Egfr
1.0
0.5
0.0
−0.5
−1.0
log2 fold change
Pre
2
15
30
time (days)

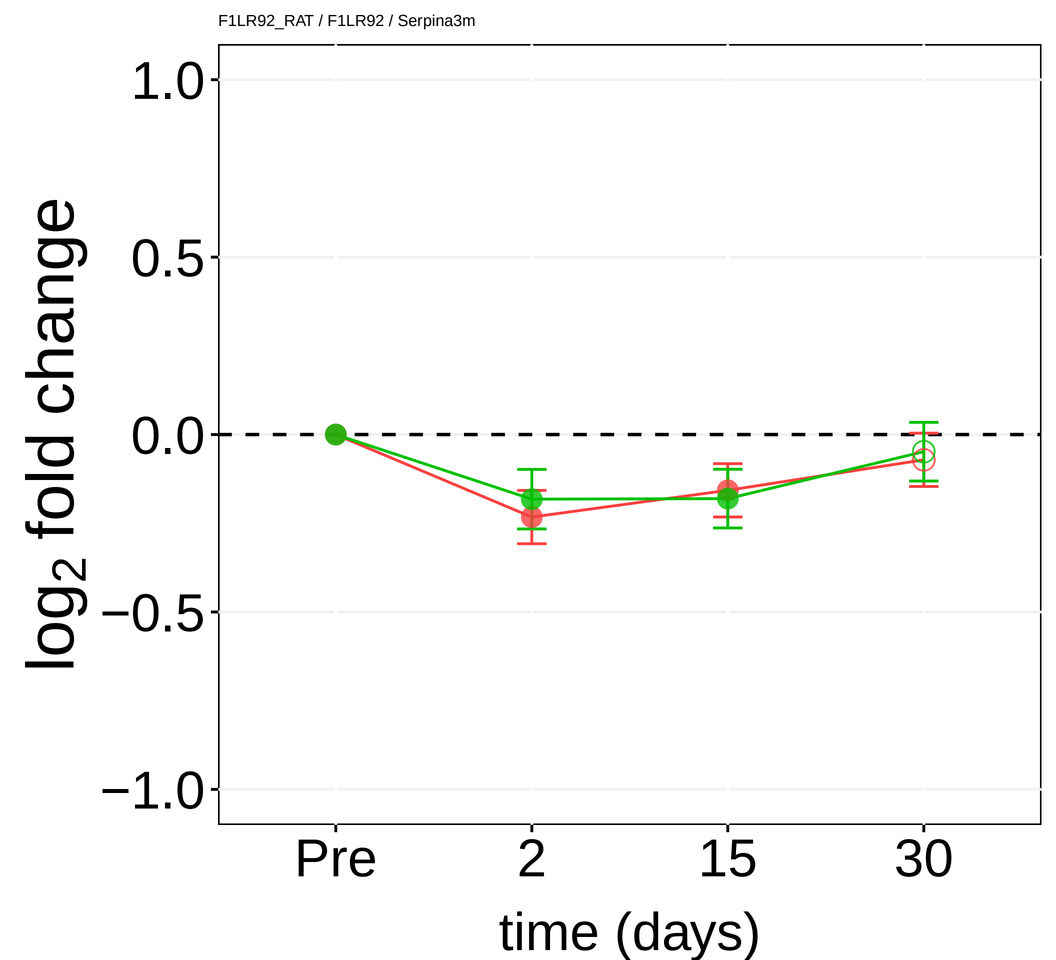
F1LR92_RAT / F1LR92 / Serpina3m
log2 fold change
1.0
0.5
0.0
−0.5
−1.0
Pre
2
15
30
time (days)

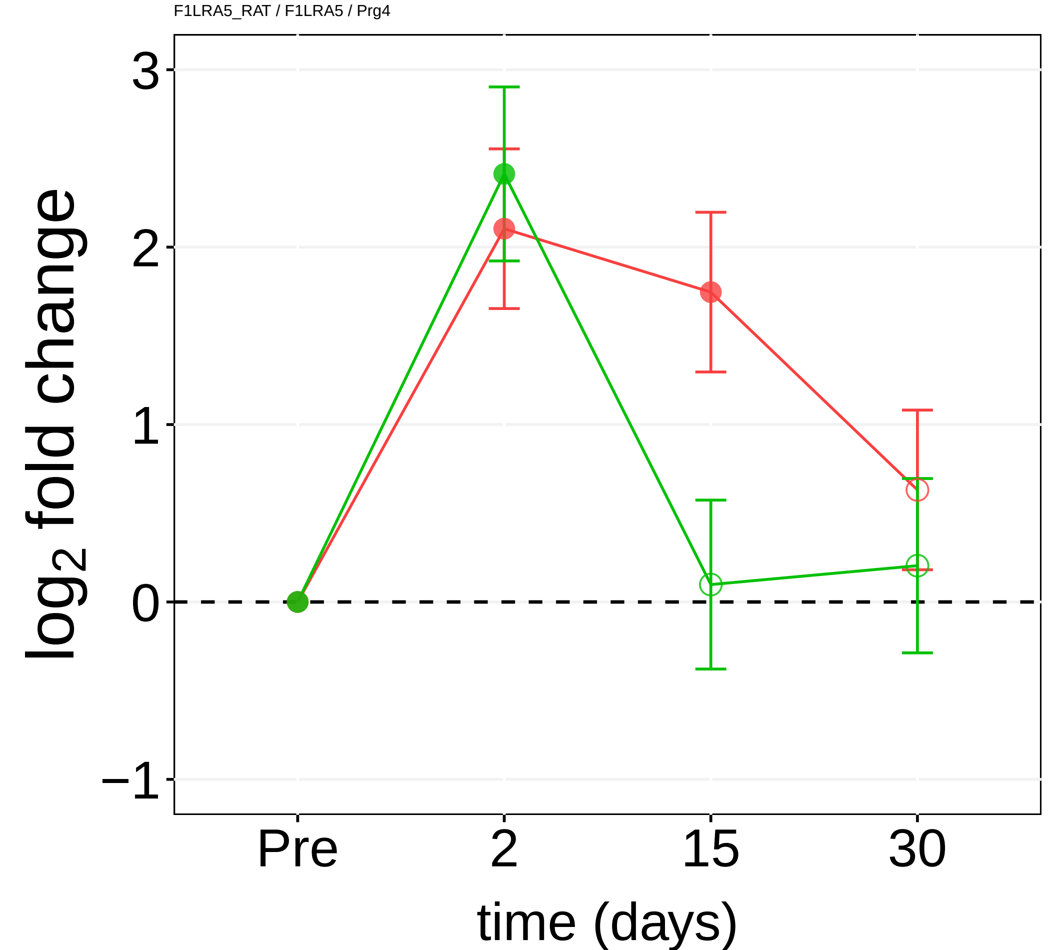

F1LRA5_RAT / F1LRA5 / Prg4
log2 fold change
3
2
1
0
−1
Pre
2
15
30
time (days)

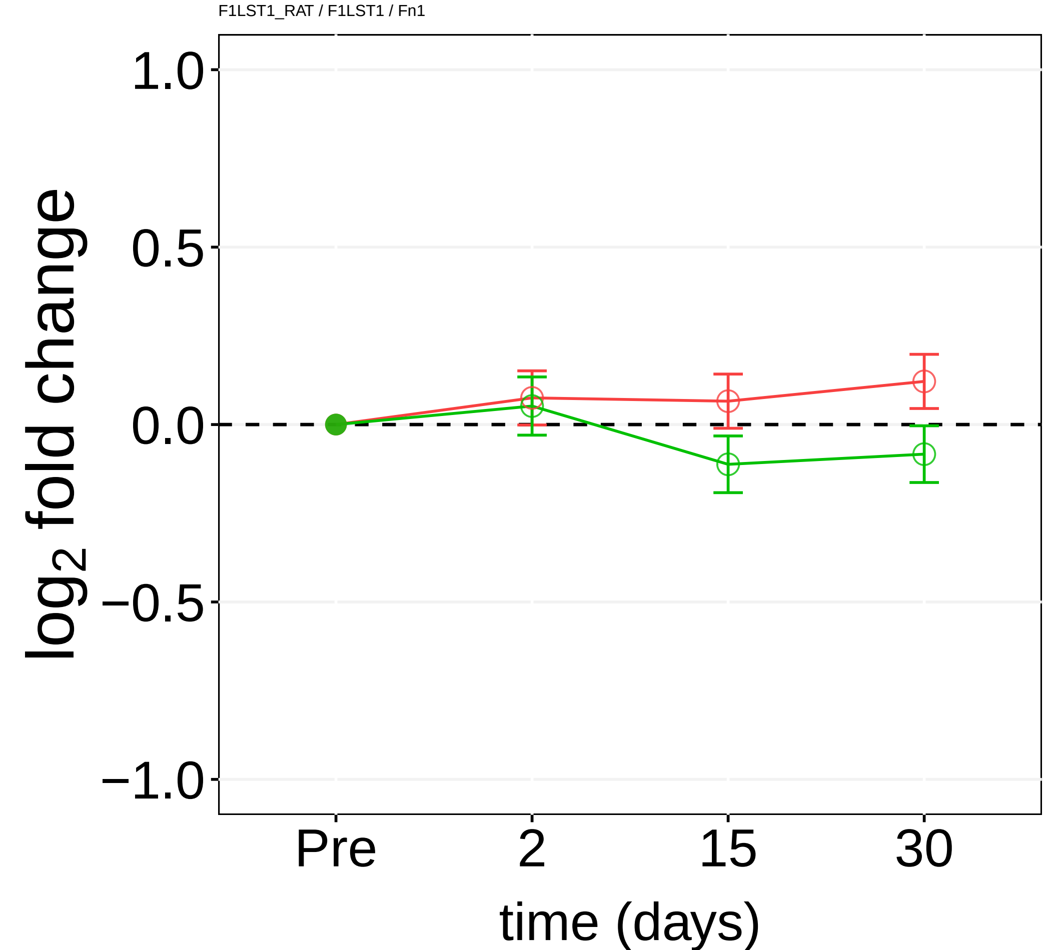
F1LST1_RAT / F1LST1 / Fn1
log2 fold change
1.0
0.5
0.0
−0.5
−1.0
Pre
2
15
30
time (days)

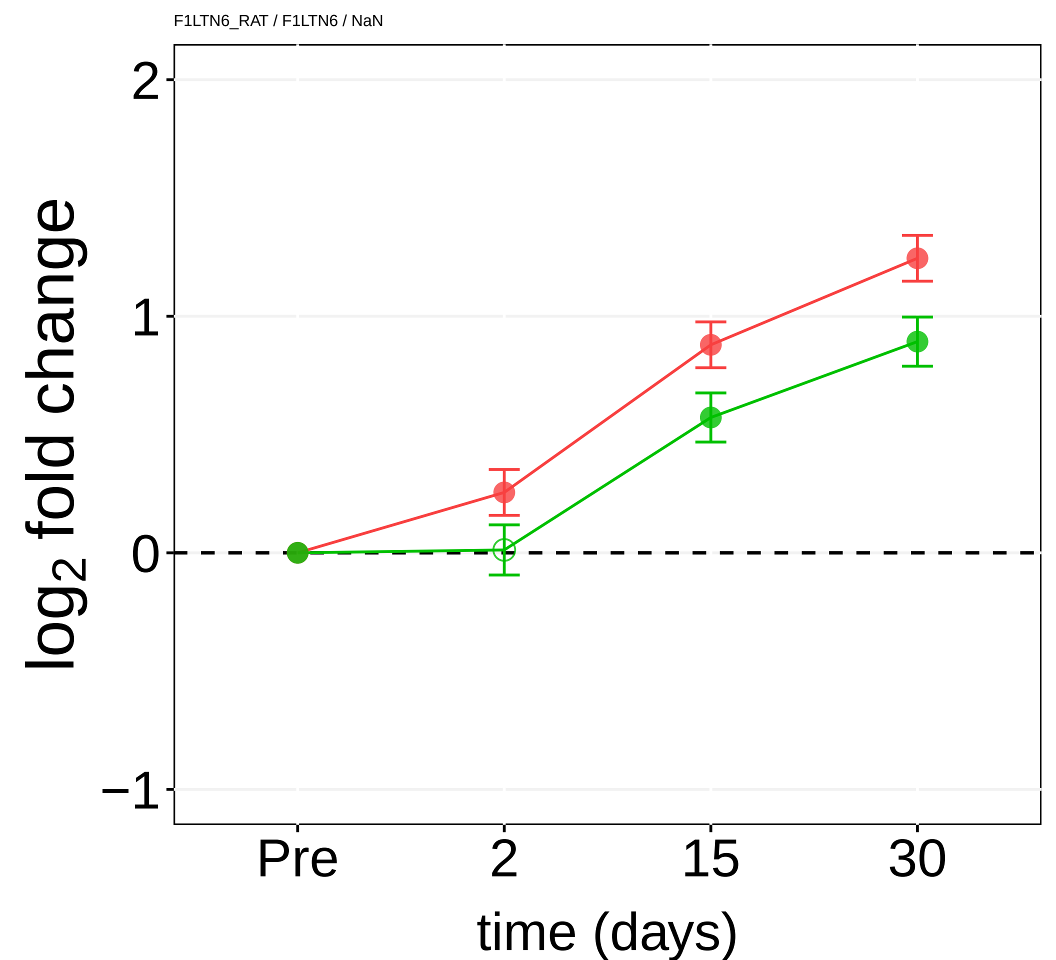
F1LTN6_RAT / F1LTN6 / NaN
2
1
0
−1
log2 fold change
Pre
2
15
30
time (days)

F1LVL2_RAT / F1LVL2 / Icoslg

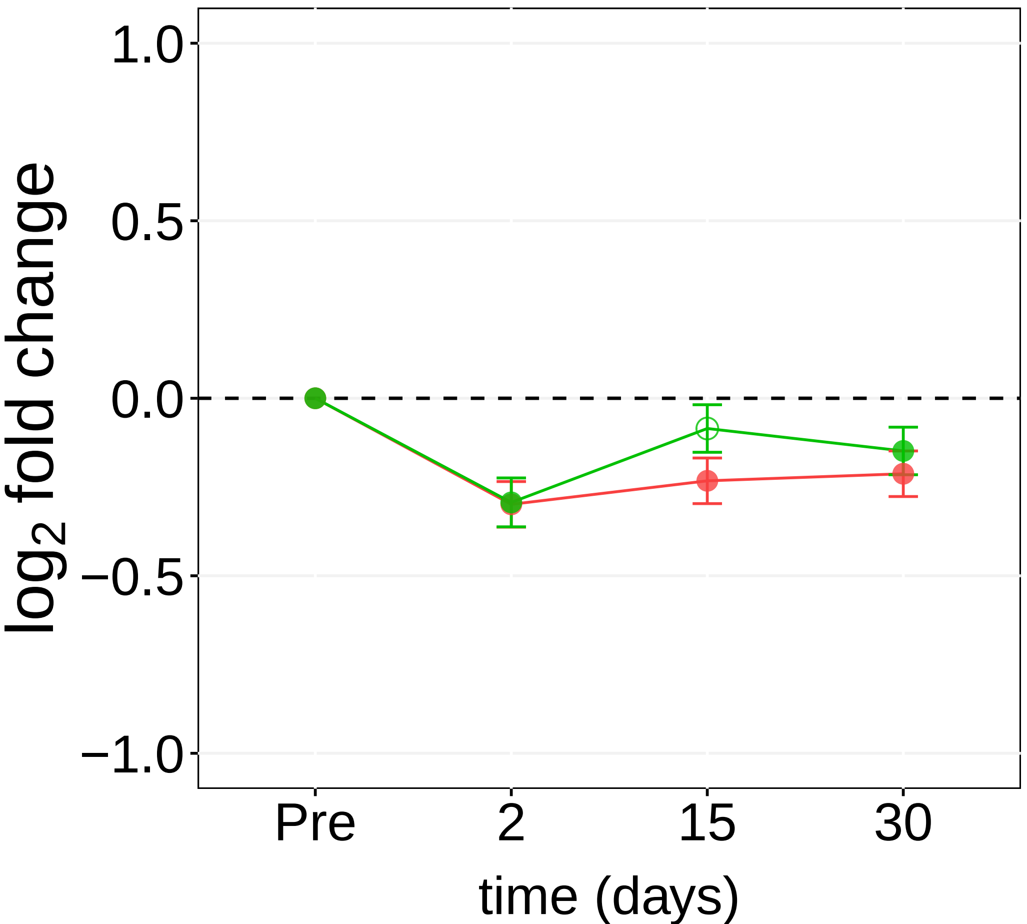

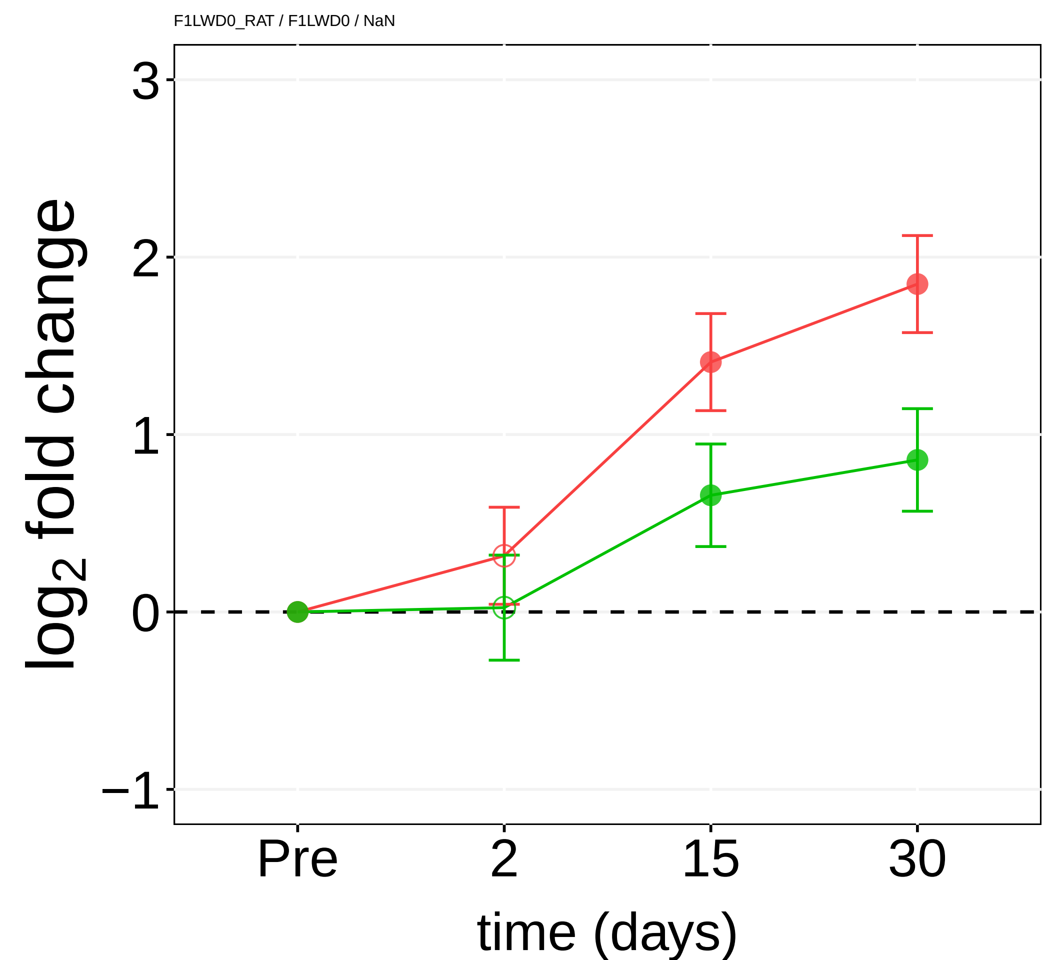
F1LWD0_RAT / F1LWD0 / NaN
log2 fold change
3
2
1
0
−1
Pre
2
15
30
time (days)

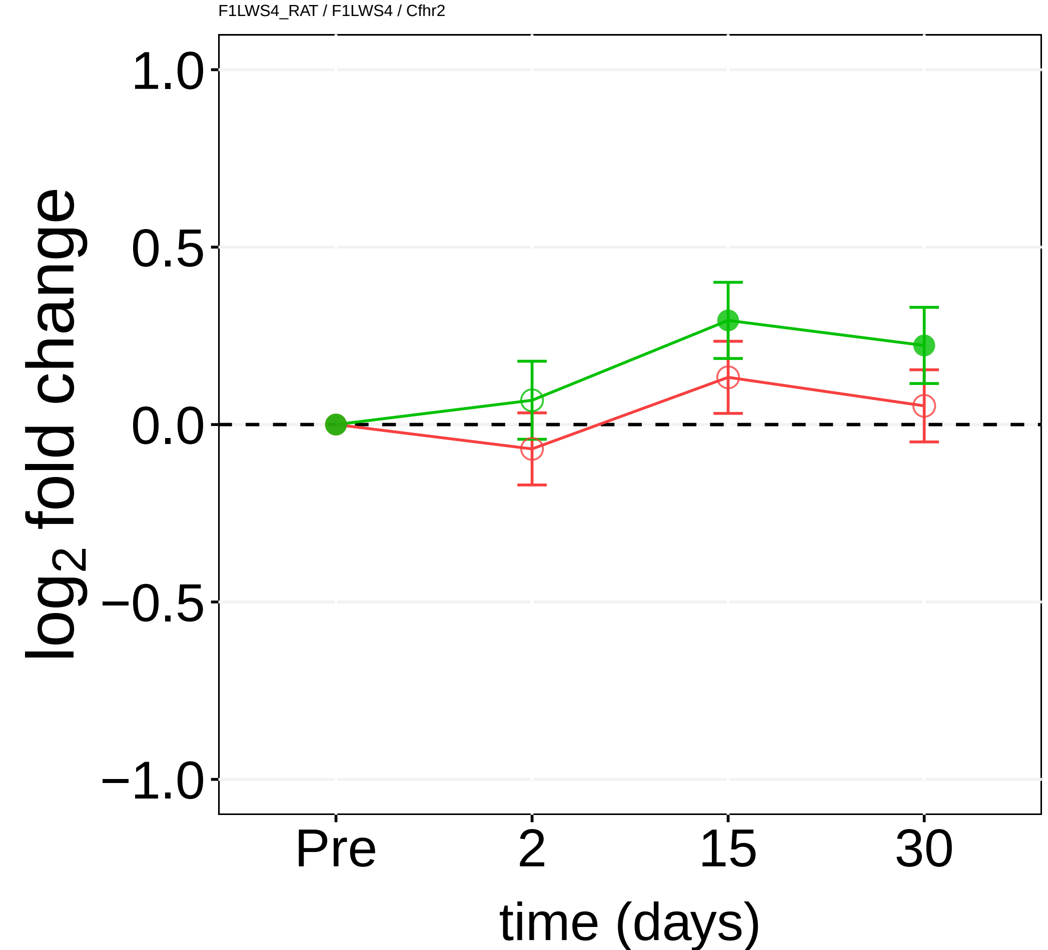
F1LWS4_RAT / F1LWS4 / Cfhr2
1.0
0.5
0.0
−0.5
−1.0
log2 fold change
Pre
2
15
30
time (days)

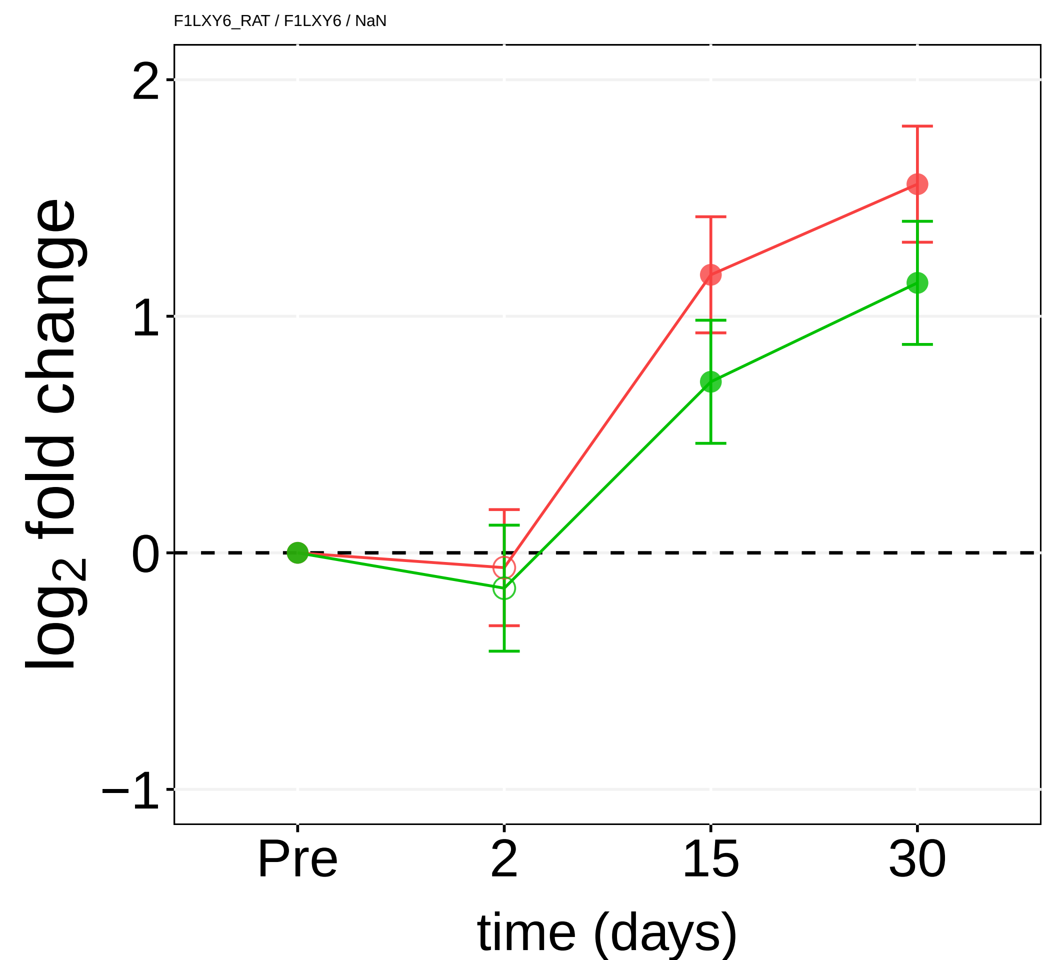

F1LXY6_RAT / F1LXY6 / NaN
2
1
0
−1
log2 fold change
Pre
2
15
30
time (days)

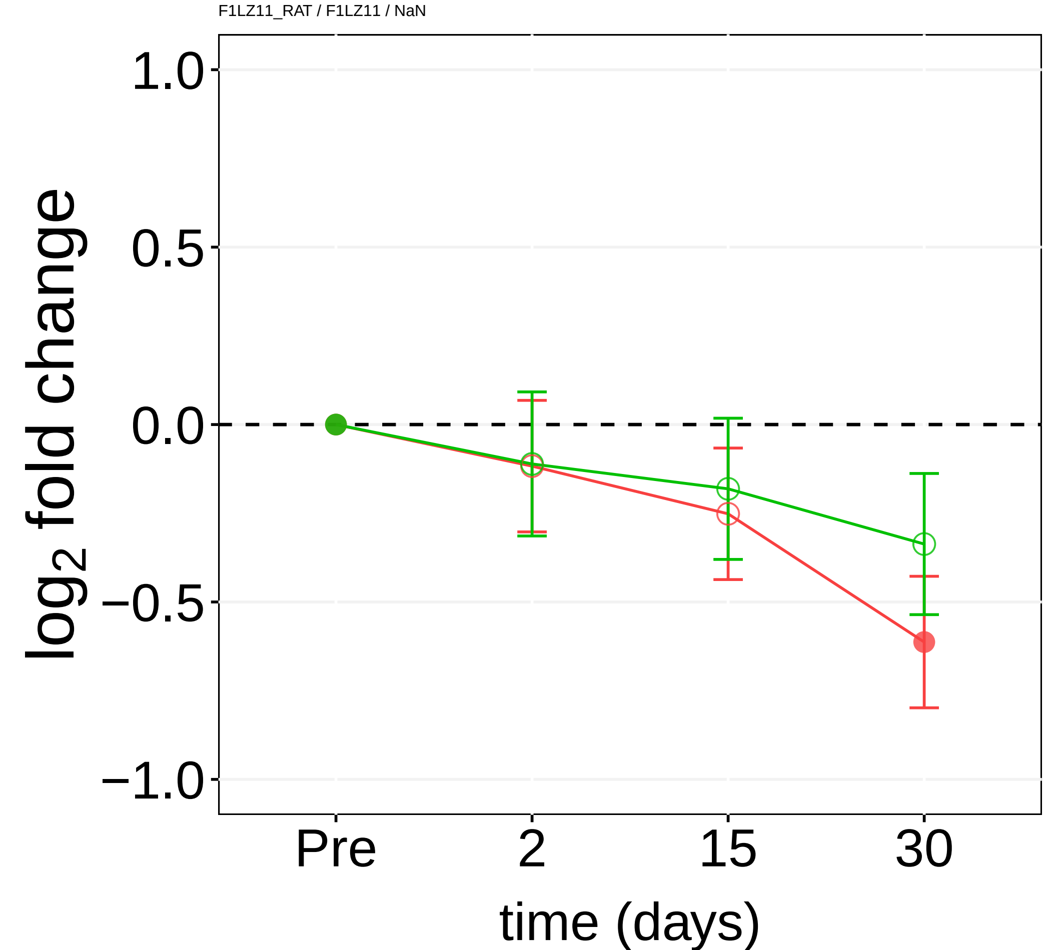
F1LZ11_RAT / F1LZ11 / NaN
log2 fold change
1.0
0.5
0.0
−0.5
−1.0
Pre
2
15
30
time (days)

F1LZH0_RAT / F1LZH0 / NaN

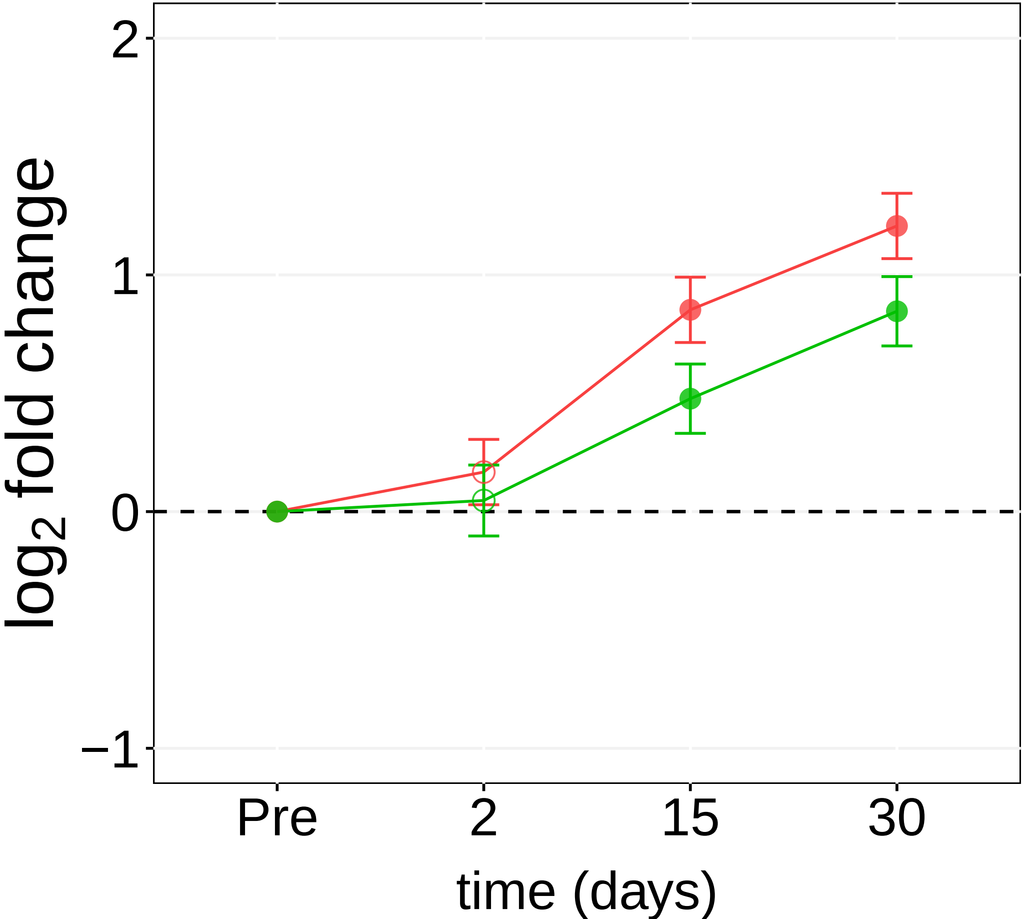

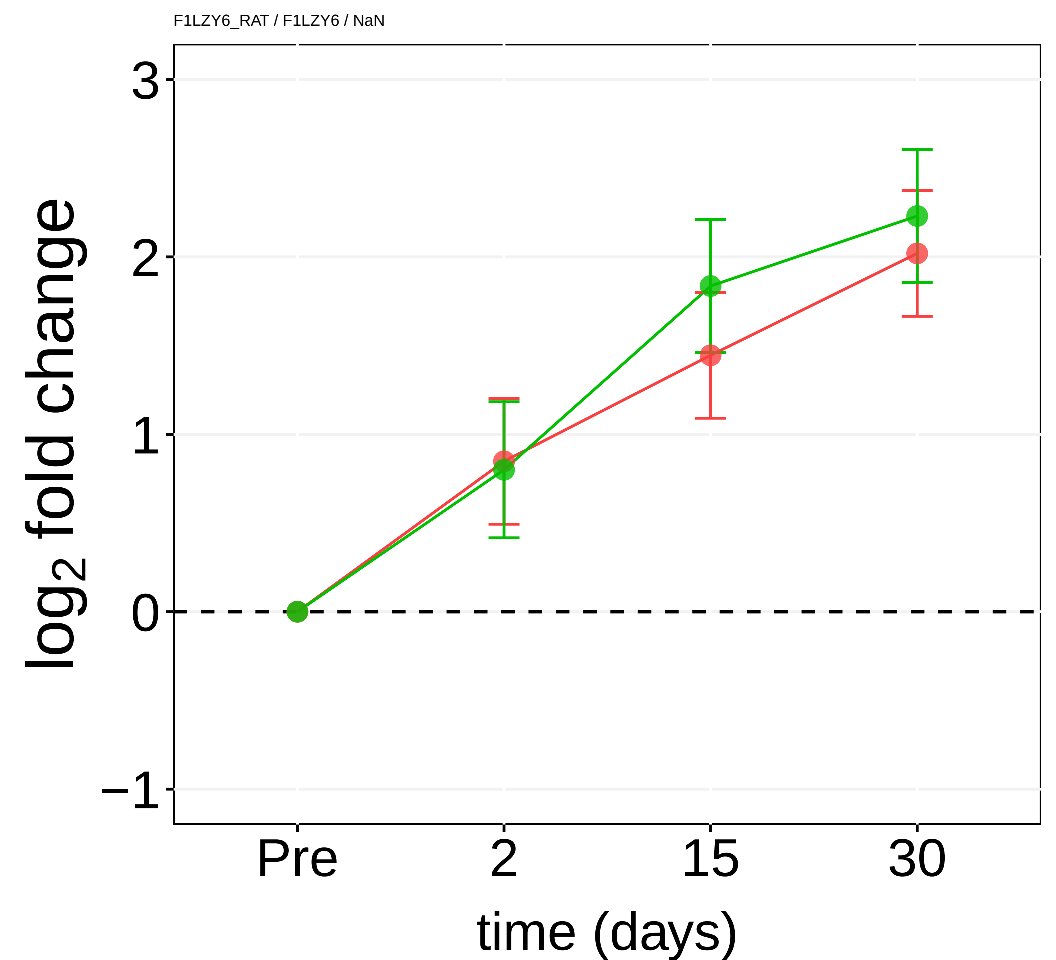

F1LZY6_RAT / F1LZY6 / NaN
log2 fold change
3
2
1
0
−1
Pre
2
15
30
time (days)

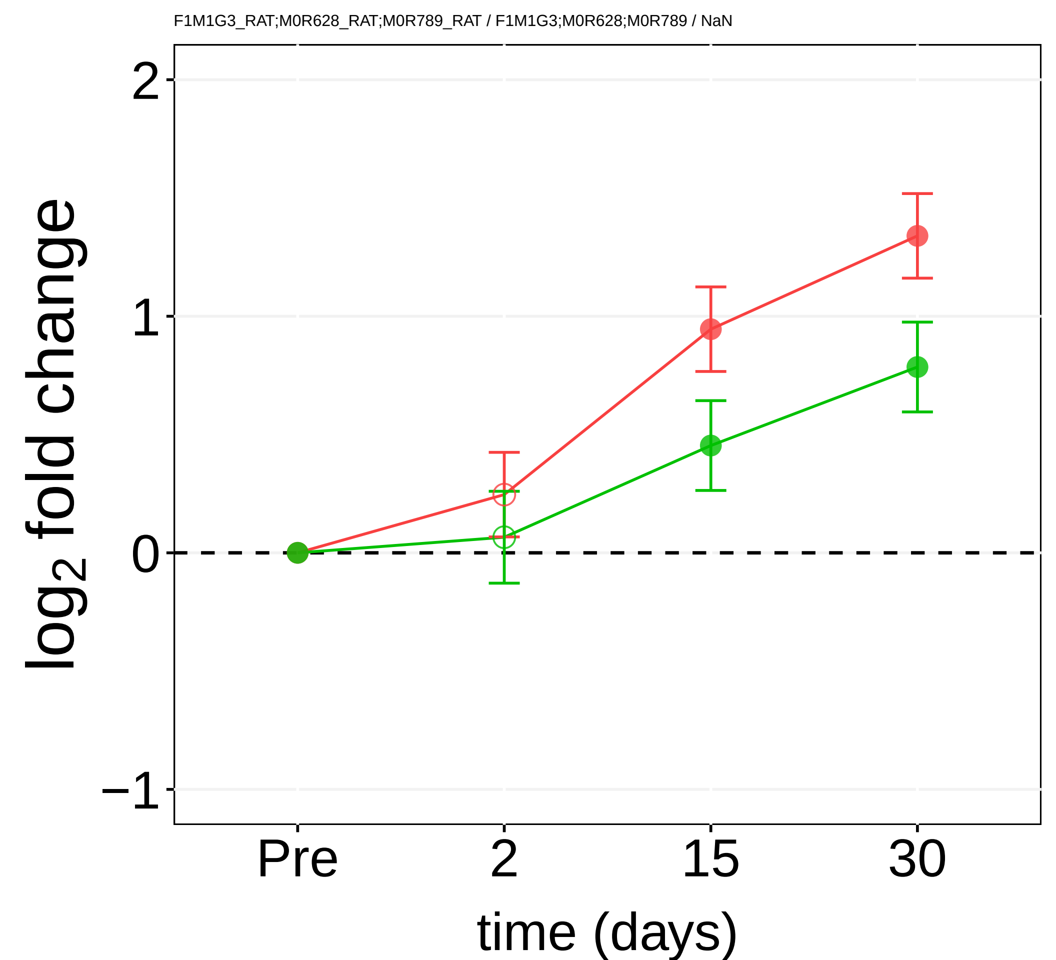
F1M1G3_RAT;M0R628_RAT;M0R789_RAT / F1M1G3;M0R628;M0R789 / NaN
log2 fold change
2
1
0
−1
Pre
2
15
30
time (days)

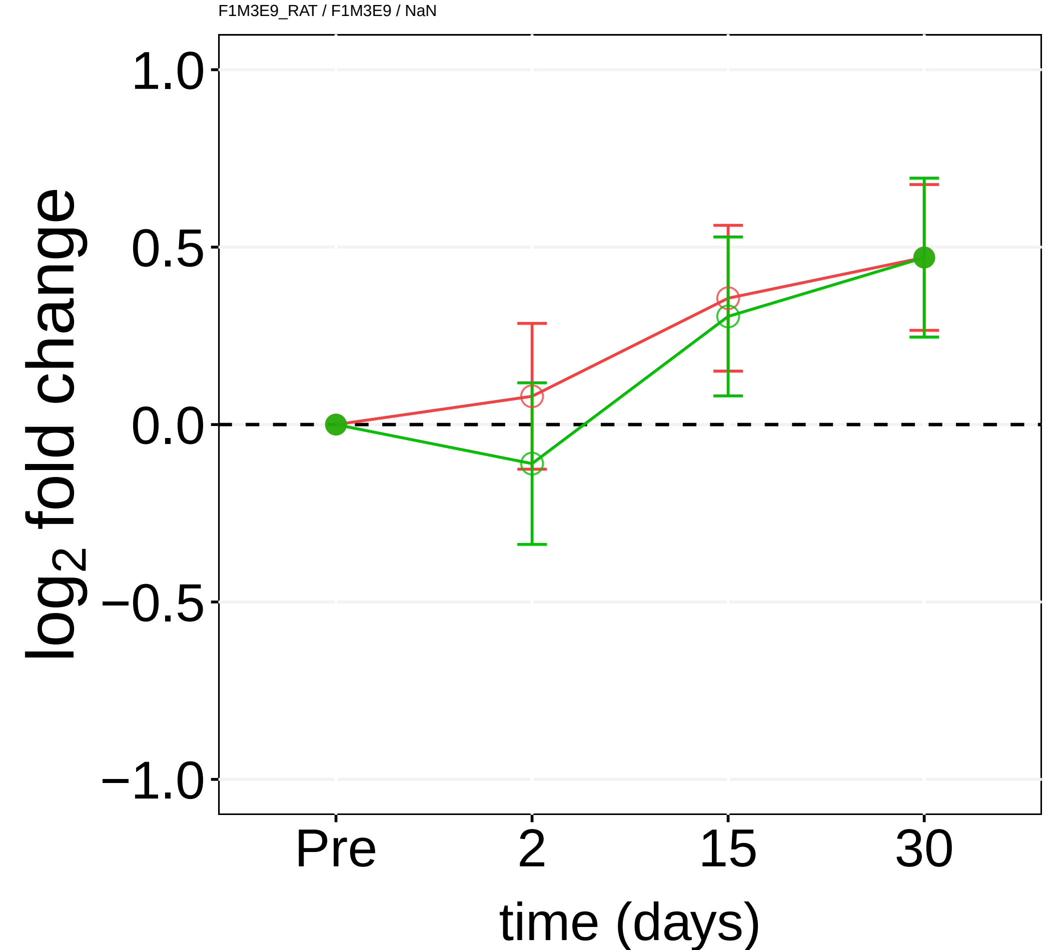
F1M3E9_RAT / F1M3E9 / NaN
1.0
0.5
0.0
−0.5
−1.0
log2 fold change
Pre
2
15
30
time (days)

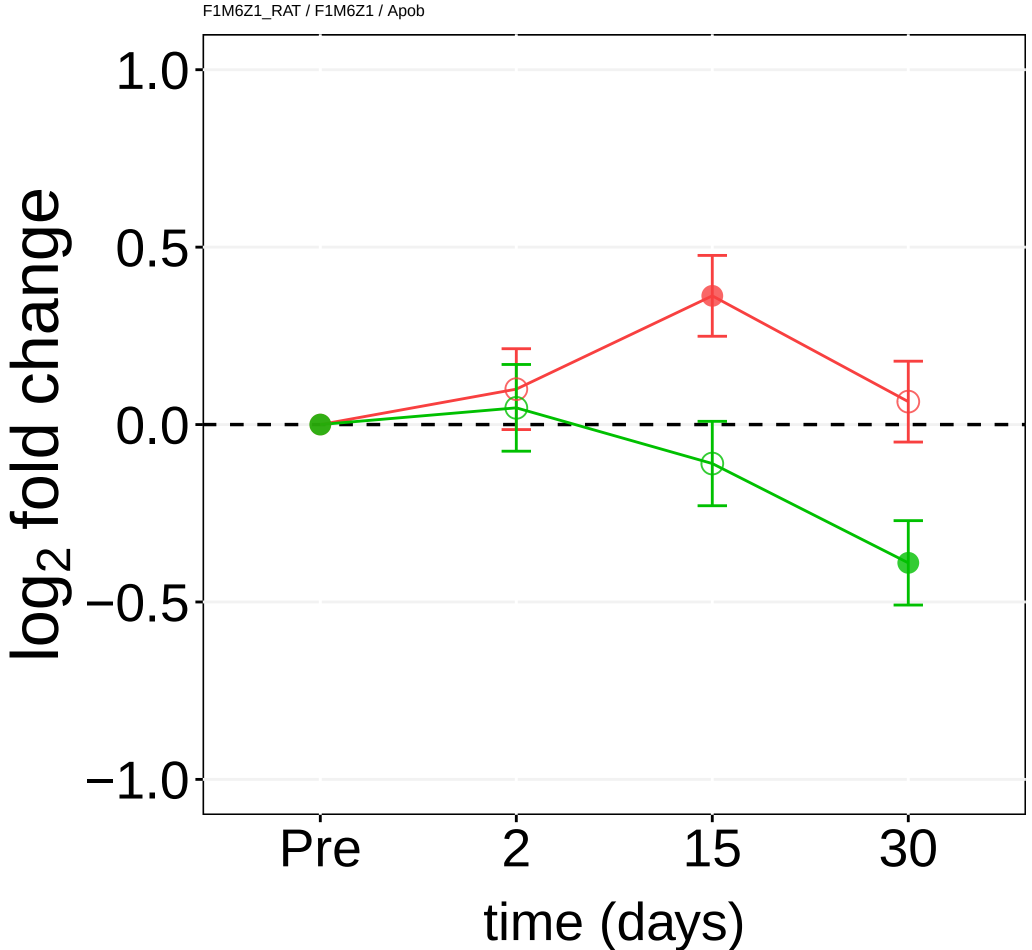
F1M6Z1_RAT / F1M6Z1 / Apob
1.0
0.5
0.0
−0.5
−1.0
log2 fold change
Pre
2
15
30
time (days)

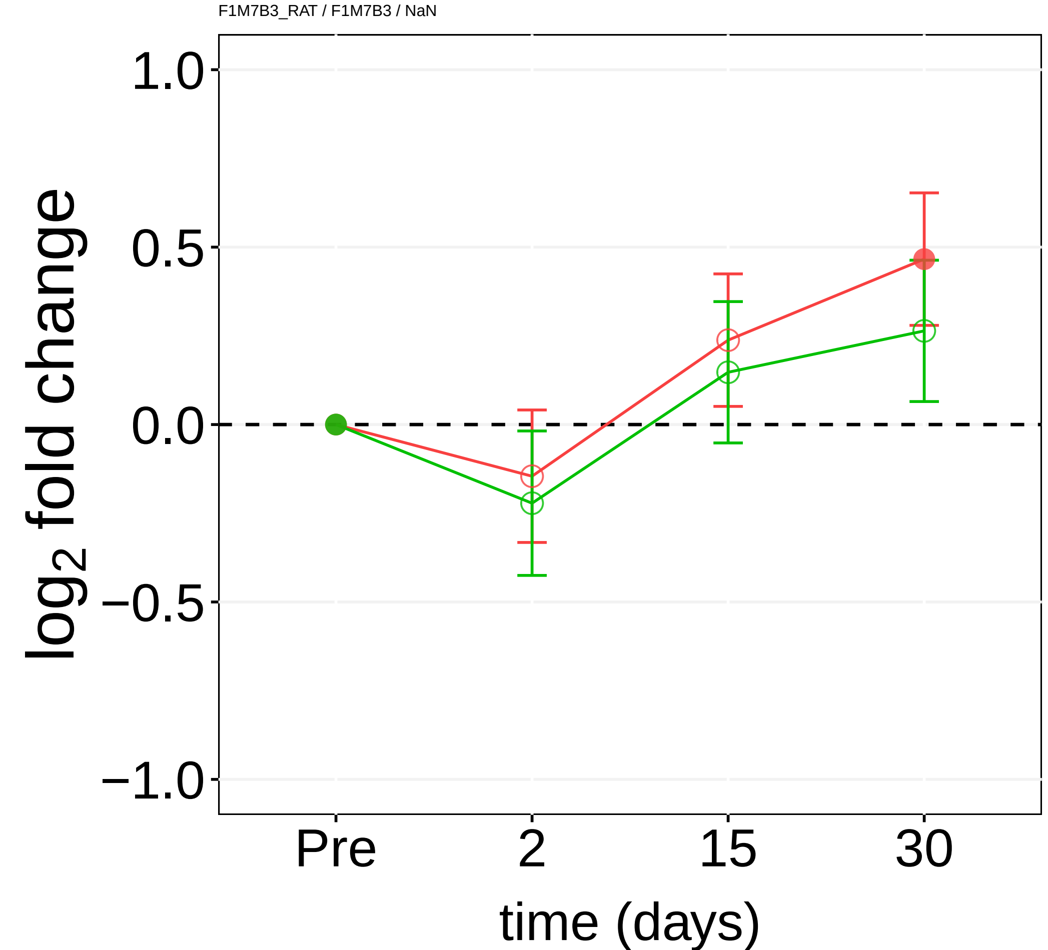
F1M7B3_RAT / F1M7B3 / NaN
1.0
0.5
0.0
−0.5
−1.0
log2 fold change
Pre
2
15
30
time (days)

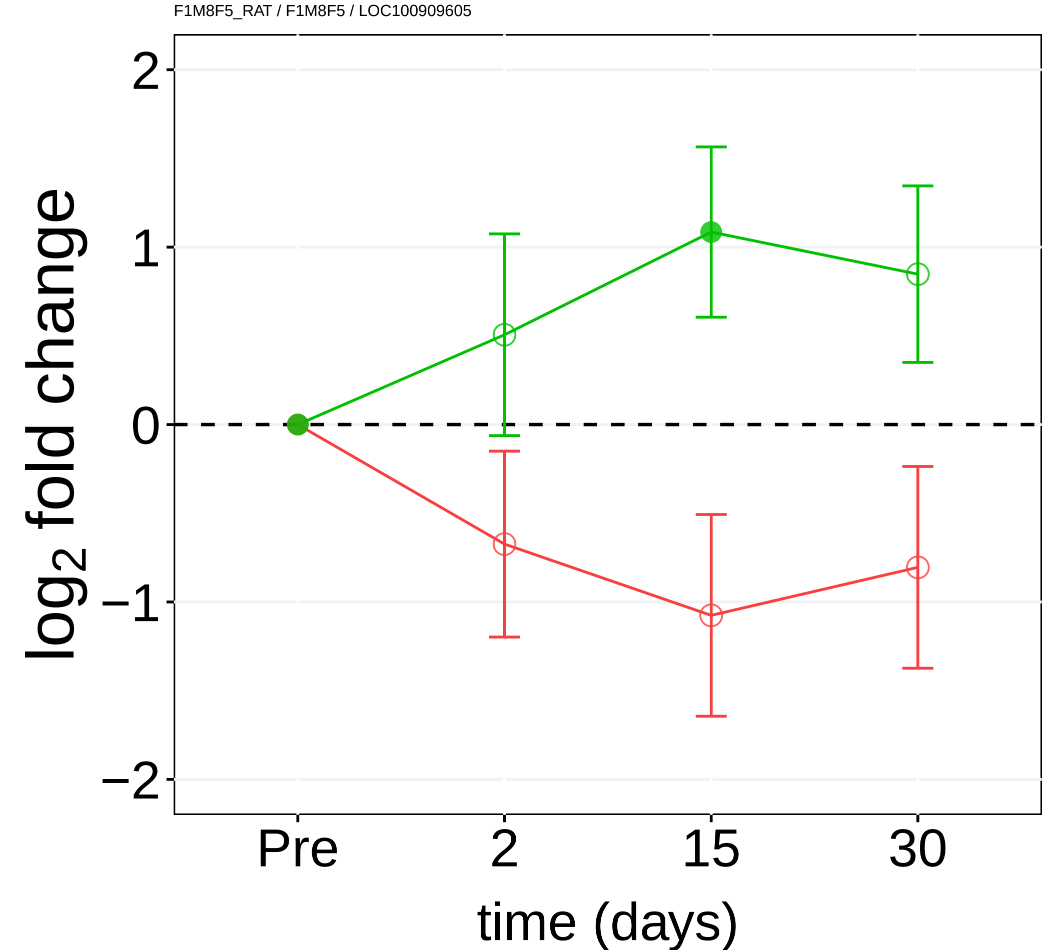
F1M8F5_RAT / F1M8F5 / LOC100909605
log2 fold change
2
1
0
−1
−2
Pre
2
15
30
time (days)

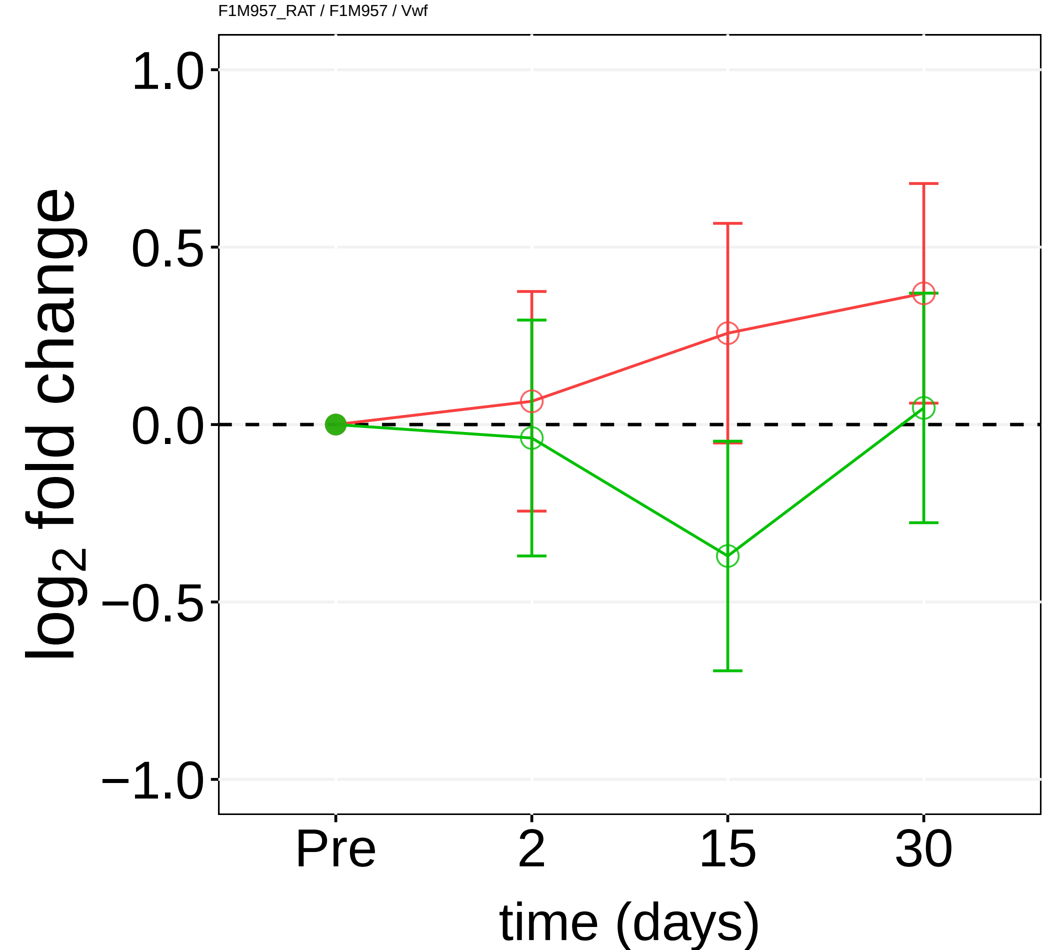

F1M957_RAT / F1M957 / Vwf
1.0
0.5
0.0
−0.5
−1.0
$\log_2$ fold change
Pre
2
15
30
time (days)

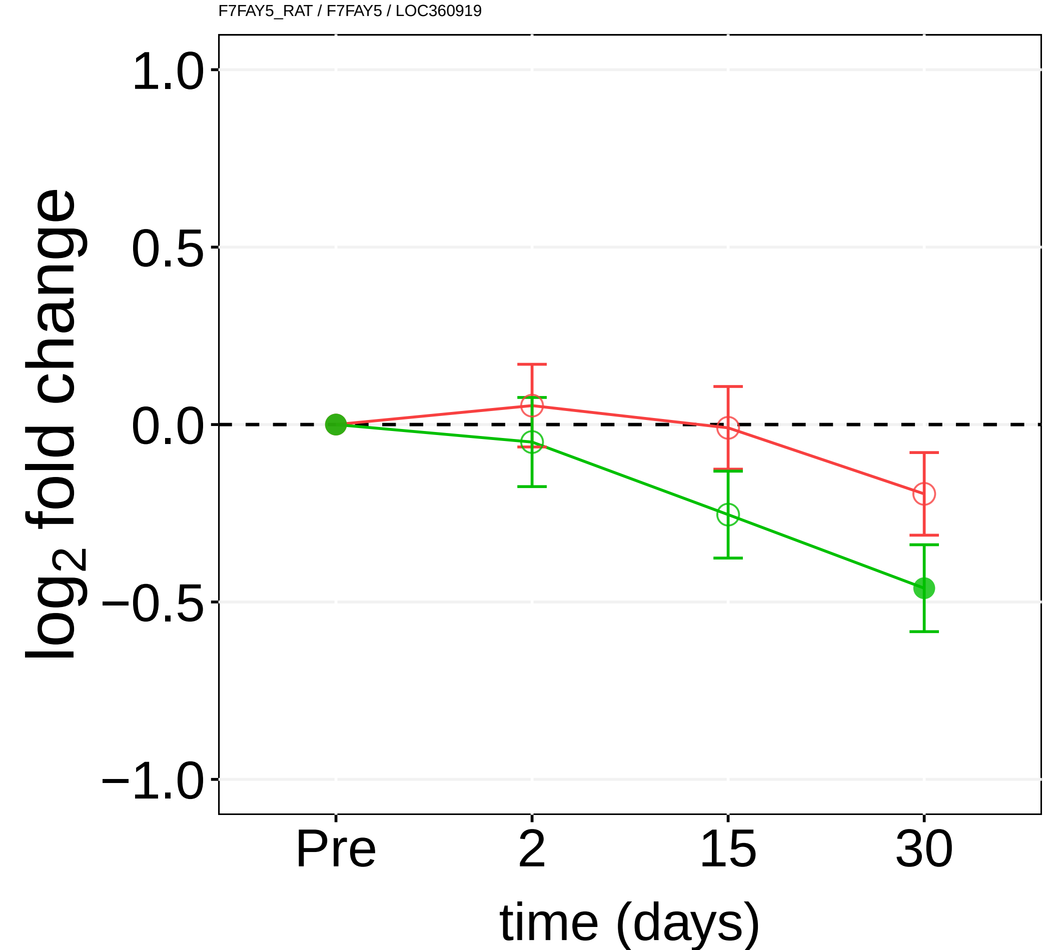
F7FAY5_RAT / F7FAY5 / LOC360919
1.0
0.5
0.0
−0.5
−1.0
log2 fold change
Pre
2
15
30
time (days)

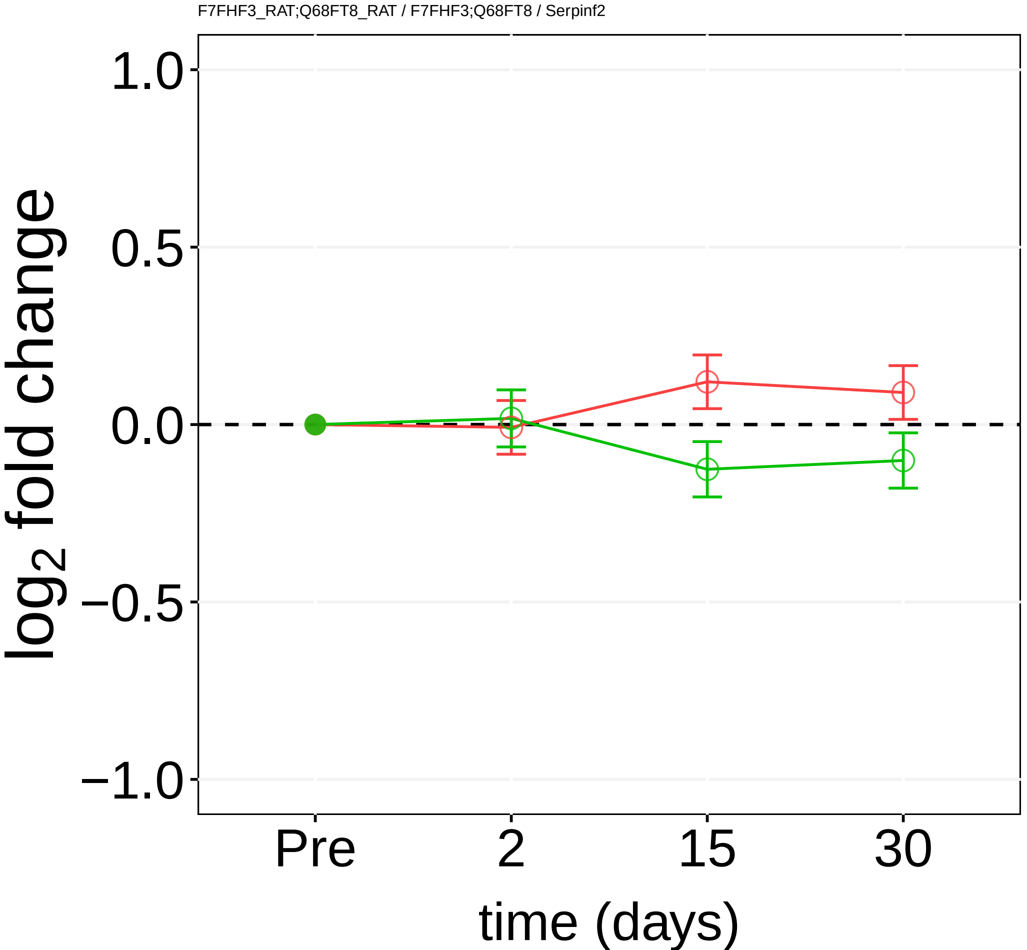

F7FHF3_RAT;Q68FT8_RAT / F7FHF3;Q68FT8 / Serpinf2
1.0
0.5
0.0
−0.5
−1.0
log2 fold change
Pre
2
15
30
time (days)

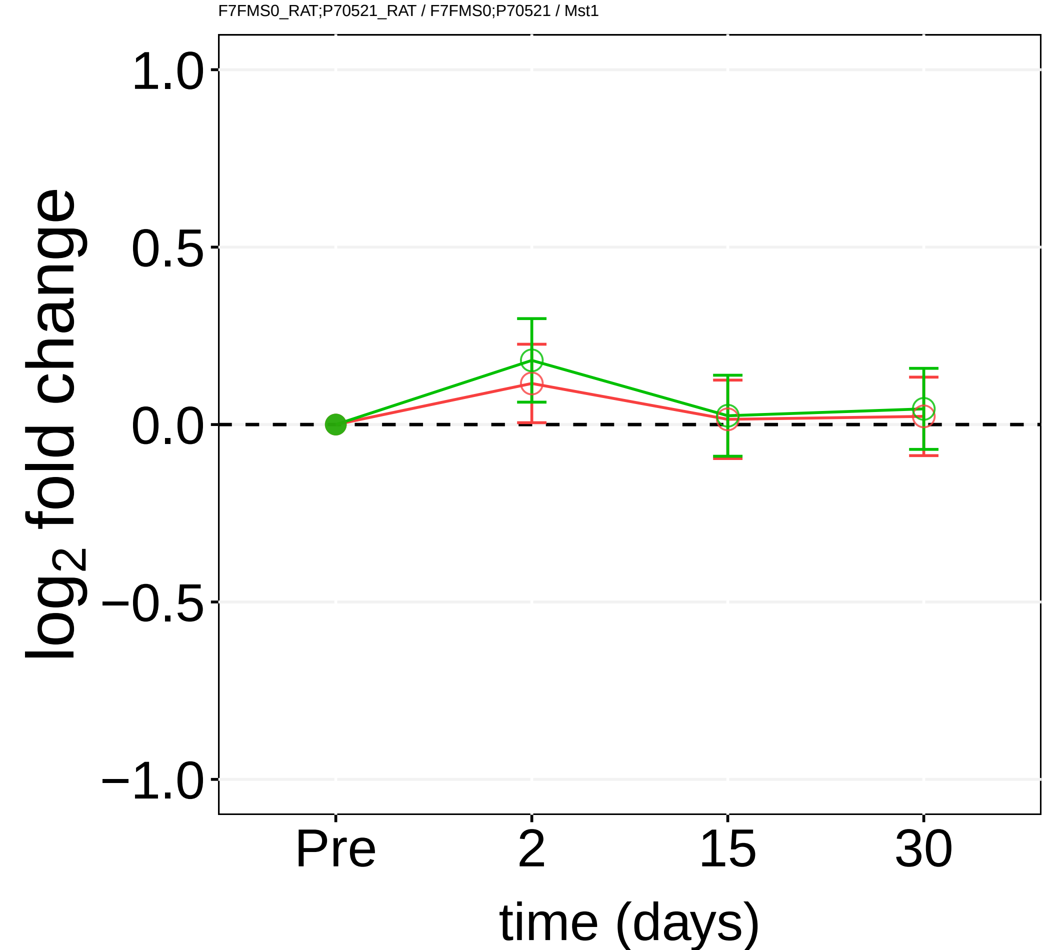

F7FMS0_RAT;P70521_RAT / F7FMS0;P70521 / Mst1
1.0
0.5
0.0
−0.5
−1.0
log2 fold change
Pre
2
15
30
time (days)

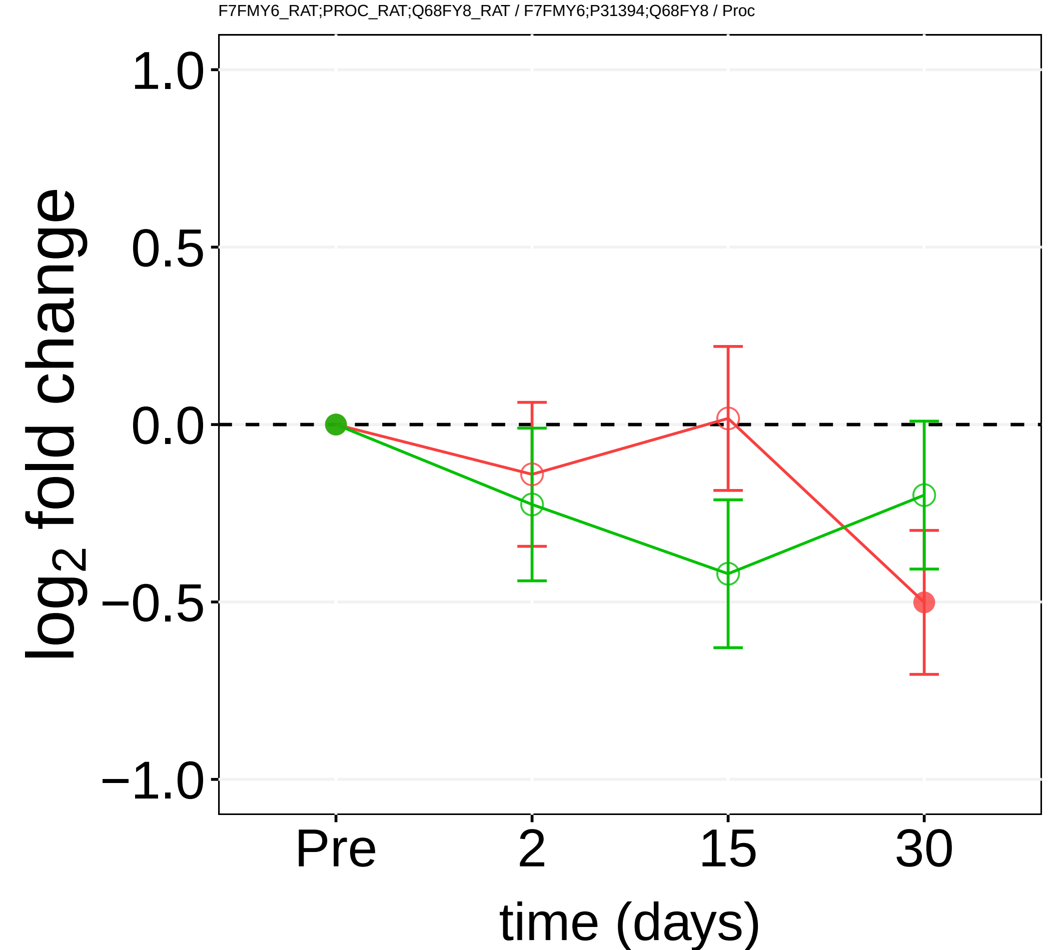
F7FMY6_RAT;PROC_RAT;Q68FY8_RAT / F7FMY6;P31394;Q68FY8 / Proc
1.0
0.5
0.0
−0.5
−1.0
log2 fold change
Pre
2
15
30
time (days)

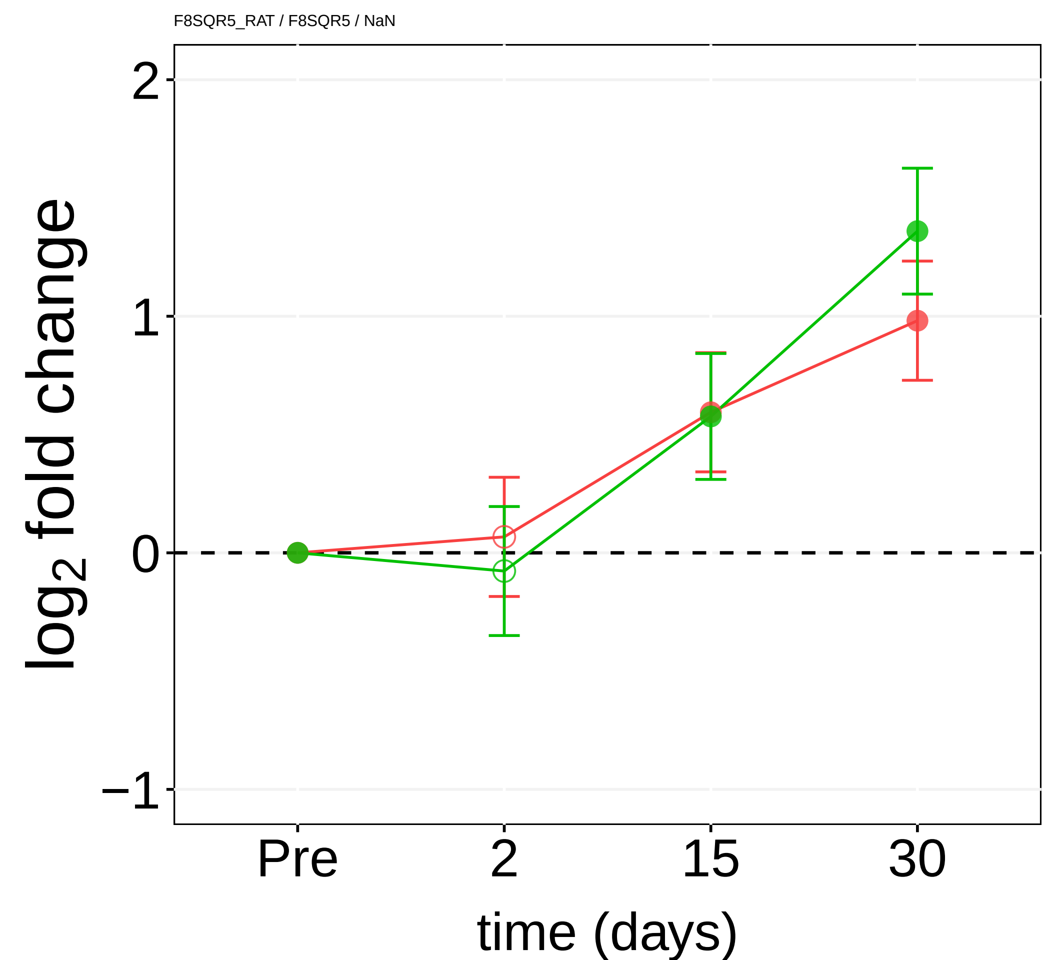

F8SQR5_RAT / F8SQR5 / NaN
log2 fold change
2
1
0
−1
Pre
2
15
30
time (days)

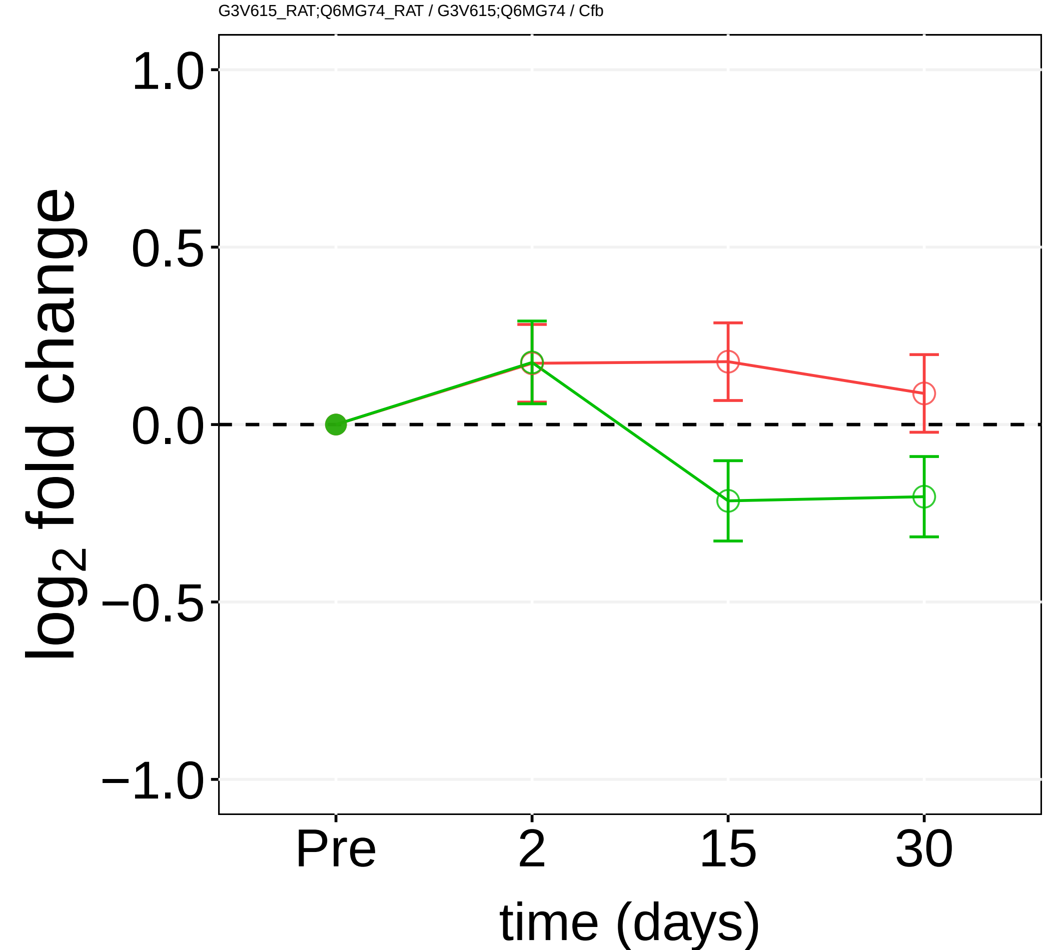
G3V615_RAT;Q6MG74_RAT / G3V615;Q6MG74 / Cfb
1.0
0.5
0.0
−0.5
−1.0
$\log_2$ fold change
Pre
2
15
30
time (days)

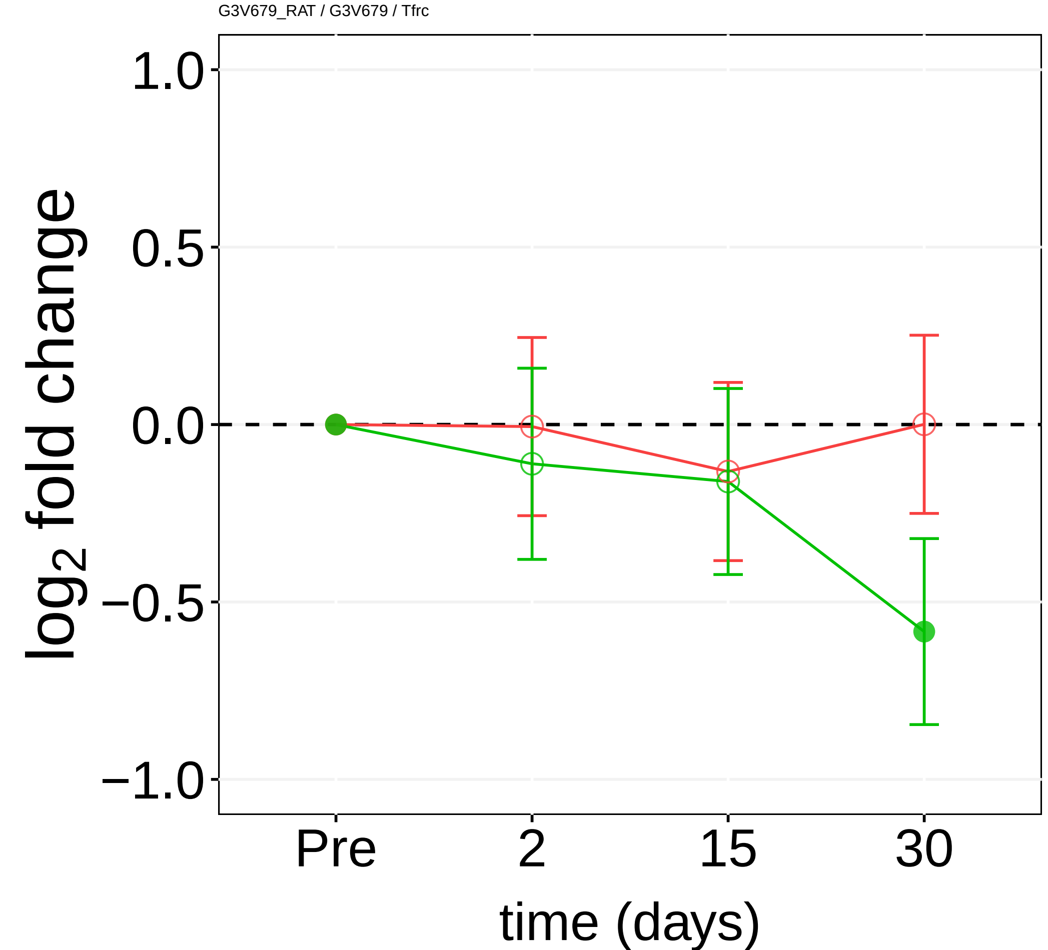
G3V679_RAT / G3V679 / Tfrc
log2 fold change
1.0
0.5
0.0
−0.5
−1.0
Pre
2
15
30
time (days)

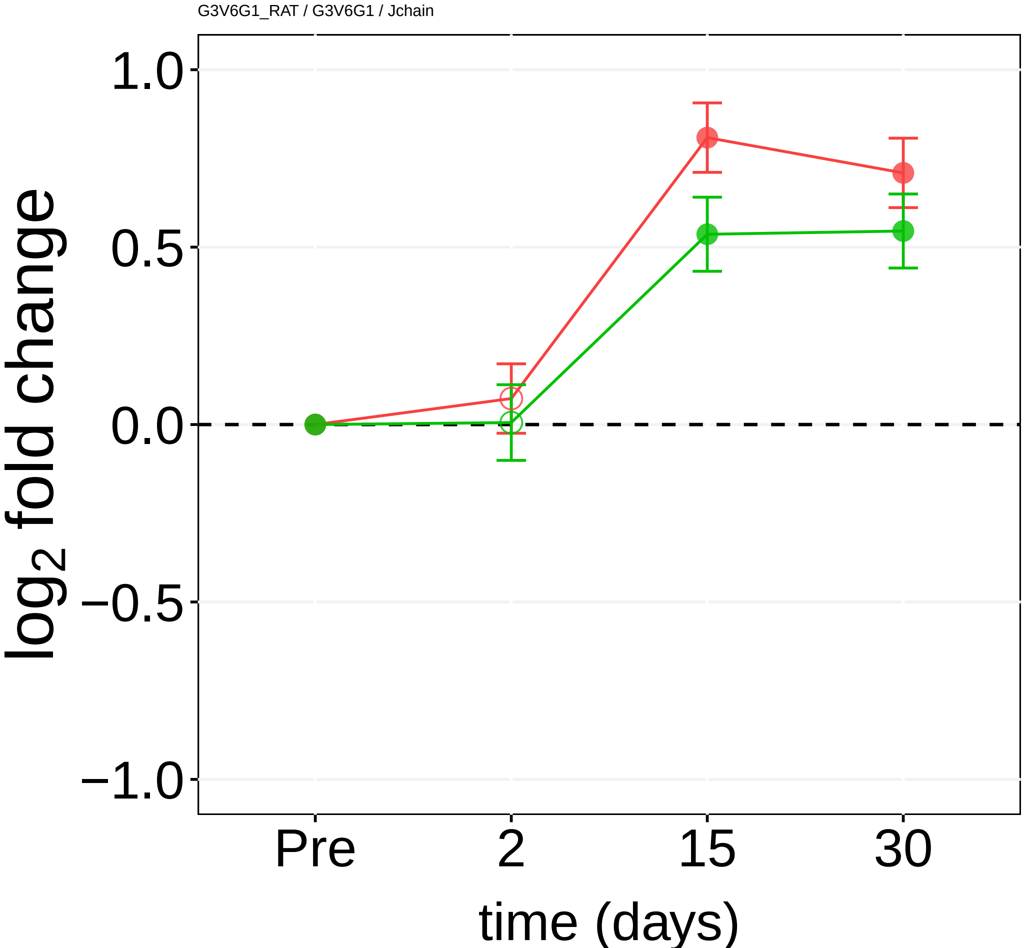
G3V6G1_RAT / G3V6G1 / Jchain
1.0
0.5
0.0
−0.5
−1.0
log2 fold change
Pre
2
15
30
time (days)

G3V7L3_RAT / G3V7L3 / C1s

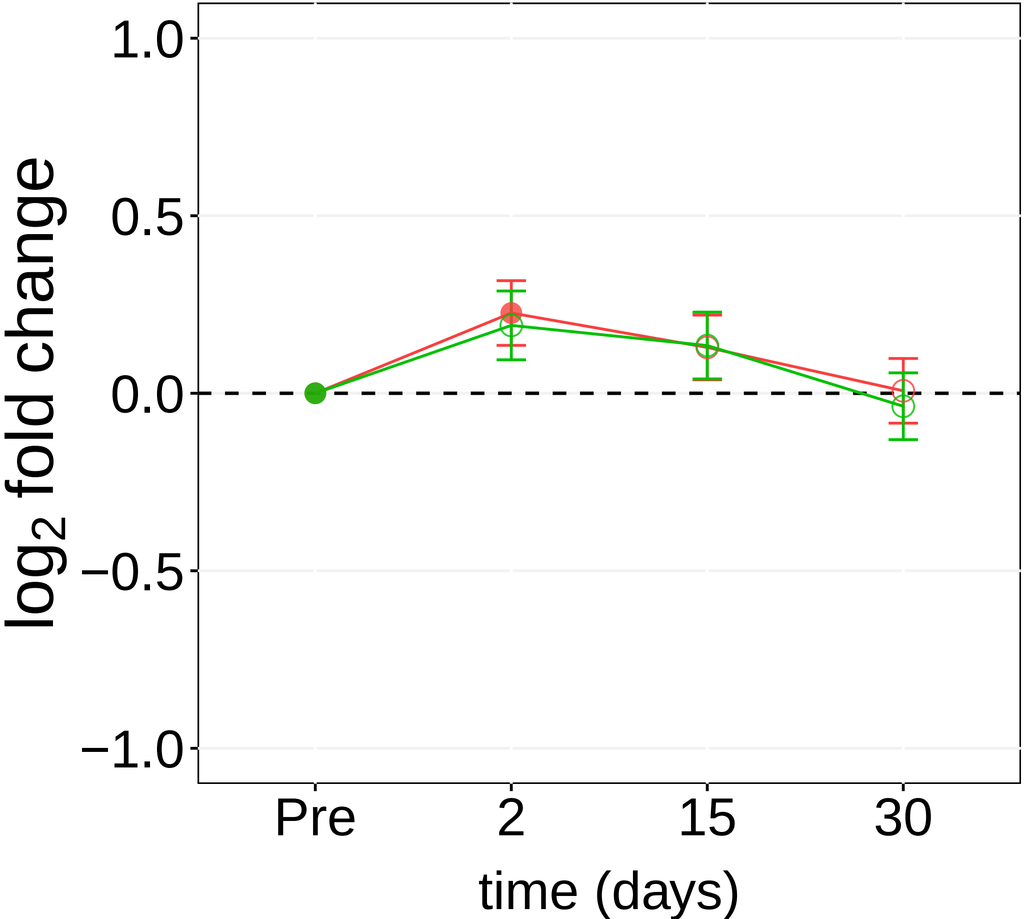

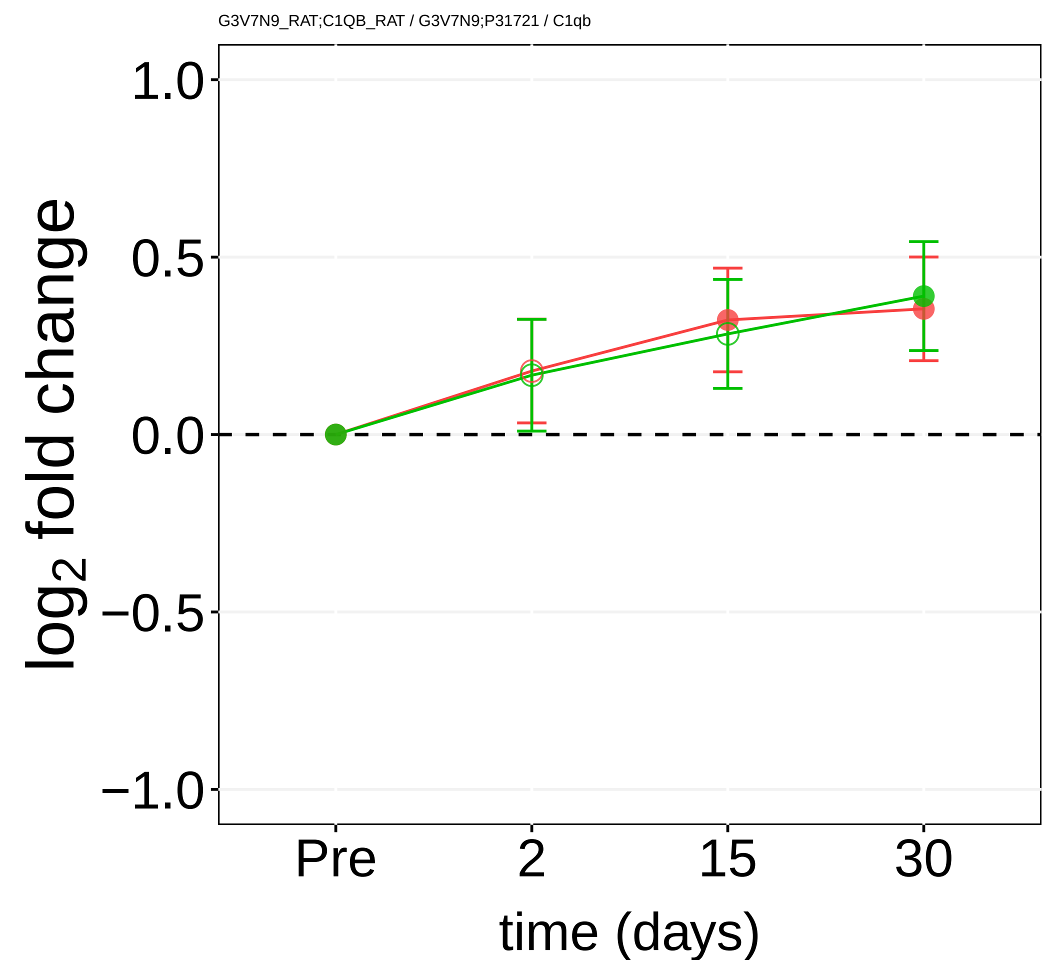
G3V7N9_RAT;C1QB_RAT / G3V7N9;P31721 / C1qb
1.0
0.5
0.0
−0.5
−1.0
$\log_2$ fold change
Pre
2
15
30
time (days)

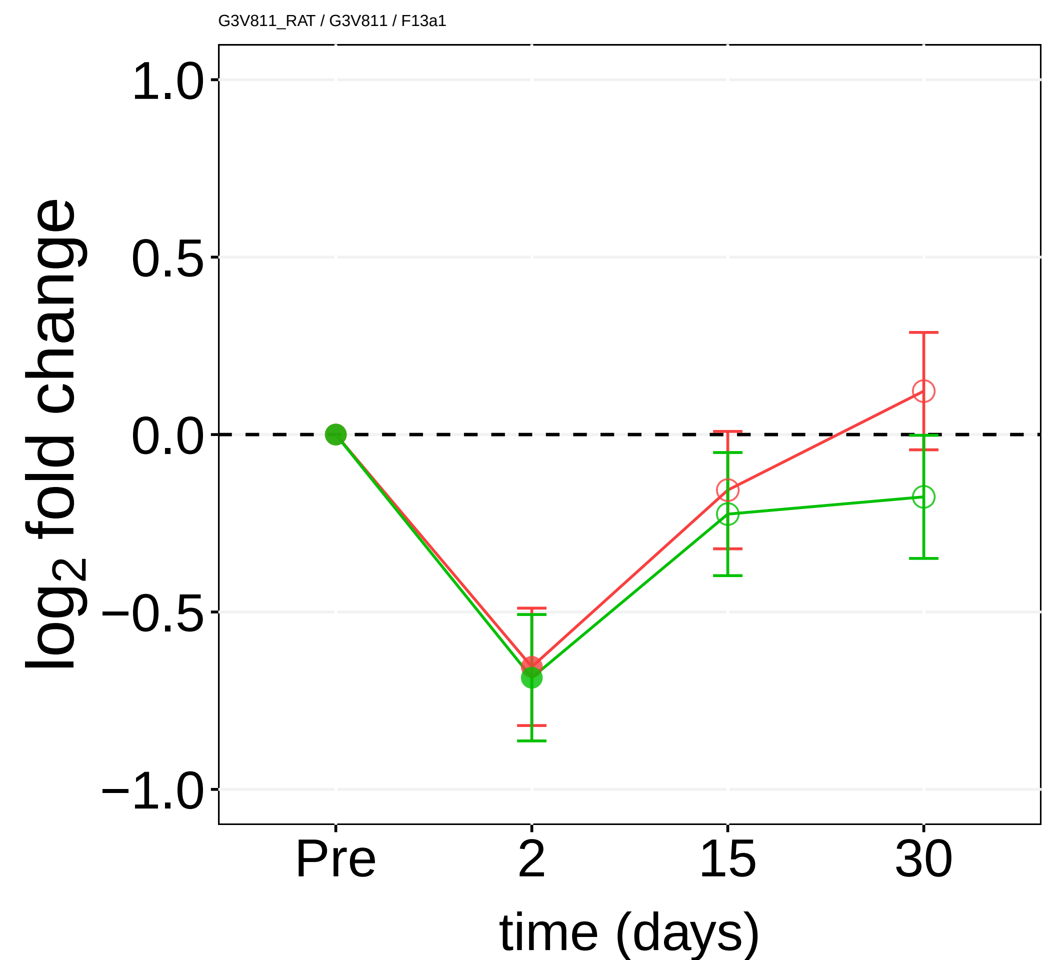
G3V811_RAT / G3V811 / F13a1
1.0
0.5
0.0
−0.5
−1.0
log2 fold change
Pre
2
15
30
time (days)

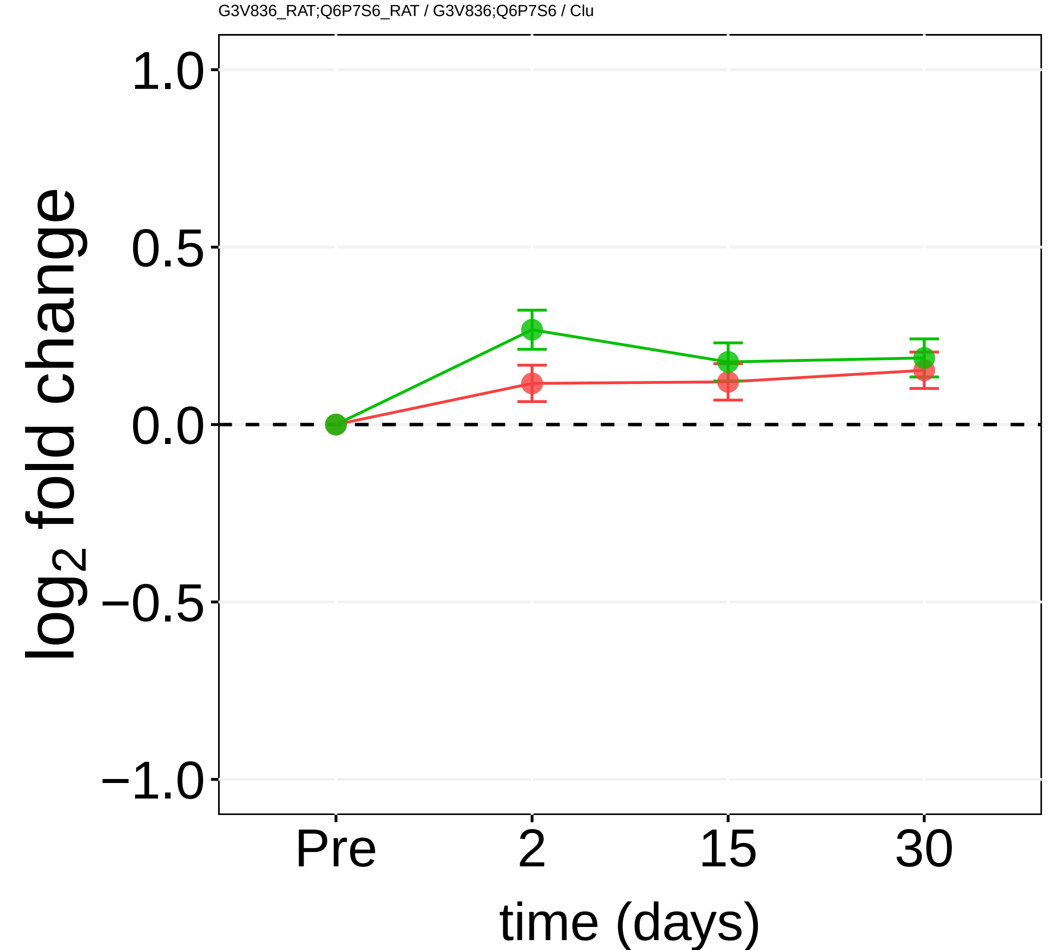
G3V836_RAT;Q6P7S6_RAT / G3V836;Q6P7S6 / Clu
1.0
0.5
0.0
−0.5
−1.0
$\log_2$ fold change
Pre
2
15
30
time (days)

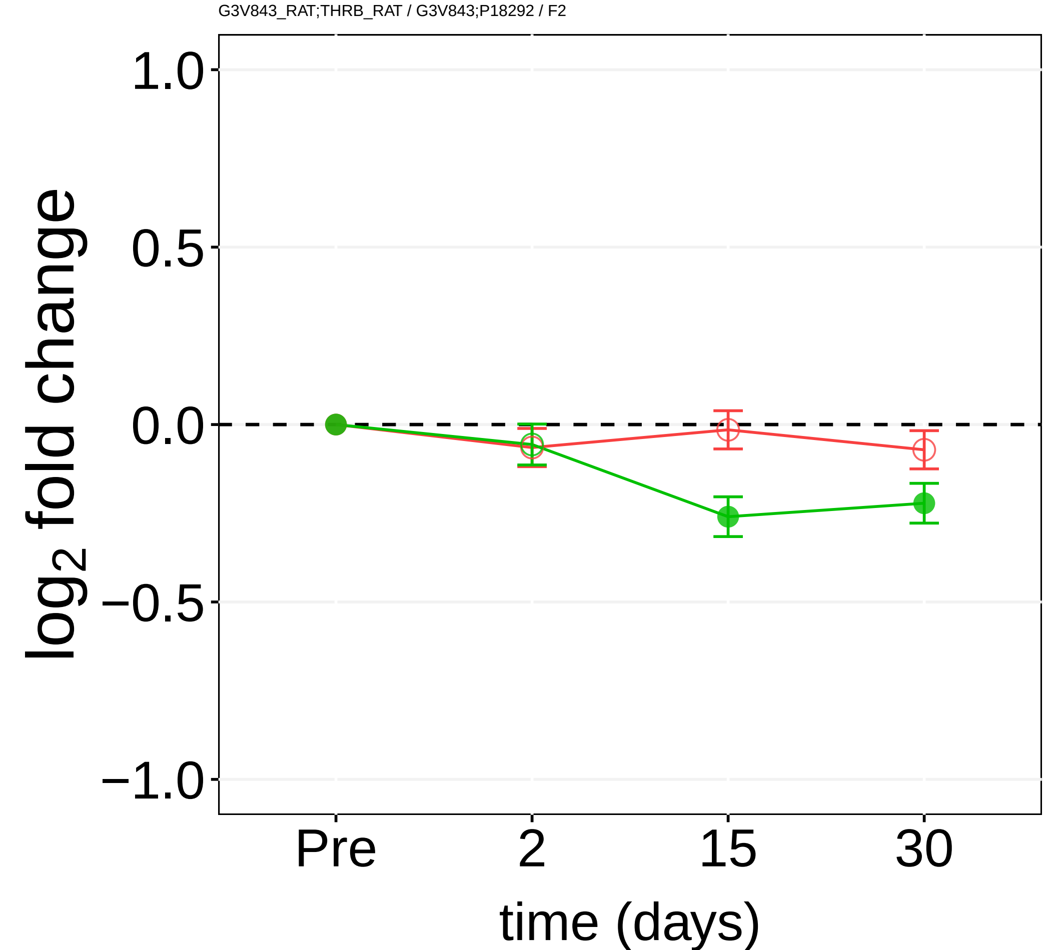
G3V843_RAT;THRB_RAT / G3V843;P18292 / F2
log2 fold change
1.0
0.5
0.0
−0.5
−1.0
Pre
2
15
30
time (days)

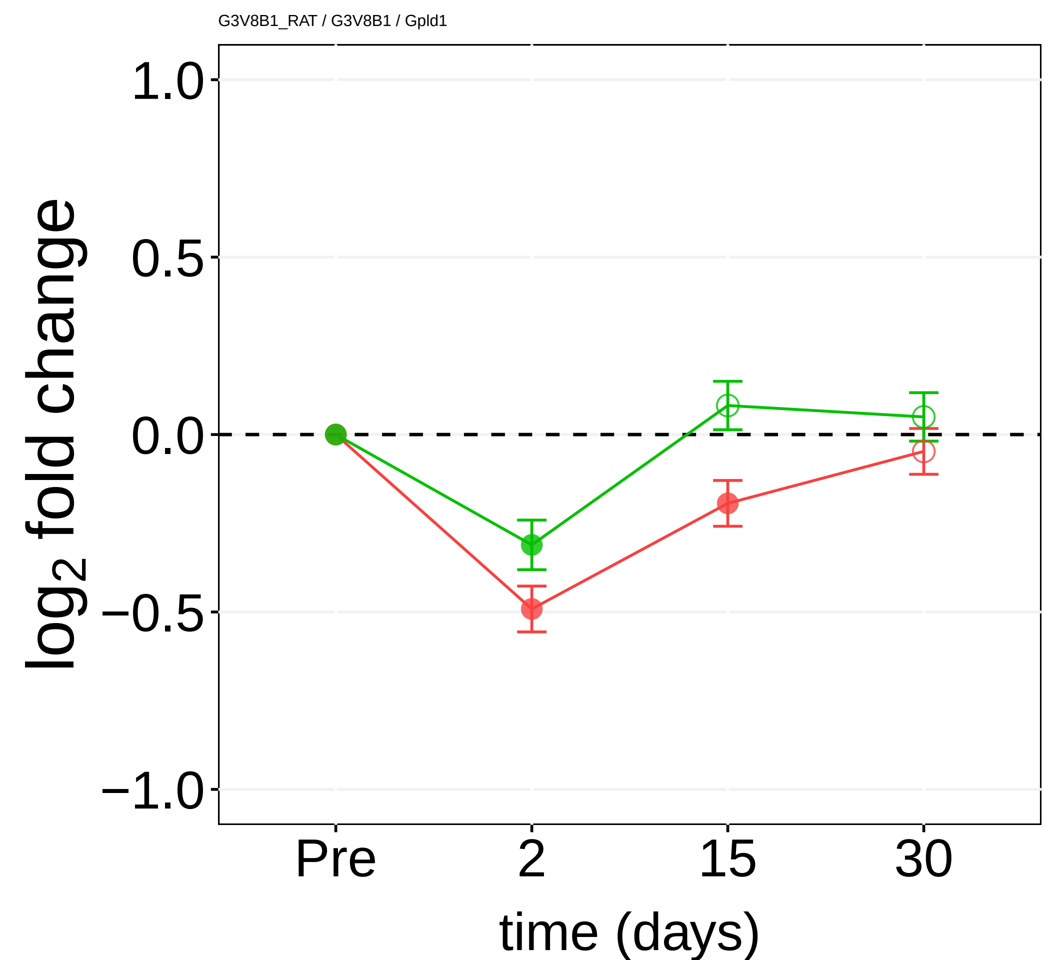
G3V8B1_RAT / G3V8B1 / Gpld1
1.0
0.5
0.0
−0.5
−1.0
log2 fold change
Pre
2
15
30
time (days)

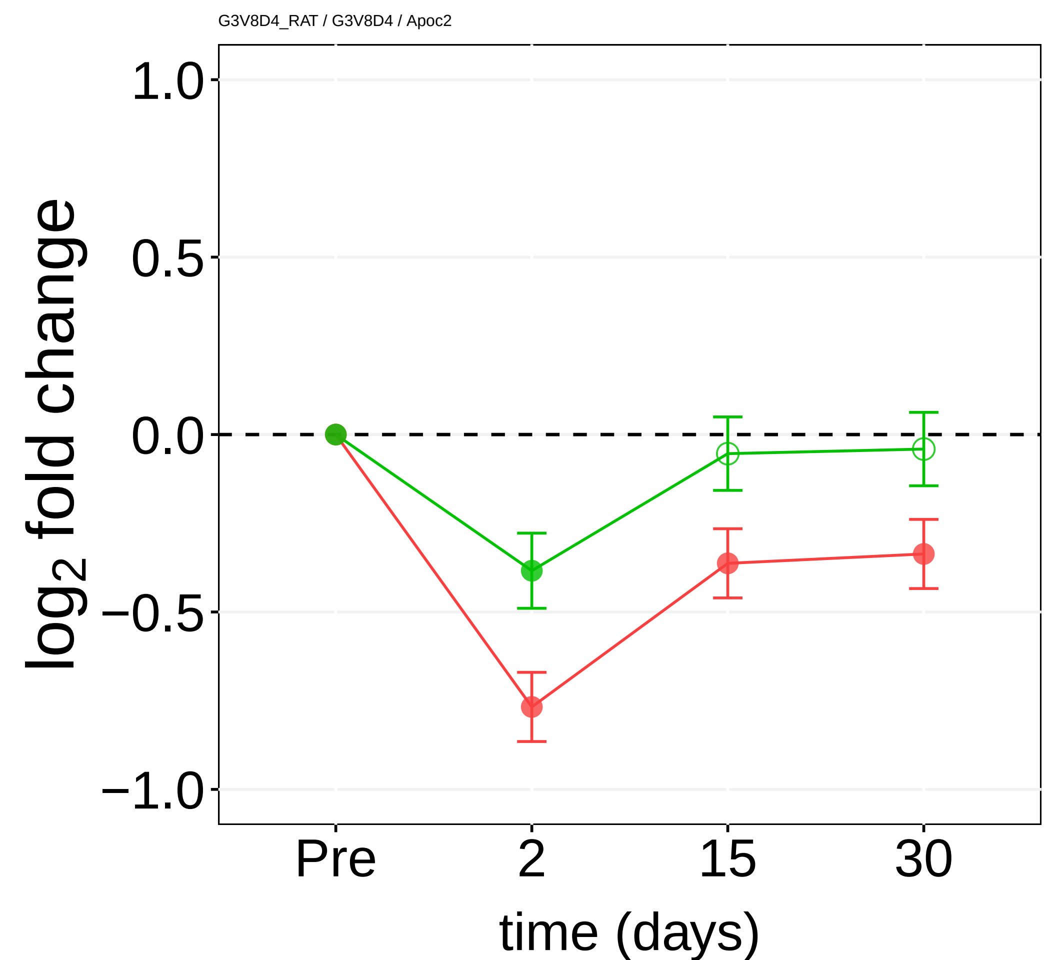
G3V8D4_RAT / G3V8D4 / Apoc2
1.0
0.5
0.0
−0.5
−1.0
log2 fold change
Pre
2
15
30
time (days)

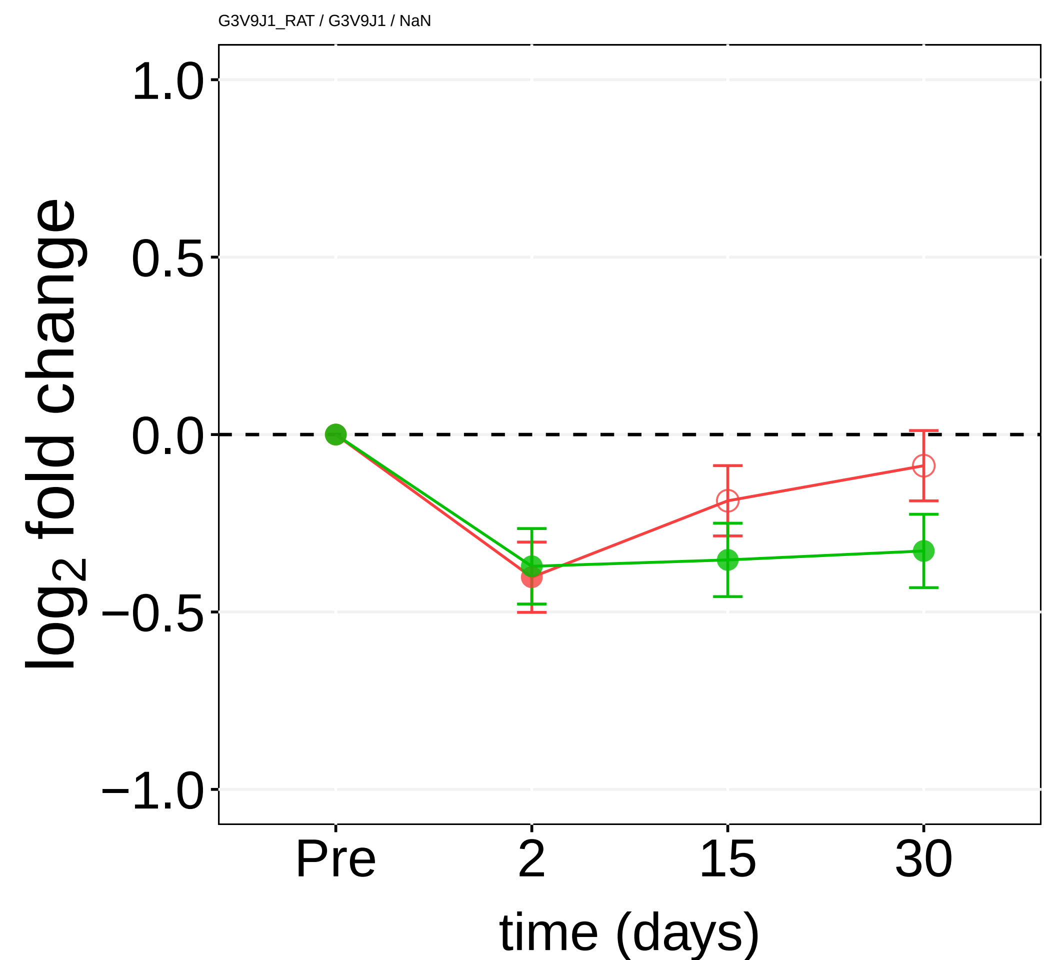

G3V9J1_RAT / G3V9J1 / NaN
1.0
0.5
0.0
−0.5
−1.0
log2 fold change
Pre
2
15
30
time (days)

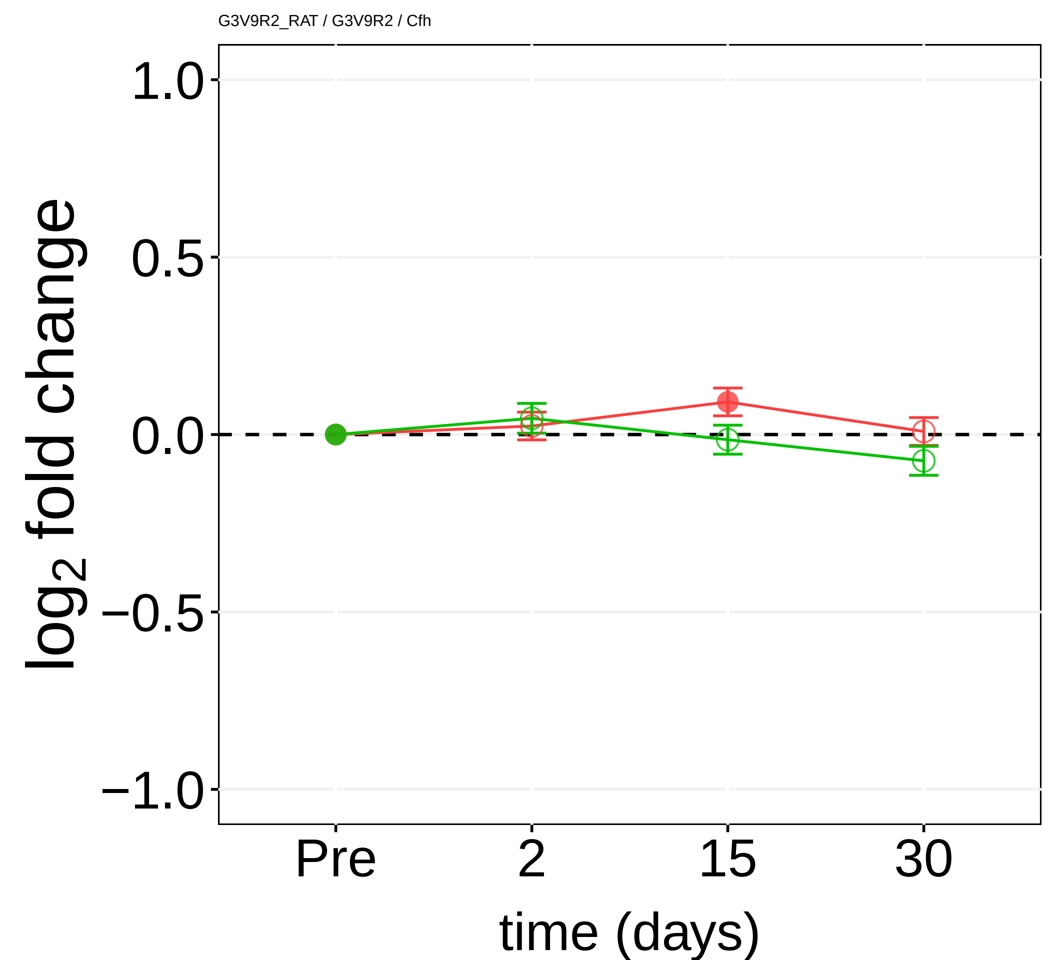
G3V9R2_RAT / G3V9R2 / Cfh
1.0
0.5
0.0
−0.5
−1.0
log2 fold change
Pre
2
15
30
time (days)

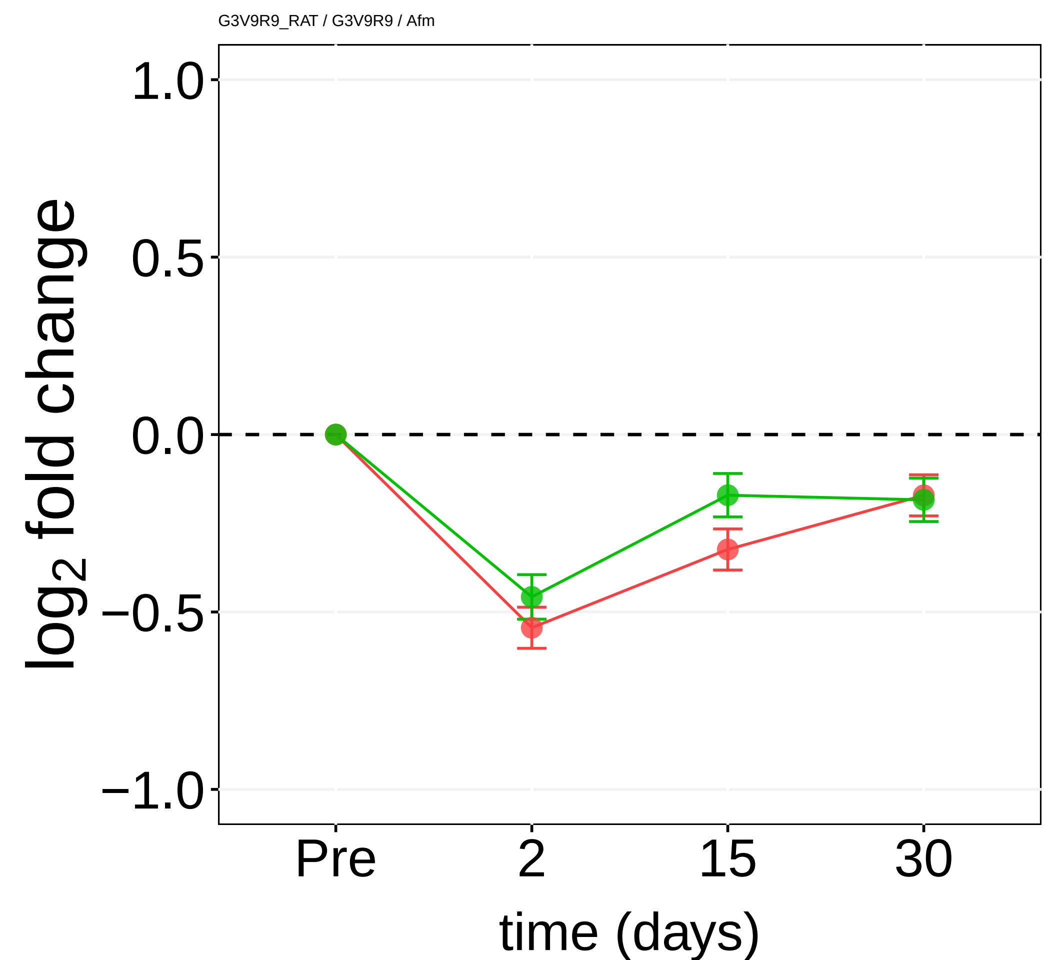
G3V9R9_RAT / G3V9R9 / Afm
1.0
0.5
0.0
−0.5
−1.0
log2 fold change
Pre
2
15
30
time (days)

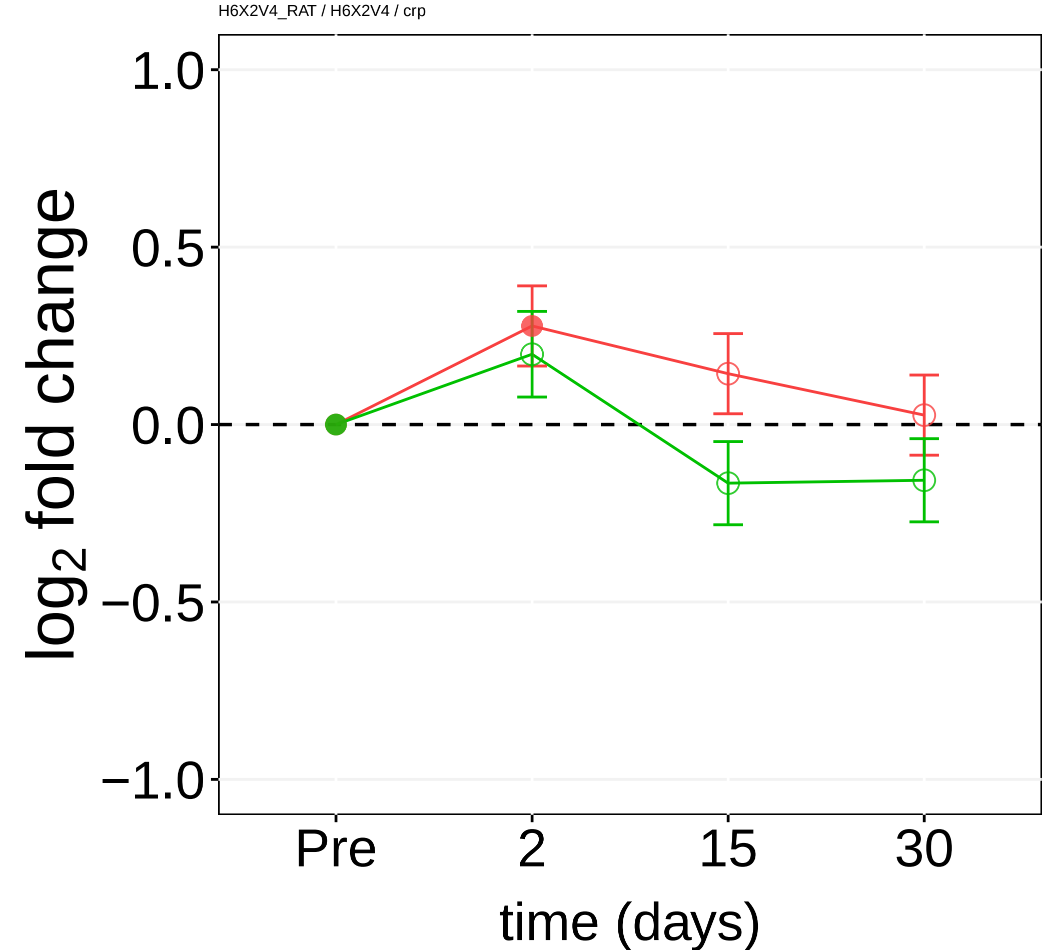
H6X2V4_RAT / H6X2V4 / crp
1.0
0.5
0.0
−0.5
−1.0
log2 fold change
Pre
2
15
30
time (days)

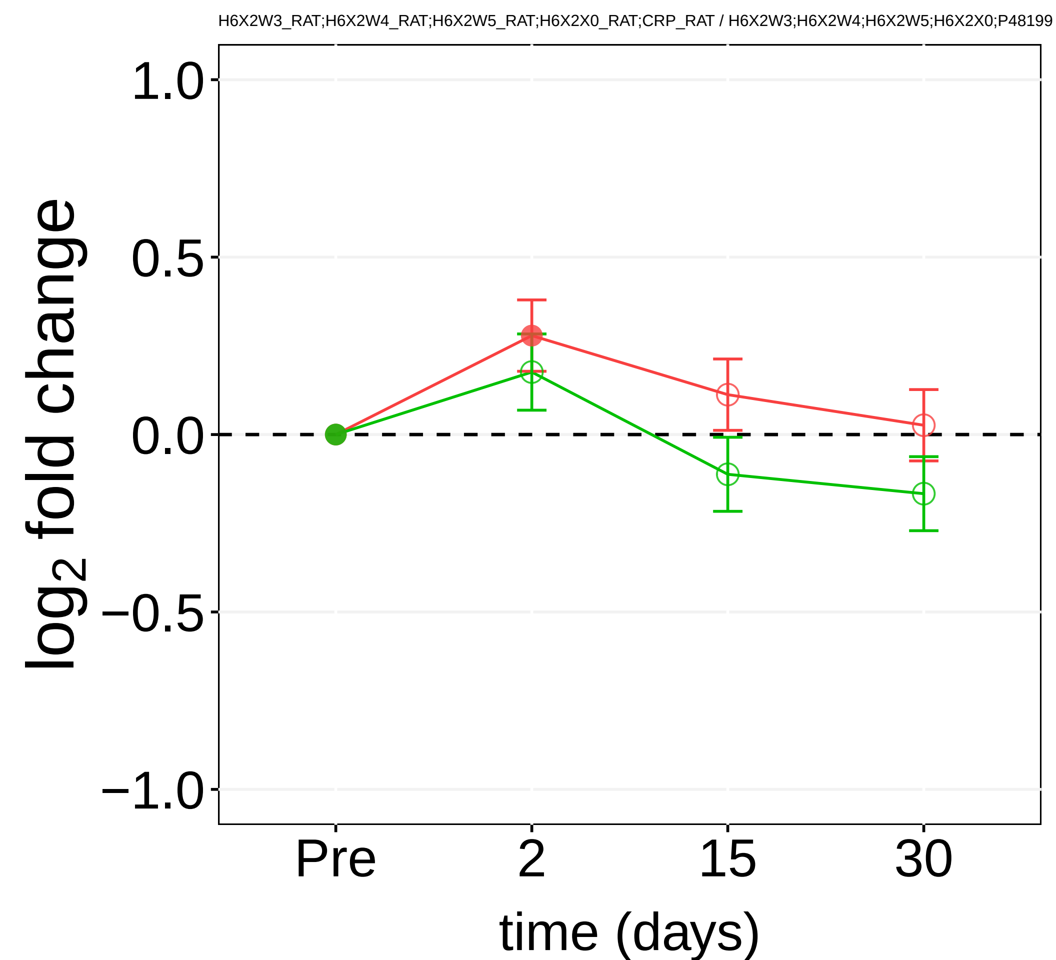

H6X2W3_RAT;H6X2W4_RAT;H6X2W5_RAT;H6X2X0_RAT;CRP_RAT / H6X2W3;H6X2W4;H6X2W5;H6X2X0;P48199
1.0
0.5
0.0
−0.5
−1.0
log2 fold change
Pre
2
15
30
time (days)

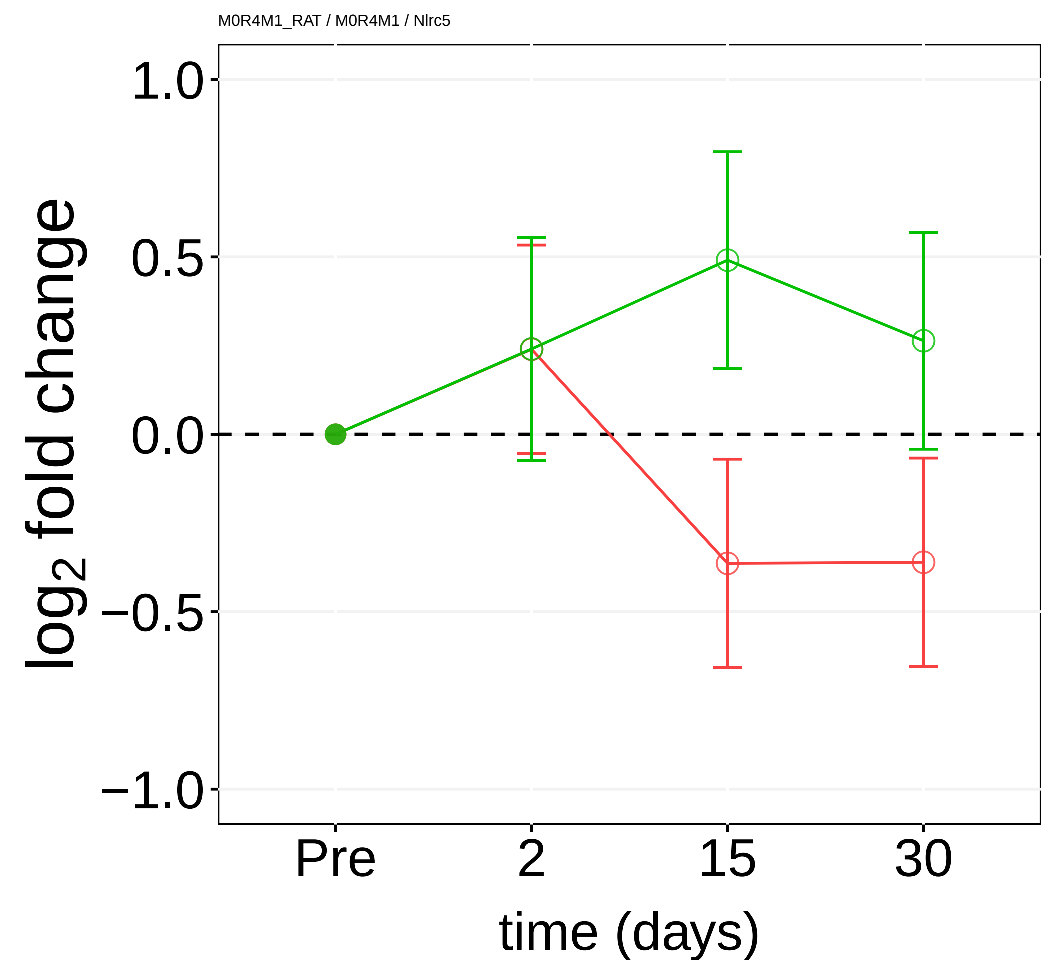
M0R4M1_RAT / M0R4M1 / Nlrc5
log2 fold change
1.0
0.5
0.0
−0.5
−1.0
Pre
2
15
30
time (days)

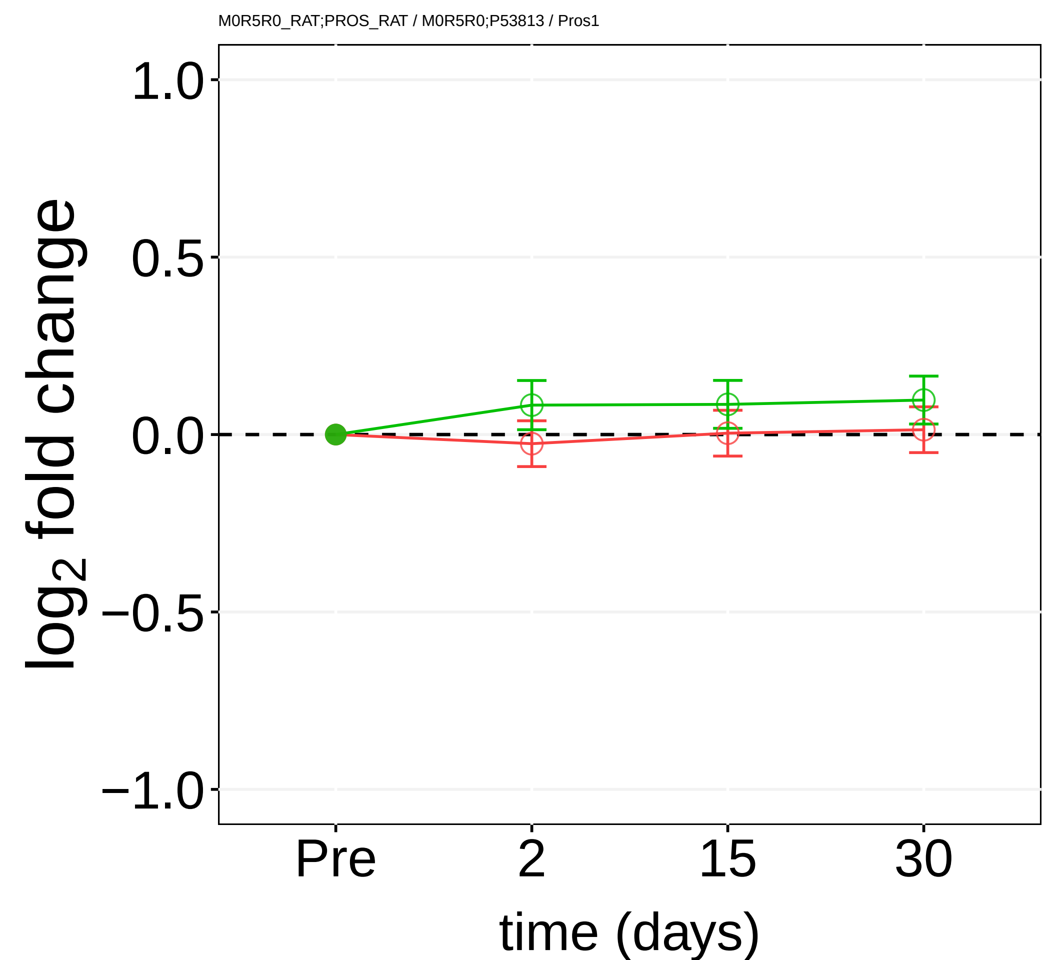

M0R5R0_RAT;PROS_RAT / M0R5R0;P53813 / Pros1
1.0
0.5
0.0
−0.5
−1.0
log2 fold change
Pre
2
15
30
time (days)

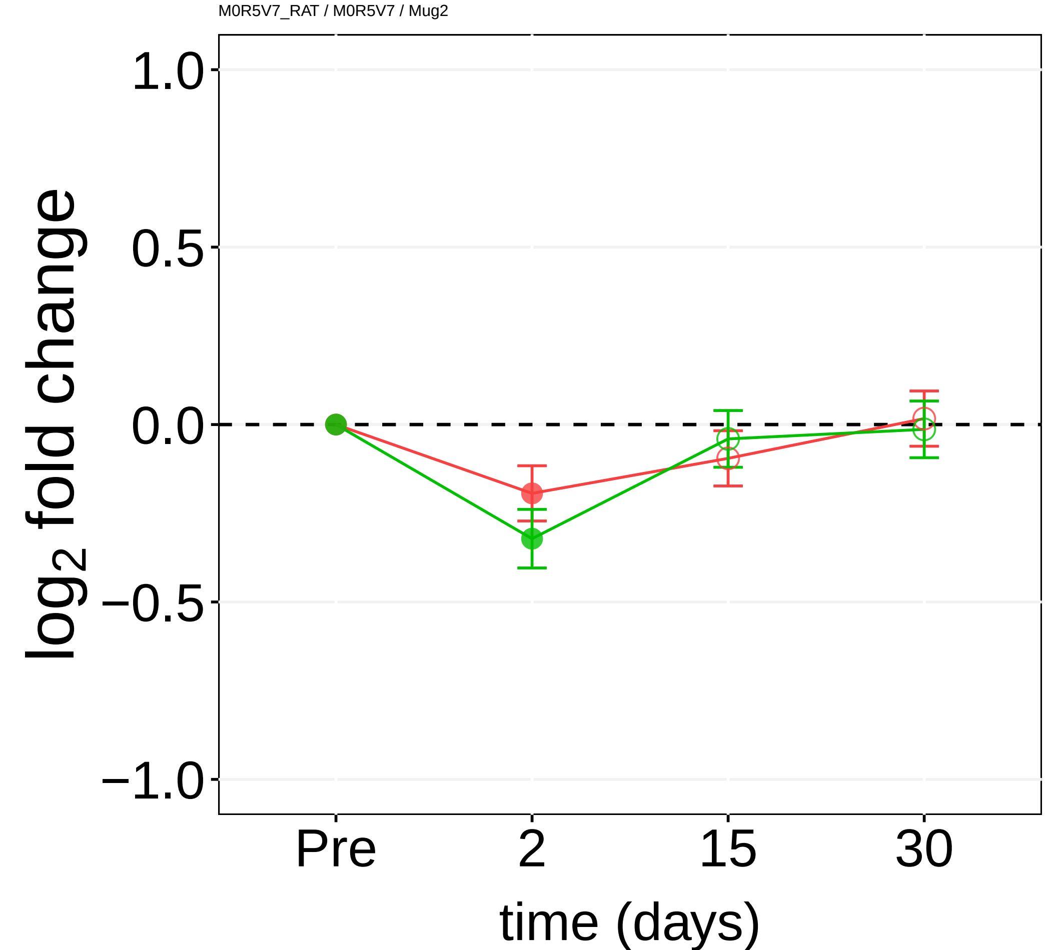

M0R5V7_RAT / M0R5V7 / Mug2
1.0
0.5
0.0
−0.5
−1.0
log2 fold change
Pre
2
15
30
time (days)

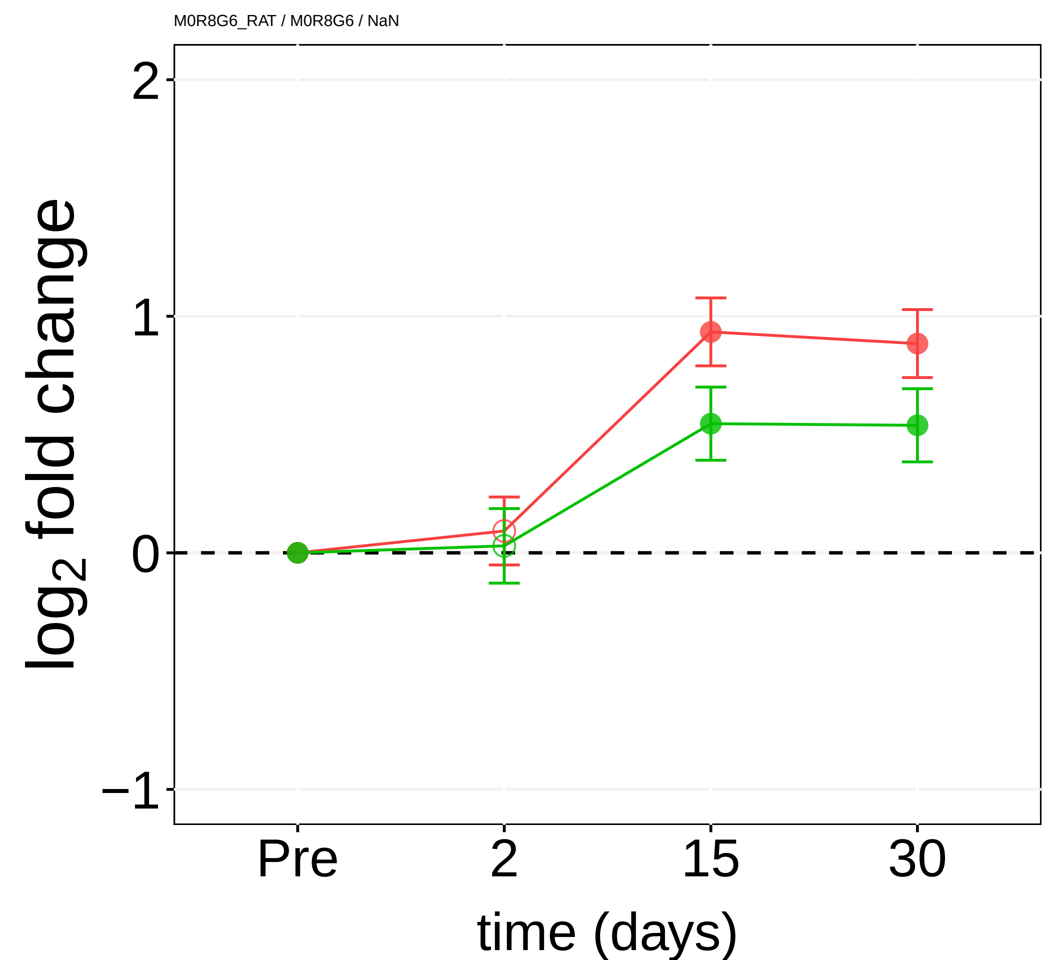

M0R8G6_RAT / M0R8G6 / NaN
2
1
0
−1
log2 fold change
Pre
2
15
30
time (days)

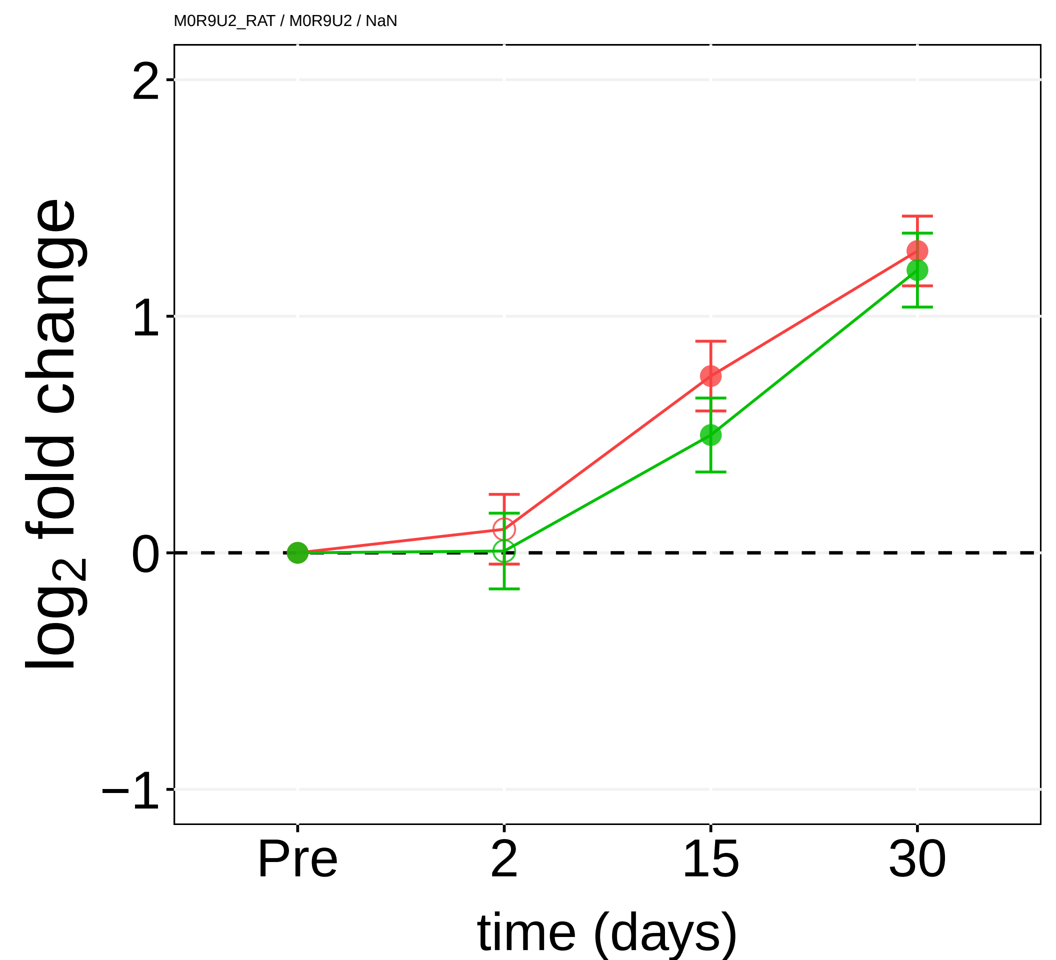

M0R9U2_RAT / M0R9U2 / NaN
$\log_2$ fold change
2
1
0
−1
Pre
2
15
30
time (days)

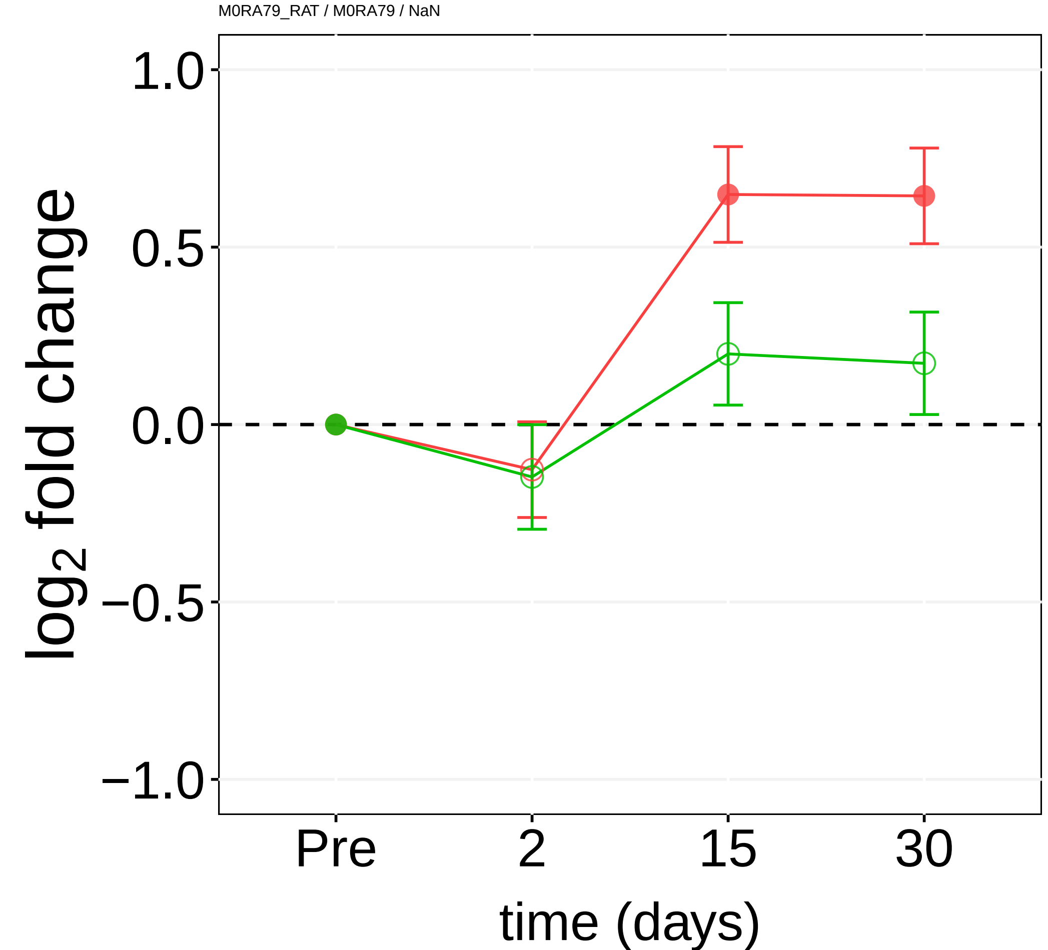
M0RA79_RAT / M0RA79 / NaN
1.0
0.5
0.0
−0.5
−1.0
log2 fold change
Pre
2
15
30
time (days)

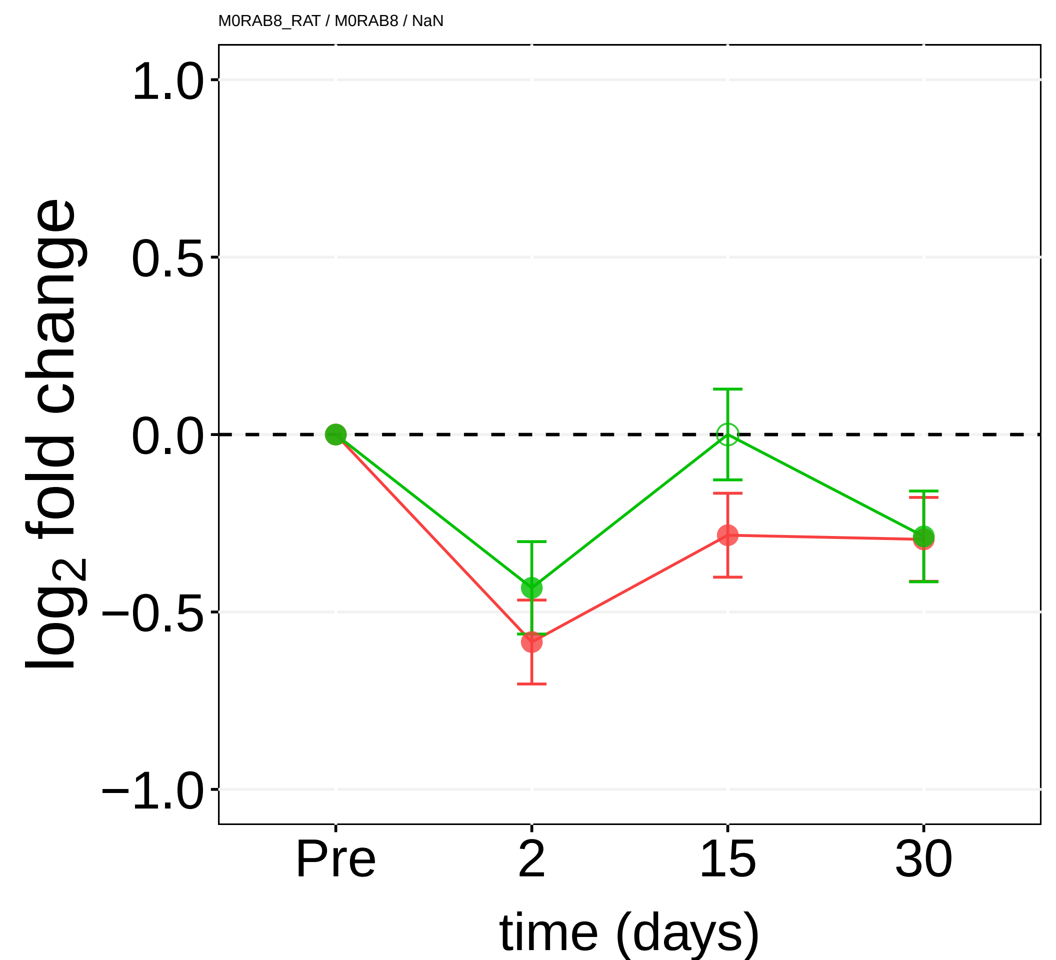

M0RAB8_RAT / M0RAB8 / NaN
1.0
0.5
0.0
−0.5
−1.0
log2 fold change
Pre
2
15
30
time (days)

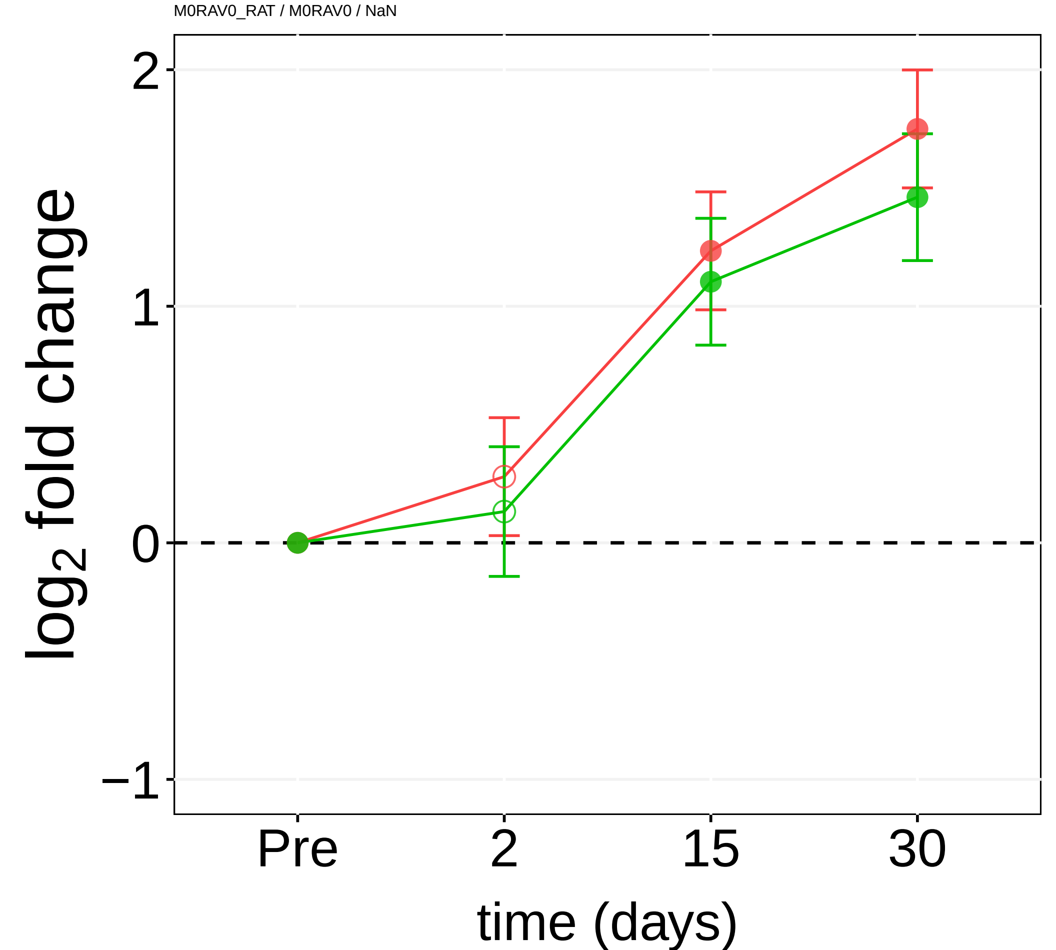
M0RAV0_RAT / M0RAV0 / NaN
log2 fold change
2
1
0
−1
Pre
2
15
30
time (days)

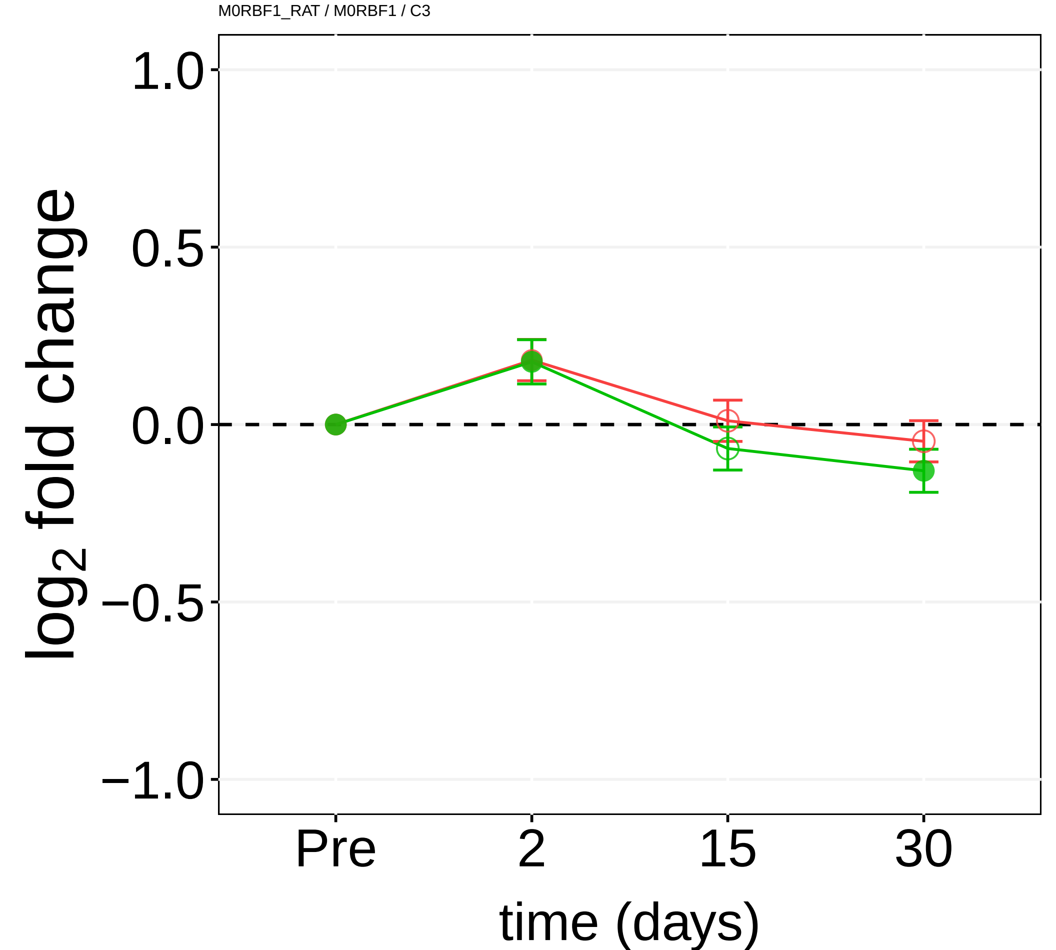
M0RBF1_RAT / M0RBF1 / C3
1.0
0.5
0.0
−0.5
−1.0
log2 fold change
Pre
2
15
30
time (days)

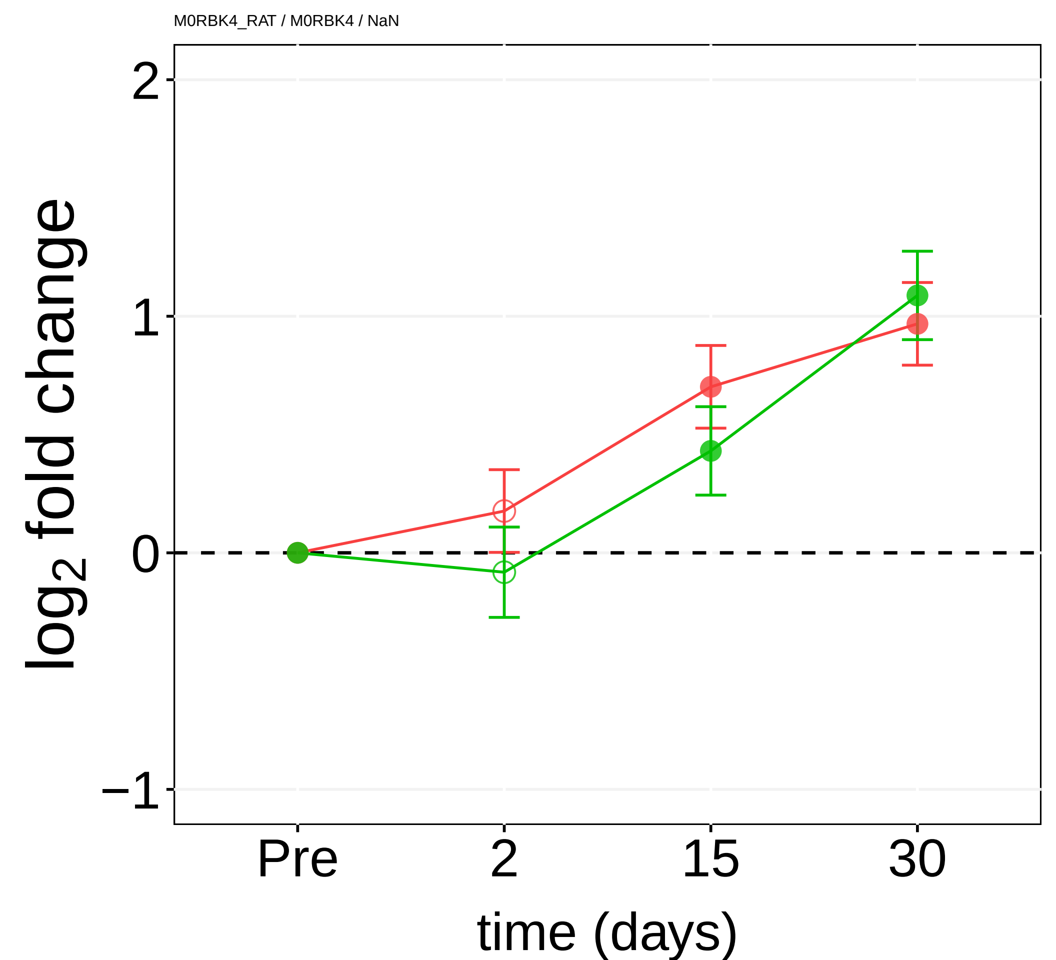
M0RBK4_RAT / M0RBK4 / NaN
2
1
0
−1
log2 fold change
Pre
2
15
30
time (days)

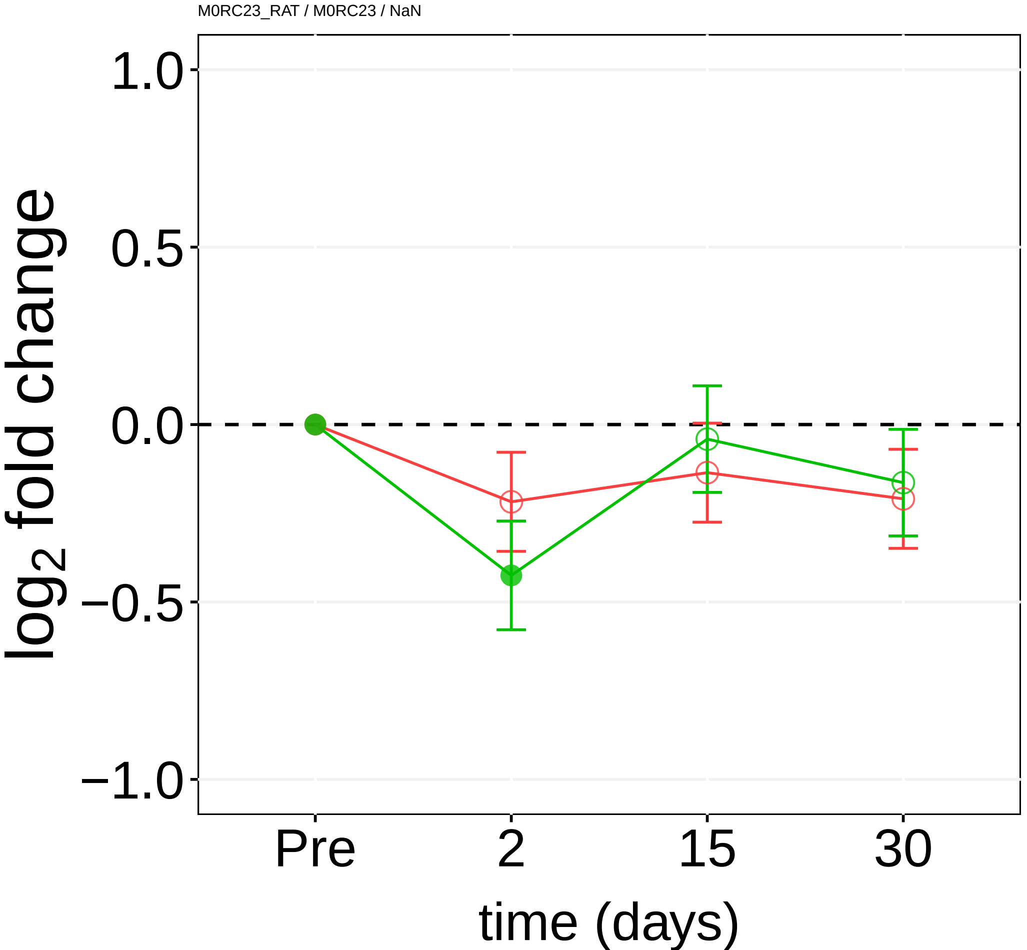
M0RC23_RAT / M0RC23 / NaN
1.0
0.5
0.0
−0.5
−1.0
log2 fold change
Pre
2
15
30
time (days)

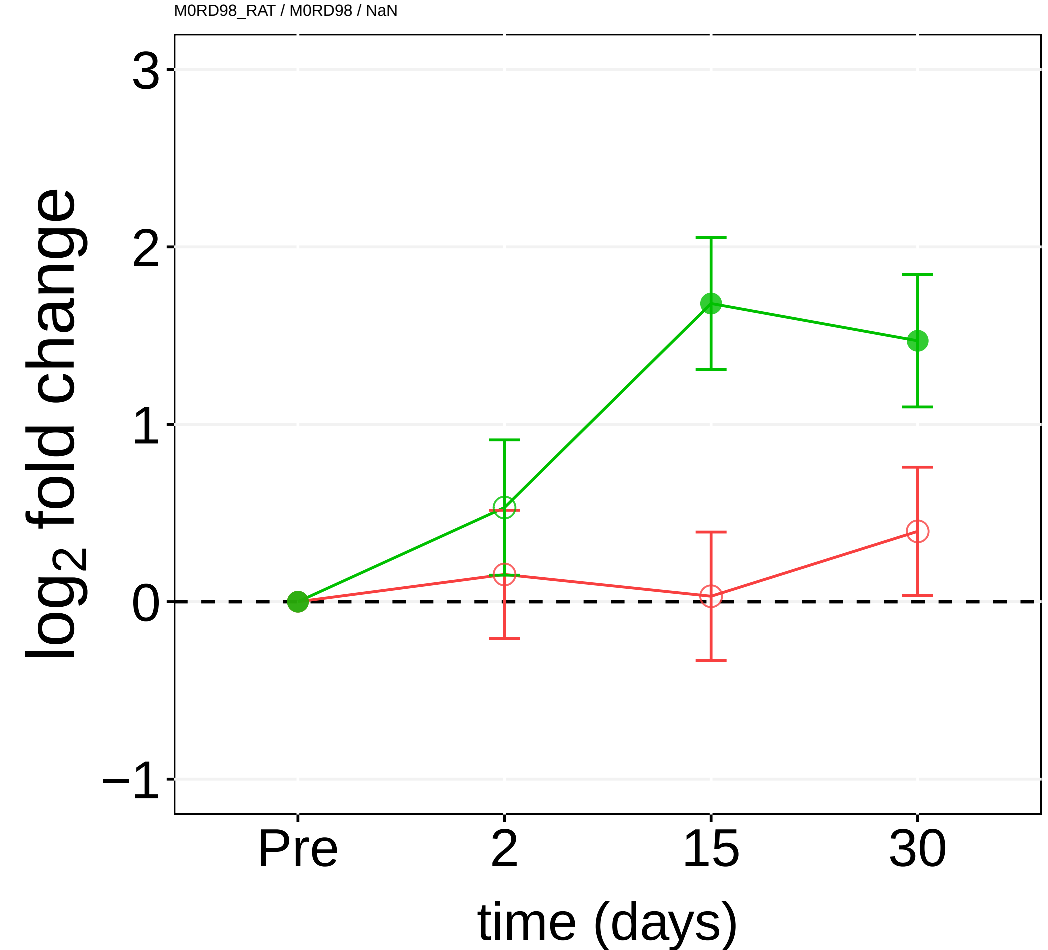

M0RD98_RAT / M0RD98 / NaN
log2 fold change
3
2
1
0
−1
Pre
2
15
30
time (days)

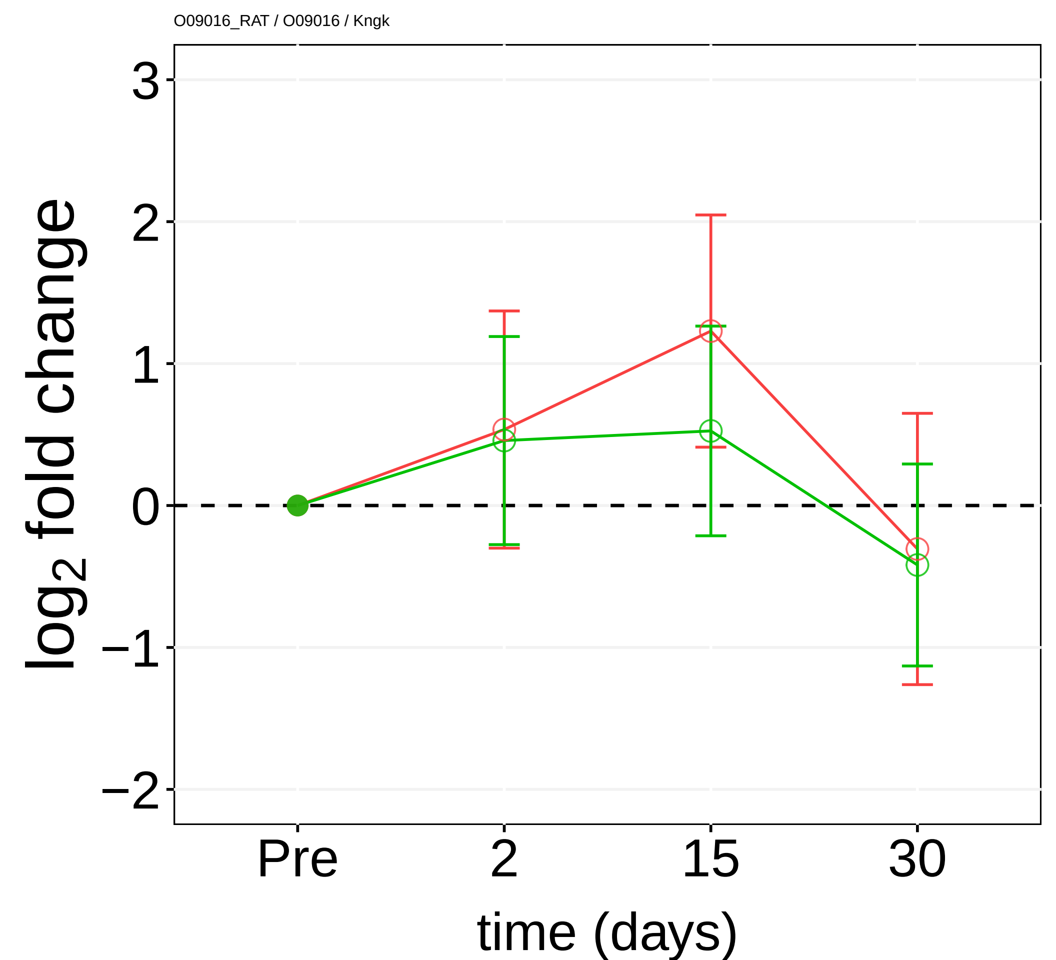
O09016_RAT / O09016 / Kngk
3
2
1
0
−1
−2
log2 fold change
Pre
2
15
30
time (days)

O35849_RAT / O35849 / Lcat

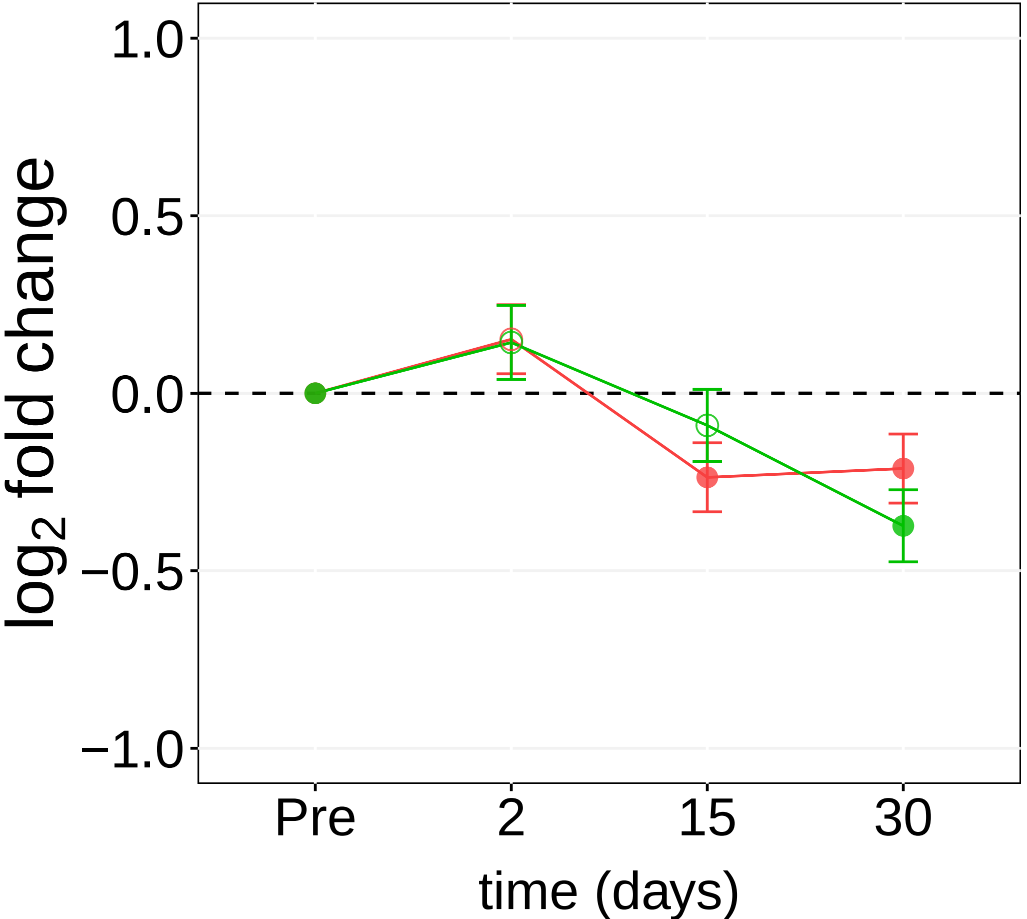

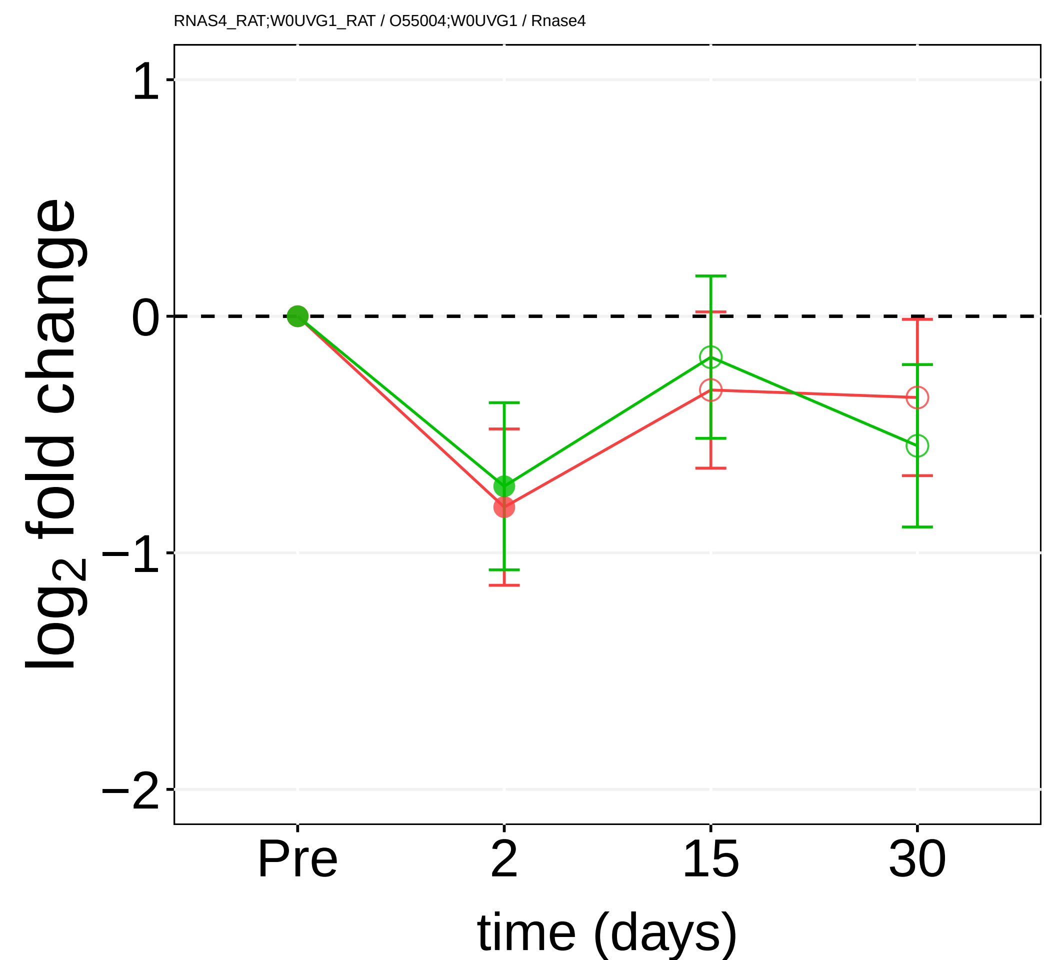
RNAS4_RAT;W0UVG1_RAT / O55004;W0UVG1 / Rnase4
1
0
−1
−2
log2 fold change
Pre
2
15
30
time (days)

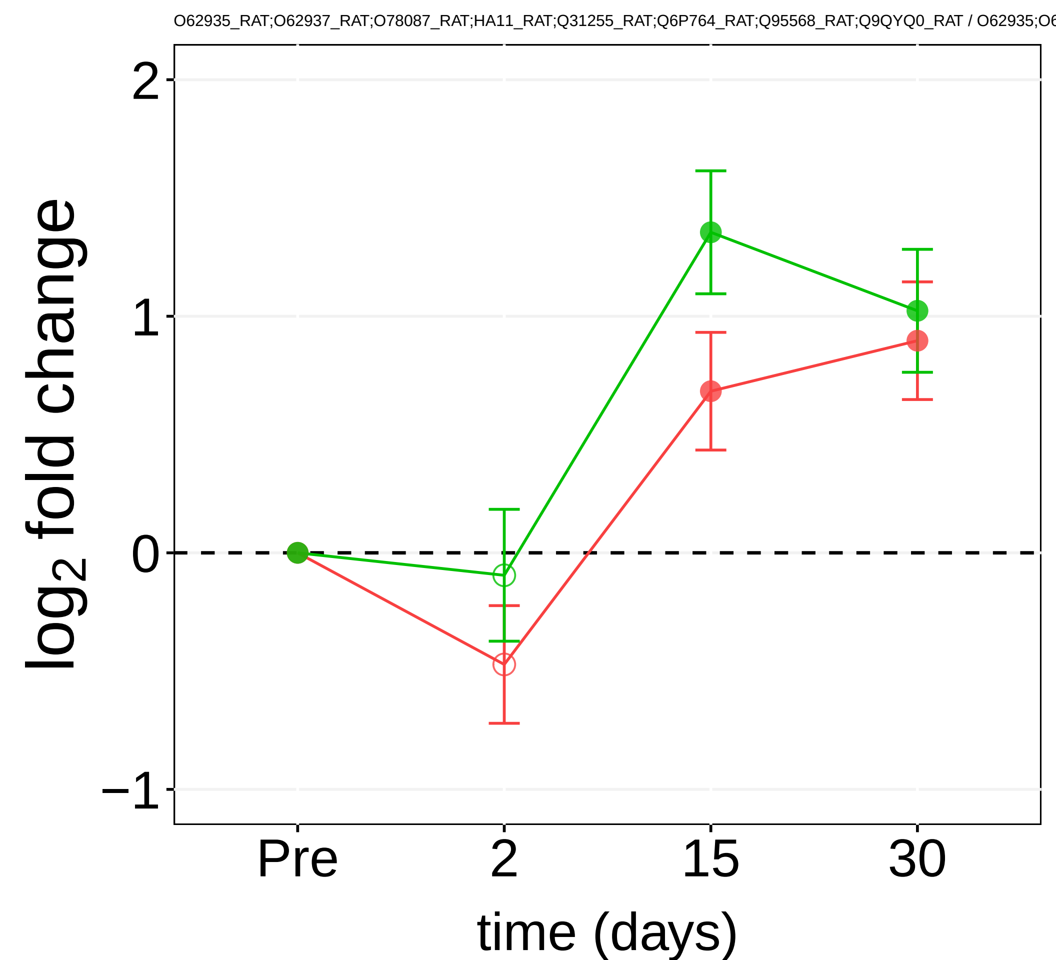

O62935_RAT;O62937_RAT;O78087_RAT;HA11_RAT;Q31255_RAT;Q6P764_RAT;Q95568_RAT;Q9QYQ0_RAT / O62935;O6
2
1
0
−1
Pre
2
15
30
time (days)
log2 fold change

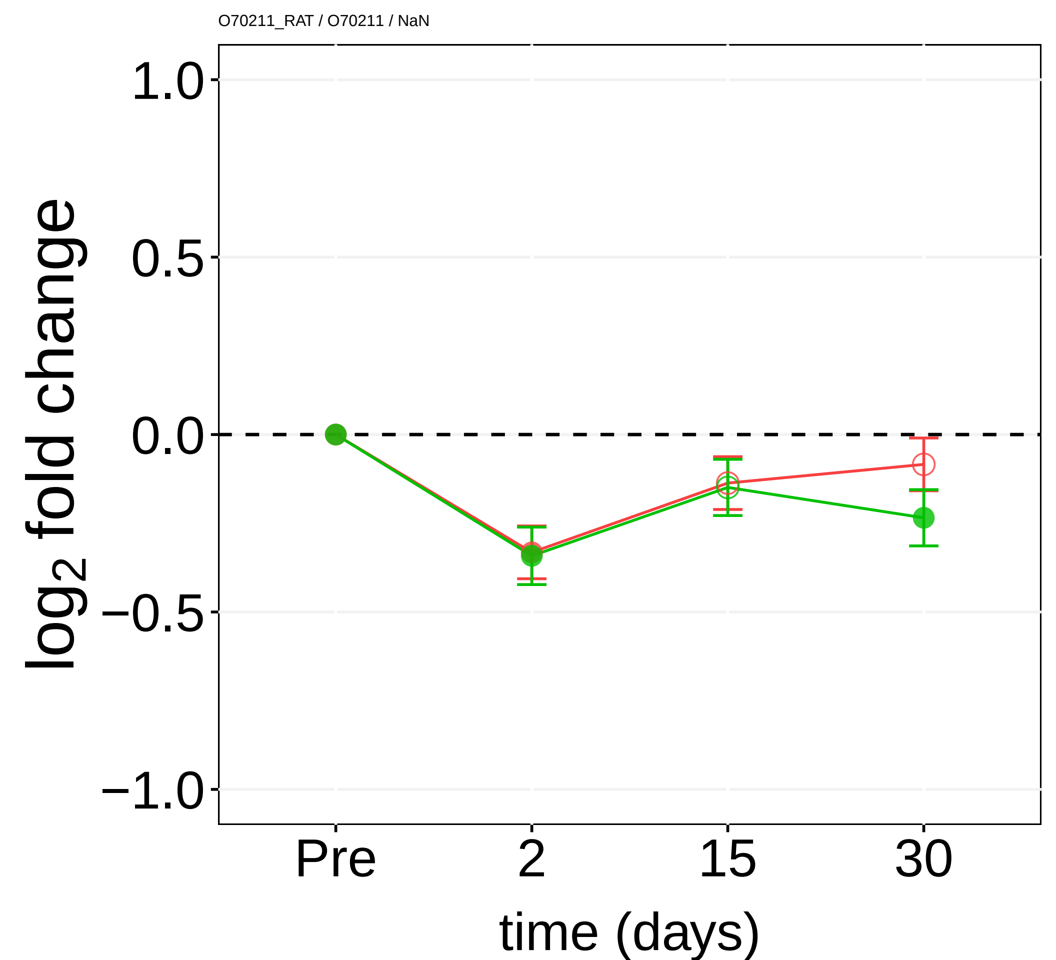
O70211_RAT / O70211 / NaN
1.0
0.5
0.0
−0.5
−1.0
log2 fold change
Pre
2
15
30
time (days)

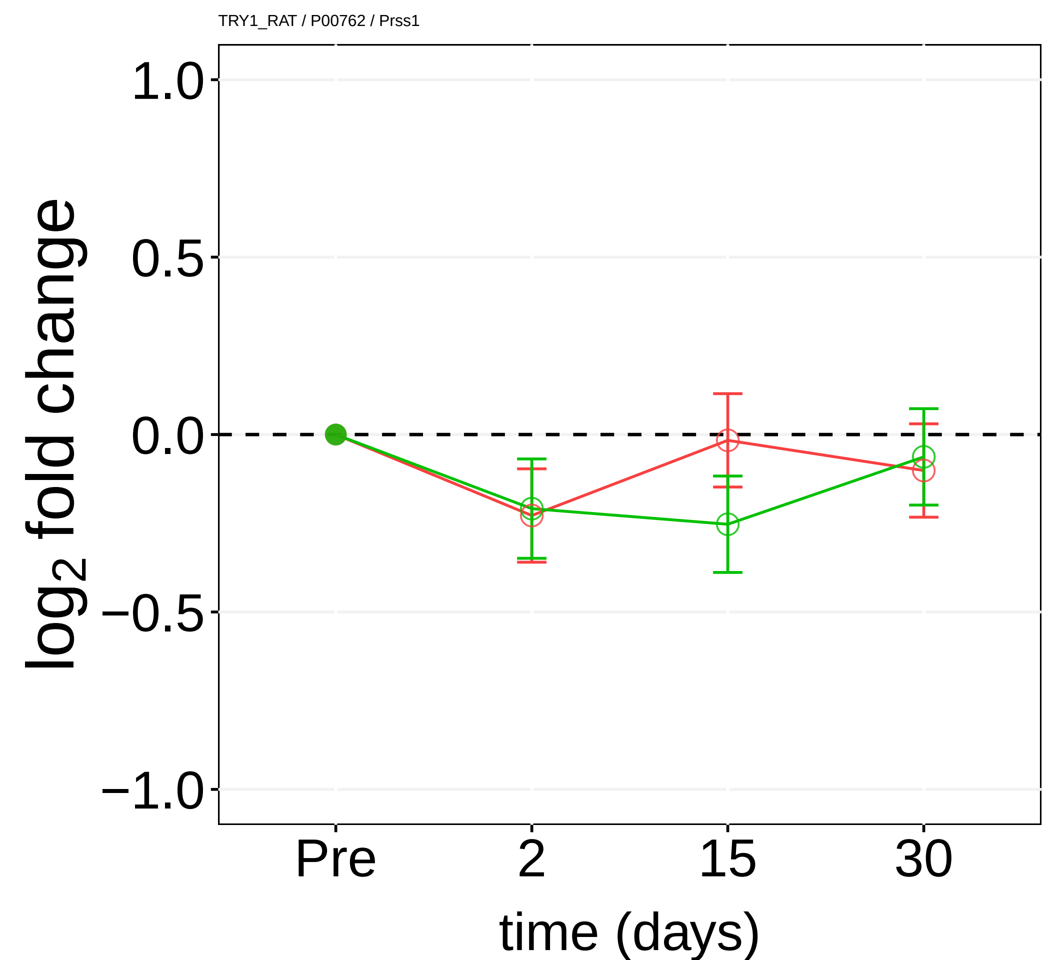
TRY1_RAT / P00762 / Prss1
1.0
0.5
0.0
−0.5
−1.0
log2 fold change
Pre
2
15
30
time (days)

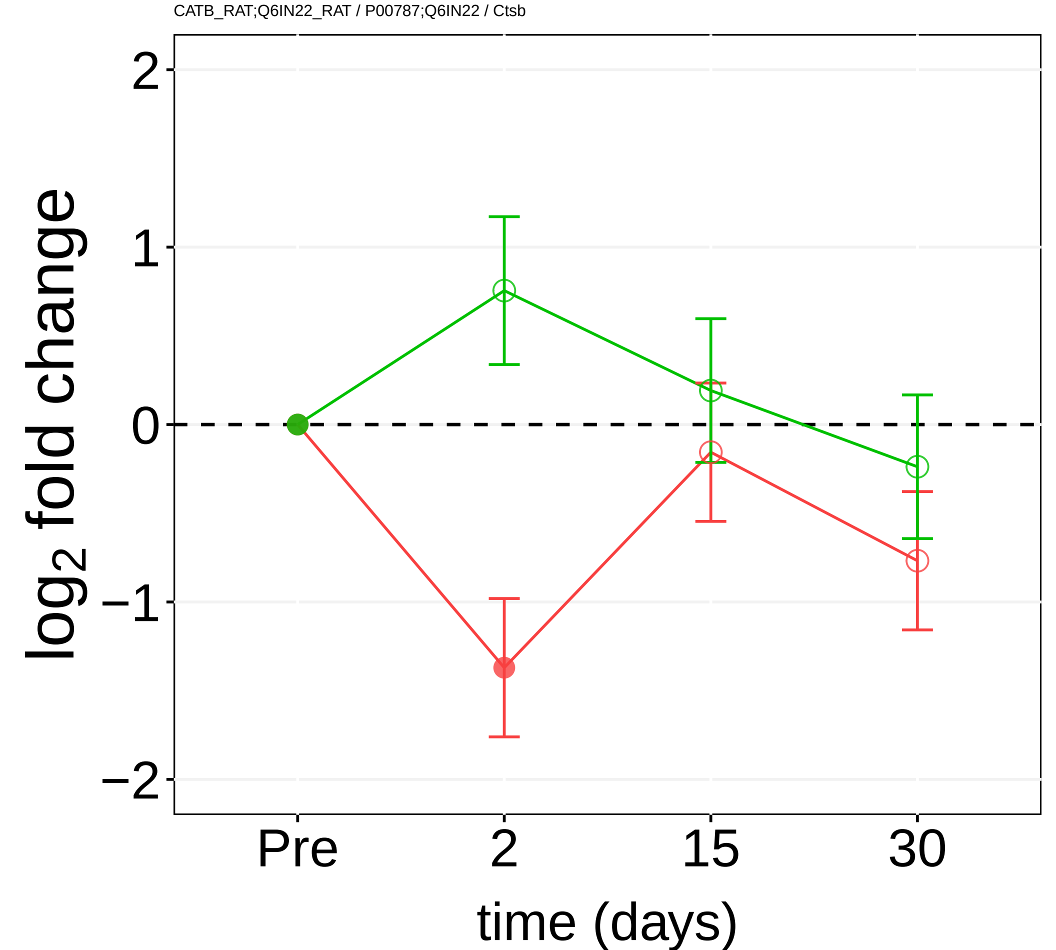

CATB_RAT;Q6IN22_RAT / P00787;Q6IN22 / Ctsb
log2 fold change
2
1
0
−1
−2
Pre
2
15
30
time (days)

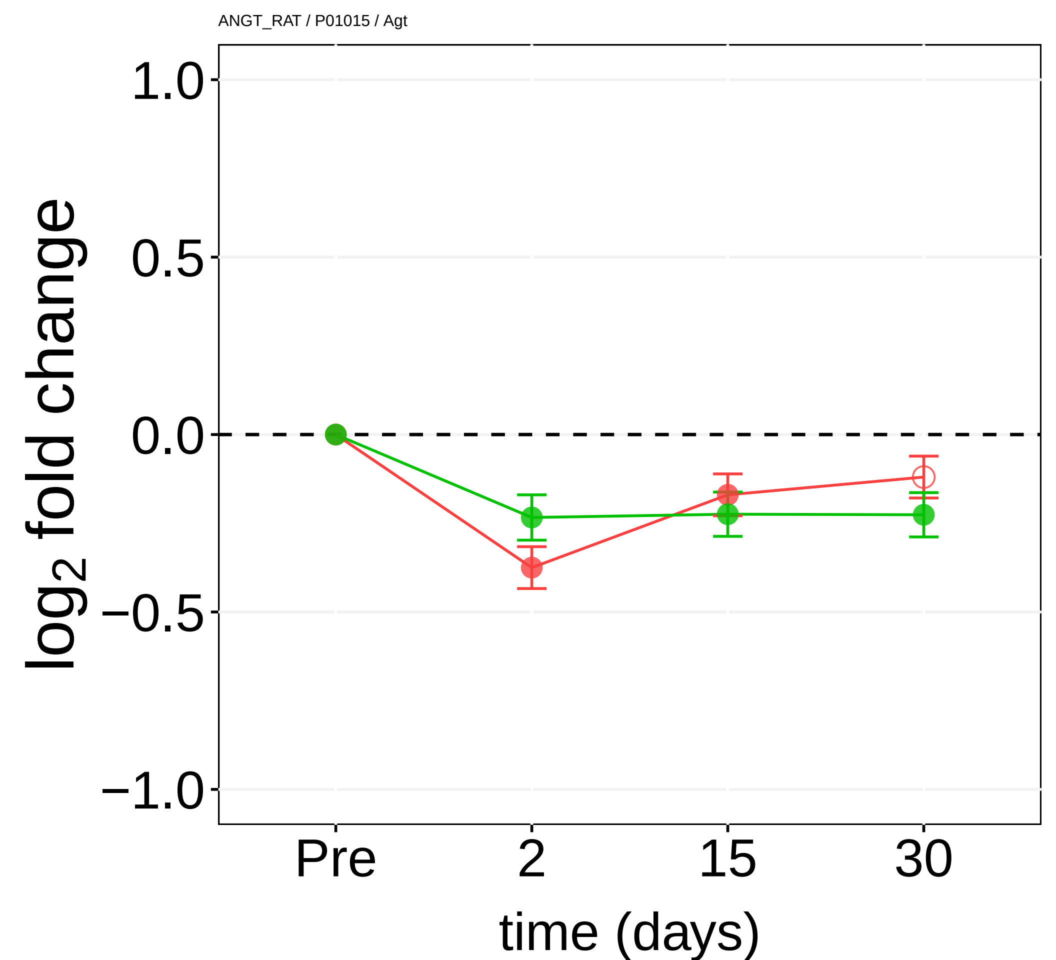

ANGT_RAT / P01015 / Agt
1.0
0.5
0.0
−0.5
−1.0
log2 fold change
Pre
2
15
30
time (days)

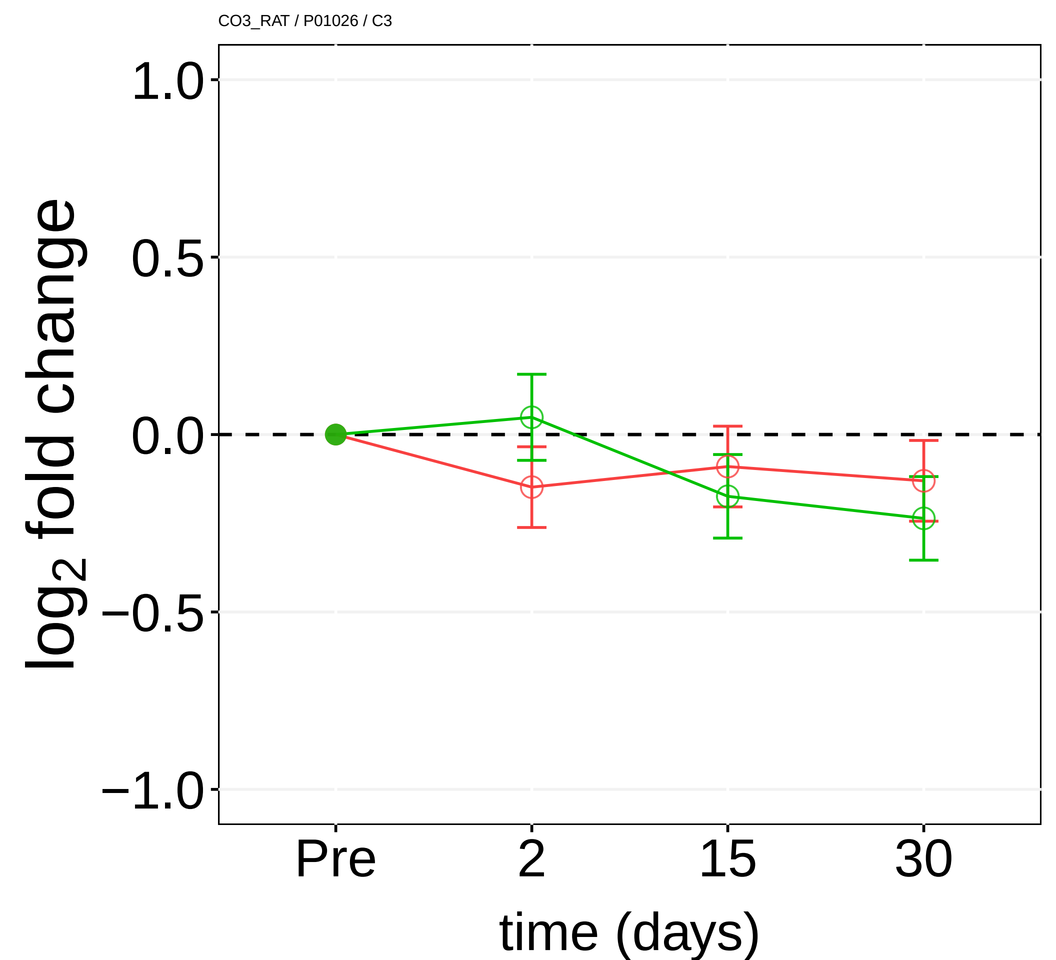
CO3_RAT / P01026 / C3
1.0
0.5
0.0
−0.5
−1.0
log2 fold change
Pre
2
15
30
time (days)

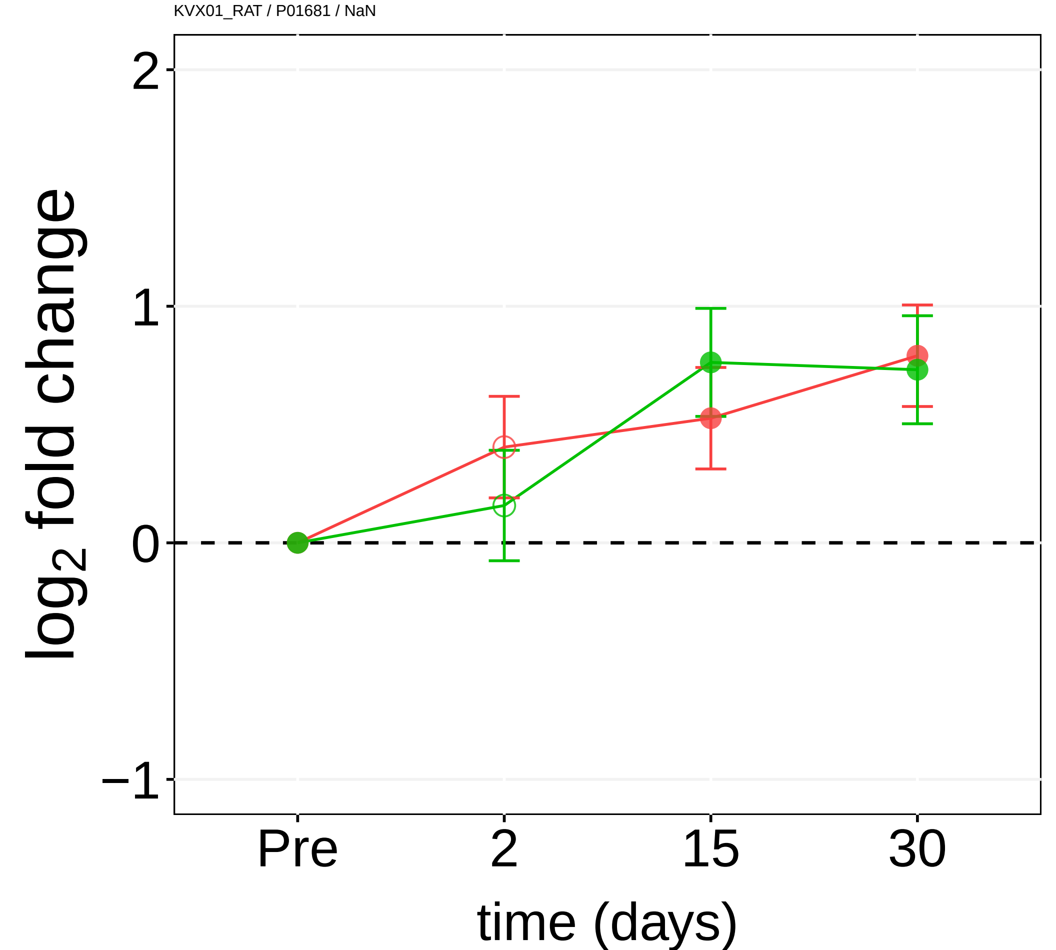
KVX01_RAT / P01681 / NaN
2
1
0
−1
log2 fold change
Pre
2
15
30
time (days)

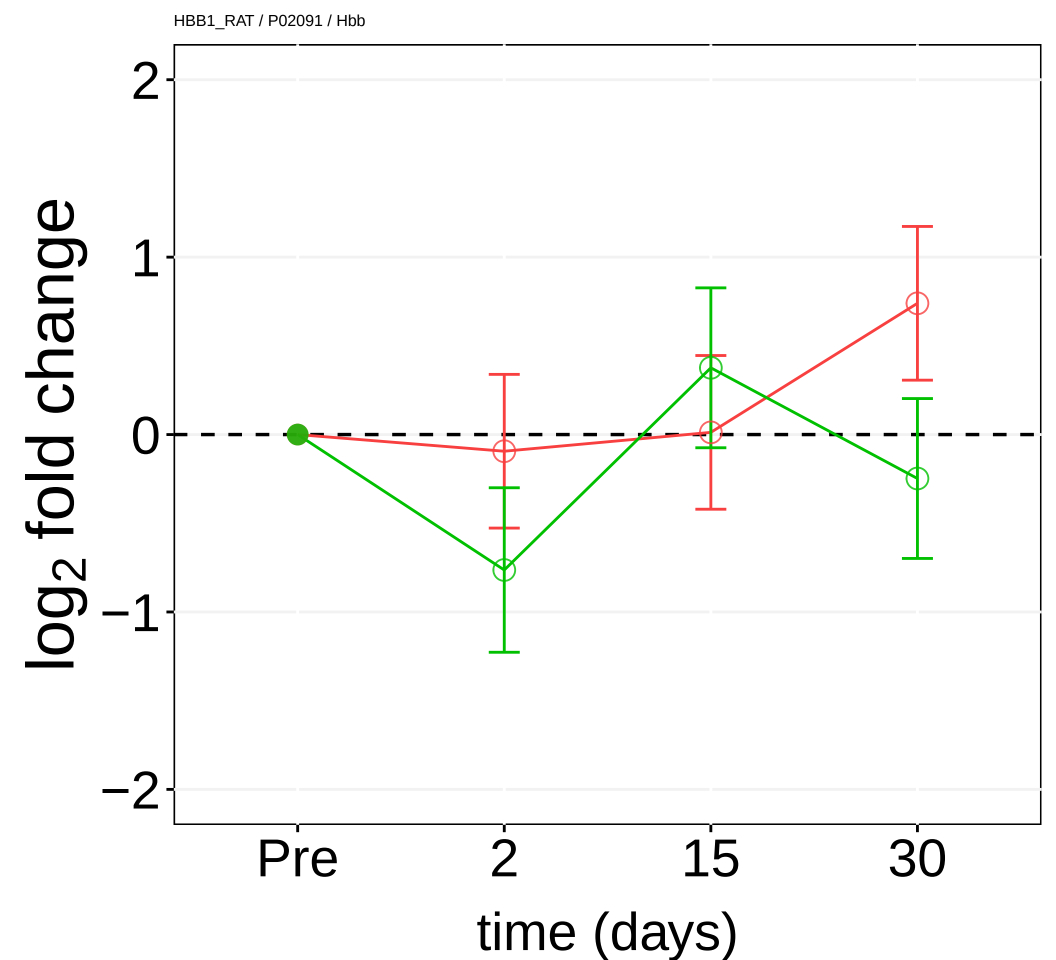
HBB1_RAT / P02091 / Hbb
log2 fold change
2
1
0
−1
−2
Pre
2
15
30
time (days)

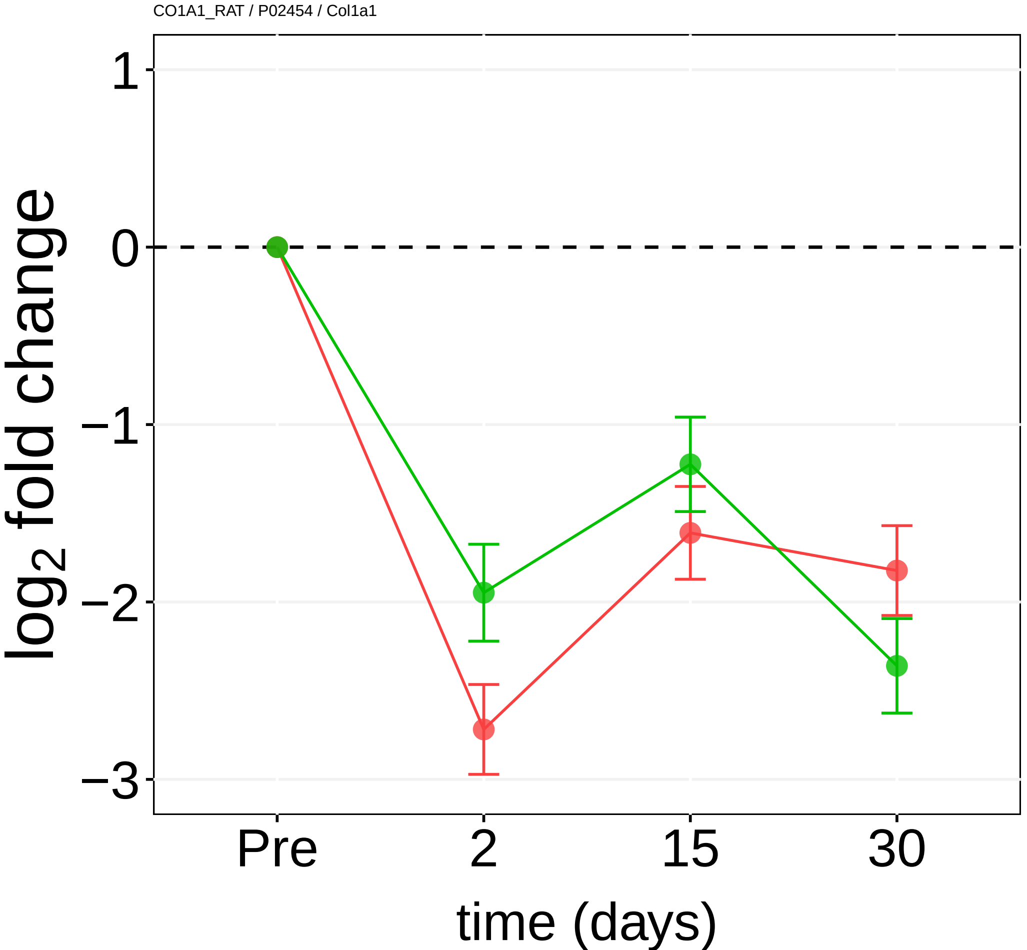

CO1A1_RAT / P02454 / Col1a1
1
0
−1
−2
−3
log2 fold change
Pre
2
15
30
time (days)

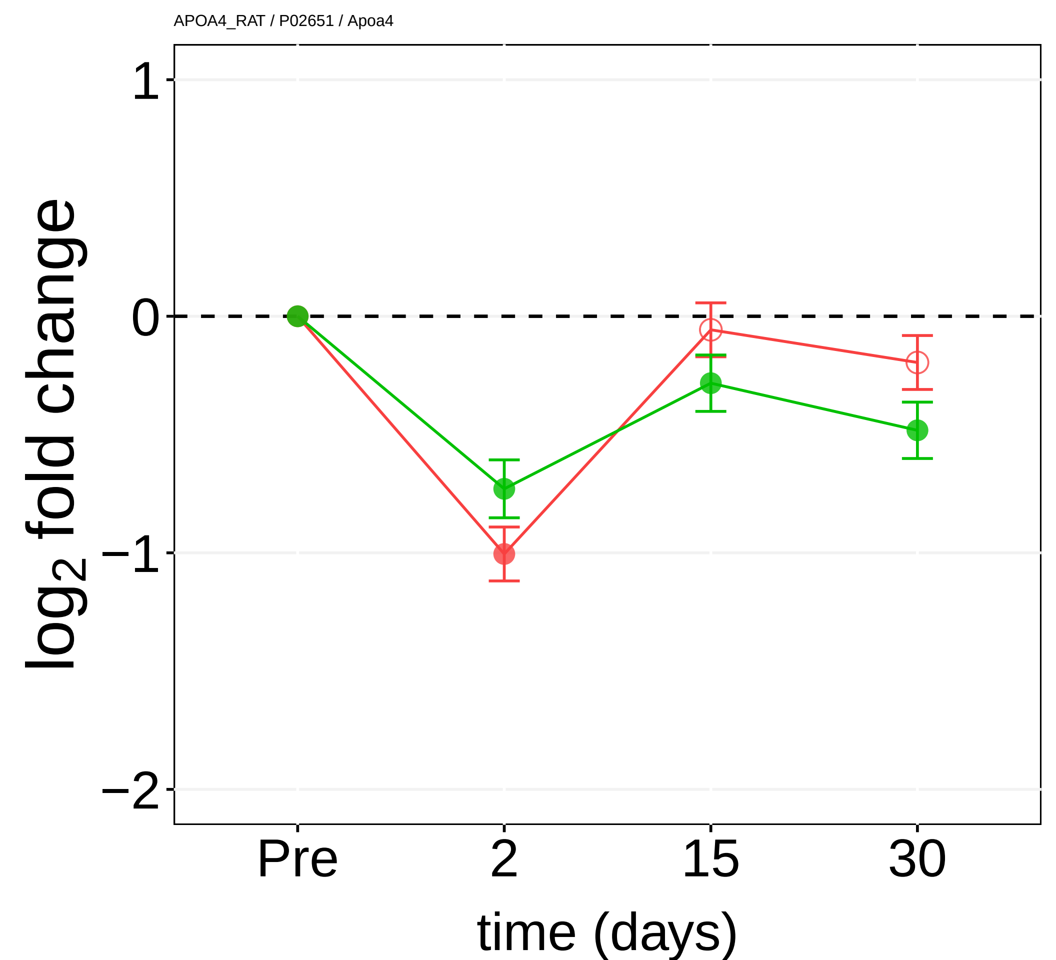
APOA4_RAT / P02651 / Apoa4
1
0
−1
−2
log2 fold change
Pre
2
15
30
time (days)

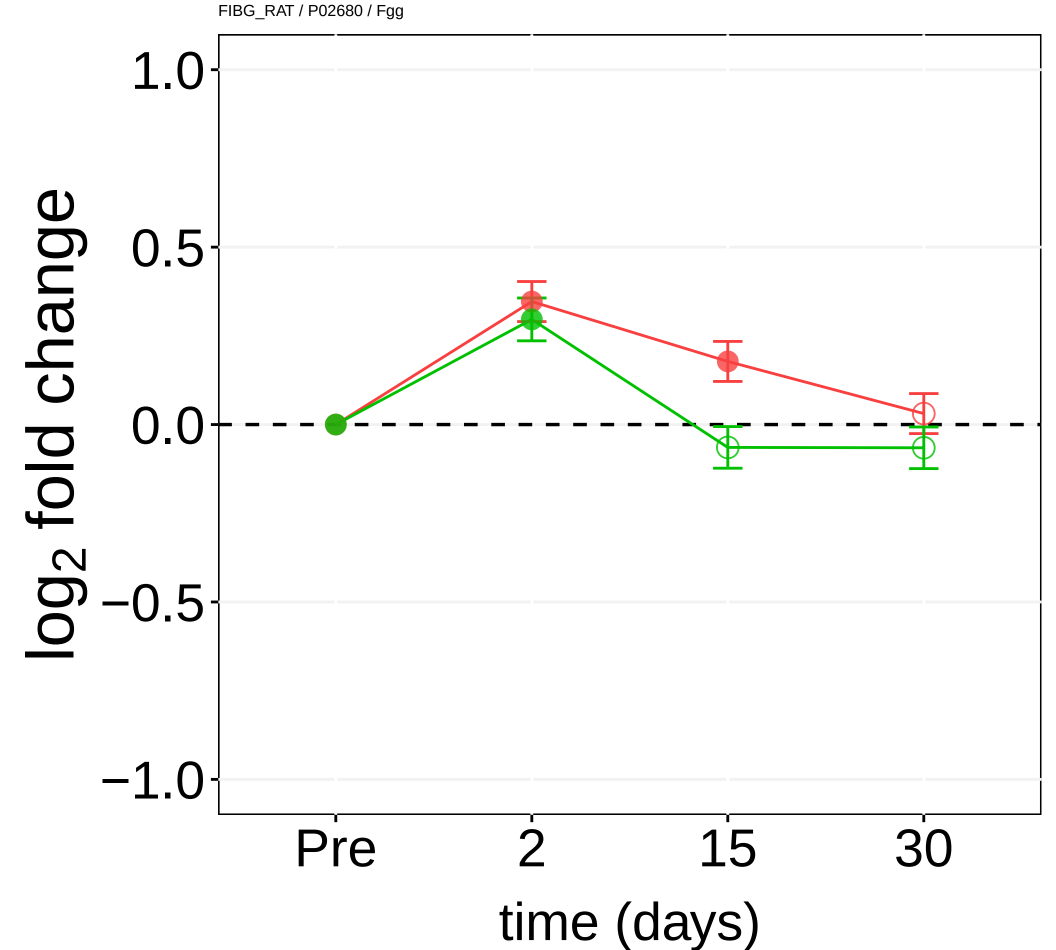
FIBG_RAT / P02680 / Fgg
1.0
0.5
0.0
−0.5
−1.0
log2 fold change
Pre
2
15
30
time (days)

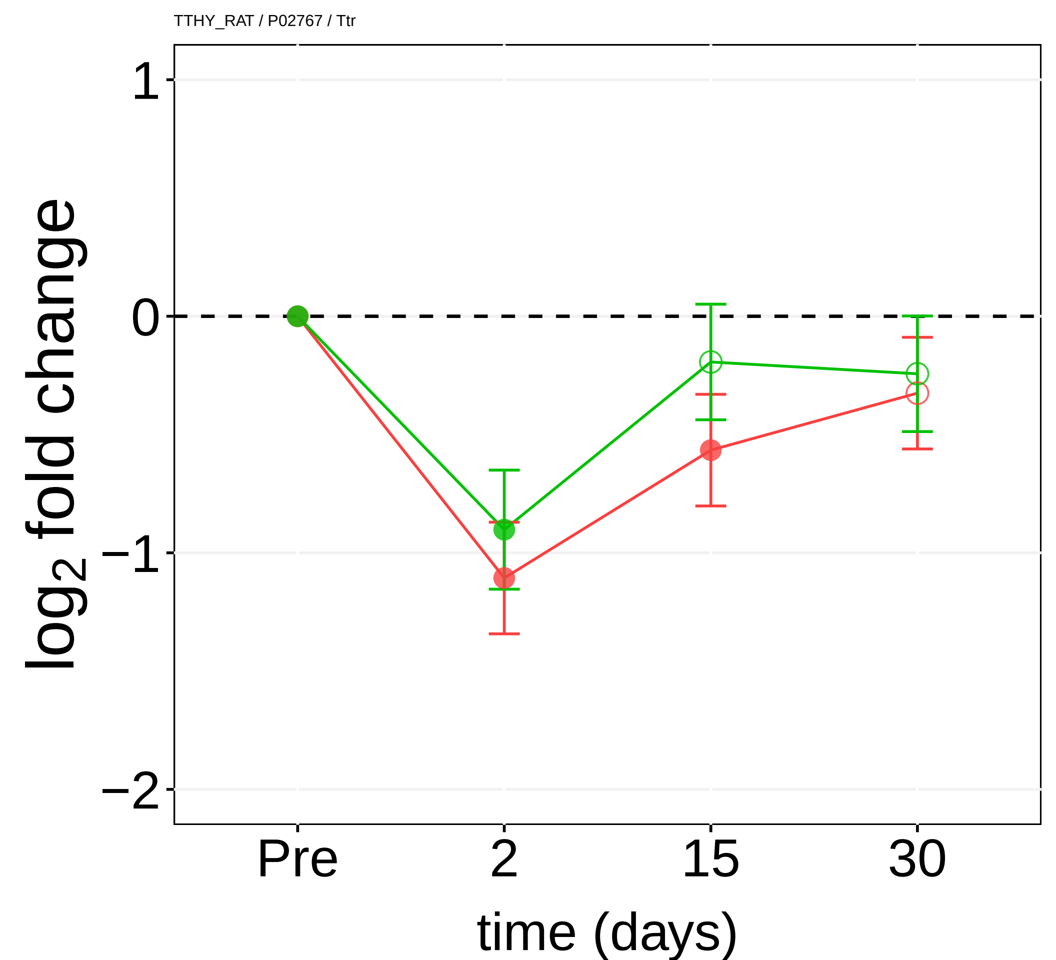

TTHY_RAT / P02767 / Ttr
1
0
−1
−2
log2 fold change
Pre
2
15
30
time (days)

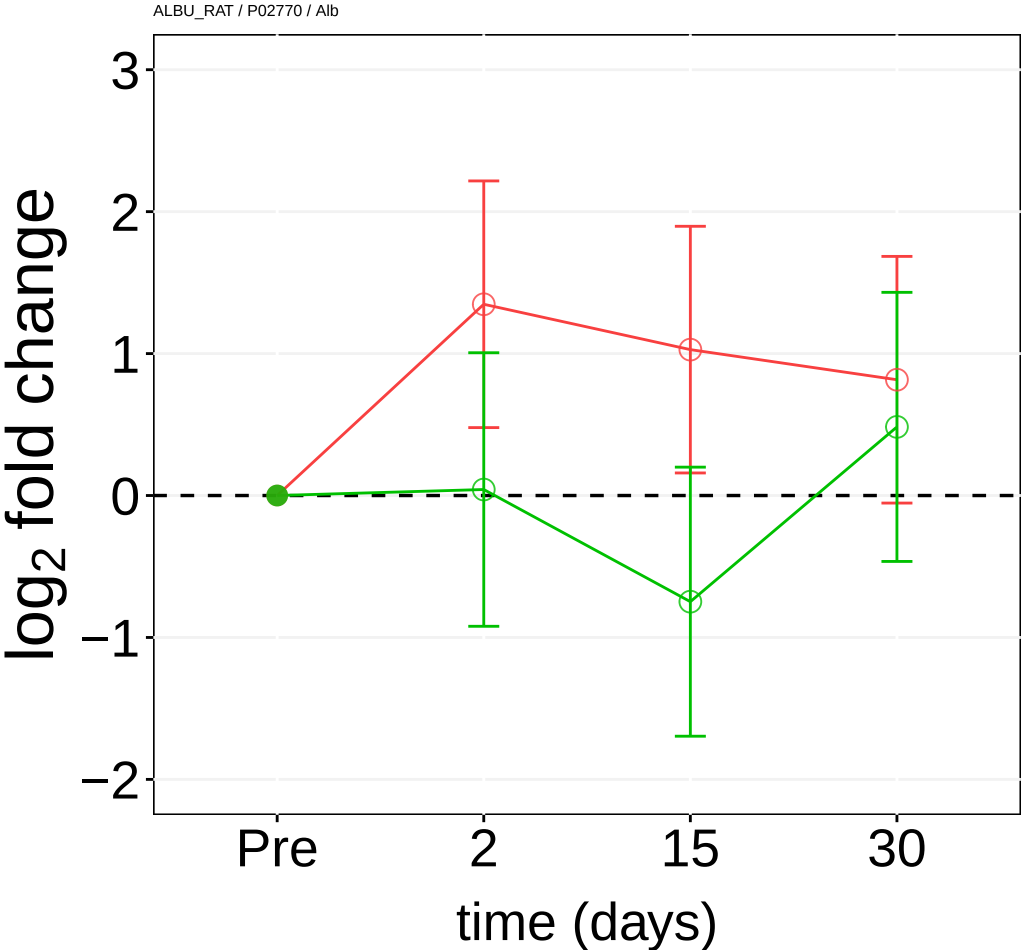
ALBU_RAT / P02770 / Alb
log2 fold change
3
2
1
0
−1
−2
Pre
2
15
30
time (days)

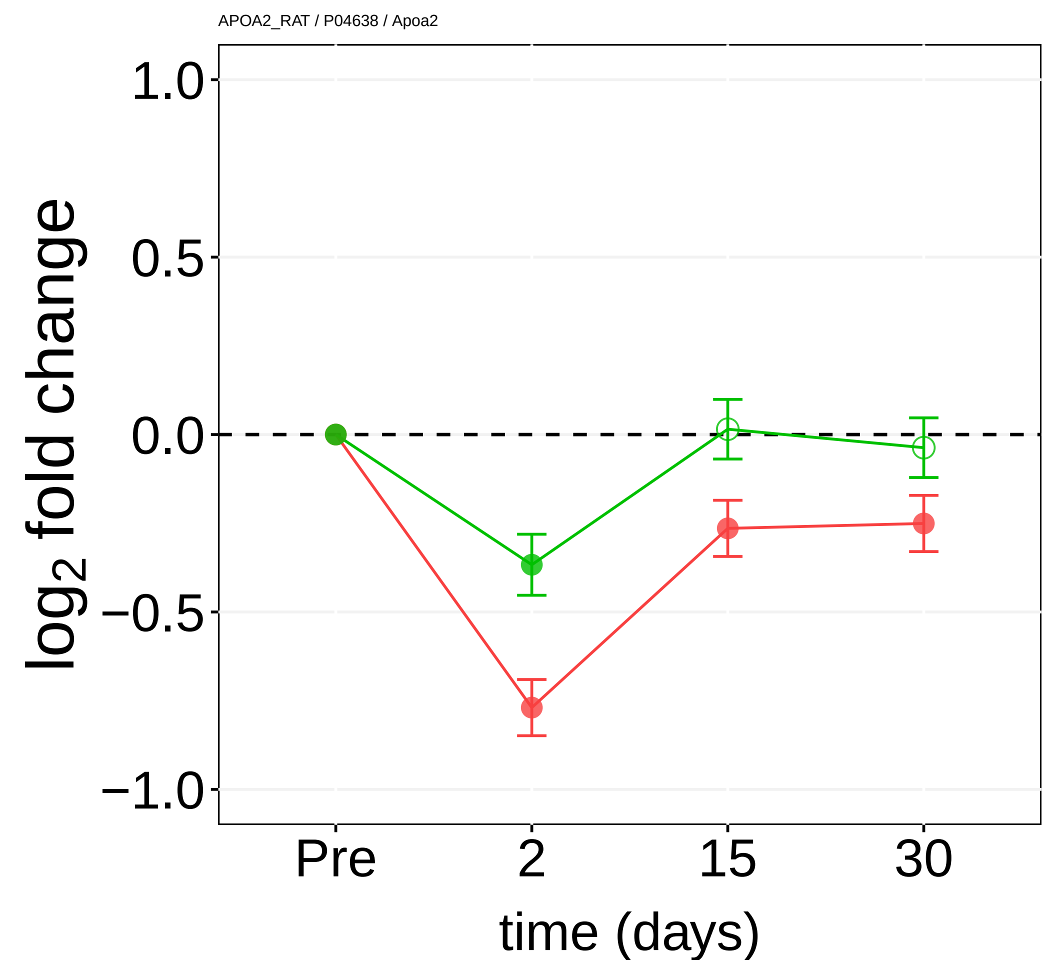
APOA2_RAT / P04638 / Apoa2
1.0
0.5
0.0
−0.5
−1.0
log2 fold change
Pre
2
15
30
time (days)

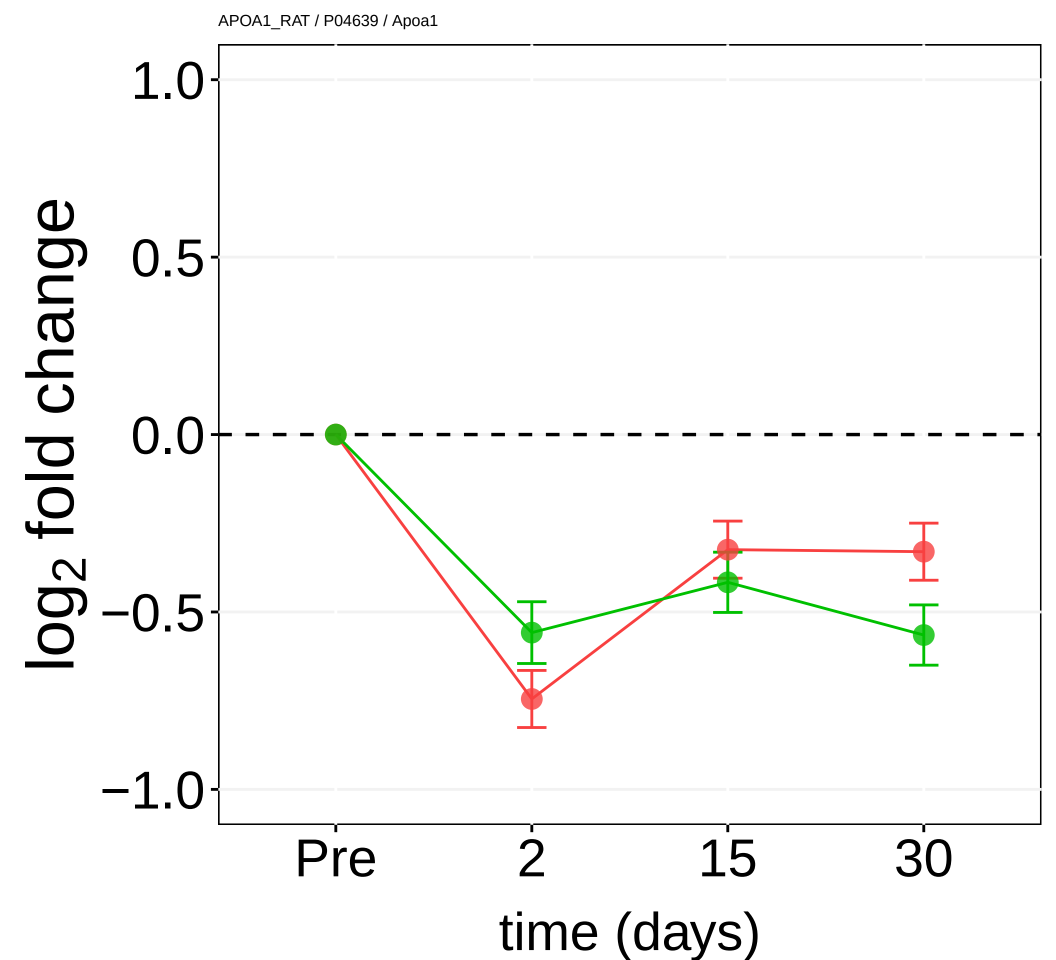
APOA1_RAT / P04639 / Apoa1
1.0
0.5
0.0
−0.5
−1.0
log_2 fold change
Pre
2
15
30
time (days)

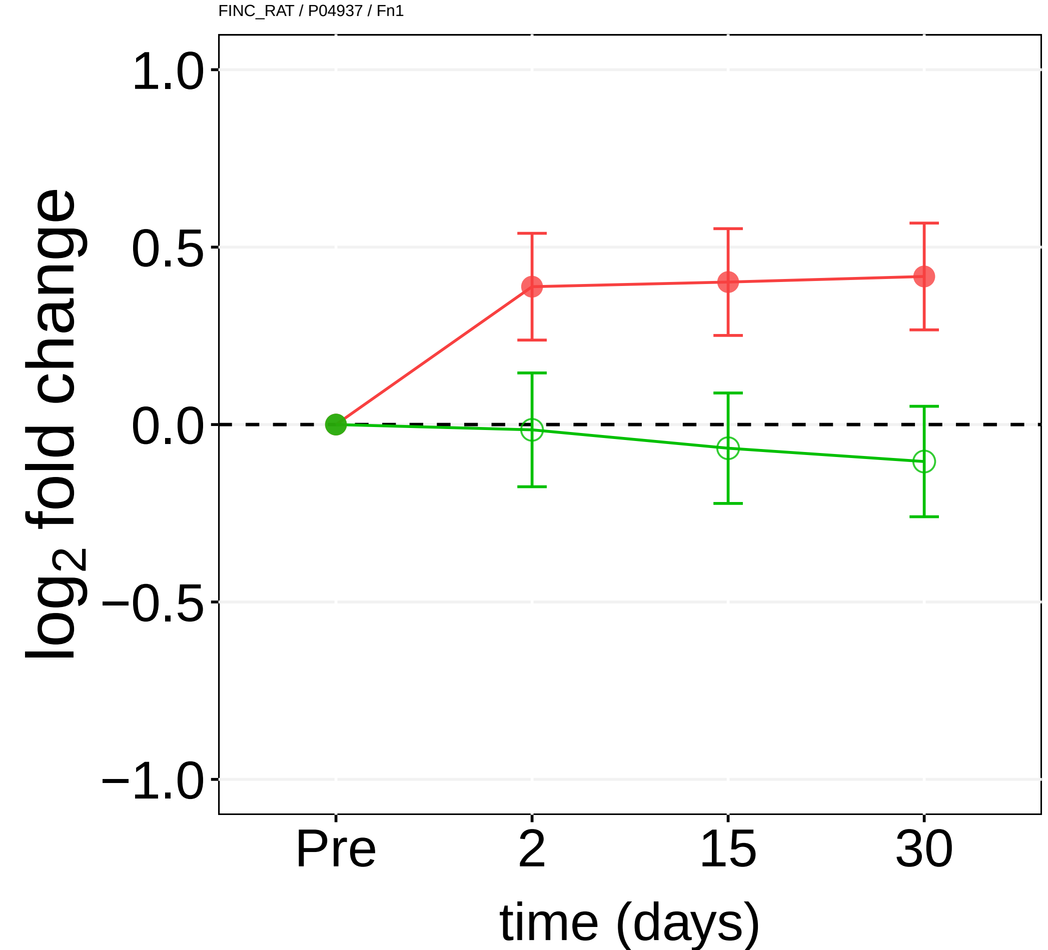
FINC_RAT / P04937 / Fn1
1.0
0.5
0.0
−0.5
−1.0
log2 fold change
Pre
2
15
30
time (days)

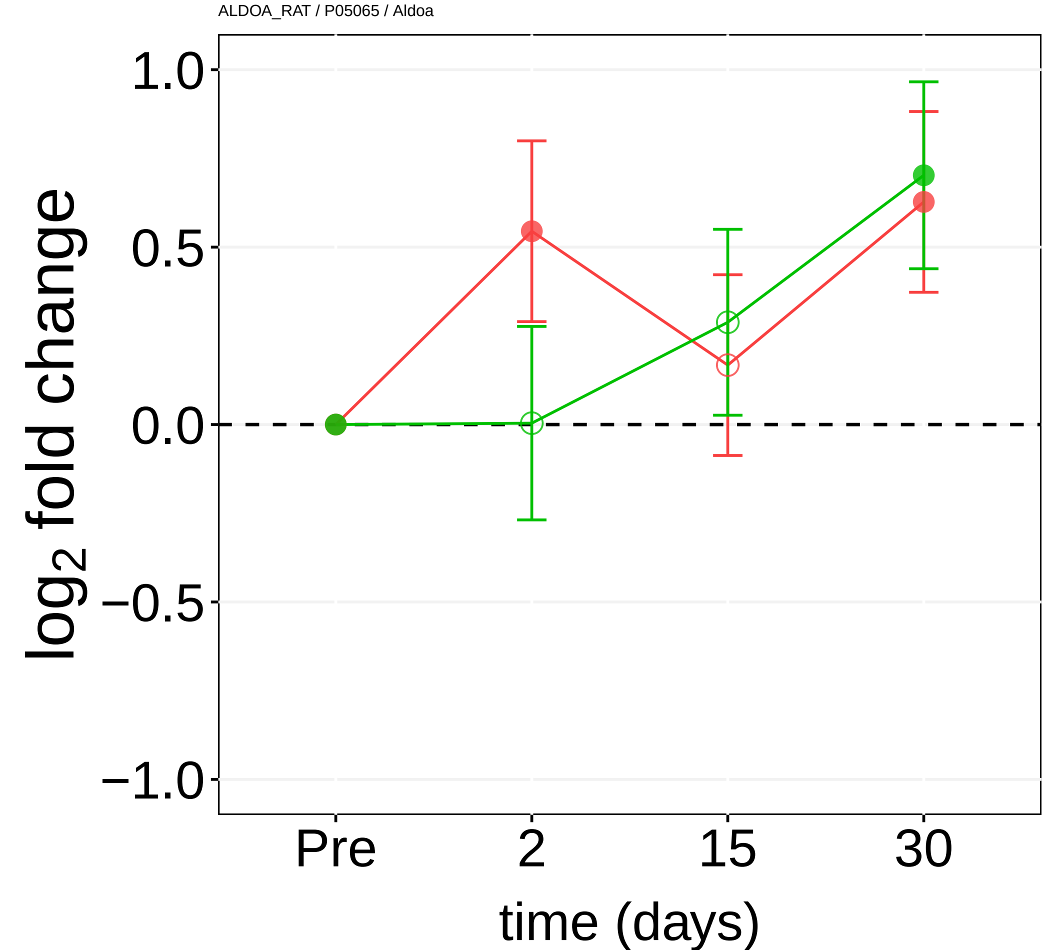
ALDOA_RAT / P05065 / Aldoa
log2 fold change
1.0
0.5
0.0
−0.5
−1.0
Pre
2
15
30
time (days)

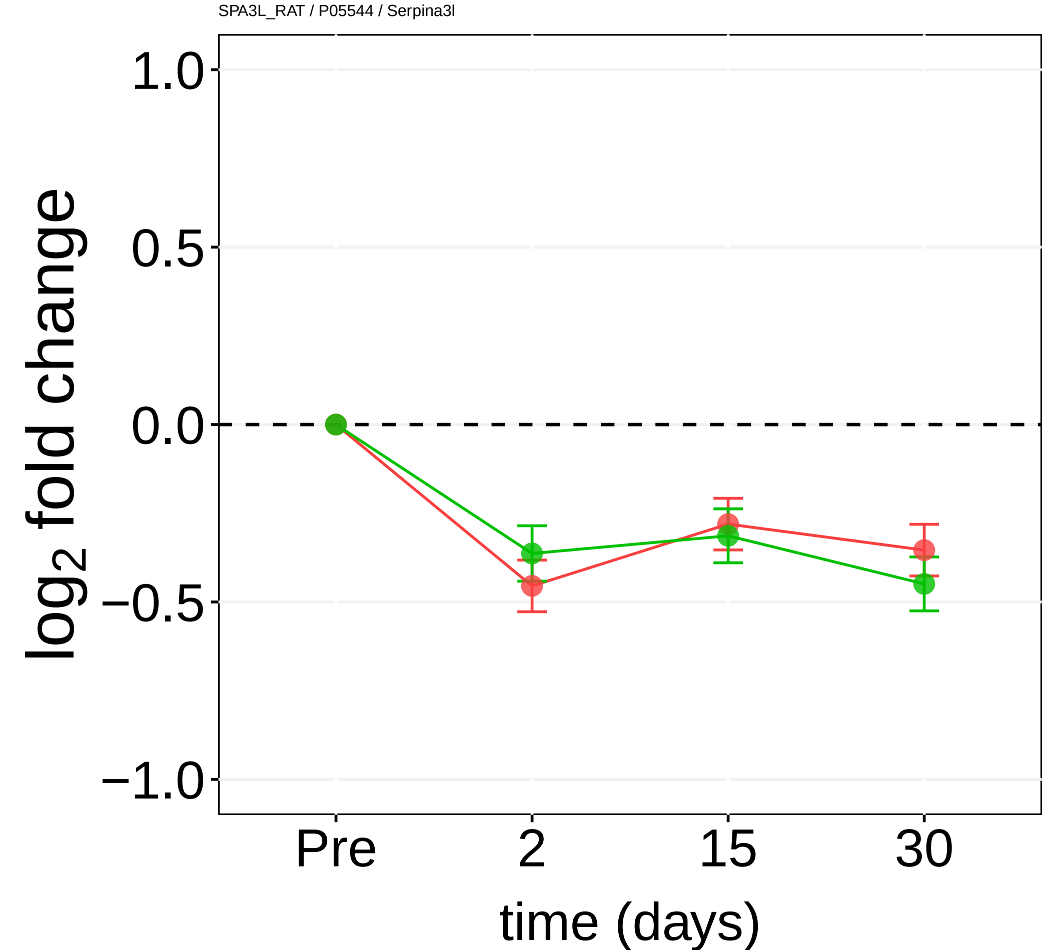

SPA3L_RAT / P05544 / Serpina3l
log₂ fold change
1.0
0.5
0.0
−0.5
−1.0
Pre
2
15
30
time (days)

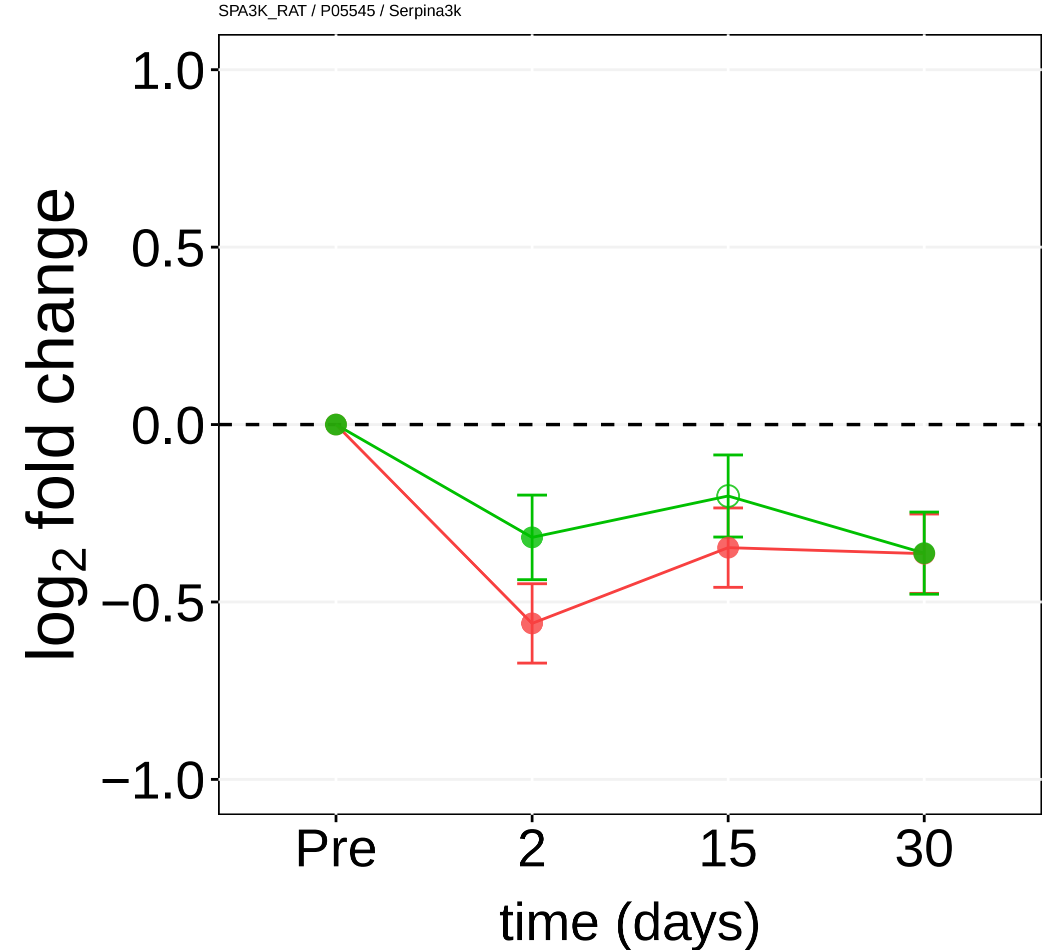
SPA3K_RAT / P05545 / Serpina3k
1.0
0.5
0.0
−0.5
−1.0
$\log_2$ fold change
Pre
2
15
30
time (days)

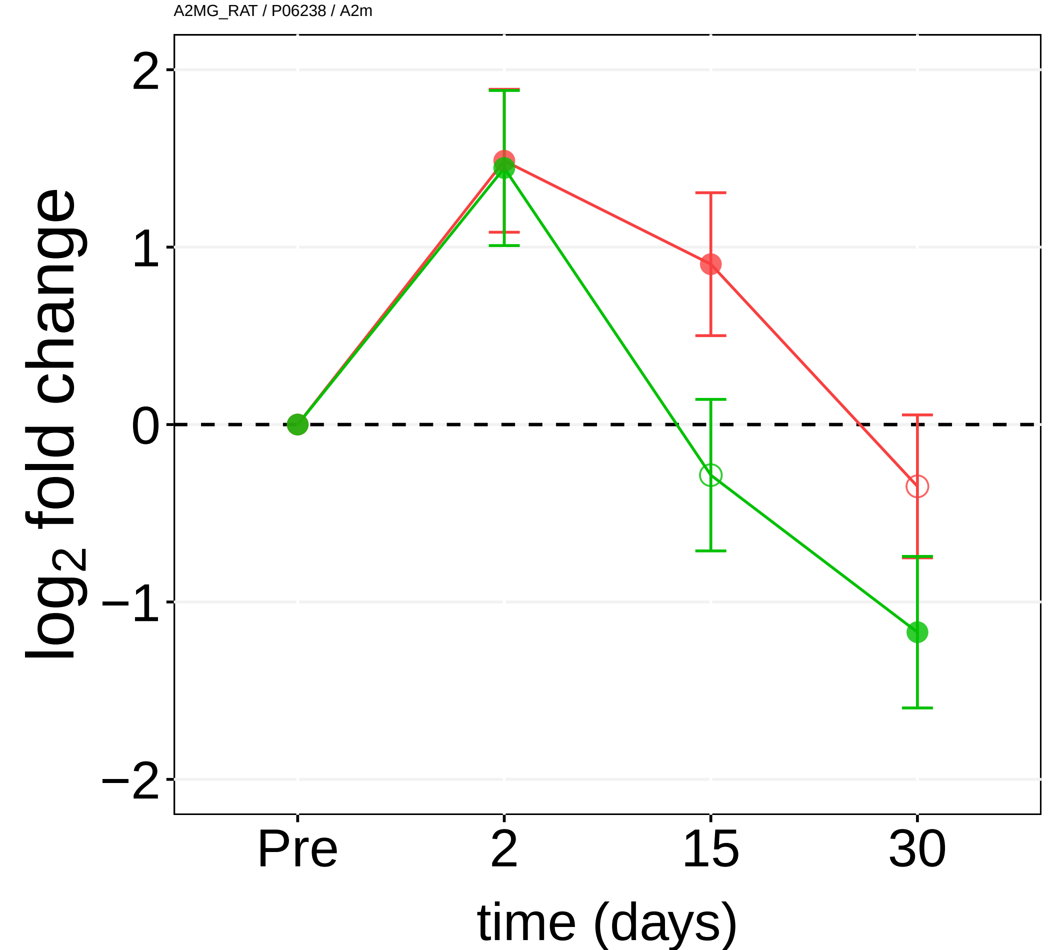
A2MG_RAT / P06238 / A2m
log2 fold change
2
1
0
−1
−2
Pre
2
15
30
time (days)

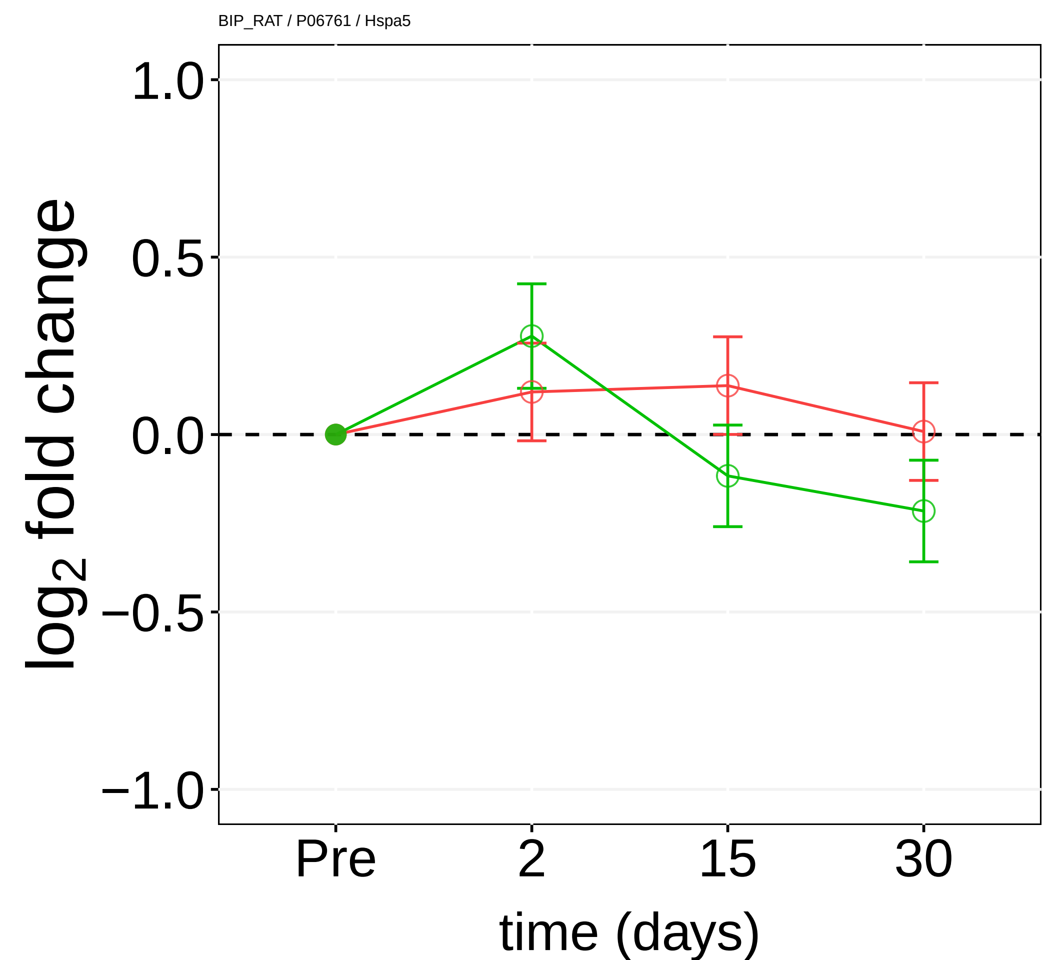
BIP_RAT / P06761 / Hspa5
1.0
0.5
0.0
−0.5
−1.0
log2 fold change
Pre
2
15
30
time (days)

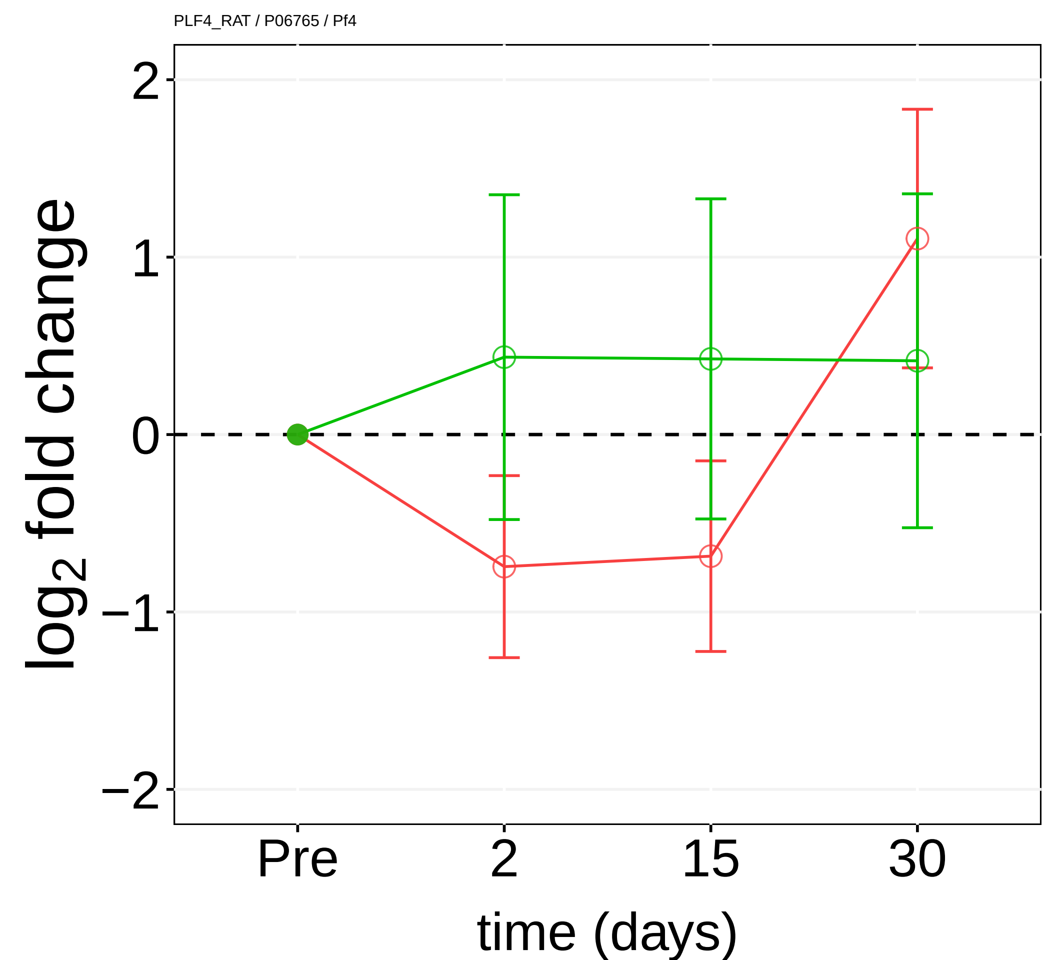
PLF4_RAT / P06765 / Pf4
log2 fold change
2
1
0
−1
−2
Pre
2
15
30
time (days)

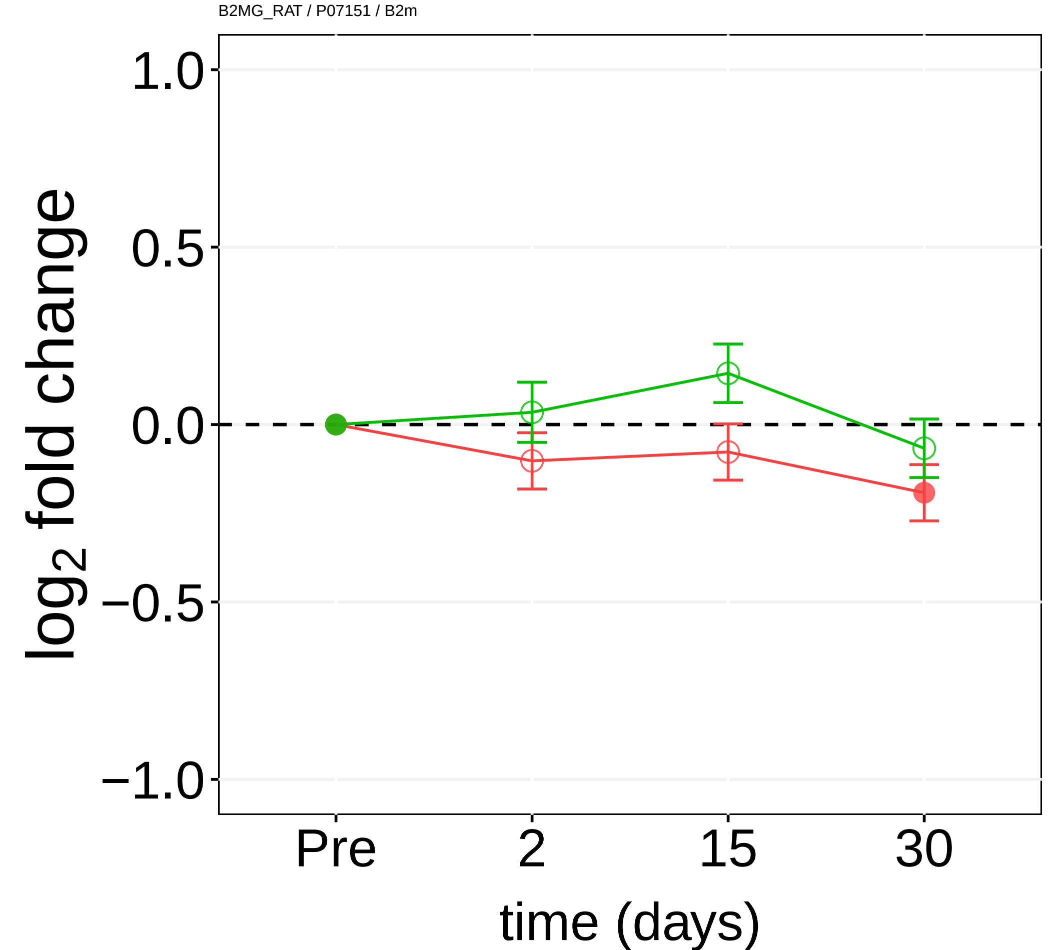

B2MG_RAT / P07151 / B2m
1.0
0.5
0.0
−0.5
−1.0
log2 fold change
Pre
2
15
30
time (days)

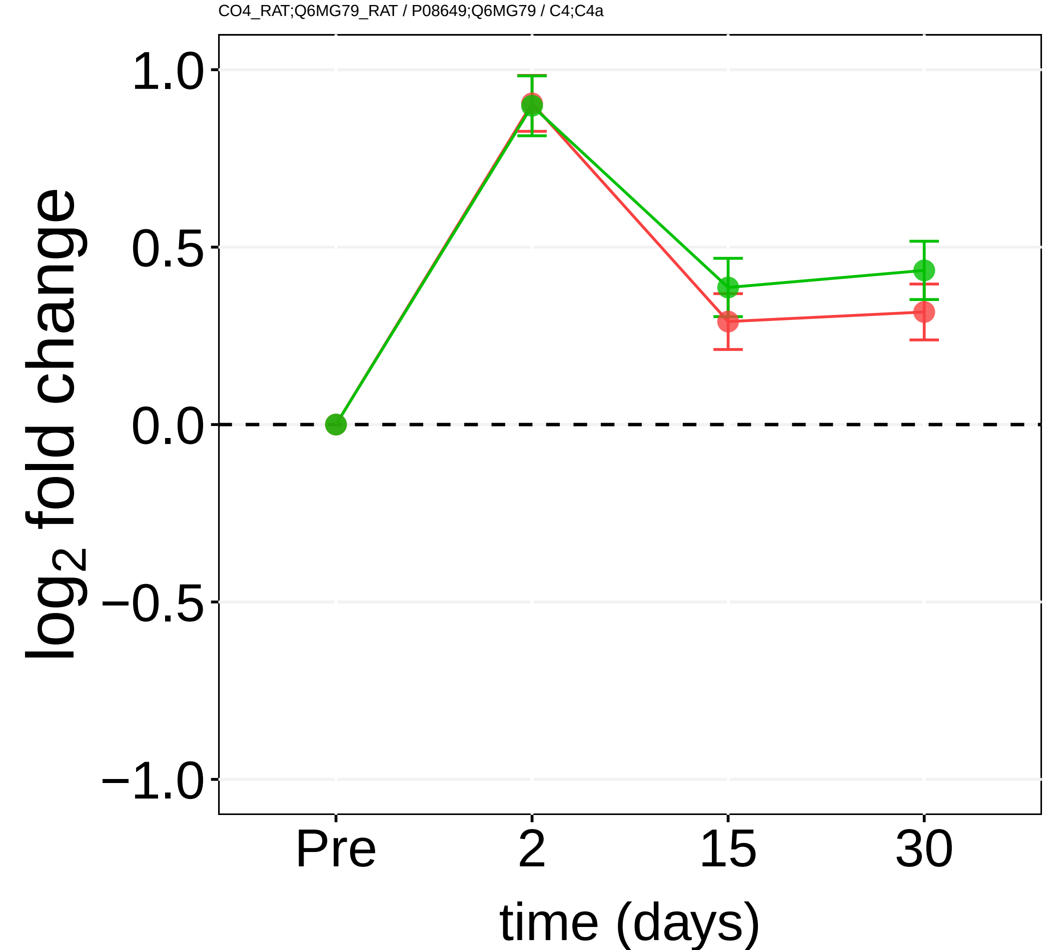

CO4_RAT;Q6MG79_RAT / P08649;Q6MG79 / C4;C4a
1.0
0.5
0.0
−0.5
−1.0
log2 fold change
Pre
2
15
30
time (days)

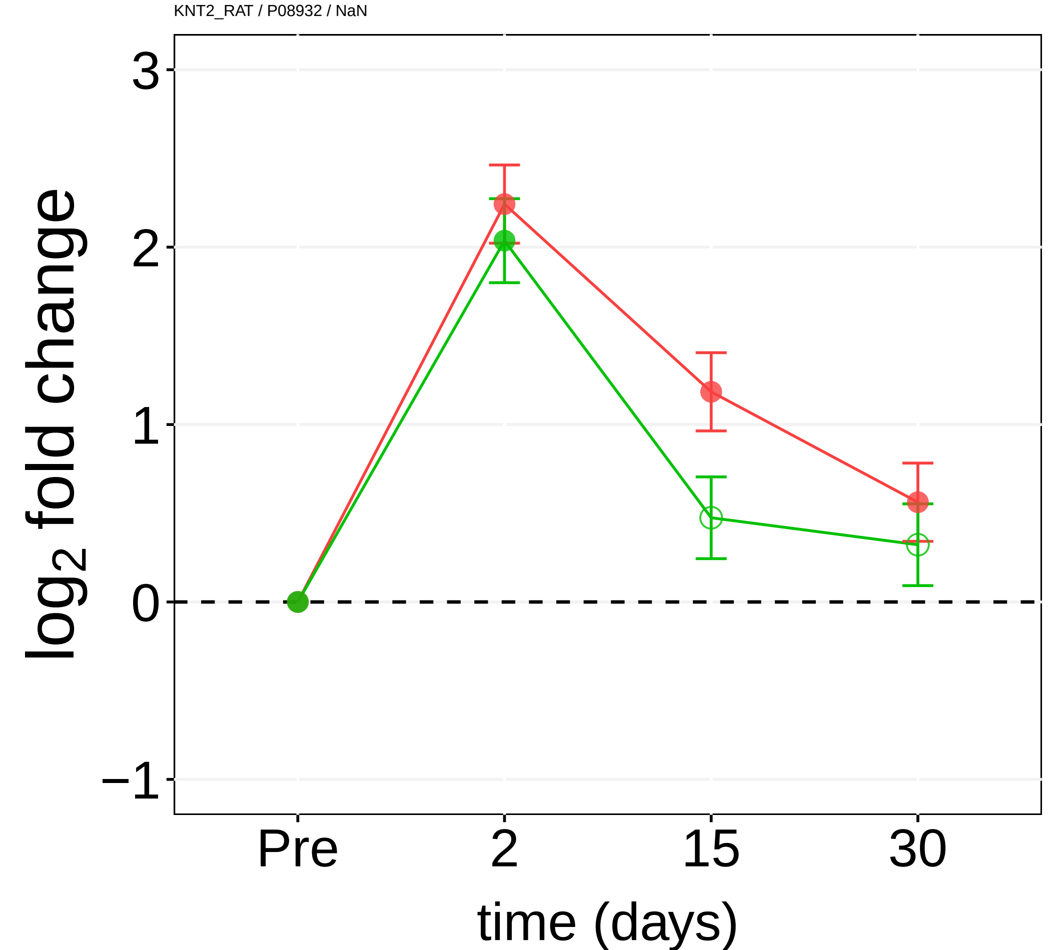
KNT2_RAT / P08932 / NaN
3
2
1
0
−1
$\log_2$ fold change
Pre
2
15
30
time (days)

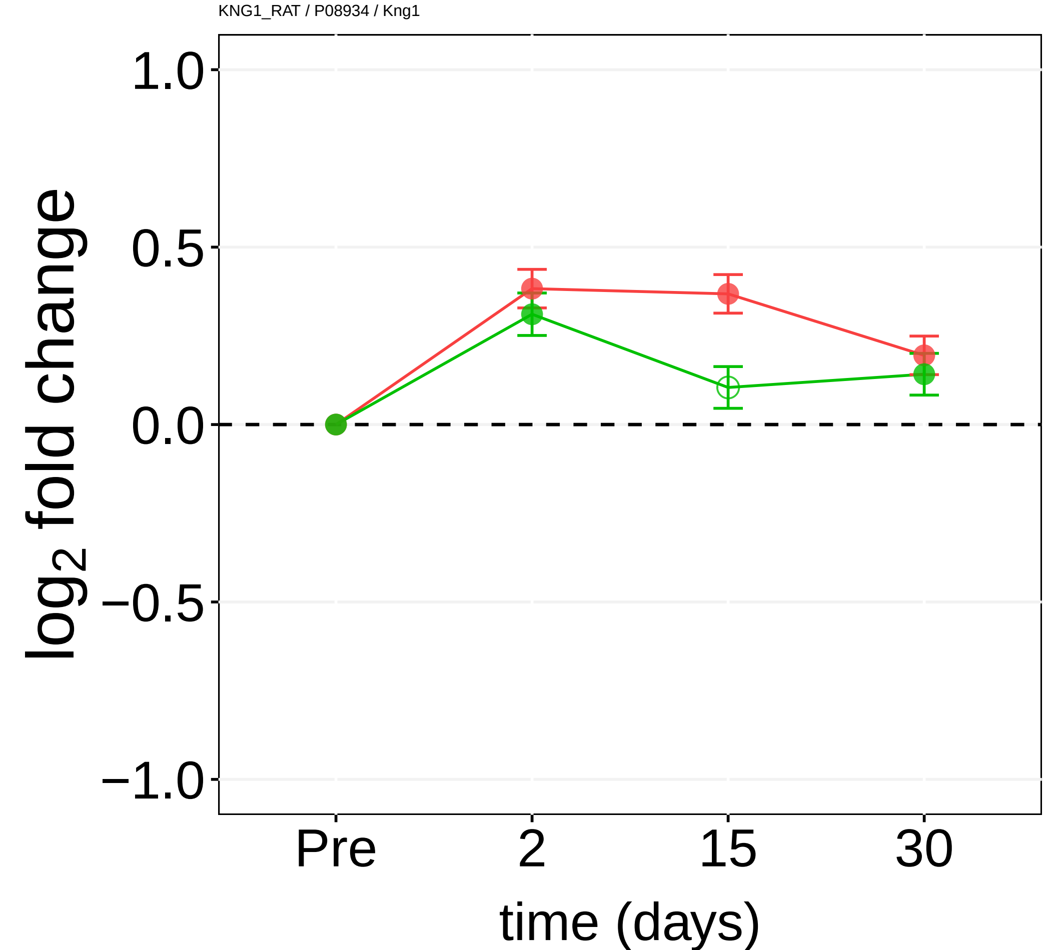
KNG1_RAT / P08934 / Kng1
log2 fold change
1.0
0.5
0.0
−0.5
−1.0
Pre
2
15
30
time (days)

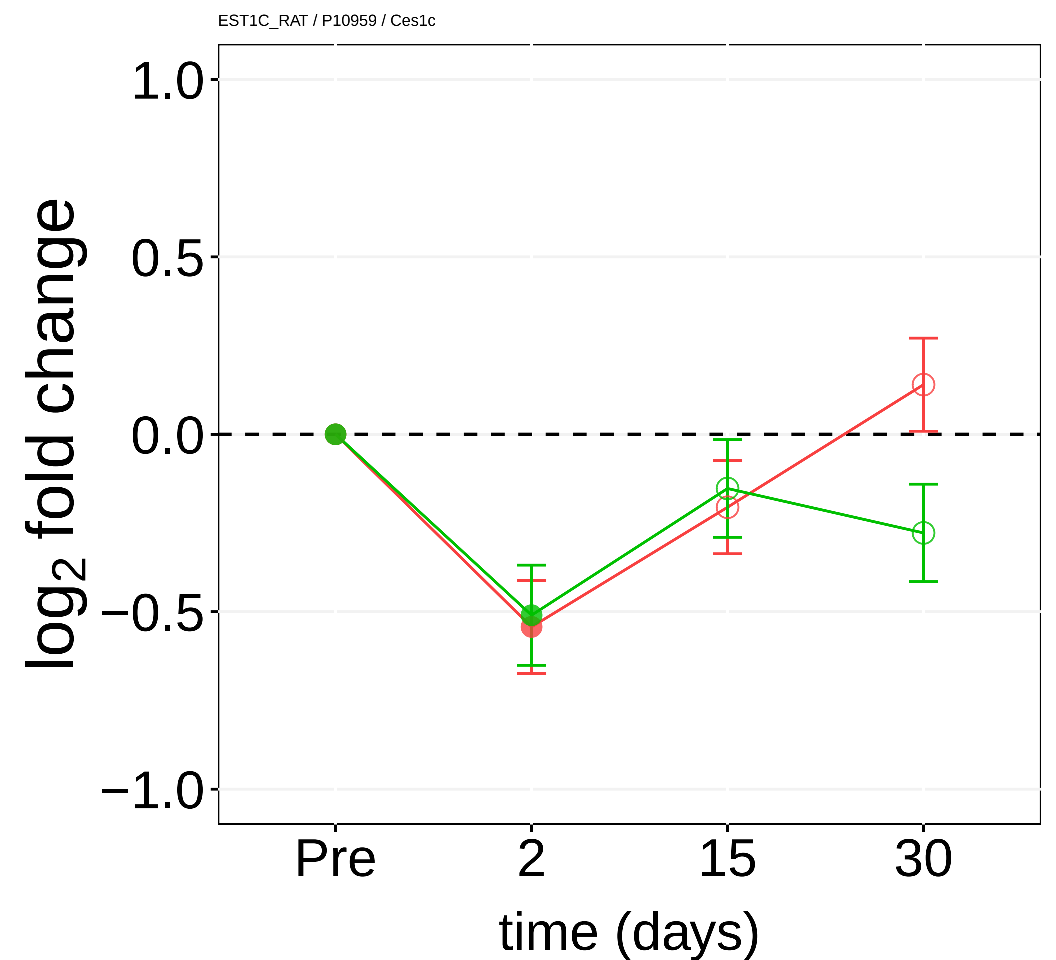
EST1C_RAT / P10959 / Ces1c
1.0
0.5
0.0
−0.5
−1.0
$\log_2$ fold change
Pre
2
15
30
time (days)

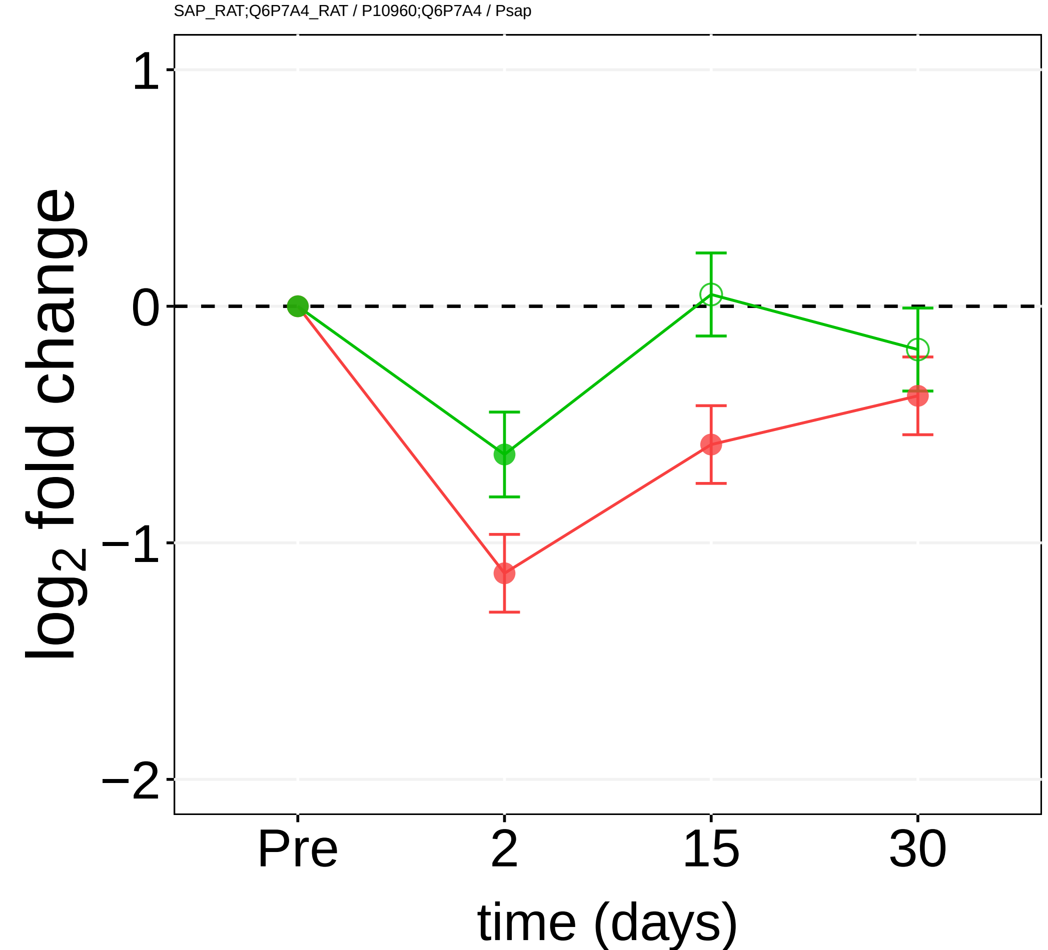

SAP_RAT;Q6P7A4_RAT / P10960;Q6P7A4 / Psap
1
0
−1
−2
log2 fold change
Pre
2
15
30
time (days)

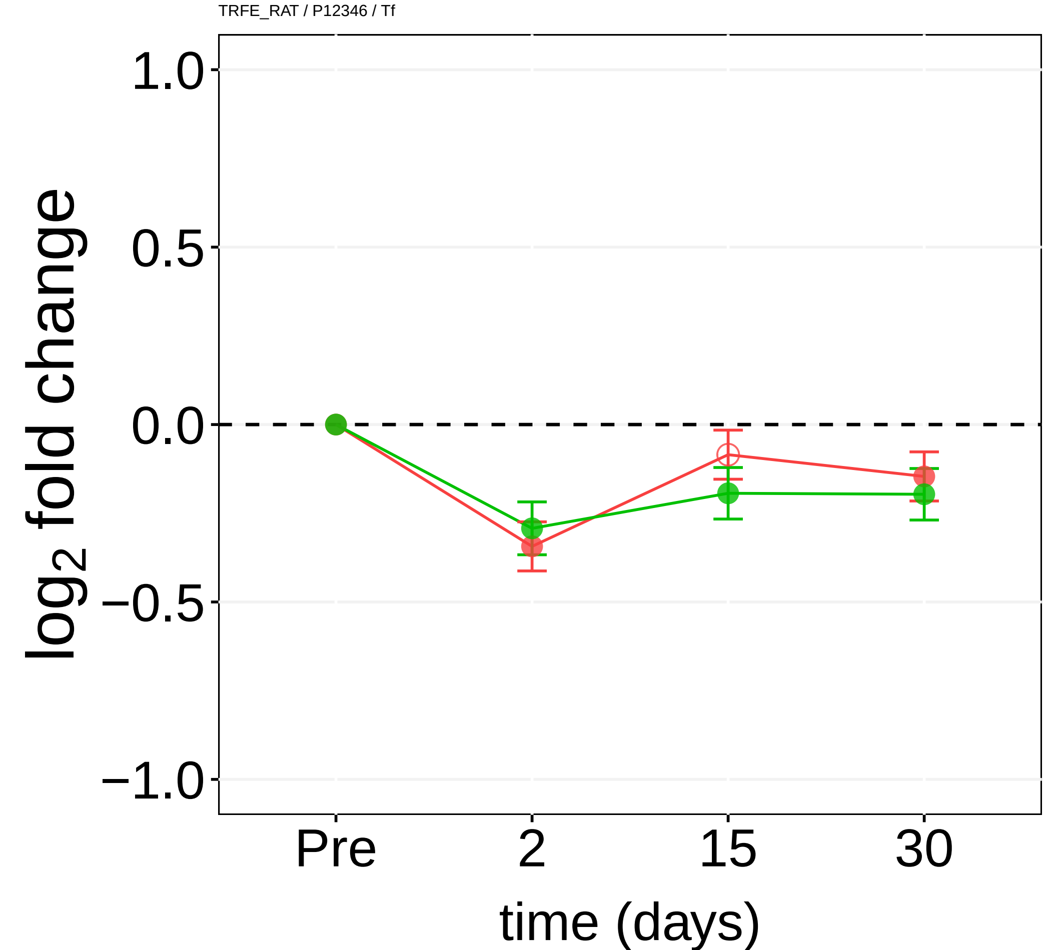
TRFE_RAT / P12346 / Tf
1.0
0.5
0.0
−0.5
−1.0
log2 fold change
Pre
2
15
30
time (days)

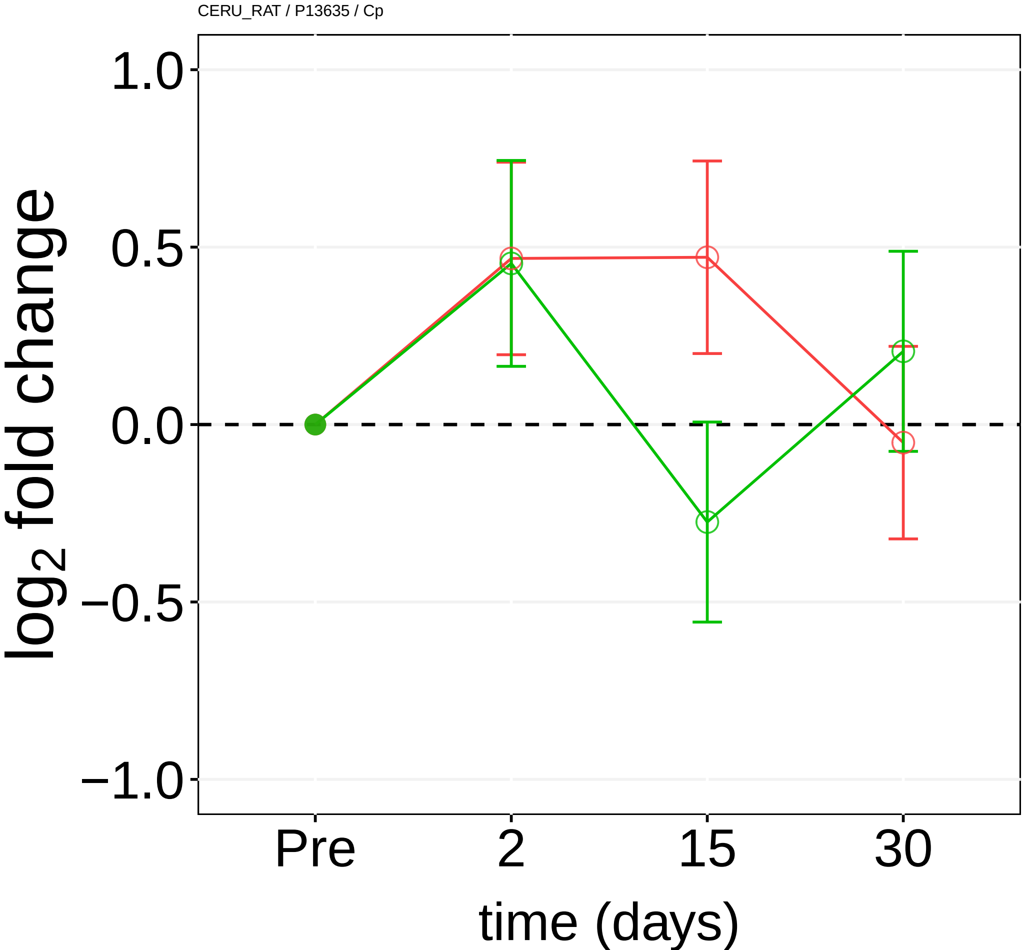

CERU_RAT / P13635 / Cp
1.0
0.5
0.0
−0.5
−1.0
log2 fold change
Pre
2
15
30
time (days)

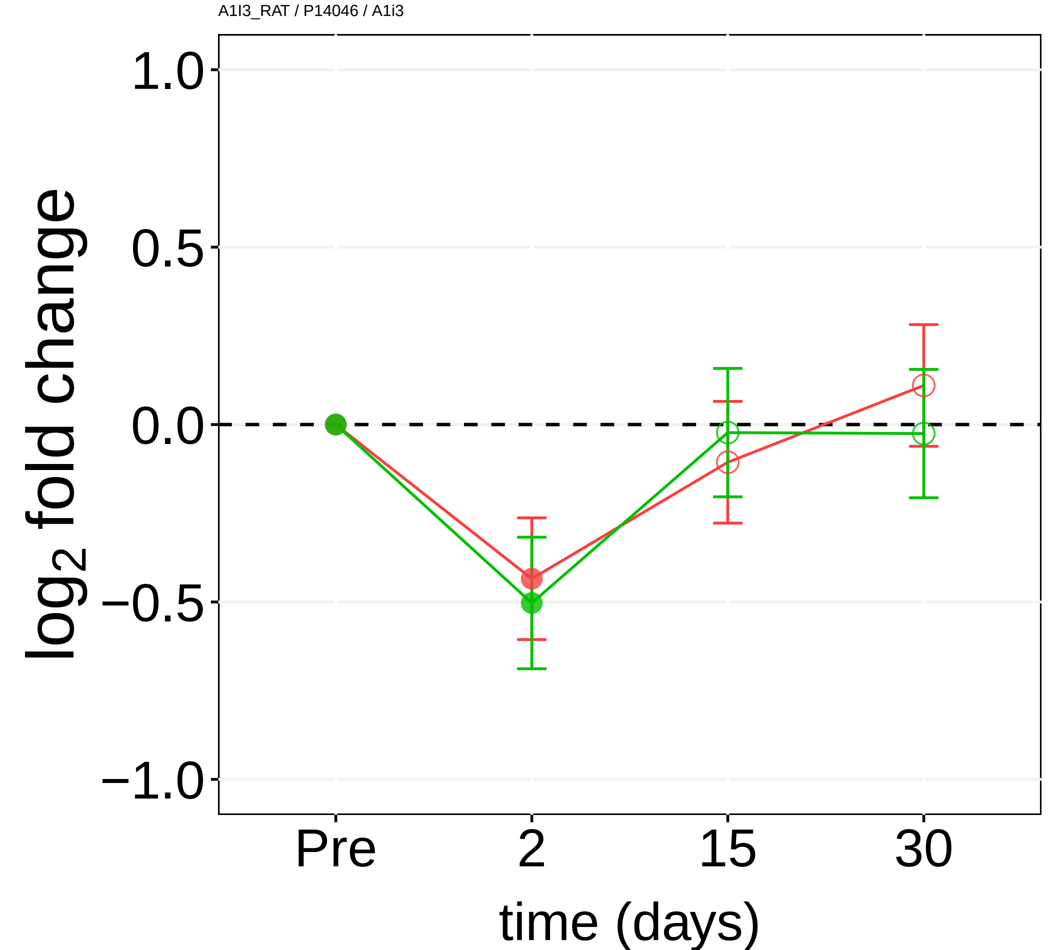
A1I3_RAT / P14046 / A1i3
1.0
0.5
0.0
−0.5
−1.0
log2 fold change
Pre
2
15
30
time (days)

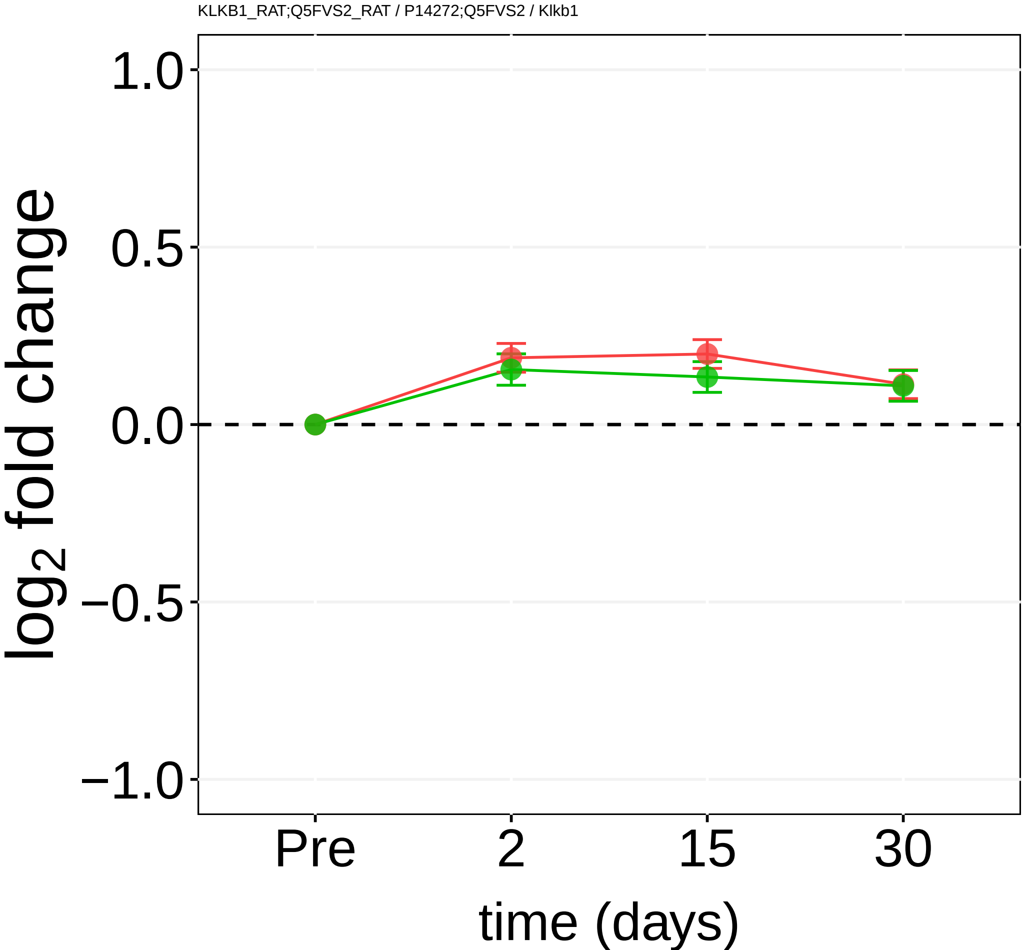

KLKB1_RAT;Q5FVS2_RAT / P14272;Q5FVS2 / Klkb1
$\log_2$ fold change
1.0
0.5
0.0
−0.5
−1.0
Pre
2
15
30
time (days)

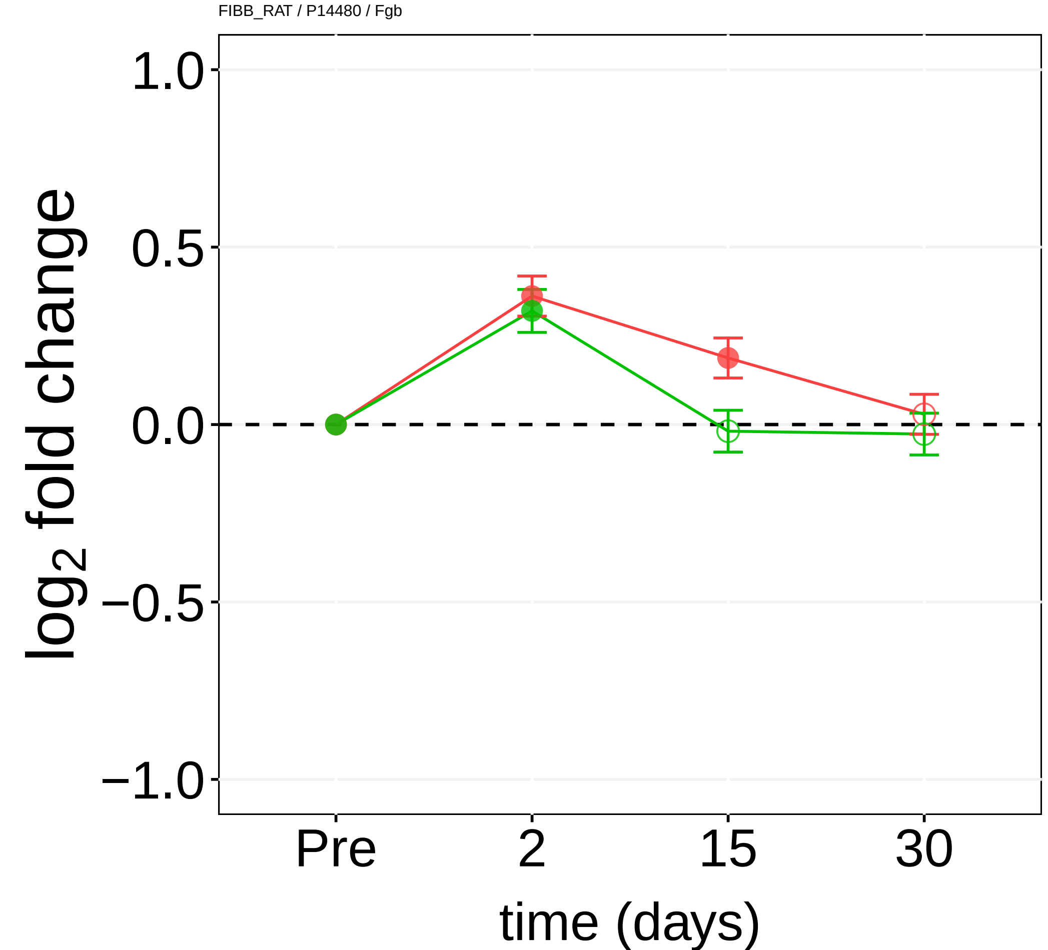

FIBB_RAT / P14480 / Fgb
1.0
0.5
0.0
−0.5
−1.0
log2 fold change
Pre
2
15
30
time (days)

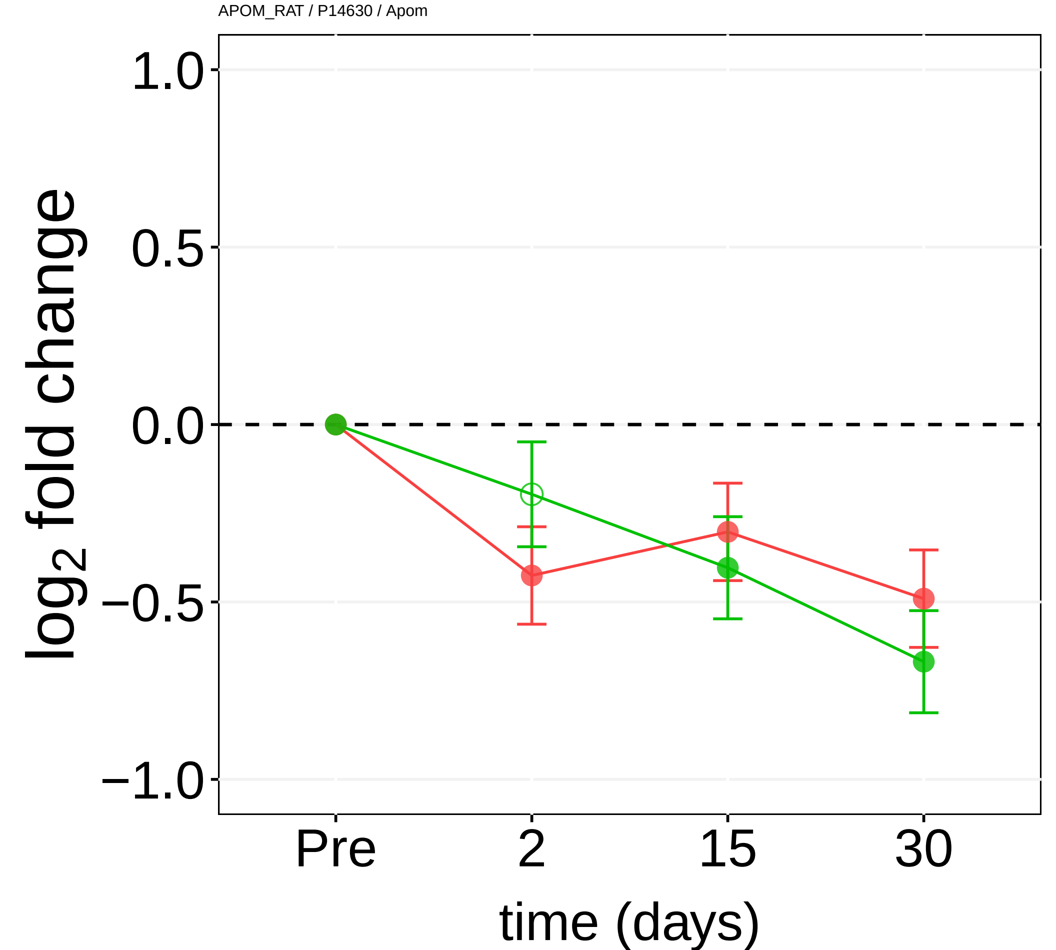
APOM_RAT / P14630 / Apom
1.0
0.5
0.0
−0.5
−1.0
log2 fold change
Pre
2
15
30
time (days)

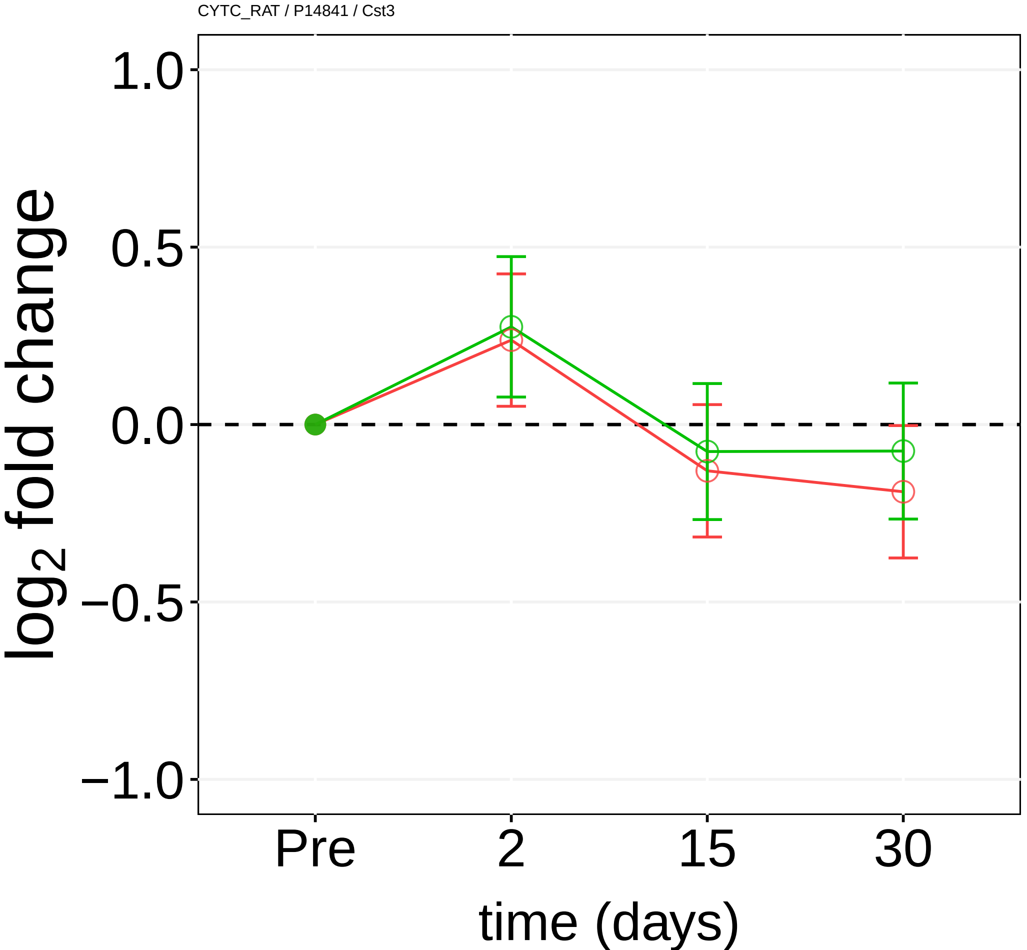
CYTC_RAT / P14841 / Cst3
1.0
0.5
0.0
−0.5
−1.0
log2 fold change
Pre
2
15
30
time (days)

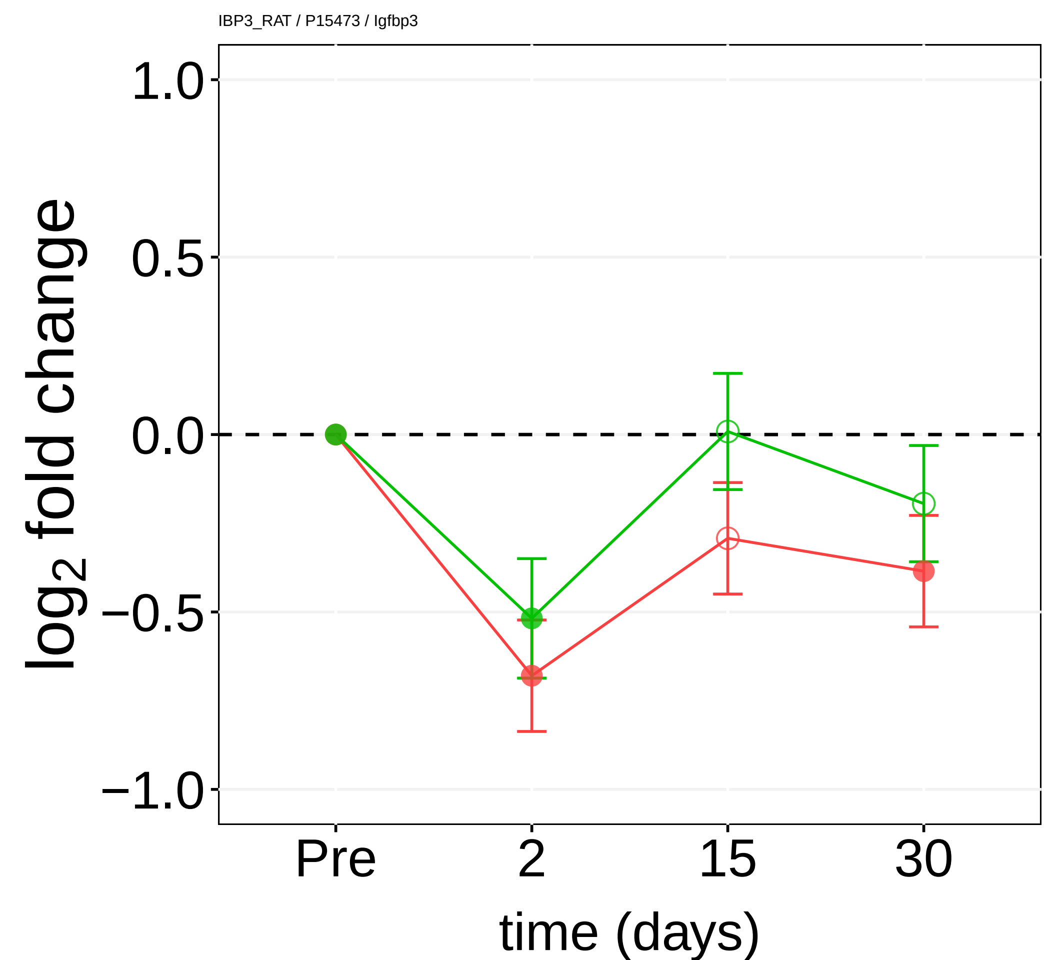
IBP3_RAT / P15473 / Igfbp3
1.0
0.5
0.0
−0.5
−1.0
log2 fold change
Pre
2
15
30
time (days)

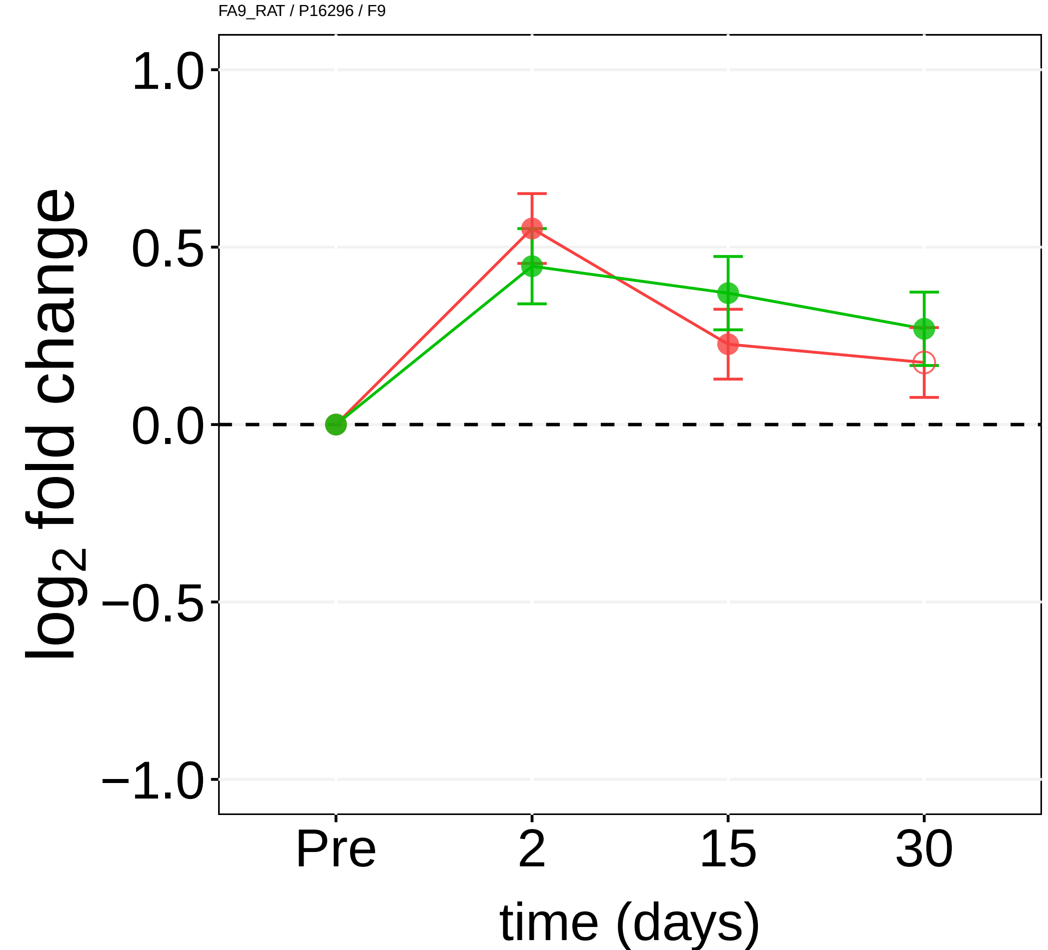

FA9_RAT / P16296 / F9
1.0
0.5
0.0
−0.5
−1.0
log2 fold change
Pre
2
15
30
time (days)

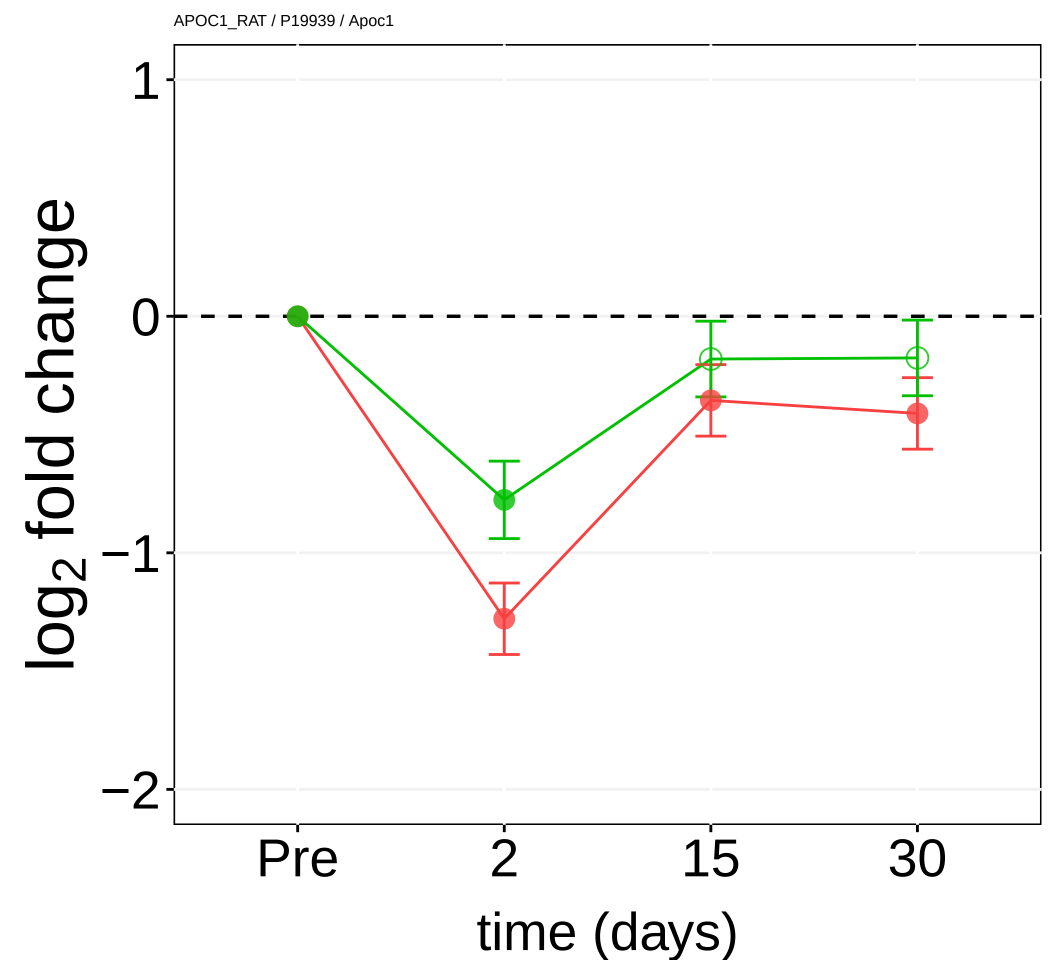
APOC1_RAT / P19939 / Apoc1
1
0
−1
−2
log2 fold change
Pre
2
15
30
time (days)

MBL1_RAT / P19999 / Mbl1

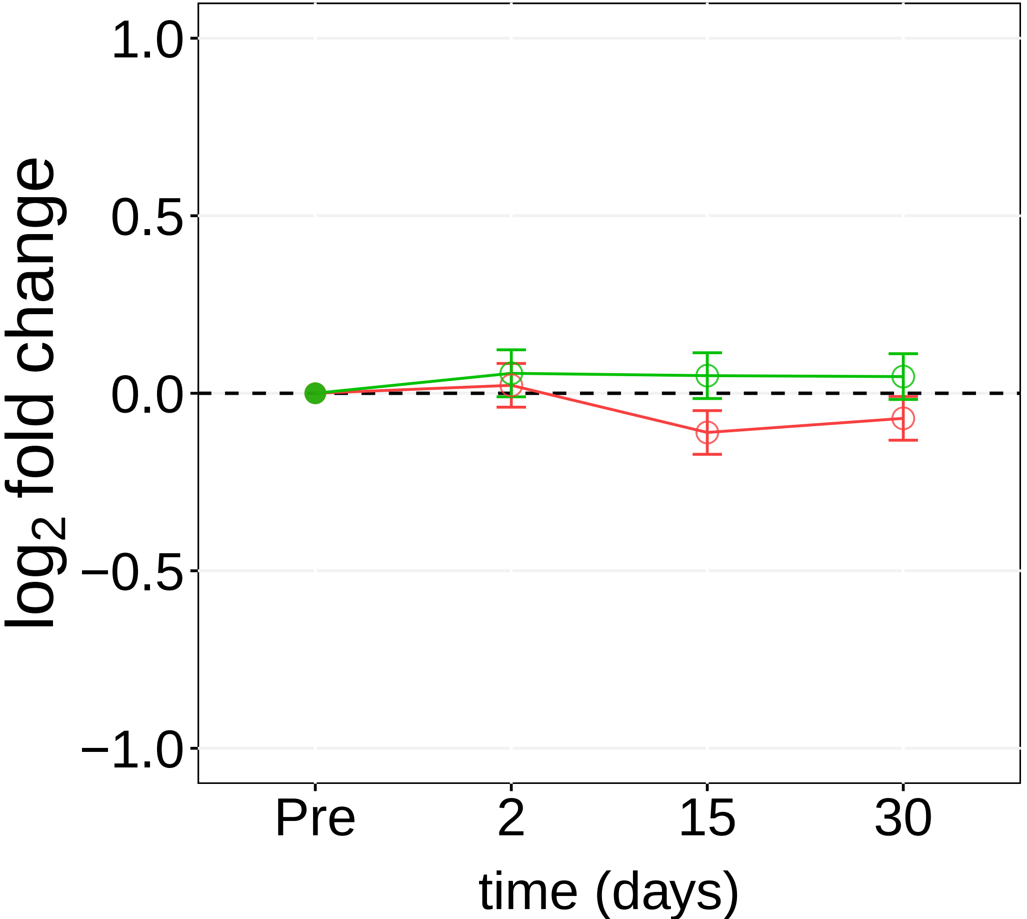

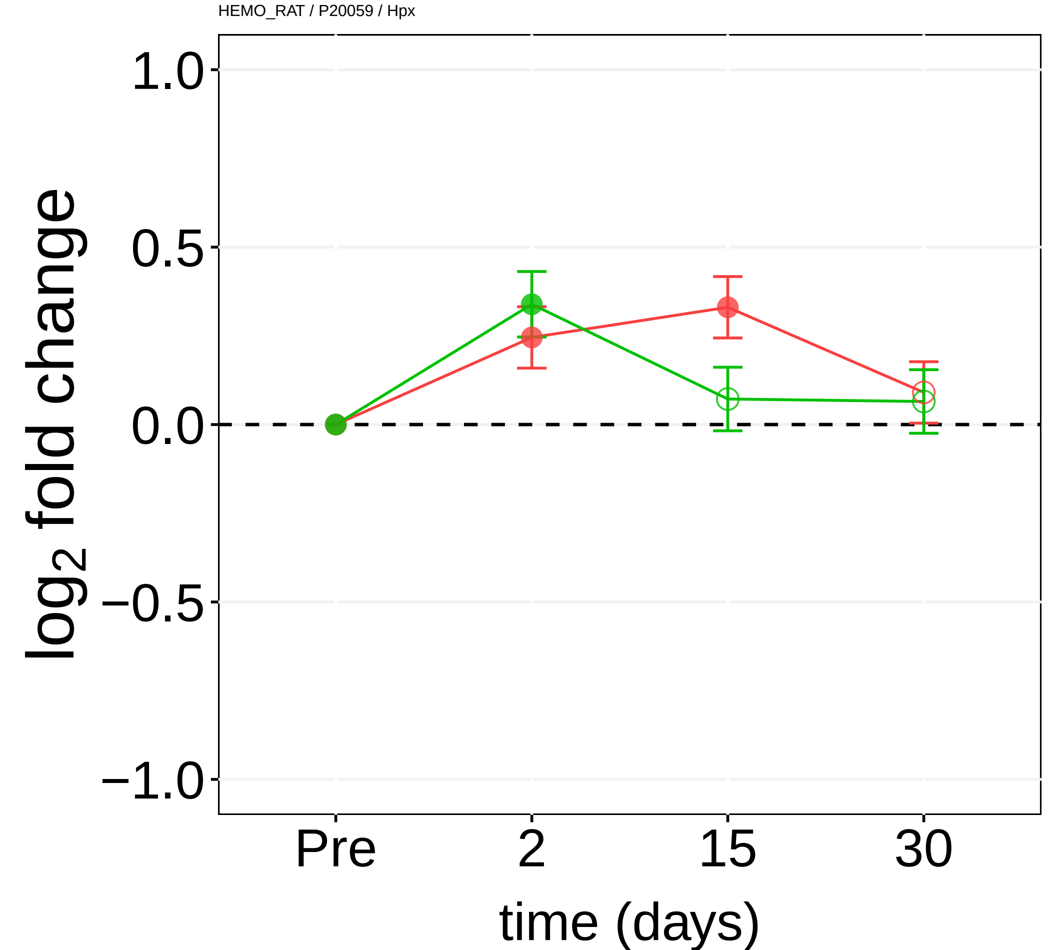
HEMO_RAT / P20059 / Hpx
1.0
0.5
0.0
−0.5
−1.0
log2 fold change
Pre
2
15
30
time (days)

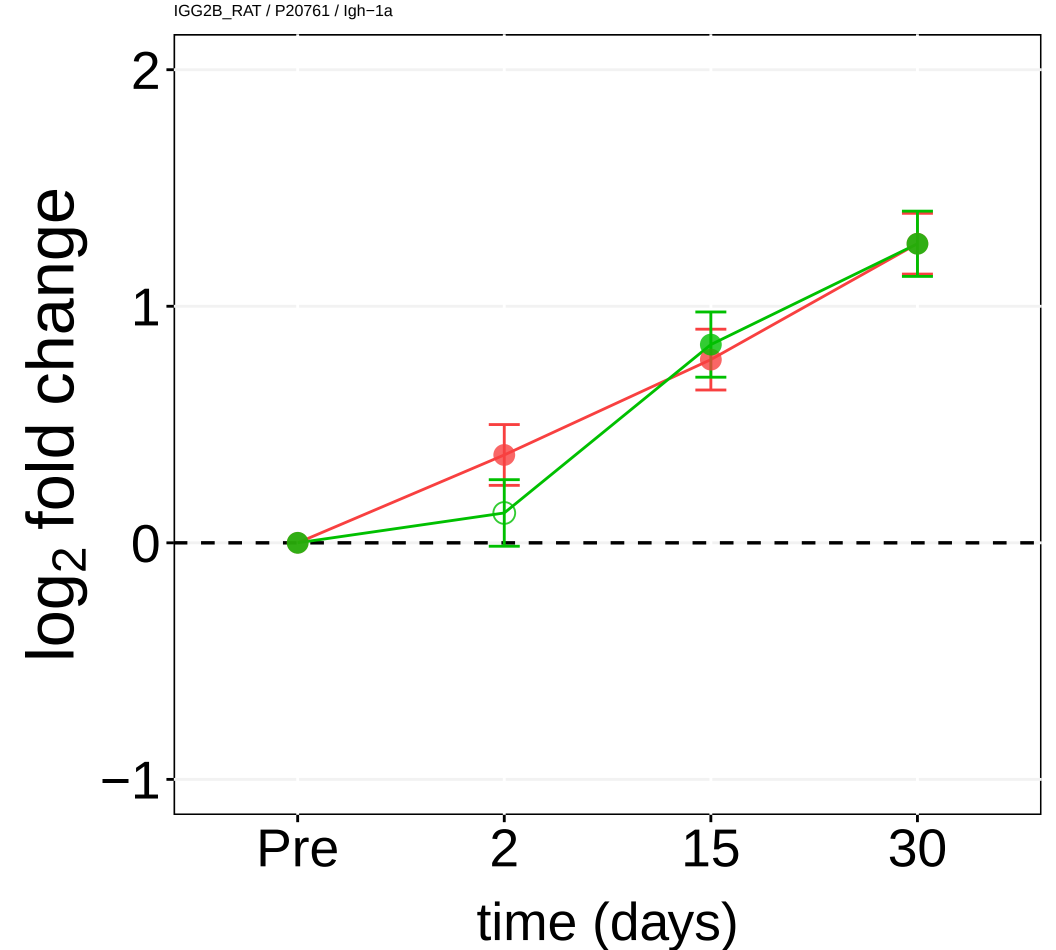
IGG2B_RAT / P20761 / Igh−1a
log2 fold change
2
1
0
−1
Pre
2
15
30
time (days)

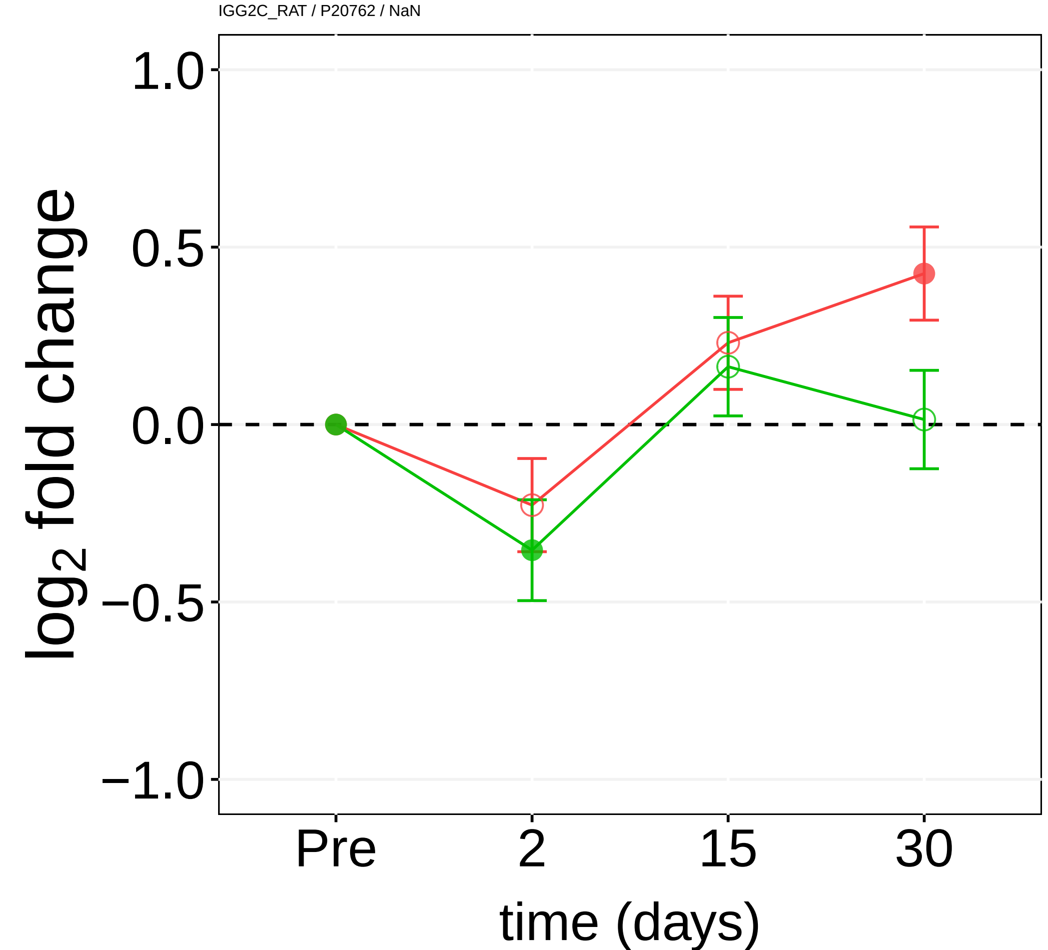
IGG2C_RAT / P20762 / NaN
1.0
0.5
0.0
−0.5
−1.0
log2 fold change
Pre
2
15
30
time (days)

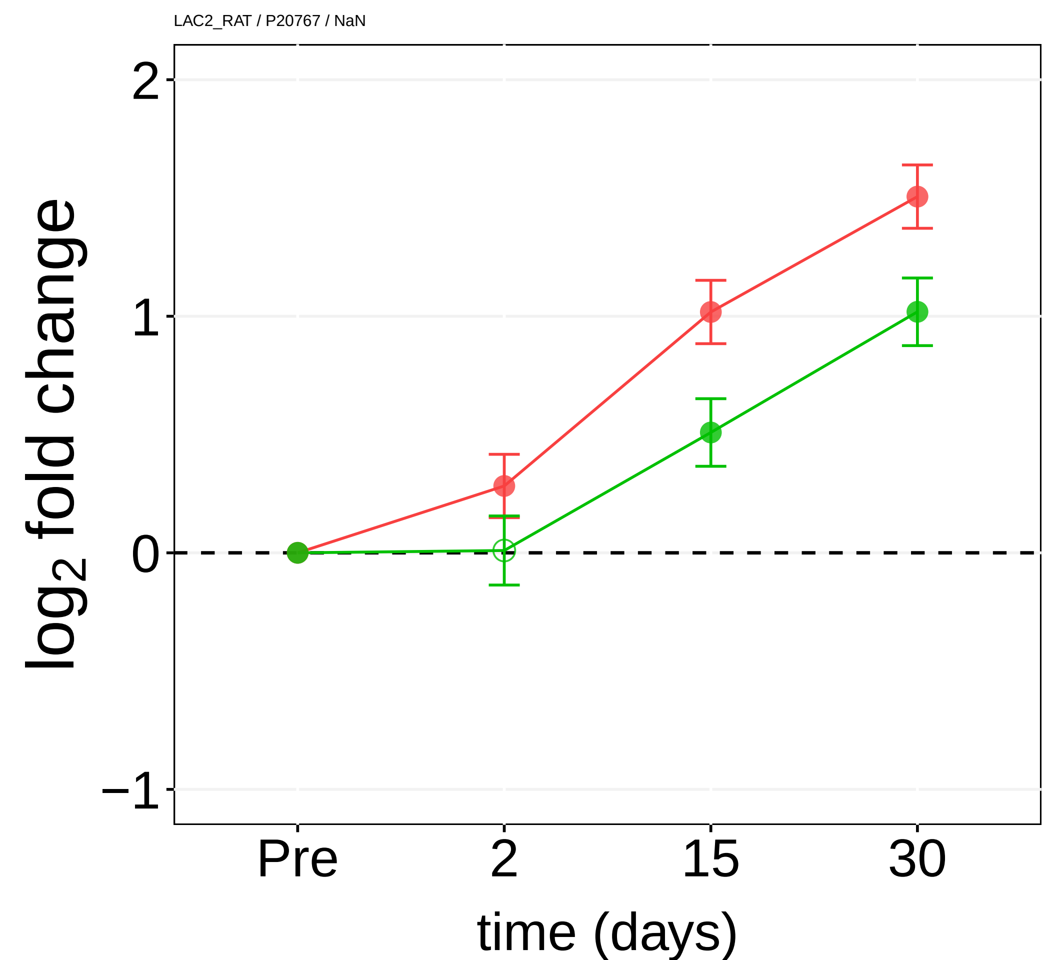
LAC2_RAT / P20767 / NaN
log2 fold change
2
1
0
−1
Pre
2
15
30
time (days)

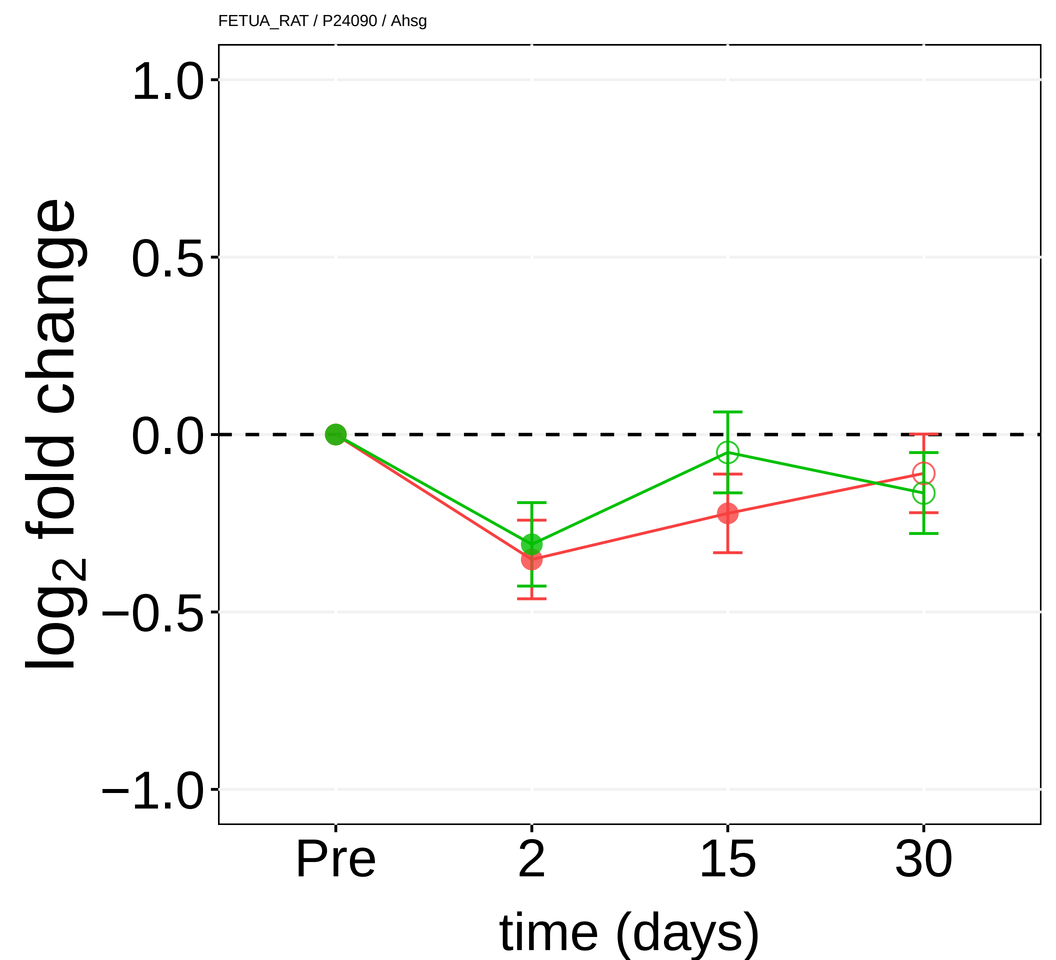
FETUA_RAT / P24090 / Ahsg
1.0
0.5
0.0
−0.5
−1.0
log2 fold change
Pre
2
15
30
time (days)

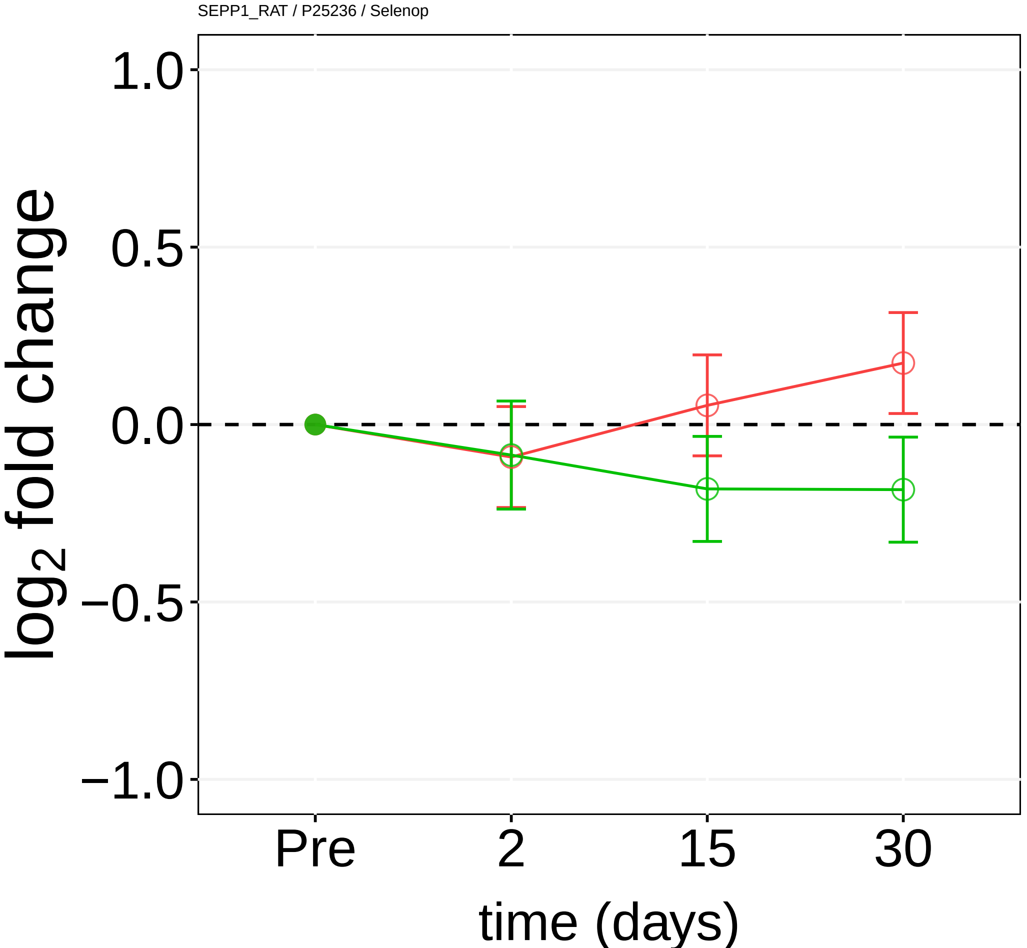
SEPP1_RAT / P25236 / Selenop
1.0
0.5
0.0
−0.5
−1.0
log2 fold change
Pre
2
15
30
time (days)

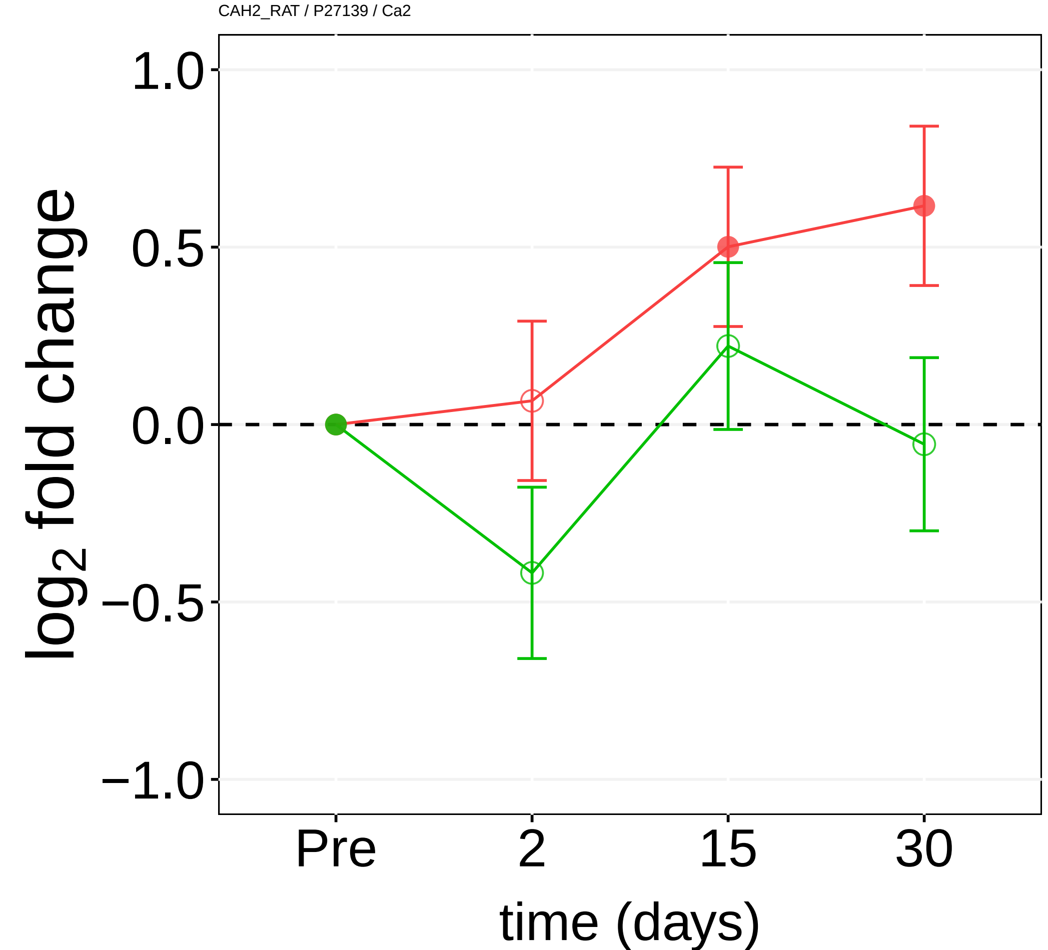
CAH2_RAT / P27139 / Ca2
1.0
0.5
0.0
−0.5
−1.0
log2 fold change
Pre
2
15
30
time (days)

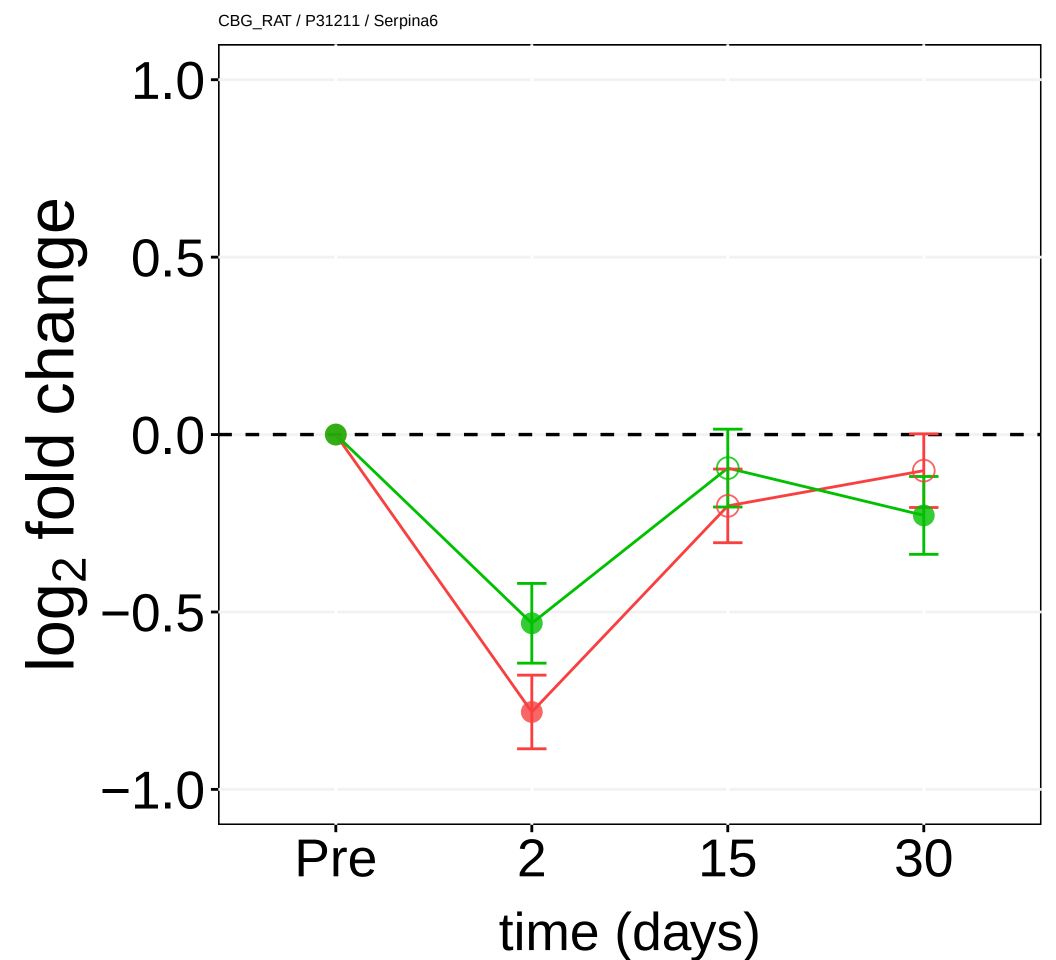

CBG_RAT / P31211 / Serpina6
1.0
0.5
0.0
−0.5
−1.0
log2 fold change
Pre
2
15
30
time (days)

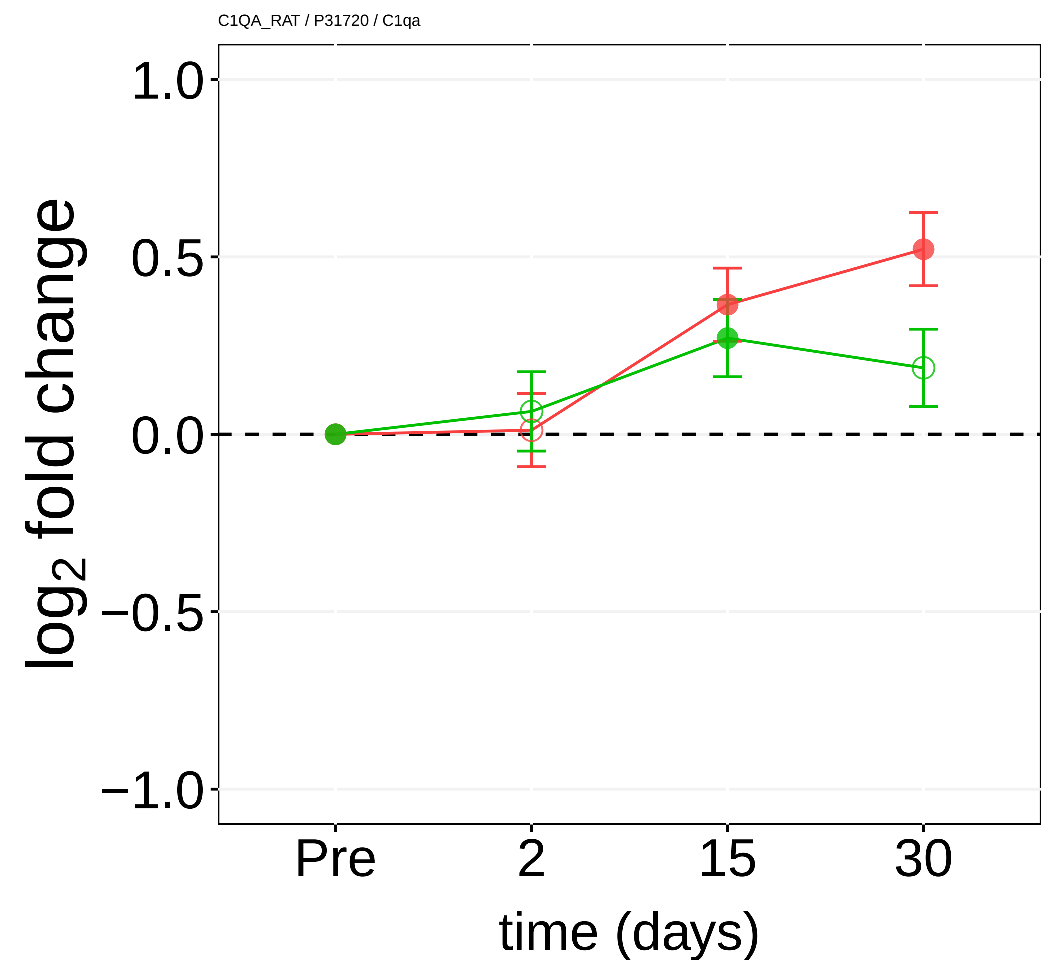
C1QA_RAT / P31720 / C1qa
1.0
0.5
0.0
−0.5
−1.0
log2 fold change
Pre
2
15
30
time (days)

CFAD_RAT / P32038 / Cfd

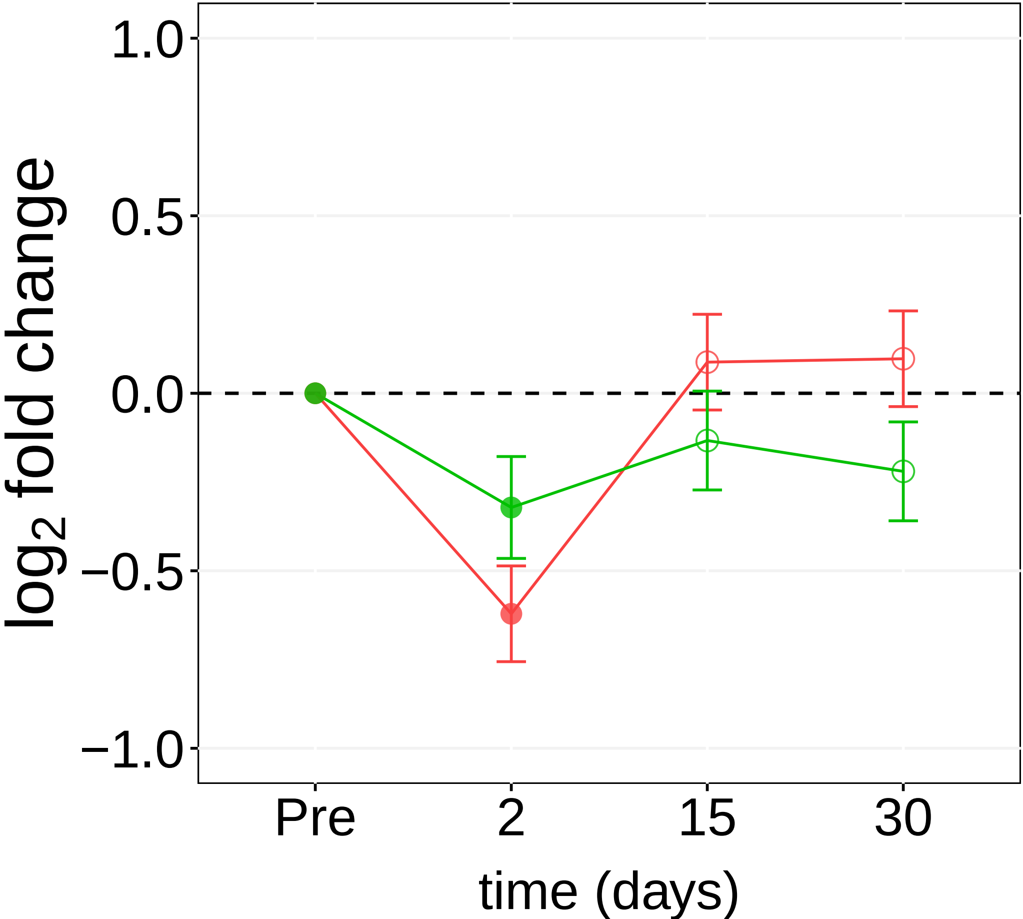

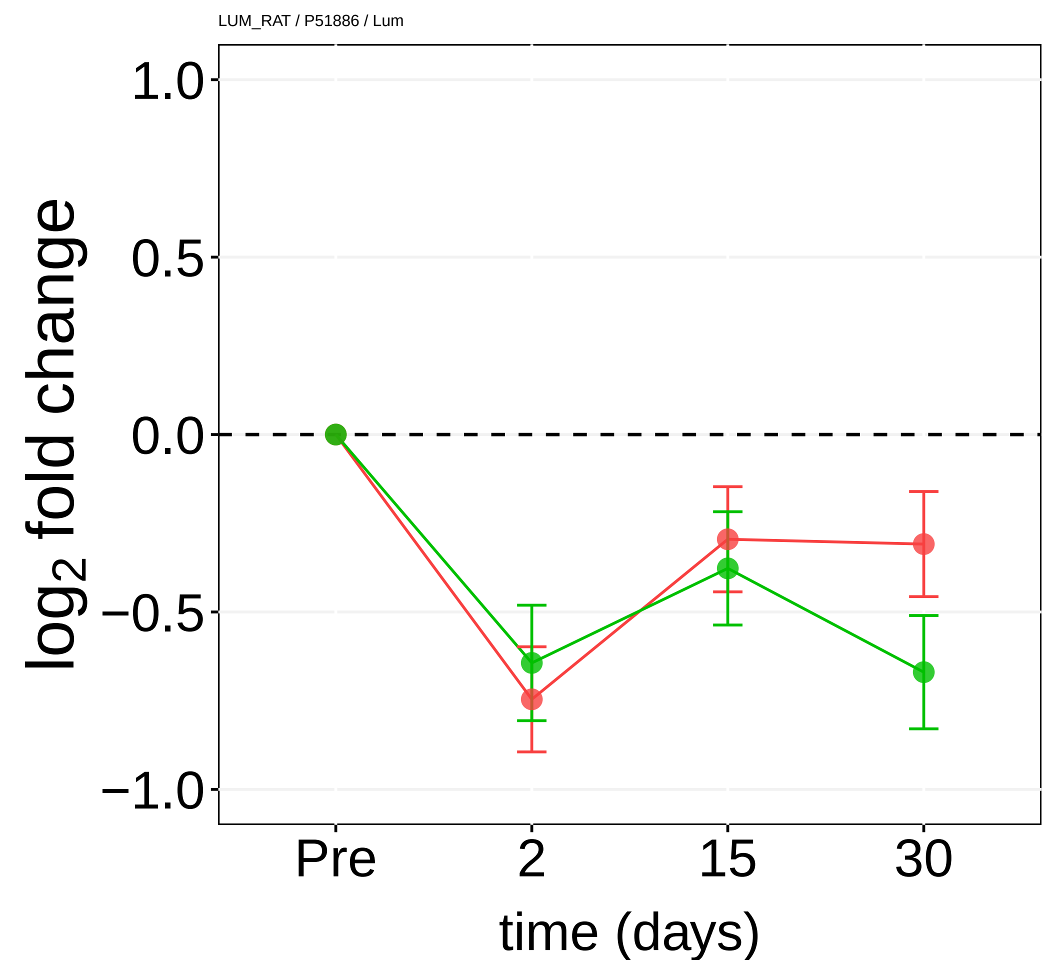

LUM_RAT / P51886 / Lum
log2 fold change
1.0
0.5
0.0
−0.5
−1.0
Pre
2
15
30
time (days)

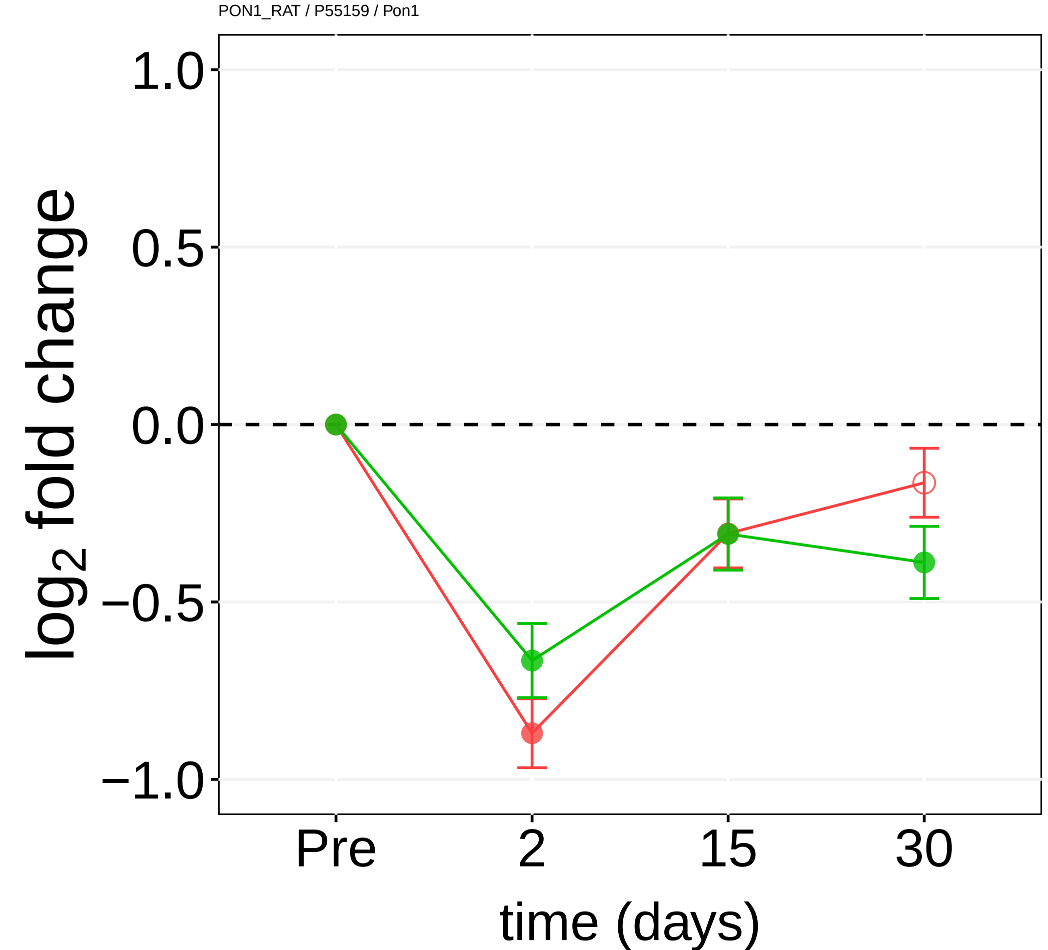

PON1_RAT / P55159 / Pon1
1.0
0.5
0.0
−0.5
−1.0
$\log_2$ fold change
Pre
2
15
30
time (days)

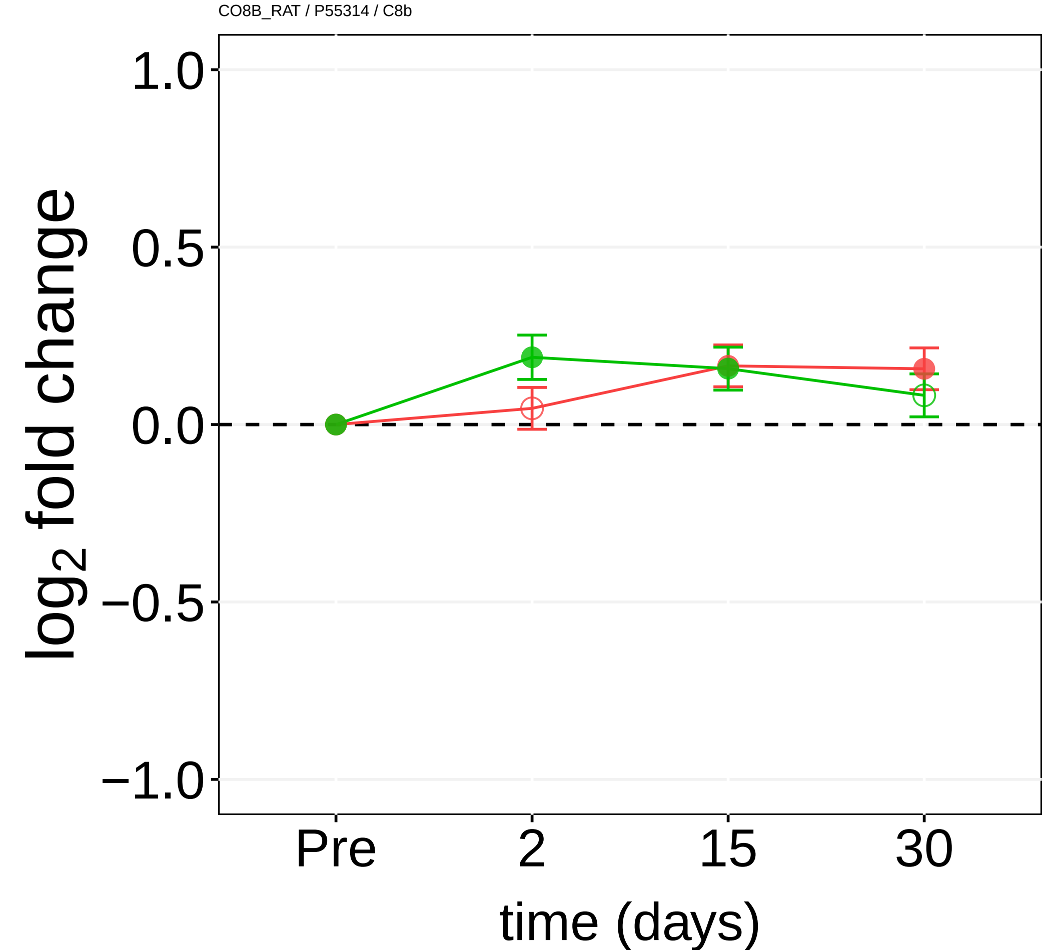

CO8B_RAT / P55314 / C8b
1.0
0.5
0.0
−0.5
−1.0
log2 fold change
Pre
2
15
30
time (days)

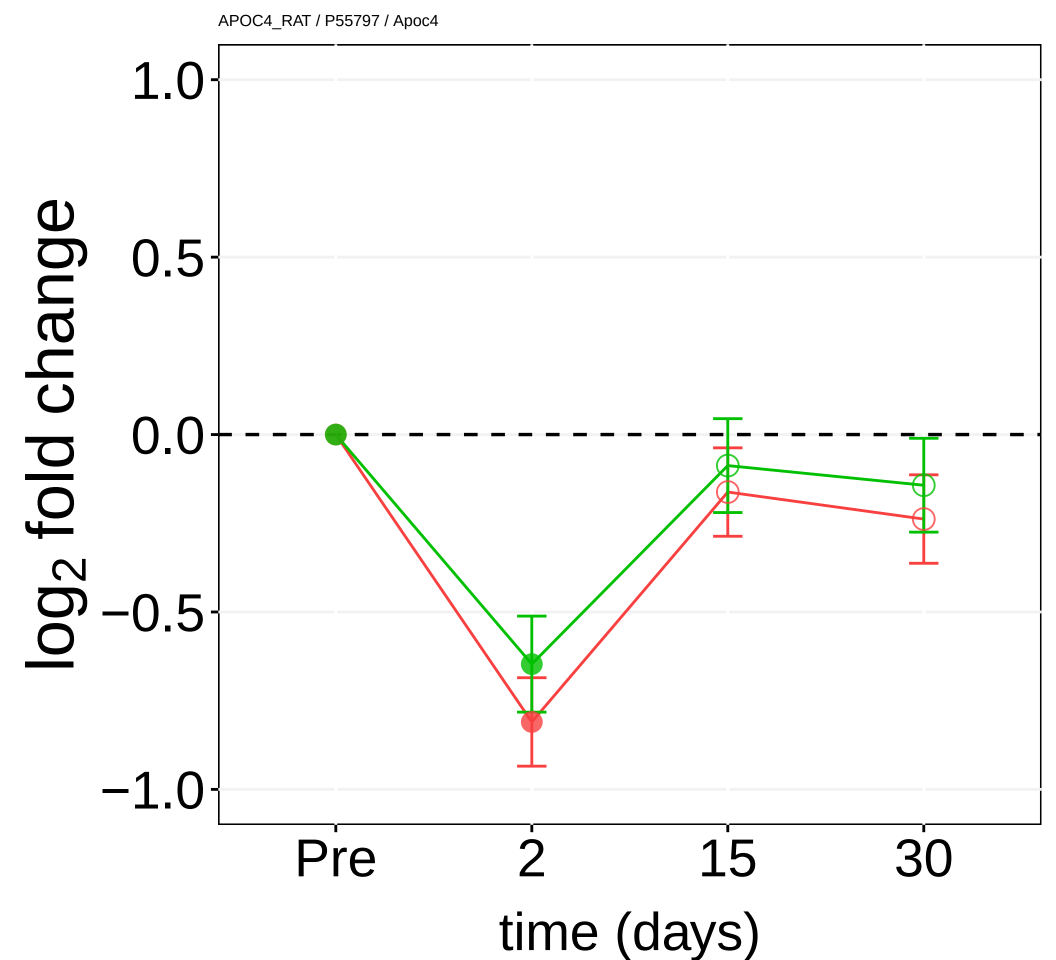

APOC4_RAT / P55797 / Apoc4
1.0
0.5
0.0
−0.5
−1.0
log2 fold change
Pre
2
15
30
time (days)

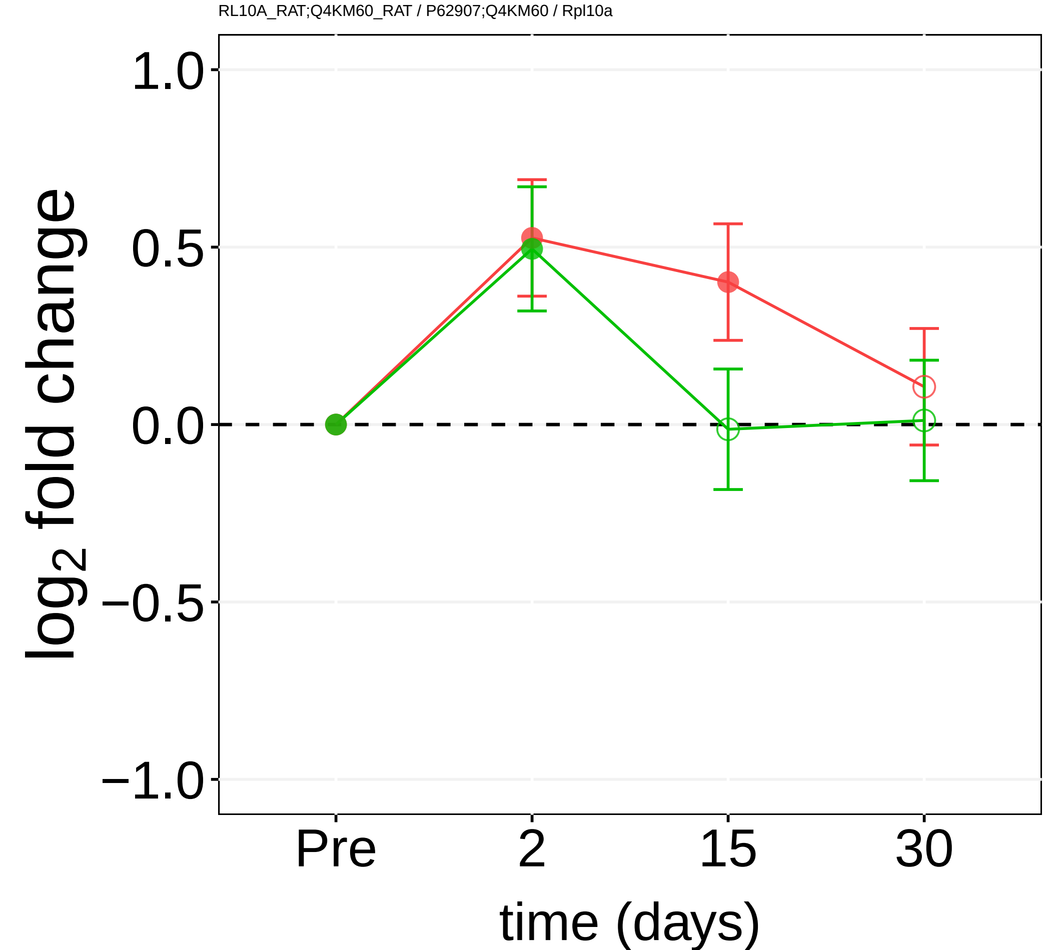
RL10A_RAT;Q4KM60_RAT / P62907;Q4KM60 / Rpl10a
log2 fold change
1.0
0.5
0.0
−0.5
−1.0
Pre
2
15
30
time (days)

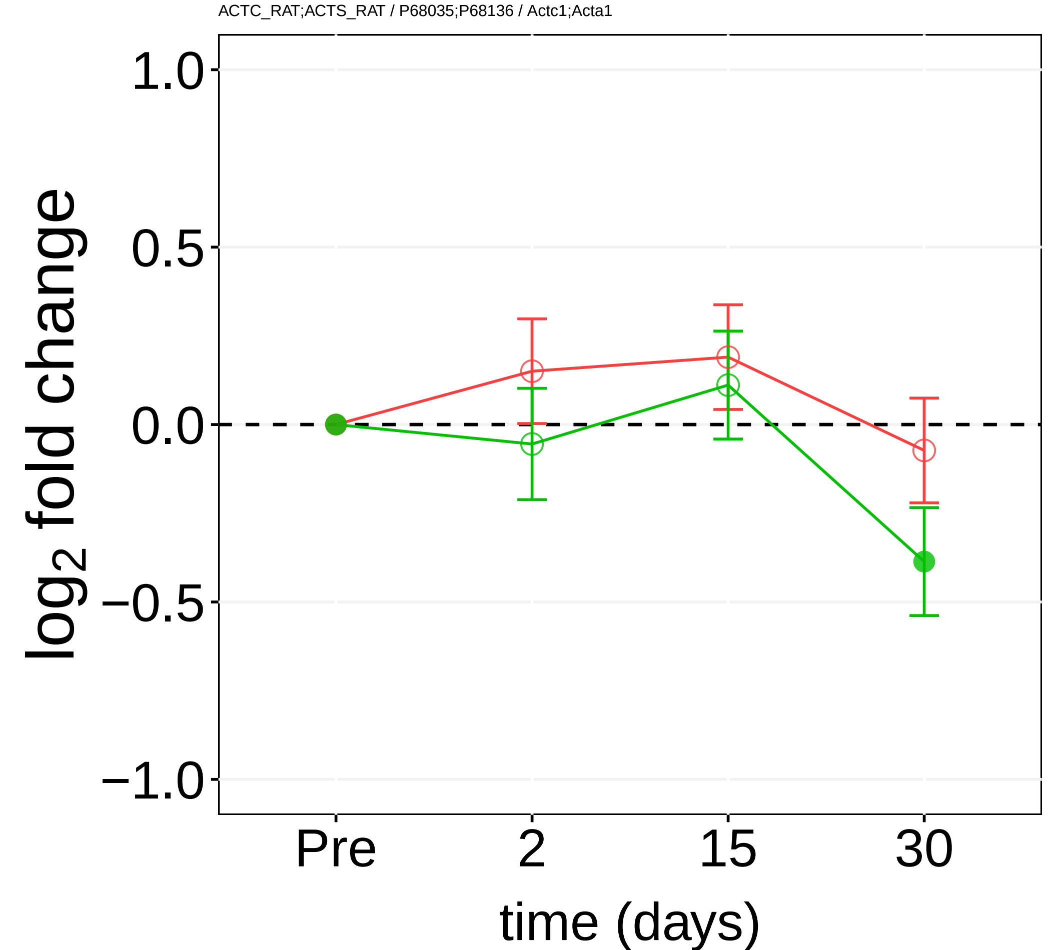
ACTC_RAT;ACTS_RAT / P68035;P68136 / Actc1;Acta1
1.0
0.5
0.0
−0.5
−1.0
log2 fold change
Pre
2
15
30
time (days)

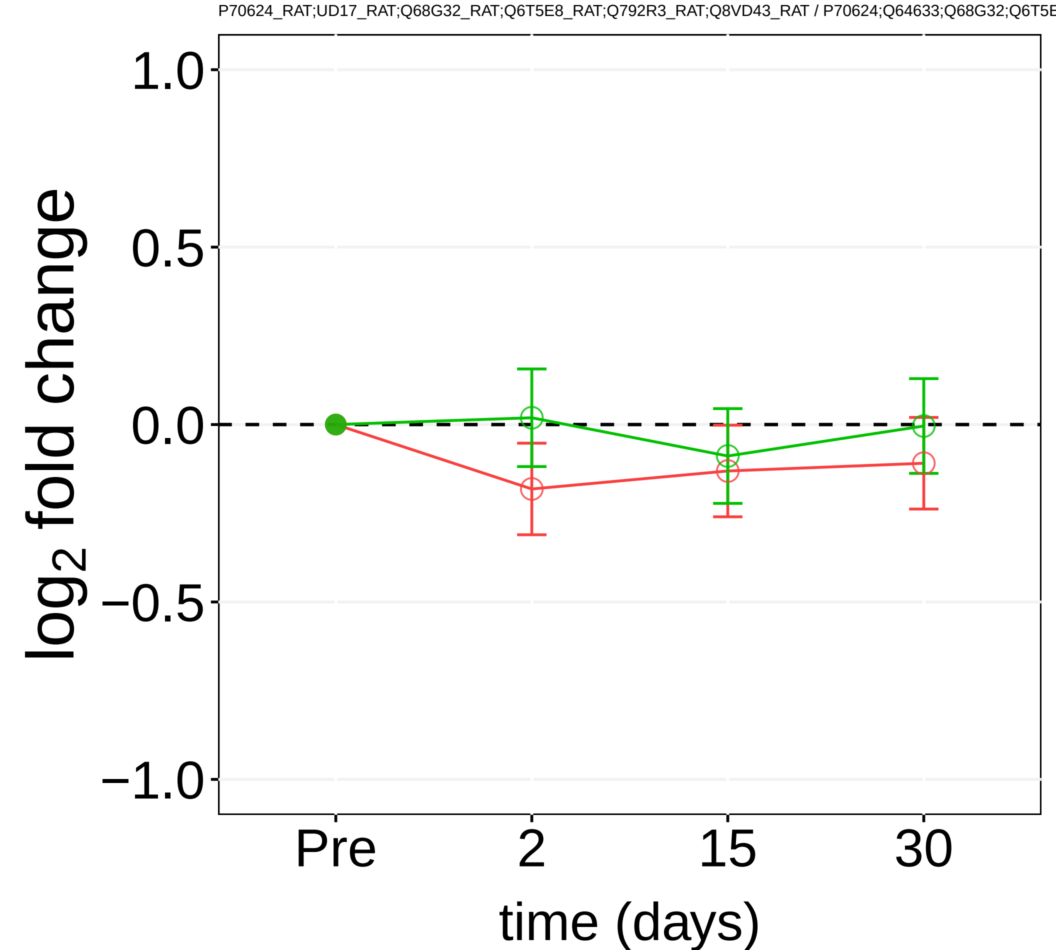

P70624_RAT;UD17_RAT;Q68G32_RAT;Q6T5E8_RAT;Q792R3_RAT;Q8VD43_RAT / P70624;Q64633;Q68G32;Q6T5E
1.0
0.5
0.0
−0.5
−1.0
log2 fold change
Pre
2
15
30
time (days)

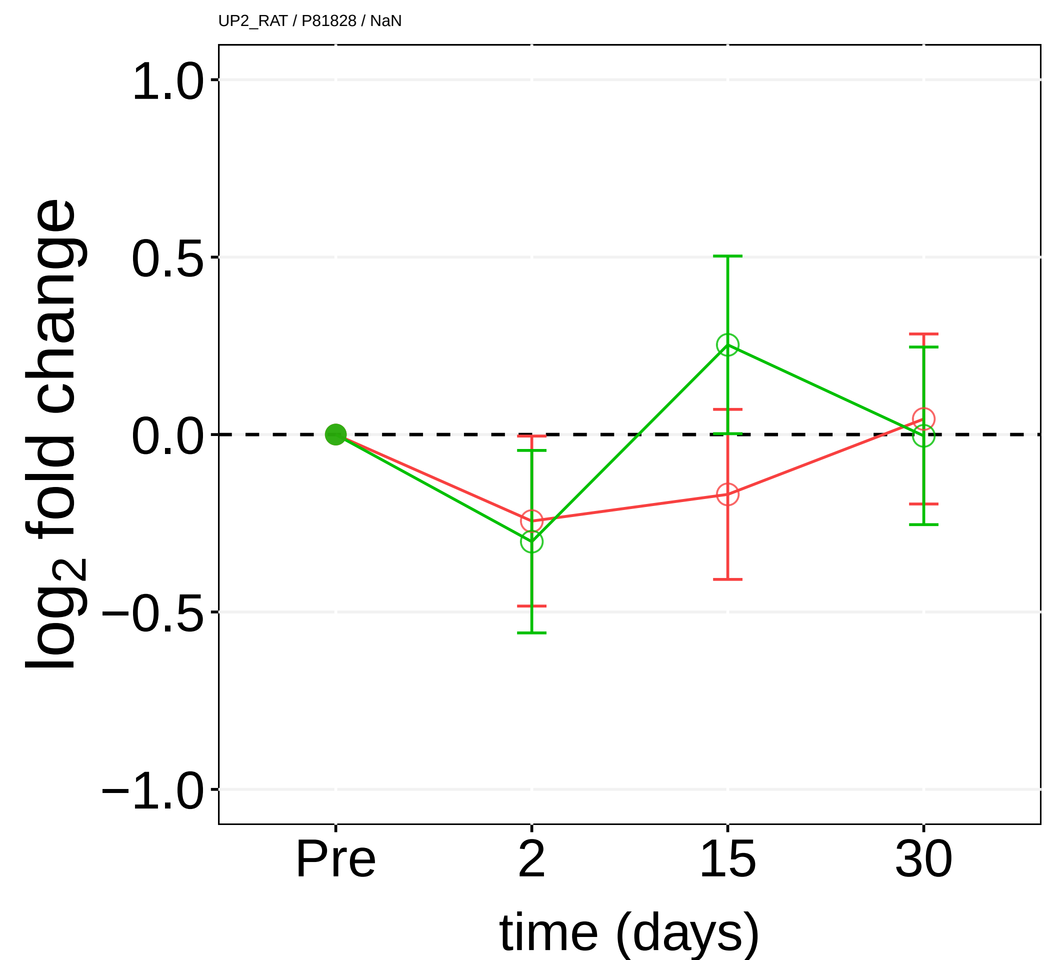

UP2_RAT / P81828 / NaN
1.0
0.5
0.0
−0.5
−1.0
log2 fold change
Pre
2
15
30
time (days)

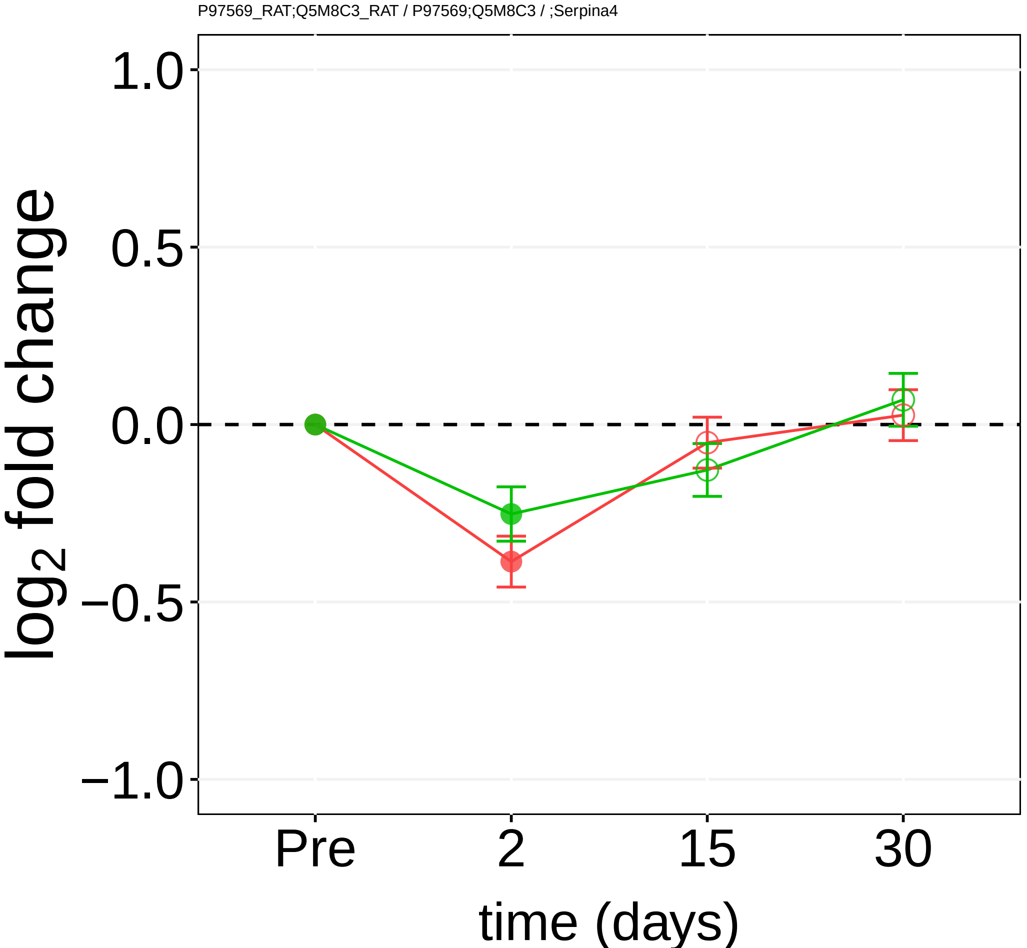
P97569_RAT;Q5M8C3_RAT / P97569;Q5M8C3 / ;Serpina4
log2 fold change
1.0
0.5
0.0
−0.5
−1.0
Pre
2
15
30
time (days)

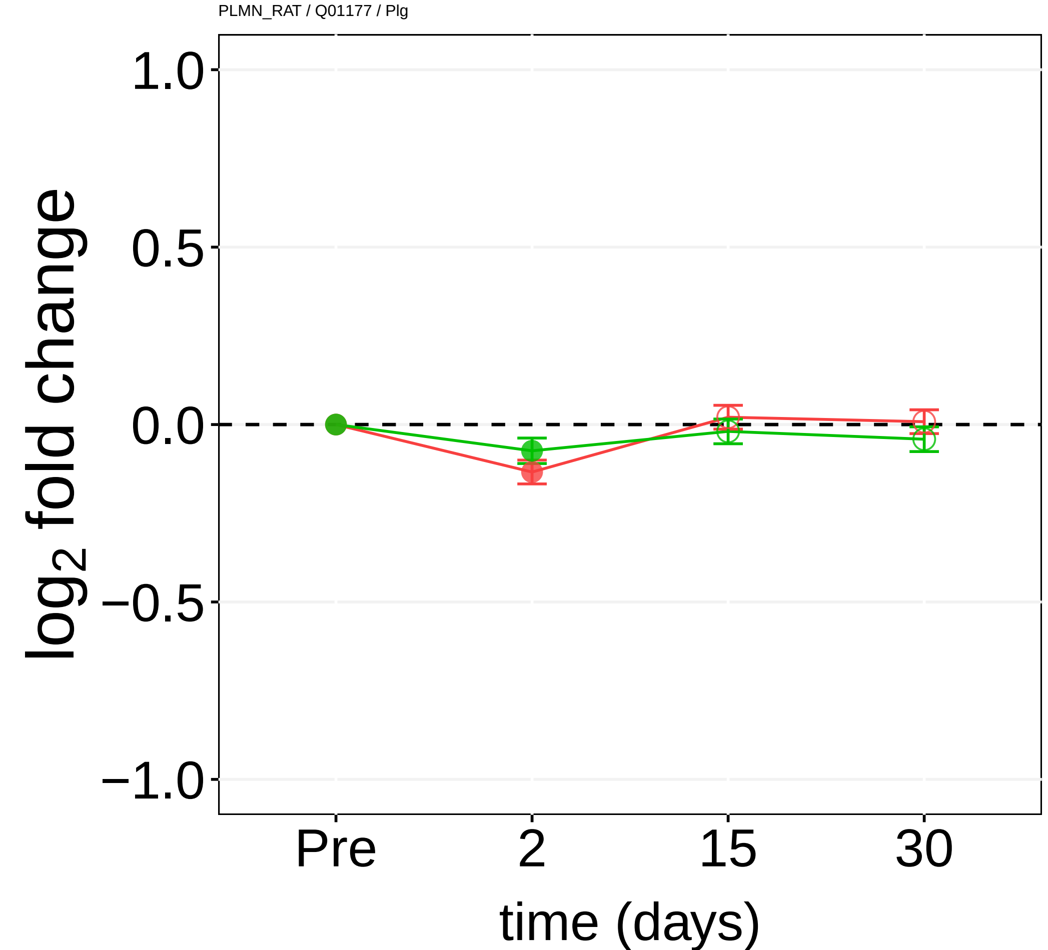
PLMN_RAT / Q01177 / Plg
1.0
0.5
0.0
−0.5
−1.0
log2 fold change
Pre
2
15
30
time (days)

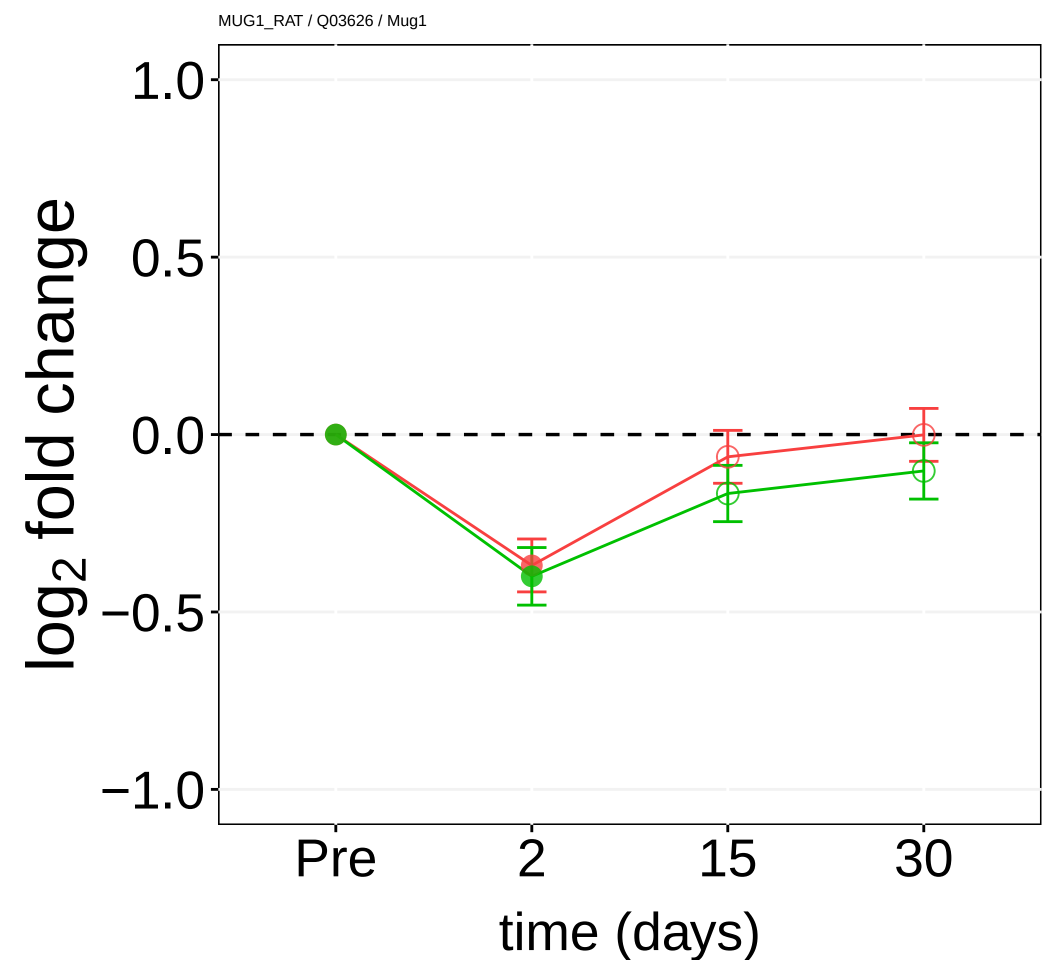

MUG1_RAT / Q03626 / Mug1
1.0
0.5
0.0
−0.5
−1.0
log2 fold change
Pre
2
15
30
time (days)

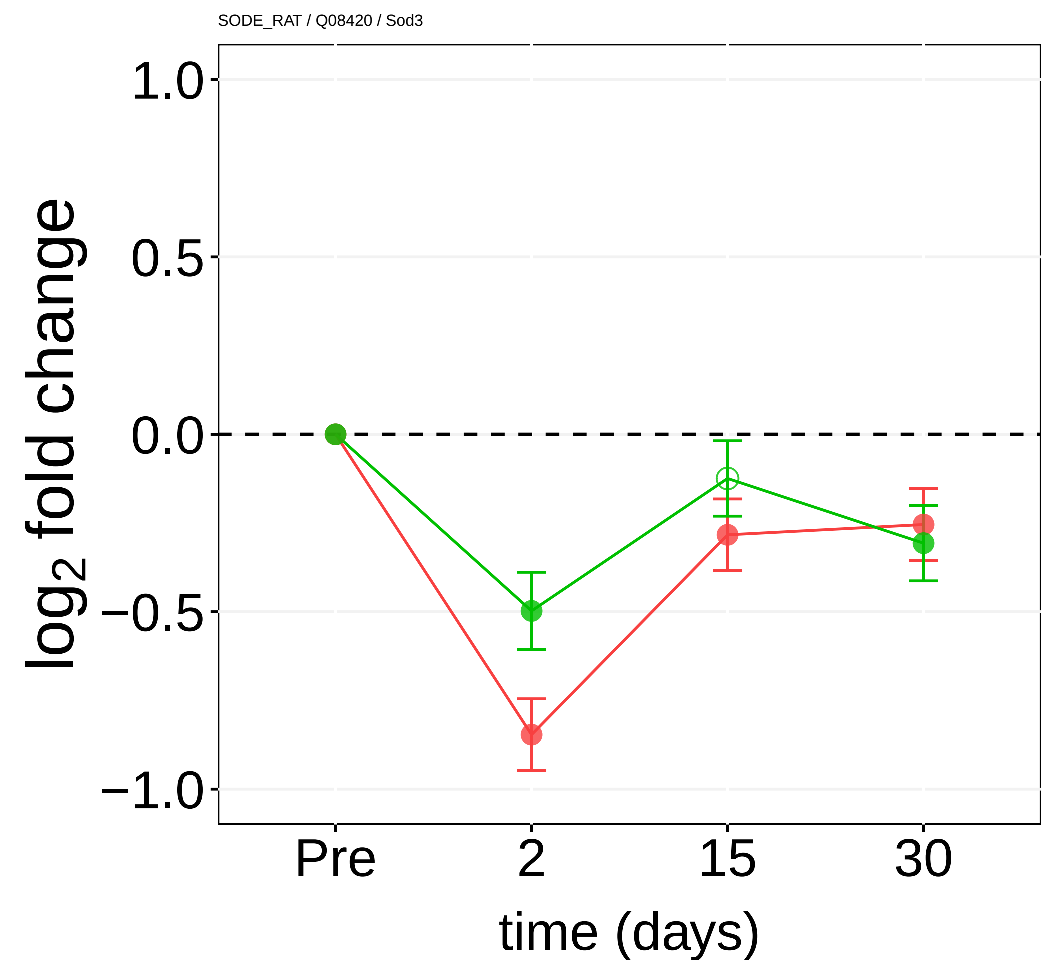
SODE_RAT / Q08420 / Sod3
1.0
0.5
0.0
−0.5
−1.0
log2 fold change
Pre
2
15
30
time (days)

CYTSA_RAT / Q2KN99 / Specc1l

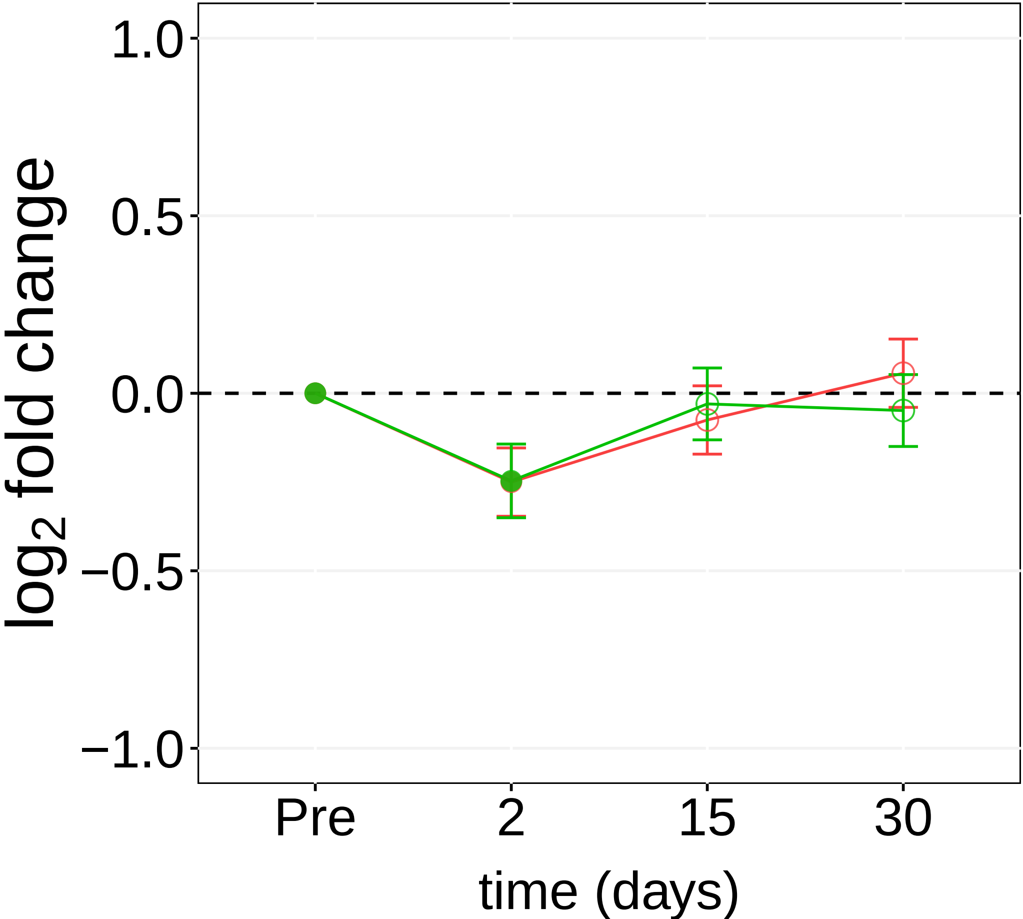

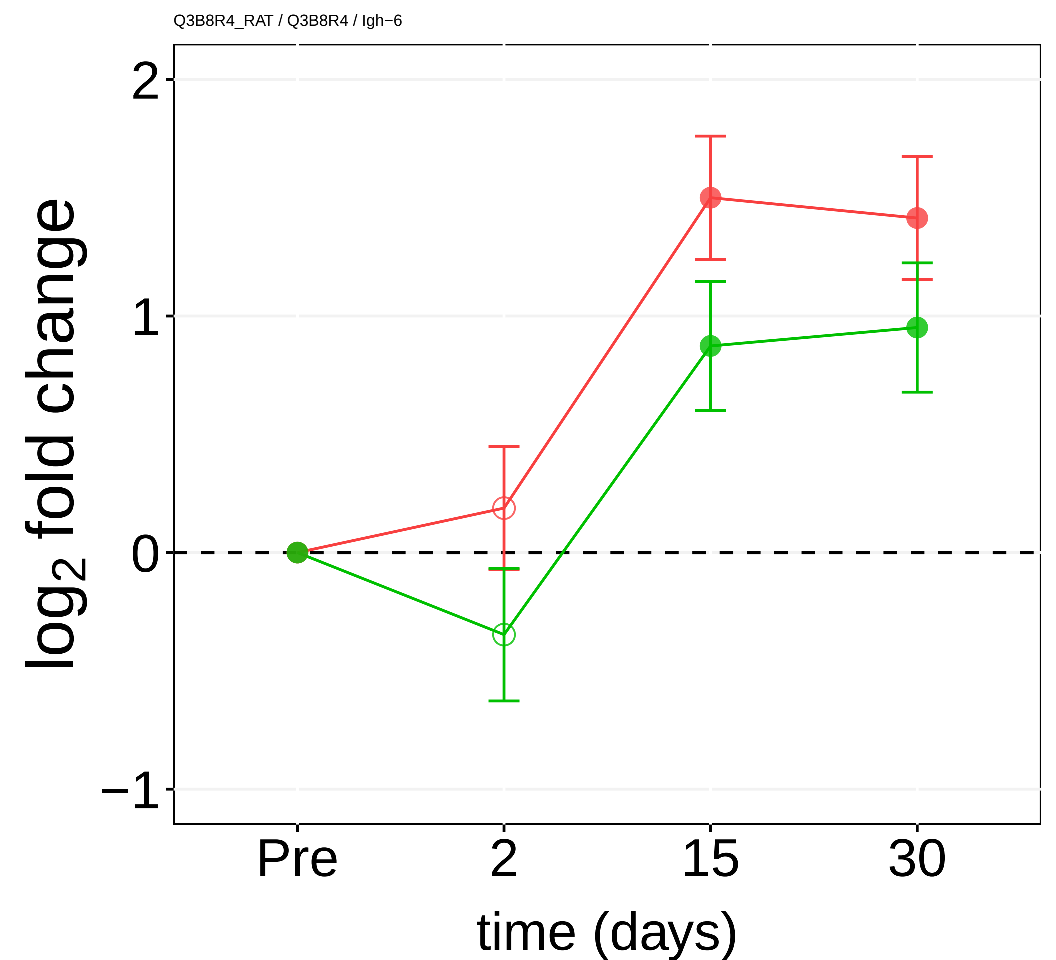

Q3B8R4_RAT / Q3B8R4 / Igh−6
2
1
0
−1
log2 fold change
Pre
2
15
30
time (days)

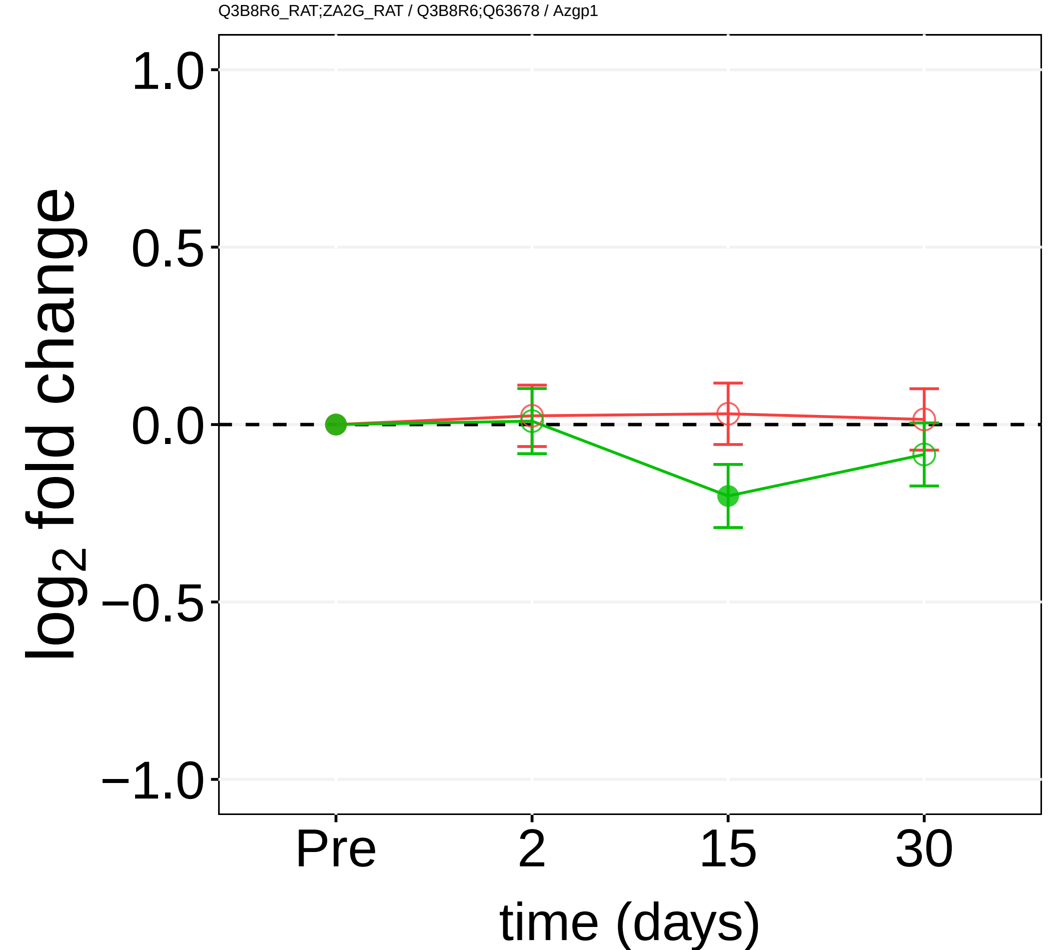

Q3B8R6_RAT;ZA2G_RAT / Q3B8R6;Q63678 / Azgp1
1.0
0.5
0.0
−0.5
−1.0
log2 fold change
Pre
2
15
30
time (days)

Q3KR94_RAT / Q3KR94 / Vtn

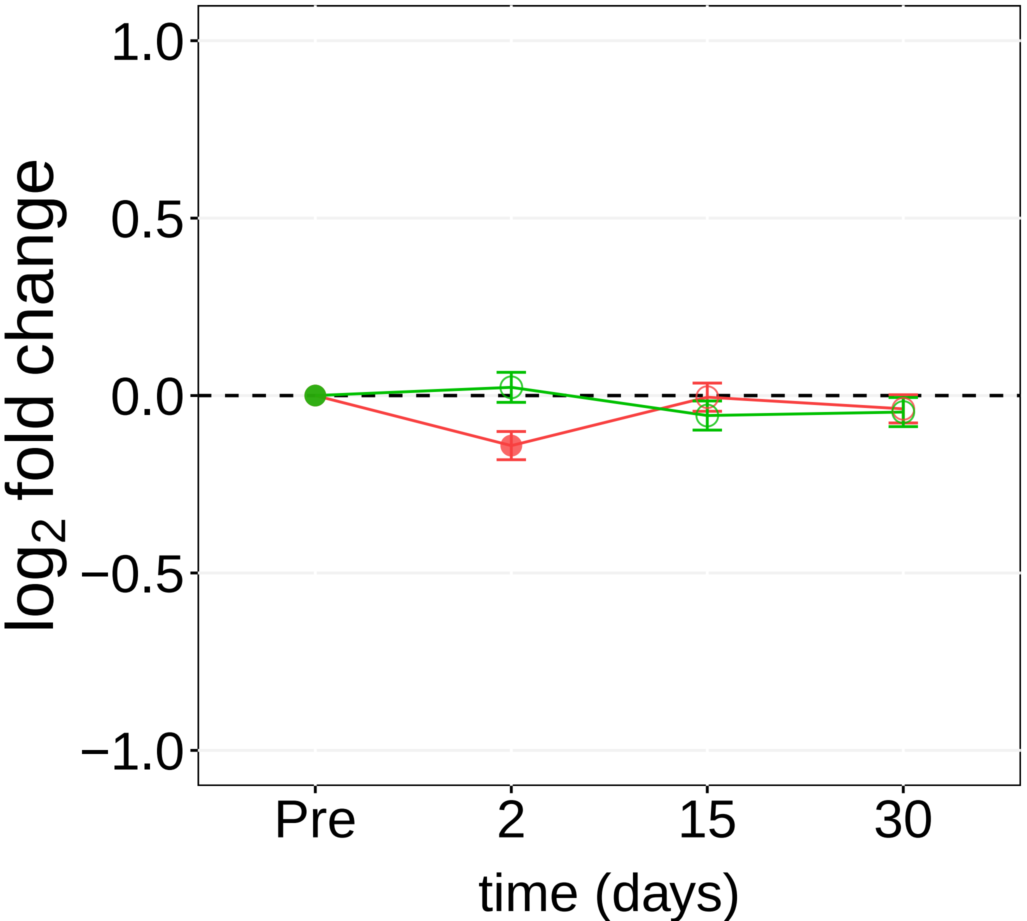

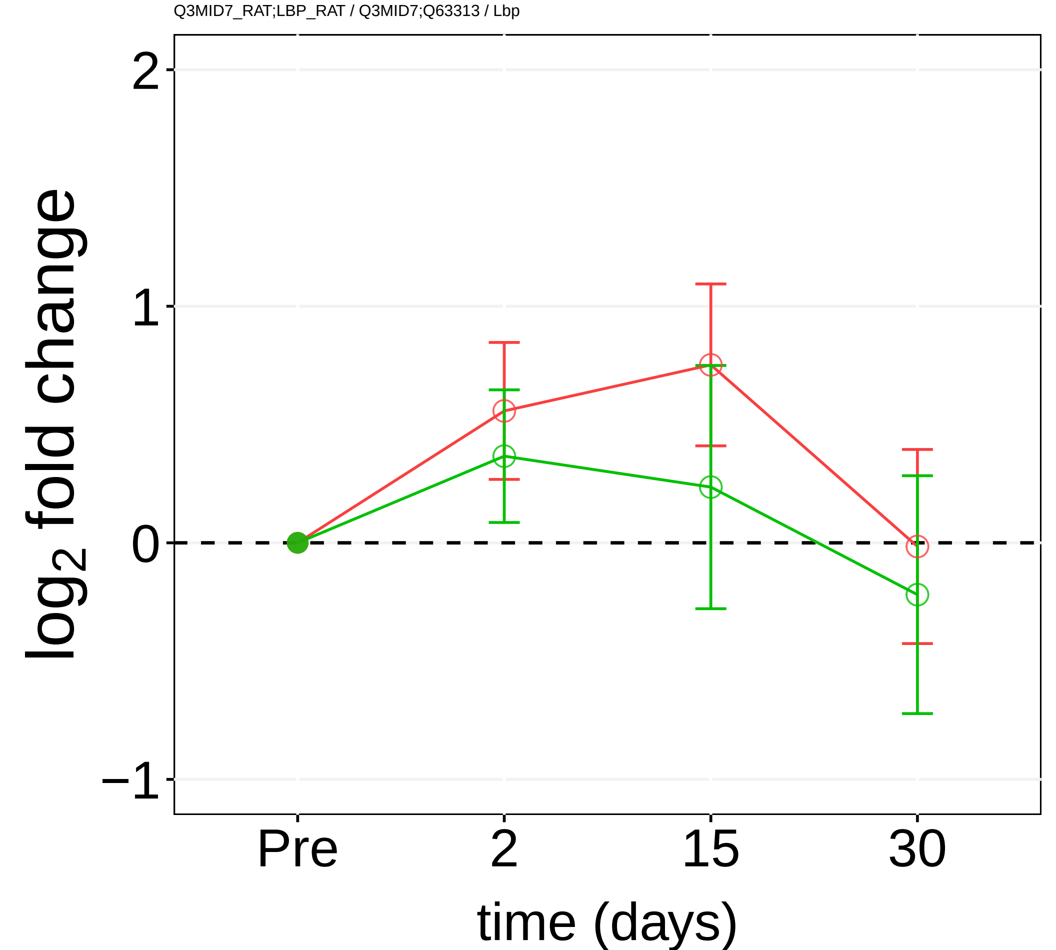

Q3MID7_RAT;LBP_RAT / Q3MID7;Q63313 / Lbp
2
1
0
−1
log2 fold change
Pre
2
15
30
time (days)

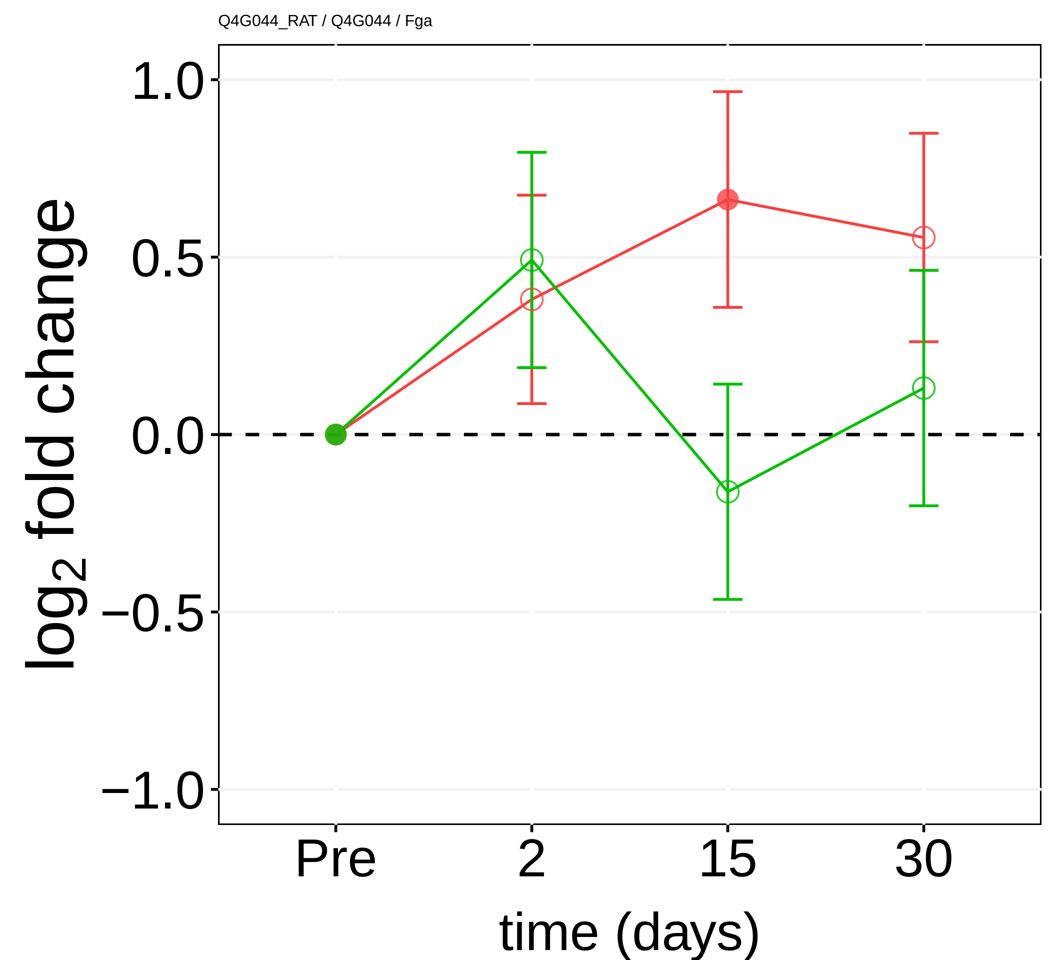
Q4G044_RAT / Q4G044 / Fga
log2 fold change
1.0
0.5
0.0
−0.5
−1.0
Pre
2
15
30
time (days)

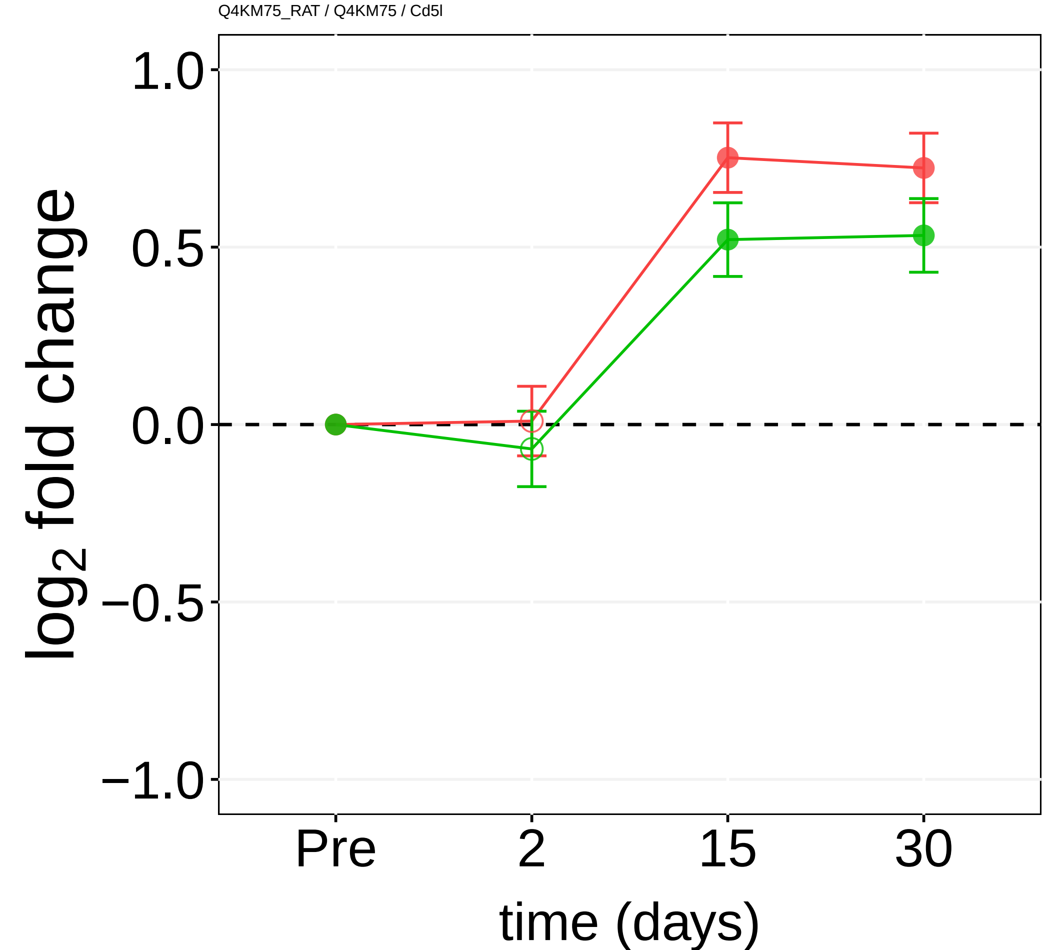
Q4KM75_RAT / Q4KM75 / Cd5l
1.0
0.5
0.0
−0.5
−1.0
log2 fold change
Pre
2
15
30
time (days)

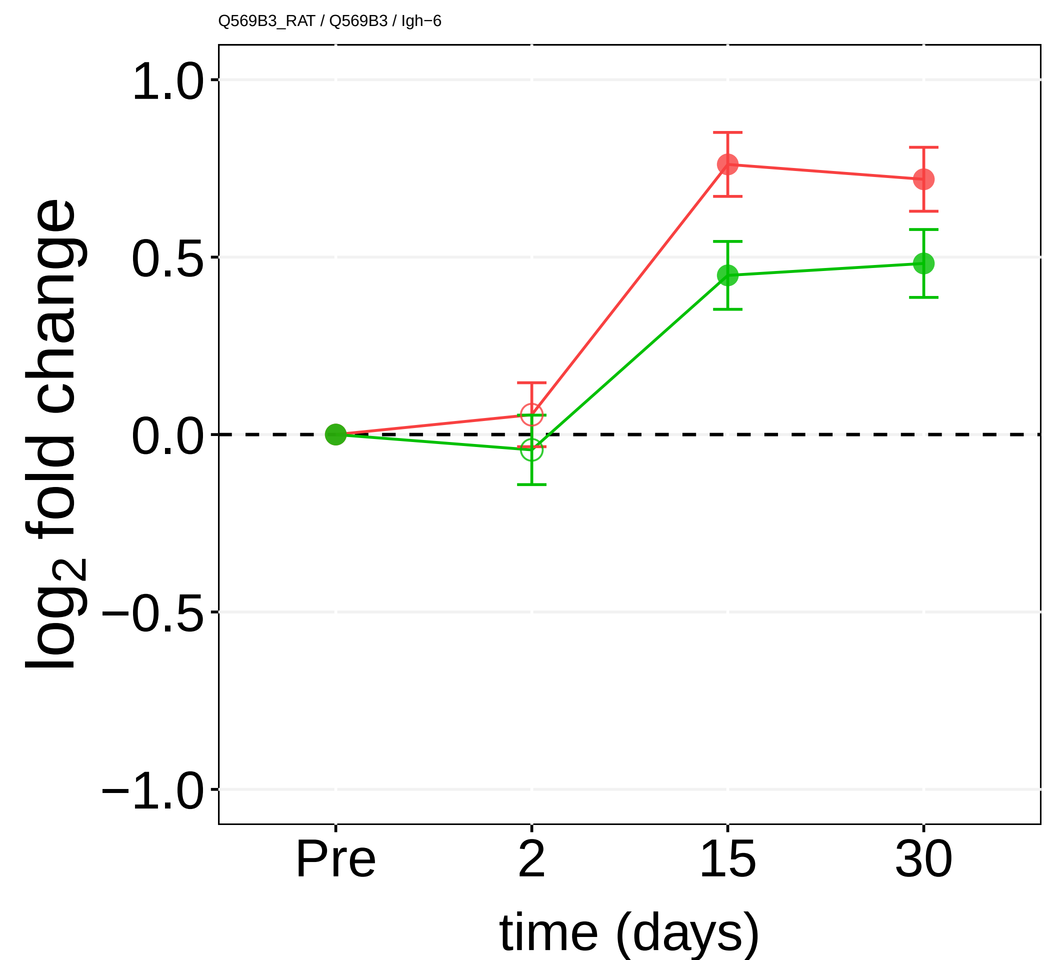
Q569B3_RAT / Q569B3 / Igh−6
log2 fold change
1.0
0.5
0.0
−0.5
−1.0
Pre
2
15
30
time (days)

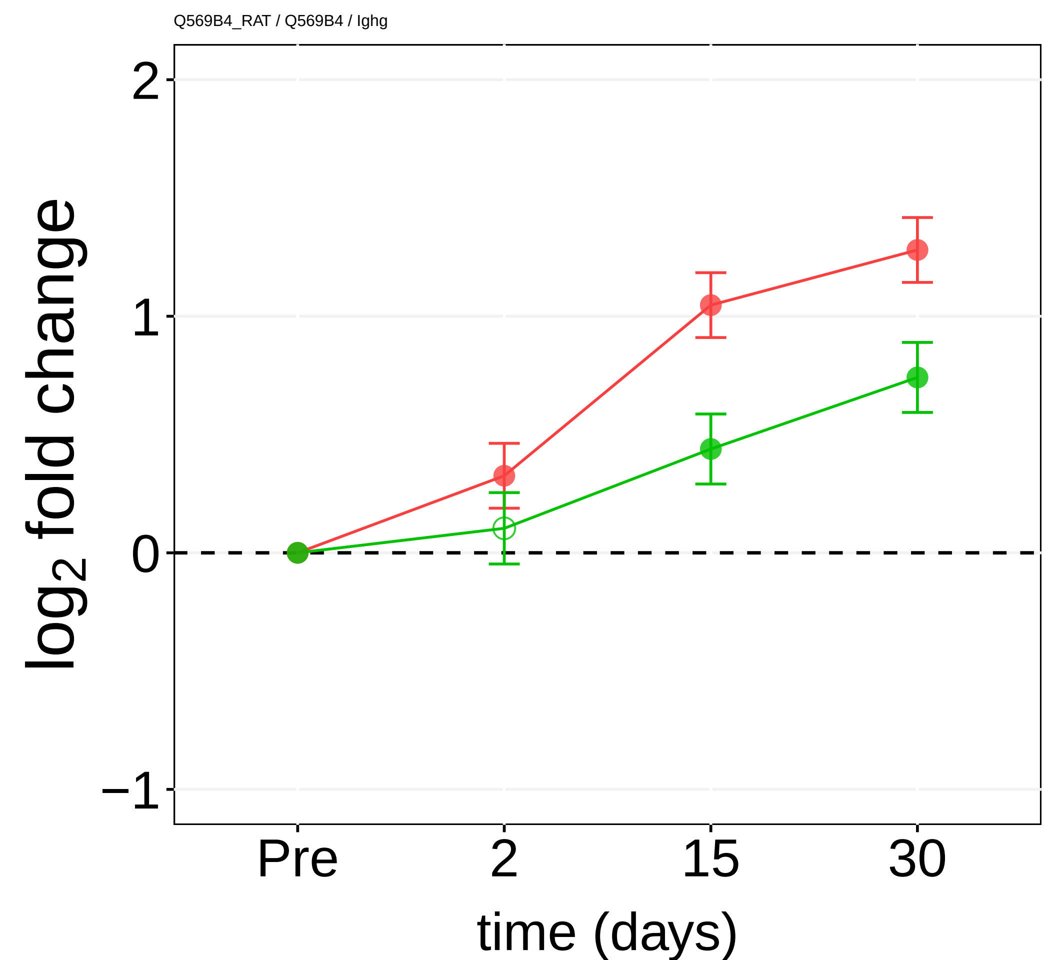
Q569B4_RAT / Q569B4 / Ighg
log2 fold change
2
1
0
−1
Pre
2
15
30
time (days)

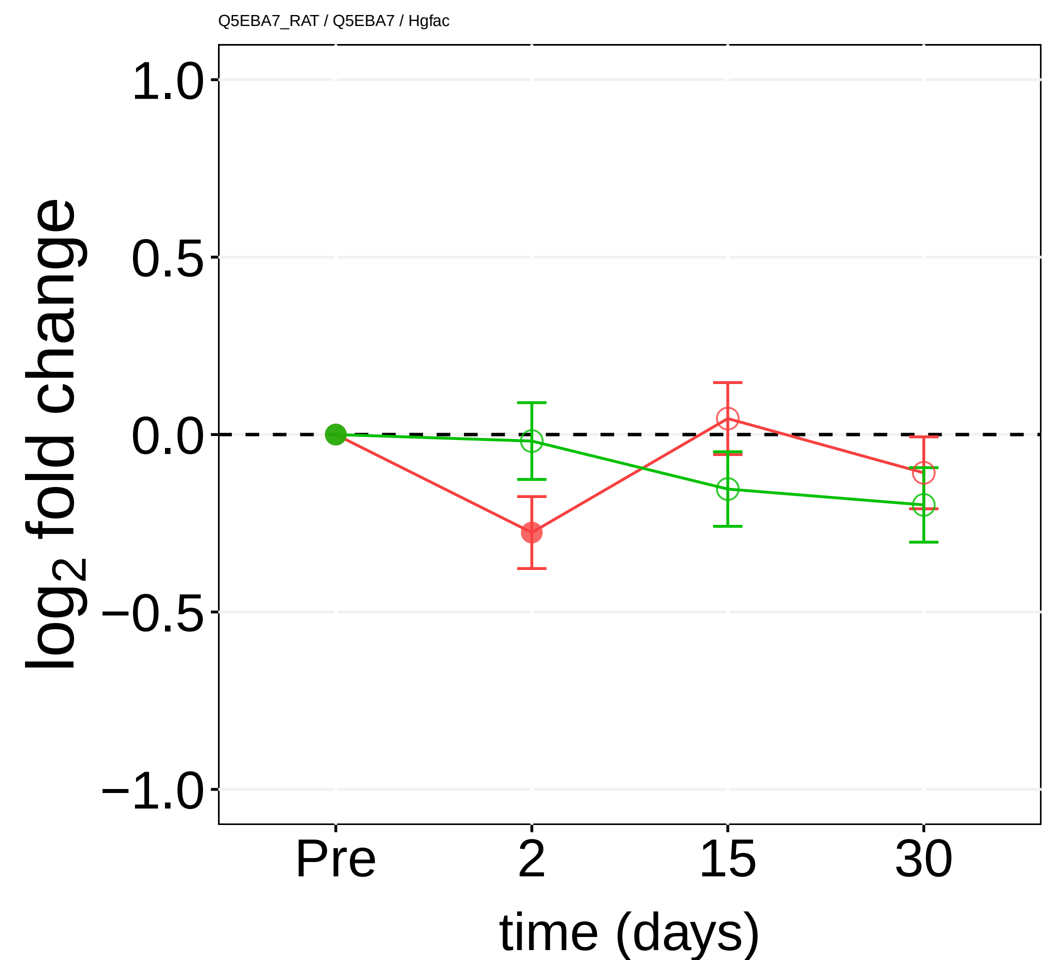

Q5EBA7_RAT / Q5EBA7 / Hgfac
1.0
0.5
0.0
−0.5
−1.0
log2 fold change
Pre
2
15
30
time (days)

Q5EBC0_RAT / Q5EBC0 / Itih4

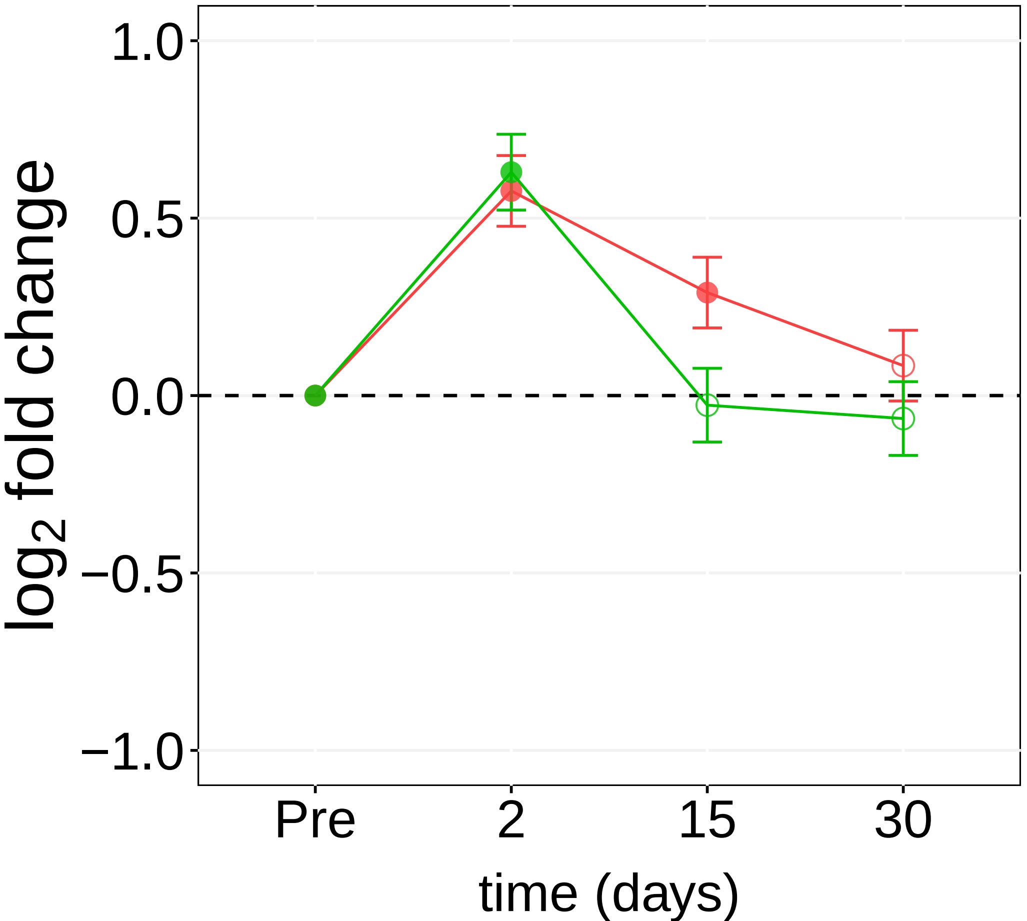

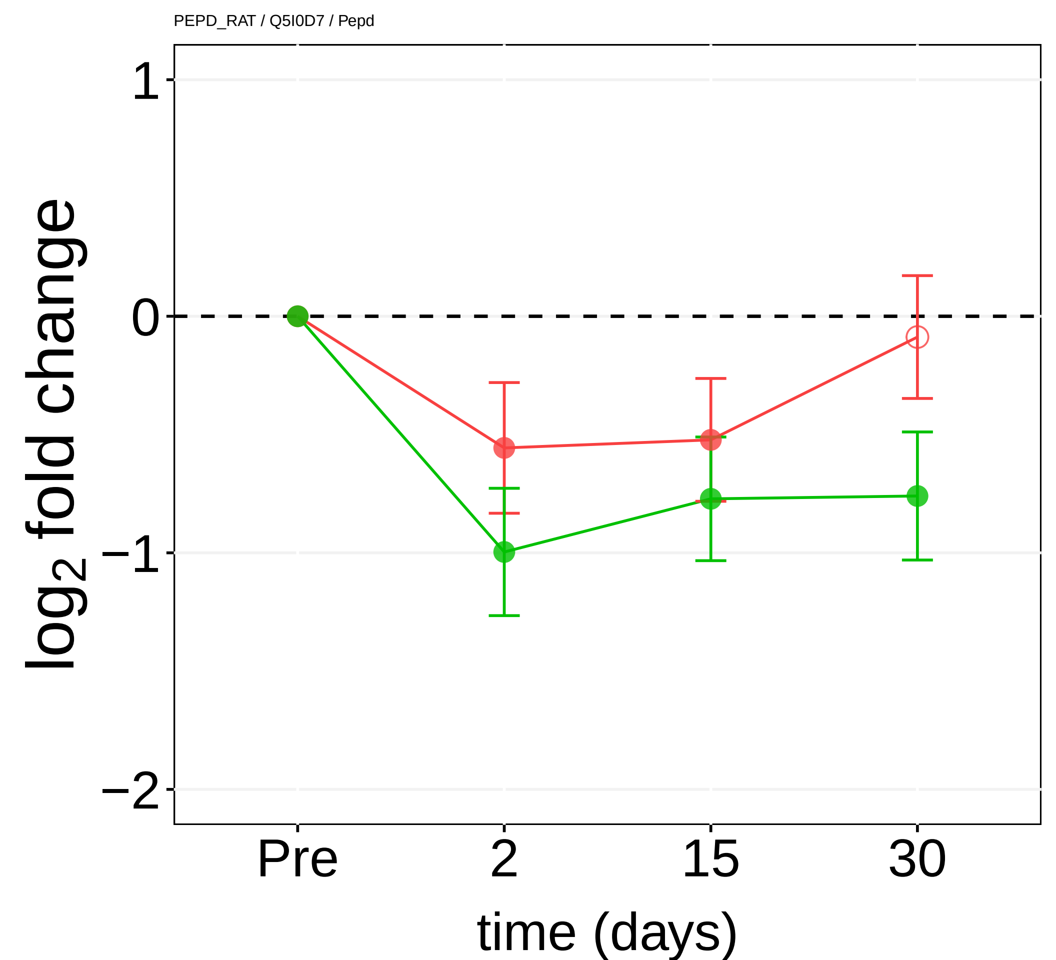
PEPD_RAT / Q5I0D7 / Pepd
log2 fold change
1
0
−1
−2
Pre
2
15
30
time (days)

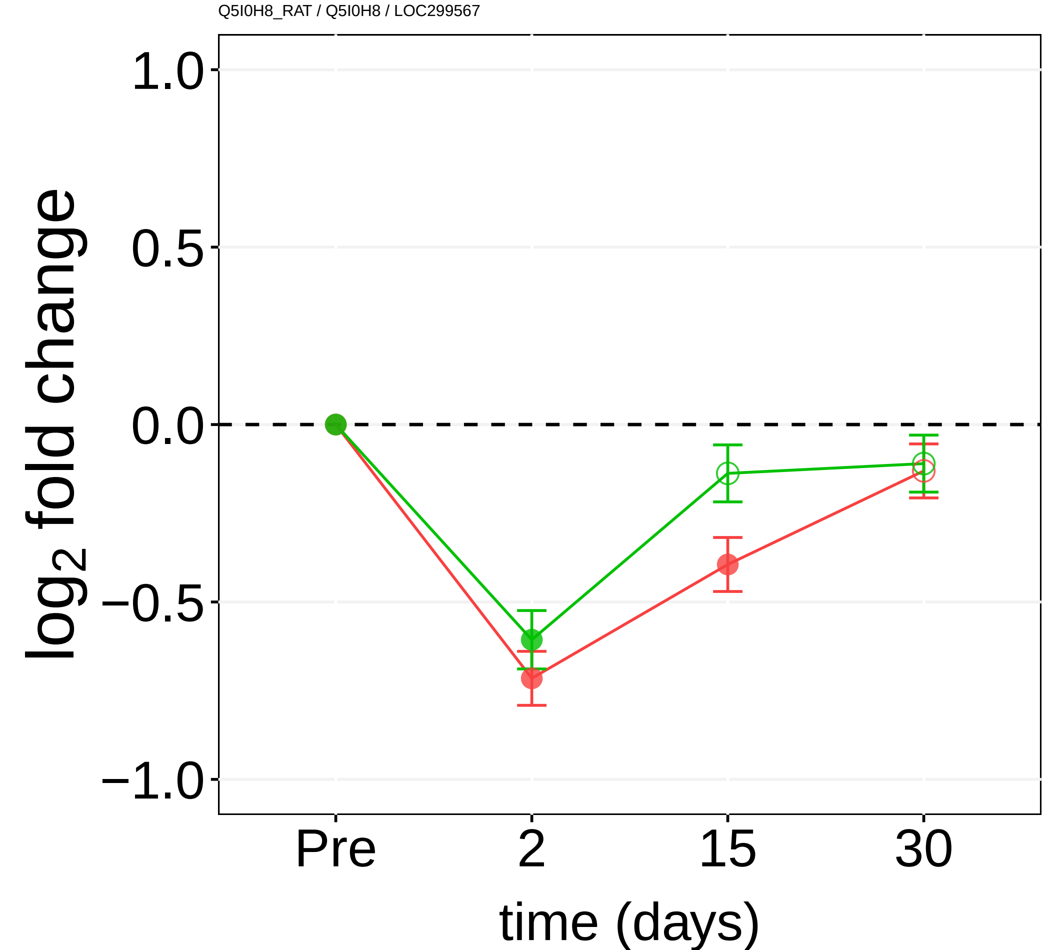
Q5I0H8_RAT / Q5I0H8 / LOC299567
1.0
0.5
0.0
−0.5
−1.0
log2 fold change
Pre
2
15
30
time (days)

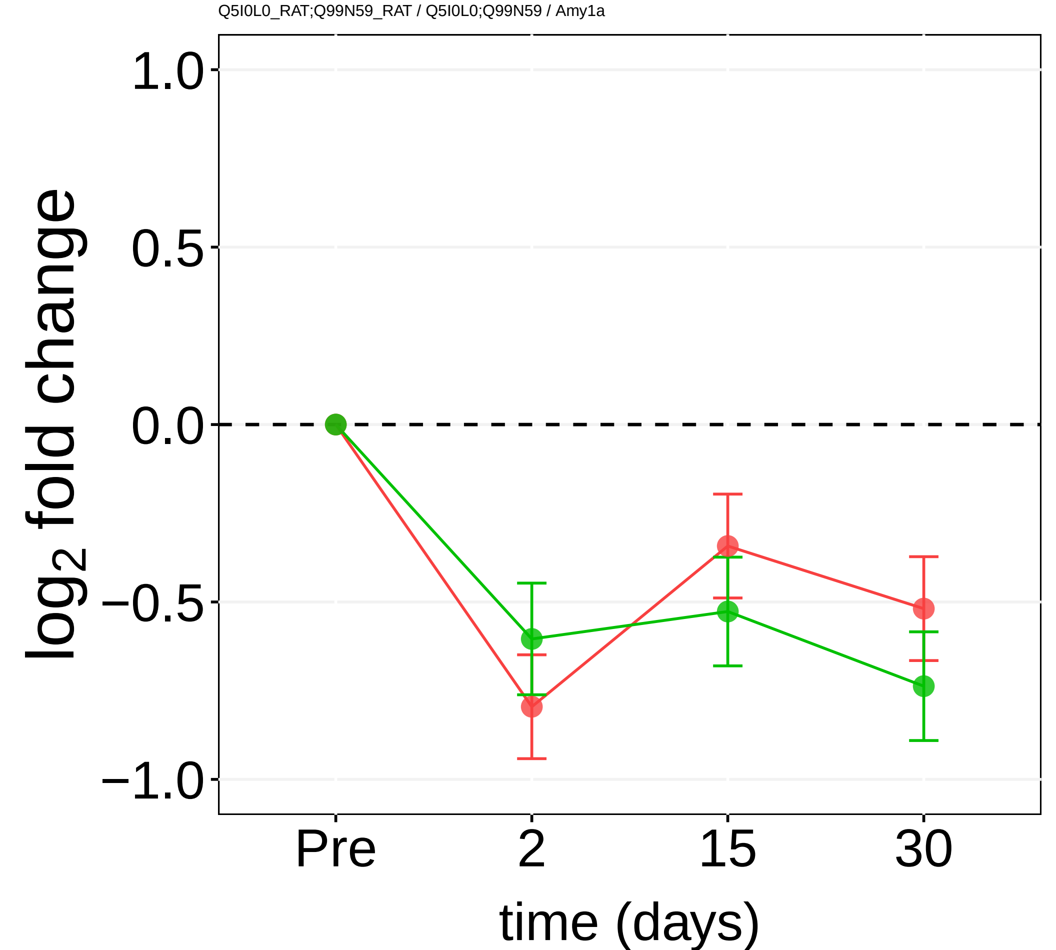

Q5I0L0_RAT;Q99N59_RAT / Q5I0L0;Q99N59 / Amy1a
1.0
0.5
0.0
−0.5
−1.0
log2 fold change
Pre
2
15
30
time (days)

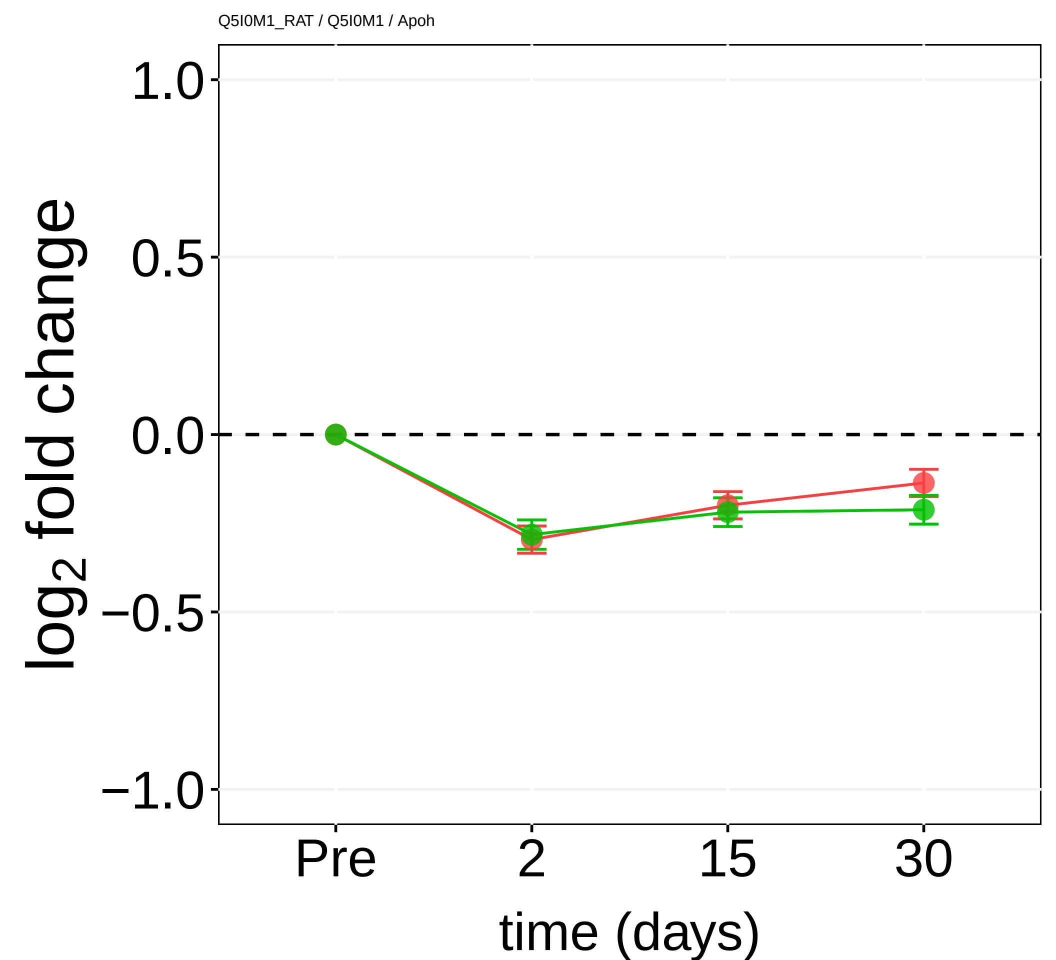

Q5I0M1_RAT / Q5I0M1 / Apoh
1.0
0.5
0.0
−0.5
−1.0
log2 fold change
Pre
2
15
30
time (days)

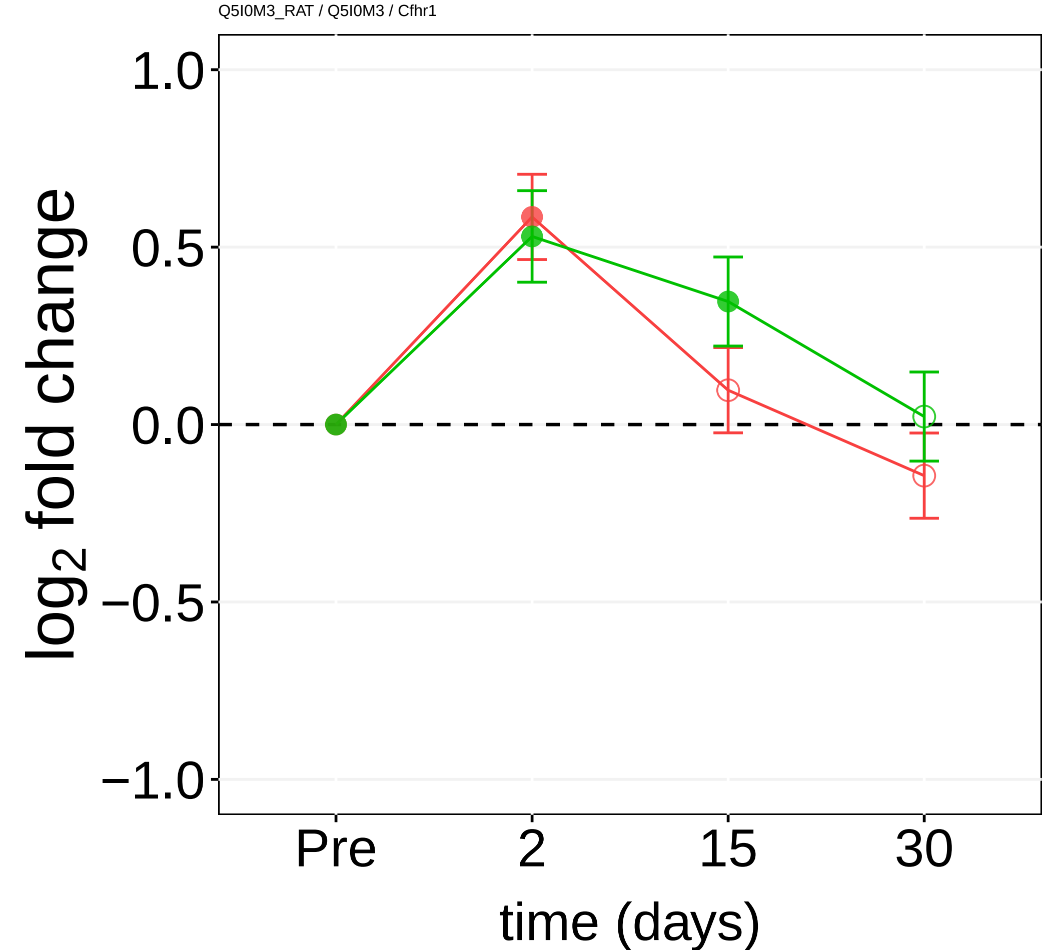
Q5I0M3_RAT / Q5I0M3 / Cfhr1
1.0
0.5
0.0
−0.5
−1.0
log2 fold change
Pre
2
15
30
time (days)

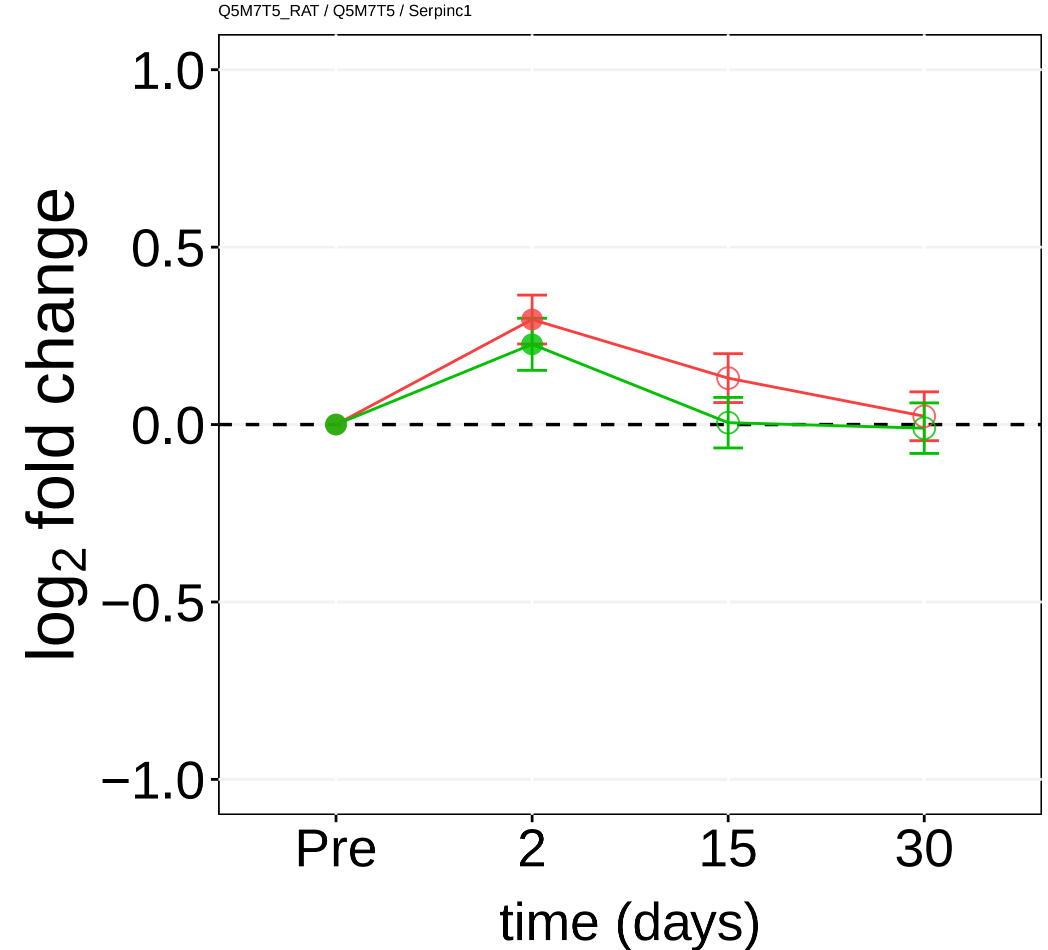
Q5M7T5_RAT / Q5M7T5 / Serpinc1
log2 fold change
1.0
0.5
0.0
−0.5
−1.0
Pre
2
15
30
time (days)

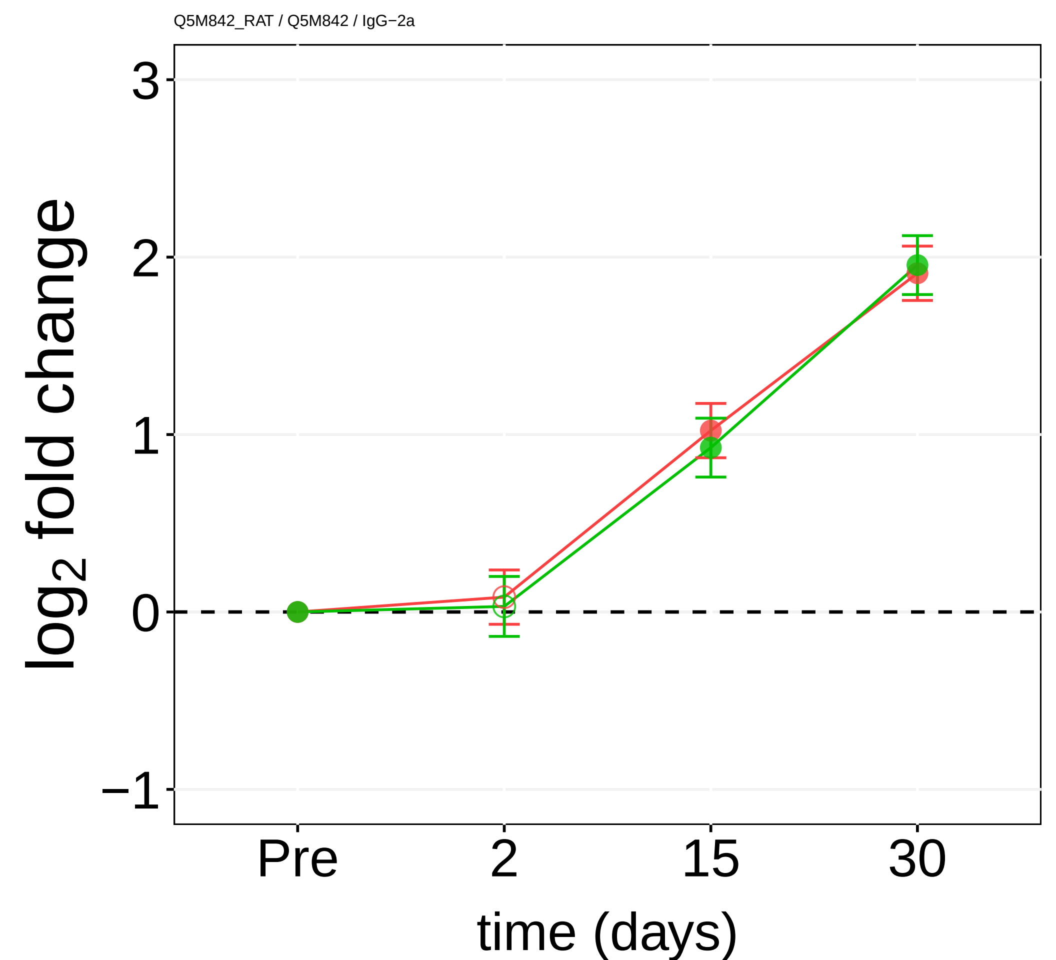

Q5M842_RAT / Q5M842 / IgG−2a
log2 fold change
3
2
1
0
−1
Pre
2
15
30
time (days)

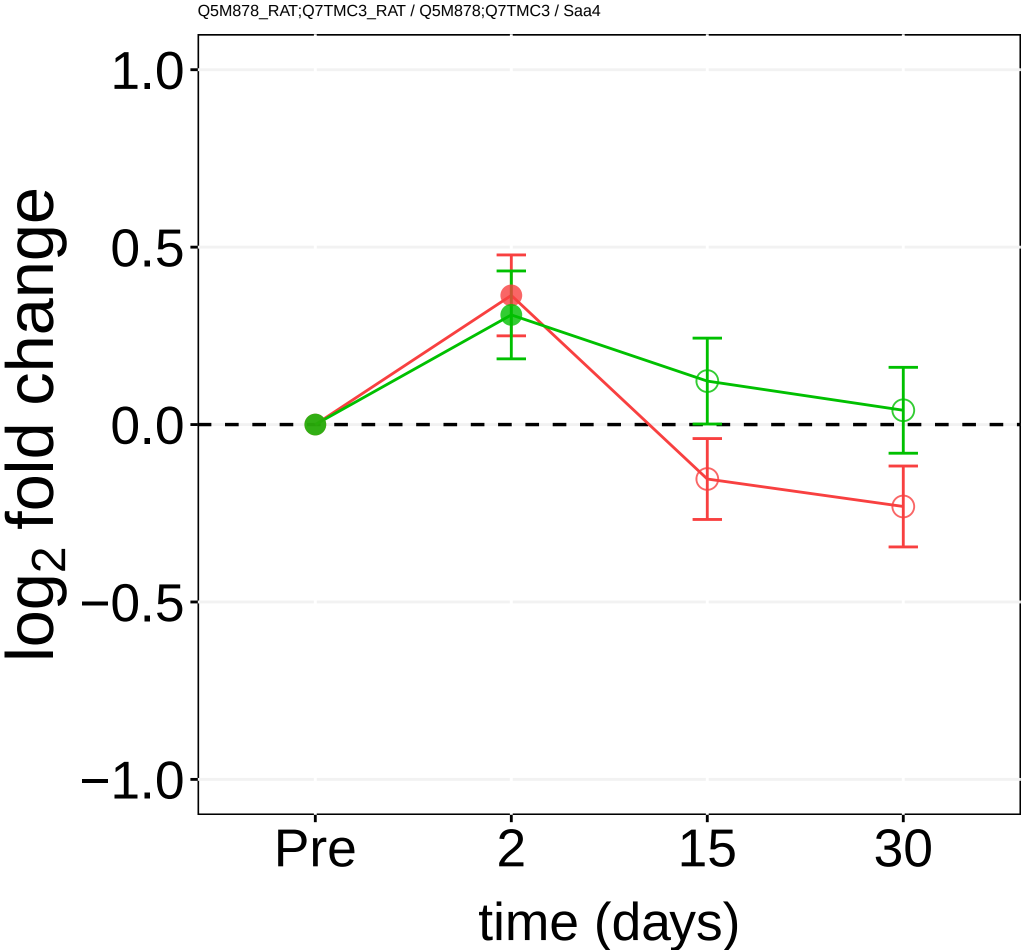
Q5M878_RAT;Q7TMC3_RAT / Q5M878;Q7TMC3 / Saa4
log2 fold change
1.0
0.5
0.0
−0.5
−1.0
Pre
2
15
30
time (days)

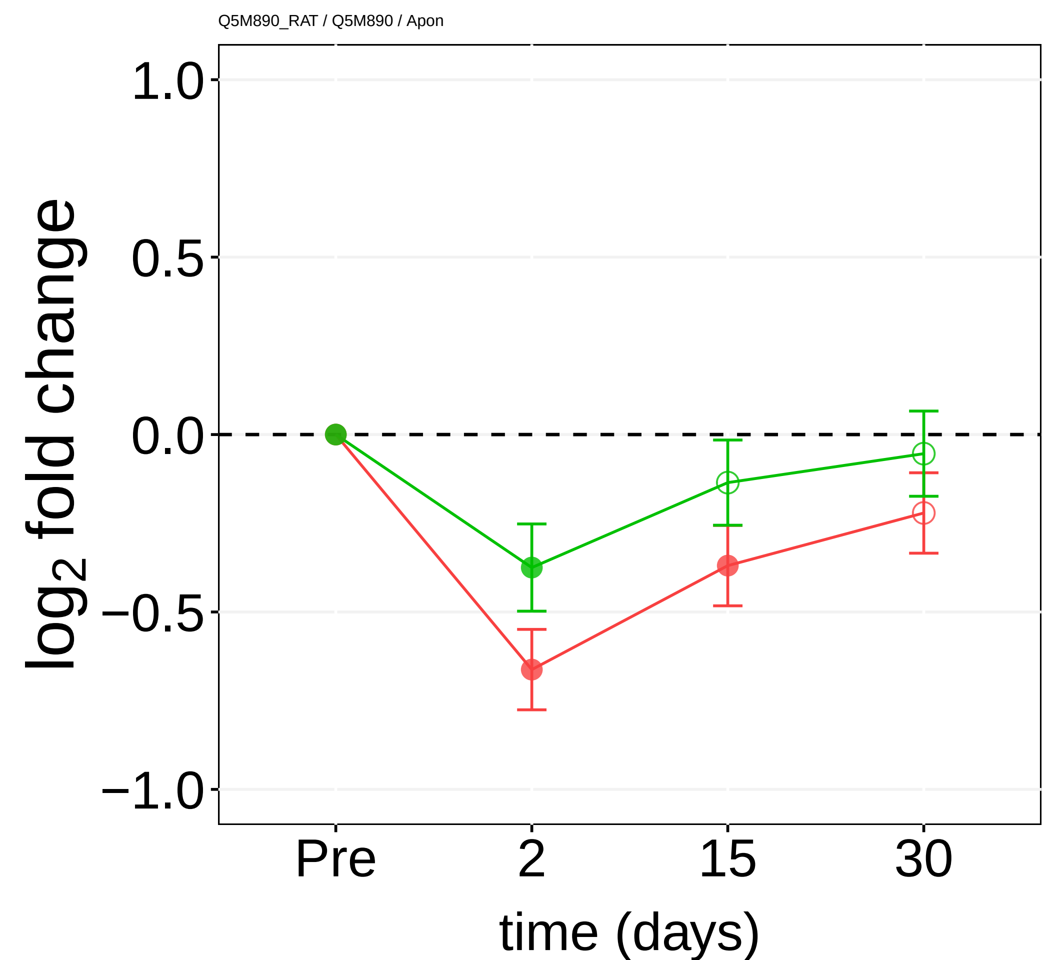
Q5M890_RAT / Q5M890 / Apon
log2 fold change
1.0
0.5
0.0
−0.5
−1.0
Pre
2
15
30
time (days)

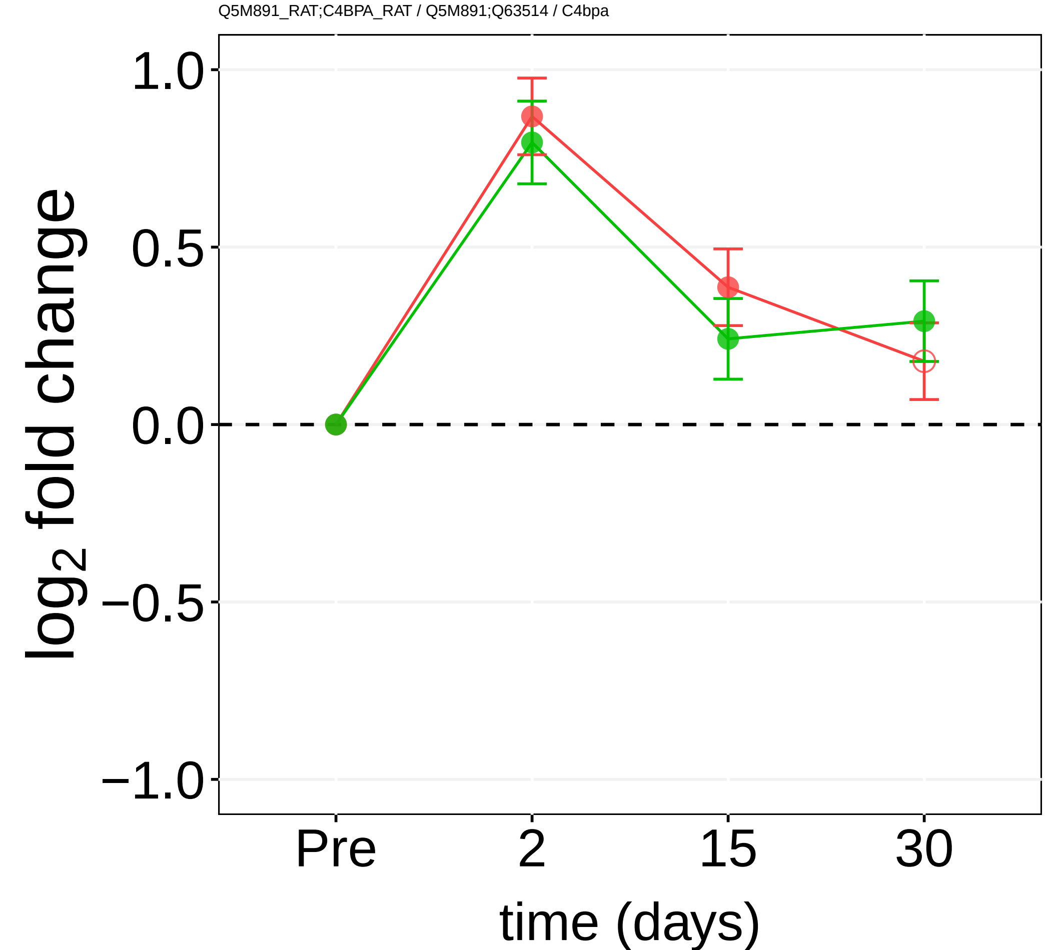
Q5M891_RAT;C4BPA_RAT / Q5M891;Q63514 / C4bpa
log2 fold change
1.0
0.5
0.0
−0.5
−1.0
Pre
2
15
30
time (days)

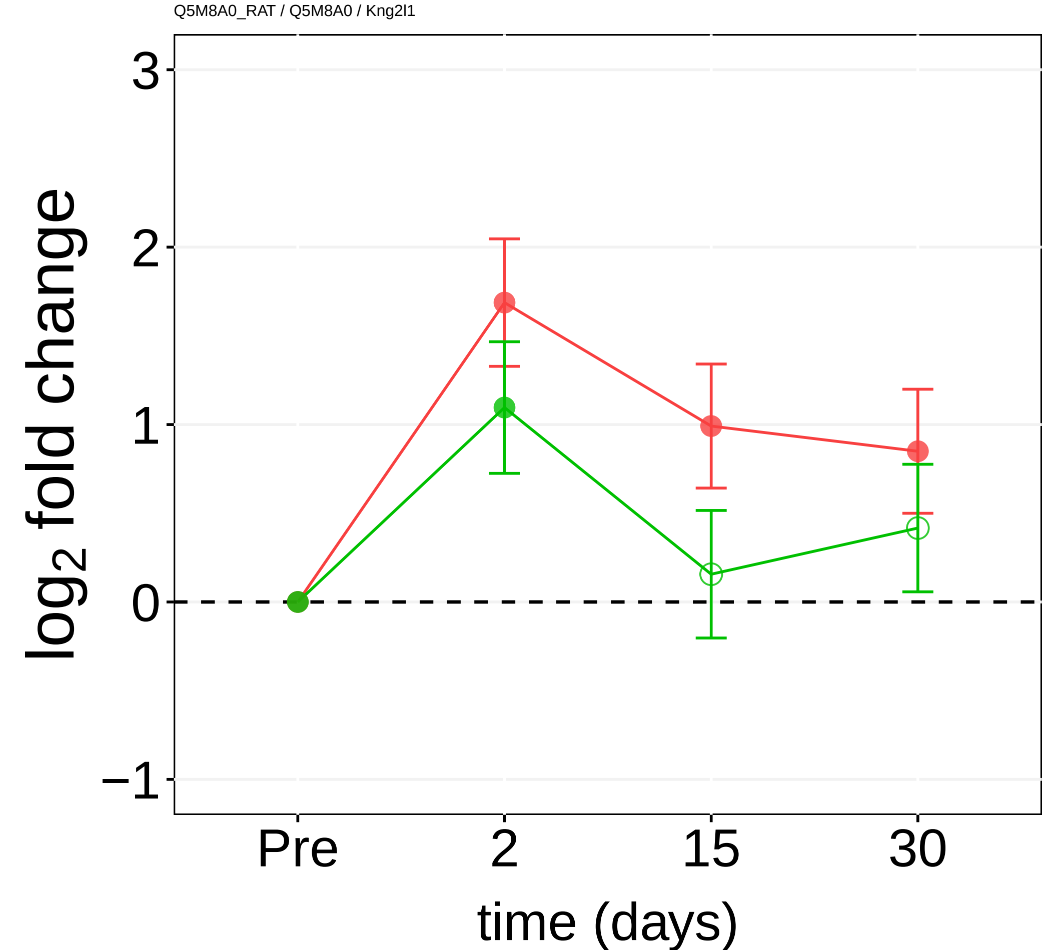
Q5M8A0_RAT / Q5M8A0 / Kng2l1
3
2
1
0
−1
log2 fold change
Pre
2
15
30
time (days)

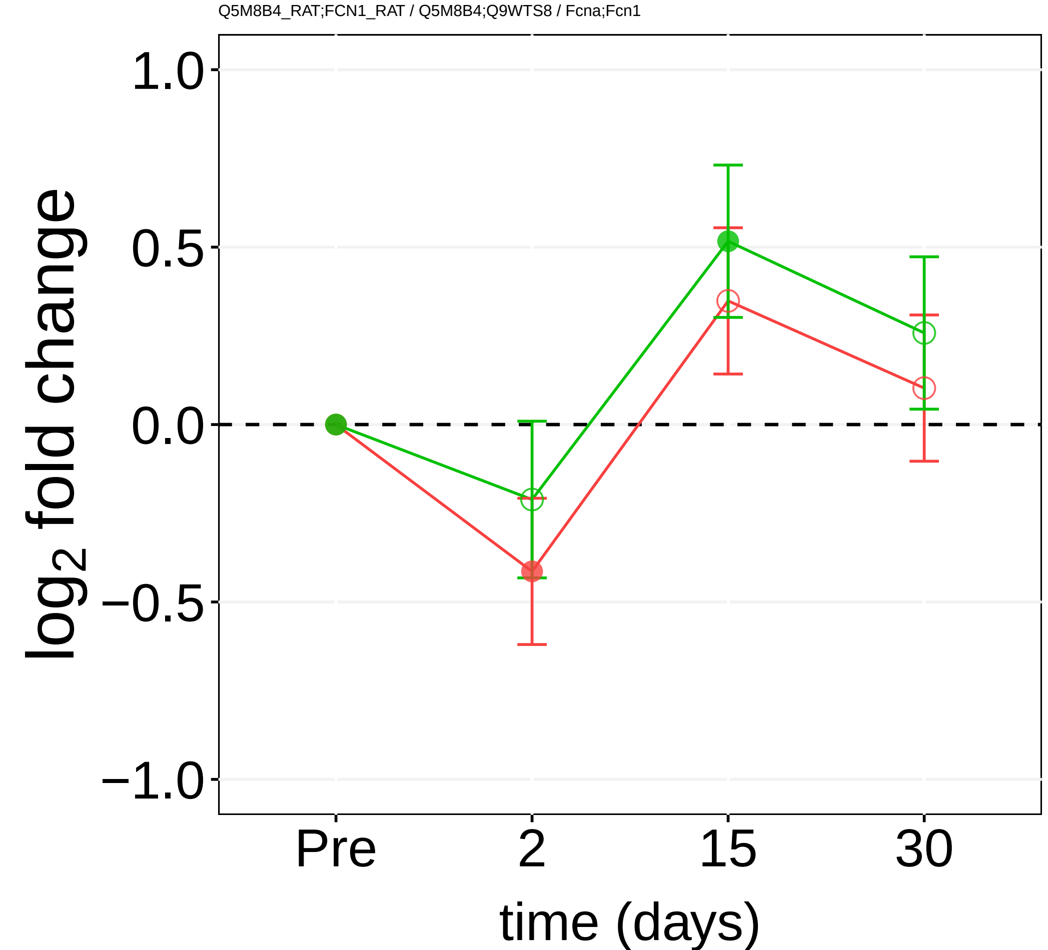

Q5M8B4_RAT;FCN1_RAT / Q5M8B4;Q9WTS8 / Fcna;Fcn1
1.0
0.5
0.0
−0.5
−1.0
log2 fold change
Pre
2
15
30
time (days)

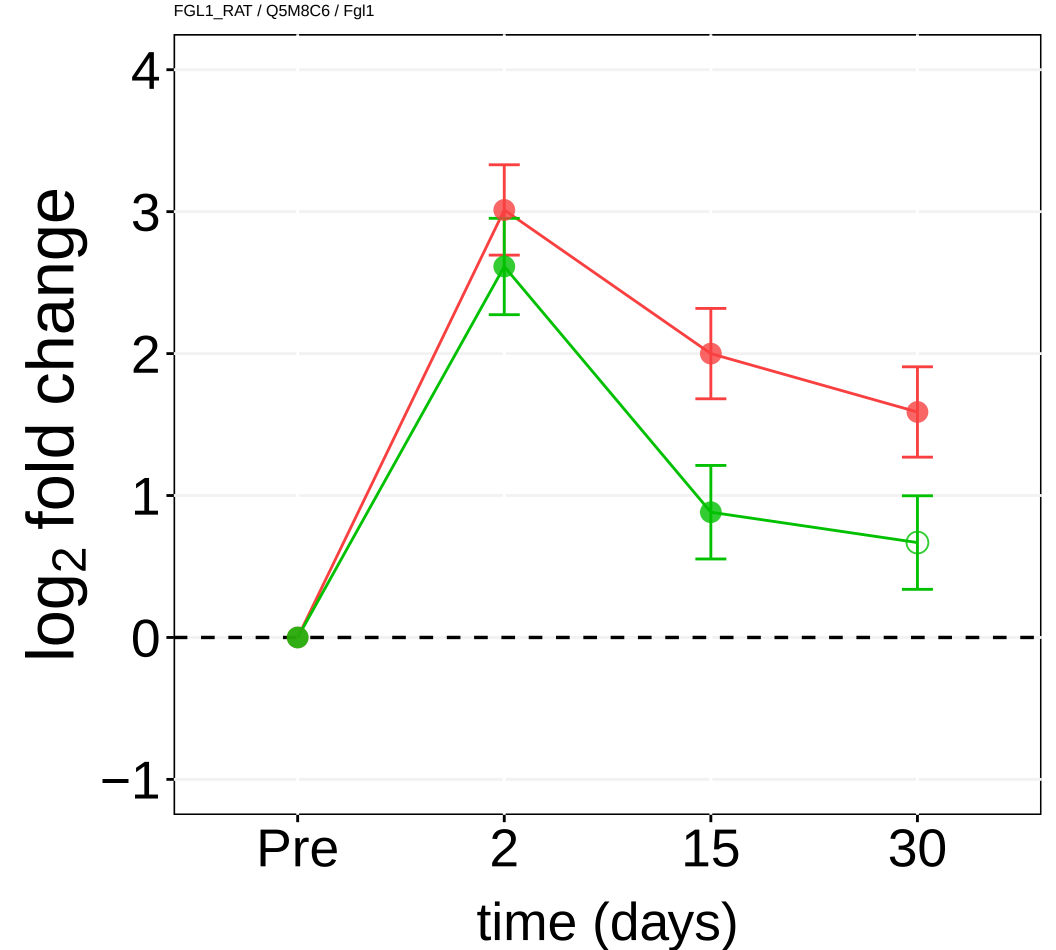
FGL1_RAT / Q5M8C6 / Fgl1
4
3
2
1
0
−1
log2 fold change
Pre
2
15
30
time (days)

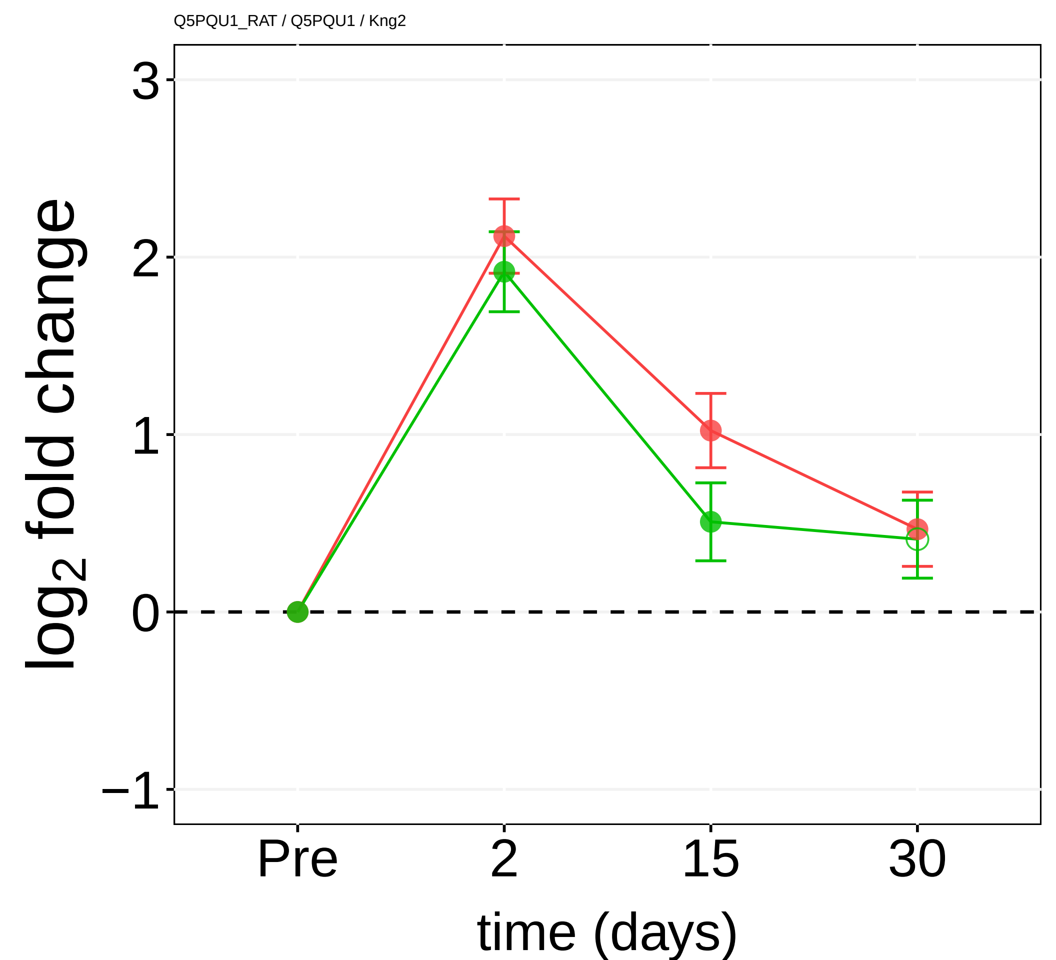

Q5PQU1_RAT / Q5PQU1 / Kng2
3
2
1
0
−1
log2 fold change
Pre
2
15
30
time (days)

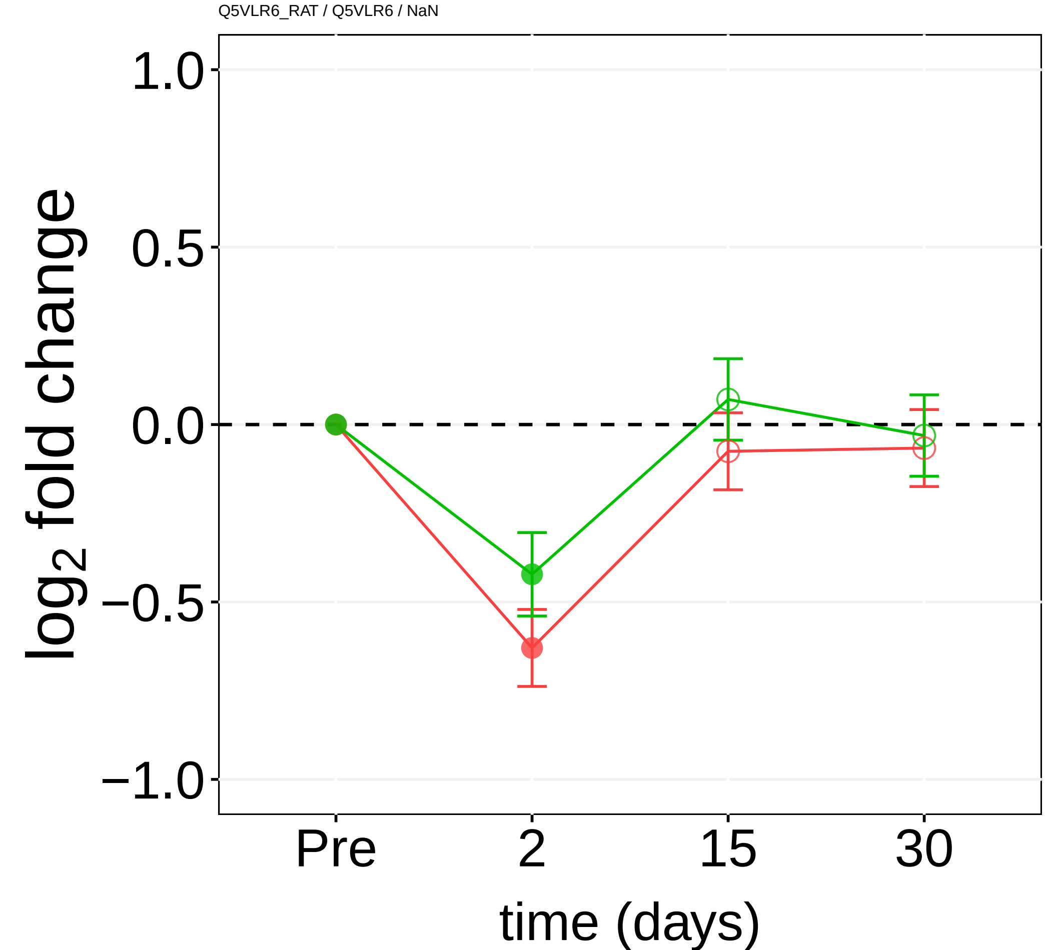
Q5VLR6_RAT / Q5VLR6 / NaN
1.0
0.5
0.0
−0.5
−1.0
log2 fold change
Pre
2
15
30
time (days)

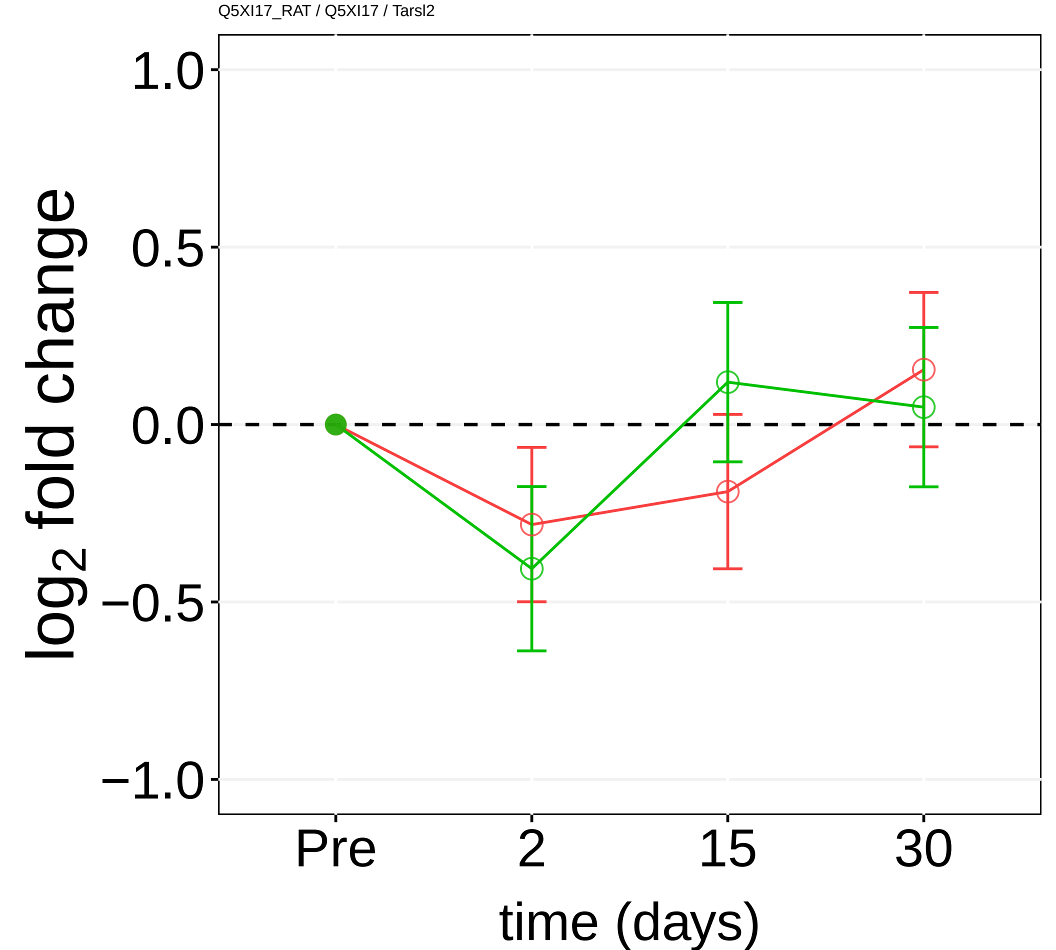

Q5XI17_RAT / Q5XI17 / Tarsl2
log2 fold change
1.0
0.5
0.0
−0.5
−1.0
Pre
2
15
30
time (days)

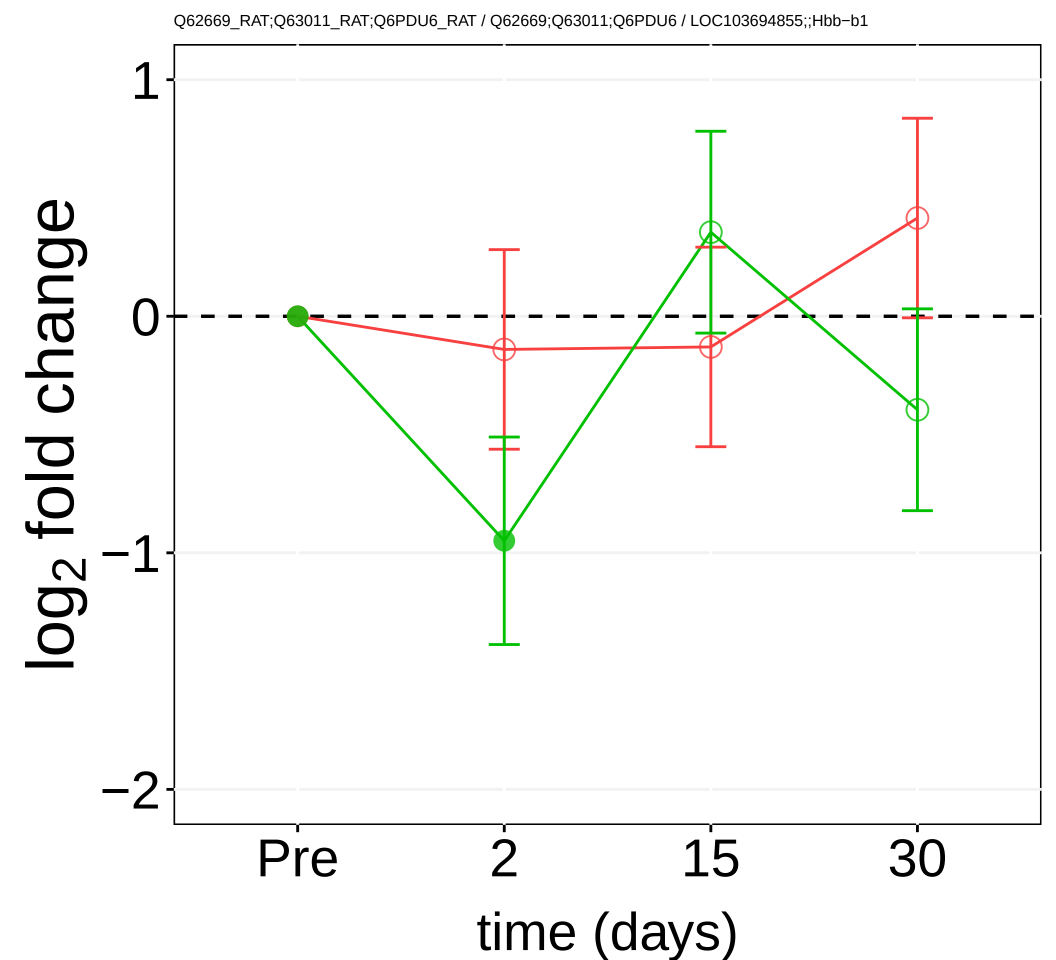
Q62669_RAT;Q63011_RAT;Q6PDU6_RAT / Q62669;Q63011;Q6PDU6 / LOC103694855;;Hbb−b1
log2 fold change
1
0
−1
−2
Pre
2
15
30
time (days)

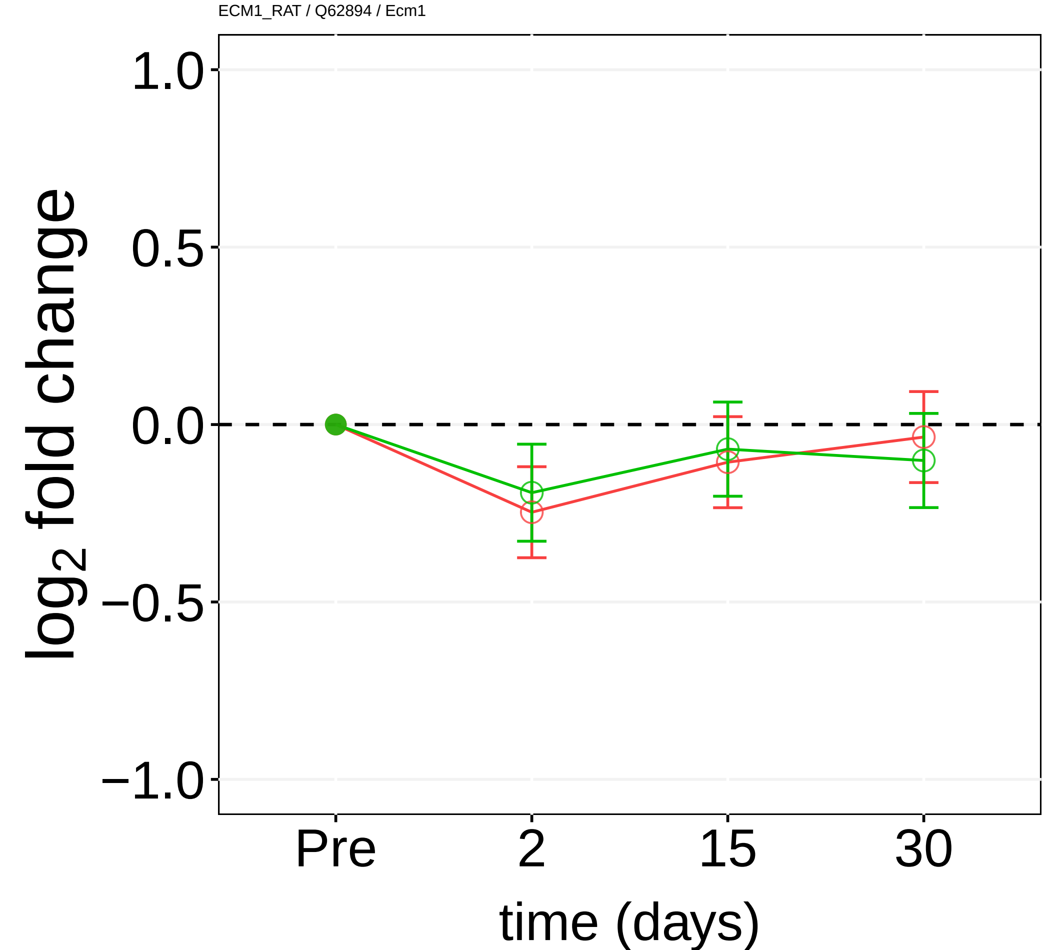

ECM1_RAT / Q62894 / Ecm1
1.0
0.5
0.0
−0.5
−1.0
log2 fold change
Pre
2
15
30
time (days)

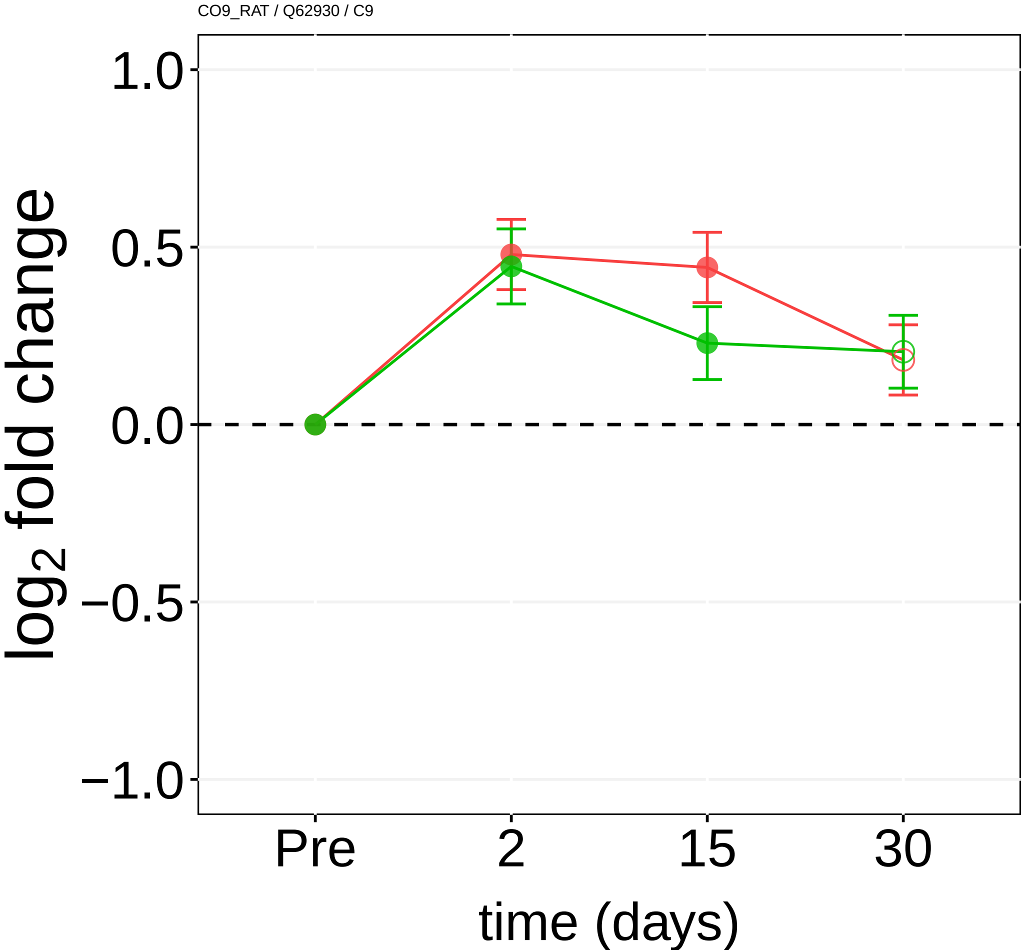

CO9_RAT / Q62930 / C9
1.0
0.5
0.0
−0.5
−1.0
log2 fold change
Pre
2
15
30
time (days)

ZPI_RAT / Q62975 / Serpina10

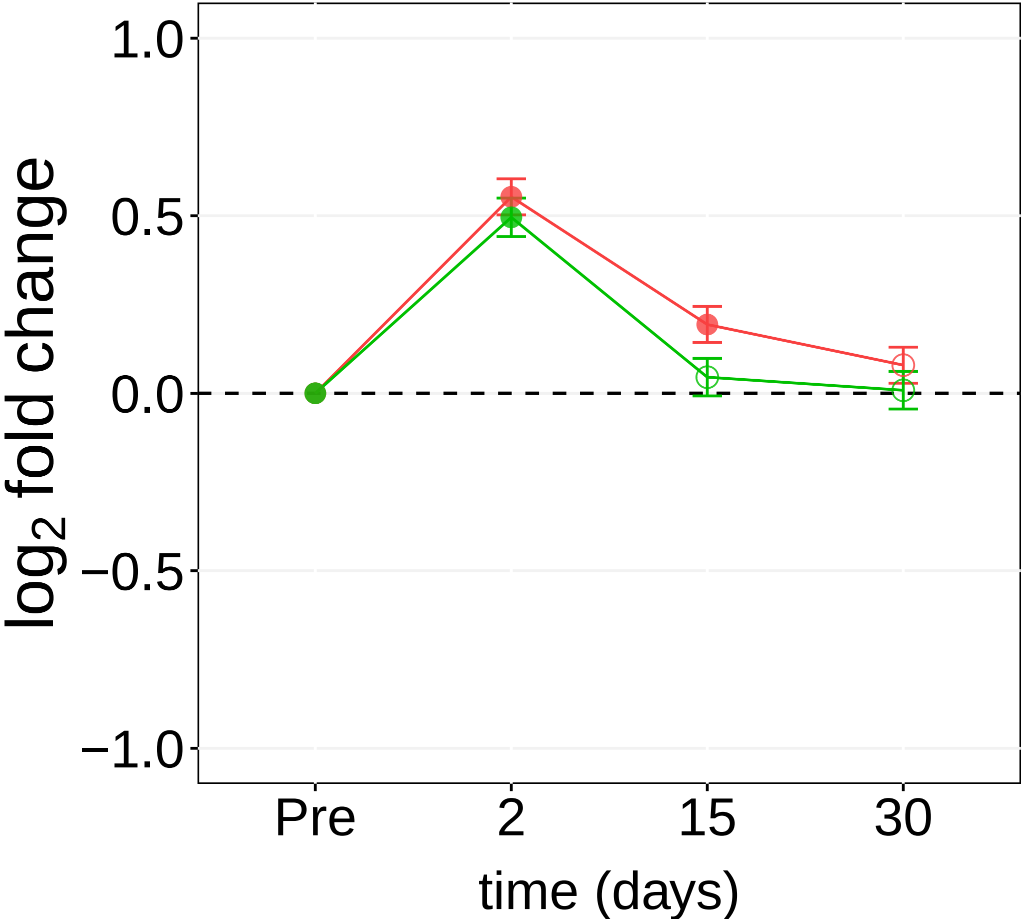

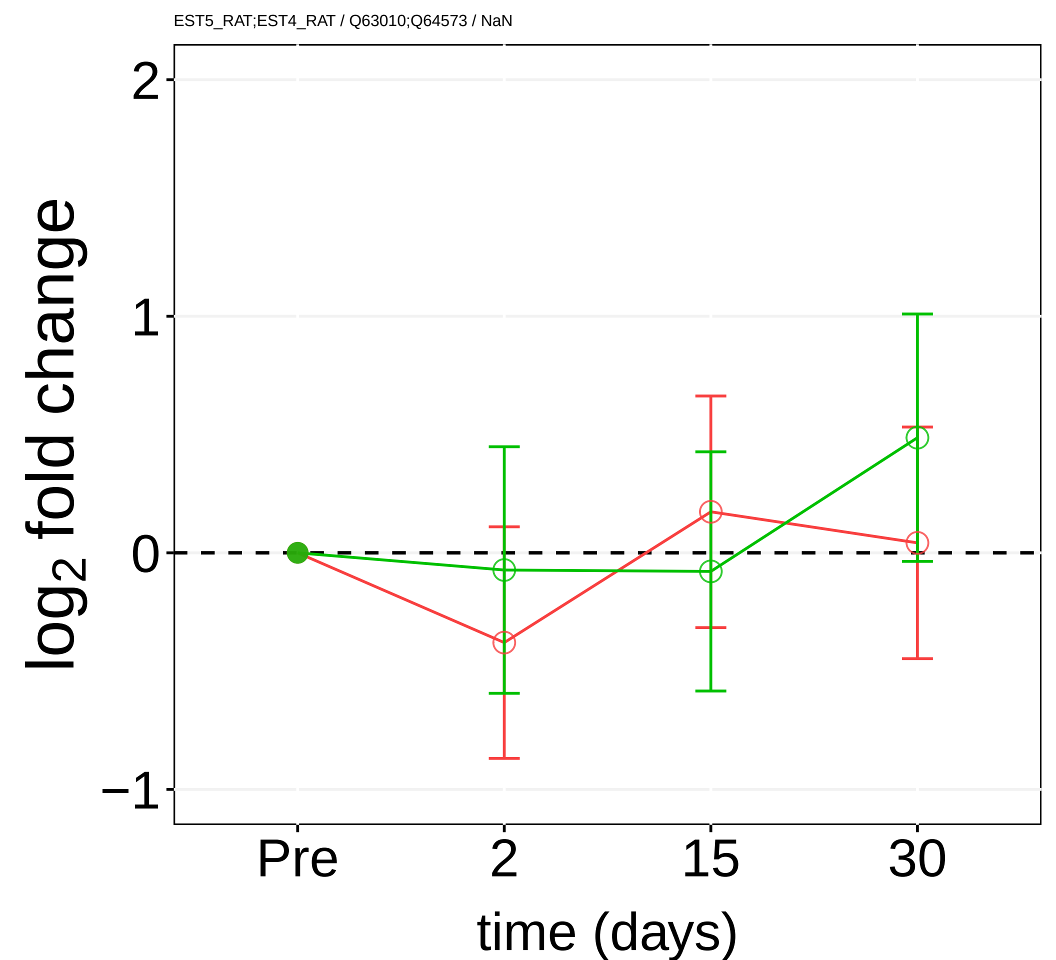

EST5_RAT;EST4_RAT / Q63010;Q64573 / NaN
log2 fold change
2
1
0
−1
Pre
2
15
30
time (days)

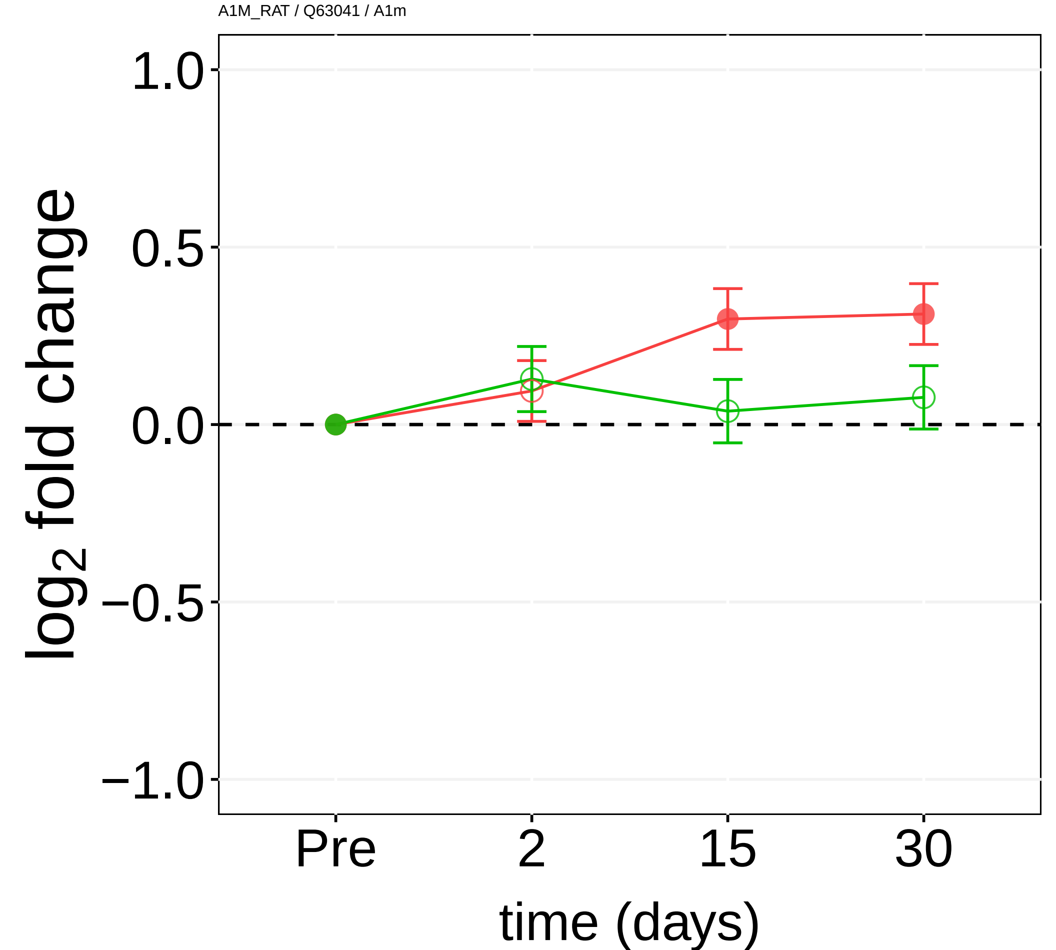
A1M_RAT / Q63041 / A1m
1.0
0.5
0.0
−0.5
−1.0
log2 fold change
Pre
2
15
30
time (days)

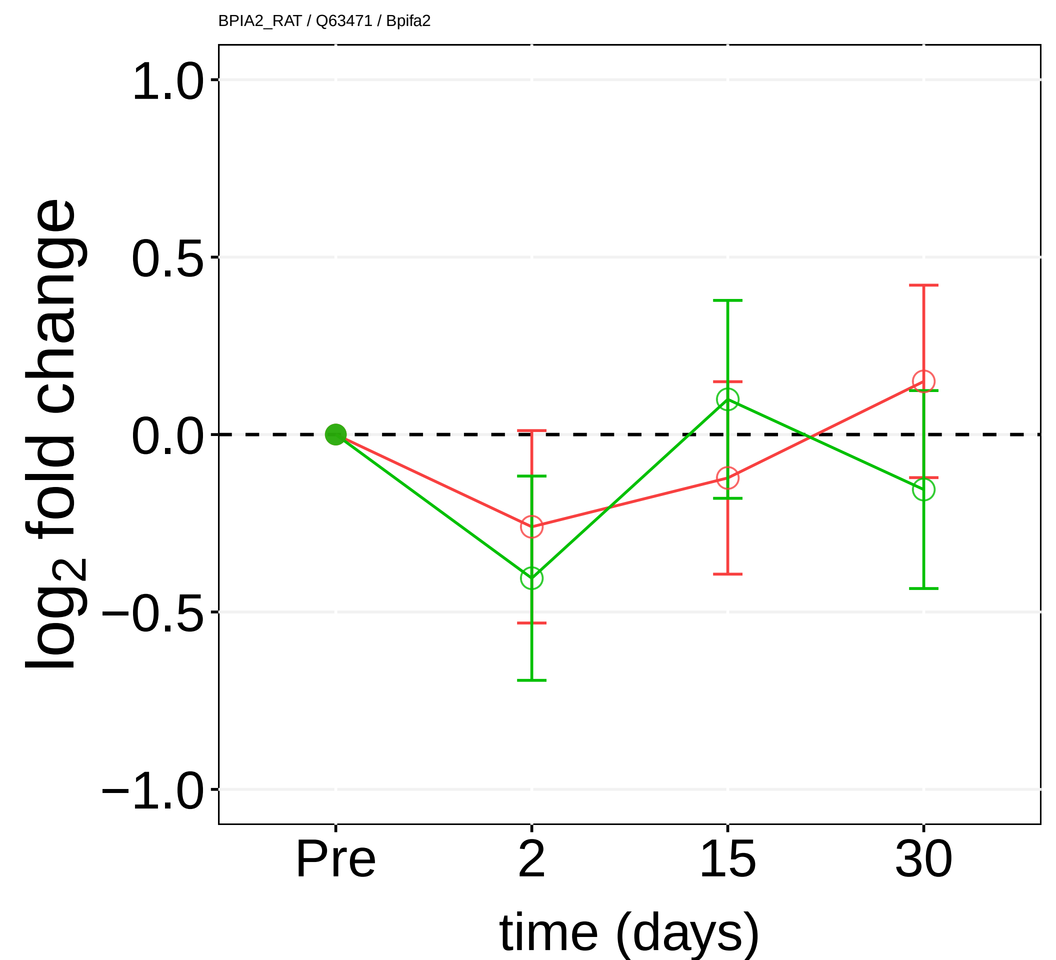

BPIA2_RAT / Q63471 / Bpifa2
log2 fold change
1.0
0.5
0.0
−0.5
−1.0
Pre
2
15
30
time (days)

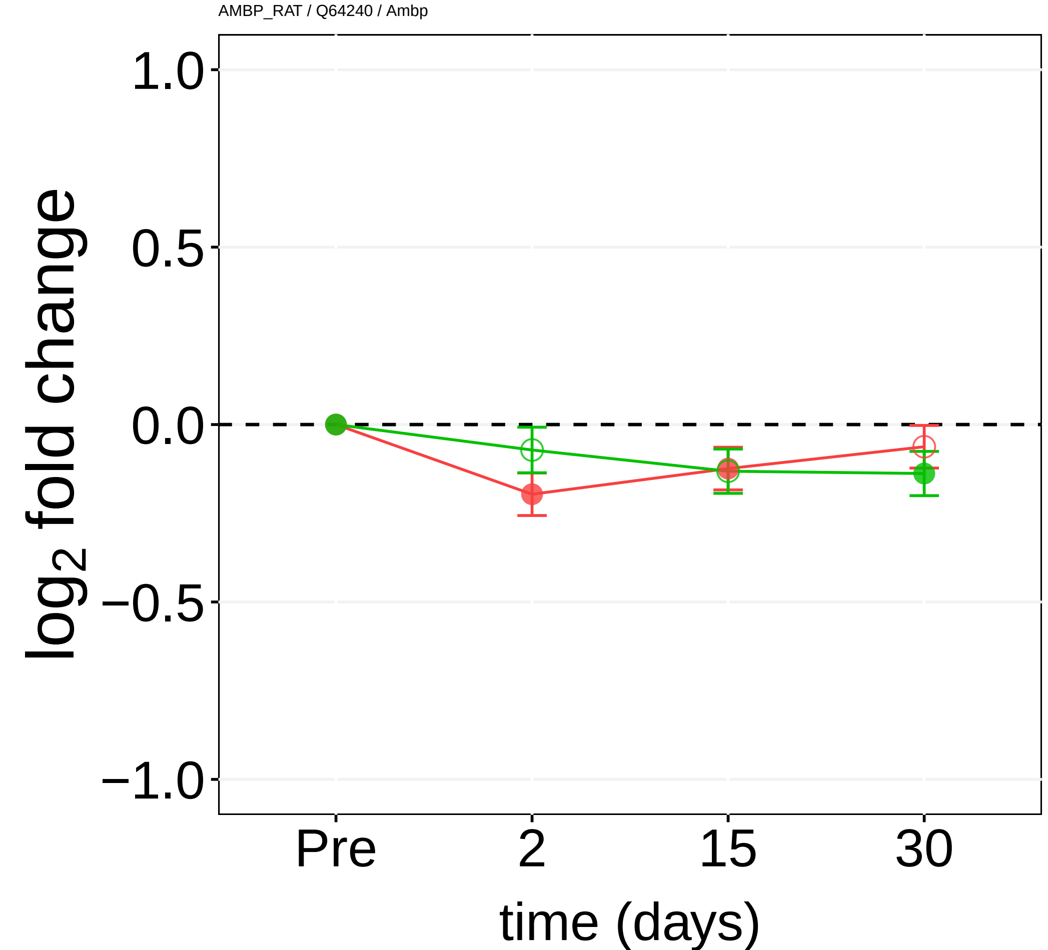
AMBP_RAT / Q64240 / Ambp
1.0
0.5
0.0
−0.5
−1.0
log2 fold change
Pre
2
15
30
time (days)

GELS_RAT / Q68FP1 / Gsn

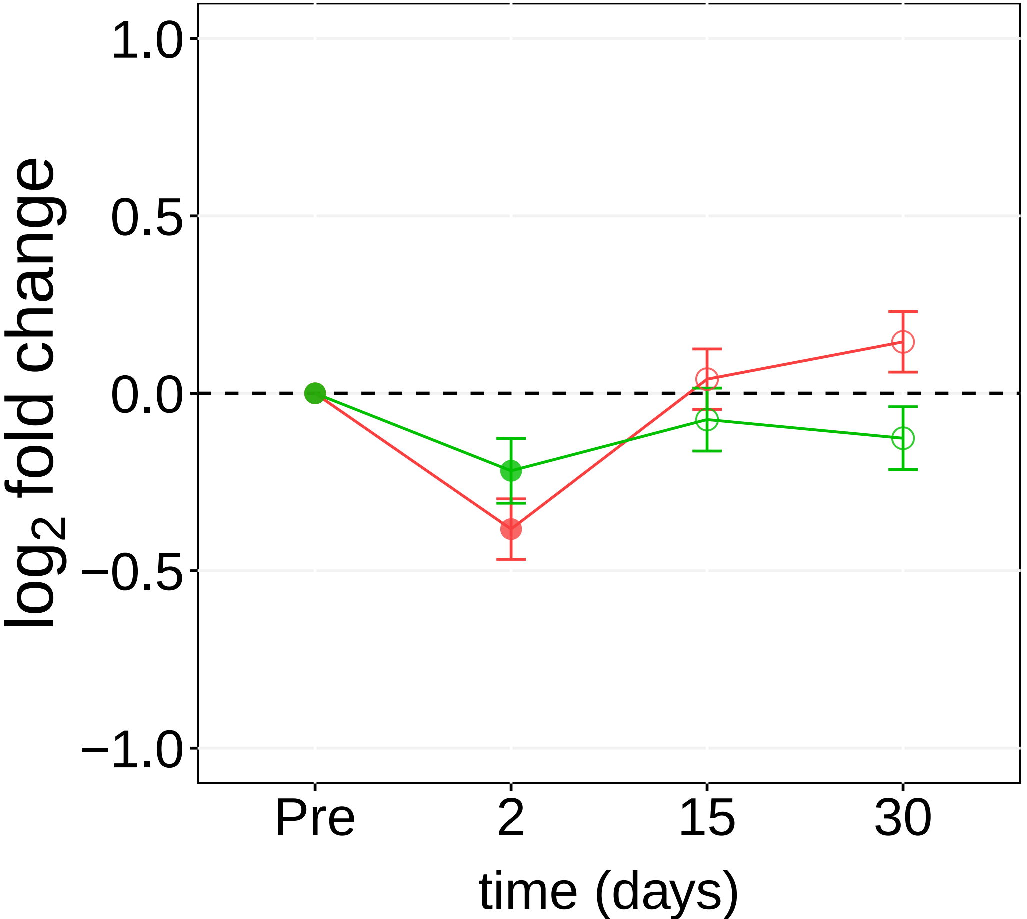

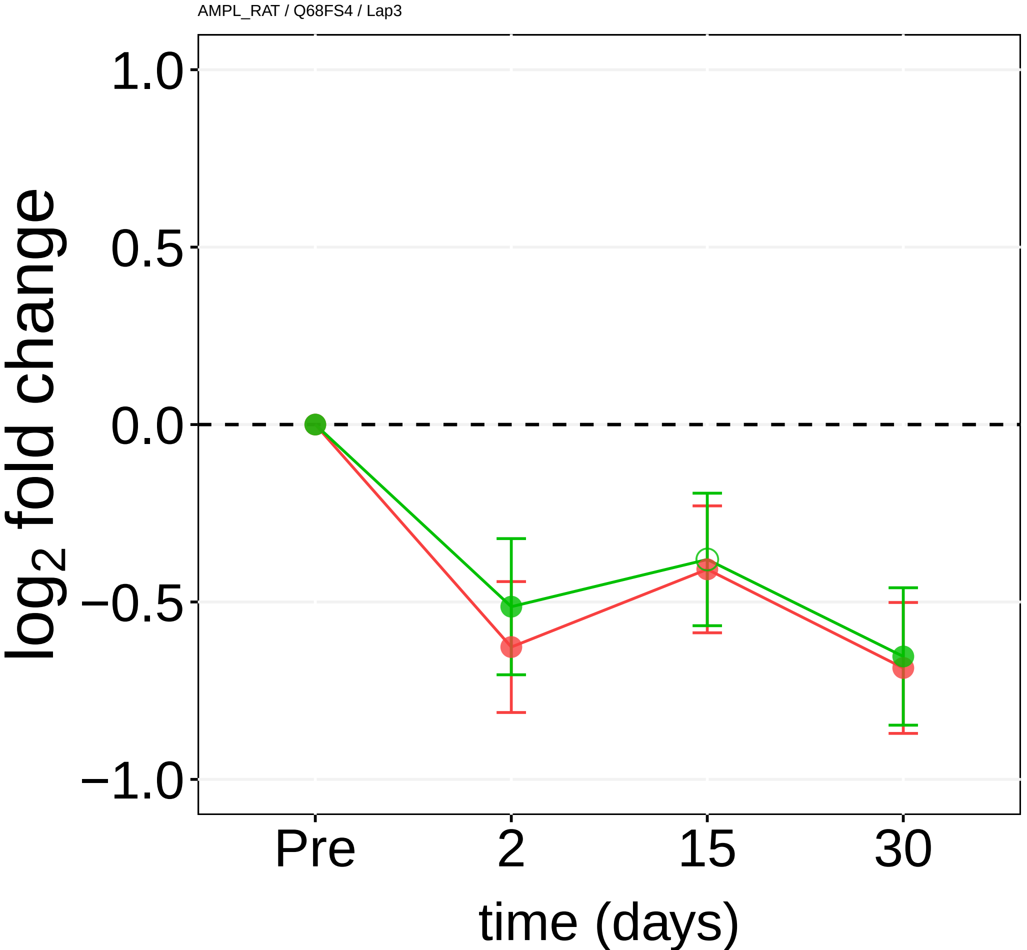

AMPL_RAT / Q68FS4 / Lap3
1.0
0.5
0.0
−0.5
−1.0
log2 fold change
Pre
2
15
30
time (days)

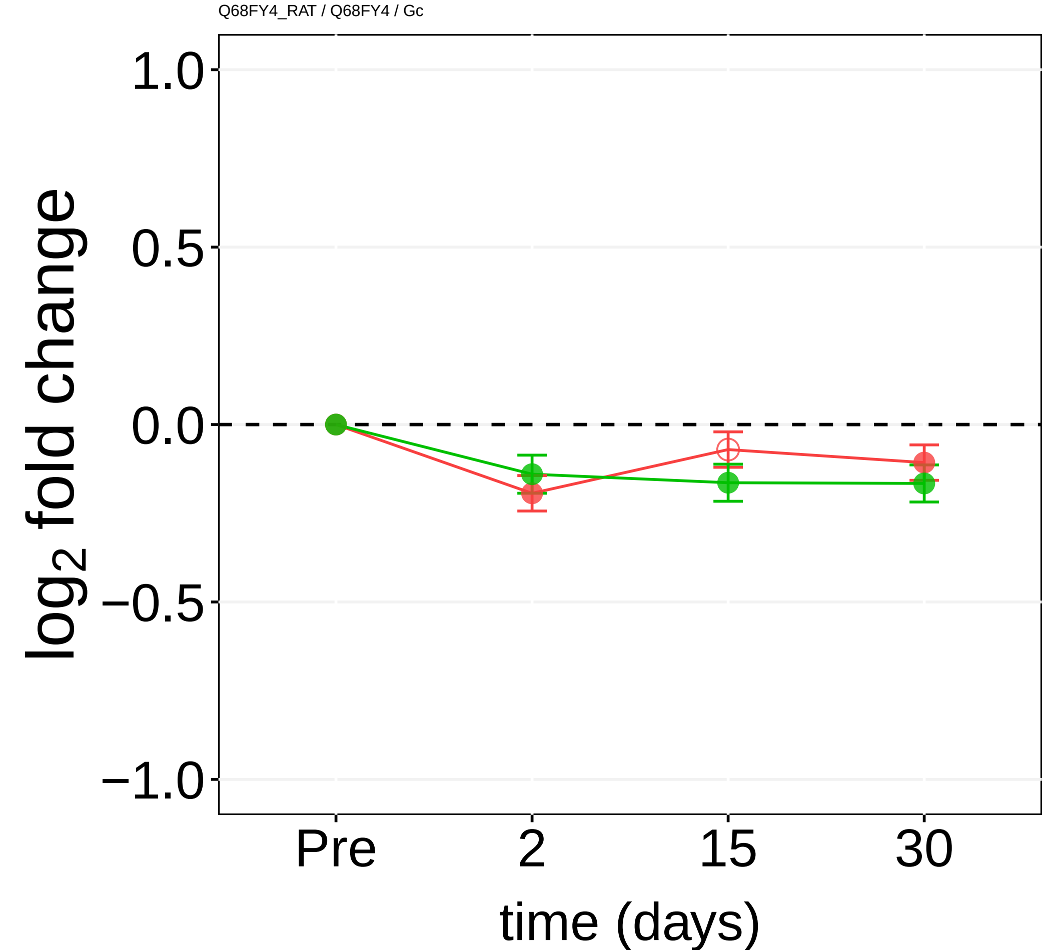

Q68FY4_RAT / Q68FY4 / Gc
1.0
0.5
0.0
−0.5
−1.0
log2 fold change
Pre
2
15
30
time (days)

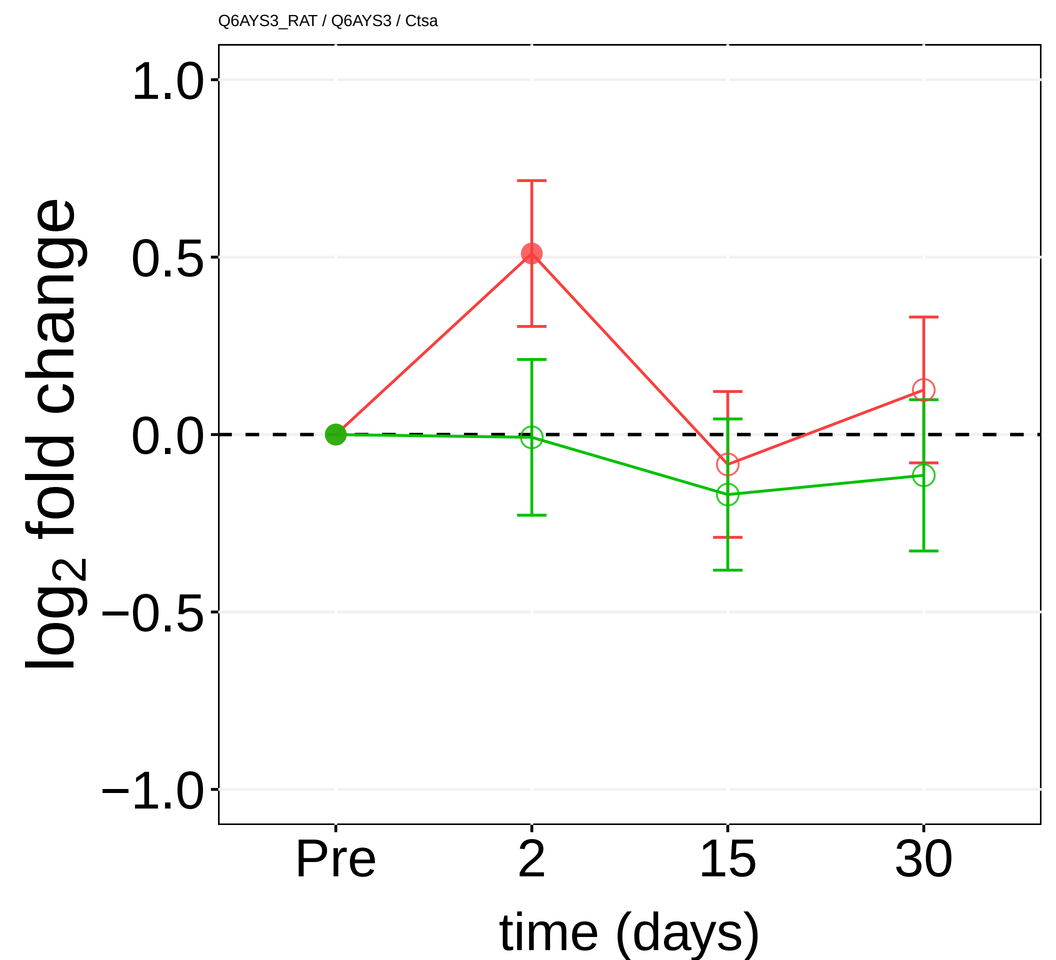
Q6AYS3_RAT / Q6AYS3 / Ctsa
1.0
0.5
0.0
−0.5
−1.0
log2 fold change
Pre
2
15
30
time (days)

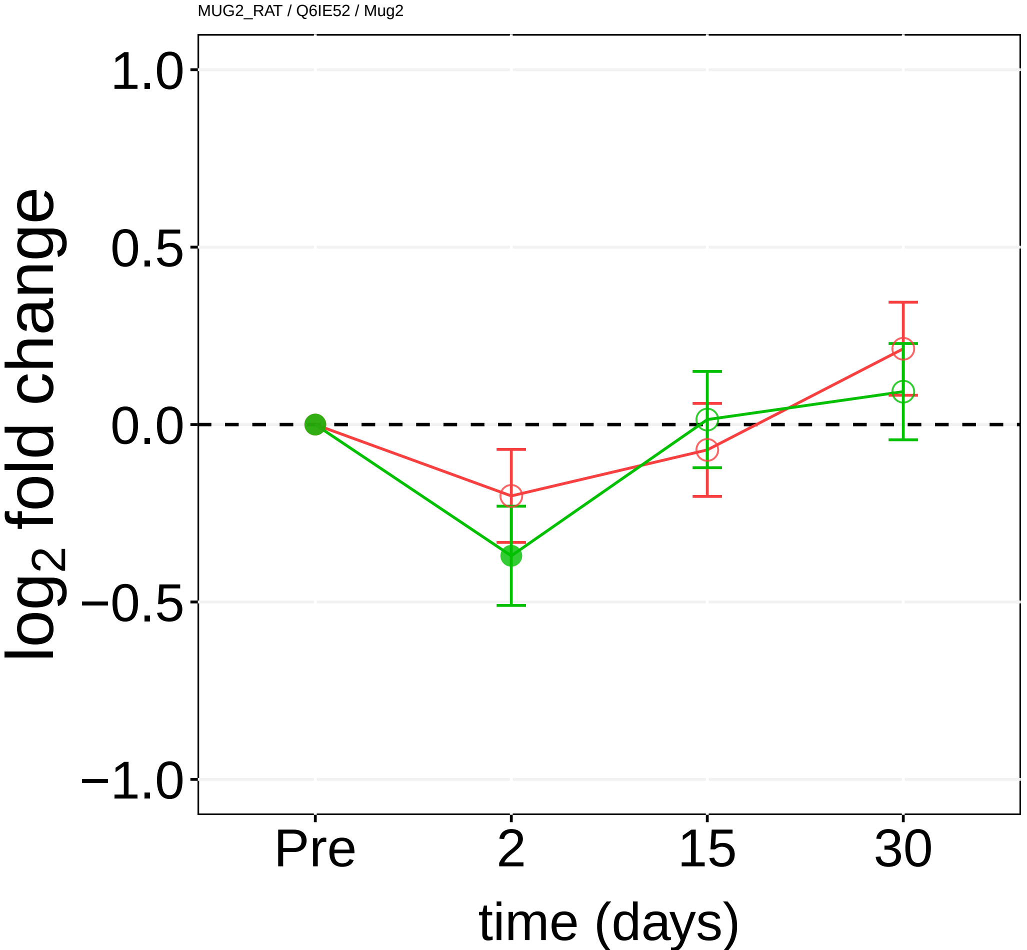

MUG2_RAT / Q6IE52 / Mug2
1.0
0.5
0.0
−0.5
−1.0
log2 fold change
Pre
2
15
30
time (days)

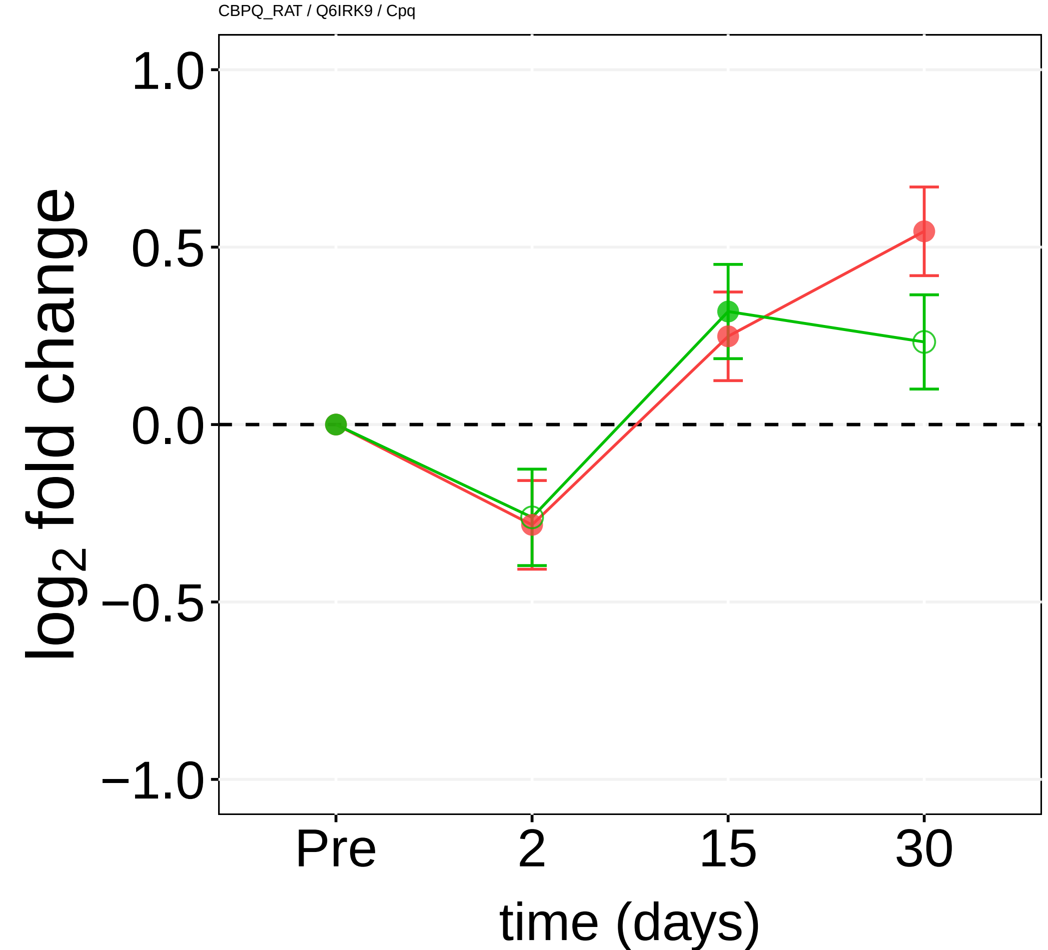

CBPQ_RAT / Q6IRK9 / Cpq
1.0
0.5
0.0
−0.5
−1.0
$\log_2$ fold change
Pre
2
15
30
time (days)

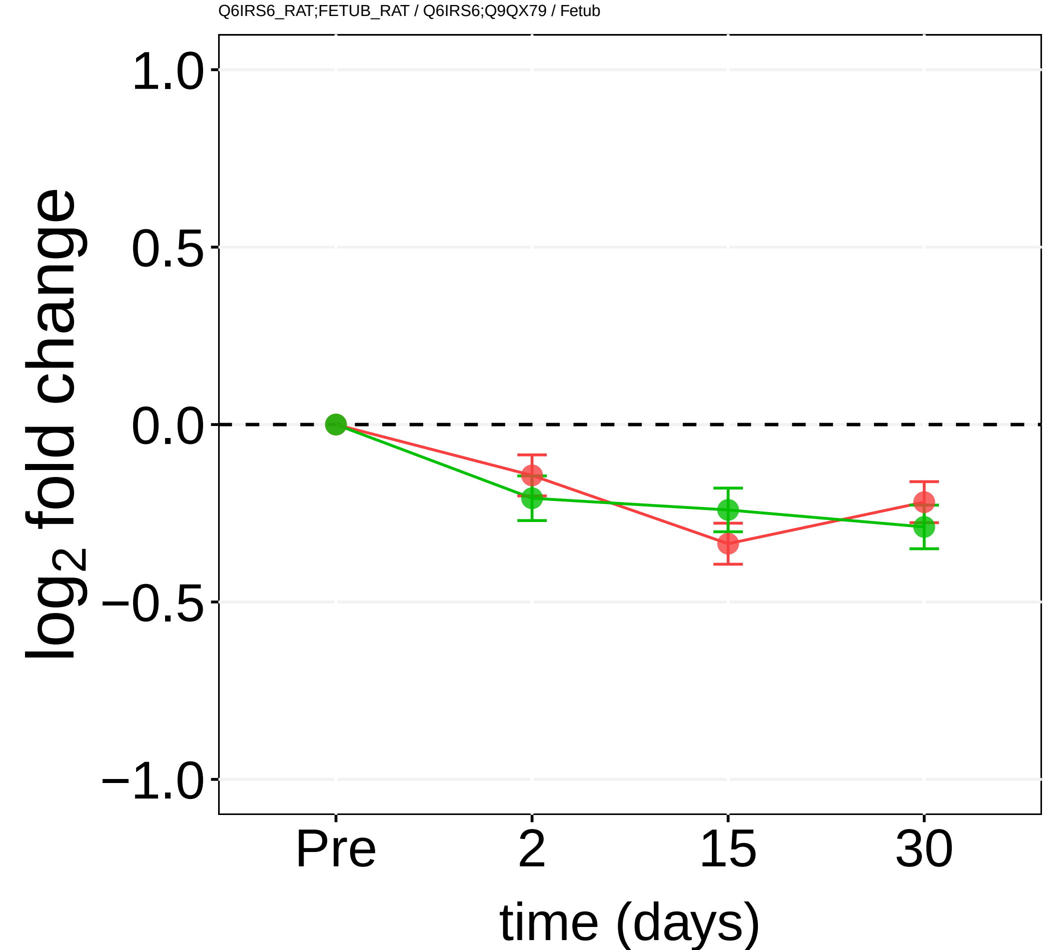

Q6IRS6_RAT;FETUB_RAT / Q6IRS6;Q9QX79 / Fetub
1.0
0.5
0.0
−0.5
−1.0
log2 fold change
Pre
2
15
30
time (days)

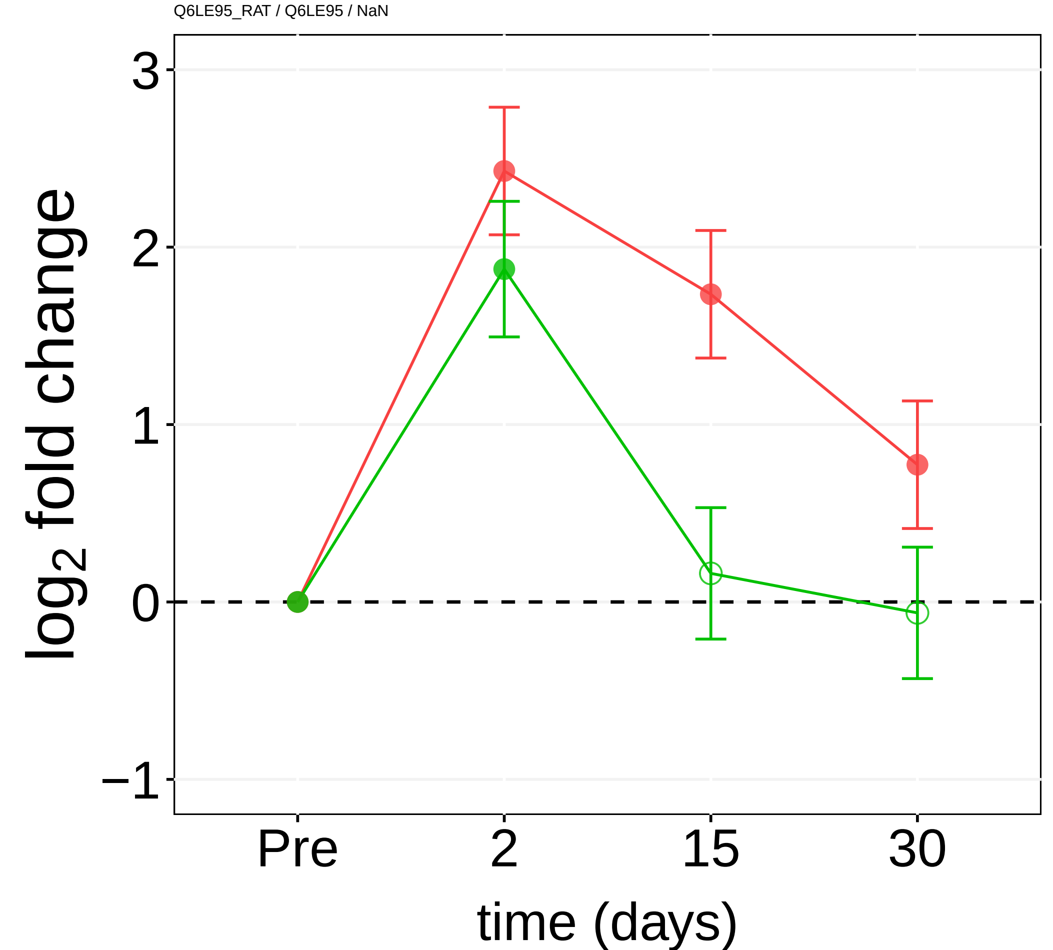
Q6LE95_RAT / Q6LE95 / NaN
log2 fold change
3
2
1
0
−1
Pre
2
15
30
time (days)

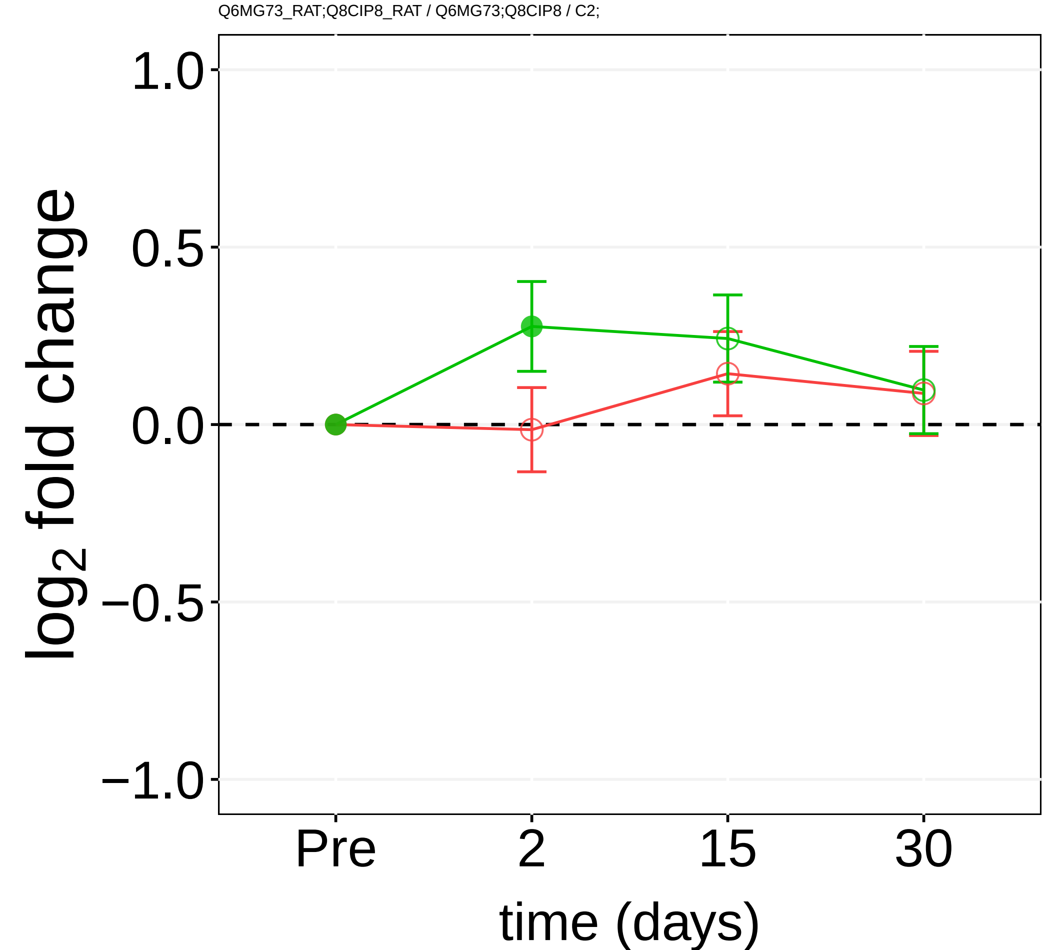
Q6MG73_RAT;Q8CIP8_RAT / Q6MG73;Q8CIP8 / C2;
1.0
0.5
0.0
−0.5
−1.0
log2 fold change
Pre
2
15
30
time (days)

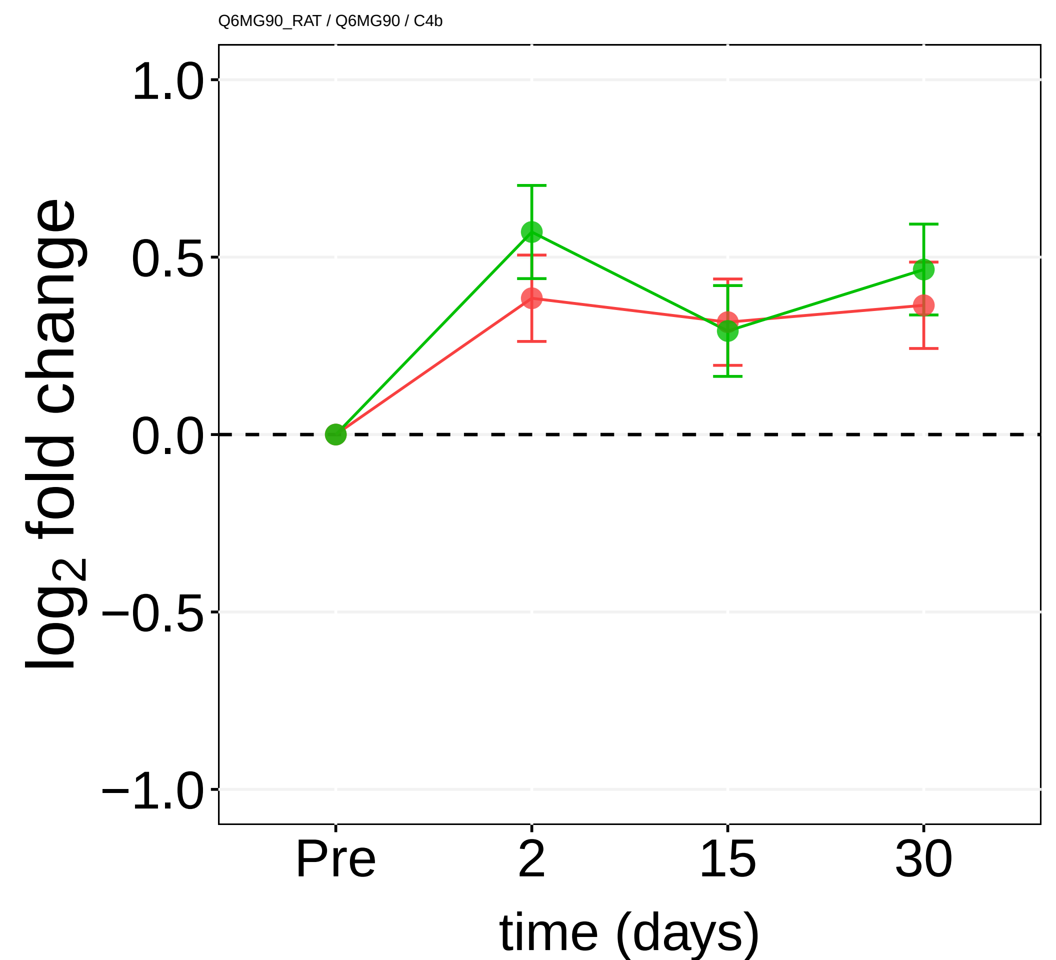

Q6MG90_RAT / Q6MG90 / C4b
log2 fold change
1.0
0.5
0.0
−0.5
−1.0
Pre
2
15
30
time (days)

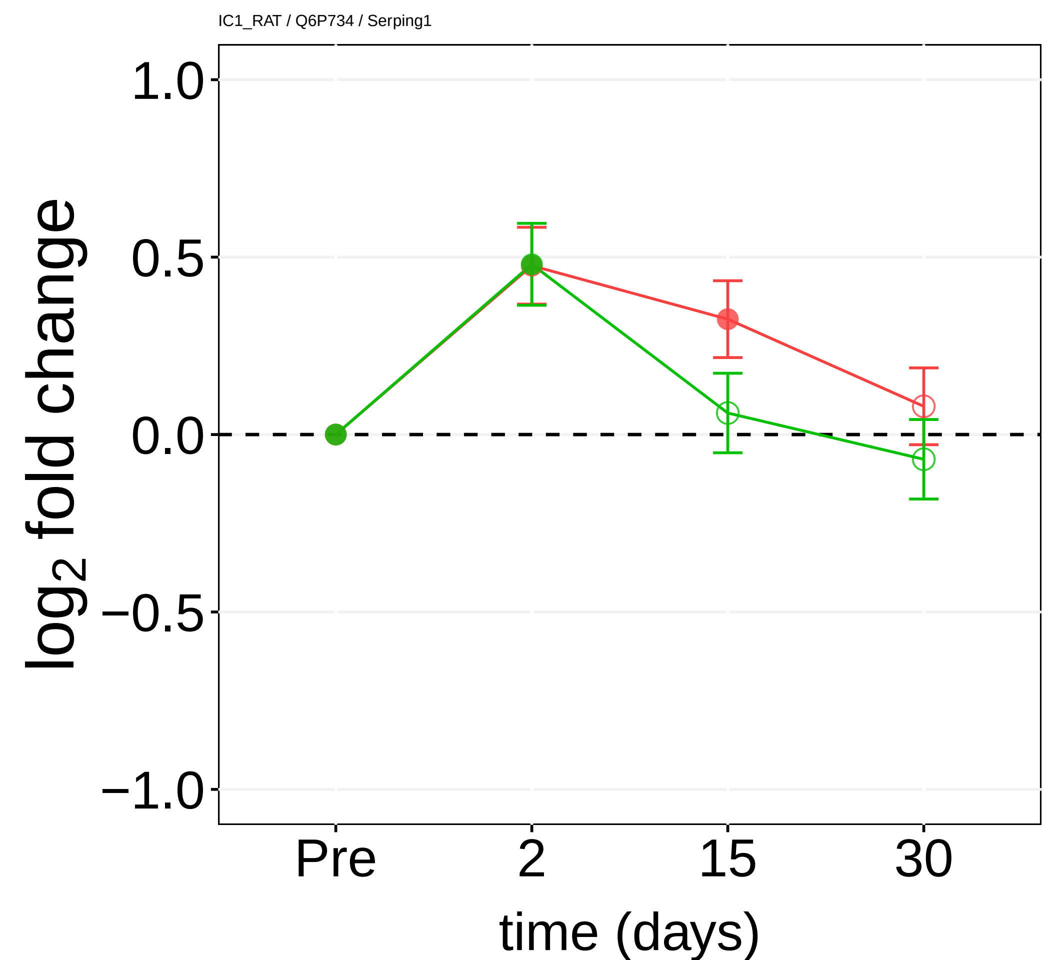
IC1_RAT / Q6P734 / Serping1
1.0
0.5
0.0
−0.5
−1.0
log2 fold change
Pre
2
15
30
time (days)

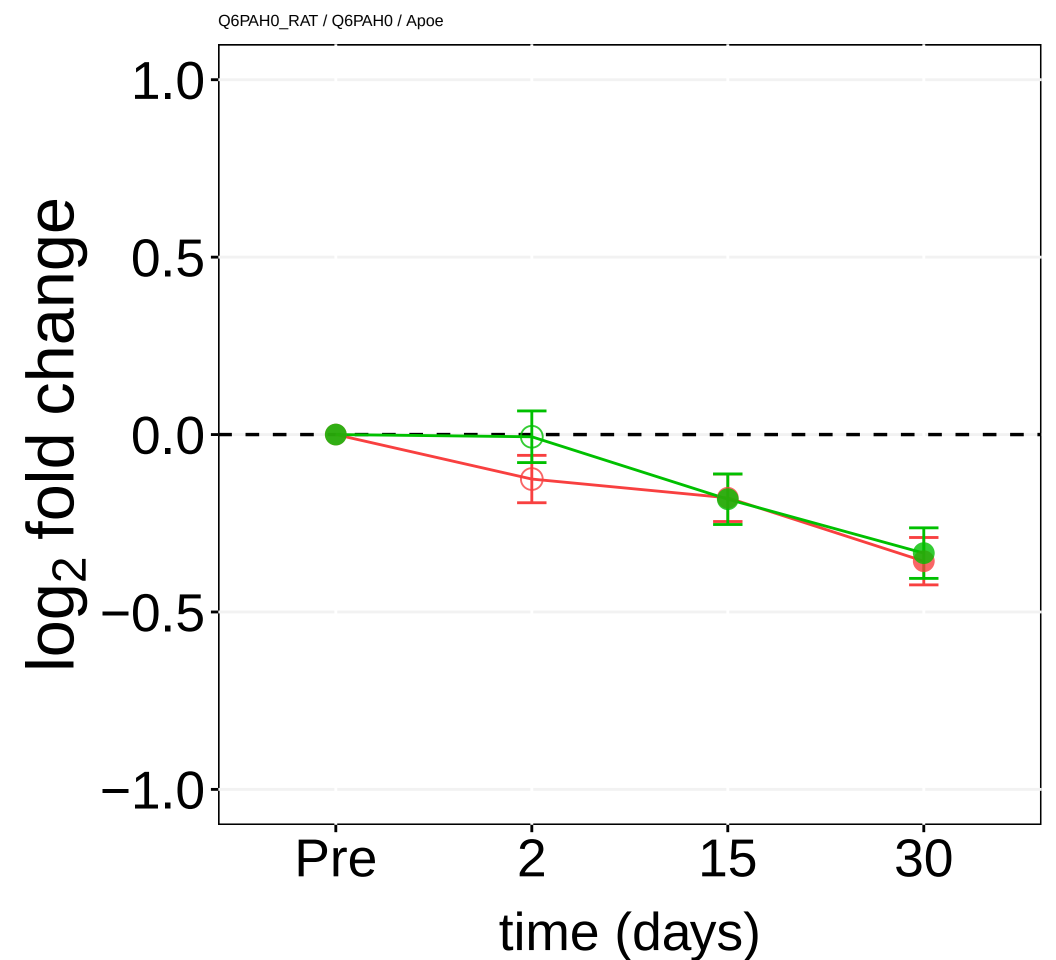
Q6PAH0_RAT / Q6PAH0 / Apoe
1.0
0.5
0.0
−0.5
−1.0
log2 fold change
Pre
2
15
30
time (days)

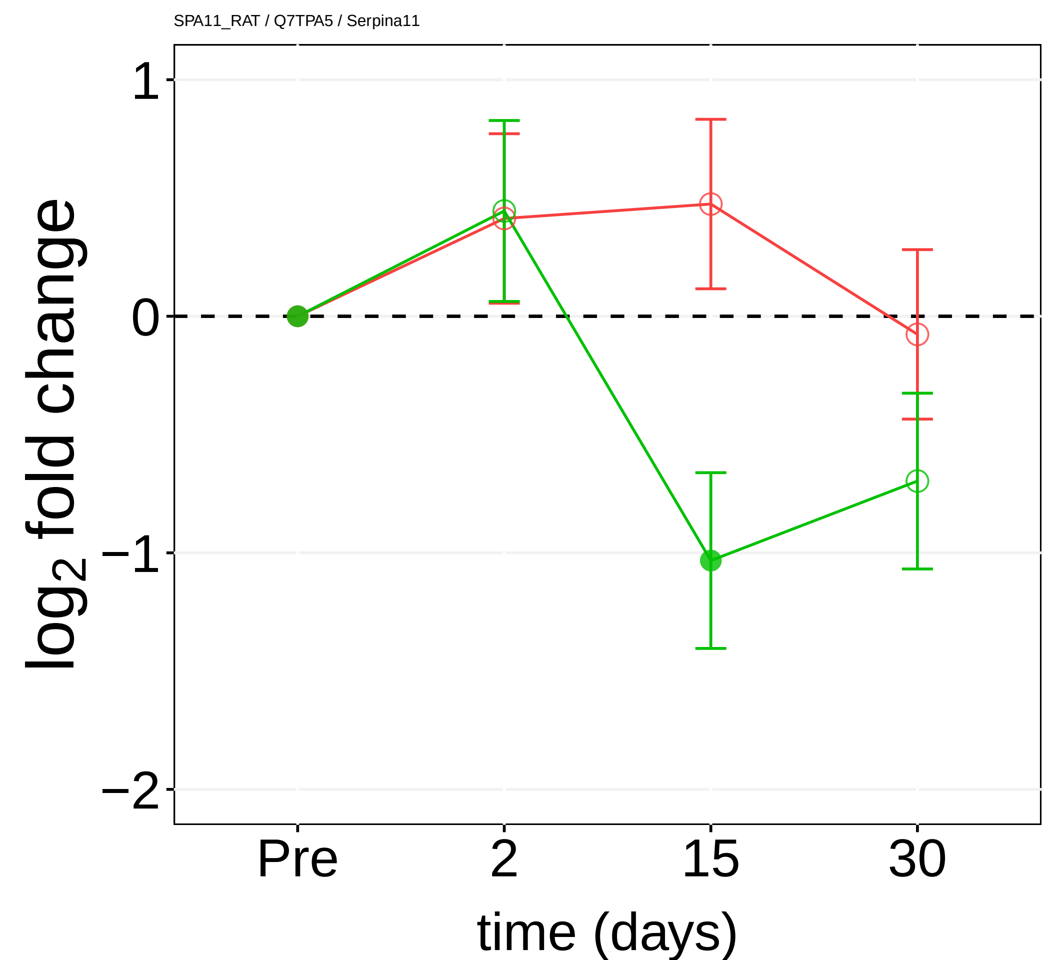
SPA11_RAT / Q7TPA5 / Serpina11
1
0
−1
−2
log2 fold change
Pre
2
15
30
time (days)

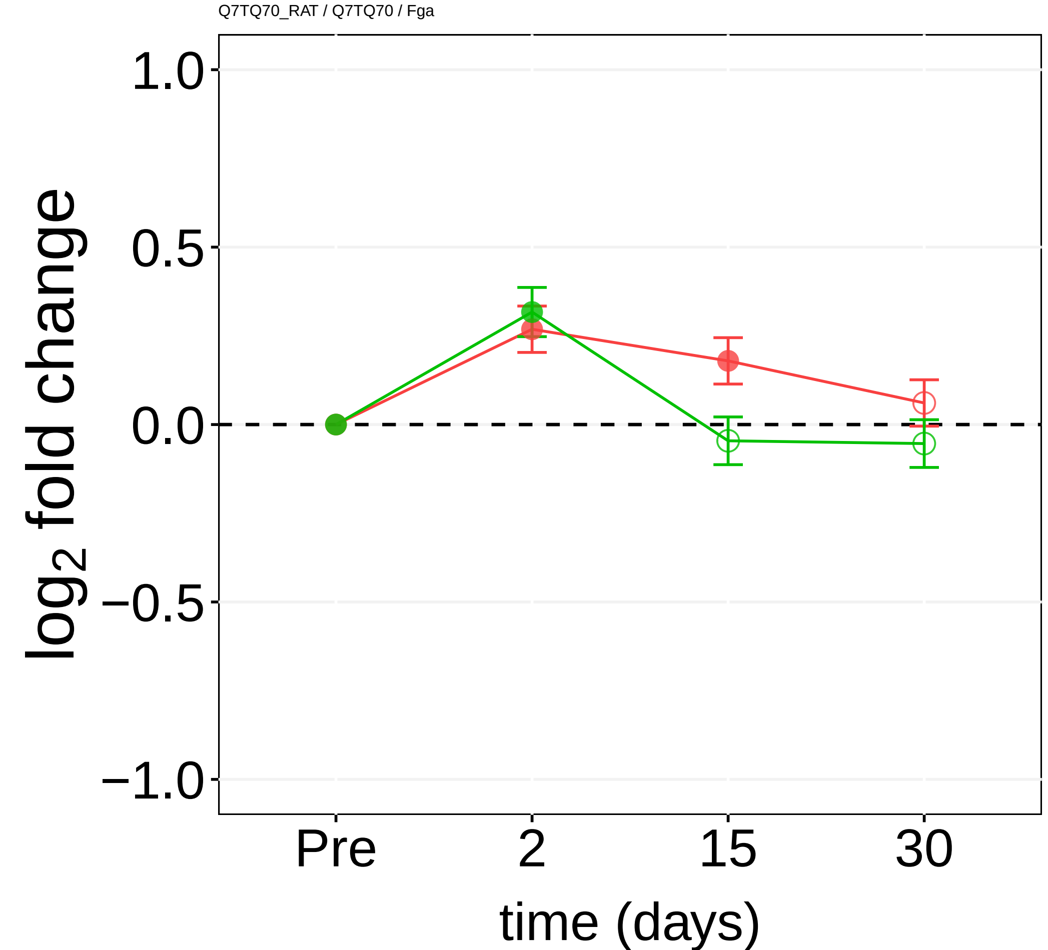

Q7TQ70_RAT / Q7TQ70 / Fga
1.0
0.5
0.0
−0.5
−1.0
log2 fold change
Pre
2
15
30
time (days)

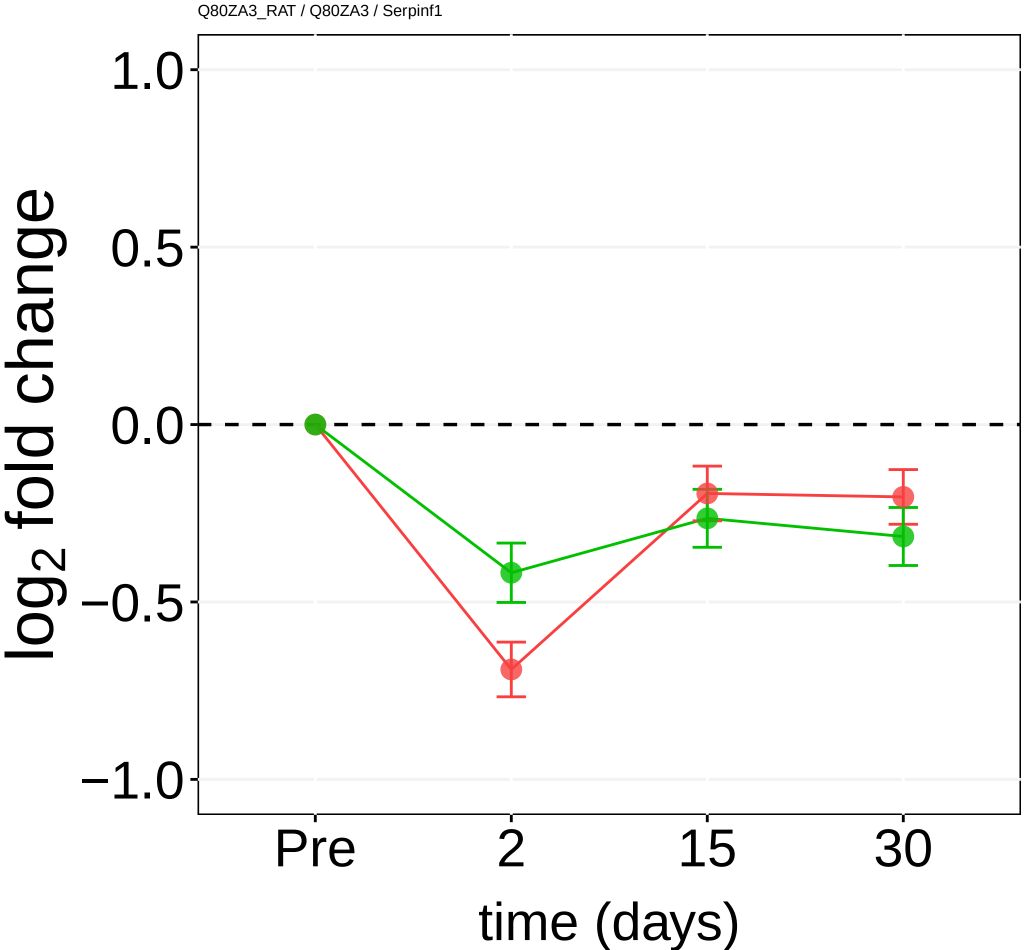

Q80ZA3_RAT / Q80ZA3 / Serpinf1
log2 fold change
1.0
0.5
0.0
−0.5
−1.0
Pre
2
15
30
time (days)

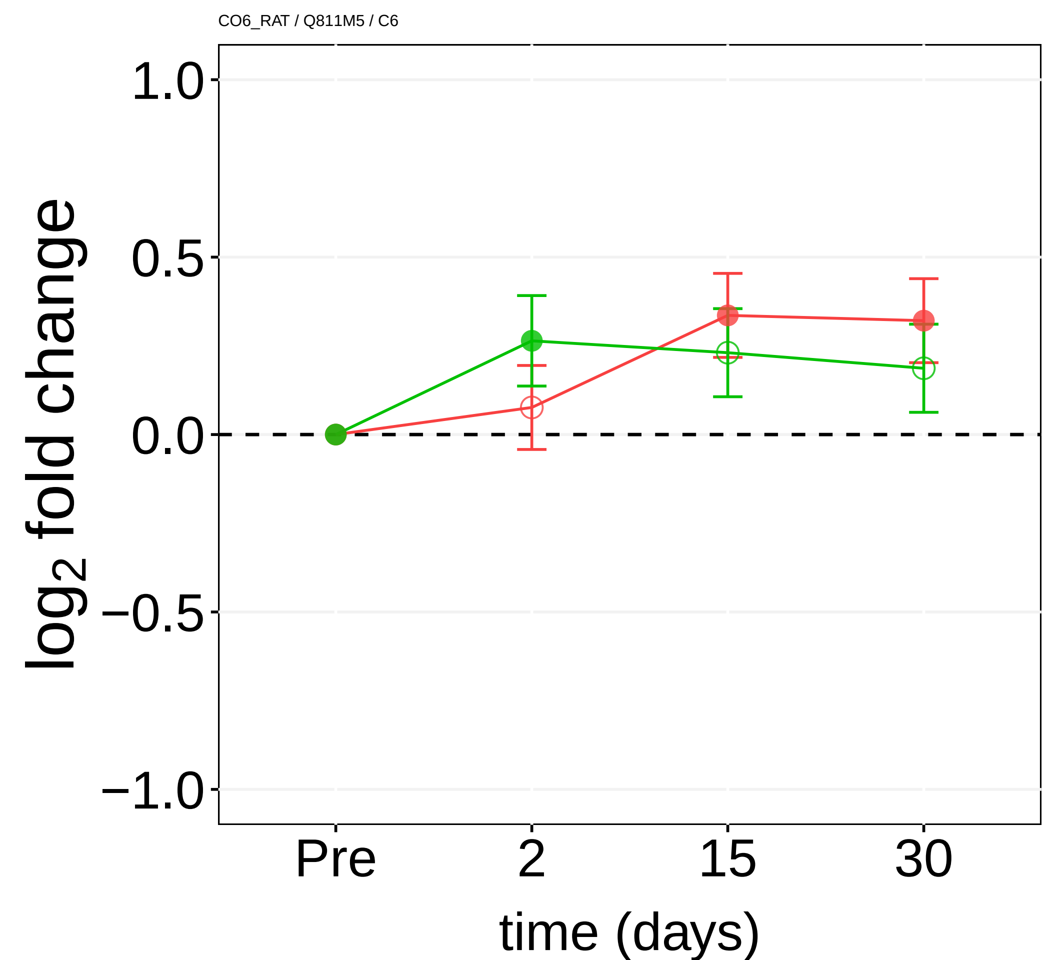
CO6_RAT / Q811M5 / C6
1.0
0.5
0.0
−0.5
−1.0
log2 fold change
Pre
2
15
30
time (days)

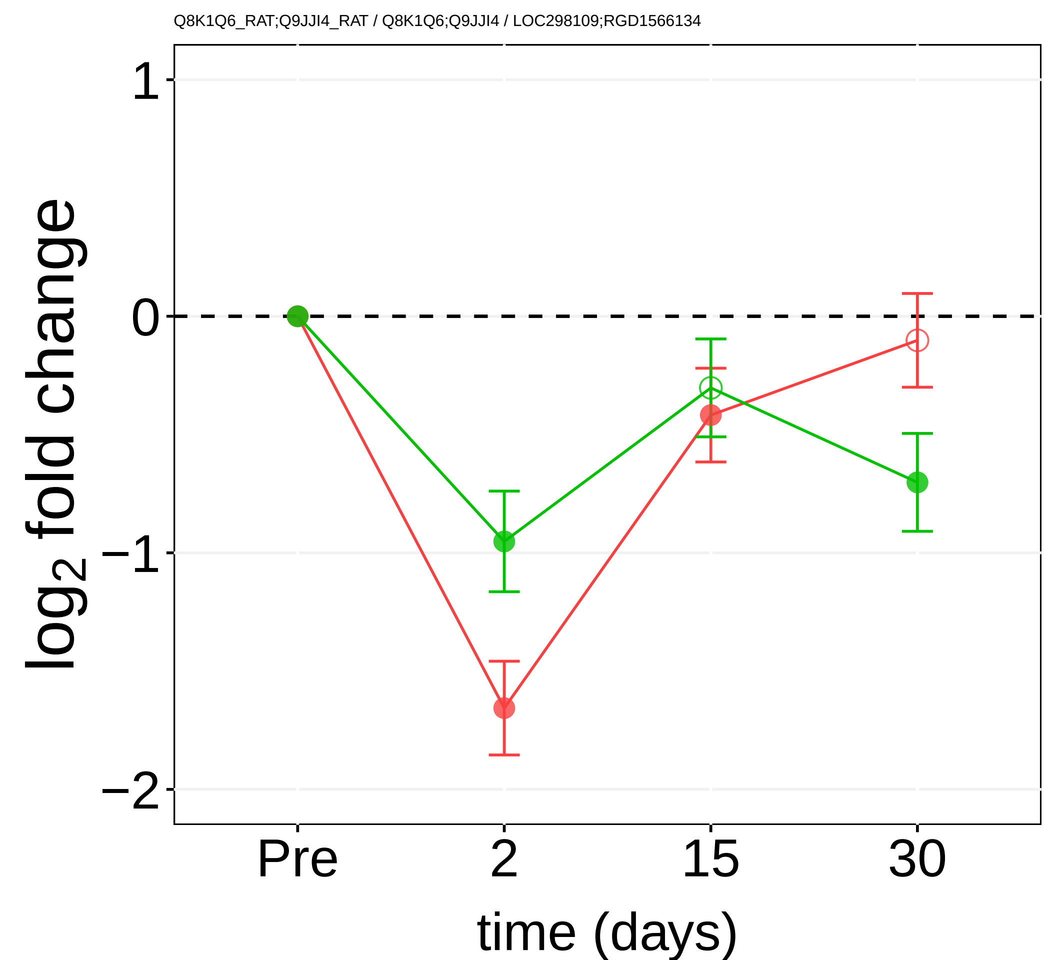

Q8K1Q6_RAT;Q9JJI4_RAT / Q8K1Q6;Q9JJI4 / LOC298109;RGD1566134
1
0
−1
−2
log2 fold change
Pre
2
15
30
time (days)

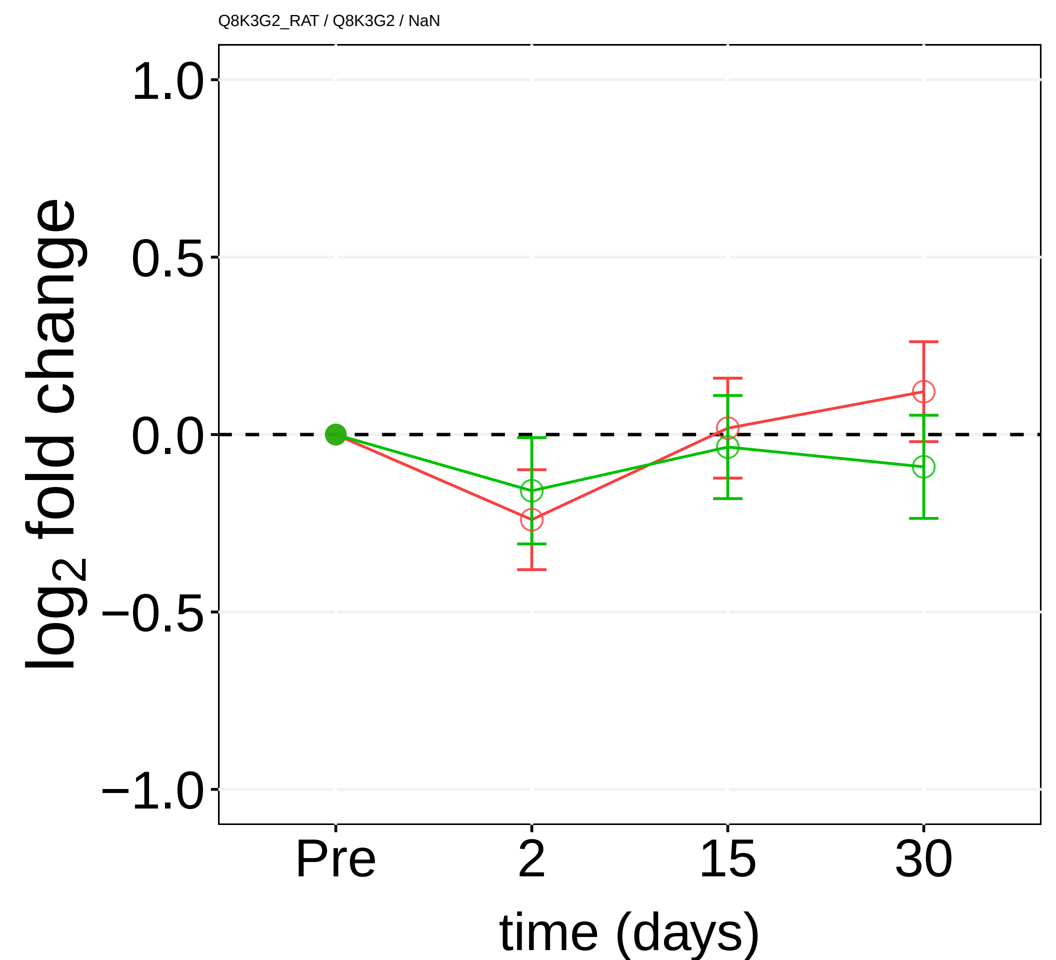
Q8K3G2_RAT / Q8K3G2 / NaN
1.0
0.5
0.0
−0.5
−1.0
log2 fold change
Pre
2
15
30
time (days)

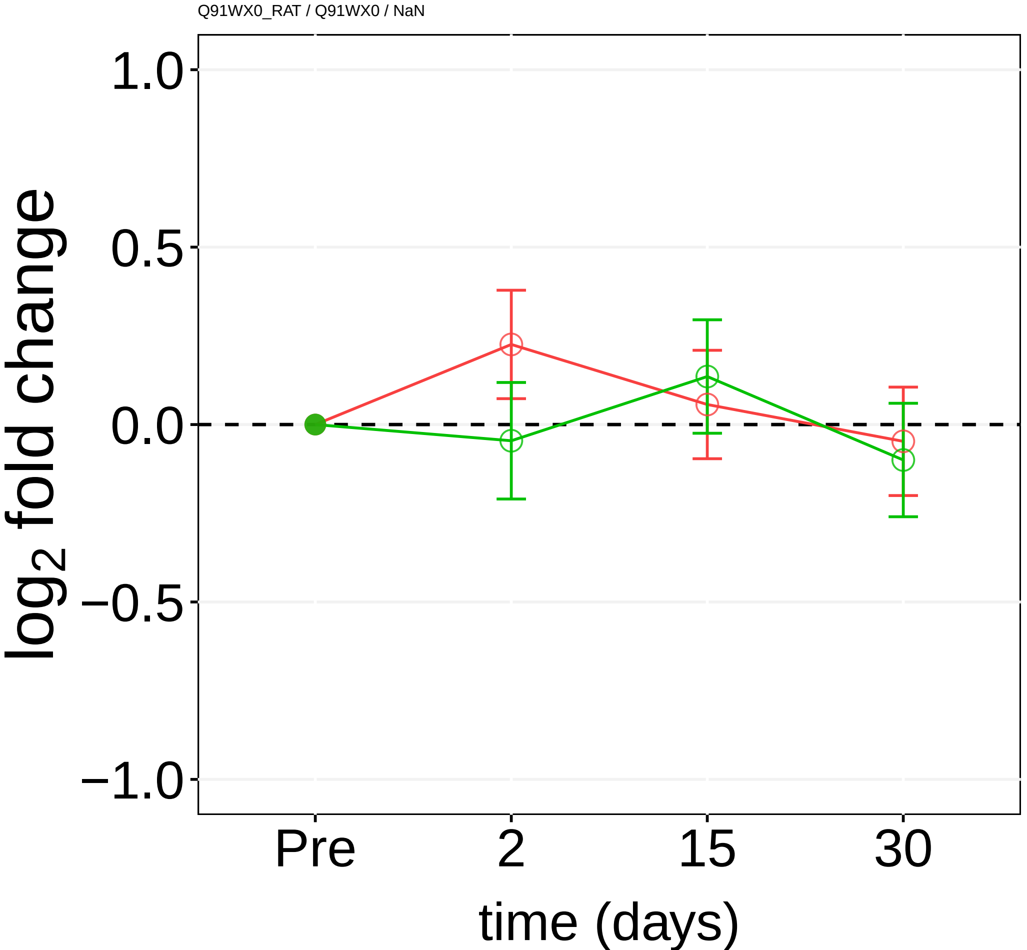

Q91WX0_RAT / Q91WX0 / NaN
1.0
0.5
0.0
−0.5
−1.0
log2 fold change
Pre
2
15
30
time (days)

Q91YB6_RAT / Q91YB6 / Cfh

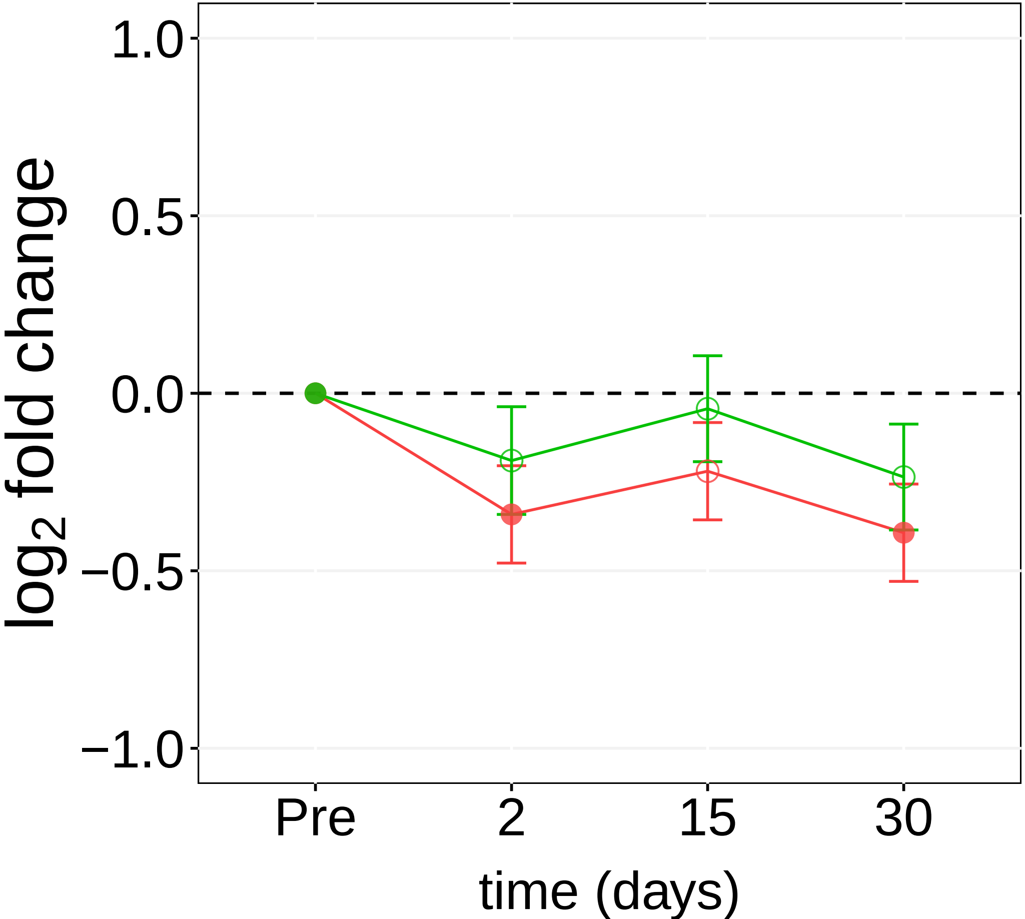

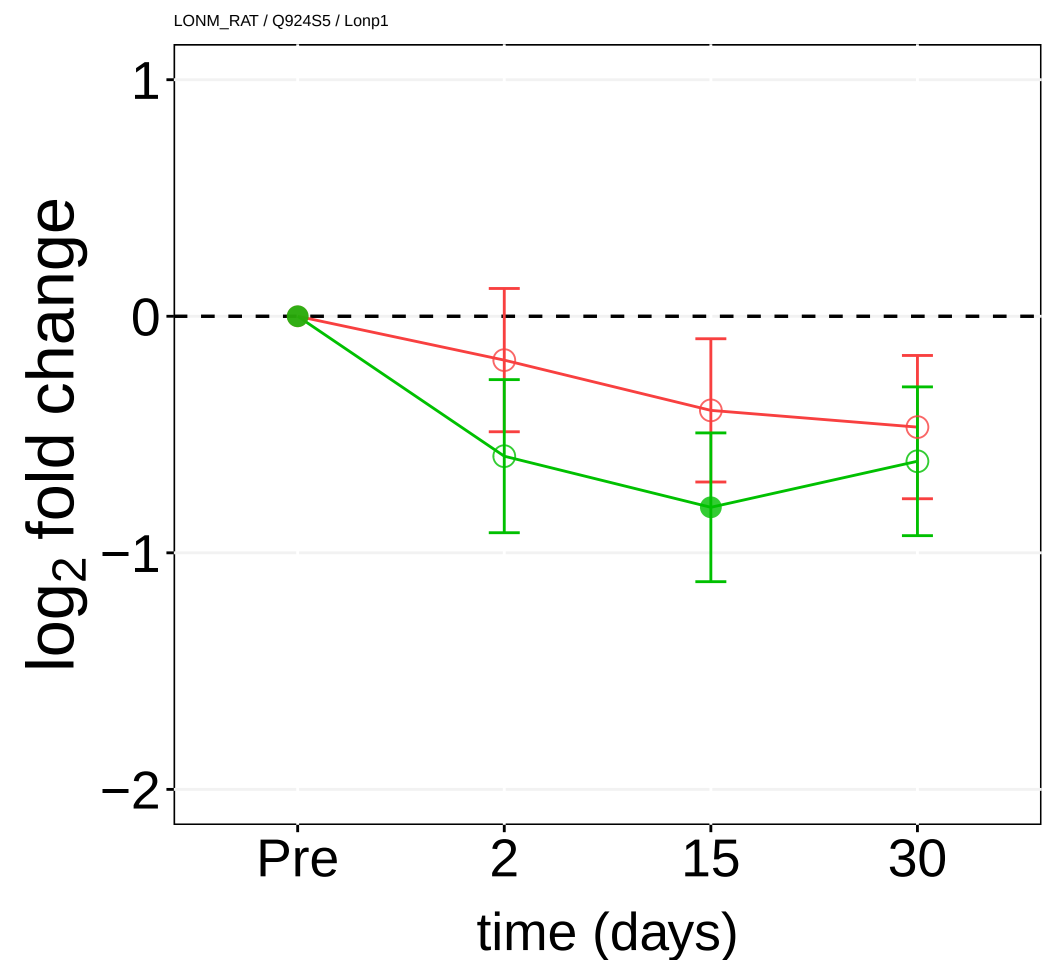
LONM_RAT / Q924S5 / Lonp1
1
0
−1
−2
log2 fold change
Pre
2
15
30
time (days)

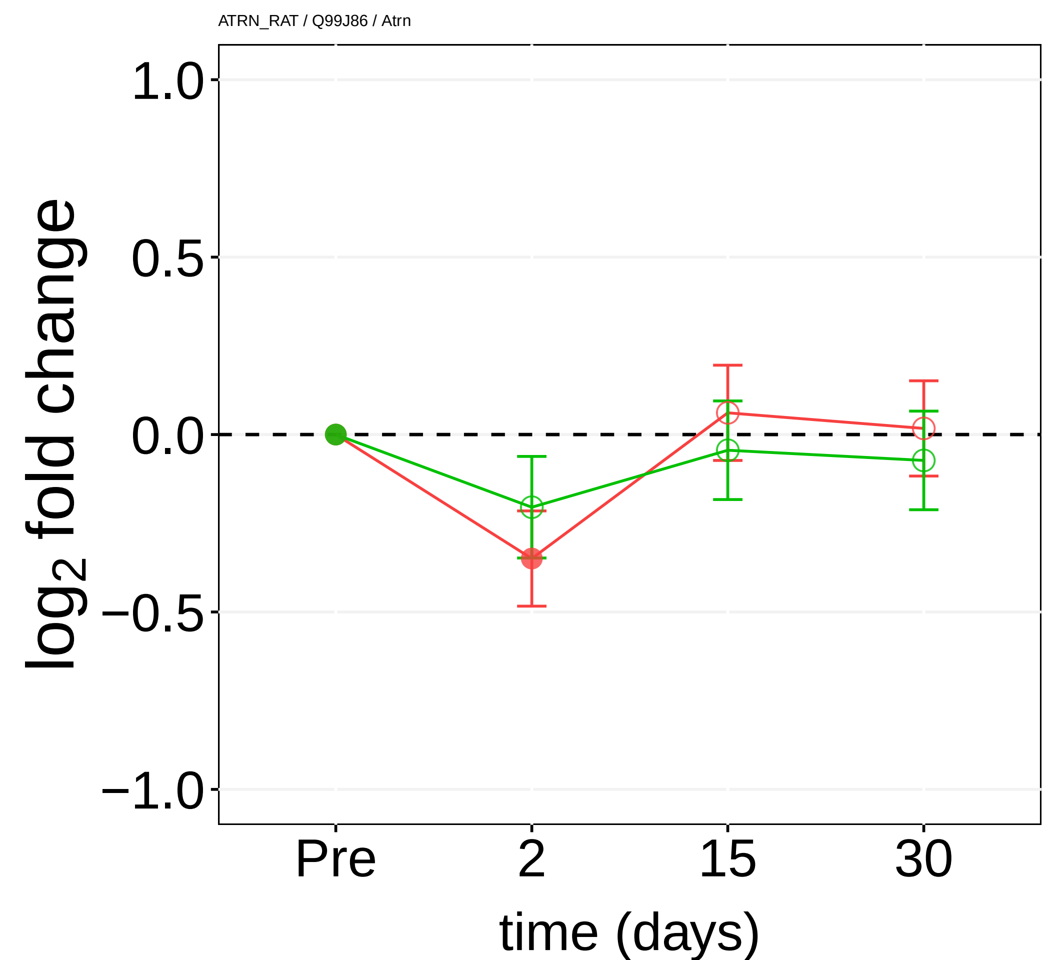

ATRN_RAT / Q99J86 / Atrn
log2 fold change
1.0
0.5
0.0
−0.5
−1.0
Pre
2
15
30
time (days)

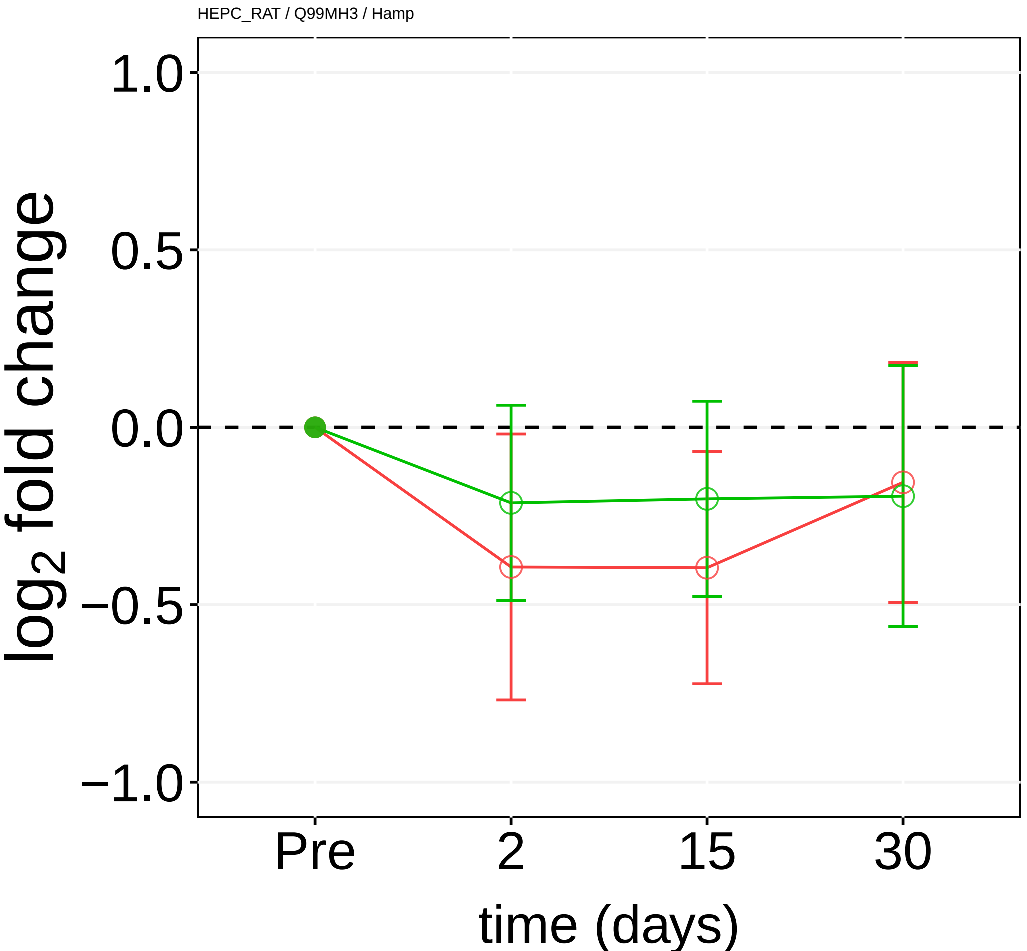

HEPC_RAT / Q99MH3 / Hamp
1.0
0.5
0.0
−0.5
−1.0
log2 fold change
Pre
2
15
30
time (days)

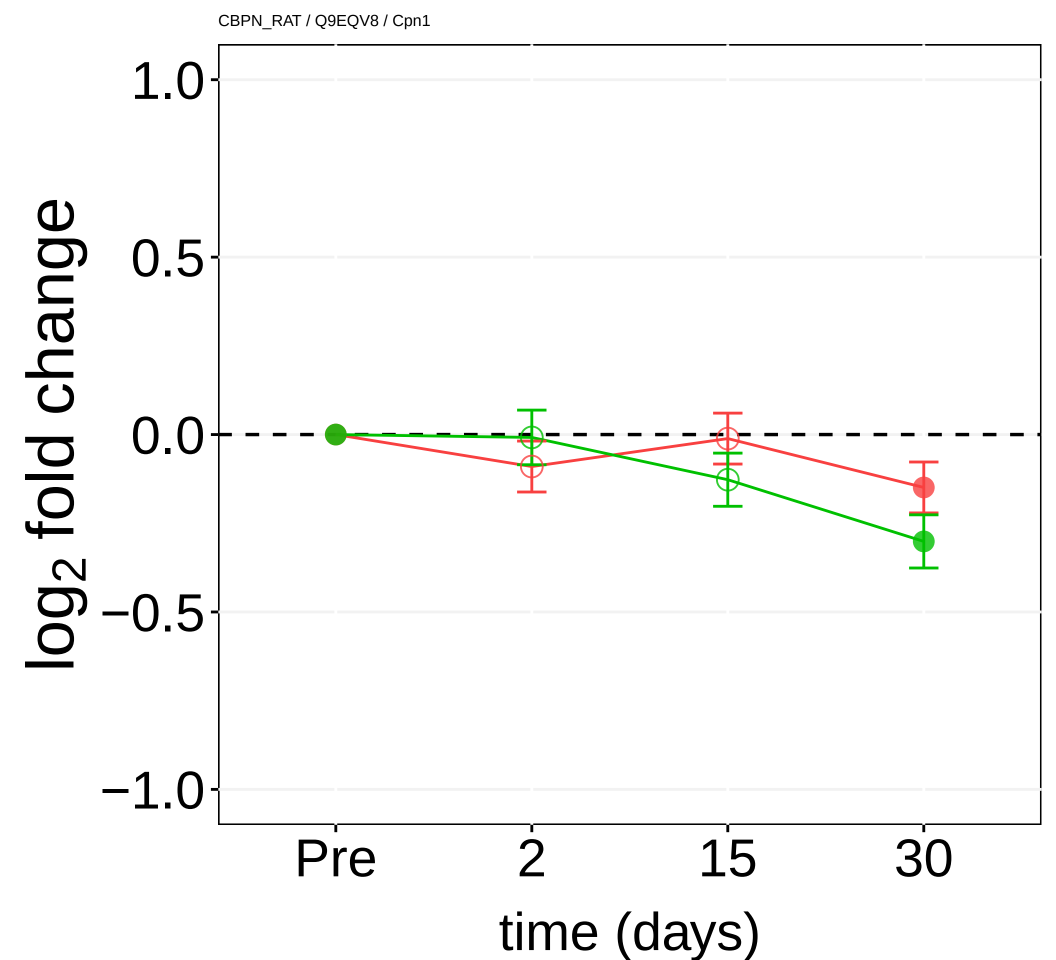
CBPN_RAT / Q9EQV8 / Cpn1
1.0
0.5
0.0
−0.5
−1.0
log2 fold change
Pre
2
15
30
time (days)

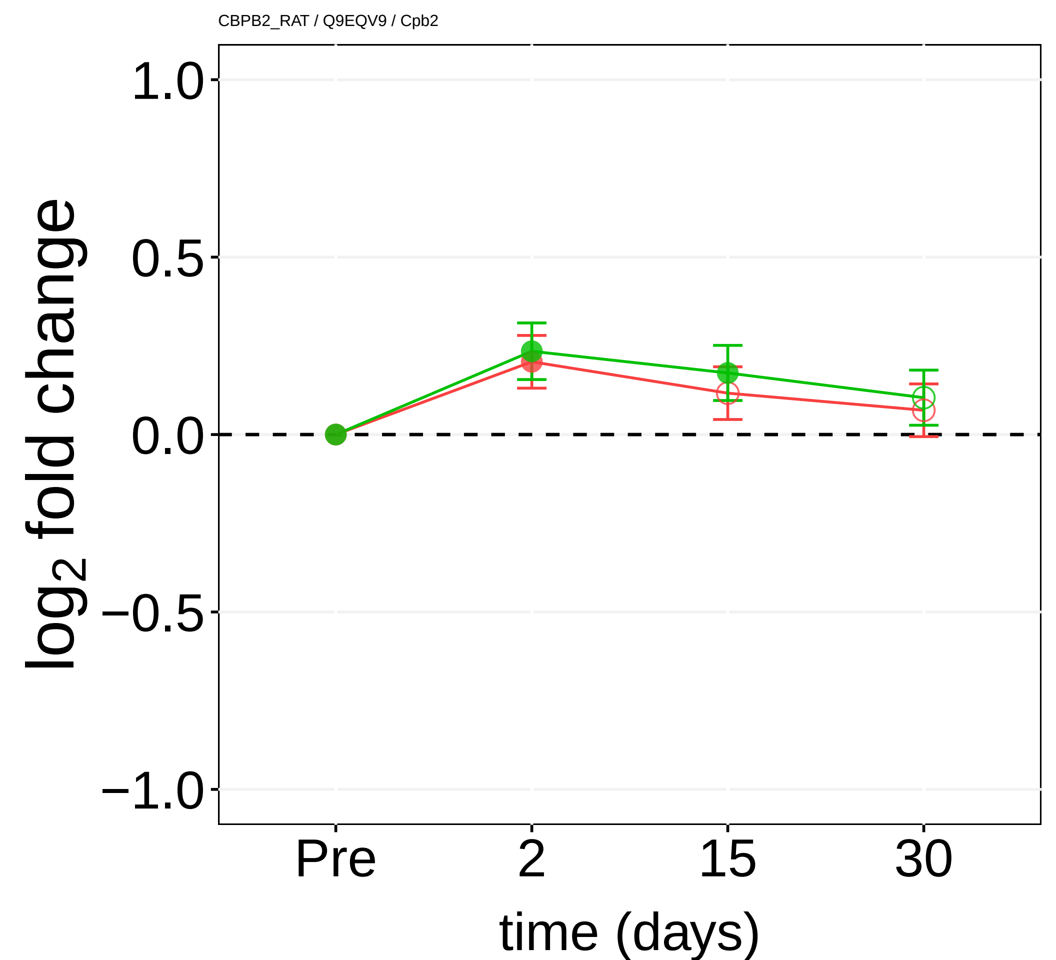

CBPB2_RAT / Q9EQV9 / Cpb2
1.0
0.5
0.0
−0.5
−1.0
log2 fold change
Pre
2
15
30
time (days)

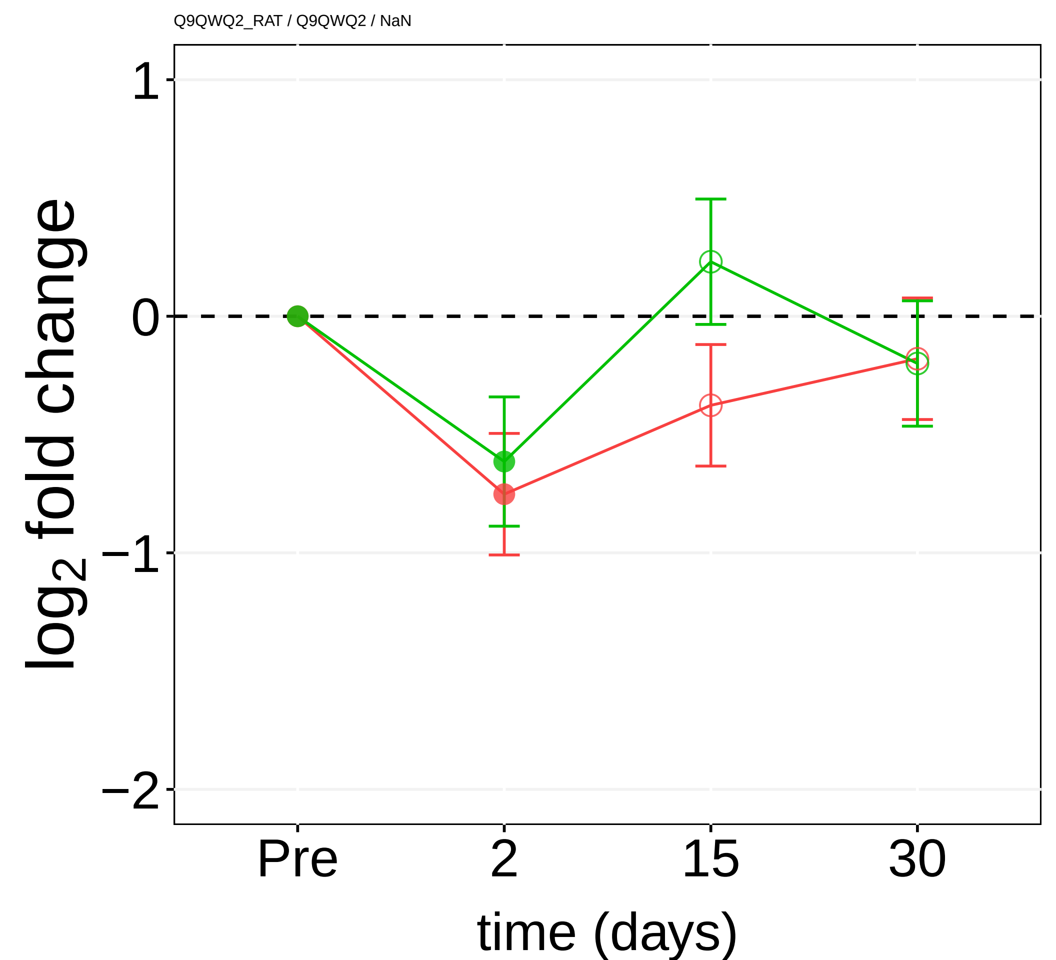
Q9QWQ2_RAT / Q9QWQ2 / NaN
log2 fold change
1
0
−1
−2
Pre
2
15
30
time (days)

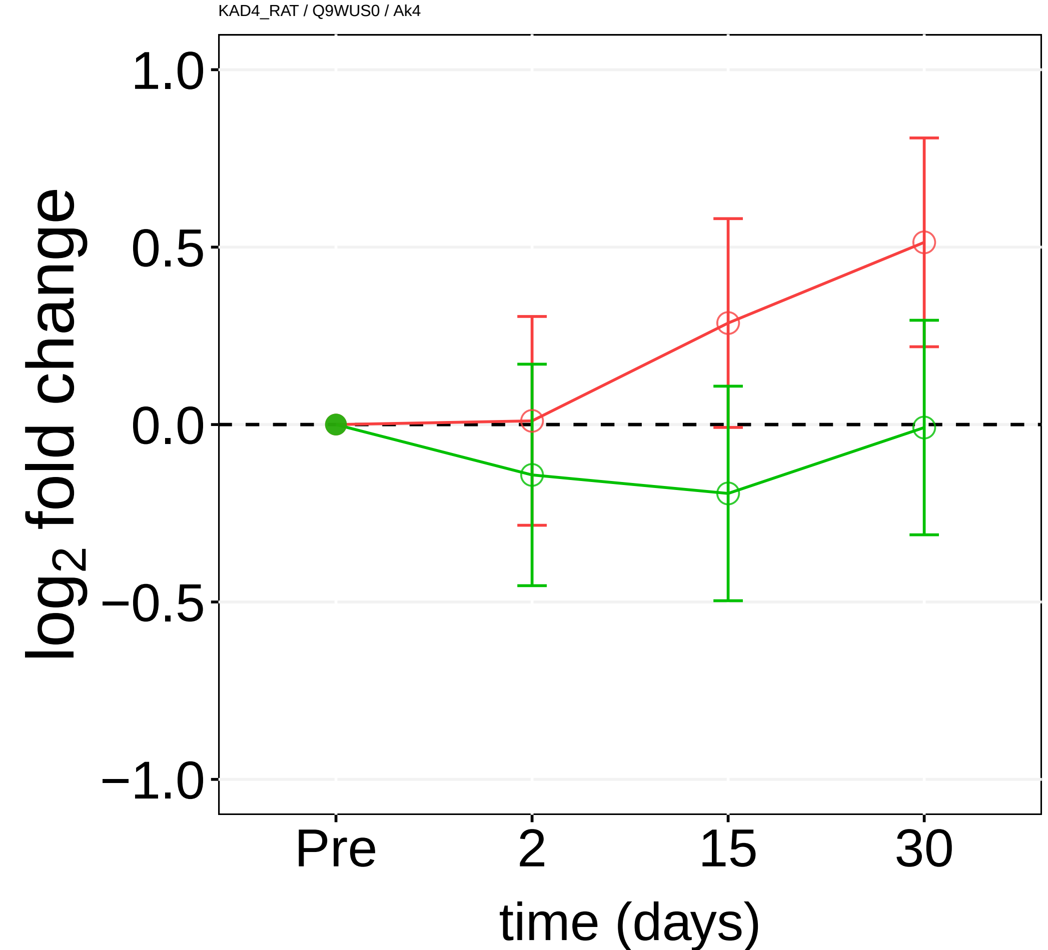
KAD4_RAT / Q9WUS0 / Ak4
1.0
0.5
0.0
−0.5
−1.0
log2 fold change
Pre
2
15
30
time (days)
